# Evolution of SL-RNA Genes and Their Splicing Targets in Parasitic Flatworms

**DOI:** 10.1093/molbev/msaf228

**Published:** 2025-09-23

**Authors:** Javier Calvelo, Héctor Musto, Uriel Koziol, Andrés Iriarte

**Affiliations:** Laboratorio Biología Computacional, Unidad Académica Desarrollo Biotecnológico, Instituto de Higiene, Facultad de Medicina, Universidad de la República, Av. Alfredo Navarro 3051, CP11600 Montevideo, Uruguay; Unidad de Genómica Evolutiva, Facultad de Ciencias, Universidad de la República, Iguá 4225, CP11400 Montevideo, Uruguay; Sección Biología Celular, Facultad de Ciencias, Universidad de la República, Iguá 4225, CP11400 Montevideo, Uruguay; Laboratorio Biología Computacional, Unidad Académica Desarrollo Biotecnológico, Instituto de Higiene, Facultad de Medicina, Universidad de la República, Av. Alfredo Navarro 3051, CP11600 Montevideo, Uruguay

**Keywords:** Spliced leader trans-splicing, operons, comparative genomics of platyhelminthes, cestodes, concerted evolution

## Abstract

Spliced leader (SL) trans-splicing is a key step in the processing of many mRNAs in different eukaryotic lineages, including in parasitic flatworms. Despite its importance, efforts for its characterization in this phylum have remained a collection of single-species studies with little exploration at a wider phylogenetic context. In this work, we present a comprehensive analysis of this process, based on the available genomic and transcriptomic data of 24 cestode and trematode species, including the identification of the SL-RNA sequences and their splicing acceptor transcripts and sites. We identified a main pattern of concerted evolution of SL-RNA loci in most flatworm species, as well as divergence of SL-RNA loci in selected species. However, even in species with several divergent SL-RNAs, there was no specialization in their targets. This, along with low SL trans-splicing levels, is in stark contrast with the global patterns of SL trans-splicing usage in nematodes. SL trans-splicing could be detected for a limited number of mRNAs in all species (<31%), and we found extensive use of the same splice acceptor sites for cis-splicing, especially for monocistronic transcripts. Ancestral SL trans-splicing sites are found in many conserved genes, including in putative ancestral operons that are shared between cestodes and trematodes.

## Graphical Abstract

**Graphical Abstract:**
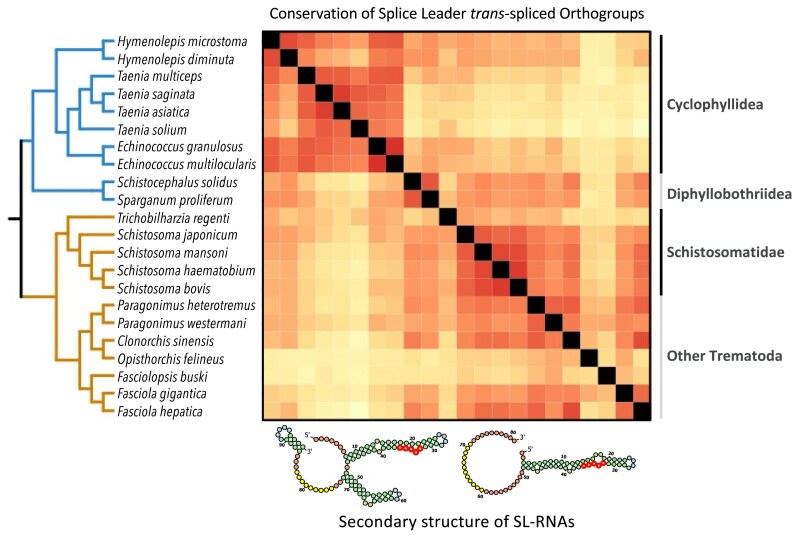

## Introduction

Spliced leader (SL) trans-splicing is a form of splicing between two RNA molecules, a specialized short RNA named spliced leader RNA (SL-RNA) and a pre-mRNA, to form a mature mRNA. In this process, the sequence of the mRNA precursor upstream of the splicing site (outron) is replaced by a SL sequence typically containing a 5′ trimethylguanosine cap. In many eukaryotes that contain operons, SL trans-splicing represents a key molecular mechanism behind the resolution of polycistronic transcripts into individually capped monocistronic mRNAs (Hastings 2005; Douris et al. 2010; Lasda and Blumenthal 2011; Bitar et al. 2013). In addition to participating in polycistron resolution, this mechanism can also result in the removal of deleterious sequences located in the 5′ UTR (Hastings 2005; Lasda and Blumenthal 2011; Bitar et al. 2013) or as the source of alternative mRNA isoforms (eg Brehm et al. 2000; Agorio et al. 2003; Siegel et al. 2011).

The distribution of SL trans-splicing in the eukaryotic phylogenetic tree is dispersed, as this mechanism is absent in many large clades, including vertebrates. Furthermore, the extent of SL trans-splicing is variable in different groups in which the mechanism is present. For example, in trypanosomatids almost all coding genes are found in operons and require SL trans-splicing for expression (Michaeli 2011), and in the nematode *Caenorhabditis elegans*, SL trans*-*splicing occurs in almost all mRNAs, even though only a fraction of its genes are organized in operons (Allen et al. 2011; Bernard et al. 2023). In contrast, in parasitic flatworms, only a fraction of mRNAs are targeted by trans-splicing (estimated to be between 11% and 47%, in different species, using different methodologies; Boroni et al. 2018; Brehm et al. 2000; Ershov et al. 2019; Protasio et al. 2012; Tsai et al. 2013), including not only genes organized in operons but also many monocistronic genes. The process has also been described in the free-living flatworms *Schmidtea mediterranea* (Zayas et al. 2005; Rossi et al. 2014) and *Macrostomum lignano* (Ustyantsev and Berezikov 2021). In the latter, a single SL sequence was identified, with approximately 30% of its genes showing evidence of trans-splicing and around 15% of all genes organized in operons.

In some eukaryotes that have several different SL-RNA genes, there is evidence of their specialization. In *C. elegans*, different SL types are specialized for particular targets, commonly referred to as SL1 (targeted to the first genes in operons and to monocistronic genes) and SL2 (involved in the resolution of polycistronic transcripts by targeting the downstream genes; Lasda and Blumenthal 2011; Blumenthal 2018). Other examples include the flatworm *S. mediterranea*, in which a particular SL-RNA is enriched in a subpopulation of stem cells (Rossi et al. 2014), and *Parvilucifera sinerae*, a parasitoid dinoflagellate displaying clear segregation in the splicing of different SL sequences depending on the function of the acceptor mRNAs (Alacid et al. 2022).

Despite the crucial roles of SL trans-splicing, its evolutionary origin remains uncertain (Sather and Agabian 1985; Krause and Hirsh 1987; Rajkovic et al. 1990; Tessier et al. 1991; Ross et al. 1995; Brehm et al. 2000; Vandenberghe et al. 2001; Steele et al. 2004; Pouchkina-Stantcheva and Tunnacliffe 2005; Lidie and Van Dolah 2007; Marlétaz et al. 2008; Douris et al. 2010; Matsuo et al. 2018). Its scattered phylogenetic distribution suggests a complex history of independent origins and/or losses (Hastings 2005; Douris et al. 2010; Lasda and Blumenthal 2011; Bitar et al. 2013; Krchňáková et al. 2017). Furthermore, few comparative studies have been performed within phyla, and the evolutionary dynamics of trans-splicing gain and loss are not well understood (Hastings 2005; Douris et al. 2010; Bitar et al. 2013; Wenzel et al. 2020). A significant roadblock in this field is the low conservation of the SL sequence at large phylogenetic scales, which makes SL-RNA identification nontrivial (Hastings 2005; Douris et al. 2010; Lasda et al. 2010; Bitar et al. 2013). While some efforts have been made to facilitate the identification of novel SL sequences (Radío et al. 2018; Yague-sanz and Hermand 2018; Calvelo et al. 2020; Wenzel et al. 2021), comprehensive studies involving multiple species remain scarce. The primary sequences of SL-RNAs from distant clades do not show any similarity except for small motifs including a site of interaction with Sm spliceosomal proteins (Sm motif). However, their sequences can be partially conserved within the same clade (Bitar et al. 2013), and most of them share the same overall features (Hastings 2005; Douris et al. 2010; Lasda et al. 2010; Bitar et al. 2013). These include a leader region that is incorporated into the pre-mRNA and an intron-like region with the Sm motif, both separated by a canonical splicing donor site. In addition, their secondary structure typically displays two hairpins surrounding the Sm motif, which has been proposed as a criterion for SL-RNA prediction (Wenzel et al. 2021), although exceptions have been reported (Zhang et al. 2007; Alacid et al. 2022). Identifying the SL-RNA sequences and the repertoire of mRNAs subjected to this form of trans-splicing is key to understanding the importance of this mechanism in different clades (Nilsson et al. 2010; Bitar et al. 2013; Islas-Flores et al. 2021) and provides crucial information to improve their genome annotation (Wenzel et al. 2020; Calvelo et al. 2023).

Because of the central role of trans-splicing in the expression of many different genes, it has been proposed as a potential drug target against parasites possessing this mechanism, particularly helminths (Pandarakalam et al. 2019). Here, we present for the first time a comprehensive approach to identify the SL-RNA complement and a catalog of trans-splicing acceptor genes throughout the parasitic flatworm groups Cestoda and Trematoda. Both groups are endoparasitic platyhelminthes (part of subphylum Neodermata) and include members that pose significant threats to human and animal health (Giri and Parija 2012; Mahmud et al. 2017; Webb and Cabada 2017). Several parasitic flatworm species have been shown to possess SL trans-splicing (Table S1), in some cases with several SL-RNAs present in each species (Tsai et al. 2013; Rossi et al. 2014; Olson et al. 2020; Calvelo et al. 2023), without clear evidence of SL-RNA specialization (Tsai et al. 2013; Olson et al. 2020; Calvelo et al. 2023). A key feature, however, is that all reported SLs for the group possess a 3′ terminal “ATG” (Cheng et al. 2006) that can potentially provide an alternative start codon for translation.

This study expands on previous work by offering a global view of SL trans-splicing in parasitic flatworms. Overall, their patterns of SL trans-splicing show important differences with those of well-characterized nematode species such as *C. elegans*, including relatively low global levels of trans-splicing, the absence of specialization of SL-RNAs, and extensive sharing of 3′ splice acceptor sites between trans- and cis-splicing. We observed the existence of shared SL trans-splicing acceptor sites (SL-ACEs) and operons within this diverse group, suggesting the conservation of ancestral patterns tracing back hundreds of millions of years.

## Results

### Candidate SL Identification and Selection

**Fig. 1.**
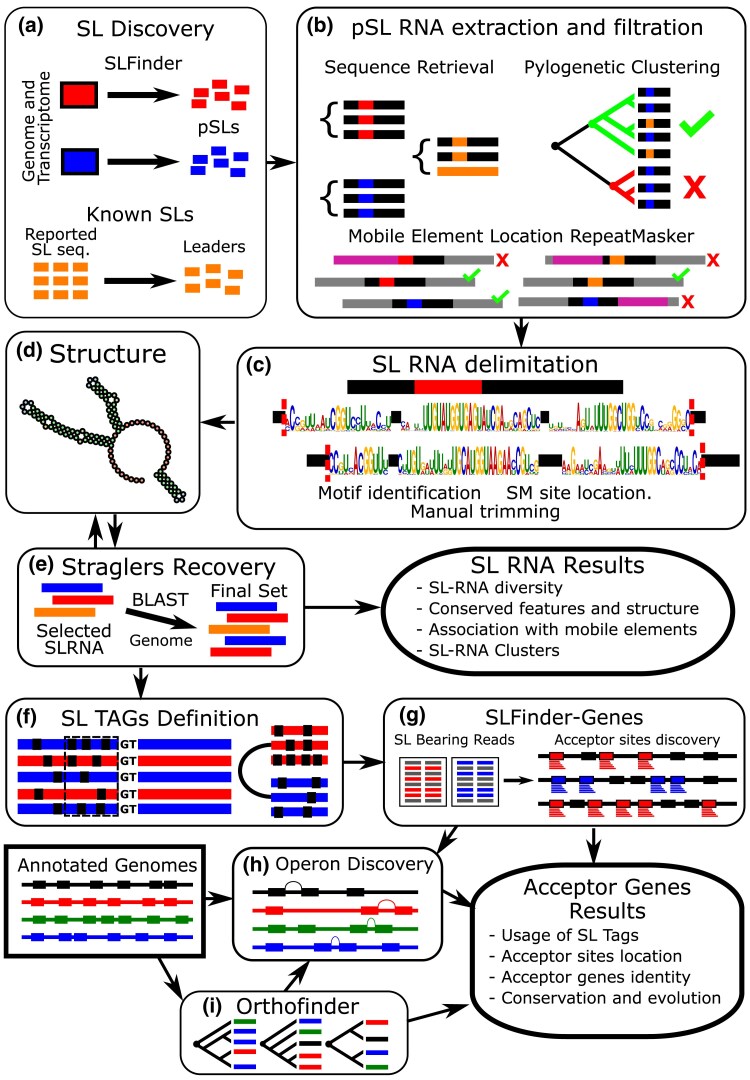
Workflow summary of the analysis pipeline. a) First, pSLs were identified, either de novo with the SLFinder pipeline or retrieved from the literature. Hits for their leader region were identified in the genome with BLASTn searches. b) The surrounding sequence of the candidate SL hits in the genome was retrieved, and potential SL-RNAs were identified by means of phylogenetic clustering based on sequence similarity. The absence of known repeated elements in proximity is set as a criterion for considering a potential SL-RNA. c) Conserved motifs were identified and used to isolate the SL-RNA from the surrounding genome sequence. Candidates whose conserved motifs appeared out of order relative to the majority were excluded, and the analysis was repeated. d) The SL-RNA secondary structure was predicted, and the final sequence SL-RNAs were defined based on shared folding structures that were present on at least one of the reference sequences and/or several other candidates (ie hairpins of comparable extension and location). e) To recover any stragglers that might have been missed in the initial analysis, an additional BLASTn search was conducted against the genomes using the identified SL-RNAs as queries. Hits were added to the pool if their secondary structure was conserved. f and g) SL tags, the last 15 bases of the leader region, were defined from the selected SL-RNAs and the reference sequences and used to identify SL-ACEs. h) In parallel, potential operons were identified based on the gene proximity, coding strand, and presence of SL-ACEs. i) The orthologous relationships between the annotated genes in the different genomes were determined, and potential conserved operons were identified.

Species of parasitic flatworms were selected based on the availability of a reference genome assembly and RNA-seq data (Table S2) and processed following the strategy described in Fig. 1. As a result, 24 species from the Trematoda and Cestoda classes were selected. The Monogenea class was excluded due to the lack of enough data available for comparative studies required for domain definition (see Materials and Methods). In short, our first step was to compile known SL-RNAs from the literature and to identify new SL-RNA candidates with SLFinder (Calvelo et al. 2020). The SLFinder pipeline identifies SL sequences by searching for overrepresented kmers at the ends of contigs of de novo transcriptome assemblies, followed by several automatic and manual filtering steps, including the identification of the SL-RNA loci with canonical splice donor sites in the corresponding genomic assemblies.

The initial combined dataset obtained from SLFinder and BLASTn searches comprised 1,179 candidate SL-RNA loci bearing a putative SL sequence. These were then filtered down to 312 SL-RNA loci across all species, guided by four sequential criteria: (i) true SL-RNAs share enough sequence similarity to be grouped together in a neighbor-joining phylogeny, clustering as a monophyletic group with known SL-RNA sequences, as defined using TreeCluster, (ii) true SL-RNA loci are not closely associated with mobile genetic elements (≤500 bp; see below), (iii) the mature portion of the SL-RNAs has specific conserved motifs in a specific order (as described below), and (iv) novel SL-RNAs share secondary structure elements commonly observed in known SL-RNAs, including a predicted hairpin containing the splice donor site and a hairpin found just before the Sm-like motif. These filtering procedures were necessary to differentiate true SL-RNAs from other types of loci that share the SL sequence, such as cases of reverse transcription of trans-spliced transcripts followed by genetic integration into the genome (Slamovits and Keeling 2008; Jaeckisch et al. 2011; Barnes et al. 2019). Lastly, a BLASTn search was conducted between the selected SL-RNAs and the genomes to search for additional SL-RNA loci that may have been missed in the initial search, followed by further filtering based on secondary structure analysis.

In particular, a large proportion of the potential SL-RNA loci that were discarded in our dataset were associated with repeated mobile elements (597 loci), primarily with LTR and LINE elements, which are the most common mobile genetic elements found in Platyhelminthes (Coghlan et al. 2019). The only identifiable SL-RNA feature in these loci was partial leader sequences. These loci may be the result of genomic integration of retrotransposons whose transcripts had received the SL sequence by trans-splicing.

### SL-RNA Sequence and Structural Conservation

A total of 71 nonredundant SL-RNA sequences were identified among the 312 SL-RNA loci in 24 flatworm species (denominated Unique_SL_1 to Unique_SL_71; File S1 and Tables S3 and S4), and an additional four unique sequences were found among previously reported full SL-RNAs (named Unique_SL_72 to Unique_SL_75). Two of these previously reported sequences correspond to species not analyzed in this study due to the lack of available genomic data (*Haematoloechus* sp. and *Stephanostomum* sp.). The other two sequences, from *Echinococcus granulosus* and *Schistosoma mansoni*, show minor differences compared to those found in current genomic assemblies. These could correspond to additional SL-RNA genes not found in the genomic assemblies or to small allelic differences between parasite isolates.

The estimated lengths of the identified SL-RNAs (78 to 103 bp) and of their SL regions (31 to 36 bp) are in line with other studies on SL trans-splicing in parasitic flatworms (Rajkovic et al 1990; Brehm et al. 2000; Ershov et al. 2019). Due to the filtering processes implemented in this study, their true lengths may be longer than these estimations, as the true 5′ end of the leader sequence is difficult to discern from RNA-seq reads, and the 3′ end of the SL-RNA may extend beyond the region of sequence conservation.

**Fig. 2.**
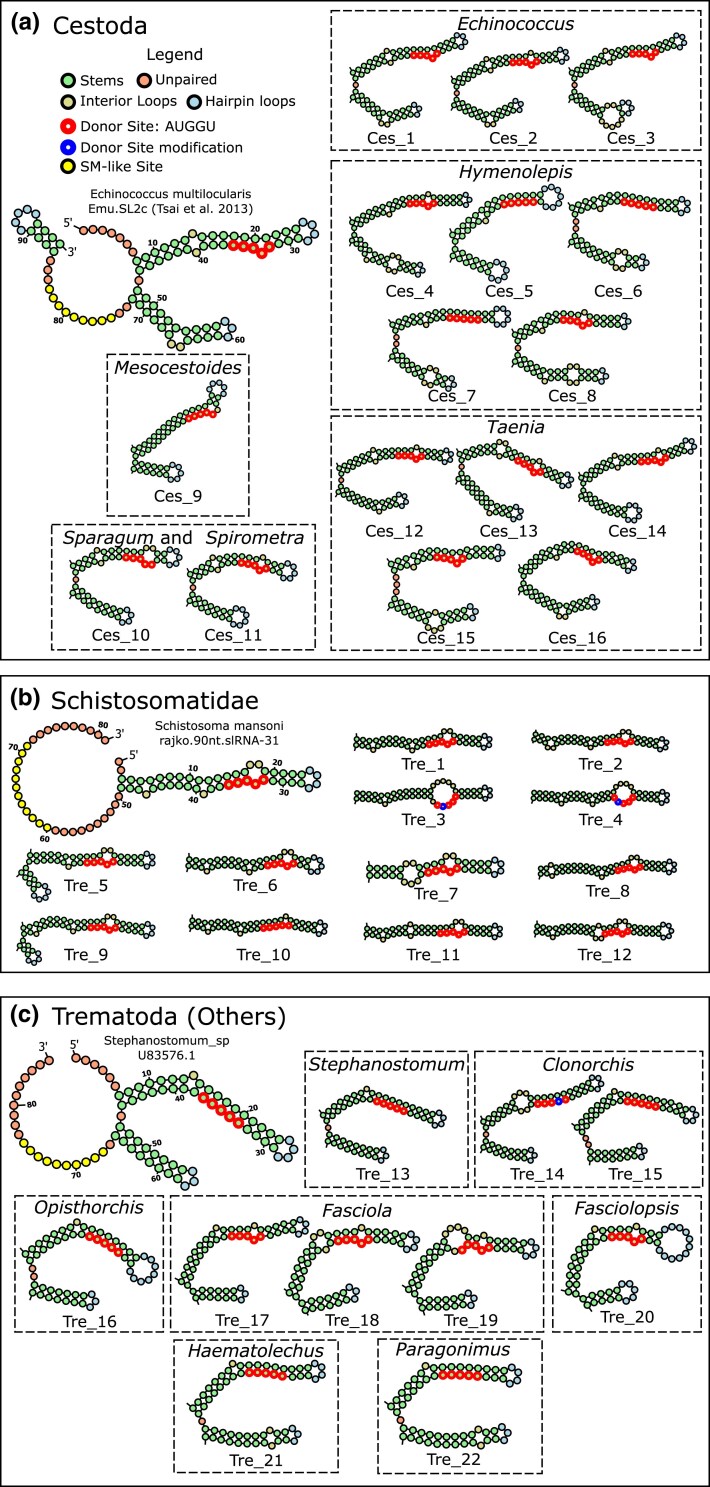
Secondary structural elements in the SL-RNAs of parasitic flatworms. a) SL-RNAs from cestodes, b) SL-RNAs from trematodes of the family Schistosomatidae, and c) SL-RNAs from other trematodes.

Two conserved regions are present among these sequences, separated by a highly variable region. The first region comprises the characteristic “AUG” motif found at the 3′ end of the leader sequences, followed by a canonical 5′ splice donor site, “GU” (Fig. 2). This motif was widely conserved, with a few exceptions: in two cases, Unique_SL-1 (*Clonorchis sinensis*) and Unique_SL-48 (*Schistosoma bovis*), the AUG motif is not conserved, and in three cases, Unique_SL-18 (*S. bovis*), Unique_SL-20 (*Schistosoma haematobium*), and Unique_SL-37 (*Fasciola hepatica*), the splice donor site is not conserved. These latter examples are unlikely to constitute functional SL-RNAs given that the transcriptomic evidence of trans-splicing was negligible (see below); therefore, we interpret these loci as possible SL-RNA pseudogenes. The second conserved region corresponds to the Sm-like motif (Fig. 2). An exact match to the canonical Sm-motif (RAU(n)GR) has never been described in parasitic flatworms, but instead a modified motif with an interrupted uracil stretch (AGUUUUCUUUGG) was originally described in *S. mansoni* (Rajkovic et al 1990). The most conserved part of this motif is the 3′ portion, a UVUUUGG 3′ sequence, while the 5′ end is considerably more variable, as we previously described in *Hymenolepis microstoma* (Calvelo et al. 2023).

The predicted secondary structure of SL-RNAs from cestodes and trematodes can be distinguished into two large groups. One group has a predominant arrangement with two predicted hairpins upstream the Sm-like site, which was previously reported in *Echinococcus multilocularis* (Brehm et al. 2000), *F. hepatica* (Davis et al. 1994), and *Opisthorchis felineus* (Ershov et al. 2019). The second group has a single large predicted hairpin (Fig. 2), initially reported in *S. mansoni* (Rajkovic et al. 1990). The latter was only predicted in schistosomatid species and is considerably more unstable, with an average Maximum expected accuracy (MEA) of −15.54 kcal/mol vs. −30.67 kcal/mol (Table S3). Full structure predictions are provided in File S2. Regardless, both types of structures share two conserved features: First, the splice donor site is located at a comparable position on the first predicted hairpin, typically associated with an internal loop or mismatch, and second, several SL-RNAs displayed another predicted hairpin immediately downstream the Sm-like motif. These results are consistent with previous reports on several flatworms (Rajkovic et al. 1990; Davis et al. 1994; Brehm et al. 2000; Ershov et al. 2019). The downstream hairpin was not recovered consistently in our analysis, but this is likely due to limitations in the definition of the 3′ end of the SL-RNA transcript.

Twelve SL-RNAs had atypical predicted secondary structures but were included in the final set of curated SL-RNAs because they were highly similar to known SL-RNAs when both sequences were trimmed at the same positions (referred to as “outliers” in File S3; three of these correspond to SL-RNAs that were recovered in the secondary blast searches for stragglers described in Fig. 1e). The most common elements in these atypical structures include self-annealing within the leader region forming an extra hairpin, or closing the structure by the annealing of the 3′ with the Leader. Our results showed that these atypical structures are very sensitive to the predictions of the 5′ and 3′ ends of the SL-RNA boundaries, down to the inclusion or removal of a single base (see selected examples in File S4), suggesting that the typical structure found in other SL-RNAs may also be present in these SL-RNAs.

**Fig. 3.**
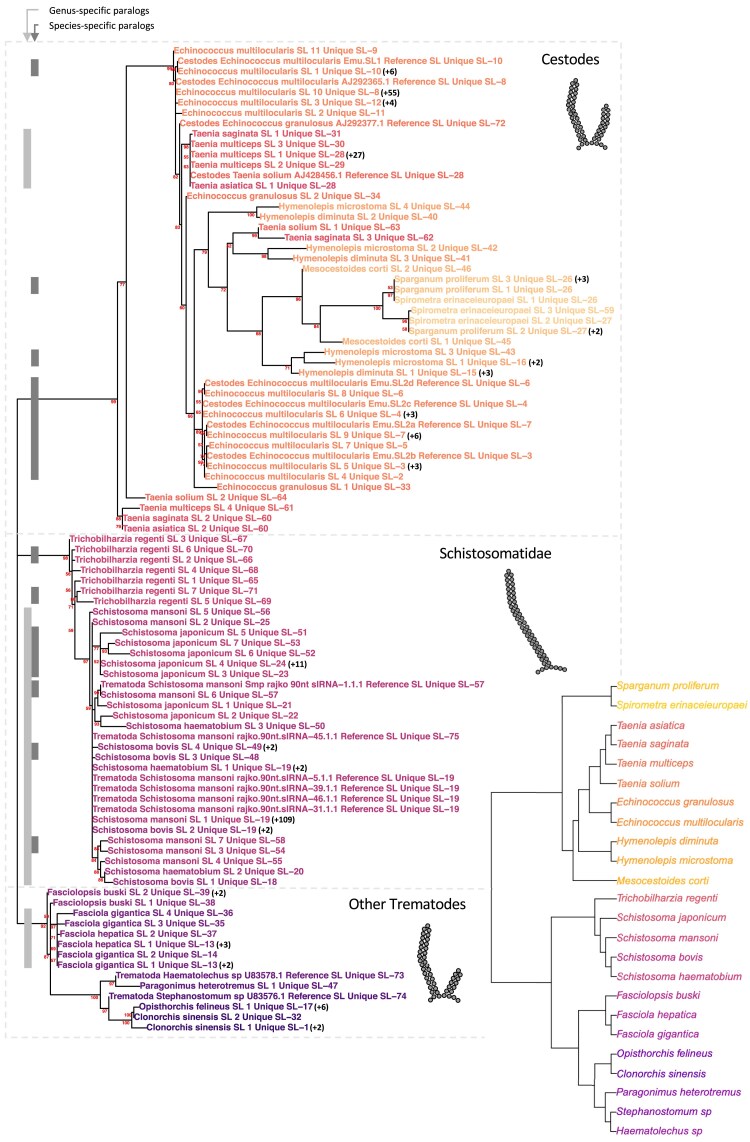
ML phylogeny of flatworm SL-RNAs, with bootstrap support indicated next to the nodes. Values below 50% are omitted. Previously described sequences are indicated as “Reference SL.” Three groups are highlighted with a schematic representation of their structure: cestode SL-RNAs, Schistosomatidae SL-RNAs, and SL-RNAs from other trematodes. Tips marked with a “+” identify the number of identical copies of SL-RNAs that have been collapsed to reduce computational cost. The tree reconciliation algorithm was used to compare the SL tree with the species tree and identify duplication events. Species- and genus-specific clades are indicated by light and dark gray bars, respectively. The species cladogram was constructed based on previously published studies (see Materials and Methods section). A schematic drawing of the generalized predicted structures of SL-RNAs in each major clade is included.

To gain insight into the evolution of SL genes, we constructed a maximum likelihood (ML) phylogenetic tree using IQ-TREE. The ML phylogeny of the SL-RNAs mirrors the split in SL-RNA structure within trematode species, with a clear division between Schistosomatidae and the other trematodes (Fig. 3). Using a tree reconciliation algorithm to compare the SL tree and the species tree (File S5), we identified numerous species- and genus-specific duplications across the phylogeny. We observed that paralogous sequences from each species and genus are typically more closely related to each other than to other SL-RNA sequences. In particular, there are several groups of identical SL genes within individual genomes (marked with a “+” symbol in Fig. 3), which have been collapsed for computational reasons. The observed branching pattern of paralog sequences suggests that SL-RNA loci undergo a continuous process of expansion and replacement or that sequence similarity between different paralogs within each species is maintained through concerted evolution. The latter process is more likely to occur as a result of unequal crossing over or gene conversion in tandem copies of genes (Liao 1999), an arrangement found for SL-RNA genes in many eukaryotes, including in different flatworms (Rajkovic et al. 1990; Davis et al. 1994; Brehm et al. 2000).

### SL-RNA Tandem Repeats

**Fig. 4.**
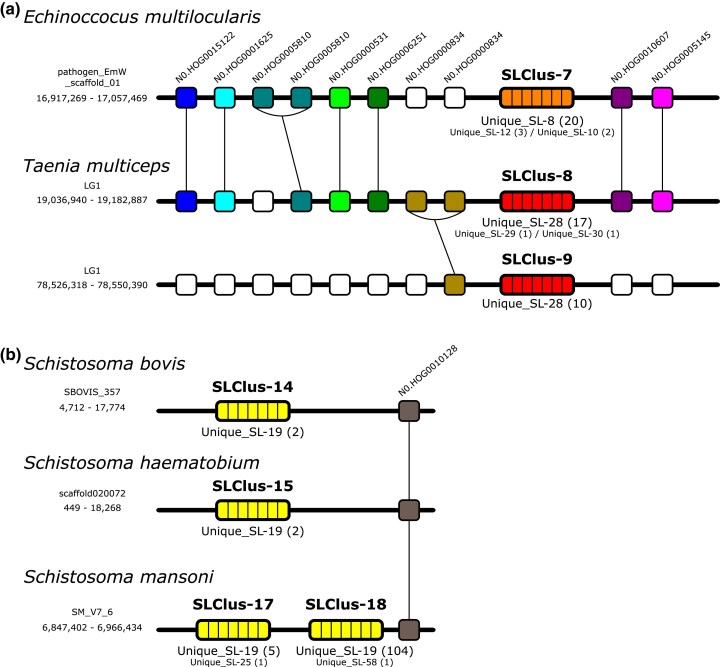
Shared HOG IDs found in a 100 kb window around syntenic SL-RNA tandem repeats. The given coordinates reflect the location of the first and last colored element. The “SLClus” numbers indicate different SL-RNA clusters from different species, as described in Table S5. The “Unique_SL” numbers correspond to unique SL-RNA sequences described in Table S3, and the numbers in parenthesis indicate the number of copies of each Unique_SL sequence in each cluster. The most common Unique_SL sequence in each cluster is indicated in larger fonts, and additional Unique_SL sequences found in each cluster are indicated in smaller fonts.

We searched for SL-RNA tandem repeats in the genomes of parasitic flatworms, as this genomic organization facilitates concerted evolution in gene families through unequal crossing over or gene conversion mechanisms. Here, SL-RNA tandem repeats, defined as SL-RNA loci encoded on the same strand and separated by fewer than 5,000 bases, were identified in several cestode and trematode species (*E. multilocularis*, *Fasciola gigantica*, *F. hepatica*, *O. felineus*, *S. bovis*, *S. haematobium*, *Schistosoma japonicum*, *S. mansoni*, and *Taenia multiceps*) (Table S5). By searching for shared homologous genes within a 100 kb region, it was possible to identify synteny for tandem repeats between *E. multilocularis* and *T. multiceps* (SLClus-7 with SLClus-8), tentatively between two clusters within *T. multiceps* (SLClus-8 with SLClus-9), and between the schistosomatid species *S. bovis* (SLClus-14), *S. haematobium* (SLClus-15) and *S. mansoni* (SLClus-17 and SLClus-18) (Fig. 4). The ability to identify these types of clusters is significantly impacted by the quality of the genome assembly, as demonstrated by *S. bovis* and *S. haematobium*, which have two SL-RNAs each and genomic assemblies with N50 values of 203 and 4,780 kb, compared to the 111 SL-RNAs identified in *S. mansoni*, with a genomic assembly with an N50 value of 50,458 kb (Table S2).

The shared clusters identified on *E. multilocularis* (SLClus-7) and *T. multiceps* (SLClus-8) are noteworthy. Their surrounding orthologous genes show a conserved synteny, but the SL-RNA genes within each species are more similar to each other than to those of the other species (sequences Unique_SL-8, 10, and 12 from *E. multilocularis* and Unique_SL-28, 29, and 30 from *T. multiceps*; see Fig. 2). Because of the importance of high-quality genome assemblies for the identification of SL-RNA gene clusters, we searched the location of the SL-RNAs described in this work on a new *E. granulosus* genome assembly (ID: ASM2155672v1) generated with long read data by Korhonen et al. (2022). In contrast with assembly ASM52419v1 generated by Tsai et al. (2013), 93 SL-RNA loci were identified, concentrated in two clusters: one on chromosome 1 and one in contig8_egr, with an additional five SL-RNA loci organized in a looser arrangement on chromosome 5. These clusters comprised mostly sequences similar but not identical to Unique_SL-8 from *E. multilocularis* (Table S6). The surrounding gene synteny confirms that the SL cluster on chromosome 1 is orthologous to the SL-RNA gene clusters found in *E. multilocularis* and *T. multiceps* (Table S7). Altogether, these results support the existence of concerted evolution of tandem SL-RNA loci in these species. However, species such as *H. microstoma*, which has a high-quality assembled genome encoding only five nonclustered SL-RNAs, suggest that the presence of these clusters is not universal in Platyhelminthes.

### SL-ACEs and Target Genes

**Fig. 5.**
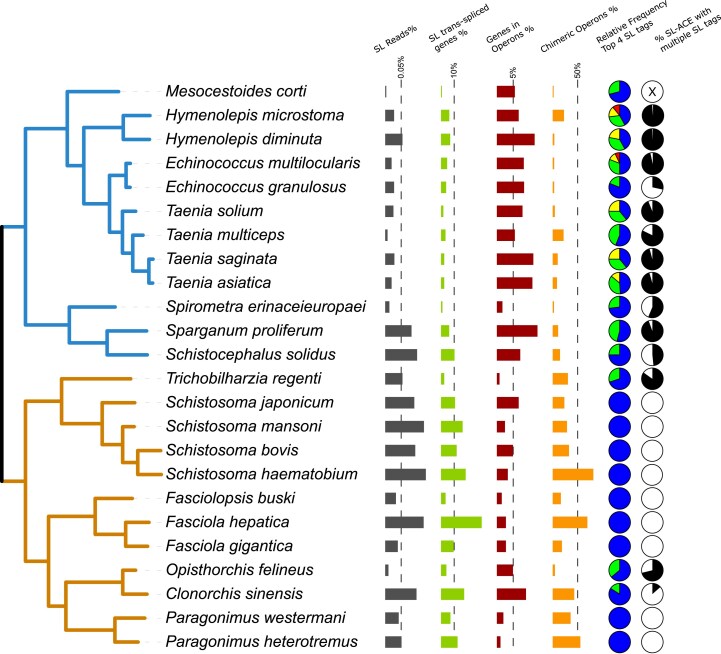
Extent of SL trans-splicing across the phylogeny of parasitic platyhelminthes (Neodermata). The species tree was estimated by OrthoFinder using the STAG method (trematode lineages are shown in orange, and cestode lineages are shown in blue). For each species, the graphs on the right show the percentage of SL-bearing reads relative to the full RNA-seq dataset for the species (“SL Reads %,” max ∼0.12%), the observed prevalence of SL trans-spliced genes in their genome (“SL trans-spliced genes %,” max ∼30%), the relative frequency of genes encoded in potential operons estimated in this study (“Genes in Operons,” max ∼13%), the proportion of operon candidates obtained from chimeric gene models (“Chimeric Operons,” max ∼80%), the relative abundance of the top four SL tags for each species (“Relative Frequency Top 4 SL Tags”; the SL tags with fewer than 5% SL-bearing reads were excluded from the percentage estimation), and the proportion of SL-ACEs supported by more than ten reads that received more than one SL tag (“%SL-ACE with multiple SL tags,” the proportion of SL-ACEs with at least 25% of the supporting reads belonging to a minor SL TAG are shown in black).

To identify and analyze individual SL-ACEs, we searched for reads containing SL sequences. To this end, we defined SL tags as the last 15 bases of the SL portion (upstream the donor site) and searched for reads supporting trans-splicing in RNA-seq data. In total, 30 SL tags were identified from the pool of SL-RNA loci: 19 for Cestoda and 11 for Trematoda (Tables S8 and S9). These SL tags were used to identify SL-ACEs using the SLFinder-Genes pipeline (Calvelo et al. 2023), in which reads containing SL tags were identified, trimmed, and mapped to known genes in each genome, confirming the presence of an adjacent canonical splice acceptor site (ie an “AG” motif). SL-ACEs with a minimum support of four reads were used for further analysis. As a result, 29,118 SL-ACEs were identified in all genomes analyzed, 63% of which are supported by at least 10 reads, with a median read support of 15 and an average of 90. In all cases, SL-bearing reads represented ∼0.001% to 0.1% of the total RNA-seq data across different species and datasets (Fig. 5). The percentage of SL-bearing reads were similar to those previously found in *S. mansoni* and *H. microstoma* (Boroni et al. 2018; Calvelo et al. 2023) and, although relatively low, still resulted in thousands of reads for most species. The reason behind the low proportion of SL-bearing reads is likely methodological, as RNA-seq samples processed with a poly-A capture protocol tend to have a lower read coverage toward the 5′ end due to degradation (Conesa et al. 2016), and conventional cDNA synthesis methods can frequently result in the loss of the 5′ end sequences (Schaefer 1995). Even in high-quality samples, some level of degradation is to be expected, and the location of the SL sequence in the transcript makes it particularly vulnerable to this bias.

Canonical splice acceptor motifs were identified adjacent to SL-ACEs for all species except *Mesocestoides corti* (due to the limited data in this species) (Table S10 and Fig. S1). All SL-ACE splice acceptor motifs identified match the motif “UUNYAG,” with nonschistosomatid trematodes favoring “UUNCAG” over “UUNUAG.” No other enriched motifs were detected within 50 bases of the SL-ACEs.

The total SL-ACE counts for each species were correlated with the total number of observed trans-spliced reads (Pearson, *r* = 0.79; *P* < 0.01), with cestode species displaying lower overall percentages of SL-bearing read counts than trematodes, especially in the case of cestode order Cyclophyllidea (including *Echinococcus*, *Taenia*, *Hymenolepis*, and *Mesocestoides*) (Fig. 5; Table S8). The percentage of trans-spliced genes detected in each species was always below 31%, and was below 10% for all cyclophyllidean cestodes, including those with high RNA-seq coverage such as *Echinococcus* spp. and *Hymenolepis* spp. We observed significant and unexpected differences in the fraction of SL trans-spliced genes between closely related species. Our results suggest that these differences are largely artificial, driven by the total number of RNA-seq reads analyzed. That is, a higher number of RNA-seq reads lead to the identification of more SL-spliced genes, making read depth a main factor explaining the observed variation. This effect is even more pronounced in species with lower-quality genome assemblies, where fewer SL-spliced genes can be detected (Table S2). Moreover, differences in library construction or the potential effect of SL RNA structure on mRNA 5′ recovery, specific to each species, cannot be completely ruled out as contributing factors to the observed results. To gain insight into these effects, we normalized the data for comparative analysis. Specifically, for each species, 5 million reads were subsampled ten times, and the distribution of the number of genes detected with SL was inferred. Also, we subsampled 1,500 reads from the pool of SL-bearing reads in each species and repeated the analysis. In these analyses, we observed in most cases only marginal differences between closely related species, indicating that sequence coverage was a major factor behind their apparent differences, and it is apparent that the available data do not reach saturation for the detection of trans-spliced genes (File S6). Determining the full extent of trans-splicing in these species will require specialized approaches such as SL trapping (Nilsson et al 2010; Boroni et al 2018).

To determine the importance of trans-splicing in the expression of highly conserved genes (presumably involved in essential processes), we verified how many Benchmarking Universal Single-Copy Orthologs (BUSCO) marker genes contained SL-ACEs in each species. All species had BUSCO marker genes with SL-ACEs, and on average 23% (SD ± 19%) of all BUSCO genes had evidence of trans-splicing (the values were similar in cestodes and trematodes; Table S11). Furthermore, we found 35 genes with trans-splicing in *S. mansoni* (with at least one SL-ACE supported by more than three reads) among the list of 195 genes that produced fully penetrant detachment RNAi phenotypes in the screening performed by Wang et al. (2020). These results indicate that affecting trans-splicing in parasitic flatworms could interfere with numerous essential processes.

### Improvement of the Annotation of Operonic Genes Based on SL-ACE Locations

**Fig. 6.**
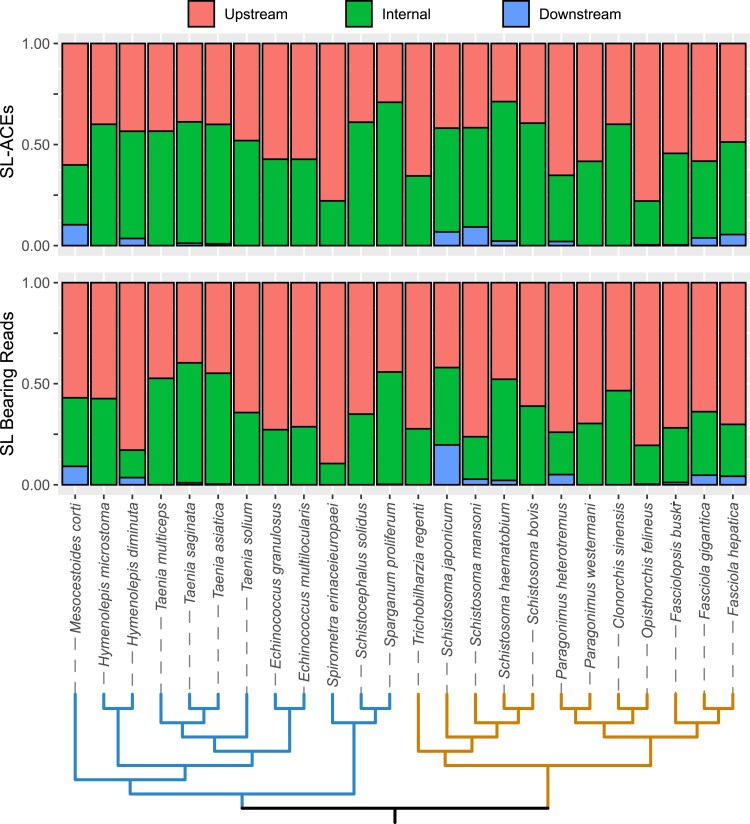
Distribution of the number of SL-ACEs and read counts assigned to Upstream, Internal, and Downstream categories (defined according to the location of the acceptor site relative to the CDS of their assigned gene model). SL-ACEs assigned to more than one gene model, or in the opposite strand, were excluded. A cladogram of the species tree is shown at the bottom.

SL-ACEs were initially classified according to their positions relative to the annotated gene models with which they were associated. Most of them were found upstream of their assigned gene model coding sequence (CDS) (generally supported by a higher number of reads) or as internal sites (generally with lower read support) (Fig. 6). In addition, a small proportion of SL-ACES were located downstream of the gene model to which they were assigned and/or on the opposite strand, especially in *S. mansoni* (9% of all acceptor sites). It is likely that these result from missing and/or overlapping genes in the genome annotation.

The high number of internal SL-ACEs that we detected was unexpected. However, it is important to emphasize that the number of SL-bearing reads and SL-ACEs in internal positions is relatively low compared to upstream positions. This is particularly notable given that internal regions have a greater sequence length and, consequently, a higher potential number of trans-splicing sites. The lower abundance of SL-bearing reads in internal regions, despite their larger size, suggests that SL trans-splicing occurs predominantly at upstream positions. On the other hand, as we previously observed, genes within operons can be mis-annotated and fused into single gene models (chimeras) in the currently available genome annotations (Calvelo et al. 2023). In order to identify chimeric gene models from other types of internal sites, and accurately discriminate between different genes, we selected four species from each group as references, selecting the best assembly quality based on N50 values and picking up to one per genus. *E. multilocularis*, *H. microstoma*, *Sparganum proliferum*, and *T. multiceps* were selected for Cestoda and *C. sinensis*, *F. hepatica*, *O. felineus*, and *S. mansoni* for Trematoda. To identify potential chimeras, genes were divided into two parts by each internal SL-ACE, and similarity searches against proteins of the reference species were done by BLASTx. The blast search was repeated with the full transcript, and the SL-ACE position was assessed relative to the reported hits. A gene model was only considered chimeric if the SL-ACE separated sequences that are most similar to different annotated genes in any reference species, with a maximum overlap of 30 bases between the different blast hits obtained with the full transcript.

The initial assessment showed that many internal SL-ACEs corresponded to trans-splicing in the 5′ region of genes in operons that have been mis-annotated and fused into chimeric gene models. However, there is significant variation in the percentage of possible chimeras depending on the species being compared, from as low as 4% when comparing *E. granulosus* with the reference annotation of *E. multilocularis*, to over 71% when comparing *Taenia saginata* with *E. multilocularis* (Tables S12 and S13). These differences likely result from methodological decisions made during the annotation process, particularly the transfer of annotation between genomes, as seen in the case of *E. granulosus* and *E. multilocularis* (Tsai et al. 2013). When all comparisons are taken into account, the results indicate that a significant proportion of the internal SL-ACEs corresponded to chimeric gene models. However, true internal SL-ACEs also exist, that is, SL-ACEs that truly divide a CDS in two, as described by Boroni et al (2018) for the trematode *S. mansoni*. Similarly, we confirmed three internal SL-ACEs by RT-PCR in the cestode *H. microstoma* (Fig. S2). Therefore, the identification of internal SL-ACEs can be an important tool for the correction of gene models in flatworms, as has been proposed for nematodes (Wenzel et al. 2020), but it needs to be used carefully as true internal trans-splicing sites are also present.

### Extensive Sharing of Acceptor Sites Between Cis- and Trans-Splicing

In other eukaryotes, the SL trans-splicing process is known to utilize much of the same machinery as cis-splicing (Hastings 2005; Lasda and Blumenthal 2011). Some competition between the two processes has been described for splicing acceptor sites (Allen et al. 2011), leading to low levels of trans-splicing at a minority of internal cis-splicing sites. Also, we previously observed high levels of cis-splicing in SL-ACEs in the tapeworm *H. microstoma*, a phenomenon that has not been described in nematode species.

**Fig. 7.**
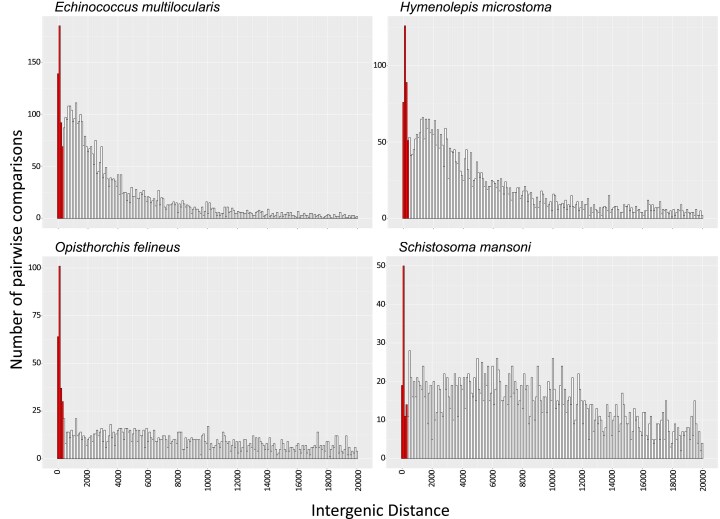
Selected examples for the distribution of intergenic distances between contiguous gene models encoded in the same strand. Bins considered to be operon candidates are highlighted. Histograms for the 24 species analyzed are provided in File S7.

To explore how widespread this phenomenon is across parasitic flatworms, we measured and compared the relative frequency of trans*-* and cis-splicing reads over three categories of SL-ACEs according to their location: (i) upstream the coding region of a monocistronic gene or of the first gene of an operon (named “Upstream Mono”); (ii) upstream of the coding region of a gene in an operon (not including the first gene), and thus related to polycistron resolution (named “Upstream Poly”); and (iii) within the coding region of a gene (named “Internal”; these sites excluded the chimeric gene models identified in the previous section). For this classification, candidate operons were defined as clusters of genes encoded in the same strand and separated by intergenic regions equal or shorter than 300 bp. We set this threshold by analyzing the distribution of intergenic distances between contiguous gene models located on the same strand. We observed a high abundance of genes separated by 300 bases or fewer in all species, including a sharp peak for several species (Fig. 7 and File S7). This distance is also similar to that selected in previous studies of candidate polycistronic loci in flatworm species (200 to 1,000 bp; Protasio et al 2012; Tsai et al 2013; Ustyantsev and Berezikov 2021).

**Fig. 8.**
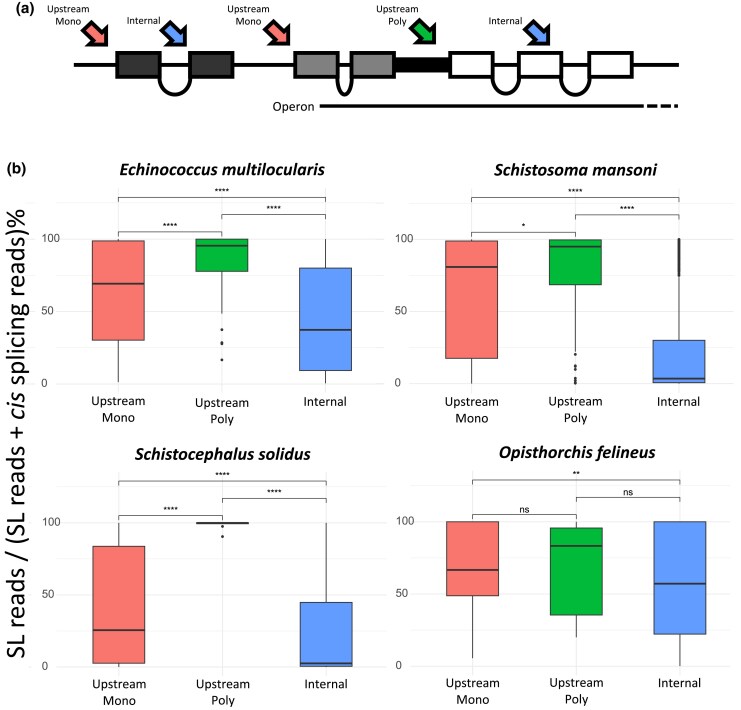
Proportion of SL-bearing reads relative to all splicing reads mapped to different sets of SL-ACEs. a) Schematic representation of each SL-ACE group: (i) Upstream Mono, reads mapped upstream the coding region of a monocistronic gene or upstream the first gene of an operon; (ii) Upstream Poly, reads mapped upstream the coding region of a gene, related to polycistronic gene resolution; and (iii) Internal, reads mapped to SL-ACEs located within the coding sequence. Only exons that are part of the coding sequence are shown. b) Percentage of SL trans-splicing relative to cis-splicing found at each type of SL-ACE in four selected species. Pairwise comparisons were carried out with the Wilcoxon rank sum and signed rank tests; significant levels of the pairwise comparisons are displayed with the following codes: ns, *P* > 0.05; *P ≤ 0.05; **P ≤ 0.01; ***P ≤ 0.001; and ****P ≤ 0.0001. Results of all species, except *M. corti*, are included in Fig. S3. *M. corti* was discarded due to limited data. SL-ACEs assigned to chimeric gene models were not considered.

The results showed that most species exhibit a high degree of coexistence of SL trans-splicing and cis-splicing at the same acceptor sites. The proportion of SL-ACEs showing any evidence of cis-splicing ranged from 20% in *Spirometra erinaceieuropaei* to 95% in *S. mansoni*, with an average of 66% (Table S14). This proportion decreased moderately when the threshold for inclusion was set to three reads supporting cis-splicing (9.5% to 91%, with an average of 52%). This important variation across species likely corresponds to a combination of varying data quality and different annotation biases of each genome. In almost all analyzed species, SL-ACEs classified as Upstream Poly had the lowest levels of cis-splicing, followed by Upstream Mono SL-ACEs with intermediate levels of cis-splicing (and with a high dispersion), whereas Internal SL-ACEs had the highest levels of cis-splicing (Fig. 8 and Fig. S3 and Table S15). Complex patterns of splicing were observed in the 5′ UTR region of many genes containing Upstream Mono SL-ACEs, including alternative donor and acceptor cis-splicing sites, as well as multiple SL-ACEs, as we previously described in the cestode *H. microstoma* (Calvelo et al. 2023). Overall, the results indicate that although cis-splicing is strongly prevented in SL-ACEs related to operon resolution, extensive coexistence of SL trans-splicing and cis-spicing occurs at other sites.

### Lack of Target Specialization of SL-RNAs

For species with two or more SL tags, we searched for evidence of SL-RNA specialization at particular SL-ACEs relative to the overall SL usage across all samples. Most SL-ACEs from these species had reads with different SL tags (Fig. 5), suggesting a lack of specialization. In order to detect any quantitative usage bias among SL-RNA paralogs, chi-square tests were conducted on highly supported SL-ACEs (with at least five observed and expected read counts for each SL tag; the median number of total reads supporting each analyzed site was between 25 and 135 for each species). Consistent with our previous analysis in *H. microstoma* (Calvelo et al. 2023), very few sites exhibited statistically significant biases in their use of different SL tags relative to the overall average, and even in these cases, the biases were marginal (summary in Table S16; full details in Table S17). Furthermore, we explored whether any SL-RNAs may be specialized for operon resolution, as shown for SL2 in nematodes. When comparing all Upstream Mono SL-ACEs with Upstream Poly SL-ACEs, only a few species showed small biases in their SL tags, with significant results under a Wilcoxon rank-sum test. However, their biological relevance is questionable given the slight differences in their median proportions (Table S18). Tags usage bias to Upstream Poly SL-ACEs goes from 0.2% to 21.1%, with an average of 5.4% (SD ± 5.3%). Note that those cases of relatively higher bias are associated with lower number of analyzed SL-ACEs. In summary, we did not find any clear evidence of SL-RNA specialization among parasitic flatworms, as different paralogs appear to be used indiscriminately.

### Conservation of SL Trans-Splicing Acceptor Genes

Our analysis includes diverse and distant parasitic flatworm species, allowing us to study the conservation of genes targeted by SL trans-splicing, which was determined based on orthology inference analysis. For this purpose, the longest isoform reported, after resolving chimeric genes, was identified and processed using OrthoFinder (Emms and Kelly 2019). In total, 338,481 sequences were sorted into 28,636 phylogenetic hierarchical orthogroups (HOGs) (Table S19). The species tree estimated using the STAG method (Emms and Kelly 2018) aligns with other phylogenetic studies published for the group (Coghlan et al. 2019).

**Fig. 9.**
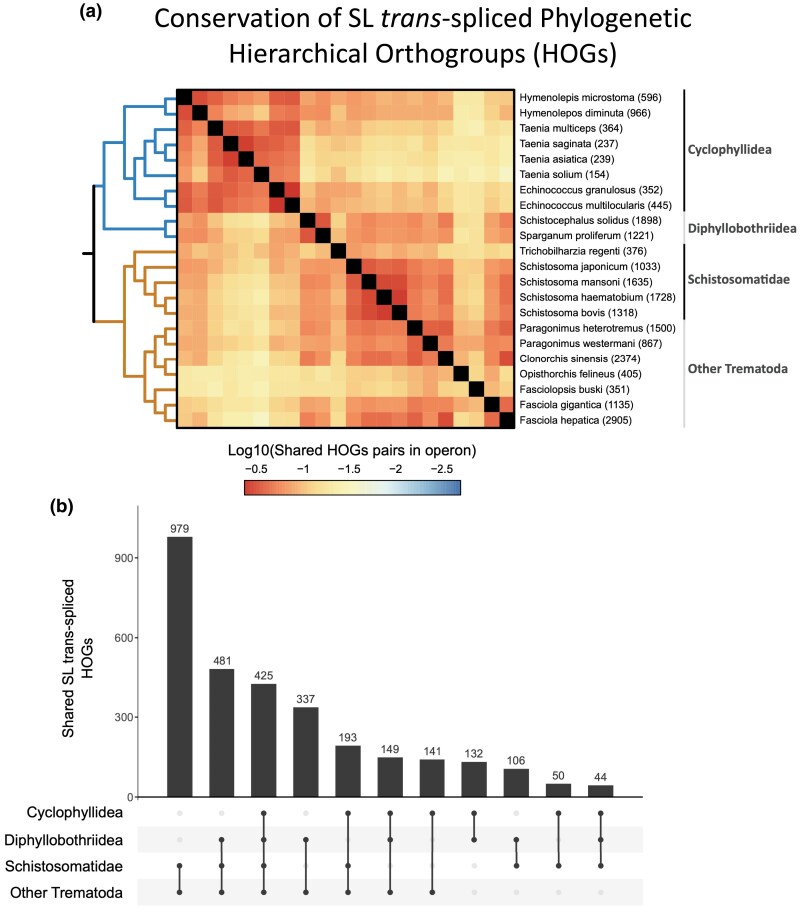
Conservation of SL trans-splicing acceptor genes. a) Heatmap showing the pairwise proportion of shared HOGs subjected to SL trans-splicing in logarithmic scale. The scale goes from −2.7 (∼0.002%) to −0.37 (∼42.658%). The number of HOGs considered per species is displayed on the row's IDs. b) Upset plot of trans-spliced HOGs shared between different groups, excluding those shared with other groups. Each bar represents the size of a distinct, nonoverlapping set of HOGs that belong only to the sets defining that specific intersection, and not to any other sets (exclusive intersection mode). Below each bar, black dots indicate groups in which the set of HOGs have evidence of trans-splicing in at least one species of each group and gray dot groups in which the set of HOGs do not have evidence of trans-splicing. The species *M. corti* and *S. erinaceieuropaei* were excluded from this analysis due to very low numbers of detected SL-ACEs.

In this analysis, a HOG was considered to have conserved SL trans-splicing between two species if at least two genes in the HOG, one in each species, were identified as having SL-ACEs. Although conservation values were relatively low, with two species sharing, on average, 11% of their HOGs subjected to SL trans-splicing, a clear phylogenetic signal was apparent when comparing, among pairs of species, the proportion of shared trans-spliced HOGs (Fig. 9a). The low global levels of shared trans-spliced HOGs may reflect both true gain and loss of trans-splicing in different lineages, as well as incomplete discovery of trans-splicing acceptor sites in most species, caused by the lack of saturation in the discovery of SL-ACEs due to the low numbers of SL-bearing reads found in conventional RNA-seq datasets. Therefore, definite conclusions regarding gain and loss of individual trans-splicing sites will require the incorporation of specialized sequencing methods such as SL-trapping (Nilsson et al 2010; Boroni et al 2018). However, the results already indicate that many trans-spliced HOGs can probably be traced back to the last common ancestor of the Neodermata (Fig. 9b). For example, there are 425 HOGs with evidence of trans-splicing in at least one cyclophyllidean cestode, one diphyllobothriidean cestode, one schistosomatid trematode, and one nonschistosomatid trematode. Furthermore, there is an even larger number of trans-spliced HOGs shared exclusively among trematodes (979 HOGs with evidence of trans-splicing in at least one schistosomatid and one nonschistosomatid trematode), indicating a large ancestral repertoire of trans-spliced genes in this class. Fewer trans-spliced HOGs were exclusively shared in cestodes (only 132 HOGs were shared between at least one cyclophyllidean cestode and one diphyllobothriidean cestode) (Fig. 9b). The complement of trans-spliced HOGs exclusively shared between diphyllobothriidean cestodes and trematodes was surprisingly higher (481 HOGs shared between at least one diphyllobothriidean cestode, one schistosomatid trematode, and one nonschistosomatid trematode), which could also correspond to ancestral neodermatan trans-spliced genes for which trans-splicing was either evolutionarily lost or could not be recovered in our cyclophyllidean datasets.

### Conservation of Operons

Two genes were defined as part of a candidate operon if they were encoded on the same strand and separated by no more than 300 bases. Also, all gene models identified as chimeric (see above) were considered as candidate operons. Cestode genomes exhibited more than double the number of operon candidates compared to trematodes, with a median of 448.5 versus 182, respectively (Tables S20 and S21). This trend is more pronounced in cyclophyllidean species, which may reflect both assembly quality and their smaller genomes. Notably, the number of operon candidates that originated from chimeric gene models varied greatly between different species, from 1% to 21% in cestodes and from 3% to 81% in trematodes (Fig. 5). The vast majority of operons comprised only two genes in the different analyzed species; the maximum number of genes per operon candidate was nine (candidate Operon-45 in *S. japonicum*). This is in line with previously described for operons in the trematode *S. mansoni* and in cyclophyllidean cestodes (Tsai et al 2013; Boroni et al 2018).

In other groups of organisms with SL trans-splicing, there are two different types of operons that have been described regarding the distance between cistrons. In most known cases, such as in the majority of operons in *C. elegans* (which are known as SL2-type operons, in which the downstream gene receives a SL2 SL), there is an intervening sequence between the site of 3′ polyadenylation of the first gene and the SL acceptor site of the second gene (Blumenthal 2018). In other cases, such as in a minority of operons of *C. elegans* (known as SL1-type operons, in which the downstream gene receives a SL1 SL; Blumenthal 2018), and in the operons of the urochordate *Ciona intestinalis* (Satou et al 2006), there are no intercistronic sequences, and the polyadenylation site of the first gene directly abuts the SL acceptor site of the second gene. Unfortunately, most flatworm species lack high-quality predictions for their 3′ UTRs, which prevented us from determining which type of operons is present. However, for *S. mansoni*, there are predictions available for the UTR regions of many genes, and for this species, all analyzed pairs of genes in candidate operons with UTR predictions and SL-ACEs (45 pairs) had an intercistronic distance of at least five base pairs (average 233 bp, median 99 bp; Table S22). Therefore, most operons in this species appear to be of the first type.

**Fig. 10.**
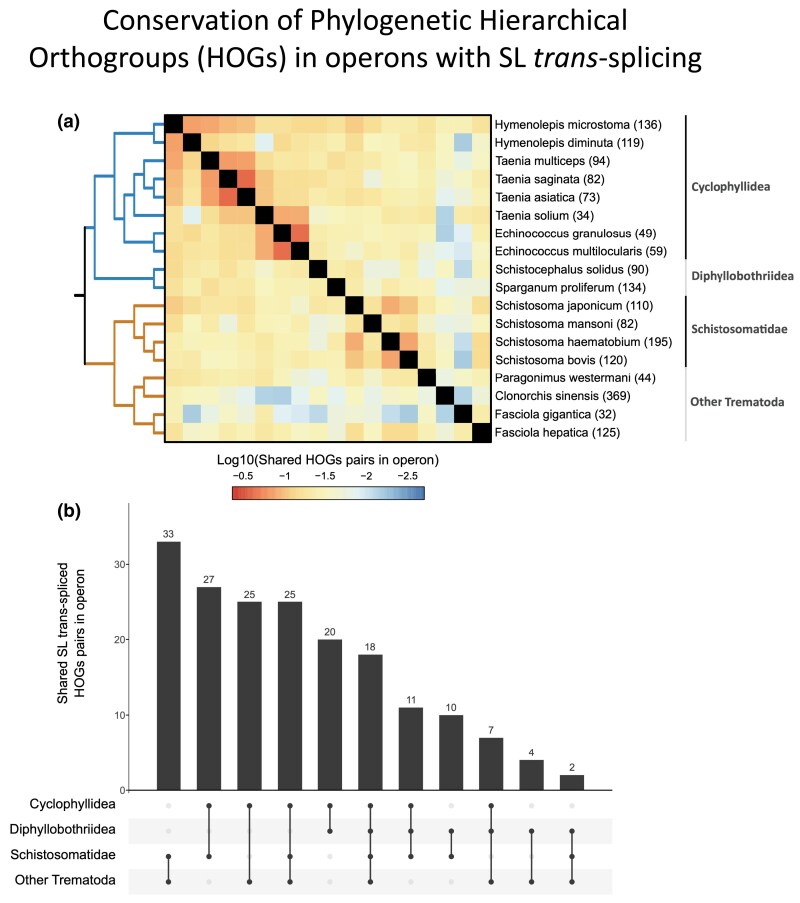
Conservation of operons. a) Heatmap showing the pairwise proportion (in logarithmic scale) of HOG pairs in operon candidates with evidence of SL trans-splicing. The scale goes from −2.7 (∼0.002%) to −0.37 (∼42.658%). The number of HOG pairs considered per species is displayed between brackets next to the species IDs. b) Upset plot of the shared HOG pairs in operons between different groups, excluding those shared with other groups. Each bar represents the size of a distinct, nonoverlapping set of HOGs that belong only to the sets defining that specific intersection, and not to any other sets (exclusive intersection mode). HOG pairs were included if they were identified as trans-spliced in at least one species of two or more groups.

Conserved operons between two species were defined based on the homology of their members and evidence of SL trans-splicing of the second gene in both species. Very few HOG pairs were conserved in more than half of the species, likely due to the low recovery of SL-ACEs by the implemented methods. Nevertheless, we found similarity of the operon complement among cyclophyllidean species, while similarity seems to be limited to *Schistosoma* within Trematoda (Fig. 10). This suggests that while there is a set of HOGs whose SL trans-splicing is conserved across parasitic flatworms, their organization into specific operons is not widely conserved.

### Phylogenetic Patterns of Trans-Splicing and Operons

In order to analyze the phylogenetic patterns of trans-splicing for individual genes in parasitic flatworms, we first focused on HOGs which (i) showed evidence of SL trans-splicing in at least five species, (ii) included representatives of at least two of the reference species, and (iii) either had evidence of SL trans-splicing in each represented species or had relatively high expression levels (equal to or higher than the median Transcripts Per Million (TPM) values of genes with confirmed SL trans-splicing in that species). For these highly expressed genes, the lack of detection of trans-splicing is less likely to be a false negative. In total, 41 HOGs were selected for manual inspection (Table S23 and File S8). Most of the analyzed HOGs displayed a conservation pattern consistent with SL trans-splicing as the ancestral condition in the last common ancestor of cestodes and trematodes, which may correspond to the last common ancestor of Neodermata (Brabec et al. 2023), followed by cases of possible gain within trematodes. These patterns indicate that the SL trans-splicing mechanism was conserved in several genes throughout the evolution of this lineage. Interestingly, the analyses of HOGs N0.HOG0007613, N0.HOG0009887, N0.HOG0010318, N0.HOG0010643, and N0.HOG0010915 suggest the loss of SL trans-splicing in these genes in Cyclophyllidea.

In order to identify evolutionary changes in operon structure, we focused on HOG pairs in operons that were well conserved within cestodes or trematodes (observed in at least four genera of cestodes but not in trematodes or vice versa). We also searched for candidate operons among the HOGs previously selected for the analysis of conservation of trans-splicing (discussed above). In total, 26 putative operons were selected for manual analysis (Table S24 and File S9). Although the phylogenetic patterns found were complex and no operons were conserved in all species, some operons were found in representatives of both cestodes and trematodes and may be ancestral to parasitic flatworms. Those species for which these operons were not conserved include changes in synteny, which clearly demonstrate that the HOGs are not part of an operon (see, eg Op3, Op8, and Op22) as well as cases in which the intergenic distances exceed the 300 bp threshold set in this study (see, eg Op2 and Op5). However, it should be noted that the 300-bases threshold distance is likely very conservative, especially in light of the chimeric gene models found, which included intergenic distances of up to several thousand base pairs.

Lastly, one interesting case is Op8, including the HOG pair N0.HOG0011542__N0.HOG0002707; annotated as “Cytochrome b-c1 complex subunit 7” and “Enolase,” respectively. SL trans-splicing of enolase has been shown in many trematode species, but was shown to be absent in some cestodes (Davis 1997; Davis and Hodgson 1997). Our analysis shows that SL trans-splicing of enolase is rare in cestodes, where it is always encoded in a monocistronic locus, but is pervasive in trematodes, where the enolase-coding gene is the second member of a conserved operon (see File S9). Thus, it appears that either the enolase transcript gained SL trans-splicing processing when it formed an operon in trematodes, or it was lost in some species after becoming monocistronic in cestodes.

## Discussion

### SL-RNA Sequence, Structure, and Lack of Specialization

The observed diversity of SL-RNAs in parasitic flatworms is intriguing. While SL-RNAs are known to be highly variable over large evolutionary distances, they have to interact with the spliceosome to function (Denker et al. 1996, 2002; Blumenthal 2018; Fasimoye et al. 2022). This is a highly conserved macromolecular complex required for cis-splicing, implying selection constraints in SL-RNA sequence and secondary structure. Nevertheless, we observed that SL-RNAs have considerable variability in their primary and secondary structures in the evolution of parasitic flatworms. This includes the fusion of two hairpins in schistosomatids, as well as variations of their Sm-like motif, although the biological implications for SL-RNA function are unknown. Regarding SL-RNAs with atypical predicted secondary structures, although our predictions are in line with previous observations in most cases (Rajkovic et al. 1990; Davis et al. 1994; Brehm et al. 2000; Ershov et al. 2019), it is important to consider that these are highly dependent on sequence delimitation, and additional factors such as the interactions of SL-RNAs with their associated proteins could influence their folding. Thus, it is possible that all SL-RNAs in parasitic flatworms, including those identified in schistosomatids, may fold similarly in vivo.

The analysis on SL-RNA usage across SL-ACEs expands our previous observations in *H. microstoma* (Calvelo et al. 2023) and shows that SL-RNAs in parasitic flatworms are used indiscriminately across their transcriptome. The most parsimonious explanation is that the specialization of SL-RNAs is a nematode-specific innovation that never evolved within parasitic flatworms. We hypothesize that concerted evolution of tandem SL-RNA genes represents a mechanism behind the observed patterns as part of a self-reinforcing loop: Concerted evolution would not be prevented by purifying selection because every functional SL-RNA can be used interchangeably, while specialization is hindered because concerted evolution periodically homogenizes the SL-RNA repertoire of the species.

### Concerted Evolution of SL-RNA Genes

The phylogenetic pattern observed in the SL-RNA phylogeny, where the SL-RNAs of the same species tend to form monophyletic groups (Fig. 3), suggests widespread concerted evolution of these genes in flatworms. This phenomenon is particularly evident within cestodes, where orthologous syntenic clusters have distinct SL-RNA sequences in each species (Fig. 4). Clusters of highly similar or identical SL-RNA loci have been described in other phyla (eg the SL1 cluster of *C. elegans*; Krause and Hirsh 1987), but to the best of our knowledge, analyses of the phylogeny of SL-RNA genes and their genomic arrangements have not been analyzed in other groups at large scales. Interestingly, the specialized SL2 loci of *C. elegans* are not associated with the SL1 cluster and show greater divergence, indicating that their nonclustered organization permits their divergent evolution.

Interestingly, *H. microstoma* lacks clusters of SL-RNA genes despite its high-quality assembly, and therefore it is possible that SL-RNAs are less subjected to concerted evolution in this lineage. Consistently, most *H. microstoma* SL-RNA loci appear to have a one-to-one orthology relationship with those of *Hymenolepis diminuta* (Fig. 3).

### Biological Functions of SL Trans-Splicing

Our results indicate that polycistron resolution is one of the main functions of SL trans-splicing in parasitic flatworms. In this case, cis-splicing utilizing the same 3′ splice acceptor site is likely to be deleterious, as it may prevent the expression of downstream coding sequences and potentially fuse them with the upstream sequences. Our data show that this is very rare in all species, indicating that during polycistron resolution, cis-splicing is effectively out-competed by SL trans-splicing.

In addition, SL trans-splicing can also be detected in the 5′ UTR of a relatively low fraction of genes outside operons, with 37% of the genes with robust evidence of SL trans*-*splicing bearing at least one “Upstream Mono” SL-ACE. The use of available conventional RNA-seq data is a limitation of this work, in comparison to the increased coverage of 5′ ends with 5′ Rapid Amplification of cDNA Ends (RACE) methods, and to the high specific enrichment of SL trapping (Nilsson et al 2010). However, although the available RNA-seq datasets do not allow us to reach saturation in the detection of SL-ACEs, the results of Boroni et al. (2018) comparing conventional RNA-seq with the more specific SL trapping method in *S. mansoni* suggest that standard RNA-seq datasets can capture the majority of frequent SL trans-splicing events. Our results indicate that only a fraction of all mRNAs undergo SL trans-splicing throughout parasitic flatworms, making it unlikely that this process is generally important for the efficient translation of mRNAs, as has been demonstrated in the nematode *C. elegans* (Yang et al. 2017), which has a much higher prevalence of SL trans-splicing (*ca*. 85%; Bernard et al. 2023). Therefore, it is unclear if there is a general function of SL trans-splicing for monocistronic genes, and it is possible that this process is largely neutral in many cases, as evidenced by the existence of abundant cis-spliced transcripts that use the same 3′ splice acceptor site. The abundance of cis-splicing acting on SL-ACEs is in sharp contrast with results published in *C. elegans*, where the phenomenon has only been observed in a minor fraction of SL-ACEs (Allen et al. 2011), and has been previously described in the trematode *S. mansoni* and in the cestode *H. microstoma* (Boroni et al. 2018; Calvelo et al. 2023). Although this could result from efficient competition between cis-splicing and SL trans-splicing for the same primary transcripts, it is possible that SL trans-splicing occurs only in alternative transcripts that originate from internal promoters within the 5′ UTR. These transcripts would present a 3′ splice acceptor site in the absence of a cognate 5′ splice donor site (ie an outron), making them ideal substrates for trans-splicing. This has been demonstrated for specific genes in cestodes (Brehm et al. 2000; Otero et al. 2010) and is common in the cestode *H. microstoma* (Calvelo et al. 2023). This phenomenon has also been described in urochordates, in which many genes are infrequently trans-spliced, and alternative promoters appear to be involved in the generation of different primary transcripts that undergo either cis- or trans*-*splicing at the same 3′ splice acceptor site (Matsumoto et al. 2010).

We also detected many internal SL-ACEs, which typically have lower coverage and have high levels of cis-splicing. Although some of these may correspond to functionally relevant alternative isoforms, it is likely that most correspond to low levels of errors in splicing, in which SL trans-splicing is incorrectly incorporated. This is akin to the documented noise observed in the cis-splicing process itself (Melamud and Moult 2009; Wan and Larson 2018).

### Use of SL-ACEs for Genome Annotation

The widespread identification of chimeric gene models across multiple flatworm genomes indicates that current annotation pipelines are unable to accurately process this type of loci. The full extent of the issue remains unclear, but it involves 21% of all operons predicted in these species, with extreme cases such as *F. hepatica* (69% of predicted operons, 131 chimeric gene models) and *S. haematobium* (81% of predicted operons, 204 chimeric gene models).

Our results show that identification of SL-ACEs can be used to diagnose the chimeric loci and correct this issue. This has been previously done in nematodes (Allen et al. 2011; Wenzel et al 2020), in which the issue is simplified by the specific use of SL2 for operon resolution, but the lack of SL-RNA specialization in parasitic flatworms requires a more complex approach. A fully automated pipeline will require overcoming several roadblocks. The first is acquiring reliable data on the SL-ACEs. While standard RNA-seq dataset can improve the annotation quality of some loci, approaches such as SL trapping (Nilsson et al. 2010) provide an increased coverage of these specific sites. However, that additional data would exacerbate the next roadblock: discriminating biologically relevant trans-splicing acceptor sites from splicing noise. Setting a stringent threshold on read support could help address this issue. Finally, how can the pipeline accurately differentiate SL-ACEs used for operon resolution from true internal SL-ACEs involved in generating alternative isoforms? One strategy that does not rely on previous annotations could be to exploit the strong depletion of cis-splicing reads on SL-ACEs associated with operon resolution.

### Conservation of SL Trans-Spliced Genes

The broad diversity of parasitic flatworms included in this work permits comparative analyses of the complement of genes that are subject to SL trans-splicing in this group, as well as their arrangement into operons. Although the observed patterns are complex and limited by the inability to achieve saturation with the available RNA-seq data, the detection of conserved SL trans-splicing across cestode and trematode species examined in this work greatly strengthens previous suggestions that this process was already present in the neodermatan ancestor, tracing back to the origins of the group, corresponding to a divergence time of at least 259 million years based on the fossil record (de Baets and Littlewood 2015; Laumer et al. 2015). Although there is a clear phylogenetic signal in the complement of SL trans-spliced genes in parasitic flatworms, there is surprising low conservation of their operons, suggesting that their organization is labile, as has been described for nematodes (Pettitt et al. 2014). Nevertheless, the observed conservation of certain trans-spliced monocistronic genes and operons indicates that some aspects of the molecular process are phylogenetically stable and have been maintained throughout the lineage.

## Materials and Methods

### Data Selection, Quality Assessment, and Transcriptome Assembly

RNA-seq data and reference genomes for 12 cestode and 12 trematode flatworm species were downloaded from NCBI (NCBI Resource Coordinators 2015) and the WormBase ParaSite database version WPS15 (Howe et al. 2017). Data retrieved on January 6, 2021. To minimize biases between samples, only paired-end RNA-seq data generated with Illumina technology were utilized. Read quality was assessed using FastQC (Andrews 2010). Adapter sequences and low-quality bases were removed with Trimmomatic v.0.36 (Bolger et al. 2014) using parameters SLIDINGWINDOW:5:20 and MINLEN:25. Lastly, transcriptomes were assembled de novo using Trinity v2.12.0 (Grabher et al. 2011) with read normalization disabled.

### Identification and Retrieval of SL Sequences

Novel putative SLs (pSLs) were identified using the SLFinder v1.09 pipeline (Calvelo et al. 2020) with the option “-me False” to maximize potential discovery. Filters based on the donor site and proximity to proteins were not applied. Possible artifacts generated during the initial Hook sequences generation were manually identified and addressed following Calvelo et al. (2020) recommendations. Novel predictions were improved through BLASTn searches of known SL sequences against the genomes, using the options -task blastn-short, -perc_identity 95, -ungapped, and -qcov_hsp_perc 75. Known SLs were obtained from previously published studies (Table S1). Lastly, based on the expected total lengths of SL-RNA for parasitic flatworms (Rajkovic et al. 1990; Davis et al. 1994; Brehm et al. 2000), putative SL-RNA sequences were defined as the surrounding region around the initial hits: 20 bases upstream and 100 bases downstream (∼140 bases in total) and were retrieved for further analysis. Potential SL-RNA loci located too close to contig boundaries or containing more than 30 unknown bases were excluded from analysis, Step A in Fig. 1.

### SL-RNA Selection and Delimitation

Potentially coding SL-RNA loci were filtered based on three assumptions: (i) the mature portion of the SL-RNAs is more conserved than the surrounding sequence in the genome, (ii) true SL-RNA loci are not overlapped or in close proximity with mobile elements, and (iii) functional SL-RNAs share enough sequence similarity to be grouped together in a neighbor-joining clustering along with already reported sequences. To achieve this, first, nearly identical putative SL-RNA sequences on each flatworm group were clustered using cd-hit-est v4.8.1 (Fu et al. 2012) with default parameters (>90% identity). Then, all representative sequences of each cluster were aligned with MAFFT v7.475 (Katoh and Standley 2013) along with known full SL-RNA sequences. Next, a basic phylogenetic tree was estimated using the neighbor-joining method as implemented in Ninja v1.2.2 (Wheeler 2009), and groups were clustered with TreeCluster v1.0.3 (Balaban et al. 2019) in “med_clade” mode and -t 0.5. Simultaneously, mobile elements were identified for each genome using RepeatMasker v4.1.1 (Smit et al. 2015) with a custom library generated for each through RepeatModeler v2.0.1 (Smit and Hubley 2015). Candidate SL-RNAs were selected based on (i) their inclusion in a phylogenetic cluster with known SL-RNAs and (ii) no mobile element identified within 500 bases of the candidate SL-RNA, unless the locus is part of a cd-hit-est cluster with at least one valid member, Step B in Fig. 1.

Conserved motifs among the filtered sequences for cestodes and trematodes, in addition to the known full SL-RNA for each group, were identified using MEME v4.11.2 (Bailey and Elkan 1994) and utilized to guide manual trimming around conserved regions. An overview of the process is provided in Fig. S4. SM-like sites were manually identified with the “locate” function from the SeqKit package v0.14.0 (Shen et al. 2016) that matches the motif RAU4-7GR, allowing up to two mismatches, Step C in Fig. 1. Secondary structure predictions were made with RNAfold v2.4.18 (Lorenz et al. 2011) setting the SM-like sites as unpaired and visualized in Forna tool (Kerpedjiev et al. 2015). SL-RNA candidates with structures compatible with known SL sequences, having a similar number and disposition of hairpins, were selected for further analysis, Step D in Fig. 1. Lastly, to rescue more potentially viable SL-RNA sequences, we conducted a new BLAST search with the preselected SL-RNA sequences against the reference genomes (-perc_identity 90, -qcov_hsp_perc 85), disregarding mobile element proximity or phylogenetic affinity, and processed the results as described before, Step E in Fig. 1. A complete registry is provided in Table S4, and the neighbor-joining trees used for selection are provided in File S10.

Selected SL-RNA sequences were clustered by sequence identity (same length and full sequence identity), leaving a unique representative per species (see “+” indicated in Fig. 3) and aligned using MAFFT (Katoh and Standley 2013) with the L-INS-I method (options –localpair and –maxiterate 1000). Then, a ML phylogenetic tree of unique representatives was built as implemented in IQ-TREE v2.2.2.3 (Minh et al. 2020), using the ultrafast bootstrap (UFBoot2) test with 1,000 replicates for branch support (Hoang et al. 2018). K2P + G4 was chosen as the best-fit model by ModelFinder (Kalyaanamoorthy et al. 2017), which was run with default parameters as implemented in IQ-TREE. We have compared the SL-RNA phylogeny to the species tree using the Tree Reconciliation algorithm implemented in the ETE package (Huerta-Cepas et al. 2016).

### SL-RNAs in *E. granulosus* Genome Assembly ID ASM2155672v1

Putative SL-RNAs were determined by BLASTn searches between the unique SL-RNA sequences defined in this study and the genome assembly. Reported coordinates are the summary of all overlapping hits for each loci. Orthology with *E. multilocularis* and *T. multiceps* was estimated using OrthoFinder v2.5.4 (Emms and Kelly 2019) with default settings.

### Identification of SL-ACEs

SL-bearing reads (namely reads with a single SL tag) were identified based on the presence of the last 15 bases preceding the donor site of the Leader region of a selected SL-RNA, using SLFinder-Genes from the SLFinder package (described in Calvelo et al. 2023). SL-bearing reads were identified using SLFinder-Genes from the SLFinder package (described in Calvelo et al. 2023). Identification relied on the presence of the last 15 bases preceding the donor site of the Leader region of a selected SL-RNA located at most 40 bases from the read boundaries (35 assumed known bases plus 5 of tolerance). SL-ACEs associated with a reported gene were preclassified based on their location in the gene model, including genes that only have internal SL acceptor sites, Steps F and G in Fig. 1, that is, “Upstream,” sites located before the first start codon annotated for the gene's isoforms; “Internal,” sites within the CDS of at least one of the isoforms; and “Downstream,” sites located after the stop codon to a single gene. Sites were considered for further analysis if they (i) were supported by a minimum of four SL-bearing reads, (ii) were assigned to a single gene, (iii) the SL strand is the same as their assigned gene, and (iv) were not classified as “Downstream.” SL-ACEs located at least 500 bases apart and assigned to a single gene were selected to identify the acceptor site motif in each species. To this end, 50 bases downstream and 10 bases upstream of each SL-ACE were retrieved using SeqKit and analyzed with MEME using the “One Occurrence Per Sequence (oops)” site distribution mode.

The effect of sample size biases in the identification of trans-spliced genes was evaluated with a subsampling strategy based on (i) the amount of RNA-seq reads analyzed for the species and (ii) only subsampling SL-bearing reads. In the first evaluation, reads representing concrete SL-ACEs and other reads of the transcriptome (defined as any other read found in the original datasets) were weighted based on their read counts. Then 10 replicate samples of 5 million reads with no replacement were taken, and the total number of trans-spliced genes supported by at least 1 SL-bearing read was counted. The second evaluation followed the same logic with a sample size of 1,500 SL-bearing reads. Lastly, rarefaction curves were calculated for each species reported genes with at least a single read of support using the rarefy function from the R package Vegan (Oksanen et al. 2025). *M. corti* was not included in this analysis due to limited data.

### Identification of Chimeric Gene Models

Potential chimeric gene models were identified in a two-stage process, aimed to first identify potential chimeric gene models and then filter out false positives that could arise due to fragmentation in the prediction of gene models in the annotation. First, genes were divided into two by their SL acceptor sites, with each pair considered one at a time and only sequences with a minimum length of 60 bases. Each sequence was compared to four reference species using BLASTx with the option “-qcov_hsp_perc 40.” The reference species were *E. multilocularis*, *H. microstoma*, *S. proliferum*, and *T. multiceps* for cestodes and *C. sinensis*, *F. hepatica*, *O. felineus*, and *S. mansoni* for Trematoda. Annotated genes, whose both sections matched a different set of genes in at least one of the reference species, were preselected as potential chimeric gene models. Second, the BLASTx search was repeated with the whole gene, and the position of the acceptor site relative to the hits was evaluated. A gene model was considered chimeric if the annotated gene is separated at the acceptor site in two sections that hit two genes in one of the reference species, with a maximum overlap tolerated of 30 bases. If two or more acceptor sites were found in a single gene model, this was subsequently subdivided, as long as acceptor sites were identified in the putative chimeric gene. Open reading frames (ORFs) within each valid segment with evidence of chimerism were predicted with getorfs from the Emboss package v6.6.0.0 (Rice et al. 2000). Only the longest ORF was used for further analysis. Schematic representations of different chimera cases are presented in Fig. S5.

### Validation of Internal SL-ACEs in *H. microstoma* by Reverse Transcription PCR

PCR was performed using cDNA obtained from adult worms as previously described (Calvelo et al 2023). For the amplification of trans-spliced isoforms, a forward primer for the *H. microstoma* SL3 SL (CGGATAATCGGTCTTACTGTAC) was combined with reverse primers for each analyzed gene (HmN_000800300 Rev: CTTGCAGAAGAAATACCAGG; HmN_000625700 Rev: TCTTAAGGTCCTTTTGCAGA; HmN_000604600 Rev: GTTCCTTCTCCTCCTCATTT). In parallel, fragments of each cDNA confirming the existence of cis-splicing in the same region were also amplified by combining the same reverse primers with gene-specific forward primers (HmN_000800300 Fwd: TGCGTTCCCTACTAATACTC; HmN_000625700 Fwd: AAACCCTCGAATGTCCTAAT; HmN_000604600 Fwd: TTGATCGTCGGAAATCCTAT).

### Orthologous Relationships and Conservation of Gene Target

Phylogenetic orthology inference among all the species' genes was estimated using OrthoFinder v2.5.4 (Emms and Kelly 2019) with default setting, Step I in Fig. 1. The longest annotated isoform for standard gene models, or the corrected annotation for chimeric genes, was used. Additionally, genes containing unknown amino acids were excluded from the analysis. The predicted HOGs were considered an approximation of protein family membership. Functional annotation was carried out with InterProScan v5.62-94.0 (Jones et al. 2014). Interpro entries assigned to one member were assumed to be valid for the entire HOG. Conservation of SL trans-splicing across different species was defined as the presence of genes from the same HOG with SL-ACEs identified in both species. Pairwise comparisons were performed using in-house scripts, and heatmaps were generated using the R package pheatmap (Kolde 2019). Similarities between the groups Cyclophyllidea (*E. granulosus*, *E. multilocularis*, *H. diminuta*, *H. microstoma*, *M. corti*, *Taenia asiatica*, *T. multiceps, T. saginata*, and *Taenia solium*), Basal Cestodes (*Schistocephalus solidus*, *S. proliferum*, and *S. erinaceieuropaei*), Schistosomatids (*S. bovis*, *S. haematobium*, S. *japonicum*, *S. mansoni*, and *Trichobilharzia regenti*), and Other Trematoda (*C. sinensis*, *F. gigantica*, *F. hepatica*, *Fasciolopsis buski*, *O. felineus*, *Paragonimus heterotremus*, and *Paragonimus westermani*) were shown with Upset graphs generated using the UpSetR package v1.4.0 (Conway et al. 2017). Genes showing extensive overlap (≥90%) in their genomic coordinates with other annotated genes were excluded, as this made the assignment of the SL-ACE unclear (see below in the Identification of Operons section). The first gene in a chimeric gene model was only included if it possessed a SL-ACE different from the one used to split the chimera into separate gene models.

### Identification of Operons

The distribution of intergenic gene distances between the original gene models encoded on the same strands was analyzed, aiming to determine an appropriate threshold for the identification of operon candidates. Gene model pairs reported to overlap were not included. Based on their distribution, we defined operon candidates as genes encoded on the same strand, with an intergenic distance of ≤300 bp, measured from their most proximal annotated isoforms to each other, Step H in Fig. 1. Identified chimeric genes were considered an operon regardless of the intergenic distance, based on the assumption that they were mis-annotated initially due to the detection of polycistronic pre-mRNAs in the annotation process. Mitochondrial genes and gene models overlapping more than 80% with another gene model were discarded. Candidate operons were considered conserved between two species if a conserved pair of HOGs was present in both genomes and if both species displayed evidence of SL trans-splicing in the gene corresponding to the second HOG in the pair. Pairwise comparisons were conducted with in-house scripts, and heatmaps were generated using the R package pheatmap (Kolde 2019). Comparison results within the groups Cyclophyllidea, Basal Cestodes, Schistosomatids, and Other Trematoda were represented with Upset graphs using the UpSetR package v1.4.0 (Conway et al. 2017).

### Gene Expression and Read Coverage at Cis-Splicing Junctions

Reads were mapped to the reference genomes using STAR v2.7.10b (Dobin et al. 2013) with the suggested parameters for the species genome size (–genomeChrBinNbits and –genomeSAindexNbases set to 12). Read counts were calculated using htseq-count v2.0.2 (Putri et al. 2022) with strand specificity disabled (“-s no”). To minimize the impact of mis-annotations, reads were considered if located anywhere within the maximum extension of each gene (the most distal coordinates of the annotated elements). Then, a second-pass protocol was conducted to identify cis-splicing reads on SL-ACEs, as described in the STAR user manual.

The competition between SL trans- and cis-splicing was evaluated by classifying SL-ACEs based on its location within the predicted gene model: (i) Upstream Mono, the SL-ACE is located upstream the CDS of a monocistronic or the first gene in the operon; (ii) Upstream Poly, the SL-ACE is located upstream the CDS of a gene encoded in an operon loci, excluding the first gene; and (iii) Internal Sites, the SL-ACE is within a CDS. Significance of differences in the ratio between SL trans-splicing and cis-splicing (defined: as SL-bearing reads/(SL-bearing reads + cis-splicing reads) were evaluated using the Kruskal–Wallis rank sum test, implemented on the R package Stats v4.2.2 (R Development Core Team 2022). Boxplots were generated using the packages ggplot (Wickham 2016) and ggpubr (Kassambara 2023). SL-ACEs assigned to chimeric gene models were discarded.

### SL TAG Specialization on Specific Sites and Operon Resolution

Species with two or more SL tags in their transcriptome, comprising at least 5% of the observed read counts, were analyzed to explore SL specialization. Frequencies for all SL tags were calculated based on the observed read counts on SL-ACEs with at least five read counts. A chi-squared test was used to test TAG specialization; the *P*-values were corrected by false discovery rate (FDR). Calculations were carried out with the R package stats. SL TAGs from SL-RNAs potentially linked to operon resolution were identified by calculating the ratio of the number of reads per SL TAG to the total SL reads observed at sites potentially involved in operon resolution versus all other sites. A “Wilcoxon rank sum test” with continuity correction, as implemented on the R package stats, was used for testing bias toward operon resolution. SL-ACEs assigned to multiple genes, located on the opposite strand, or after their assigned gene model, were discarded.

### Evaluation of the Distances Between Cistrons Within Operons in *S. mansoni*

We reviewed operon candidates that met the following criteria: (i) SL-ACE identified in the downstream gene, (ii) 3′ UTR prediction reported for the upstream gene, (iii) no more than 1,000 bases between the end of the 3′ UTR and the first SL-ACE in the downstream gene, and (iv) no overlap or chimeric gene models in the entire operon.

### Selection of Particular Cases of Gain and Loss of Trans-Splicing on Individual HOGs

In order to study the evolutionary dynamics of gain and loss of trans-splicing, 41 HOGs were selected. These HOGs, which must show well-supported evidence of SL trans-splicing, were defined based on three conditions. First, HOGs must have members from at least two species used to identify chimeric gene models. Second, at least one of the genes from each represented species in the HOG had evidence of SL trans-splicing or have a TPM equal or higher than the median TPM of genes with confirmed SL trans-splicing. And third, at least five species represented in the HOG have genes subjected to SL. The protein sequences of the Selected HOGs were aligned with MAFFT and their phylogeny estimated with IQ-TREE v2.2.2.3 (Minh et al. 2020). Figures showing the estimated TPM and percentage of detected SL reads were generated in iTOL v6 (Letunic and Bork 2021). For these evaluations, all SL-ACEs were considered, regardless of read count. Internal SL-ACEs on chimeric gene models were manually evaluated and reassigned to the upstream portion when their position relative to the overall read mappings and exon structure suggested so.

### Selection of Particular Cases of Gain and Loss of Trans-Splicing

Candidate polycistronic loci were selected for manual inspection if a conserved pair of HOGs showed evidence of SL trans-splicing, were exclusive to either Cestoda or Trematoda, were observed in at least five species, and were distributed across four genera. Polycistronic loci associated with a HOG identified in the previous section were also analyzed.

## Supplementary Material

msaf228_Supplementary_Data

## Data Availability

The scripts utilized for this research are available on the GitHub repository: https://github.com/LBC-Iriarte/SL-Flatworms.git.
