# Supplementary material for: Evolution of SL-RNA Genes and Their Splicing Targets in Parasitic Flatworms
Source: Mol Biol Evol. 2025 Sep 23;42(11):msaf228. doi: 10.1093/molbev/msaf228 (PMC12582326; doi:10.1093/molbev/msaf228)

Supplementary Figure 1: Acceptor site motifs for SL-ACEs described in this study, generated with MEME using the oops site distribution (One occurrence per sequence). Species are ordered to match the species tree. With Cestodes in the left column and trematodes right. Neither of the motifs found in *Mesocestoides corti*'s match the SL- ACE location, likely due to limited data, and was excluded.

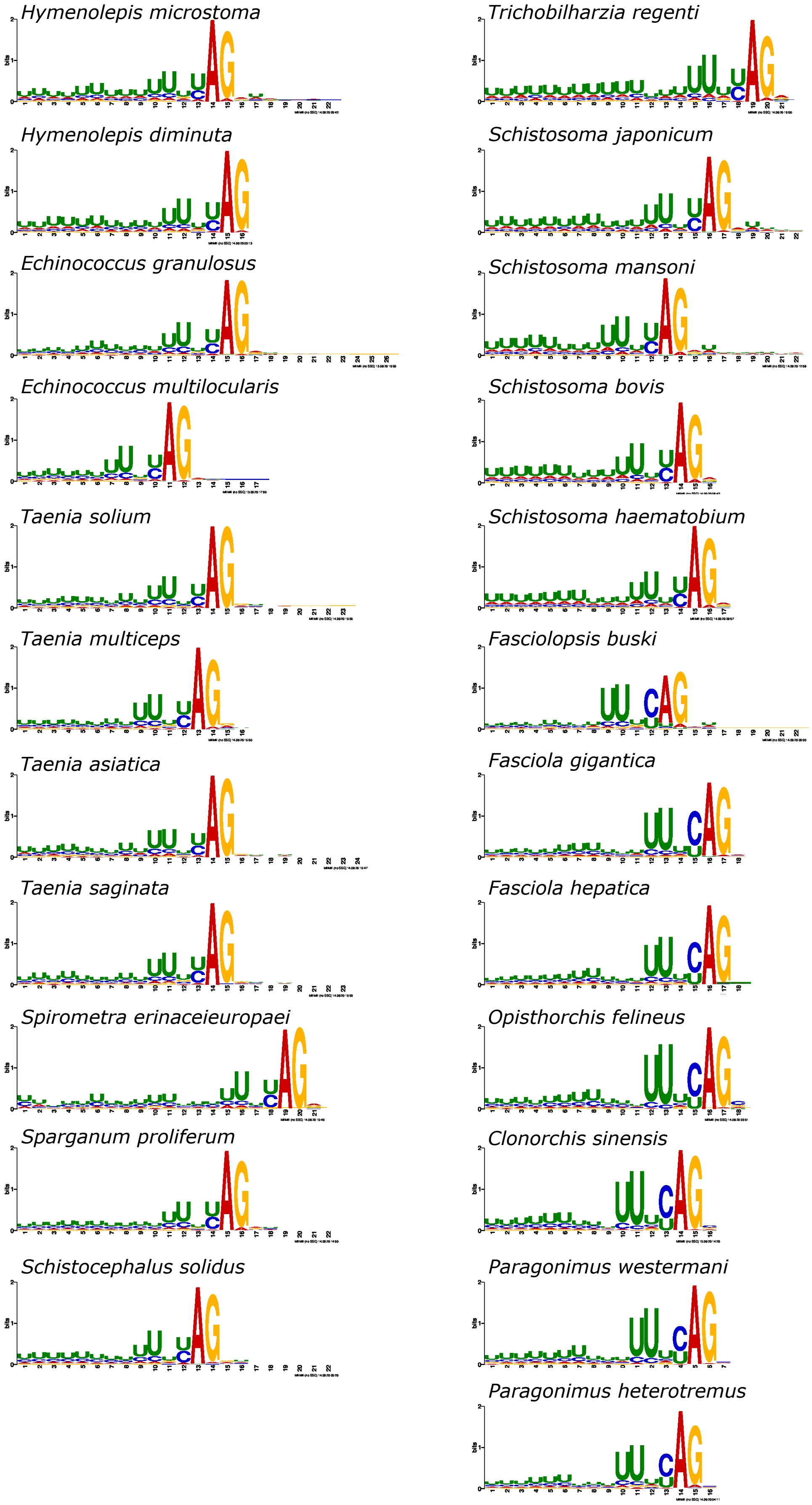

Supplement: msaf228_Supplementary_Data [file msaf228_supplementary_data.zip › Supplementary Figure 1 - 24052025.pdf]
