# Supplementary material for: Evolution of SL-RNA Genes and Their Splicing Targets in Parasitic Flatworms
Source: Mol Biol Evol. 2025 Sep 23;42(11):msaf228. doi: 10.1093/molbev/msaf228 (PMC12582326; doi:10.1093/molbev/msaf228)
Supplement: msaf228_Supplementary_Data [file msaf228_supplementary_data.zip › Supplementary Figure 2 - 24052025.pdf]

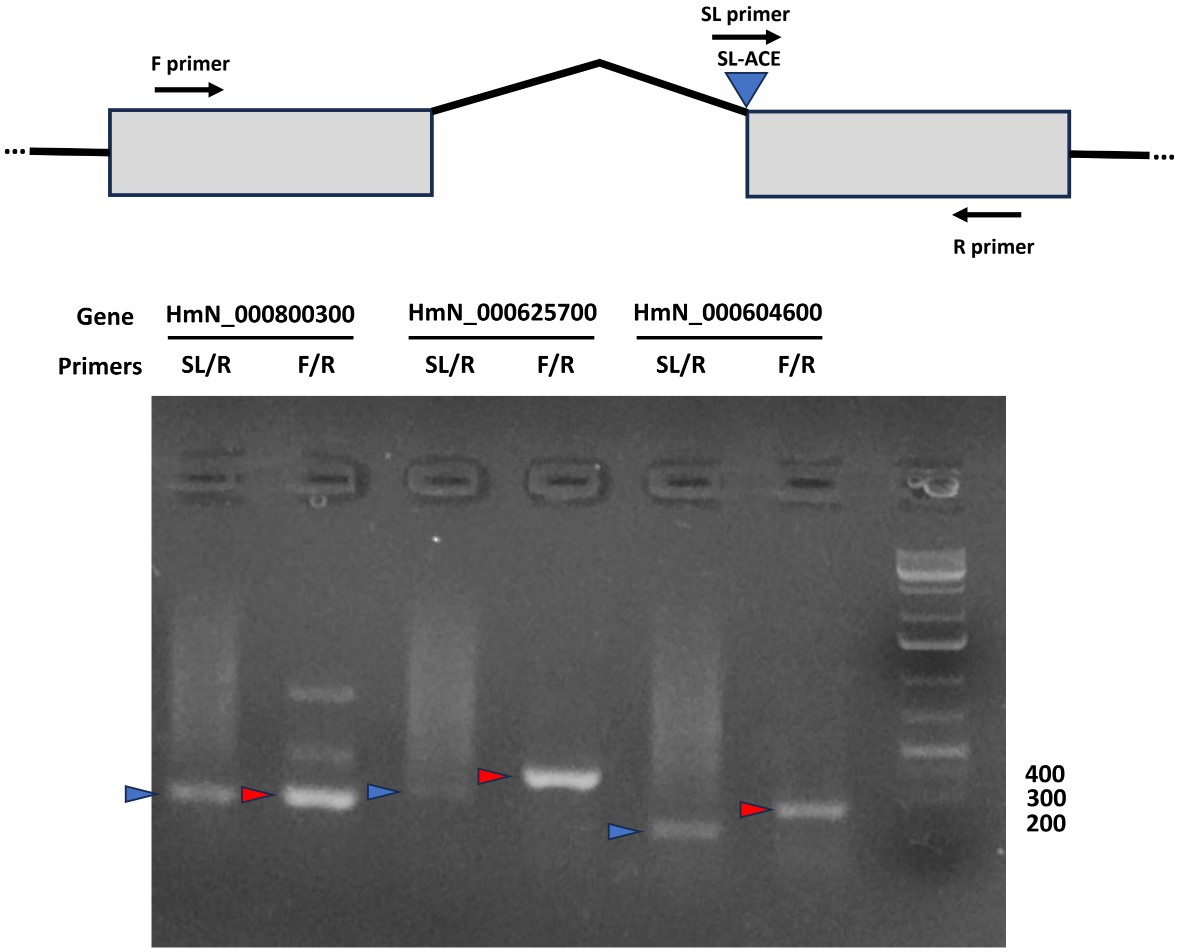

Supplementary Figure 2: Confirmation of internal SL-ACEs in Hymenolepis microstoma. Three internal SL-ACEs in three different H. microstoma genes were confirmed by reverse transcription PCR with cDNA from adult worms, by combining a SL3 forward primer and a gene-specific reverse primer (amplicons of the expected size are indicated with blue arrowheads in the gel). For each gene, we confirmed the existence of cis-splicing in the same 3' splicing acceptor site by PCR with the same reverse primer, combined with a gene-specific forward primer (amplicons of the expected size are indicated with red arrowheads in the gel).
