# Supplementary material for: Evolution of SL-RNA Genes and Their Splicing Targets in Parasitic Flatworms
Source: Mol Biol Evol. 2025 Sep 23;42(11):msaf228. doi: 10.1093/molbev/msaf228 (PMC12582326; doi:10.1093/molbev/msaf228)
Supplement: msaf228_Supplementary_Data [file msaf228_supplementary_data.zip › Supplementary Figure 3 - 24052025.pdf]

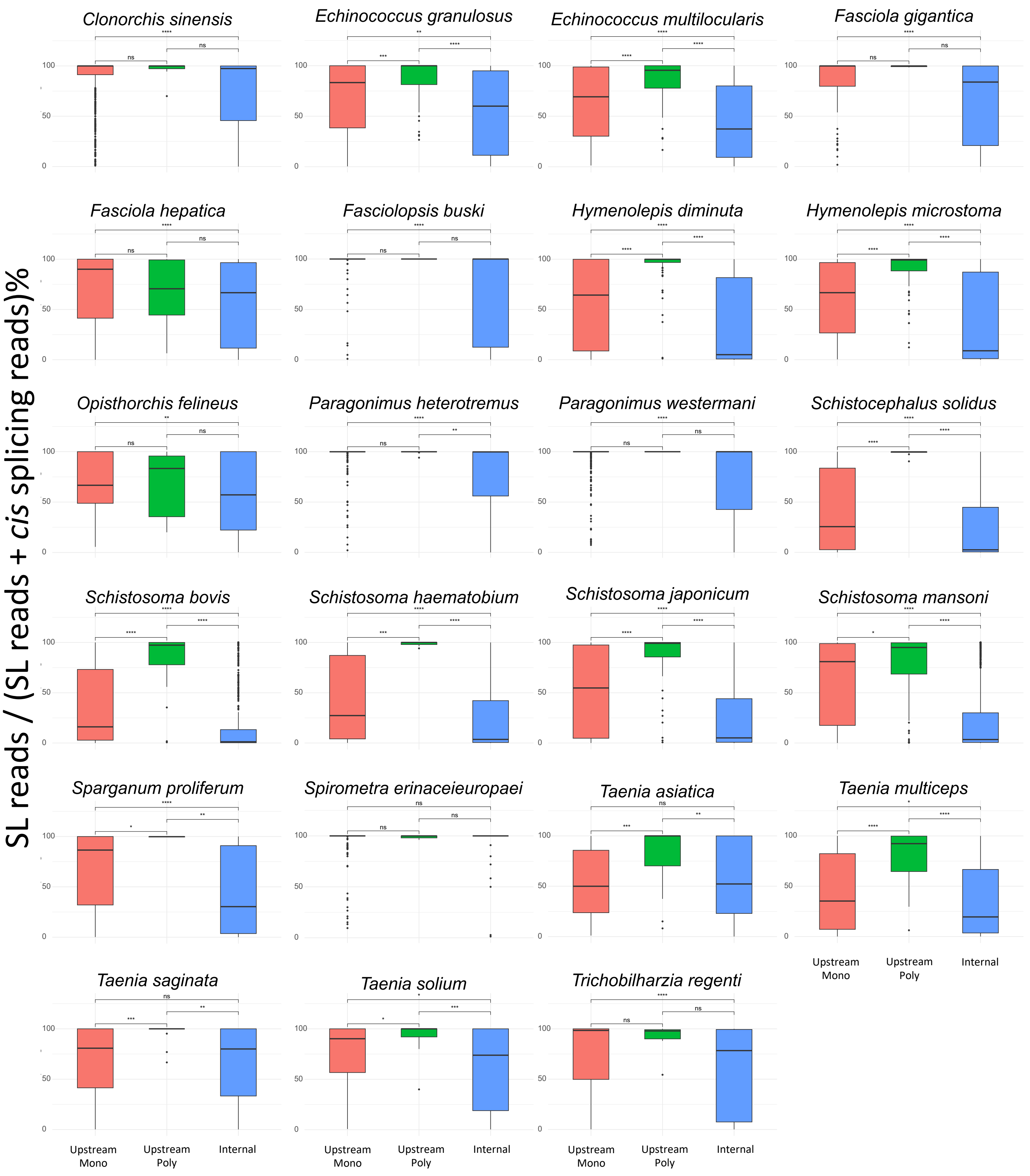

Supplementary Figure 3: Proportion of SL bearing reads relative to all splicing reads mapped to different sets of SL-ACEs: 1) Upstream Mono (Red), reads mapped upstream the coding region of a monocistronic gene or upstream the first gene of an operon, 2) Upstream Poly (Green), reads mapped upstream the coding region of a gene related to polycistronic gene resolution, and 3) Internal (Blue), reads mapped to SL-ACEs located within the coding sequence. Percentage of SL *trans*-splicing relative to *cis*-splicing found at each type of SL-ACE in four selected species. Pairwise comparisons were carried out with the Wilcoxon Rank Sum and Signed Rank Tests, significant levels of the pairwise comparisons are displayed with the following code: ns (p > 0.05), * (p <= 0.05), ** (p <= 0.01), *** (p <= 0.001), and **** (: p <= 0.0001). *Mesocestoides corti* was discarded due to limited data. SL-ACEs assigned to a chimeric gene model were not considered.
