# Supplementary material for: Evolution of SL-RNA Genes and Their Splicing Targets in Parasitic Flatworms
Source: Mol Biol Evol. 2025 Sep 23;42(11):msaf228. doi: 10.1093/molbev/msaf228 (PMC12582326; doi:10.1093/molbev/msaf228)
Supplement: msaf228_Supplementary_Data [file msaf228_supplementary_data.zip › Supplementary Figure 4 - 24052025.pdf]

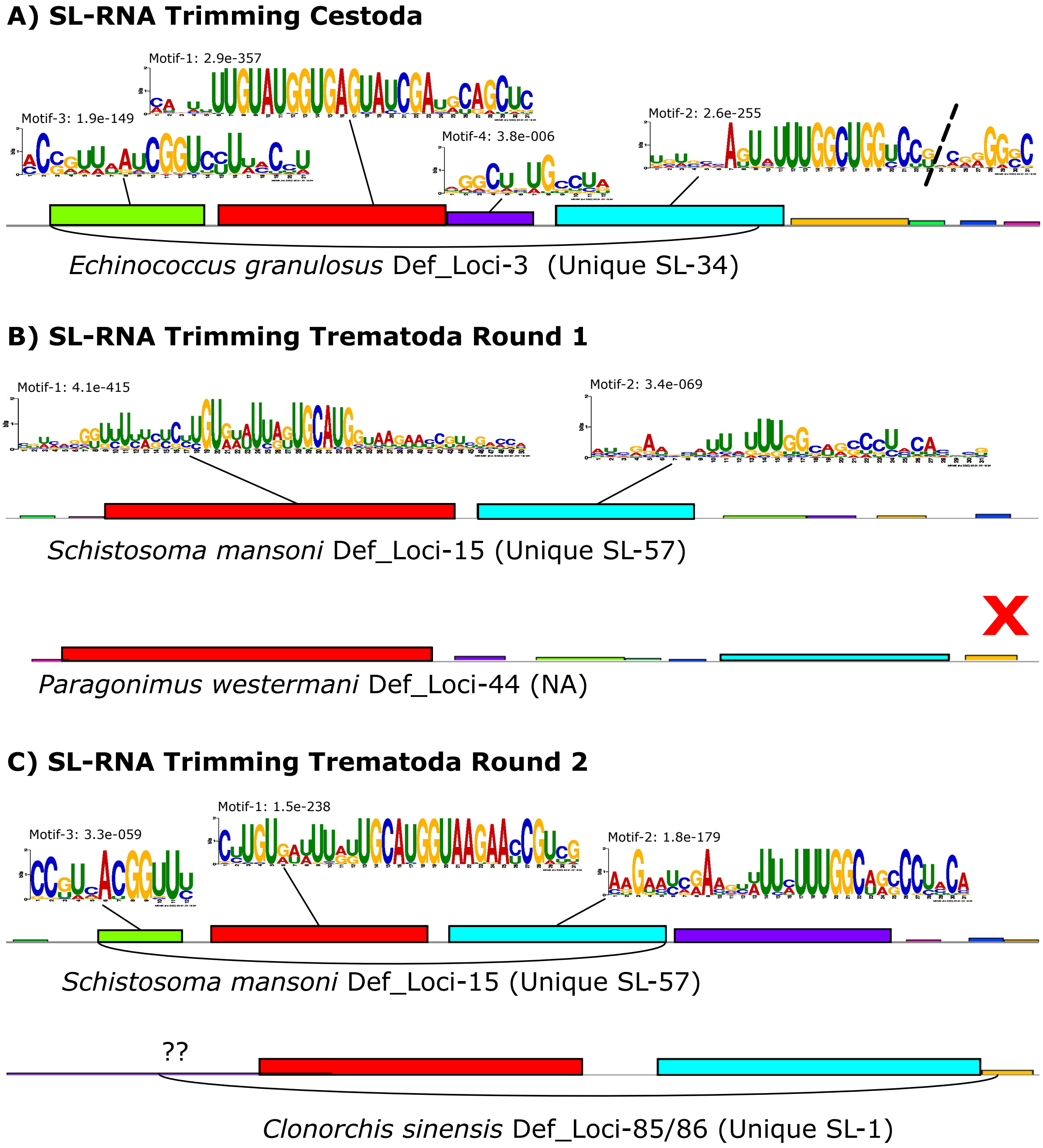

Supplementary Figure 4: Overview of the SL RNAs trimming process based on MEME motifs, with selected examples. In Cestodes the process was straightforward as four highly conserved motifs were identified (A). Trimming was conducted from the start of Motif-3 to within Motif-2. This approach was taken because the less conserved second half was more difficult to recognize outside Taeniidae. Trematoda, on the other hand, required two rounds of trimming. First, only two motifs were predicted, but their boundaries were unclear (B). After the exclusion of sequences with poor matches for Motif-2, like *Paragonimus westermani* Def_Loci-44, the analysis was repeated in a second round (C). As a result, three motifs were identified consistently in the selected sequences, including the reference SL-RNA for Trematoda and the boundaries of Motifs -2 and -3 were defined. The loci Def_Loci-85 and -86 of Clonorchis sinensis were included in the final analysis despite lacking Motif-2 because it's SL TAG "Trematoda_E" was found in the species *C. sinensis*, *Fasciola gigantica*, *Fasciola hepatica* and *Fasciolopsis buski*. Despite low numbers, the phylogenetic distribution of SL TAG "Trematoda_E" suggests that it exists within this lineage, even if it may possess limited functionality.
