# Supplementary material for: Evolution of SL-RNA Genes and Their Splicing Targets in Parasitic Flatworms
Source: Mol Biol Evol. 2025 Sep 23;42(11):msaf228. doi: 10.1093/molbev/msaf228 (PMC12582326; doi:10.1093/molbev/msaf228)
Supplement: msaf228_Supplementary_Data [file msaf228_supplementary_data.zip › Supplementary Figure 5 - 24052025.pdf]

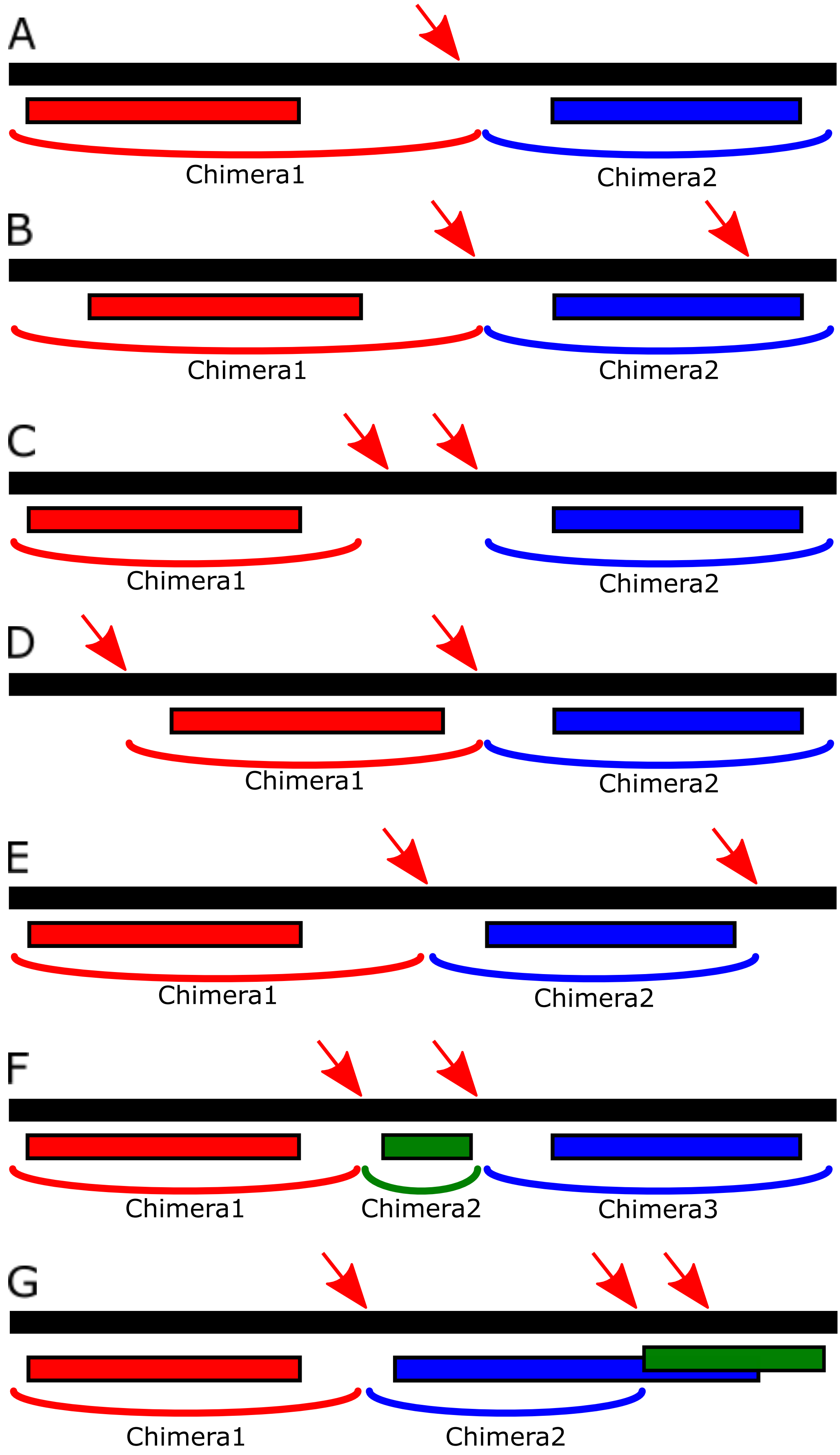

Supplementary Figure 5: Schematic representation of the resolution of several gene models identified in this study. The annotated transcript is represented in black, with SL acceptor sites indicated by red arrows. BLAST hits with genes in reference species shown as colored boxes, red, blue, and green. The chimeric portions defined as a result of the analysis are represented by arches. A) A single SL-ACE that separates two halves with different BLAST hits. B) Similar to A, but with an additional SL-ACE that does not correspond to changes in the BLAST hits. In this case, the second SL-ACE is ignored. C) Multiple SL-ACEs that separate both sets of BLAST hits. The positions of the SL-ACEs were used to define the intergenic space, excluding the sequence between them. D and E) SL-ACEs located in upstream or downstream regions define sequences with no BLAST hits. Regions with no BLAST hits are subsequently trimmed. F) SL-ACEs define three or more regions in the chimeric gene model, each with its own BLAST hit. As a result, each region is annotated as a chimera. G) Subdivisions which have unclear boundaries between genes were not considered.
