# Supplementary material for: Evolution of SL-RNA Genes and Their Splicing Targets in Parasitic Flatworms
Source: Mol Biol Evol. 2025 Sep 23;42(11):msaf228. doi: 10.1093/molbev/msaf228 (PMC12582326; doi:10.1093/molbev/msaf228)
Supplement: msaf228_Supplementary_Data [file msaf228_supplementary_data.zip › Supplementary File 1.pdf]

Supplementary File 1: Sequences of the unique SL-RNA loci identified for each species.

>Unique_SL_1
AAUGUUCGGUUUUCUGCCGUGUAUAUUAGUGCACGGUAAUAAUCGACUCCGACCUAUGGUCGGA
UGAAUUCUUUGGCUAGCCCACC
>Unique_SL_2
CCGAUAAAUCGGUCCUUGCCUGCACUUUUGUAUGGUGAGUAUCGAUGCAGCUCAGGCUCUGCCU
ACGAGCUGACAGUAUUUGGCUGGUCCGACGAGGGC
>Unique_SL_3
CCGAUAAAUCGGUCCUUGCCUGCACUUUUGUAUGGUGAGUAUCGAUGCAGCUCAGCCUCUGCCU
ACGAGCUGACAGUAUUUGGCUGGUCCGACGAGGGC
>Unique_SL_4
CCGAUAAAUCGGUCCUUACCUGCACUUUUGUAUGGUGAGUAUCGAUGCAGCUCAGGCUCUGCCU
ACGAGCUGACAGUAUUUGGCUGGUCCGACGAGGGC
>Unique_SL_5
CCGAUAAAUCGGUCCUUGCCUGCACUCUUGUAUGGUGAGUAUCGAUGCAGCUCAGCCUCUGCCU
ACGAGCUGACAGUAUUUGGCUGGUCCGACGAGGGC
>Unique_SL_6
CCGAUAAAUCGGUCCUUACCUGCACUUCUGUAUGGUGAGUAUCGAUGCAGCUCAGGCUCUGCCU
ACGAGCUGACAGUAUUUGGCUGGUCCGACGAGGGC
>Unique_SL_7
CCGAUAAAUCGGUCCUUGCCUGCACUCUUGUAUGGUGAGUAUCGAUGCAGCUCAGGCUCUGCCU
ACGAGCUGACAGUAUUUGGCUGGUCCGACGAGGGC
>Unique_SL_8
ACCGUUAAUCGGUCCUUACCUUGCAGUUUUGUAUGGUGAGUAUCGAUGCAGCUGAGGCUGUGCC
UACGAGCUGACCCAGUAUUUGGCUGGUCCUUCGAGGGC
>Unique_SL_9
ACCGUUAAUCGGUCCUUACCUUGCAGUUUUGUAUGGUGAGUAUCGAUGCAGCUGAGGCUGUGCC
UACGAGCUGACCCAUUAUUUGGCUGGUCCUUCGAGGGC
>Unique_SL_10
ACCGUUAAUCGGUCCUUACCUUGCAGUUUUGUAUGGUGAGUAUCGAUGCAGCUGAGGCUGUGCC
UACGAGCUGACCCAGUAUUUGGCUGGUCCUUCGGGGGC
>Unique_SL_11
ACCGUUAAUCGGUCCUUACCUUGCAGUUUUGUAUGGUGAGUAUCGAUGCAGCUGAGGCUGUGCC
UACGAGCUGACCCAGUAUUUGGCCGGGCCCUCGAGGGC
>Unique_SL_12
ACCGUUAAUCGGUCCUUACCUUGCAGUUUUGUAUGGUGAGUAUCGAUGCAGCUGAGGCUGUGCC
UACGAGGUGACCCAGUAUUUGGCUGGUCCUUCGAGGGC
>Unique_SL_13
CCUUAACGGUUCUCUGCCCUGUAUAUUAGUGCAUGGUAAGAAUCGUUGGACCAUCGGUCCAAAC
CCAUUAUUUGGCUAGCCUCCA
>Unique_SL_14
CCUUAACGGUUCUCUGCCCUGUAUAUUAGUGCAUGGUAAGAAUCGUUGGACCAUCGGUCCAAAC
CCAUUAAUUUGGCUAGCCUCCA
>Unique_SL_15
ACCGAUUAUCGGUCUUACCAUAAAACUUGUAUGGUGAGUAUCGAGGCAGCGUCUGAGCUGUUCA
AUUGUUUGGCUGGUCCUGCGGGGCC
>Unique_SL_16

CACCGAUUAUCGGUCUUACCAUAAAACUUGUAUGGUGAGUAUCGAGGCAGCACUUUAGCUGUCCA
AUUAUUUGGCUGGUCUUUCGGGACC
>Unique_SL_17
CCUAUACGGUUCUCUGCCGUGUGAAUUAGUGCAUGGUAAGAAUCGACUCCGGCCUAUGGUCGGA
UGAAUUCUUUGGCUAGCCCACC
>Unique_SL_18
UGGUUAUGGUUUUACUCUUGUGAUUUGUUGCAUGCUAAGAACCGUCGACCAAGAAUCGAAGUUU
UCUUUGGCAGCCCUACA
>Unique_SL_19
CCGUCACGGUUUUACUCUUGUGAUUUGUUGCAUGGUAAGAACCGUCGACCAAGAAUCGAAGUUU
UCUUUGGCAGCCCUACA
>Unique_SL_20
UGGUCACGGUCUUACUCUUGUGAUUUGUUGCAUGCUAAGAACCGUCGACCAAGAAUCGAAGUUU
UCUUUGGCAGCCCUACA
>Unique_SL_21
CCGUCACGGUUUUACUCUUGUGAUUUGUUGCAUGGUAAGAACCGUCGACCAGAAUCGAAGCUUU
CUUUUGCCAGCCCUGCA
>Unique_SL_22
CCGUCACGGUUUUACUCUUGUGAUUUGUUGCAUGGUAAGAACCGUCGACCAAGAAUCGAAGCUU
UCUUUGCAGCCCUGCA
>Unique_SL_23
CCGUCACGGUUUUACUCUUGUGAUUUGUUGCAUGGUAAGAACCGUCCGACCAAGAAUCGAAGCU
UUCUUUGCCAGCCCUGCA
>Unique_SL_24
CCGUCACGGUUUUACUCUUGUGAUUUGUUGCAUGGUAAGAACCGUCGACCAAGAAUCGAAGCUU
UCUUUGCCAGCCCUGCA
>Unique_SL_25
CCGUCACGGUUUUACUCUUGUGAUUUGCAUGGUAAGAACCGUCGACCAAGAAUCGAAGUUUUCU
UUGGCAGCCCUACA
>Unique_SL_26
ACCGUUUAACGGUCCUUACCUCACUCGUUGUAUGGUGAGUACCGACAUGACUCGCUAGAGUUAU
GCUAGUCUUUGGCUGGCCCGCAAGGGCC
>Unique_SL_27
ACCGUUCAACGGUUCUUGCCUUGCUCGUUGUAUGGUGAGUACCGACAUGACUCAUUGAAGUCAU
GCAAGUCUUUGGCUGGUCCGAAAGGGCC
>Unique_SL_28
ACCGUUAAUCGGUCCUUACCUUGCAAUUUUGUAUGGUGAGUAUCGAUGCAGCUCGGGCUCUGGC
UACGAGCUGACCCAGUAUUUGGCUGGUCCGUCAAGGGC
>Unique_SL_29
ACCGUUAAUCGGUCCUUACCUUGCAAUUUUGUAUGGUGAUAUCGAUGCAGCUCGGGCUCUGGCU
ACGAGCUGACCCAGUAUUUGGCUGGUCCGUCAAGGGC
>Unique_SL_30
ACCGUUAAUCGGUCCUUACCUUGCAAUUUUGUAUGGUGAGUAUCGAUGCAGCUCGGGCUCUGGC
UACGAGCUGACCAGUUUGGCUGGUCCGUCAAGGGC
>Unique_SL_31
ACCGUUAAUCGGUCCUUACCUUGCAAUUUUGUAUGGUGAGUAUCGAUGCAGCUCGGGCUCUGGC
UACGAGCUGACCCAGAAUUUGGCUGGUCCGUCAAGGGC
>Unique_SL_32
CCUAUACGGUUCUCUGCCGUGUAUAUUAGUGCAUGGUAAGAAUCGACUCCGGCCUAUGGUCGGA

UGAAUUCUUUGGCUAGCCCACC
>Unique_SL_33
GUCGAGUUACUCGAUUCUUGCCUGCAGUUGUGUAUGGUGAGUAUCGAUGCAGCUGAGGCUCUGC
CUACGAGCUGACGCAGUAUUUGGCUGGUCCGACGAGGA
>Unique_SL_34
ACCGUUAAUCGGUCCUUACCUUGCAAUUUUGUAUGGUGAGUAUCGAUGCAGCUCAGGCUGUGCC
UACGAGCUGACAGUAUUUGGCUGGUCCGACGAGGAC
>Unique_SL_35
CCUUAACGGUUCUCUGUCCCUGUAUAUUAGUGCAUGGUAAGAAUCGUUGGACCAUCGGUCCAAA
CCCAUUAUUUGGCUAGCCUUCC
>Unique_SL_36
UAACAGCGGUUCACUGCCCUGUAUAUUAGUGCAUGGUAAGAAUCGUUGGACCAUCGGUCCAAAC
CCAUUAUUUGGCUAGCCUCCA
>Unique_SL_37
CCUUAACGGUUCUCUGCCCUGUAUAUUAGUGCAUGGCAAGAAUCGUUGGACCAUCGGUCCAAAC
CCAUUAUUUGGCUAGCCUCCA
>Unique_SL_38
UAACGGUUCUCUGCCCUGCUGUAUAUUAGUGCAUGGUAAGAAUCGUUGGACCAUCAGUCCAAAU
CCAUUAUUUGGCUAGCCUCG
>Unique_SL_39
UAACGGUUCUCUGCCCUGCUGUGUAUUAGUGCAUGGUAAGAAUCGUUGGACCAUCAGUCCAAAU
CCAUUAUUUGGCUAGCCUCUA
>Unique_SL_40
CCGAUUAAUCGGUCUUACCUUGCAAUUUUUGUAUGGUGAGUAUCGAUACAGCACUUGGCUCUGC
CUUGUGUCUGUGAGAUUCUUUGGCUGGUCCUUGCGGGCC
>Unique_SL_41
CCGAGUAUUCGGUCUUACUAUACGAACUUGUAUGGUGAGUAUCGUGACAGCUCUCGUUUUGCUU
GAGCUGUAGAGUUUUUGGCUGGUCCGCGAGGGCC
>Unique_SL_42
CCGGAUAAUCGGUCUUACUGUACUAACUUGUAUGGUGAGUAUCGAAUCAGUUCUUGCUUUGCUC
GAACUGUACUUUUUGGCUGGCCCGCGAGGGCC
>Unique_SL_43
ACCGUUUUGCGGUCUUGCCAUAAAGUUUGUAUGGUGAGUAUCGAGGCAGCUCUUUAGCUGUCCA
AUUGUUUGGCUGGUCUUACGGGACC
>Unique_SL_44
ACCGUUUACCGGUCUUACCUUGCAAUUUUUGUAUGGUGAGUAUCGAUACAGCGCUUGGCUUUGC
CUUGUGUCUGUAAAAUUCUUUGGCUGGUCCUAACGGGCC
>Unique_SL_45
ACCGAUUAUCGGUAUUUACCACAUUUAUUGUAUGGUGAGUAUCGAUGCAGCUCAUGUAGCUGUG
AAAGAAUUUGGCUGGCCCGGAAGGGCC
>Unique_SL_46
ACCGUUUAUCGGUGUUUGCCACAUUCAUUGUAUGGUGAGUAUCGAUGCAGCUCAAGCAGCUGUG
GAAGUGUUUGGCUGGCCCGAAAGGGCC
>Unique_SL_47
UAUAUAUGGUUCUCUGCCGUGUAUCAGUGCAUGGUAAGAAUCGAGUUCGACUCACUCAGUUGGU
CGAAUUAAUUAUUUGGCUAGCC
>Unique_SL_48
CCGUCACGGUUUUACUCUUGUGAUUUGUUGCAUAGUAAGAACCGUCGACCAAGAAUCGAAGUUU
UCUUUGGCAGCCCUACA

>Unique_SL_49
CCGUCACGGUUUUACUCUUGUGAUUUGUUGCAUGGUAAGAACCGUCGACCAAGAAUCAAAGUUU
UCUUUGGCAGCCGUACA
>Unique_SL_50
CAGUCGCGCUUUUACUCUUGUGAUUUGUUGCAUGGUAAGAACCGUCGACCAGGAAUCGAAGUUU
UUUUGGAACUCCCACA
>Unique_SL_51
UUUUCACGGUUUUACUCUUGUGAUUUUUUGCAUGGUAAGAACCGUCGACCAAGAGUCGAAGGUU
UCUUUGCCAGCCCUUCA
>Unique_SL_52
CCGUCACGGUUUUACUCUUGUGAUUUGUUGCAUGGUAAGAACCGUCGACCAAGAAUCGAAGCUU
UCUUUGAACUGACAGUU
>Unique_SL_53
CCGUCACGGUUUUACUCUUGUGAUUUGUUGCAUGGUAAGAACCGUCGACCAAGAAUCGAAGCUU
UCUUAGACACUGAACA
>Unique_SL_54
UACUCACGGUUUUACUCUUGUGAUUUGUUGCAUGGUAAGAACUGUCGACGAAGAAUCGAAGUUU
UCUUUGGCAGCCCUACA
>Unique_SL_55
UAGUCACGGUCUUACUAUUGUGAUUUGUUGCAUGGUAAGAACCGUCGACCAAGAAUCGAAGUUU
UCUUUGGCAGCCCUACA
>Unique_SL_56
CCGUCACGGUUUUACUCUUGUGAUUUAUUGCAUGGUAAGAACCGUCGACCAAGAAUCGAAGUUU
UCUUUGGCAACCCUACA
>Unique_SL_57
CCGUCACGGUUUUACUCUUGUGAUUUGUUGCAUGGUAAGAACCGUCAACCAAGAAUCGAAGUUU
CUUUUGGCAGCCCUACA
>Unique_SL_58
GGCUGACGGUUUUACUCUUGUGAUUUGUUGCAUGGUAAGAACCGUCGACCAAGAAUCGAAGUUU
UCUUUGGCAGCCCUACA
>Unique_SL_59
ACCGUUCAACGGUUCUUGCCUUGCUCGUUGUAUGGUGAGUACCGACAUGACUCAUUAAAGUCAU
GCAAGUCUUUGGCUGGUCCGAAAGGGCC
>Unique_SL_60
ACCGUAGAUCGGUUCUUACCCUACGAUAUUGUAUGGUGAGUAUCGAUACGGCUCGAGACUACGA
GCUGUUAUUGUUUGGCUGGUCCUAACAGGGG
>Unique_SL_61
ACCGUAGAUCGGUUCUUACCCUACGAUAUUGUAUGGUGAGUAUCGAUACGGCUCGAGACUACGA
GCUGUCAUUGUUUGGCUGGCUCCUAGCGGGG
>Unique_SL_62
CCGUUUAGUCGGUCUUACCUACGAGUGUUGUAUGGUGAGCAUCAAUAUAGCUAGGGCUCUGCCC
AGGAGCUAUCGUAGUAUUUGGCUGGCCCAGCGUGGGC
>Unique_SL_63
GCCGUUUAGUCGGUCUUACCUACGAGUGUUGUAUGGUGAGCAUCGAUGCAGCUCGGGCUUUGU
CCAGGAGCUGUUGUAGUAUUUGGCUGGCCCGGCGGGGCC
>Unique_SL_64
ACCGUAGAUCGGUUCUUACCCUACGCUAUUGUAUGGUGAGUAUCGAUACGGCUCGGGCUAAGCC
UACGAGCUGUUAUUCUUUGGCUGGUCCUGCUGAGGG
>Unique_SL_65

CCGUCACGGUUUUACUCUUGUGAUUUAUUGCAUGGUAAGAACCGUCGACUAAGAAUCGAAGCAUU
AUUUGGCAGCUCCUCA
>Unique_SL_66
CCGUCACGGUUUUACUCUUGUGAUUUAUUGCAUGGUAAGGACCGUCGACCAAGAAUCGAAUCAU
UAUUUGGCAGCCCCUCA
>Unique_SL_67
CCGUCACGGUUUUACUCUUGUGAUUUAUUGCAUGGUAAGAACCGUCGACCAAGAAUCGAAUCAUU
AUUUGGCAGCCCUUCA
>Unique_SL_68
CCGUCAUGGUUUUACUCUUGUGAUUUAUUGCAUGGUAAGAACCGUCGACCAAGAAUCGAACCAUU
AUUUGGCAGCCCCUCG
>Unique_SL_69
CCGUCACGGUUUGACUCUUGUGAUUUAUUAUUGCAUGGUAAGAACCGUCGACCAAGAAUCGAAG
CAUUAUUUGGCAAAACA
>Unique_SL_70
CCGUCACGGUUUUACUCUUGUGAUUUAUUGCAUGGUAAGAACCGUCGACCAAGAAUCGAAUCAUU
AUUUGGCAGCCUCUCA
>Unique_SL_71
CCGUCACGGUUUUACUCUUGUGAUUUAUUGCAUGGUAAGAACCGUCGACCAAGAAUCGAAGCAUU
AUUUGGCAGCCCCCCC
>Unique_SL_72
ACCGUUAAUCGGUCCUUACCUUGCAAUUUUGUAUGGUGAGUAUCGAUGCAGCUCAGGCUGUGCC
UACGAGCUGACCCAGUAUUUGGCUGGUCCUUCGAGGGC
>Unique_SL_73
CCUAUACGGUUCUCUGCCGUGUAUCAGUGCAUGGUAAGAAUCGAGUUCGACUCACAUCGUUGGU
CGAAUAGAUUAUUUGGCUAGCCUCCA
>Unique_SL_74
CCUAUACGGUUCUCUGCCGUGUAUAUUAGUGCAUGGUAAGAAUCGAAUUCGACCUAUGGUCGAA
UAAAUUCUUUGGCUAGCCUCUU
>Unique_SL_75
CCGUCACGGGUUUUACUCUUGUGAUUUGUUGCAUGGUAAGAACCGUCGACCAAGAAUCGAAGUU
UUCUUUGGCAGCCCUAC
