# Supplementary material for: Evolution of SL-RNA Genes and Their Splicing Targets in Parasitic Flatworms
Source: Mol Biol Evol. 2025 Sep 23;42(11):msaf228. doi: 10.1093/molbev/msaf228 (PMC12582326; doi:10.1093/molbev/msaf228)
Supplement: msaf228_Supplementary_Data [file msaf228_supplementary_data.zip › Supplementary File 10 - 14082025.pdf]

Supplementary File 10: Neighbor joining trees of the potential SL-RNA candidates found in Cestodes (A) and Trematodes (B) in Newick format. For each SL-RNA candidate the ID indicates: 1) CD-HIT Cluster ID, 2) Species 3) Loci ID 4) Repeat element found within a 500 bases. Note that points 1 and 4 are only included if relevant.

A) Cestodes

((((((((((((Egranulosus_Def_Loci-
3:0.02809,Cestodes_Echinococcus_granulosus_AJ292377.1:0.00000):0.01872,(((Ces_cdhit_C
lus-57_Emultilocularis_Def_Loci-1:0.00000,Emultilocularis_Def_Loci-
76:0.10217):0.01555,Cestodes_Echinococcus_multilocularis_AJ292365.1:0.00000):0.01476,
Cestodes_Echinococcus_multilocularis_Emu.SL1:0.00000):0.03856):0.01657,((((((Ces_cdhi
t_Clus-1_Emultilocularis_Def_Loci-19:0.03721,Ces_cdhit_Clus-
0_Emultilocularis_Def_Loci-21:0.01560):-
0.01961,Cestodes_Echinococcus_multilocularis_Emu.SL2a:0.00000):0.02229,Cestodes_Echin
ococcus_multilocularis_Emu.SL2b:0.00000):0.01037,Cestodes_Echinococcus_multilocularis
_Emu.SL2d:0.00000):0.00000,Cestodes_Echinococcus_multilocularis_Emu.SL2c:0.00000):0.0
4706,((Ces_cdhit_Clus-54_Taenia_multiceps_Def_Loci-
1:0.00000,Cestodes_Taenia_solium_AJ428456.1:0.00000):0.01427,Ces_cdhit_Clus-
55_Taenia_multiceps_Def_Loci-8:0.04590):0.04442):0.01523):0.02456,((Ces_cdhit_Clus-
35_Hdiminuta_Def_Loci-2:0.12604,((Hmicrostoma_Def_Loci-
2:0.00000,Cestodes_Hymenolepis_microstoma_Hmic_SL4_SLC:0.00000):0.05841,(Ces_cdhit_Cl
us-77_Hmicrostoma_Def_Loci-
3:0.00000,Cestodes_Hymenolepis_microstoma_Hmic_SL1_SLD:0.00000):0.03278):0.02209):0.0
1830,((Taenia_multiceps_Def_Loci-20:0.07160,Taenia_saginata_Def_Loci-
1:0.07658):0.05011,Taenia_solium_Def_Loci-
1:0.09380):0.11266):0.02154):0.03873,((((Hdiminuta_Def_Loci-
1:0.07276,Hdiminuta_Def_Loci-7:0.13084):0.00812,(Hmicrostoma_Def_Loci-
1:0.00000,Cestodes_Hymenolepis_microstoma_Hmic_SL3_SLB:0.00000):0.03898):0.06738,(Hmi
crostoma_Def_Loci-
6:0.00000,Cestodes_Hymenolepis_microstoma_Hmic_SL2_SLA:0.00000):0.06241):0.02386,(Mes
ocestoides_corti_Def_Loci-1:0.03727,Mesocestoides_corti_Def_Loci-
2:0.06334):0.09562):0.01271):0.02549,(Taenia_saginata_Def_Loci-
3:0.14877,Taenia_solium_Def_Loci-2:0.04463):0.06814):0.06676,(((Ces_cdhit_Clus-
7_Sparganum_proliferum_Def_Loci-2_LINE__Penelope_LINE__RTE-
BovB:0.04019,Spirometra_erinaceieuropaei_Def_Loci-
4:0.04240):0.02087,Spirometra_erinaceieuropaei_Def_Loci-
5_LINE__Penelope:0.04291):0.12365,((Ces_cdhit_Clus-11_Sparganum_proliferum_Def_Loci-
4:0.03271,Sparganum_proliferum_Def_Loci-
6:0.00975):0.04899,Spirometra_erinaceieuropaei_Def_Loci-
2_LINE__CR1:0.06043):0.06665):0.04462):0.09849,Schistocephalus_solidus_Def_Loci-
9:0.32062):0.05863,Schistocephalus_solidus_Def_Loci-
32_LINE__Penelope:0.33544):0.01762,((((((Hdiminuta_Def_Loci-
6_LTR__Gypsy:0.22202,Hdiminuta_Def_Loci-
5_LTR__Gypsy:0.16598):0.17578,Schistocephalus_solidus_Def_Loci-
16_LINE__CR1:0.22163):0.07728,(Schistocephalus_solidus_Def_Loci-
8:0.24330,Sparganum_proliferum_Def_Loci-

7_LINE__CR1:0.41377):0.05937):0.04062,((Schistocephalus_solidus_Def_Loci-
22_LINE__RTE-BovB:0.36332,Sparganum_proliferum_Def_Loci-
13:0.39633):0.05385,((((Schistocephalus_solidus_Def_Loci-23_DNA__TcMar-
Tigger_LINE__CR1:0.10433,Sparganum_proliferum_Def_Loci-
12_LINE__CR1:0.14487):0.05647,Sparganum_proliferum_Def_Loci-
18_LINE__CR1:0.30854):0.11572,Sparganum_proliferum_Def_Loci-17_LINE__CR1_LINE__RTE-
BovB:0.34990):0.03825,(Sparganum_proliferum_Def_Loci-
11:0.33311,(Sparganum_proliferum_Def_Loci-
14_LINE__CR1_LINE__Penelope:0.17142,Spirometra_erinaceieuropaei_Def_Loci-
19_LTR__Gypsy:0.41428):0.14512):0.04449):0.02618):0.02951):0.01117,((Hmicrostoma_Def_
Loci-5:0.57516,(Schistocephalus_solidus_Def_Loci-
1_DNA__MULE:0.30497,Schistocephalus_solidus_Def_Loci-
26:0.33282):0.00670):0.07712,(Schistocephalus_solidus_Def_Loci-
12_LINE__Penelope_LINE__RTE-BovB:0.28986,Schistocephalus_solidus_Def_Loci-
20:0.33692):0.06694):0.02600):0.01406,(((Schistocephalus_solidus_Def_Loci-
31:0.32183,Sparganum_proliferum_Def_Loci-16_LINE__CR1_LINE__RTE-
BovB:0.22010):0.05702,Schistocephalus_solidus_Def_Loci-
33_LINE__CR1_LINE__L2:0.27920):0.04076,Sparganum_proliferum_Def_Loci-
15:0.28593):0.04960):0.00741):0.00692,((((Schistocephalus_solidus_Def_Loci-
3_LINE__RTE-BovB:0.30345,((Schistocephalus_solidus_Def_Loci-
28_LINE__CR1_LINE__Penelope:0.15414,Schistocephalus_solidus_Def_Loci-
30_LINE__CR1:0.00000):0.11301,Spirometra_erinaceieuropaei_Def_Loci-
8_LINE__CR1:0.27787):0.01693):0.11966,((Schistocephalus_solidus_Def_Loci-
10_LINE__CR1:0.01713,(Schistocephalus_solidus_Def_Loci-
14_LINE__CR1:0.00000,Schistocephalus_solidus_Def_Loci-
15_LINE__CR1:0.09824):0.02685):0.18894,(Spirometra_erinaceieuropaei_Def_Loci-
13_LTR__Gypsy:0.00000,Spirometra_erinaceieuropaei_Def_Loci-
18:0.24156):0.19150):0.04523):0.07632,(Sparganum_proliferum_Def_Loci-
8_LINE__Penelope:0.19387,Spirometra_erinaceieuropaei_Def_Loci-11_DNA__CMC-
EnSpm_LINE__RTE-
BovB_LTR__Gypsy:0.39594):0.06133):0.02252,(Schistocephalus_solidus_Def_Loci-
17_LINE__CR1_LINE__Penelope:0.22239,Schistocephalus_solidus_Def_Loci-
18_LINE__Penelope_LINE__RTE-
BovB:0.34548):0.12416):0.04610):0.00629,(((((Schistocephalus_solidus_Def_Loci-
2_LINE__RTE-BovB:0.24425,(Sparganum_proliferum_Def_Loci-
1_Low_complexity:0.05855,Spirometra_erinaceieuropaei_Def_Loci-
1:0.03776):0.30715):0.10419,(Schistocephalus_solidus_Def_Loci-
6_LINE__CR1:0.30959,(Spirometra_erinaceieuropaei_Def_Loci-14_LINE__RTE-
BovB:0.22681,Spirometra_erinaceieuropaei_Def_Loci-
16:0.11943):0.17859):0.03250):0.01739,((Schistocephalus_solidus_Def_Loci-
11:0.30537,Ces_cdhit_Clus-69_Spirometra_erinaceieuropaei_Def_Loci-
6_LINE__CR1:0.24208):0.04115,Sparganum_proliferum_Def_Loci-
10_LINE__Penelope:0.33577):0.04829):0.01184,(Schistocephalus_solidus_Def_Loci-
25_LINE__CR1:0.30696,(Spirometra_erinaceieuropaei_Def_Loci-
7_LINE__Penelope:0.22550,Spirometra_erinaceieuropaei_Def_Loci-
9:0.36745):0.19511):0.06394):0.01290,((Schistocephalus_solidus_Def_Loci-
4_LINE__CR1:0.36286,(Schistocephalus_solidus_Def_Loci-
24:0.30198,(Spirometra_erinaceieuropaei_Def_Loci-

3_LTR__Gypsy:0.18402,Taenia_solium_Def_Loci-3_DNA__TcMar-
Tc1_Low_complexity:0.48727):0.10263):0.06614):0.01825,(((Schistocephalus_solidus_Def_
Loci-19_DNA__MULE_LINE__L2_LTR__Gypsy:0.14455,Schistocephalus_solidus_Def_Loci-
29_LINE__CR1_LINE__L2_LTR__Gypsy:0.00000):0.05416,Schistocephalus_solidus_Def_Loci-
27_LINE__CR1_LINE__Rex-
Babar_LTR__Gypsy:0.00000):0.14679,Sparganum_proliferum_Def_Loci-
9_LTR__Gypsy:0.35445):0.09591):0.03169):0.00629);

B) Trematodes

(((((((((((((((((((((((((((Clonorchis_sinensis_Def_Loci-
1:0.03168,(((Clonorchis_sinensis_Def_Loci-13_LINE__RTE-
BovB:0.00641,Clonorchis_sinensis_Def_Loci-
59_LINE__CR1:0.06856):0.00748,((Clonorchis_sinensis_Def_Loci-
24:0.02806,Clonorchis_sinensis_Def_Loci-
56_LINE__CR1:0.02622):0.00516,(Clonorchis_sinensis_Def_Loci-
29:0.02130,(Clonorchis_sinensis_Def_Loci-63_LINE__RTE-
BovB:0.04534,Clonorchis_sinensis_Def_Loci-
66_LINE__CR1_LTR__Gypsy:0.04815):0.01748):0.01075):0.00775):0.00304,Clonorchis_sinens
is_Def_Loci-80_DNA__MULE-MuDR:0.05096):0.00075):0.00632,Clonorchis_sinensis_Def_Loci-
31_LTR__Gypsy:0.03777):0.01313,(Clonorchis_sinensis_Def_Loci-
21_LTR__Pao:0.03853,Clonorchis_sinensis_Def_Loci-41_DNA__MULE-
MuDR:0.05137):0.00539):0.00099,((((Clonorchis_sinensis_Def_Loci-
8:0.02857,(Clonorchis_sinensis_Def_Loci-40:0.08161,Clonorchis_sinensis_Def_Loci-
32:0.03792):0.01759):0.01668,Tre_cdhit_Clus-192_Clonorchis_sinensis_Def_Loci-
23:0.01860):0.00298,Clonorchis_sinensis_Def_Loci-
64:0.01359):0.00332,(Clonorchis_sinensis_Def_Loci-
11:0.02224,Clonorchis_sinensis_Def_Loci-
79:0.02795):0.00574):0.00661):0.00711,Clonorchis_sinensis_Def_Loci-
68_LINE__CR1:0.03990):0.00570,Clonorchis_sinensis_Def_Loci-58_DNA__MULE-
MuDR:0.02543):0.01258,(((Clonorchis_sinensis_Def_Loci-
9_LTR__Pao:0.04182,Clonorchis_sinensis_Def_Loci-50_DNA__MULE-MuDR_LINE__RTE-
BovB:0.19156):0.00713,(Clonorchis_sinensis_Def_Loci-
10_LINE__CR1:0.02121,Clonorchis_sinensis_Def_Loci-17_LINE__RTE-
BovB:0.05221):0.00901):0.01071,(Clonorchis_sinensis_Def_Loci-74_DNA__MULE-
MuDR:0.06867,Clonorchis_sinensis_Def_Loci-
88:0.07272):0.01419):0.00801):0.00813,Clonorchis_sinensis_Def_Loci-78_LINE__RTE-
BovB:0.05124):0.02699,Clonorchis_sinensis_Def_Loci-
69_LTR__Gypsy:0.12450):0.02554,((((((((((((Clonorchis_sinensis_Def_Loci-
19:0.04384,Opisthorchis_felineus_Def_Loci-
51:0.05831):0.03374,Opisthorchis_felineus_Def_Loci-
30:0.03948):0.00347,(Opisthorchis_felineus_Def_Loci-
24_LINE__CR1:0.05945,(Opisthorchis_felineus_Def_Loci-
86_LINE__CR1_LTR__Gypsy:0.03350,Opisthorchis_felineus_Def_Loci-
118_LINE__CR1:0.06276):0.06103):0.01660):0.01415,Tre_cdhit_Clus-
488_Opisthorchis_felineus_Def_Loci-58_DNA__MULE-
MuDR:0.03186):0.00849,Opisthorchis_felineus_Def_Loci-
3_LINE__CR1:0.04263):0.01700,(Opisthorchis_felineus_Def_Loci-
15_LINE__CR1:0.01950,Opisthorchis_felineus_Def_Loci-

102_LINE__CR1:0.03545):0.01289):0.01366,(Opisthorchis_felineus_Def_Loci-
54_LINE__CR1:0.00384,Opisthorchis_felineus_Def_Loci-119_LINE__CR1_LINE__RTE-
BovB:0.04499):0.01057):0.00726,Opisthorchis_felineus_Def_Loci-
85_LINE__CR1_tRNA:0.03893):0.00142,(Opisthorchis_felineus_Def_Loci-40_DNA__MULE-
MuDR:0.02522,Opisthorchis_felineus_Def_Loci-
113:0.02656):0.01169):0.00777,(Opisthorchis_felineus_Def_Loci-
32:0.06887,Opisthorchis_felineus_Def_Loci-
34_LINE__CR1:0.04056):0.00303):0.01362,Opisthorchis_felineus_Def_Loci-
130:0.06937):0.00810,(Opisthorchis_felineus_Def_Loci-116_LINE__L1-
DRE:0.06034,((Opisthorchis_felineus_Def_Loci-
107_LINE__CR1:0.07302,Opisthorchis_felineus_Def_Loci-
115_LINE__CR1:0.07325):0.04058,Opisthorchis_felineus_Def_Loci-126_DNA__MULE-
MuDR:0.08222):0.00408):0.02442):0.00874):0.00750,Opisthorchis_felineus_Def_Loci-
21:0.08341):0.02410,(Tre_cdhit_Clus-497_Opisthorchis_felineus_Def_Loci-
77:0.14750,Opisthorchis_felineus_Def_Loci-
132_LINE__CR1_LTR__Gypsy_LTR__Pao:0.10307):0.02474):0.01627,Clonorchis_sinensis_Def_L
oci-25:0.15913):0.01800,Opisthorchis_felineus_Def_Loci-117_DNA__MULE-
MuDR:0.20117):0.04340,(Clonorchis_sinensis_Def_Loci-
48_LTR__Pao:0.07593,(Clonorchis_sinensis_Def_Loci-
62_LTR__Pao:0.00000,Clonorchis_sinensis_Def_Loci-33_LINE__RTE-
BovB:0.09543):0.03091):0.12630):0.04368,Tre_cdhit_Clus-
213_Clonorchis_sinensis_Def_Loci-
71:0.22309):0.07160,((((((Clonorchis_sinensis_Def_Loci-6_LINE__RTE-
BovB:0.09842,Clonorchis_sinensis_Def_Loci-55_LINE__RTE-
BovB:0.11260):0.04852,((Clonorchis_sinensis_Def_Loci-
83_LINE__CR1:0.08500,Opisthorchis_felineus_Def_Loci-
76_LINE__CR1:0.05149):0.00489,(Opisthorchis_felineus_Def_Loci-
120_LTR__Gypsy:0.01847,Tre_cdhit_Clus-15_Opisthorchis_felineus_Def_Loci-
121_LTR__Gypsy:0.05463):0.03883):0.00785):0.01635,((((((((Opisthorchis_felineus_Def_
Loci-1_DNA__MULE-MuDR:0.03842,Opisthorchis_felineus_Def_Loci-
106:0.06217):0.00593,Opisthorchis_felineus_Def_Loci-
55_LINE__CR1:0.02031):0.01165,(Opisthorchis_felineus_Def_Loci-
36_LINE__CR1:0.07837,Opisthorchis_felineus_Def_Loci-
131:0.02538):0.02800):0.00479,(Opisthorchis_felineus_Def_Loci-23_LINE__I-
Jockey:0.10781,Opisthorchis_felineus_Def_Loci-
109:0.03773):0.01379):0.00520,Opisthorchis_felineus_Def_Loci-
142_LINE__CR1:0.04535):0.01981,Opisthorchis_felineus_Def_Loci-31_DNA__CMC-
EnSpm:0.07465):0.00791,(Opisthorchis_felineus_Def_Loci-
92_LTR__Pao:0.09101,Opisthorchis_felineus_Def_Loci-122_LINE__RTE-
BovB_LTR__Gypsy:0.03563):0.01268):0.00848,(Opisthorchis_felineus_Def_Loci-
71:0.05232,Opisthorchis_felineus_Def_Loci-
82:0.04879):0.02665):0.01107,(Opisthorchis_felineus_Def_Loci-
45_LTR__Pao:0.07227,Opisthorchis_felineus_Def_Loci-
26:0.07921):0.02312):0.00802):0.00810,(Opisthorchis_felineus_Def_Loci-
22_LINE__CR1:0.02099,Opisthorchis_felineus_Def_Loci-
125_LINE__CR1:0.02861):0.05793):0.02451,Opisthorchis_felineus_Def_Loci-
37:0.15583):0.07427,Opisthorchis_felineus_Def_Loci-
19:0.31945):0.03763,((Clonorchis_sinensis_Def_Loci-

12_LTR__Gypsy:0.09074,((Clonorchis_sinensis_Def_Loci-76_DNA__MULE-
MuDR_LINE__CR1:0.12811,Opisthorchis_felineus_Def_Loci-
101:0.12060):0.01816,(((Opisthorchis_felineus_Def_Loci-
13:0.05136,((Opisthorchis_felineus_Def_Loci-
14:0.03728,((Opisthorchis_felineus_Def_Loci-
20:0.08189,Opisthorchis_felineus_Def_Loci-
99:0.05383):0.01136,Opisthorchis_felineus_Def_Loci-
62:0.05702):0.01770):0.02940,((Opisthorchis_felineus_Def_Loci-60_DNA__CMC-
EnSpm:0.01575,Opisthorchis_felineus_Def_Loci-4_DNA__MULE-
MuDR:0.08281):0.02089,Opisthorchis_felineus_Def_Loci-
140:0.08806):0.01287):0.00537):0.00562,Opisthorchis_felineus_Def_Loci-
57:0.03567):0.00307,(Opisthorchis_felineus_Def_Loci-
72_LTR__Gypsy:0.05427,Opisthorchis_felineus_Def_Loci-
133_LINE__CR1:0.06169):0.03224):0.04385):0.02755):0.03611,Clonorchis_sinensis_Def_Loc
i-36_LINE__CR1_LINE__RTE-
BovB:0.20477):0.05113):0.09451):0.03298,((Opisthorchis_felineus_Def_Loci-
110_LINE__CR1:0.24528,Schistosoma_mansoni_Def_Loci-
143_LINE:0.04609):0.04126,(Opisthorchis_felineus_Def_Loci-
114:0.03509,Opisthorchis_felineus_Def_Loci-
89:0.05489):0.21936):0.05692):0.02964,((Fasciola_gigantica_Def_Loci-
27_LINE__CR1_LINE__RTE-BovB:0.25013,(Paragonimus_heterotremus_Def_Loci-
9:0.13232,Tre_cdhit_Clus-447_Schistosoma_mansoni_Def_Loci-136_LINE__CR1_LINE__RTE-
BovB:0.15549):0.08841):0.08035,((Fasciola_gigantica_Def_Loci-70_DNA__CMC-
EnSpm:0.23393,Schistosoma_japonicum_Def_Loci-
3:0.18562):0.11136,(Paragonimus_westermani_Def_Loci-
42:0.18351,Schistosoma_bovis_Def_Loci-
10:0.19563):0.07416):0.03379):0.01547):0.01274,((((((((Clonorchis_sinensis_Def_Loci-
4_LINE__CR1:0.15141,Trematoda_Schistosoma_mansoni_Smp_rajko_90nt_slRNA-
1.1.1:0.00000):0.04039,Trichobilharzia_regenti_Def_Loci-
1_LINE__Penelope:0.04459):0.05613,((((((Opisthorchis_felineus_Def_Loci-
111:0.18132,Schistosoma_haematobium_Def_Loci-4_LINE__RTE-
BovB:0.01856):0.01672,Schistosoma_bovis_Def_Loci-8_LINE__RTE-
BovB:0.00165):0.02427,Schistosoma_bovis_Def_Loci-
9:0.02326):0.02800,Trematoda_Schistosoma_mansoni_rajko.90nt.slRNA-
46.1.1:0.00000):0.01543,(((((Paragonimus_heterotremus_Def_Loci-
4:0.27164,Schistosoma_japonicum_Def_Loci-
4_LINE__CR1_LINE__Penelope:0.00000):0.13081,(Schistosoma_bovis_Def_Loci-3_LINE__RTE-
BovB:0.05384,Schistosoma_mansoni_Def_Loci-13_LTR__Gypsy:0.16820):0.01627):-
0.03943,Trematoda_Schistosoma_mansoni_rajko.90nt.slRNA-
39.1.1:0.00000):0.02124,((Tre_cdhit_Clus-145_Schistosoma_bovis_Def_Loci-
20:0.01520,((Tre_cdhit_Clus-373_Schistosoma_mansoni_Def_Loci-
24:0.00000,Schistosoma_mansoni_Def_Loci-35:0.12528):0.01983,Tre_cdhit_Clus-
117_Schistosoma_mansoni_Def_Loci-29:0.00000):0.03572):-
0.02768,Trematoda_Schistosoma_mansoni_rajko.90nt.slRNA-5.1.1:0.00000):0.02836):-
0.02021,Trematoda_Schistosoma_mansoni_rajko.90nt.slRNA-
31.1.1:0.00000):0.00912):0.00962,Schistosoma_mansoni_Def_Loci-
15:0.06222):0.01736,(((Paragonimus_westermani_Def_Loci-
9_LINE__CR1_LTR__Pao:0.12965,Trichobilharzia_regenti_Def_Loci-

36_LTR__Gypsy:0.10197):0.08451,Schistosoma_mansoni_Def_Loci-22_LINE__RTE-
BovB:0.09412):-0.00684,Trematoda_Schistosoma_mansoni_rajko.90nt.slRNA-
45.1.1:0.00000):0.02450):0.00697):0.00321,(Fasciola_hepatica_Def_Loci-
89_LINE__CR1_Low_complexity:0.27813,Schistosoma_mansoni_Def_Loci-23_LINE__RTE-
BovB:0.06425):0.07760):0.02226,(((((Paragonimus_heterotremus_Def_Loci-37_DNA__CMC-
EnSpm_LINE__L1-Tx1:0.13961,Trichobilharzia_regenti_Def_Loci-
33:0.00000):0.04282,Trichobilharzia_regenti_Def_Loci-
25:0.01534):0.02357,(Paragonimus_westermani_Def_Loci-36_DNA__CMC-
EnSpm_LINE__CR1:0.28739,Trichobilharzia_regenti_Def_Loci-
14:0.00131):0.02900):0.01504,(((Trichobilharzia_regenti_Def_Loci-
24_LINE__Penelope_LINE__RTE-BovB:0.06379,((Trichobilharzia_regenti_Def_Loci-
71:0.06529,Trichobilharzia_regenti_Def_Loci-
100_LINE__Penelope:0.06794):0.09675,Trichobilharzia_regenti_Def_Loci-79_LINE__Rex-
Babar:0.06785):0.01723):0.03547,Trichobilharzia_regenti_Def_Loci-
84_tRNA:0.01458):0.03490,Trichobilharzia_regenti_Def_Loci-
56_LINE_LINE__CR1:0.06391):0.05336):0.05689,(Paragonimus_westermani_Def_Loci-
45_LINE__RTE-BovB:0.16708,Schistosoma_bovis_Def_Loci-14_LINE__Penelope_LINE__RTE-
BovB:0.11360):0.11014):0.01600):0.00628,(((Paragonimus_heterotremus_Def_Loci-
12:0.21353,Tre_cdhit_Clus-137_Schistosoma_bovis_Def_Loci-6_LINE__Penelope_LINE__RTE-
BovB_Low_complexity:0.07023):0.13843,(Paragonimus_heterotremus_Def_Loci-
57:0.16250,(Schistosoma_bovis_Def_Loci-5_LINE__RTE-
BovB:0.00000,Schistosoma_haematobium_Def_Loci-3_LINE__RTE-
BovB:0.00994):0.12385):0.16690):0.03456,(Paragonimus_heterotremus_Def_Loci-
17_LINE__CR1:0.22824,Schistosoma_mansoni_Def_Loci-
140_LINE__CR1:0.14391):0.09704):0.02838):0.01642,(((((Fasciolopsis_buski_Def_Loci-
7_LINE__Rex-Babar:0.19750,Schistosoma_mansoni_Def_Loci-
6_LINE__CR1:0.07321):0.09441,((Paragonimus_heterotremus_Def_Loci-
46_LINE__CR1:0.15973,Schistosoma_haematobium_Def_Loci-
11:0.13973):0.04466,(Schistosoma_mansoni_Def_Loci-14_LINE__RTE-
BovB:0.17154,Schistosoma_mansoni_Def_Loci-144_LINE__RTE-
BovB:0.11851):0.08272):0.03240):0.01275,(Opisthorchis_felineus_Def_Loci-
127:0.26763,Schistosoma_mansoni_Def_Loci-
20:0.16392):0.06375):0.01400,(Schistosoma_japonicum_Def_Loci-5_LINE__RTE-
BovB:0.11610,(Schistosoma_mansoni_Def_Loci-12_LINE__RTE-
BovB:0.13915,Paragonimus_heterotremus_Def_Loci-13_LINE__RTE-
BovB:0.20566):0.05432):0.03572):0.02505,(((Opisthorchis_felineus_Def_Loci-
69_LINE__Penelope:0.24557,(Schistosoma_mansoni_Def_Loci-2_LINE__RTE-
BovB:0.39717,Trichobilharzia_regenti_Def_Loci-40_LINE__RTE-
BovB:0.01476):0.18537):0.03529,((Tre_cdhit_Clus-142_Schistosoma_bovis_Def_Loci-
15_LINE__Penelope:0.13599,Tre_cdhit_Clus-144_Schistosoma_bovis_Def_Loci-
17_LINE__Penelope_LINE__RTE-BovB:0.09555):0.00972,Schistosoma_japonicum_Def_Loci-
21_LINE__CR1_LTR__Gypsy:0.20034):0.06428):0.01472,(Schistosoma_haematobium_Def_Loci-
18_LINE__RTE-BovB:0.20371,Trematoda_Schistosoma_mansoni_rajko.90nt.slRNA-
3.1.1:0.03057):0.18653):0.04038):0.00709):0.01419,((((Fasciola_hepatica_Def_Loci-
134:0.17849,Schistosoma_mansoni_Def_Loci-
139_tRNA:0.09457):0.06843,(Schistosoma_mansoni_Def_Loci-4_LINE__RTE-
BovB:0.10202,Paragonimus_heterotremus_Def_Loci-
52_LINE__CR1:0.17727):0.07108):0.05106,((Paragonimus_westermani_Def_Loci-

18_DNA__TcMar-Tc1_LINE__Penelope:0.24177,Trichobilharzia_regenti_Def_Loci-
105_LINE__Penelope:0.23936):0.16788,(Paragonimus_westermani_Def_Loci-
35_LTR__Gypsy:0.03793,Paragonimus_westermani_Def_Loci-
48:0.08394):0.18809):0.03290):0.05250,((((((Paragonimus_heterotremus_Def_Loci-
1_LINE__CR1:0.03910,((Paragonimus_heterotremus_Def_Loci-
49_LINE__CR1:0.09298,(Paragonimus_westermani_Def_Loci-
47_LTR__Gypsy:0.00000,((Tre_cdhit_Clus-732_Paragonimus_westermani_Def_Loci-
54_DNA__CMC-EnSpm_LINE__CR1:0.03040,Paragonimus_westermani_Def_Loci-58_DNA__CMC-
EnSpm_LINE__CR1:0.04086):0.04205,Paragonimus_westermani_Def_Loci-
51_LTR__Gypsy:0.00967):0.02028):0.02951):0.02863,Paragonimus_heterotremus_Def_Loci-
30_LINE__CR1:0.03150):0.02151):0.00764,Paragonimus_heterotremus_Def_Loci-
25_DNA__MULE-
MuDR_LINE__CR1_Low_complexity:0.06154):0.04726,Paragonimus_heterotremus_Def_Loci-
7_LINE__CR1_LINE__RTE-BovB:0.17635):0.01264,Paragonimus_heterotremus_Def_Loci-
23_LINE__CR1:0.16144):0.05978,((((Paragonimus_heterotremus_Def_Loci-
11_LINE__CR1:0.10652,((Paragonimus_heterotremus_Def_Loci-
18_LINE__CR1:0.04630,Paragonimus_heterotremus_Def_Loci-
39_LINE__CR1:0.03818):0.03492,Paragonimus_heterotremus_Def_Loci-
24_LINE__Penelope:0.06303):0.01584):0.00434,Paragonimus_heterotremus_Def_Loci-
21_LINE__CR1:0.05085):0.03368,Paragonimus_heterotremus_Def_Loci-
32:0.13521):0.04040,(Paragonimus_heterotremus_Def_Loci-
56_LINE__CR1_LTR__Gypsy:0.18293,Paragonimus_westermani_Def_Loci-
10_LINE__CR1_LINE__RTE-
BovB:0.14642):0.05821):0.05035):0.08066,(Paragonimus_heterotremus_Def_Loci-
42_LINE__CR1_LINE__L1-Tx1:0.26454,Trichobilharzia_regenti_Def_Loci-
91_LINE__CR1:0.26723):0.06875):0.03260,((Paragonimus_heterotremus_Def_Loci-
6_LINE__RTE-BovB:0.15731,Schistosoma_mansoni_Def_Loci-1_LINE__Penelope_LINE__RTE-
BovB:0.13139):0.07554,(Paragonimus_heterotremus_Def_Loci-
8:0.23972,Trichobilharzia_regenti_Def_Loci-
123_LTR__Gypsy:0.19508):0.06495):0.05877):0.03874):0.00697):0.01806,(((((((((((Clono
rchis_sinensis_Def_Loci-22_DNA__CMC-EnSpm_DNA__MULE-
MuDR_LINE__CR1:0.09328,Opisthorchis_felineus_Def_Loci-49_LINE__CR1_LINE__RTE-
BovB:0.07568):0.01203,(((Clonorchis_sinensis_Def_Loci-70_LINE__CR1_LINE__RTE-
BovB:0.14882,Opisthorchis_felineus_Def_Loci-88_LINE__CR1_LINE__RTE-
BovB:0.15760):0.01939,Tre_cdhit_Clus-40_Opisthorchis_felineus_Def_Loci-
79_LINE__CR1:0.00000):0.04091,Opisthorchis_felineus_Def_Loci-
123_LINE__CR1:0.01545):0.03635):0.01384,Opisthorchis_felineus_Def_Loci-
135_LINE__CR1:0.16101):0.00000,Opisthorchis_felineus_Def_Loci-
10_LINE__CR1:0.10985):0.02195,(Opisthorchis_felineus_Def_Loci-
7_LINE__CR1:0.03114,Opisthorchis_felineus_Def_Loci-52_LINE__CR1_LINE__RTE-
BovB:0.15198):0.02017):0.01617,Clonorchis_sinensis_Def_Loci-49_DNA__MULE-
MuDR_LINE__CR1:0.19083):0.02555,((Opisthorchis_felineus_Def_Loci-
9_LINE__CR1:0.07060,Opisthorchis_felineus_Def_Loci-108_DNA__MULE-
MuDR_LINE__CR1:0.06635):0.01937,Opisthorchis_felineus_Def_Loci-
103_LINE__CR1_LTR__Gypsy:0.09574):0.02075):0.01856,((Opisthorchis_felineus_Def_Loci-
5_LINE__CR1:0.08216,Opisthorchis_felineus_Def_Loci-
53_LINE__CR1:0.07844):0.08533,Opisthorchis_felineus_Def_Loci-66_LINE__CR1_LINE__RTE-
BovB:0.12747):0.00622):0.00408,(Opisthorchis_felineus_Def_Loci-

17_LINE__CR1_LINE__RTE-BovB:0.13020,Opisthorchis_felineus_Def_Loci-
47_LINE__CR1:0.15951):0.04383):0.02416,Clonorchis_sinensis_Def_Loci-
67_LINE__CR1:0.11477):0.16546,(((Fasciola_gigantica_Def_Loci-
122_LINE:0.23570,((((Trichobilharzia_regenti_Def_Loci-
2:0.02284,Trichobilharzia_regenti_Def_Loci-
41:0.00302):0.01715,Trichobilharzia_regenti_Def_Loci-
69:0.01988):0.01508,Trichobilharzia_regenti_Def_Loci-
16:0.08199):0.02359,Trichobilharzia_regenti_Def_Loci-
66_LTR__Pao:0.00006):0.09097):0.09877,(Paragonimus_westermani_Def_Loci-
56_LINE__CR1:0.26394,Tre_cdhit_Clus-115_Schistosoma_bovis_Def_Loci-22_LINE__RTE-
BovB:0.18744):0.07054):0.01837,((Fasciola_hepatica_Def_Loci-
90_LINE__CR1:0.22739,Tre_cdhit_Clus-670_Schistosoma_mansoni_Def_Loci-
7_rRNA:0.16728):0.11481,(Paragonimus_westermani_Def_Loci-
5_tRNA:0.21825,Schistosoma_bovis_Def_Loci-7_LINE__RTE-
BovB:0.11231):0.11979):0.03210):0.00987):0.02372,(((Clonorchis_sinensis_Def_Loci-
51:0.22611,(Paragonimus_heterotremus_Def_Loci-
22:0.29075,Schistosoma_mansoni_Def_Loci-
16_LINE__Penelope:0.23830):0.01173):0.06537,((Paragonimus_westermani_Def_Loci-
37:0.22180,(((Trichobilharzia_regenti_Def_Loci-
3:0.25572,Trichobilharzia_regenti_Def_Loci-
5:0.12904):0.05493,(((Trichobilharzia_regenti_Def_Loci-
4:0.06718,(((((Trichobilharzia_regenti_Def_Loci-
9:0.04236,(Trichobilharzia_regenti_Def_Loci-
10:0.07424,((Trichobilharzia_regenti_Def_Loci-
97_LINE__CR1:0.03326,Trichobilharzia_regenti_Def_Loci-
117_LTR__Pao:0.02904):0.00720,Trichobilharzia_regenti_Def_Loci-
102_LTR__Pao:0.03832):0.02406):0.01176):0.00966,((Trichobilharzia_regenti_Def_Loci-
22_LTR__Pao:0.08172,(Trichobilharzia_regenti_Def_Loci-
98:0.07711,Trichobilharzia_regenti_Def_Loci-
115:0.04078):0.00696):0.01849,(Trichobilharzia_regenti_Def_Loci-
110_LINE__CR1:0.03624,Trichobilharzia_regenti_Def_Loci-
124_LINE__CR1:0.06315):0.00991):0.01598):0.00460,(Trichobilharzia_regenti_Def_Loci-
19:0.03149,Trichobilharzia_regenti_Def_Loci-
78:0.06152):0.04242):0.01124,(Trichobilharzia_regenti_Def_Loci-
48:0.02374,Trichobilharzia_regenti_Def_Loci-
83:0.01330):0.02568):0.00868,((((Trichobilharzia_regenti_Def_Loci-
11_LINE__CR1:0.04138,(Trichobilharzia_regenti_Def_Loci-
80:0.04691,Trichobilharzia_regenti_Def_Loci-
82_LINE_LINE__CR1_Low_complexity:0.00000):0.03041):0.02061,Trichobilharzia_regenti_De
f_Loci-13_LINE__CR1:0.03931):0.00932,Trichobilharzia_regenti_Def_Loci-
108:0.04589):0.02274,(((Trichobilharzia_regenti_Def_Loci-
15:0.03826,(Trichobilharzia_regenti_Def_Loci-
30_LTR__Pao:0.03981,Trichobilharzia_regenti_Def_Loci-
85_LINE__CR1:0.00000):0.03211):0.03372,((Trichobilharzia_regenti_Def_Loci-
32_LINE__RTE-BovB:0.03594,Trichobilharzia_regenti_Def_Loci-
119:0.05470):0.00657,Trichobilharzia_regenti_Def_Loci-
95:0.05743):0.00074):0.00850,(((((((Trichobilharzia_regenti_Def_Loci-
20_LTR__Pao:0.01270,(((Trichobilharzia_regenti_Def_Loci-

23_LTR__Pao:0.01374,(Trichobilharzia_regenti_Def_Loci-
39_LINE__CR1:0.05891,((Trichobilharzia_regenti_Def_Loci-
47_LINE__Penelope:0.12792,Trichobilharzia_regenti_Def_Loci-
50_LTR__Gypsy_LTR__Pao:0.06329):0.12695,Trichobilharzia_regenti_Def_Loci-
81:0.00000):0.07926):0.05364):0.01312,Trichobilharzia_regenti_Def_Loci-
99:0.04671):0.02014,Trichobilharzia_regenti_Def_Loci-
93_LTR__Pao:0.04657):0.01876):0.01390,((Trichobilharzia_regenti_Def_Loci-
42_LINE__RTE-BovB:0.03462,Trichobilharzia_regenti_Def_Loci-
49:0.05717):0.02391,Trichobilharzia_regenti_Def_Loci-
70:0.00028):0.01967):0.00949,Trichobilharzia_regenti_Def_Loci-54_LINE__RTE-
BovB:0.02001):0.00904,Trichobilharzia_regenti_Def_Loci-64_LINE_LINE__Rex-
Babar_LTR__Pao:0.01297):0.01867,((Trichobilharzia_regenti_Def_Loci-
58:0.03771,Trichobilharzia_regenti_Def_Loci-
86_LTR__Gypsy_LTR__Pao:0.02000):0.00613,Trichobilharzia_regenti_Def_Loci-
113:0.09560):0.01318):0.00700,(Tre_cdhit_Clus-387_Trichobilharzia_regenti_Def_Loci-
21_LINE__CR1:0.00825,Trichobilharzia_regenti_Def_Loci-
101:0.03352):0.02647):0.00161,Trichobilharzia_regenti_Def_Loci-
31_LINE__CR1:0.02087):0.01935):0.00229):0.01009):0.00673):0.03046,(Trichobilharzia_re
genti_Def_Loci-6_LINE__CR1:0.07284,(Trichobilharzia_regenti_Def_Loci-
7_LTR__Gypsy:0.07901,Trichobilharzia_regenti_Def_Loci-
116:0.06006):0.04231):0.01518):0.00865,((Trichobilharzia_regenti_Def_Loci-
12:0.06691,Trichobilharzia_regenti_Def_Loci-120_LINE__RTE-
BovB_LTR__Pao:0.04878):0.02863,Trichobilharzia_regenti_Def_Loci-
103:0.09339):0.04131):0.02893):0.01377,(Trichobilharzia_regenti_Def_Loci-
28_LINE__CR1:0.12613,Trichobilharzia_regenti_Def_Loci-
106:0.18090):0.07826):0.09424):0.09778,Paragonimus_heterotremus_Def_Loci-
43_LINE__CR1:0.27946):0.05763):0.04227,((Opisthorchis_felineus_Def_Loci-
8:0.29581,(Opisthorchis_felineus_Def_Loci-95_LINE__CR1_LINE__RTE-
BovB:0.22528,Trichobilharzia_regenti_Def_Loci-89_LINE__RTE-
BovB:0.22743):0.14548):0.04188,((Trichobilharzia_regenti_Def_Loci-
8:0.12128,Paragonimus_westermani_Def_Loci-
43_LINE__Penelope_LTR__Gypsy:0.05795):0.15101,Paragonimus_westermani_Def_Loci-
28:0.20060):0.07494):0.02348):0.00885):0.00405):0.00576):0.00735,(((Clonorchis_sinens
is_Def_Loci-57:0.23915,Fasciola_gigantica_Def_Loci-
92_LINE__CR1_LTR__Pao:0.28203):0.13887,(Tre_cdhit_Clus-
125_Fasciola_gigantica_Def_Loci-
99_LTR__Gypsy:0.37657,Paragonimus_heterotremus_Def_Loci-
19:0.21818):0.08902):0.03137,((Opisthorchis_felineus_Def_Loci-
27:0.23488,Opisthorchis_felineus_Def_Loci-
139:0.27765):0.06412,(Paragonimus_heterotremus_Def_Loci-
31:0.14665,Trichobilharzia_regenti_Def_Loci-
55:0.07203):0.12960):0.02384):0.04289):0.00632,((((((Clonorchis_sinensis_Def_Loci-
2_LINE__CR1:0.24089,Paragonimus_westermani_Def_Loci-
50_LINE:0.20589):0.13646,((((((((Fasciola_gigantica_Def_Loci-23_LINE__CR1_LINE__RTE-
BovB:0.06241,Fasciola_hepatica_Def_Loci-
103_LINE__CR1_LTR__Pao:0.17584):0.07947,Fasciola_hepatica_Def_Loci-
81_LINE__CR1:0.10903):0.02573,Fasciola_gigantica_Def_Loci-
74_LINE__CR1_LTR__Gypsy:0.07263):0.04650,Fasciola_hepatica_Def_Loci-

22_LINE__CR1:0.04479):0.02144,Fasciola_hepatica_Def_Loci-
97_LINE__CR1:0.08526):0.03958,Fasciola_hepatica_Def_Loci-
73_LINE__CR1:0.11048):0.05147,Fasciola_hepatica_Def_Loci-101_LINE__CR1_LINE__RTE-
BovB:0.10159):0.14049,Fasciola_hepatica_Def_Loci-
65_LINE__CR1:0.33568):0.03121):0.07198,((Tre_cdhit_Clus-
724_Paragonimus_westermani_Def_Loci-20:0.28247,Paragonimus_westermani_Def_Loci-
40_LINE__RTE-BovB:0.28713):0.09096,((Trichobilharzia_regenti_Def_Loci-
57_LINE__Penelope:0.07207,Trichobilharzia_regenti_Def_Loci-
60:0.08857):0.04508,Paragonimus_westermani_Def_Loci-
19_LTR__Gypsy:0.25889):0.13481):0.02942):0.01018,(((Fasciola_hepatica_Def_Loci-
79:0.29781,Tre_cdhit_Clus-113_Schistosoma_bovis_Def_Loci-
1:0.13918):0.06019,((Fasciola_hepatica_Def_Loci-
74_LTR__Gypsy:0.28060,Schistosoma_japonicum_Def_Loci-6_LINE__RTE-
BovB:0.06655):0.09886,(Trichobilharzia_regenti_Def_Loci-
67_LINE__Penelope:0.08920,Paragonimus_westermani_Def_Loci-
52:0.30927):0.07930):0.01584):0.01012,(Fasciola_hepatica_Def_Loci-
133_LINE__CR1:0.22600,Trichobilharzia_regenti_Def_Loci-
35_LTR__Gypsy:0.13576):0.07250):0.04967):0.00633,((((Clonorchis_sinensis_Def_Loci-
27_LINE__RTE-BovB:0.26965,Trichobilharzia_regenti_Def_Loci-
38:0.07343):0.09249,(Fasciola_hepatica_Def_Loci-
80_LINE__CR1:0.27531,Paragonimus_heterotremus_Def_Loci-48_LINE__RTE-
BovB:0.27821):0.02901):0.05756,((Clonorchis_sinensis_Def_Loci-
75:0.33495,Opisthorchis_felineus_Def_Loci-112_LINE__RTE-
BovB:0.19757):0.05369,Schistosoma_haematobium_Def_Loci-
13:0.18151):0.08155):0.00995,(((Clonorchis_sinensis_Def_Loci-
61:0.28353,(Opisthorchis_felineus_Def_Loci-81:0.21922,Schistosoma_bovis_Def_Loci-
24_LINE__CR1_LINE__Penelope_LINE__RTE-
BovB_Low_complexity:0.00495):0.03511):0.02854,Clonorchis_sinensis_Def_Loci-
81:0.21332):0.04661,((Fasciola_gigantica_Def_Loci-16_LINE__Rex-
Babar:0.17350,Trichobilharzia_regenti_Def_Loci-
17_LTR__Gypsy:0.05188):0.12869,(Paragonimus_heterotremus_Def_Loci-
14_LINE__Penelope:0.37226,Paragonimus_westermani_Def_Loci-
55:0.26536):0.07471):0.03283):0.03700):0.01546):0.00559,(((((Clonorchis_sinensis_Def_
Loci-7_LINE__RTE-BovB_LTR__Gypsy:0.17202,(Paragonimus_heterotremus_Def_Loci-
44_LINE__RTE-BovB:0.18131,Trichobilharzia_regenti_Def_Loci-
44_LINE__CR1:0.09937):0.08707):0.05244,(((Paragonimus_heterotremus_Def_Loci-
2_DNA__MULE-MuDR_LINE__CR1:0.17937,Paragonimus_heterotremus_Def_Loci-
16_LINE__CR1:0.05976):0.07686,Paragonimus_heterotremus_Def_Loci-
28_LINE__CR1:0.13838):0.07538,Paragonimus_heterotremus_Def_Loci-3_DNA__MULE-
MuDR_LINE__CR1:0.21744):0.13054):0.02828,(((((((Opisthorchis_felineus_Def_Loci-
12_LINE__CR1:0.09103,Opisthorchis_felineus_Def_Loci-
48_LTR__Gypsy:0.09791):0.01669,Opisthorchis_felineus_Def_Loci-
61_LINE__CR1:0.04997):0.02519,Opisthorchis_felineus_Def_Loci-70_DNA__MULE-
MuDR_LINE__CR1:0.04993):0.02642,Opisthorchis_felineus_Def_Loci-
97:0.06794):0.02583,(Paragonimus_heterotremus_Def_Loci-
41_LINE__CR1:0.08528,Paragonimus_heterotremus_Def_Loci-45_LINE__CR1_LINE__L1-
Tx1:0.04245):0.20202):0.04615,(Paragonimus_heterotremus_Def_Loci-
26_LTR__Gypsy:0.24139,Paragonimus_heterotremus_Def_Loci-

47_LINE__CR1:0.18540):0.11288):0.00791,(((Paragonimus_heterotremus_Def_Loci-
35_LINE__CR1:0.09494,Paragonimus_heterotremus_Def_Loci-
40_LINE__CR1:0.05467):0.01210,(Paragonimus_heterotremus_Def_Loci-
38_LINE__CR1:0.01162,Paragonimus_heterotremus_Def_Loci-
51_LINE__CR1:0.09308):0.02014):0.20196,Paragonimus_westermani_Def_Loci-
46_LTR__Gypsy:0.20522):0.06434):0.05338):0.01610,((Clonorchis_sinensis_Def_Loci-
28:0.23125,Paragonimus_heterotremus_Def_Loci-
20:0.23166):0.10364,((Opisthorchis_felineus_Def_Loci-50_DNA__MULE-
MuDR_LINE__CR1:0.23300,Schistosoma_haematobium_Def_Loci-
1_LINE__Penelope_LTR__Gypsy:0.10527):0.08739,((Paragonimus_heterotremus_Def_Loci-
10_LINE__CR1_LINE__RTE-BovB:0.10454,Paragonimus_heterotremus_Def_Loci-
34_LINE__CR1:0.14064):0.05368,(Paragonimus_heterotremus_Def_Loci-
36_LINE__CR1:0.04825,Paragonimus_heterotremus_Def_Loci-
54:0.04982):0.10191):0.17177):0.04722):0.00717):0.00940,((((Clonorchis_sinensis_Def_L
oci-52:0.30124,Opisthorchis_felineus_Def_Loci-128_LINE__RTE-
BovB:0.29023):0.02638,(Opisthorchis_felineus_Def_Loci-
93_LINE__CR1:0.25917,Schistosoma_haematobium_Def_Loci-
12_LINE__Penelope_LTR__Gypsy:0.13986):0.13071):0.03545,(Opisthorchis_felineus_Def_Loc
i-29_LINE__CR1:0.20276,Paragonimus_westermani_Def_Loci-61_LINE__CR1_LINE__RTE-
BovB:0.28953):0.09396):0.02900,((Clonorchis_sinensis_Def_Loci-
15:0.24826,Tre_cdhit_Clus-445_Schistosoma_japonicum_Def_Loci-
7:0.05700):0.11264,(Fasciola_gigantica_Def_Loci-107_LINE__RTE-
BovB_LTR__Gypsy:0.26684,(Fasciola_hepatica_Def_Loci-
76_LINE__Penelope_LTR__Gypsy:0.19757,Fasciola_hepatica_Def_Loci-
14_LINE__CR1_LINE__Rex-
Babar_LTR__Gypsy:0.28110):0.05922):0.05707):0.06733):0.00619):0.01064):0.00289):0.004
26,(((((((Clonorchis_sinensis_Def_Loci-16:0.32806,Clonorchis_sinensis_Def_Loci-
73_LINE__CR1_LTR__Pao:0.22898):0.08334,(Fasciola_gigantica_Def_Loci-
66:0.21144,Opisthorchis_felineus_Def_Loci-
137_LINE__CR1:0.32322):0.05725):0.04798,(Fasciola_hepatica_Def_Loci-
122_LTR__Pao:0.34392,(Fasciolopsis_buski_Def_Loci-
5_LINE__CR1_LTR__Pao:0.28079,(Paragonimus_westermani_Def_Loci-
44:0.30810,((Tre_cdhit_Clus-116_Schistosoma_haematobium_Def_Loci-
6_LINE__CR1_LTR__Gypsy:0.01996,(Schistosoma_mansoni_Def_Loci-3_LINE__CR1_LINE__RTE-
BovB:0.11092,Schistosoma_mansoni_Def_Loci-
21_LINE__CR1_LTR__Gypsy:0.05085):0.02885):0.03768,Schistosoma_mansoni_Def_Loci-
5_LINE__CR1:0.04048):0.08546):0.07686):0.01188):0.05441):0.00921,(((((Fasciola_gigant
ica_Def_Loci-20_LINE_LINE__CR1_LTR__ERVK:0.12313,Fasciola_gigantica_Def_Loci-
67_LTR__Pao:0.34838):0.04163,(((Fasciola_gigantica_Def_Loci-
60_LINE__CR1_LTR__Pao:0.27668,Fasciola_hepatica_Def_Loci-
67:0.18414):0.05905,Fasciola_gigantica_Def_Loci-
112:0.05978):0.03251,(((((Fasciola_gigantica_Def_Loci-
89_LINE__CR1_LTR__Pao:0.06535,Tre_cdhit_Clus-288_Fasciola_hepatica_Def_Loci-
17:0.01596):0.01247,Fasciola_hepatica_Def_Loci-
18:0.02376):0.00237,Fasciola_gigantica_Def_Loci-
95:0.00000):0.03672,((Fasciola_hepatica_Def_Loci-
4_LINE__Penelope:0.17790,Fasciola_hepatica_Def_Loci-59_LINE__CR1:0.05770):-
0.02647,Trematoda_Fasciola_hepatica_U10296.1_Fas.SL:0.00000):0.00953):0.00995,Tre_cdh

it_Clus-265_Fasciola_gigantica_Def_Loci-
110:0.07281):0.05629):0.02581):0.02145,Fasciola_gigantica_Def_Loci-
94_LINE__CR1:0.36836):0.03423,(((Fasciola_gigantica_Def_Loci-127_DNA__CMC-
EnSpm:0.06506,Fasciola_hepatica_Def_Loci-
62_LINE__CR1_LTR__Pao:0.03644):0.02595,Fasciola_hepatica_Def_Loci-
1_LINE__CR1:0.20223):0.12018,(Fasciola_hepatica_Def_Loci-
42_LINE__CR1_LTR__Gypsy_LTR__Pao:0.05924,Tre_cdhit_Clus-
654_Fasciola_hepatica_Def_Loci-
44_LINE__CR1:0.13680):0.27249):0.07198):0.01914,((((Fasciola_gigantica_Def_Loci-
24_LINE__CR1:0.27233,(Opisthorchis_felineus_Def_Loci-
75_LTR__Gypsy:0.12521,Opisthorchis_felineus_Def_Loci-
94_LTR__Gypsy:0.06089):0.21240):0.03279,Fasciola_gigantica_Def_Loci-
113:0.12935):0.03969,(Fasciola_gigantica_Def_Loci-
111:0.17169,Fasciolopsis_buski_Def_Loci-
10_LINE__CR1:0.30297):0.01847):0.03636,(Fasciola_gigantica_Def_Loci-
49_LTR__Pao:0.19926,((Fasciola_gigantica_Def_Loci-
64:0.04623,Fasciola_hepatica_Def_Loci-11:0.08321):0.10121,Fasciola_hepatica_Def_Loci-
40_LINE__CR1:0.05298):0.15553):0.11433):0.02075):0.01963):0.00977,((((((((((((((((Fa
sciola_gigantica_Def_Loci-1_DNA__MULE-
MuDR_LINE__CR1_LTR__Gypsy:0.02699,Tre_cdhit_Clus-227_Fasciola_gigantica_Def_Loci-
8_LINE__CR1_LTR__Pao:0.07108):0.02984,Fasciola_hepatica_Def_Loci-
124:0.06835):0.00458,((Fasciola_gigantica_Def_Loci-65_DNA__CMC-
EnSpm_LINE__CR1_LTR__Pao:0.02672,(((Fasciola_gigantica_Def_Loci-
82_LINE__CR1_LTR__Gypsy:0.10949,Fasciola_gigantica_Def_Loci-
50_LINE__CR1:0.01448):0.04727,Fasciola_hepatica_Def_Loci-
15_LINE__CR1:0.18440):0.02467,Fasciola_gigantica_Def_Loci-
121_LINE__CR1:0.01545):0.00513):0.00450,Fasciola_hepatica_Def_Loci-
75_LTR__Pao:0.01695):0.00892):0.00550,(Fasciola_hepatica_Def_Loci-
58:0.06180,Fasciola_hepatica_Def_Loci-
111_LINE__CR1:0.00882):0.00755):0.00839,Fasciola_gigantica_Def_Loci-
103_LINE__CR1_LTR__Pao:0.05063):0.00986,Tre_cdhit_Clus-
89_Fasciola_gigantica_Def_Loci-
22_LINE__CR1_LTR__Pao:0.07559):0.01358,((Fasciola_gigantica_Def_Loci-
17_LINE__CR1:0.05576,(Fasciola_gigantica_Def_Loci-
33_LINE__CR1:0.04125,Fasciola_gigantica_Def_Loci-
118_LINE__CR1:0.02745):0.00763):0.00885,Fasciola_gigantica_Def_Loci-
125_LINE_LINE__CR1:0.07601):0.00427):0.00740,Fasciola_gigantica_Def_Loci-
56_LINE__CR1:0.13342):0.01522,(Fasciola_gigantica_Def_Loci-
4_LINE__CR1:0.07430,Fasciola_gigantica_Def_Loci-
58_LINE__CR1_LTR__Gypsy:0.08214):0.00175):0.01238,Fasciola_hepatica_Def_Loci-
77_LINE__CR1:0.09092):0.02014,Fasciola_hepatica_Def_Loci-
47_LINE__CR1:0.18588):0.00437,((Tre_cdhit_Clus-642_Fasciola_gigantica_Def_Loci-
3_LINE__CR1_LINE__RTE-BovB:0.05661,Fasciola_hepatica_Def_Loci-91_LINE__CR1_LINE__RTE-
BovB:0.04150):0.07774,(Fasciola_gigantica_Def_Loci-
37_LINE__CR1:0.11824,Fasciola_hepatica_Def_Loci-
38_DNA_LINE__CR1:0.06177):0.03504):0.02187):0.05622,Fasciola_gigantica_Def_Loci-
75_LINE__CR1_LINE__Penelope_LTR__Pao:0.24923):0.07860,(Fasciola_hepatica_Def_Loci-
39_LINE__CR1_LTR__Pao:0.21473,Fasciolopsis_buski_Def_Loci-

3_LTR__Pao:0.28817):0.05496):0.08763,(Fasciola_hepatica_Def_Loci-
105_LINE__CR1:0.28062,Schistosoma_mansoni_Def_Loci-
138_LINE__Penelope_Low_complexity:0.13460):0.07543):0.02585,((((Fasciola_gigantica_De
f_Loci-5_LTR__Pao:0.04437,Fasciola_hepatica_Def_Loci-
78_LTR__Gypsy:0.02162):0.09910,Fasciolopsis_buski_Def_Loci-
1_LINE__CR1:0.13358):0.11413,Fasciola_gigantica_Def_Loci-
38_LINE__Penelope:0.23042):0.01759,(Fasciola_gigantica_Def_Loci-
102:0.06090,Fasciola_hepatica_Def_Loci-
45:0.06868):0.16678):0.06085):0.03030,(((Fasciola_gigantica_Def_Loci-
12_LINE__CR1:0.29189,Opisthorchis_felineus_Def_Loci-
67:0.27022):0.05387,((Fasciola_gigantica_Def_Loci-
97:0.23972,Fasciolopsis_buski_Def_Loci-
9_LINE__CR1:0.20641):0.11122,(Fasciola_gigantica_Def_Loci-
134:0.27532,Fasciola_hepatica_Def_Loci-
50_LINE__CR1:0.30543):0.03456):0.01855):0.01405,((((((Fasciola_gigantica_Def_Loci-
25_LINE__CR1_LTR__Gypsy:0.12144,Fasciola_hepatica_Def_Loci-
31_LTR__Pao:0.07261):0.02129,Fasciola_hepatica_Def_Loci-
84_LINE__CR1_LINE__Penelope:0.10447):0.10691,Fasciola_hepatica_Def_Loci-
6_LINE__CR1_LTR__Pao:0.27627):0.06380,Fasciola_hepatica_Def_Loci-
55_LINE_LINE__CR1:0.25409):0.01295,(Fasciola_hepatica_Def_Loci-
107_LTR__Gypsy:0.19211,Trichobilharzia_regenti_Def_Loci-53_LINE__Penelope_LINE__RTE-
BovB:0.07286):0.08919):0.02975,(Fasciola_gigantica_Def_Loci-
124_LINE__Penelope_LINE__Rex-Babar:0.22182,(Fasciola_hepatica_Def_Loci-
23_LTR__Pao:0.10326,Fasciola_hepatica_Def_Loci-
32_LINE__CR1:0.01791):0.24595):0.10319):0.03109):0.00895):0.01245):0.00582,(((Clonorc
his_sinensis_Def_Loci-47_tRNA:0.31551,(Clonorchis_sinensis_Def_Loci-
39_LTR__Pao:0.30730,Trichobilharzia_regenti_Def_Loci-26_LINE__RTE-
BovB:0.25964):0.09286):0.05789,(((Fasciola_gigantica_Def_Loci-
6:0.22141,Schistosoma_japonicum_Def_Loci-2_LINE__RTE-
BovB:0.13684):0.05195,(Fasciola_gigantica_Def_Loci-98_LINE__RTE-
BovB_LTR__Gypsy_LTR__Pao:0.21622,Fasciola_hepatica_Def_Loci-
28_LINE__CR1:0.28075):0.03890):0.05311,(((((((Fasciola_gigantica_Def_Loci-
9_LINE__CR1:0.07261,(Fasciola_hepatica_Def_Loci-
13_LINE__CR1:0.04141,(Fasciola_hepatica_Def_Loci-
26_LINE__CR1:0.05470,Fasciola_hepatica_Def_Loci-
69_LINE__CR1:0.08573):0.00868):0.02743):0.00864,((Fasciola_gigantica_Def_Loci-
48_LINE__CR1:0.11346,Fasciola_gigantica_Def_Loci-
84:0.00254):0.04458,Fasciola_hepatica_Def_Loci-
2_LINE__CR1:0.03130):0.01991):0.03204,Fasciola_gigantica_Def_Loci-
40:0.06619):0.02561,Fasciola_gigantica_Def_Loci-
72_LTR__Gypsy:0.13497):0.06538,(((Fasciola_gigantica_Def_Loci-
46_LINE__CR1:0.00000,Fasciola_gigantica_Def_Loci-
73_LINE__CR1:0.30197):0.05375,Fasciola_hepatica_Def_Loci-
82_LINE__CR1:0.02802):0.03213,Fasciola_hepatica_Def_Loci-
68:0.08438):0.13469):0.11644,Paragonimus_westermani_Def_Loci-
31_LTR__Pao:0.29923):0.01701,(Fasciola_hepatica_Def_Loci-30_LINE__CR1_LINE__RTE-
BovB:0.22246,(Fasciola_hepatica_Def_Loci-
117_LINE__CR1_LTR__Pao:0.19565,Schistosoma_japonicum_Def_Loci-

1_LTR__Gypsy:0.05025):0.11495):0.06915):0.02861):0.03019):0.01430,(((((((Fasciola_gi
gantica_Def_Loci-2_LINE__CR1:0.01281,Fasciola_hepatica_Def_Loci-
53:0.04777):0.01239,Fasciola_hepatica_Def_Loci-
52:0.00733):0.15996,Fasciola_gigantica_Def_Loci-
93_LTR__Gypsy:0.23260):0.07261,Fasciola_gigantica_Def_Loci-
132_LTR__Pao:0.27306):0.06677,((Fasciola_gigantica_Def_Loci-
39_LINE__CR1_LTR__Pao:0.07647,Fasciola_gigantica_Def_Loci-
123_LINE__CR1_LTR__Pao:0.09051):0.03813,(Fasciola_gigantica_Def_Loci-
7_LINE__CR1:0.06766,Fasciola_hepatica_Def_Loci-
21_LINE__CR1_LTR__Gypsy:0.06923):0.04104):0.15820):0.02001,(((Fasciola_gigantica_Def_
Loci-43_LINE__CR1:0.03903,Fasciola_hepatica_Def_Loci-
120_LINE__CR1_LINE__L2:0.07490):0.13528,Fasciola_hepatica_Def_Loci-
54_LINE__CR1:0.18616):0.06522,Fasciola_hepatica_Def_Loci-
8:0.19406):0.05343):0.04412,((((Fasciola_gigantica_Def_Loci-
15_LTR__Gypsy:0.08996,Fasciola_hepatica_Def_Loci-
132_LTR__Gypsy:0.07353):0.24052,(Fasciola_gigantica_Def_Loci-
57_LINE__CR1:0.17534,Fasciola_hepatica_Def_Loci-
121_LINE__CR1:0.27668):0.03694):0.01692,Fasciola_gigantica_Def_Loci-
62:0.22749):0.05795,((Fasciola_gigantica_Def_Loci-
31_LINE__CR1_LTR__Gypsy:0.14962,Fasciola_gigantica_Def_Loci-
32_LINE__CR1_LTR__Gypsy:0.09193):0.04923,((((((((((Tre_cdhit_Clus-
238_Fasciola_gigantica_Def_Loci-
35_LTR__Gypsy:0.01119,((((((Fasciola_gigantica_Def_Loci-68_LINE__CR1_LINE__RTE-
BovB_LTR__Gypsy:0.00000,Fasciola_gigantica_Def_Loci-76_LINE__Rex-
Babar_LTR__Gypsy:0.21043):0.05149,Fasciola_hepatica_Def_Loci-
51_LTR__Gypsy:0.03238):0.02003,Fasciola_hepatica_Def_Loci-72_DNA__CMC-
EnSpm_LTR__Gypsy_LTR__Pao:0.09130):0.02115,Fasciola_hepatica_Def_Loci-
116_LTR__Gypsy_LTR__Pao:0.00000):0.00743,Fasciola_hepatica_Def_Loci-
85_LTR__Gypsy:0.01402):0.00402,Fasciola_hepatica_Def_Loci-
29:0.02384):0.00826,Fasciola_hepatica_Def_Loci-
57_LINE:0.02564):0.00661):0.00453,Fasciola_hepatica_Def_Loci-
125_LINE__CR1:0.01122):0.00713,Tre_cdhit_Clus-305_Fasciola_hepatica_Def_Loci-
49_LTR__Gypsy:0.02575):0.00774,Fasciola_gigantica_Def_Loci-53_DNA__MULE-
MuDR_LTR__Gypsy:0.02295):0.00712,((Fasciola_gigantica_Def_Loci-
69_LINE__CR1_LTR__Gypsy:0.09040,Fasciola_gigantica_Def_Loci-71_DNA__CMC-
EnSpm_LINE__CR1_LTR__Gypsy:0.00748):0.02008,Fasciola_gigantica_Def_Loci-
120_LTR__Gypsy:0.02774):0.00135):0.00327,((Fasciola_gigantica_Def_Loci-77_LINE__Rex-
Babar_LTR__Gypsy:0.02938,Fasciola_hepatica_Def_Loci-
129:0.00098):0.03415,Fasciola_hepatica_Def_Loci-27_DNA__CMC-
EnSpm_LINE__CR1_LTR__Gypsy:0.03673):0.00723):0.00266,Tre_cdhit_Clus-
515_Fasciola_gigantica_Def_Loci-
87_LTR__Gypsy:0.01723):0.00773,(Fasciola_gigantica_Def_Loci-63_DNA__CMC-
EnSpm_LTR__Gypsy:0.04396,(Tre_cdhit_Clus-57_Fasciola_gigantica_Def_Loci-
29_LTR__Gypsy:0.00539,Fasciola_hepatica_Def_Loci-
115:0.01562):0.02498):0.00906):0.00452,((Fasciola_gigantica_Def_Loci-
14_LTR__Gypsy_LTR__Pao:0.09299,Tre_cdhit_Clus-329_Fasciola_hepatica_Def_Loci-
94_LTR__Gypsy:0.06487):0.02979,Fasciola_hepatica_Def_Loci-
16_LINE__CR1:0.05827):0.01326):0.00827,Fasciola_hepatica_Def_Loci-

25:0.08856):0.02386,Fasciola_hepatica_Def_Loci-
86:0.12529):0.07044):0.12605):0.02398,((Fasciola_gigantica_Def_Loci-
19:0.19777,Fasciola_gigantica_Def_Loci-
131:0.23512):0.06752,(Fasciola_gigantica_Def_Loci-
109:0.16992,Trichobilharzia_regenti_Def_Loci-
87_LINE__CR1:0.05569):0.13269):0.03830):0.01844):0.00142,(((Fasciola_gigantica_Def_Lo
ci-11_LINE:0.02669,Fasciola_hepatica_Def_Loci-
123_LINE__CR1:0.06655):0.16719,Fasciola_hepatica_Def_Loci-
70:0.25480):0.12769,((Fasciola_gigantica_Def_Loci-78_LINE__RTE-
BovB:0.28457,Tre_cdhit_Clus-338_Fasciola_hepatica_Def_Loci-
108_LINE__CR1:0.31889):0.02623,(Fasciolopsis_buski_Def_Loci-
4:0.26880,Trichobilharzia_regenti_Def_Loci-
76:0.06757):0.07346):0.06206):0.02435):0.01308):0.01058):0.00648,((((Clonorchis_sinen
sis_Def_Loci-34:0.26107,((((Fasciola_gigantica_Def_Loci-
10_LTR__Pao:0.17172,Fasciola_hepatica_Def_Loci-
88_LINE__CR1:0.09119):0.03301,(((((((Tre_cdhit_Clus-688_Fasciola_gigantica_Def_Loci-
52_LINE__CR1:0.07509,Fasciola_gigantica_Def_Loci-
55_LINE__CR1_LTR__Pao:0.04437):0.02763,Fasciola_hepatica_Def_Loci-
110_LINE__CR1_LTR__Pao:0.05608):0.04866,Fasciola_hepatica_Def_Loci-
93_LINE__CR1:0.02653):0.04333,Fasciola_hepatica_Def_Loci-
5_LINE__CR1:0.16673):0.02036,Fasciola_hepatica_Def_Loci-
102_LINE__CR1:0.08374):0.01969,Fasciola_hepatica_Def_Loci-
87_LINE__CR1:0.06444):0.01133,Fasciola_hepatica_Def_Loci-
118_LTR__Pao:0.08091):0.00964):0.01071,Fasciola_gigantica_Def_Loci-
42_LTR__Pao:0.08544):0.03035,Fasciola_hepatica_Def_Loci-130_DNA__CMC-
EnSpm:0.13188):0.11764):0.07015,((Fasciola_gigantica_Def_Loci-
26_LINE__CR1:0.17399,(Fasciola_hepatica_Def_Loci-
10_LINE__CR1:0.22450,Fasciola_hepatica_Def_Loci-
98:0.23513):0.03632):0.07246,((((Fasciola_gigantica_Def_Loci-
44_LINE__CR1:0.04074,Trichobilharzia_regenti_Def_Loci-
77:0.18803):0.05111,((((Fasciola_gigantica_Def_Loci-
51_LINE__CR1:0.08136,Fasciola_hepatica_Def_Loci-
24_LTR__Pao:0.05186):0.02810,(Fasciola_gigantica_Def_Loci-
116:0.05753,((Fasciola_hepatica_Def_Loci-43:0.03582,(Fasciola_hepatica_Def_Loci-
48:0.08303,Fasciola_hepatica_Def_Loci-
104:0.06590):0.00952):0.01515,Fasciola_hepatica_Def_Loci-
63_LINE__CR1_LTR__Pao:0.00530):0.02968):0.02378):0.02368,Fasciola_hepatica_Def_Loci-
106_LTR__Pao:0.02734):0.03647,Fasciola_gigantica_Def_Loci-
128_LINE__CR1:0.04438):0.06104):0.02315,((((((Tre_cdhit_Clus-
242_Fasciola_gigantica_Def_Loci-45:0.22335,(Fasciola_gigantica_Def_Loci-
133_LINE__CR1:0.10792,Fasciola_hepatica_Def_Loci-
99_LTR__Pao:0.04886):0.02782):0.01534,(Fasciola_hepatica_Def_Loci-
46:0.06501,Fasciola_hepatica_Def_Loci-
119:0.16072):0.04311):0.01479,(Fasciola_hepatica_Def_Loci-
7_LINE__CR1:0.18183,Fasciola_hepatica_Def_Loci-
33_LINE__CR1_LTR__Pao:0.20884):0.05605):0.02070,Fasciola_gigantica_Def_Loci-
61:0.06646):0.00588,Fasciola_hepatica_Def_Loci-
64_LINE__CR1:0.07380):0.01445,Fasciola_hepatica_Def_Loci-

113_LTR__Pao:0.03148):0.03667):0.02332,(Fasciola_gigantica_Def_Loci-
117:0.31118,Fasciola_hepatica_Def_Loci-
128:0.22422):0.04619):0.03820):0.03236):0.01427,((((((Fasciola_gigantica_Def_Loci-
21_LINE__CR1:0.11280,Fasciola_gigantica_Def_Loci-
91_LINE__CR1_LTR__Pao:0.17311):0.03945,Fasciola_hepatica_Def_Loci-
83_LINE__CR1:0.01826):0.03062,Fasciola_hepatica_Def_Loci-
126_LINE__CR1:0.13956):0.07809,Paragonimus_westermani_Def_Loci-2_DNA__TcMar-
Tc1:0.18916):0.07519,((((Tre_cdhit_Clus-648_Fasciola_gigantica_Def_Loci-
104:0.06279,Fasciola_hepatica_Def_Loci-114_LINE__CR1:0.02356):0.11883,Tre_cdhit_Clus-
310_Fasciola_hepatica_Def_Loci-
60_LINE__CR1:0.00260):0.02958,Fasciola_hepatica_Def_Loci-
56_LINE__CR1:0.16451):0.01458,Fasciola_hepatica_Def_Loci-
100_LINE__CR1:0.04996):0.03877):0.04156,Fasciola_gigantica_Def_Loci-59_LINE__RTE-
BovB:0.26459):0.02372,(((((((((Paragonimus_heterotremus_Def_Loci-5_LINE__RTE-
BovB:0.07455,Paragonimus_westermani_Def_Loci-15_DNA__TcMar-
Tc1:0.08389):0.00637,Paragonimus_westermani_Def_Loci-30_LINE__RTE-
BovB_LTR__Gypsy:0.06066):0.03309,(((Paragonimus_westermani_Def_Loci-
6_LTR__Pao:0.01358,(((Paragonimus_westermani_Def_Loci-7_LINE__RTE-
BovB:0.05458,(Paragonimus_westermani_Def_Loci-11_LINE__RTE-
BovB:0.01360,Paragonimus_westermani_Def_Loci-
39_LINE__CR1:0.06473):0.02959):0.01733,Paragonimus_westermani_Def_Loci-
34:0.02721):0.01026,Paragonimus_westermani_Def_Loci-8_LINE__RTE-
BovB:0.03244):0.01114):0.04285,Paragonimus_westermani_Def_Loci-
4:0.04772):0.01114,(Paragonimus_westermani_Def_Loci-24_LINE__RTE-
BovB:0.04358,Paragonimus_westermani_Def_Loci-25_LINE__RTE-
BovB:0.04030):0.01121):0.02460):0.01379,Paragonimus_westermani_Def_Loci-
27_LINE__CR1_LINE__RTE-
BovB_LTR__Gypsy:0.01123):0.01924,Paragonimus_westermani_Def_Loci-
49:0.06475):0.03009,Paragonimus_westermani_Def_Loci-
41_LTR__Gypsy:0.10248):0.01964,Paragonimus_westermani_Def_Loci-1_LINE__RTE-
BovB:0.13617):0.02939,(Paragonimus_westermani_Def_Loci-22_LINE__RTE-
BovB:0.13162,Paragonimus_westermani_Def_Loci-23_LINE__RTE-
BovB:0.10723):0.05551):0.05036,Paragonimus_westermani_Def_Loci-
3:0.12445):0.05083,Paragonimus_westermani_Def_Loci-62_LINE__RTE-
BovB:0.16579):0.13666):0.03884):0.01028,((((((Clonorchis_sinensis_Def_Loci-
35_LINE__RTE-BovB_LTR__Gypsy:0.28292,Clonorchis_sinensis_Def_Loci-45_LINE__RTE-
BovB:0.14244):0.05503,Fasciola_hepatica_Def_Loci-
3_LTR__Pao:0.30436):0.04874,((Clonorchis_sinensis_Def_Loci-84_LINE__RTE-
BovB:0.15613,(Tre_cdhit_Clus-623_Clonorchis_sinensis_Def_Loci-
85:0.15800,(Opisthorchis_felineus_Def_Loci-2_LINE__CR1:0.00731,Tre_cdhit_Clus-
70_Opisthorchis_felineus_Def_Loci-74_LINE__CR1:0.04884):0.07097):0.05872):-
0.06109,Trematoda_Stephanostomum_sp_U83576.1:0.00000):0.09111):0.01808,(Clonorchis_si
nensis_Def_Loci-60_LINE:0.19617,(Paragonimus_westermani_Def_Loci-
16:0.10445,Schistosoma_mansoni_Def_Loci-145_LINE__RTE-
BovB:0.09150):0.10660):0.04183):0.03391,((Fasciola_gigantica_Def_Loci-
54_LINE__CR1:0.30952,Trematoda_Haematolechus_sp_U83578.1:0.00818):0.05308,(Opisthorch
is_felineus_Def_Loci-39:0.22146,Paragonimus_heterotremus_Def_Loci-33_LINE__RTE-
BovB:0.17655):0.07974):0.02461):0.02574,((Clonorchis_sinensis_Def_Loci-87_LINE__RTE-

BovB:0.28292,Opisthorchis_felineus_Def_Loci-
33:0.36641):0.07033,(Opisthorchis_felineus_Def_Loci-
105_LTR__Gypsy:0.21253,Trichobilharzia_regenti_Def_Loci-
34_LINE__Penelope:0.19629):0.06618):0.05062):0.02988):0.01556):0.00769):0.00175,(((((
Clonorchis_sinensis_Def_Loci-54:0.33526,(Opisthorchis_felineus_Def_Loci-
38:0.20165,Schistosoma_mansoni_Def_Loci-
11_LINE__CR1:0.04828):0.06004):0.05781,(((Clonorchis_sinensis_Def_Loci-20_LINE__RTE-
BovB:0.22611,Tre_cdhit_Clus-155_Schistosoma_mansoni_Def_Loci-141_LINE__RTE-
BovB:0.18310):0.06229,Paragonimus_heterotremus_Def_Loci-27_LINE__Penelope_LINE__RTE-
BovB:0.33625):0.03004,((Paragonimus_heterotremus_Def_Loci-
15_LTR__Gypsy:0.04806,Paragonimus_westermani_Def_Loci-
14:0.06664):0.06323,Paragonimus_westermani_Def_Loci-
26:0.20813):0.15297):0.04998):0.02260,(((Opisthorchis_felineus_Def_Loci-46_LINE__RTE-
BovB:0.14974,Schistosoma_bovis_Def_Loci-
11_LTR__Gypsy:0.11862):0.11492,Paragonimus_westermani_Def_Loci-
13:0.23810):0.04313,(Paragonimus_westermani_Def_Loci-33_DNA__MULE-
MuDR_LINE__CR1_LTR__Pao:0.19733,Schistosoma_bovis_Def_Loci-
12_LTR__Gypsy:0.08173):0.11929):0.04131):0.01849,(((((Clonorchis_sinensis_Def_Loci-
46_LINE__RTE-BovB:0.11700,(Tre_cdhit_Clus-481_Opisthorchis_felineus_Def_Loci-
41_LINE__RTE-BovB_LTR__Gypsy:0.07224,(Opisthorchis_felineus_Def_Loci-
63:0.09840,Opisthorchis_felineus_Def_Loci-
16_LINE__CR1_LINE__Penelope:0.10980):0.02936):0.02505):0.01852,Tre_cdhit_Clus-
80_Opisthorchis_felineus_Def_Loci-
42_LINE__CR1:0.17128):0.09667,Opisthorchis_felineus_Def_Loci-
6:0.26877):0.10510,(Opisthorchis_felineus_Def_Loci-56_DNA__MULE-
MuDR:0.27226,(Opisthorchis_felineus_Def_Loci-
104_LTR__Gypsy:0.18861,Paragonimus_westermani_Def_Loci-
53_LINE__Penelope:0.17663):0.12166):0.02185):0.02588,((Fasciola_gigantica_Def_Loci-
41:0.29741,Fasciola_hepatica_Def_Loci-
20_LINE__CR1:0.21808):0.09708,(Opisthorchis_felineus_Def_Loci-
11:0.21334,Opisthorchis_felineus_Def_Loci-
84:0.19341):0.05451):0.03812):0.02960):0.01131,(((Clonorchis_sinensis_Def_Loci-
3_DNA__PiggyBac:0.26290,((Fasciola_gigantica_Def_Loci-
115_LINE__CR1:0.10957,Schistosoma_mansoni_Def_Loci-
19:0.03892):0.06896,Schistosoma_mansoni_Def_Loci-
18_LTR__Gypsy:0.10210):0.09895):0.02794,(Opisthorchis_felineus_Def_Loci-
73:0.22346,Schistosoma_haematobium_Def_Loci-10_LINE__RTE-
BovB_LTR__Gypsy:0.13123):0.05112):0.01436,((Fasciola_gigantica_Def_Loci-
83_LINE__CR1:0.26137,Fasciola_gigantica_Def_Loci-
130_LINE__CR1:0.25025):0.07357,(Fasciola_hepatica_Def_Loci-
96_LINE_LINE__CR1:0.26786,Tre_cdhit_Clus-143_Schistosoma_bovis_Def_Loci-16_LINE__RTE-
BovB_LTR__Gypsy:0.05662):0.08498):0.04250):0.04065):0.00643):0.00669,(((Clonorchis_si
nensis_Def_Loci-5_LINE__RTE-BovB:0.28356,(Opisthorchis_felineus_Def_Loci-
64_LINE__RTE-BovB:0.02985,Opisthorchis_felineus_Def_Loci-
134:0.06119):0.23743):0.02066,(Clonorchis_sinensis_Def_Loci-44_LINE__RTE-
BovB:0.15193,Tre_cdhit_Clus-53_Schistosoma_bovis_Def_Loci-
13_LINE__CR1:0.04403):0.15205):0.05832,(Clonorchis_sinensis_Def_Loci-38_LINE__RTE-
BovB:0.22801,(Clonorchis_sinensis_Def_Loci-82:0.09803,Opisthorchis_felineus_Def_Loci-

68:0.16330):0.06122):0.09271):0.03450):0.00216,(((Opisthorchis_felineus_Def_Loci-
18_LINE__CR1:0.20185,Paragonimus_heterotremus_Def_Loci-
50:0.22130):0.12434,((Opisthorchis_felineus_Def_Loci-
96:0.27091,Trichobilharzia_regenti_Def_Loci-
43_tRNA:0.14103):0.06568,(Paragonimus_westermani_Def_Loci-
60:0.04248,Paragonimus_westermani_Def_Loci-
32:0.04658):0.22704):0.10112):0.03098,(((Opisthorchis_felineus_Def_Loci-
25_LINE__CR1:0.27661,Opisthorchis_felineus_Def_Loci-
65:0.31499):0.05042,(Paragonimus_westermani_Def_Loci-
57_LINE__CR1:0.27188,Paragonimus_westermani_Def_Loci-38_LINE__RTE-
BovB:0.24397):0.09587):0.01498,(Opisthorchis_felineus_Def_Loci-
28_LINE__Penelope:0.24229,Opisthorchis_felineus_Def_Loci-
83:0.35196):0.06533):0.02314):0.03987):0.00356,((((((Clonorchis_sinensis_Def_Loci-
14_LTR__Gypsy:0.06391,Opisthorchis_felineus_Def_Loci-98_LINE__RTE-
BovB_LTR__Gypsy:0.09628):0.12339,Clonorchis_sinensis_Def_Loci-65_LINE__RTE-
BovB_LTR__Gypsy:0.19739):0.04862,Opisthorchis_felineus_Def_Loci-
124:0.23695):0.07465,Fasciolopsis_buski_Def_Loci-
11_LINE__CR1:0.30289):0.05698,((Clonorchis_sinensis_Def_Loci-
30:0.24751,Opisthorchis_felineus_Def_Loci-
87:0.29939):0.04246,(Opisthorchis_felineus_Def_Loci-
35_LINE__L2:0.31981,Paragonimus_westermani_Def_Loci-29_LINE__RTE-
BovB:0.13623):0.06463):0.05968):0.02980,((((Clonorchis_sinensis_Def_Loci-
18:0.09122,Trichobilharzia_regenti_Def_Loci-
45_LINE__Penelope_Low_complexity:0.24767):0.12907,(Paragonimus_westermani_Def_Loci-
17:0.17380,Schistosoma_mansoni_Def_Loci-17_LINE__RTE-
BovB:0.26515):0.14819):0.06058,(((Fasciola_gigantica_Def_Loci-
80_LTR__Gypsy:0.10531,(Fasciola_gigantica_Def_Loci-
85_LINE__CR1:0.00000,Tre_cdhit_Clus-297_Fasciola_hepatica_Def_Loci-
35_LINE__CR1_LTR__Pao:0.14124):0.00879):0.22082,(Fasciola_gigantica_Def_Loci-
126_LINE__CR1:0.22695,(Fasciola_hepatica_Def_Loci-
9_LINE__CR1:0.02691,Fasciola_hepatica_Def_Loci-
12_LINE_LINE__CR1:0.06743):0.03755):0.04829):0.12582,(Fasciola_gigantica_Def_Loci-
101_DNA__TcMar-ISRm11_LINE__Rex-Babar:0.23367,(Paragonimus_westermani_Def_Loci-
12:0.15920,Trichobilharzia_regenti_Def_Loci-
51:0.07479):0.10230):0.08337):0.02716):0.01799,(((Fasciola_gigantica_Def_Loci-
96:0.19457,Fasciola_hepatica_Def_Loci-
112_LINE__CR1:0.26758):0.10014,(Fasciola_hepatica_Def_Loci-
137_LTR__Pao:0.18313,Tre_cdhit_Clus-136_Schistosoma_bovis_Def_Loci-
2:0.13967):0.10903):0.02470,((Fasciola_gigantica_Def_Loci-
79_LINE__CR1:0.14896,(Fasciola_hepatica_Def_Loci-
41_LINE__CR1:0.10002,Fasciola_hepatica_Def_Loci-
131_LINE__CR1_LTR__Pao:0.02581):0.11353):0.18379,Fasciola_hepatica_Def_Loci-
66_LINE__CR1:0.27493):0.04693):0.03321):0.00902):0.00356);
