# Supplementary material for: Evolution of SL-RNA Genes and Their Splicing Targets in Parasitic Flatworms
Source: Mol Biol Evol. 2025 Sep 23;42(11):msaf228. doi: 10.1093/molbev/msaf228 (PMC12582326; doi:10.1093/molbev/msaf228)
Supplement: msaf228_Supplementary_Data [file msaf228_supplementary_data.zip › Supplementary File 2.pdf]

Supplementary File 2: Full secondary structures predicted by RNAfold for the unique SL-RNAs sequences and the original reference sequences. For each unique SL-RNA 6 lines are shown: Line 1) Sequence ID (fasta format), Line 2) Sequence, Line 3) unpaired sequences set as input (the SM-like site), Line 4) Maximum Expected Accuracy (MEA) prediction, Line 5) Minimum Free Energy (MFE) prediction, and Line 6) Ensemble Free Energy prediction.

```
>Unique_SL_1
AAUGUUCGGUUUUCUGCCGUGUAUAUUAGUGCACGGUAAUAAUCGACUCCGACCUAUGGUCGGAUGAAUUCUUUGGCUAGCCCACC
..................................................................xxxxxxxxxx..........
.....((((((...((((((((((....))))))))))..)))))).(((((((...))))))).................... MEA
.....((((((...((((((((((....))))))))))..)))))).(((((((...))))))).................... MFE
...,.((((((...((((((((((....))))))))))..)))))),(((((((...))))))).................... EFE

>Unique_SL_2
CCGAUAAAUCGGUCCUUGCCUGCACUUUUGUAUGGUGAGUAUCGAUGCAGCUCAGGCUCUGCCUACGAGCUGACAGUAUUUGGCUGGUCCGACGAGGGC
..........................................................................xxxxxxxxx................
.......((((((.((((((((((....)))).)))))).)))))).((((((((((...))))..))))))..............((((.....)))) MEA
.......((((((.((((((((((....)))).)))))).)))))).((((((((((...))))..))))))..............((((.....)))) MFE
.......((((((.((((((((((....)))).)))))).)))))),((((((((((...))))..)))))).,............((((.....)))) EFE

>Unique_SL_3
CCGAUAAAUCGGUCCUUGCCUGCACUUUUGUAUGGUGAGUAUCGAUGCAGCUCAGCCUCUGCCUACGAGCUGACAGUAUUUGGCUGGUCCGACGAGGGC
..........................................................................xxxxxxxxx................
.......((((((.((((((((((....)))).)))))).)))))).((((((..(....).....))))))..............((((.....)))) MEA
.......((((((.((((((((((....)))).)))))).)))))).((((((.((....))....))))))..............((((.....)))) MFE
.......((((((.((((((((((....)))).)))))).)))))),((((((.({....},....)))))).,............((((.....)))) EFE

>Unique_SL_4
CCGAUAAAUCGGUCCUUACCUGCACUUUUGUAUGGUGAGUAUCGAUGCAGCUCAGGCUCUGCCUACGAGCUGACAGUAUUUGGCUGGUCCGACGAGGGC
..........................................................................xxxxxxxxx................
.......((((((.((((((((((....)))).)))))).)))))).((((((((((...))))..))))))..............((((.....)))) MEA
.......((((((.((((((((((....)))).)))))).)))))).((((((((((...))))..))))))..............((((.....)))) MFE
```

```
.......((((((.((((((((((....)))).)))))).)))))),((((((((((...))))..)))))).,............((((.....)))) EFE

>Unique_SL_5
CCGAUAAAUCGGUCCUUGCCUGCACUCUUGUAUGGUGAGUAUCGAUGCAGCUCAGCCUCUGCCUACGAGCUGACAGUAUUUGGCUGGUCCGACGAGGGC
..........................................................................xxxxxxxxx................
.......((((((.((((((((((....)))).)))))).)))))).((((((..(....).....))))))..............((((.....)))) MEA
.......((((((.((((((((((....)))).)))))).)))))).((((((.((....))....))))))..............((((.....)))) MFE
.......((((((.((((((((((....)))).)))))).)))))),((((((.({....},....)))))).,............((((.....)))) EFE

>Unique_SL_6
CCGAUAAAUCGGUCCUUACCUGCACUUCUGUAUGGUGAGUAUCGAUGCAGCUCAGGCUCUGCCUACGAGCUGACAGUAUUUGGCUGGUCCGACGAGGGC
..........................................................................xxxxxxxxx................
.......((((((.((((((((((....)))).)))))).)))))).((((((((((...))))..))))))..............((((.....)))) MEA
.......((((((.((((((((((....)))).)))))).)))))).((((((((((...))))..))))))..............((((.....)))) MFE
.......((((((.((((((((((....)))).)))))).)))))),((((((((((...))))..)))))).,............((((.....)))) EFE

>Unique_SL_7
CCGAUAAAUCGGUCCUUGCCUGCACUCUUGUAUGGUGAGUAUCGAUGCAGCUCAGGCUCUGCCUACGAGCUGACAGUAUUUGGCUGGUCCGACGAGGGC
..........................................................................xxxxxxxxx................
.......((((((.((((((((((....)))).)))))).)))))).((((((((((...))))..))))))..............((((.....)))) MEA
.......((((((.((((((((((....)))).)))))).)))))).((((((((((...))))..))))))..............((((.....)))) MFE
.......((((((.((((((((((....)))).)))))).)))))),((((((((((...))))..)))))).,............((((.....)))) EFE

>Unique_SL_8
ACCGUUAAUCGGUCCUUACCUUGCAGUUUUGUAUGGUGAGUAUCGAUGCAGCUGAGGCUGUGCCUACGAGCUGACCCAGUAUUUGGCUGGUCCUUCGAGGGC
.............................................................................xxxxxxxxx................
.......((((((.((((((.((((....)))).)))))).))))))(((((....)))))((((.((((..((((............)))).)))).)))) MEA
.......((((((.((((((.((((....)))).)))))).))))))(((((....)))))((((.((((..((((............)))).)))).)))) MFE
.......((((((.((((((.((((....)))).)))))).))))))(((((....)))))((((.((((..((((............)))).)))).)))) EFE

>Unique_SL_9
```

```
ACCGUUAAUCGGUCCUUACCUUGCAGUUUUGUAUGGUGAGUAUCGAUGCAGCUGAGGCUGUGCCUACGAGCUGACCCAUUAUUGGCUGGUCCUUCGAGGGC
...........................................................................xxxxxxxxxx................
.......((((((.((((((.((((....)))).)))))).))))))(((((....)))))((((.((((..((((...........)))).)))).)))) MEA
.......((((((.((((((.((((....)))).)))))).))))))(((((....)))))((((.((((..((((...........)))).)))).)))) MFE
.......((((((.((((((.((((....)))).)))))).))))))(((((....)))))((((.((((..((((...........)))).)))).)))) EFE

>Unique_SL_10
ACCGUUAAUCGGUCCUUACCUUGCAGUUUUGUAUGGUGAGUAUCGAUGCAGCUGAGGCUGUGCCUACGAGCUGACCCAGUAUUUGGCUGGUCCUUCGGGGGC
............................................................................xxxxxxxxx................
.......((((((.((((((.((((....)))).)))))).))))))(((((....)))))((((.((((..((((...........)))).)))).)))) MEA
.......((((((.((((((.((((....)))).)))))).))))))(((((....)))))((((.((((..((((...........)))).)))).)))) MFE
.......((((((.((((((.((((....)))).)))))).))))))(((((....)))))((({.((((..((((...........)))).)))).}))) EFE

>Unique_SL_11
ACCGUUAAUCGGUCCUUACCUUGCAGUUUUGUAUGGUGAGUAUCGAUGCAGCUGAGGCUGUGCCUACGAGCUGACCCAGUAUUUGGCCGGGCCCUCGAGGGC
............................................................................xxxxxxxxx................
.......((((((.((((((.((((....)))).)))))).))))))(((((....)))))((((.((((..(.(((..........)))).)))).)))) MEA
.......((((((.((((((.((((....)))).)))))).))))))(((((....)))))((((.((((..(.(((..........)))).)))).)))) MFE
.,,,...((((((.((((((.((((....)))).)))))).)))))){((((,.,,||}}}||||.|{||,,|.{{{.........,))||{|}}}.)))) EFE

>Unique_SL_12
ACCGUUAAUCGGUCCUUACCUUGCAGUUUUGUAUGGUGAGUAUCGAUGCAGCUGAGGCUGUGCCUACGAGGUGACCCAGUAUUUGGCUGGUCCUUCGAGGGC
............................................................................xxxxxxxxx................
.......((((((.((((((.((((....)))).)))))).))))))(((((....)))))((((.(((((.((((...........))))))))).)))) MEA
.......((((((.((((((.((((....)))).)))))).))))))(((((....)))))((((.(((((.((((...........))))))))).)))) MFE
.......((((((.((((((.((((....)))).)))))).))))))(((((....)))))((((.(((((.((((...........))))))))).)))) EFE

>Unique_SL_13
CCUUAACGGUUCUCUGCCCUGUAUAUUAGUGCAUGGUAAGAAUCGUUGGACCAUCGGUCCAAACCCAUUAUUGGCUAGCCUCCA
................................................................xxxxxxxxxx..........
.....(((((((.((((.(((((....))))).)))))))))))((((((...)))))).......................... MEA
```

```
.....((((((((.((((.(((((....))))).))))))))))))((((((...)))))).......................... MFE
....,((((((((.((((.(((((....))))).))))))))))))|(((((...)))))}......................... EFE

>Unique_SL_14
CCUUAACGGUUCUCUGCCCUGUAUAUUAGUGCAUGGUAAGAAUCGUUGGACCAUCGGUCCAAACCCAUUAAUUUGGCUAGCCUCCA
.................................................................xxxxxxxxxxx..........
.....((((((((.((((.(((((....))))).))))))))))))((((((...)))))).......................... MEA
.....((((((((.((((.(((((....))))).))))))))))))((((((...)))))).......................... MFE
....,((((((((.((((.(((((....))))).))))))))))))|(((((...)))))}......................... EFE

>Unique_SL_15
ACCGAUUAUCGGUCUUACCAUAAAACUUGUAUGGUGAGUAUCGAGGCAGCGUCUGAGCUGUUCAAUUGUUGGCUGGUCCUGCGGGGCC
..............................................................xxxxxxxxxx................
........((((((((((((((.......))))))))).)))))((((((......))))))............(((((....))))) MEA
........((((((((((((((.......))))))))).)))))((((((......))))))............(((((....))))) MFE
........((((((((((((((.......))))))))).)))))((((((......))))))............(((((....))))) EFE

>Unique_SL_16
CACCGAUUAUCGGUCUUACCAUAAAACUUGUAUGGUGAGUAUCGAGGCAGCACUUUAGCUGUCCAAUUAUUUGGCUGGUCUUUCGGGACC
................................................................xxxxxxxxxx................
.........((((((((((((((.......))))))))).)))))((((((......)))))).............(((((....))))) MEA
.........((((((((((((((.......))))))))).)))))((((((......)))))).............(((((....))))) MFE
.........((((((((((((((.......))))))))).)))))((((((......)))))).............(((((....))))) EFE

>Unique_SL_17
CCUAUACGGUUCUCUGCCGUGUGAAUUAGUGCAUGGUAAGAAUCGACUCCGGCCUAUGGUCGGAUGAAUUCUUUGGCUAGCCCACC
..................................................................xxxxxxxxxx..........
......(((((((.((((((((........)))))))))))))))..(((((((...))))))).................... MEA
......(((((((.((((((((........)))))))))))))))..(((((((...))))))).................... MFE
......(((((((.((((((((,......,)))))))))))))))..(((((((...))))))).................... EFE
```

```
>Unique_SL_18
UGGUUAUGGUUUUACUCUUGUGAUUUGUUGCAUGCUAAGAACCGUCGACCAAGAAUCGAAGUUUUCUUUGGCAGCCCUACA
...........................................................xxxxxxxxxxxx..........
(((((((((((((.....(((((....))))).....)))))))).))))).............................. MEA
(((((((((((((.....(((((....))))).....)))))))).))))).............................. MFE
(((((((((((((...,.(((({....})))).,...)))))))).))))).............................. EFE

>Unique_SL_19
CCGUCACGGUUUUACUCUUGUGAUUUGUUGCAUGGUAAGAACCGUCGACCAAGAAUCGAAGUUUUCUUUGGCAGCCCUACA
...........................................................xxxxxxxxxxxx..........
..((((((((((((((..(((((....))))).))))).)))))).)))................................ MEA
..((((((((((((((..(((((....))))).))).)))))))).)))................................ MFE
..((((((((((((({,.(((({....})))).)))))))))))).)))................................ EFE

>Unique_SL_20
UGGUCACGGUCUUACUCUUGUGAUUUGUUGCAUGCUAAGAACCGUCGACCAAGAAUCGAAGUUUUCUUUGGCAGCCCUACA
...........................................................xxxxxxxxxxxx..........
((((((((((((((....(((((....)))))...)))).))))).))))).............................. MEA
((((((((((((((....(((((....)))))...)))).))))).))))).............................. MFE
((((((((((((((....(((({....})))).,.)))),})))).))))).............................. EFE

>Unique_SL_21
CCGUCACGGUUUUACUCUUGUGAUUUGUUGCAUGGUAAGAACCGUCGACCAGAAUCGAAGCUUUCUUUUGCCAGCCCUGCA
...........................................................xxxxxxxxxxxx..........
..((((((((((((((..(((((....))))).))))).)))))).)))................................ MEA
..((((((((((((((..(((((....))))).))).)))))))).)))................................ MFE
..((((((((((((({,.(((({....})))).)))))))))))).))).............................,.. EFE

>Unique_SL_22
CCGUCACGGUUUUACUCUUGUGAUUUGUUGCAUGGUAAGAACCGUCGACCAAGAAUCGAAGCUUUCUUUGCAGCCCUGCA
............................................................xxxxxxxxxxx.........
```

..((((((((((((((..(((((....))))).))))).)))))).)))............................... MEA
..((((((((((((((..(((((....))))).))).)))))))).)))............................... MFE
..((((((((((((({,.(((({....})))).)))))))))))).)))............................... EFE

>Unique_SL_23
CCGUCACGGUUUUACUCUUGUGAUUUGUUGCAUGGUAAGAACCGUCCGACCAAGAAUCGAAGCUUUCUUUGCCAGCCCUGCA
....xxxxxxxxxxx..........
..((((((((((((((..(((((....))))).))))).))))))..)))................................ MEA
..((((((((((((((..(((((....))))).))).))))))))..)))................................ MFE
..((((((((((((({,.(((({....})))).))))))))))))..)))................................ EFE

>Unique_SL_24
CCGUCACGGUUUUACUCUUGUGAUUUGUUGCAUGGUAAGAACCGUCGACCAAGAAUCGAAGCUUUCUUUGCCAGCCCUGCA
....xxxxxxxxxxxx..........
..((((((((((((((..(((((....))))).))))).)))))).)))................................ MEA
..((((((((((((((..(((((....))))).))).)))))))).)))................................ MFE
..((((((((((((({,.(((({....})))).)))))))))))).)))................................ EFE

>Unique_SL_25
CCGUCACGGUUUUACUCUUGUGAUUUGCAUGGUAAGAACCGUCGACCAAGAAUCGAAGUUUUCUUUGGCAGCCCUACA
....xxxxxxxxxxxx..........
..((((((((((((((..(((.....))).))))).)))))).)))................................ MEA
..((((((((((((((..(((.....))).))).)))))))).)))................................ MFE
..((((((((((((({,.(((.....))).)))))))))))).)))................................ EFE

>Unique_SL_26
ACCGUUUAACGGUCCUUACCUCACUCGUUGUAUGGUGAGUACCGACAUGACUCGCUAGAGUUAUGCUAGUCUUUGGCUGGCCCGCAAGGGCC
....xxxxxxxxx................
.........((((.((((((..((.....))..)))))).)))).((((((((....)))))))).............(((((....))))) MEA
.........((((.((((((..((.....))..)))))).)))).((((((((....)))))))).............(((((....))))) MFE
.........((((.((((((..((.....))..)))))).)))).((((((((....)))))))).............(((((....))))) EFE

>Unique_SL_27
ACCGUUCAACGGUUCUUGCCUUGCUCGUUGUAUGGUGAGUACCGACAUGACUCAUUGAAGUCAUGCAAGUCUUUGGCUGGUCCGAAAGGGCC
....xxxxxxxxx................
.........((((.((((((.(((.....))).)))))).)))).(((((((......))))))).............(((((....))))) MEA
.........((((.((((((.(((.....))).)))))).)))).(((((((......))))))).............(((((....))))) MFE
.........((((.((((((.(((.....))).)))))).)))).(((((((......))))))).............(((((....))))) EFE

>Unique_SL_28
ACCGUUAAUCGGUCCUUACCUUGCAAUUUUGUAUGGUGAGUAUCGAUGCAGCUCGGGCUCUGGCUACGAGCUGACCCAGUAUUUGGCUGGUCCGUCAAGGGC
....xxxxxxxxx................
.......((((((.((((((.((((....)))).)))))).)))))).((((((((((....))).)))))))................((((.....)))) MEA
.......((((((.((((((.((((....)))).)))))).)))))).((((((((((....))).)))))))................((((.....)))) MFE
.......((((((.((((((.((((....)))).)))))).)))))).((((((((((....))).)))))))................((((.....)))) EFE

>Unique_SL_29
ACCGUUAAUCGGUCCUUACCUUGCAAUUUUGUAUGGUGAUAUCGAUGCAGCUCGGGCUCUGGCUACGAGCUGACCCAGUAUUUGGCUGGUCCGUCAAGGGC
....xxxxxxxxx................
.......((((((..(((((.((((....)))).))))).)))))).((((((((((....))).)))))))................((((.....)))) MEA
.......((((((..(((((.((((....)))).))))).)))))).((((((((((....))).)))))))................((((.....)))) MFE
.......((((((..(((((.((((....)))).))))).)))))).((((((((((....))).)))))))................{(((.....)))} EFE

>Unique_SL_30
ACCGUUAAUCGGUCCUUACCUUGCAAUUUUGUAUGGUGAGUAUCGAUGCAGCUCGGGCUCUGGCUACGAGCUGACCAGUUUGGCUGGUCCGUCAAGGGC
....xxxxxxx................
.......((((((.((((((.((((....)))).)))))).)))))).((((((((((....))).))))))).............((((.....)))) MEA
.......((((((.((((((.((((....)))).)))))).)))))).((((((((((....))).))))))).............((((.....)))) MFE
.......((((((.((((((.((((....)))).)))))).)))))).((((((((((....))).)))))))...........,.((((.....)))) EFE

>Unique_SL_31
ACCGUUAAUCGGUCCUUACCUUGCAAUUUUGUAUGGUGAGUAUCGAUGCAGCUCGGGCUCUGGCUACGAGCUGACCCAGAAUUUGGCUGGUCCGUCAAGGGC

```
..............................................................................xxxxxxxx................
.......((((((.((((((.((((....)))).)))))).)))))).((((((((((....))).)))))))................((((.....)))) MEA
.......((((((.((((((.((((....)))).)))))).)))))).((((((((((....))).)))))))................((((.....)))) MFE
.......((((((.((((((.((((....)))).)))))).)))))).((((((((((....))).)))))))................((((.....)))) EFE

>Unique_SL_32
CCUAUACGGUUCUCUGCCGUGUAUAUUAGUGCAUGGUAAGAAUCGACUCCGGCCUAUGGUCGGAUGAAUUCUUUGGCUAGCCCACC
..................................................................xxxxxxxxxx..........
......(((((((.((((((((((....)))))))))))))))))..(((((((...)))))))...................... MEA
......(((((((.((((((((((....)))))))))))))))))..(((((((...)))))))...................... MFE
......(((((((.((((((((((....)))))))))))))))))..(((((((...)))))))...................... EFE

>Unique_SL_33
GUCGAGUUACUCGAUUCUUGCCUGCAGUUGUGUAUGGUGAGUAUCGAUGCAGCUGAGGCUCUGCCUACGAGCUGACGCAGUAUUUGGCUGGUCCGACGAGGA
..............................................................................xxxxxxxxx...............
((((......(((((.((((((((((....)))).)))))).))))).(((((((((((...)))).).))))).).................))))..... MEA
((((......(((((.((((((((((....)))).)))))).)))))..((((((((((...)))).).)))))...................))))..... MFE
((((......(((((.((((((((((....)))).)))))).))))).{((((((((((...)))).).))))).}................,))))..... EFE

>Unique_SL_34
ACCGUUAAUCGGUCCUUACCUUGCAAUUUUGUAUGGUGAGUAUCGAUGCAGCUCAGGCUGUGCCUACGAGCUGACAGUAUUUGGCUGGUCCGACGAGGAC
...........................................................................xxxxxxxxx................
.......((((((.((((((.((((....)))).)))))).)))))).((((((((((...))))..))))))..............((((.....)))) MEA
.......((((((.((((((.((((....)))).)))))).)))))).((((((((((...))))..))))))..............((((.....)))) MFE
.......((((((.((((((.((((....)))).)))))).)))))),((((((((((...))))..)))))).,............((((.....)))) EFE

>Unique_SL_35
CCUUAACGGUUCUCUGUCCCUGUAUAUUAGUGCAUGGUAAGAAUCGUUGGACCAUCGGUCCAAACCCAUUAUUUGGCUAGCCUUCC
..................................................................xxxxxxxxxx..........
.....((((((((....((.(((((....))))).))..))))))))((((((...)))))))........................ MEA
.....((((((((.((.((.(((((....))))).))))))))))))((((((...)))))))........................ MFE
```

....,((((((((.{,.((.(((((....))))).)),,))))))))|(((((...)))))}........................ EFE

>Unique_SL_36
UAACAGCGGUUCACUGCCCUGUAUAUUAGUGCAUGGUAAGAAUCGUUGGACCAUCGGUCCAAACCCAUUAUUUGGCUAGCCUCCA
....xxxxxxxxxx..........
.....(((((((..((((.(((((....))))).)))).)))))))((((((...))))))........................ MEA
.....(((((((..((((.(((((....))))).)))).)))))))((((((...))))))........................ MFE
....,(((((((..((((.(((((....))))).)))).)))))))|(((((...)))))}........................ EFE

>Unique_SL_37
CCUUAACGGUUCUCUGCCCUGUAUAUUAGUGCAUGGCAAGAAUCGUUGGACCAUCGGUCCAAACCCAUUAUUUGGCUAGCCUCCA
....xxxxxxxxxx..........
.....((((((((.((((.(((((....))))).))))))))))))((((((...))))))........................ MEA
.....((((((((.((((.(((((....))))).))))))))))))((((((...))))))........................ MFE
....,((((((((.((((.(((((....))))).))))))))))))|(((((...)))))}........................ EFE

>Unique_SL_38
UAACGGUUCUCUGCCCUGCUGUAUAUUAGUGCAUGGUAAGAAUCGUUGGACCAUCAGUCCAAAUCCAUUAUUUGGCUAGCCUCGA
....xxxxxxxxxx..........
..((((((((.((((.(((...........))).))))))))))))(((((.....)))))........................ MEA
..((((((((.((((.(((...........))).))))))))))))(((((.....)))))........................ MFE
.,((((((((.((((.(((...........))).))))))))))))|((((.....))))}........................ EFE

>Unique_SL_39
UAACGGUUCUCUGCCCUGCUGUGUAUUAGUGCAUGGUAAGAAUCGUUGGACCAUCAGUCCAAAUCCAUUAUUUGGCUAGCCUCUA
....xxxxxxxxxx..........
..((((((((.((((.(((...........))).))))))))))))(((((.....)))))........................ MEA
..((((((((.((((.(((...........))).))))))))))))(((((.....)))))........................ MFE
.,((((((((.((((.(((...........))).))))))))))))|((((.....))))}........................ EFE

>Unique_SL_40

```
CCGAUUAAUCGGUCUUACCUUGCAAUUUUUGUAUGGUGAGUAUCGAUACAGCACUUGGCUCUGCCUUGUGUCUGUGAGAUUCUUUGGCUGGUCCUUGCGGGCC
.............................................................................xxxxxxxxxx................
.......((((((((((((.(((((...))))).)))))).))))))((((((((..(((...)))..))).)))).............(((((....))))) MEA
.......((((((((((((.(((((...))))).)))))).))))))((((((((..(((...)))..))).)))).............(((((....))))) MFE
.......((((((((((((.(((({...})))).)))))).))))))((((((((..(((...)))..))).)))).............(((((....))))) EFE

>Unique_SL_41
CCGAGUAUUCGGUCUUACUAUACGAACUUGUAUGGUGAGUAUCGUGACAGCUCUCGUUUUGCUUGAGCUGUAGAGUUUUUGGCUGGUCCGCGAGGGCC
.........................................................................xxxxxxxxx................
.........(((((((((((((((....))))))))))).))))..(((((((..((...))..))))))).............(((((....))))) MEA
.........(((((((((((((((....))))))))))).))))..(((((((..((...))..))))))).............(((((....))))) MFE
,,,,....,|||{(((((((((({....})))))))))).}}}}..(((((((..({...})..))))))).............(((((....))))) EFE

>Unique_SL_42
CCGGAUAAUCGGUCUUACUGUACUAACUUGUAUGGUGAGUAUCGAAUCAGUUCUUGCUUUGCUCGAACUGUACUUUUGGCUGGCCCGCGAGGGCC
.......................................................................xxxxxxxxx................
........(((((((((((((((......)))))))))).)))))..((((((..((...))..))))))............(((((....))))) MEA
........(((((((((((((((......)))))))))).)))))..((((((..((...))..))))))............(((((....))))) MFE
........(((((((((((((((......)))))))))).)))))..((((((..((...))..))))))............(((((....))))) EFE

>Unique_SL_43
ACCGUUUUGCGGUCUUGCCAUAAAGUUUGUAUGGUGAGUAUCGAGGCAGCUCUUUAGCUGUCCAAUUGUUUGGCUGGUCUUACGGGACC
...............................................................xxxxxxxxxx................
.(((.....)))((((((((((.......))))))))).)....(((((((....))))))).............(((((....))))) MEA
.........(((((((((((((.......))))))))).)))).(((((((....))))))).............(((((....))))) MFE
,{{{{...,||||(((((((((.......))))))))).,,,|.(((((((....))))))),............(((((....))))) EFE

>Unique_SL_44
ACCGUUUACCGGUCUUACCUUGCAAUUUUUGUAUGGUGAGUAUCGAUACAGCGCUUGGCUUUGCCUUGUGUCUGUAAAAUUCUUUGGCUGGUCCUAACGGGCC
.............................................................................xxxxxxxxxx................
.........((((((((((.(((((...))))).)))))).)))).((((((((..(((...)))..))).))))).............(((((....))))) MEA
```

```
.........((((((((((.(((((...))))).)))))).)))).((((((((..(((...)))..))).))))).............(((((....))))) MFE
.........((((((((((.(((({...}))))).)))))).)))).((((((((..(((...)))..))).))))).............(((((....))))) EFE

>Unique_SL_45
ACCGAUUAUCGGUAUUUACCACAUUUAUUGUAUGGUGAGUAUCGAUGCAGCUCAUGUAGCUGUGAAAGAAUUUGGCUGGCCCGGAAGGGCC
....................................................................xxxxxxx................
.......((((((((((((((((.....))..))))))))))))))((((((.....))))))..............(((((....))))) MEA
.......((((((((((((((.((.....)).))))))))))))))((((((.....))))))..............(((((....))))) MFE
.......(((((((((((((({(,....,)}.))))))))))))))((((((.....))))))..............(((((....))))) EFE

>Unique_SL_46
ACCGUUUAUCGGUGUUUGCCACAUUCAUUGUAUGGUGAGUAUCGAUGCAGCUCAAGCAGCUGUGGAAGUGUUUGGCUGGCCCGAAAGGGCC
..................................................................xxxxxxxxx................
.......((((((((((((((((.....))..))))))))))))))((((((.....))))))..............(((((....))))) MEA
.......((((((((((((((.((.....)).))))))))))))))((((((.....))))))..............(((((....))))) MFE
.......(((((((((((((({(,....,)}.))))))))))))))((((((.....))))))..............(((((....))))) EFE

>Unique_SL_47
UAUAUAUGGUUCUCUGCCGUGUAUCAGUGCAUGGUAAGAAUCGAGUUCGACUCACUCAGUUGGUCGAAUUAAUUAUUUGGCUAGCC
......................................................................xxxxxxxxxx......
.......((((((.(((((((((....))))))))))))))).(((((((((.((...)).)))))))))................ MEA
.......((((((.(((((((((....))))))))))))))).(((((((((.((...)).)))))))))................ MFE
......,((((((.(((((((((....))))))))))))))),(((((((((.({...}}.)))))))))................ EFE

>Unique_SL_48
CCGUCACGGUUUUACUCUUGUGAUUUGUUGCAUAGUAAGAACCGUCGACCAAGAAUCGAAGUUUUCUUUGGCAGCCCUACA
...........................................................xxxxxxxxxxxx..........
..((((((((((((((..(((((....))))).))))).)))))).)))................................ MEA
..((((((((((((((..(((((....))))).))).)))))))).)))................................ MFE
..((((((((((((((..(((({....})))).)))))))))))).)))................................ EFE
```

>Unique_SL_49
CCGUCACGGUUUUACUCUUGUGAUUUGUUGCAUGGUAAGAACCGUCGACCAAGAAUCAAAGUUUUCUUUGGCAGCCGUACA
....xxxxxxxxxxxx..........
..((((((((((((((..(((((....))))).))))).)))))).)))................................ MEA
..((.((((((.........((((((((((.(((((....)))))))))...))))))..............)))))))). MFE
..((((((((((((({..(((({....|}))).)))}}}})))))).)))................................ EFE

>Unique_SL_50
CAGUCGCGCUUUUACUCUUGUGAUUUGUUGCAUGGUAAGAACCGUCGACCAGGAAUCGAAGUUUUUUUGGAACUCCCACA
....xxxxxxxxxxx..........
..((((((.(((((((..(((((....))))).)))))))..)).))))..(.......................).... MEA
..((((((.(((((((..(((((....))))).)))))))..)).))))............................... MFE
..((((((.(((((({..(((({....})))).)))))))..)).))))..{{,...................,,}.... EFE

>Unique_SL_51
UUUUCACGGUUUUACUCUUGUGAUUUUUUGCAUGGUAAGAACCGUCGACCAAGAGUCGAAGGUUUCUUUGCCAGCCCUUCA
....xxxxxxxxxxx..........
.....(((((((((((..(((((....))))).))))).))))))((((.....)))))....................... MEA
.....(((((((((((..(((((....))))).))).))))))))((((.....))))....................... MFE
.....,((((((((({,.(((((....))))).)))))))))))|((((.....)))),...................... EFE

>Unique_SL_52
CCGUCACGGUUUUACUCUUGUGAUUUGUUGCAUGGUAAGAACCGUCGACCAAGAAUCGAAGCUUUCUUUGAACUGACAGUU
....xxxxxxxxxxx..........
..((((((((((((((..(((((....))))).))))).)))))).)))................................ MEA
..((((((((((((((..(((((....))))).))).)))))))).)))................................ MFE
..((((((((((((({,.(((({....})))).)))))))))))).)))................................ EFE

>Unique_SL_53
CCGUCACGGUUUUACUCUUGUGAUUUGUUGCAUGGUAAGAACCGUCGACCAAGAAUCGAAGCUUUCUUAGACACUGAACA
....xxxxxxxxxx..........

```
..((((((((((((((..(((((....))))).))))).)))))).))).............................. MEA
..((((((((((((((..(((((....))))).))).)))))))).))).............................. MFE
..((((((((((((({,.(((({....})))).)))))))))))).))).............................. EFE
```

```
>Unique_SL_54
UACUCACGGUUUUACUCUUGUGAUUUGUUGCAUGGUAAGAACUGUCGACGAAGAAUCGAAGUUUUCUUUGGCAGCCCUACA
...........................................................xxxxxxxxxxxx..........
...(((((((((((((..(((((....))))).))))).)))))).))(((....)))....................... MEA
...((((((........))))))(((((((.(((((....))))))))))))............................. MFE
...{{(((((((((({..(((({....})))).)))})))))))).)}{{{....}))....................... EFE
```

```
>Unique_SL_55
UAGUCACGGUCUUACUAUUGUGAUUUGUUGCAUGGUAAGAACCGUCGACCAAGAAUCGAAGUUUUCUUUGGCAGCCCUACA
...........................................................xxxxxxxxxxxx..........
..(((((((((((((((.(((((....)))))))))))).))))).)))................................ MEA
..((((((((((((((((.((((....)))))))))))).))))).)))................................ MFE
..(((((((((((((((,{((({....})))))))))),,)))).)))................................ EFE
```

```
>Unique_SL_56
CCGUCACGGUUUUACUCUUGUGAUUUAUUGCAUGGUAAGAACCGUCGACCAAGAAUCGAAGUUUUCUUUGGCAACCCUACA
...........................................................xxxxxxxxxxxx..........
..((((((((((((((..(((((....))))).))))).)))))).)))................................ MEA
..((((((((((((((..(((((....))))).))).)))))))).)))................................ MFE
..((((((((((((({,.(((({....})))).)))))))))))).)))................................ EFE
```

```
>Unique_SL_57
CCGUCACGGUUUUACUCUUGUGAUUUGUUGCAUGGUAAGAACCGUCAACCAAGAAUCGAAGUUUCUUUUGGCAGCCCUACA
...........................................................xxxxxxxxxxxx..........
.....(((((..((((..(((((....))))).))))...))))).................................... MEA
..(((((((........)))))))..((((.(((((....)))))))))................................ MFE
..,,.((((({{{{{,..{{{{|...,|||}|,|||,,,,))))),,}}................................ EFE
```

```
>Unique_SL_58
GGCUGACGGUUUUACUCUUGUGAUUUGUUGCAUGGUAAGAACCGUCGACCAAGAAUCGAAGUUUCUUUGGCAGCCCUACA
..........................................................xxxxxxxxxxxx..........
((.(((((((((((((..(((((....))))).))))).)))))))).)).............................. MEA
((.(((((((((((((..(((((....))))).))).)))))))))).)).............................. MFE
((,(((((((((((({,.(((({....}))))).))))))))))))||,}}....,,,.............,,,,,..... EFE
```

```
>Unique_SL_59
ACCGUUCAACGGUUCUUGCCUUGCUCGUUGUAUGGUGAGUACCGACAUGACUCAUUAAAGUCAUGCAAGUCUUUGGCUGGUCCGAAAGGGCC
...................................................................xxxxxxxxx................
.........((((.((((((.(((.....))).)))))).)))).(((((((......))))))).............(((((....))))) MEA
.........((((.((((((.(((.....))).)))))).)))).(((((((......))))))).............(((((....))))) MFE
.........((((.((((((.(((.....))).)))))).)))).(((((((......))))))).............(((((....))))) EFE
```

```
>Unique_SL_60
ACCGUAGAUCGGUUCUUACCCUACGAUAUUGUAUGGUGAGUAUCGAUACGGCUCGAGACUACGAGCUGUUAUUGUUUGGCUGGUCCUAACAGGGG
.....................................................................xxxxxxxxxx................
.((....((((((.((((((.((((....)))).)))))).))))))((((((((......))))))))..........(((.......))))). MEA
.((....((((((.((((((.((((....)))).)))))).))))))((((((((......))))))))..........(((.......))))). MFE
.((....((((((.((((((.((({....}))).)))))).))))))((((((((......))))))))..........,{|,,,{,..,}))). EFE
```

```
>Unique_SL_61
ACCGUAGAUCGGUUCUUACCCUACGAUAUUGUAUGGUGAGUAUCGAUACGGCUCGAGACUACGAGCUGUCAUUGUUUGGCUGGCUCCUAGCGGGG
.....................................................................xxxxxxxxxx................
.(((...((((((.((((((.((((....)))).)))))).))))))((((((((......))))))))..............(.....)))).. MEA
.......((((((.((((((.((((....)))).)))))).))))))((((((((......))))))))..........((.(((...))).)). MFE
.{{{,..((((((.((((((.((({....}))).)))))).))))))((((((((......))))))))..........,{.{{,.,,}))})}. EFE
```

```
>Unique_SL_62
CCGUUUAGUCGGUCUUACCUACGAGUGUUGUAUGGUGAGCAUCAAUAUAGCUAGGGCUCUGCCCAGGAGCUAUCGUAGUAUUUGGCUGGCCCAGCGUGGGC
```

```
............................................................................xxxxxxxxx................
..........((((((((((((((...))))).)))))).)))...((((((.((((...))))...))))))...............(((((...))))) MEA
(((......))).(((((((((((...))))).)))))).......((((((.((((...))))...))))))...............(((((...))))) MFE
,,,......,||{(((((((((({...})))).)))))).}}}...((((((.((((...))))...))))))...............(((({...})))) EFE
```

```
>Unique_SL_63
GCCGUUUAGUCGGUCUUACCUACGAGUGUUGUAUGGUGAGCAUCGAUGCAGCUCGGGCUUUGUCCAGGAGCUGUUGUAGUAUUUGGCUGGCCCGGCGGGGCC
.............................................................................xxxxxxxxx................
........(((((((((((((((((...))))).)))))).))))))(((((((((((...))))..)))))))..............(((((....))))) MEA
........(((((((((((((((((...))))).)))))).))))))(((((((((((...))))..)))))))..............(((((....))))) MFE
........(((((((((((((((({...})))).)))))).))))))(((((((((((...))))..)))))))..............(((((....))))) EFE
```

```
>Unique_SL_64
ACCGUAGAUCGGUUCUUACCCUACGCUAUUGUAUGGUGAGUAUCGAUACGGCUCGGGCUAAGCCUACGAGCUGUUAUUCUUUGGCUGGUCCUGCUGAGGG
..........................................................................xxxxxxxxxx................
.((....((((((.((((((.(((......))).)))))).))))))(((((((((((...)))..))))))))............)).(((....))). MEA
((((...((((((.((((((.(((......))).)))))).))))))(((((((((((...)))..))))))))...........))))(((....))). MFE
,((,...((((((.((((((.(((,....,))).)))))).))))))(((((((((((...)))..))))))))............}},(((....))). EFE
```

```
>Unique_SL_65
CCGUCACGGUUUUACUCUUGUGAUUUAUUGCAUGGUAAGAACCGUCGACUAAGAAUCGAAGCAUUAUUUGGCAGCUCCUCA
.............................................................xxxxxxxxxx..........
..((((((((((((((..(((((....))))).))))).)))))).)))................................ MEA
..((((((((((((((..(((((....))))).))).)))))))).)))................................ MFE
..((((((((((((({,.(((({....})))).))))))))))))).)))................................ EFE
```

```
>Unique_SL_66
CCGUCACGGUUUUACUCUUGUGAUUUAUUGCAUGGUAAGGACCGUCGACCAAGAAUCGAAUCAUUAUUUGGCAGCCCCUCA
.............................................................xxxxxxxxxx..........
..((((((((((((((..(((((....))))).))))).)))))).)))................................ MEA
..((((((((((((((..(((((....))))).))))).)))))).)))................................ MFE
```

```
..(((((((((((((({,.(((({....})))).))))))))))))).)))................................ EFE

>Unique_SL_67
CCGUCACGGUUUUACUCUUGUGAUUUAUUGCAUGGUAAGAACCGUCGACCAAGAAUCGAAUCAUUAUUUGGCAGCCCUUCA
.............................................................xxxxxxxxxx..........
..((((((((((((((..(((((....))))).))))).)))))).)))................................ MEA
..((((((((((((((..(((((....))))).))).)))))))).)))................................ MFE
..(((((((((((((({,.(((({....})))).))))))))))))).)))................................ EFE

>Unique_SL_68
CCGUCAUGGUUUUACUCUUGUGAUUUAUUGCAUGGUAAGAACCGUCGACCAAGAAUCGAACCAUUAUUUGGCAGCCCCUCG
.............................................................xxxxxxxxxx..........
..((((((((((((((..(((((....))))).))))).)))))).)))................................ MEA
..((((((((((((((..(((((....))))).))).)))))))).)))................................ MFE
..(((((((((((((({,.(((({....})))).))))))))))))).)))................................ EFE

>Unique_SL_69
CCGUCACGGUUUGACUCUUGUGAUUUAUUAUUGCAUGGUAAGAACCGUCGACCAAGAAUCGAAGCAUUAUUUGGCAAAACA
................................................................xxxxxxxxxx.......
..((((((((((.(((..((((((.....)))))).)))..))))))).)))............................. MEA
..((((((((((.(((..((((((.....)))))).)))..))))))).)))............................. MFE
..((((((((((.{{{{.((((((.....)))))).))}..))))))).)))............................. EFE

>Unique_SL_70
CCGUCACGGUUUUACUCUUGUGAUUUAUUGCAUGGUAAGAACCGUCGACCAAGAAUCGAAUCAUUAUUUGGCAGCCUCUCA
.............................................................xxxxxxxxxx..........
..((((((((((((((..(((((....))))).))))).)))))).)))................................ MEA
..((((((((((((((..(((((....))))).))).)))))))).)))................................ MFE
..(((((((((((((({,.(((({....})))).))))))))))))).)))................................ EFE

>Unique_SL_71
```

CCGUCACGGUUUUACUCUUGUGAUUUAUUGCAUGGUAAGAACCGUCGACCAAGAAUCGAAGCAUUAUUUGGCAGCCCCCC
....xxxxxxxxxx..........
..((((((((((((((..(((((....))))).))))).)))))).)))................................ MEA
..((((((((((((((..(((((....))))).))).)))))))).)))................................ MFE
..((((((((((((({,.(((({....})))).)))))))))))).)))................................ EFE

>Unique_SL_72
ACCGUUAAUCGGUCCUUACCUUGCAAUUUUGUAUGGUGAGUAUCGAUGCAGCUCAGGCUGUGCCUACGAGCUGACCCAGUAUUUGGCUGGUCCUUCGAGGGC
....xxxxxxxxx................
.......((((((.((((((.((((....)))).)))))).))))))(((((....)))))((((.((((..((((...........)))).)))).)))) MEA
.......((((((.((((((.((((....)))).)))))).))))))(((((....)))))((((.((((..((((...........)))).)))).)))) MFE
.......((((((.((((((.((((....)))).)))))).))))))(((((....)))))((((.((((..((((...........)))).}))).)))) EFE

>Unique_SL_73
CCUAUACGGUUCUCUGCCGUGUAUCAGUGCAUGGUAAGAAUCGAGUUCGACUCACAUCGUUGGUCGAAUAGAUUAUUUGGCUAGCCUCCA
....xxxxxxxxxx..........
......(((((((.(((((((((....)))))))))))))))).((((((((.((...)).))))))))).................... MEA
......(((((((.(((((((((....)))))))))))))))).((((((((.((...)).))))))))).................... MFE
......(((((((.(((((((((....)))))))))))))))).((((((((.({...}}.))))))))).................... EFE

>Unique_SL_74
CCUAUACGGUUCUCUGCCGUGUAUAUUAGUGCAUGGUAAGAAUCGAAUUCGACCUAUGGUCGAAUAAAUUCUUUGGCUAGCCUCUU
....xxxxxxxxxx..........
......(((((((.((((((((((....))))))))))))))))).((((((((...))))))))).................... MEA
......(((((((.((((((((((....))))))))))))))))).((((((((...))))))))).................... MFE
......(((((((.((((((((((....))))))))))))))))).((((((((...))))))))).................... EFE

>Unique_SL_75
CCGUCACGGGUUUUACUCUUGUGAUUUGUUGCAUGGUAAGAACCGUCGACCAAGAAUCGAAGUUUCUUUGGCAGCCCUAC
....xxxxxxxxxxxx.........
..(((((((.(((((((..(((((....))))).))))))).)))).)))).............................. MEA

```
..(((((((.(((((((..(((((....))))).))))))).)))).)))............................... MFE
..(((((((.{((((({,.(((({....})))).))))))}.)))).)))............................... EFE

>Cestodes_Echinococcus_multilocularis_Emu.SL1_sm1
ACCGUUAAUCGGUCCUUACCUUGCAGUUUUGUAUGGUGAGUAUCGAUGCAGCUGAGGCUGUGCCUACGAGCUGACCCAGUAUUUGGCUGGUCCUUCGGGGGC
.............................................................................xxxxxxxxx................
.......((((((.((((((.((((....)))).)))))).))))))(((((....)))))((((.((((..((((............)))).)))).)))) MEA
.......((((((.((((((.((((....)))).)))))).))))))(((((....)))))((((.((((..((((............)))).)))).)))) MFE
.......((((((.((((((.((((....)))).)))))).))))))(((((....)))))((({.((((..((((............)))).)))).}))) EFE

>Cestodes_Echinococcus_multilocularis_Emu.SL2a_sm1
CCGAUAAAUCGGUCCUUGCCUGCACUCUUGUAUGGUGAGUAUCGAUGCAGCUCAGGCUCUGCCUACGAGCUGACAGUAUUUGGCUGGUCCGACGAGGGC
..........................................................................xxxxxxxxx................
.......((((((.((((((((((....)))).)))))).)))))).((((((((((...))))..))))))..............((((.....)))) MEA
.......((((((.((((((((((....)))).)))))).)))))).((((((((((...))))..))))))..............((((.....)))) MFE
.......((((((.((((((((((....)))).)))))).)))))),((((((((((...))))..)))))).,............((((.....)))) EFE

>Cestodes_Echinococcus_multilocularis_Emu.SL2b_sm1
CCGAUAAAUCGGUCCUUGCCUGCACUUUUGUAUGGUGAGUAUCGAUGCAGCUCAGCCUCUGCCUACGAGCUGACAGUAUUUGGCUGGUCCGACGAGGGC
..........................................................................xxxxxxxxx................
.......((((((.((((((((((....)))).)))))).)))))).((((((..(....).....))))))..............((((.....)))) MEA
.......((((((.((((((((((....)))).)))))).)))))).((((((.((....))....))))))..............((((.....)))) MFE
.......((((((.((((((((((....)))).)))))).)))))),((((((.({....},....)))))).,............((((.....)))) EFE

>Cestodes_Echinococcus_multilocularis_Emu.SL2c_sm1
CCGAUAAAUCGGUCCUUACCUGCACUUUUGUAUGGUGAGUAUCGAUGCAGCUCAGGCUCUGCCUACGAGCUGACAGUAUUUGGCUGGUCCGACGAGGGC
..........................................................................xxxxxxxxx................
.......((((((.((((((((((....)))).)))))).)))))).((((((((((...))))..))))))..............((((.....)))) MEA
.......((((((.((((((((((....)))).)))))).)))))).((((((((((...))))..))))))..............((((.....)))) MFE
.......((((((.((((((((((....)))).)))))).)))))),((((((((((...))))..)))))).,............((((.....)))) EFE
```

>Cestodes_Echinococcus_multilocularis_Emu.SL2d_sm1
CCGAUAAAUCGGUCCUUACCUGCACUUCUGUAUGGUGAGUAUCGAUGCAGCUCAGGCUCUGCCUACGAGCUGACAGUAUUUGGCUGGUCCGACGAGGGC
....xxxxxxxxx................
.......((((((.((((((((((....)))).)))))).)))))).((((((((((...))))..))))))..............((((.....)))) MEA
.......((((((.((((((((((....)))).)))))).)))))).((((((((((...))))..))))))..............((((.....)))) MFE
.......((((((.((((((((((....)))).)))))).)))))),((((((((((...))))..)))))).,............((((.....)))) EFE

>Cestodes_Echinococcus_granulosus_AJ292377.1_sm1
ACCGUUAAUCGGUCCUUACCUUGCAAUUUUGUAUGGUGAGUAUCGAUGCAGCUCAGGCUGUGCCUACGAGCUGACCCAGUAUUUGGCUGGUCCUUCGAGGGC
....xxxxxxxxx..................
.......((((((.((((((.((((....)))).)))))).)))))).((((((((((...))))..))))))................(((((...))))) MEA
.......((((((.((((((.((((....)))).)))))).)))))).((((((((((...))))..))))))................(((((...))))) MFE
.,,....((((((.((((((.((((....)))).)))))).)))))).((((((((((...))))..))))))............,...(((((...))))) EFE

>Cestodes_Taenia_solium_AJ428456.1_sm1
ACCGUUAAUCGGUCCUUACCUUGCAAUUUUGUAUGGUGAGUAUCGAUGCAGCUCGGGCUCUGGCUACGAGCUGACCCAGUAUUUGGCUGGUCCGUCAAGGGC
....xxxxxxxxx................
.......((((((.((((((.((((....)))).)))))).)))))).((((((((((....))).)))))))................((((.....)))) MEA
.......((((((.((((((.((((....)))).)))))).)))))).((((((((((....))).)))))))................((((.....)))) MFE
.......((((((.((((((.((((....)))).)))))).)))))).((((((((((....))).)))))))................((((.....)))) EFE

>Cestodes_Echinococcus_multilocularis_AJ292365.1_sm1
ACCGUUAAUCGGUCCUUACCUUGCAGUUUUGUAUGGUGAGUAUCGAUGCAGCUGAGGCUGUGCCUACGAGCUGACCCAGUAUUUGGCUGGUCCUUCGAGGGC
....xxxxxxxxx................
.......((((((.((((((.((((....)))).)))))).))))))(((((....)))))((((.((((..((((............)))).)))).)))) MEA
.......((((((.((((((.((((....)))).)))))).))))))(((((....)))))((((.((((..((((............)))).)))).)))) MFE
.......((((((.((((((.((((....)))).)))))).))))))(((((....)))))((((.((((..((((............)))).)))).)))) EFE

>Trematoda_Schistosoma_mansoni_rajko.90nt.slRNA-5.1.1_sm1
CCGUCACGGUUUUACUCUUGUGAUUUGUUGCAUGGUAAGAACCGUCGACCAAGAAUCGAAGUUUUCUUUGGCAGCCCUACA
....xxxxxxxxxxxx..........

```
..((((((((((((((..(((((....))))).))))).)))))).)))................................ MEA
..((((((((((((((..(((((....))))).))).)))))))).)))................................ MFE
..((((((((((((({,.(((({....}))))).)))))))))))).)))................................ EFE

>Trematoda_Schistosoma_mansoni_rajko.90nt.slRNA-39.1.1_sm1
CCGUCACGGUUUUACUCUUGUGAUUUGUUGCAUGGUAAGAACCGUCGACCAAGAAUCGAAGUUUUCUUUGGCAGCCCUACA
...........................................................xxxxxxxxxxxx..........
..((((((((((((((..(((((....))))).))))).)))))).)))................................ MEA
..((((((((((((((..(((((....))))).))).)))))))).)))................................ MFE
..((((((((((((({,.(((({....}))))).)))))))))))).)))................................ EFE

>Trematoda_Schistosoma_mansoni_rajko.90nt.slRNA-46.1.1_sm1
CCGUCACGGUUUUACUCUUGUGAUUUGUUGCAUGGUAAGAACCGUCGACCAAGAAUCGAAGUUUUCUUUGGCAGCCCUACA
..........................................................xxxxxxxxxxxx...........
..((((((((((((((..(((((....))))).))))).)))))).)))................................ MEA
..((((((((((((((..(((((....))))).))).)))))))).)))................................ MFE
..((((((((((((({,.(((({....}))))).)))))))))))).)))................................ EFE

>Trematoda_Schistosoma_mansoni_rajko.90nt.slRNA-45.1.1_sm1
CCGUCACGGGUUUUACUCUUGUGAUUUGUUGCAUGGUAAGAACCGUCGACCAAGAAUCGAAGUUUUCUUUGGCAGCCCUAC
............................................................xxxxxxxxxxxx.........
..(((((((.(((((((..(((((....))))).))))))).)))).))).............................. MEA
..(((((((.(((((((..(((((....))))).))))))).)))).))).............................. MFE
..(((((((.{((((({,.(((({....}))))).))))))}.)))).))).............................. EFE

>Trematoda_Schistosoma_mansoni_rajko.90nt.slRNA-31.1.1_sm1
CCGUCACGGUUUUACUCUUGUGAUUUGUUGCAUGGUAAGAACCGUCGACCAAGAAUCGAAGUUUUCUUUGGCAGCCCUACA
...........................................................xxxxxxxxxxxx..........
..((((((((((((((..(((((....))))).))))).)))))).)))................................ MEA
..((((((((((((((..(((((....))))).))).)))))))).)))................................ MFE
..((((((((((((({,.(((({....}))))).)))))))))))).)))................................ EFE
```

```
>Trematoda_Stephanostomum_sp_U83576.1_sm1
CCUAUACGGUUCUCUGCCGUGUAUAUUAGUGCAUGGUAAGAAUCGAAUUCGACCUAUGGUCGAAUAAAUUCUUUGGCUAGCCUCUU
..................................................................xxxxxxxxxx..........
......(((((((.((((((((((....))))))))))))))))).((((((((...)))))))).................... MEA
......(((((((.((((((((((....))))))))))))))))).((((((((...)))))))).................... MFE
......(((((((.((((((((((....))))))))))))))))).((((((((...)))))))).................... EFE

>Trematoda_Haematolechus_sp_U83578.1_sm1
CCUAUACGGUUCUCUGCCGUGUAUCAGUGCAUGGUAAGAAUCGAGUUCGACUCACAUCGUUGGUCGAAUAGAUUAUUUGGCUAGCCUCCA
......................................................................xxxxxxxxxx..........
......(((((((.(((((((((....)))))))))))))))).((((((((.((...)).)))))))).................... MEA
......(((((((.(((((((((....)))))))))))))))).((((((((.((...)).)))))))).................... MFE
......(((((((.(((((((((....)))))))))))))))).((((((((.({...}}.)))))))).................... EFE
```
