# Supplementary material for: Evolution of SL-RNA Genes and Their Splicing Targets in Parasitic Flatworms
Source: Mol Biol Evol. 2025 Sep 23;42(11):msaf228. doi: 10.1093/molbev/msaf228 (PMC12582326; doi:10.1093/molbev/msaf228)
Supplement: msaf228_Supplementary_Data [file msaf228_supplementary_data.zip › Supplementary File 3.pdf]

Supplementary File 3: Description of the secondary structures of the atypical SL-RNA sequences, and case-by-case explanation of why they were included as valid putative SL-RNAs.

- **Outlier1**: Unique_SL-8, Unique_SL-9, Unique_SL-10 and Unique_SL-72

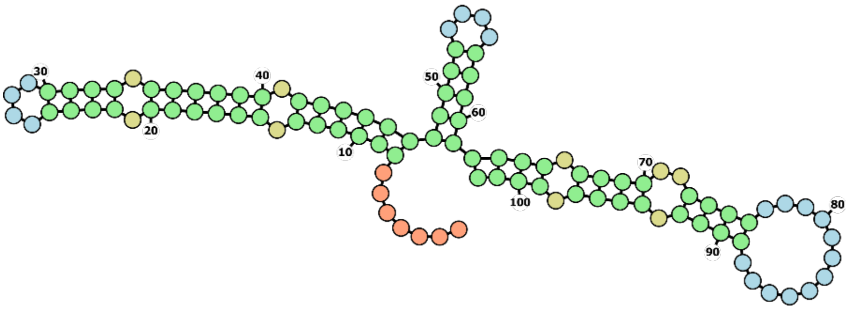

The first expected hairpin is recognizable but the 3’ region joins with parts of the second, leaving the SM-like site in the third hairpin loop. These sequences were selected because they resemble the untrimmed reference sequences AJ292365.1 and AJ292377.1 for *E. multilocularis* and *E. granulosus*, respectively.

- **Outlier2**: Unique_SL-33

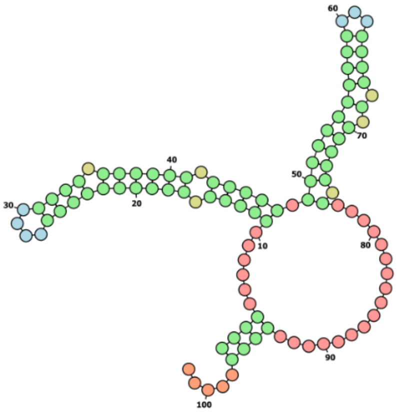

A portion of the terminal regions of the SL joins, closing the structure. This was selected because our tests suggest that this is a common occurrence when the 3’ terminal hairpin is not fully recovered (see **Supplementary File 4**).

- **Outlier3**: Unique_SL-11

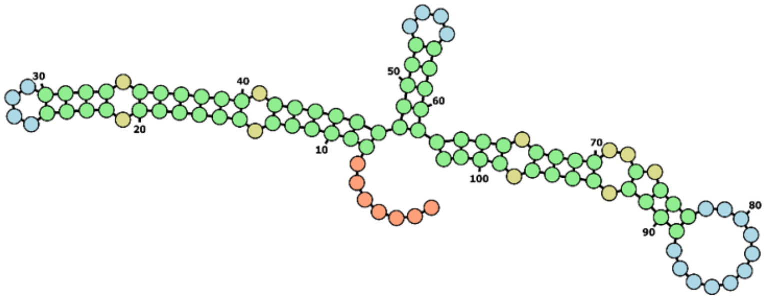

The first expected hairpin is recognizable but the 3’ region joins with parts of the second, leaving the SM-like site in the third hairpin loop. These sequences were selected because they resemble the untrimmed reference sequences AJ292365.1 and AJ292377.1 for *E. multilocularis* and *E. granulosus*, respectively.

- **Outlier4**: Unique_SL-12

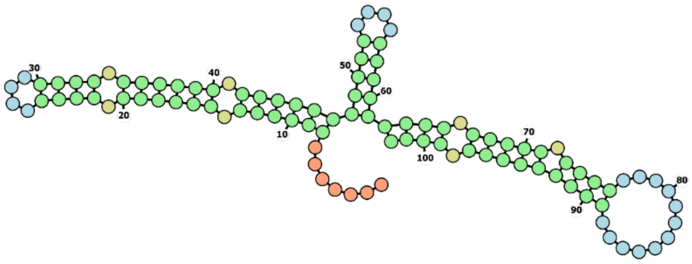

The first expected hairpin is recognizable but the 3’ region joins with parts of the second, leaving the SM-like site in the third hairpin loop. These sequences were selected because they resemble the untrimmed reference sequences AJ292365.1 and AJ292377.1 for *E. multilocularis* and *E. granulosus*, respectively.

- **Outlier5**: Unique_SL-43

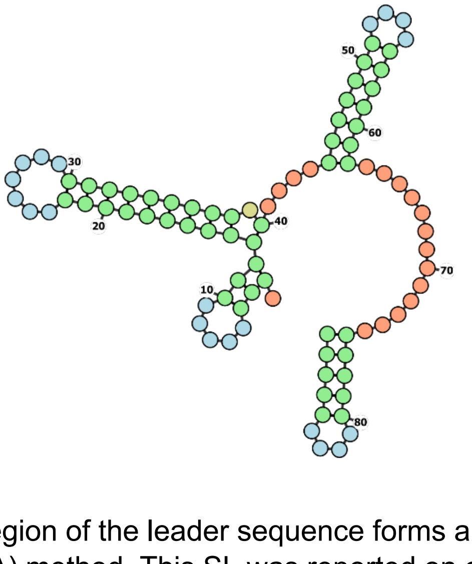

The normally unpaired region of the leader sequence forms a hairpin with the Maximum Expected Accuracy (MEA) method. This SL was reported on our previous work (Calvelo

et al., 2023) and is present on *H. microstoma* transcriptome in approximately 15% of the processed reads, and therefore included in the analysis.

- **Outlier6**: Unique_SL-50

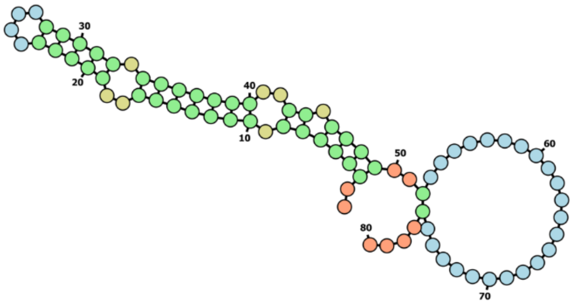

The main hairpin of Schistomidae SLs is present, which is why it was included in the analysis. However, the 3' end shows a weak tendency to form an internal loop around the SM-like site.

- **Outlier7**: Unique_SL-60

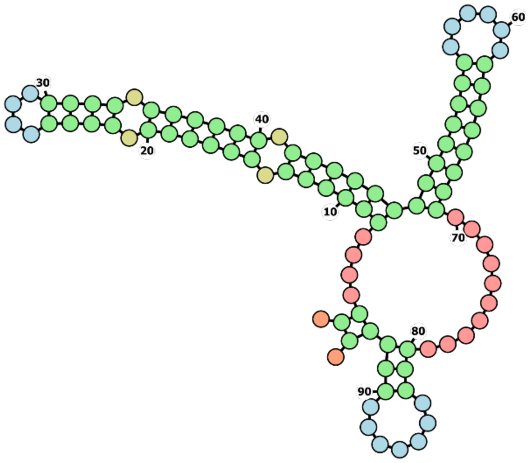

While the 3' hairpin is present in some form, a portion of the terminal regions of the SL joins, closing the structure. This was selected because our tests suggest that this is a common occurrence when the 3' terminal hairpin is not fully recovered (see **Supplementary File 4**).

- **Outlier8:** Unique_SL-61

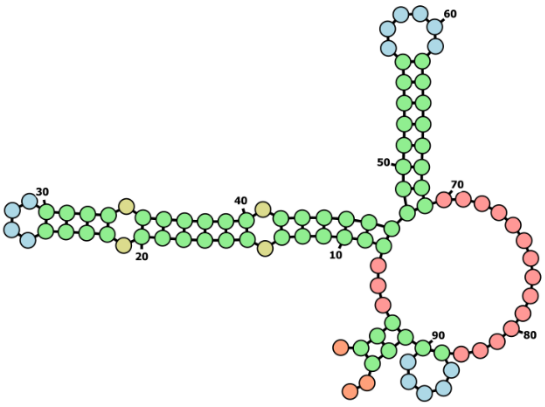

A portion of the terminal regions of the SL joins, closing the structure. This was selected because our tests suggest that this is a common occurrence when the 3' terminal hairpin is not fully recovered (see **Supplementary File 4**).

- **Outlier9**: Unique_SL-64

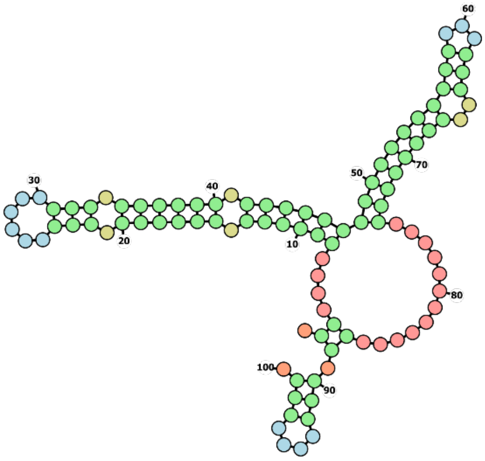

A portion of the terminal regions of the SL joins, closing the structure. This was selected because our tests suggest that this is a common occurrence when the 3' terminal hairpin is not fully recovered (see **Supplementary File 4**).

Calvelo, J., Brehm, K., Iriarte, A., & Koziol, U. (2023). Trans-splicing in the cestode Hymenolepis microstoma is constitutive across the life cycle and depends on gene structure and composition. *International Journal for Parasitology*, *53*(2), 103–117. https://doi.org/10.1016/j.ijpara.2022.11.006
