# Supplementary material for: Evolution of SL-RNA Genes and Their Splicing Targets in Parasitic Flatworms
Source: Mol Biol Evol. 2025 Sep 23;42(11):msaf228. doi: 10.1093/molbev/msaf228 (PMC12582326; doi:10.1093/molbev/msaf228)
Supplement: msaf228_Supplementary_Data [file msaf228_supplementary_data.zip › Supplementary File 4.pdf]

Supplementary File 4: Observations on the sensitivity of trimming in the predicted SL-RNA structures.

Delimiting SL RNA based on sequence conservation is not a straightforward approach, as the structure prediction of several known SLs can vary significantly with the inclusion or removal of even a single base. This is particularly true among the Cestode sequences. To illustrate this effect, we selected three SL RNAs. Each section presents the sequence, followed by the unpaired base constraints (the SM-like site), and the predicted structures based on Maximum Expected Accuracy (MEA), Minimum Free Energy (MFE), and Ensemble Free Energy (EFE). Added bases are highlighted in green, removed bases in red, and the SM-like site, which was constrained as unpaired, is underlined.

1) Addition of 1 base at the 5' of SL-RNA Unique_SL-41, locus Hdiminuta_Def_Loci-7

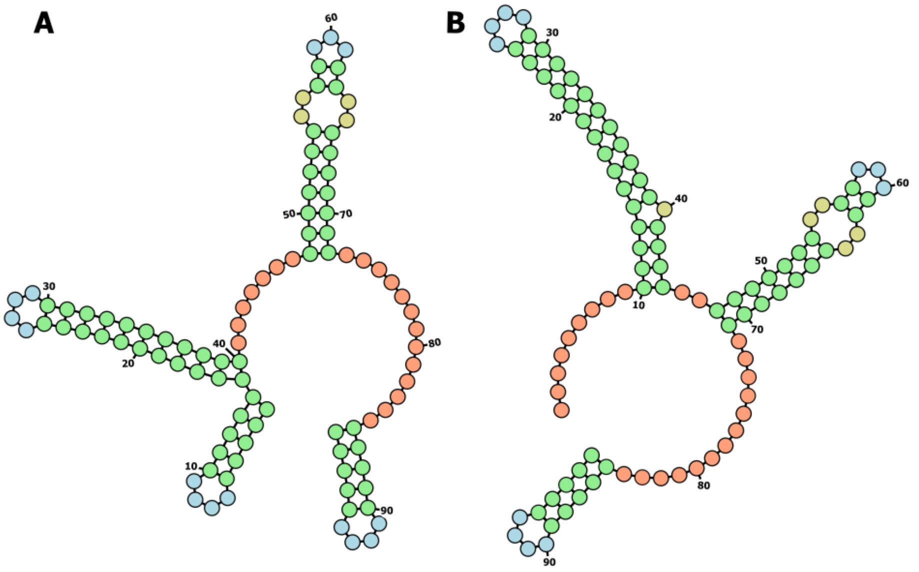

A

```
>Unique_SL_41 -- Hdiminuta_Def_Loci-7 (plus 1 base 5')
ACCGAGUAUUCGGUCUUACUAUACGAACUUGUAUGGUGAGUAUCGUGACAGCUCUCGUUUUGCUUGAGCUGUAGAGUUUUUGGCUGGUCCGCGAGGGCC
(((((....)))))(((((((((((....))))))))))).......(((((((..((...))..))))))).............(((((....))))) MEA
(((((....)))))(((((((((((....))))))))))).......(((((((..((...))..))))))).............(((((....))))) MFE
{((((....))))}(((((((((({....})))))))))).......(((((((..({...})..))))))).............(((((....))))) EFE
```

B

```
>Unique_SL_41
CCGAGUAUUCGGUCUUACUAUACGAACUUGUAUGGUGAGUAUCGUGACAGCUCUCGUUUUGCUUGAGCUGUAGAGUUUUUGGCUGGUCCGCGAGGGCC
.........(((((((((((((((....))))))))))).))))..(((((((..((...))..))))))).............(((((....))))) MEA
.........(((((((((((((((....))))))))))).))))..(((((((..((...))..))))))).............(((((....))))) MFE
,,,,....,|||{(((((((((({....})))))))))).}}}}..(((((((..({...})..))))))).............(((((....))))) EFE
```

This modification induces that portion of the leader into a small hairpin, compromising the first hairpin (B), similar to SL-4 described in our previous work (Calvelo et al., 2023). It matches the Outlier 5 conformation.

2) Addition of 3 base at the 5’ of SL-RNA Unique_SL-8, reference sequence AJ292365.1

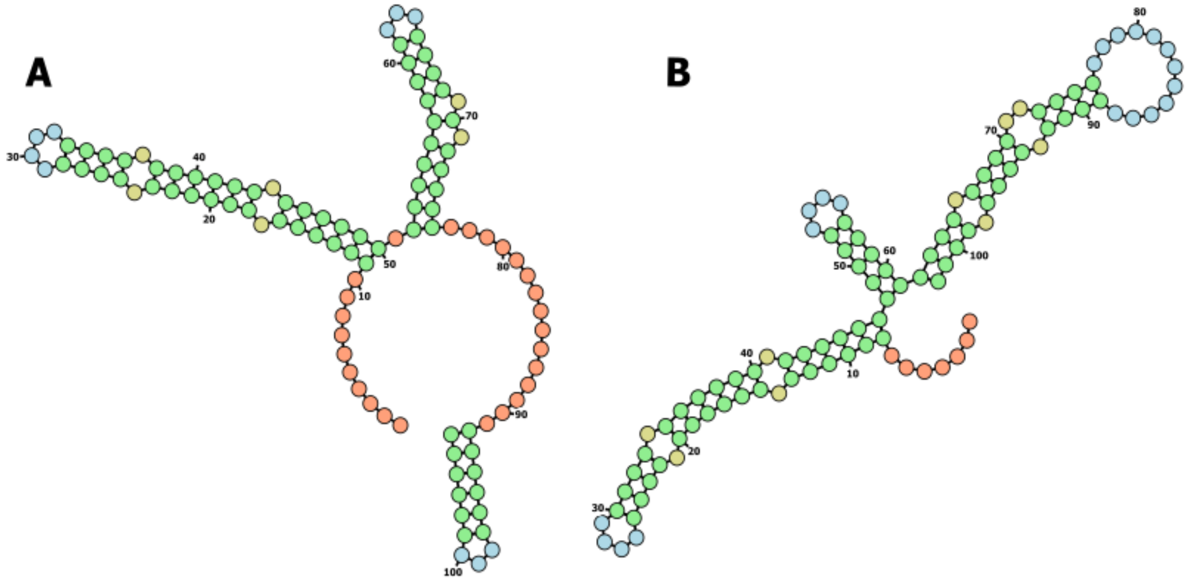


```
A
>Unique_SL_8 -- Reference SL Echinococcus multilocularis AJ292365.1 (plus 3 bases 5')
GUCACCGUUAAUCGGUCCUUACCUUGCAGUUUUGUAUGGUGAGUAUCGAUGCAGCUGAGGCUGUGCCUACGAGCUGACCCAGUAUUUGGCUGGUCCUUCGAGGGCC
..........((((((.((((((.((((....)))).)))))).)))))).((((((((((...)))).).)))))...............((((((...)))))) MEA
..........((((((.((((((.((((....)))).)))))).))))))(((((....)))))((((.((((..((((............)))).)))).)))). MFE
..........((((((.((((((.((((....)))).)))))).)))))),(((({,{{||,,,||||.|{||}}|,{,............||||{|,,,})))))} EFE

B
>Unique_SL_8
ACCGUUAAUCGGUCCUUACCUUGCAGUUUUGUAUGGUGAGUAUCGAUGCAGCUGAGGCUGUGCCUACGAGCUGACCCAGUAUUUGGCUGGUCCUUCGAGGGC
.......((((((.((((((.((((....)))).)))))).))))))(((((....)))))((((.((((..((((............)))).)))).)))) MEA
.......((((((.((((((.((((....)))).)))))).))))))(((((....)))))((((.((((..((((............)))).)))).)))) MFE
.......((((((.((((((.((((....)))).)))))).))))))(((((....)))))((((.((((..((((............)))).)))).)))) EFE
```

The addition of three bases at the 5’ end of Unique_SL-8, as reported in the reference sequence AJ292365.1 (A), restores the structure of Unique_SL-8 (B). However, the MEA and MFE predictions remain contradictory. This is consistent with Outliers 1, 3, and 4.

3) The removal of a single base at the 3' of Unique_SL-44, locus Hmicrostoma_Def_Loci-6.

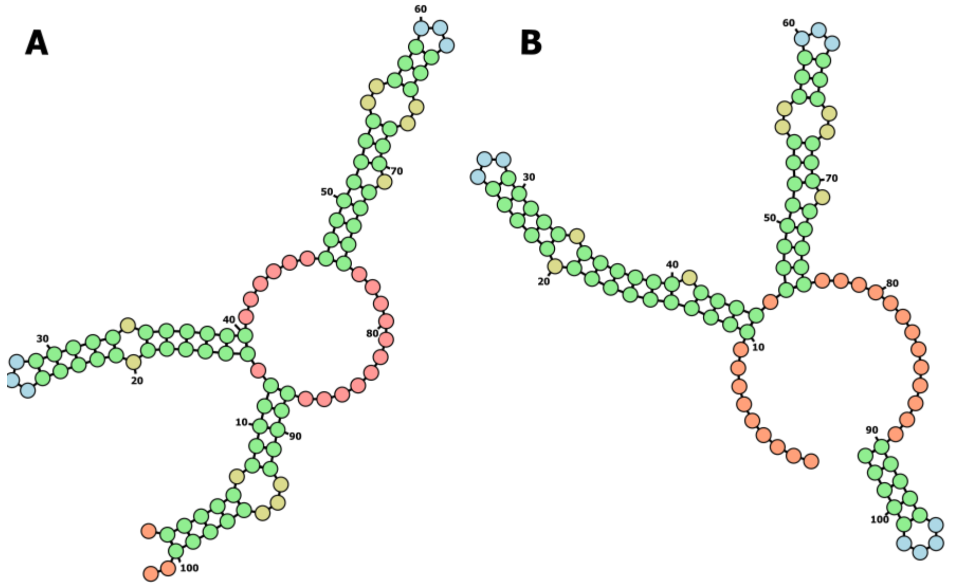

A

>Unique_SL_44 (minus 1 base 3')

ACCGUUUACCGGUCUUACCUUGCAAUUUUUGUAUGGUGAGUAUCGAUACAGCGCUUGGCUUUGCCUUGUGUCUGUAA<u>AAUUCUUUGG</u>CUGGUCCUAACGGGC

.(((((.(((((.((((((.(((((...))))).))))))......((((((((..(((...)))..))).))))))..........)))))...))))).. MEA

.(((((.(((((.((((((.(((((...))))).))))))......((((((((..(((...)))..))).))))))..........)))))...))))).. MFE

.(((((.(((((.((((((.(((({...})))).))))))......((((((((..(((...)))..))).))))))..........)))))...))))).. EFE

B

>Unique_SL_44

ACCGUUUACCGGUCUUACCUUGCAAUUUUUGUAUGGUGAGUAUCGAUACAGCGCUUGGCUUUGCCUUGUGUCUGUAA<u>AAUUCUUUGG</u>CUGGUCCUAACGGGCC

.........((((((((((.(((((...))))).)))))).)))).((((((((..(((...)))..))).)))))............(((((....))))) MEA

.........((((((((((.(((((...))))).)))))).)))).((((((((..(((...)))..))).)))))............(((((....))))) MFE

.........((((((((((.(((({...})))).)))))).)))).((((((((..(((...)))..))).)))))............(((((....))))) EFE

This removal of a single base at the 3' end causes the circularization of the SL-RNA (A) when compared to the original sequence (B), leading to the compromise of the structure prediction. This matches Outliers 2, 7, 8, and 9. And arguably Outlier 6.

References:

Calvelo, J., Brehm, K., Iriarte, A., & Koziol, U. (2023). Trans-splicing in the cestode Hymenolepis microstoma is constitutive across the life cycle and depends on gene structure and composition. *International Journal for Parasitology*, *53*(2), 103–117. https://doi.org/10.1016/j.ijpara.2022.11.006
