# Supplementary material for: Evolution of SL-RNA Genes and Their Splicing Targets in Parasitic Flatworms
Source: Mol Biol Evol. 2025 Sep 23;42(11):msaf228. doi: 10.1093/molbev/msaf228 (PMC12582326; doi:10.1093/molbev/msaf228)
Supplement: msaf228_Supplementary_Data [file msaf228_supplementary_data.zip › Supplementary File 5 - 10032025.pdf]

**Supplementary File 5:** Tesults of the tree reconciliation analysis comparing the SL RNA and species phylogenies (shown in Figure 3) using the ETE package.

| Code | Gene | Species |
|---|---|---|
| Csi.1 | Clonorchis_sinensis_SL_1_Unique_SL-1 | Clonorchis_sinensis |
| Csi.2 | Clonorchis_sinensis_SL_2_Unique_SL-32 | Clonorchis_sinensis |
| Ofe.1 | Opisthorchis_felineus_SL_1_Unique_SL-17 | Opisthorchis_felineus |
| Ssp.1 | Trematoda_Stephanostomum_sp_U83576.1_Reference_SL_Unique_SL-74 | Stephanostomum_sp |
| Phe.1 | Paragonimus_heterotremus_SL_1_Unique_SL-47 | Paragonimus_heterotremus |
| Hsp.1 | Trematoda_Haematolechus_sp_U83578.1_Reference_SL_Unique_SL-73 | Haematolechus_sp |
| Fgi.1 | Fasciola_gigantica_SL_1_Unique_SL-13 | Fasciola_gigantica |
| Fgi.2 | Fasciola_gigantica_SL_2_Unique_SL-14 | Fasciola_gigantica |
| Fhe.1 | Fasciola_hepatica_SL_1_Unique_SL-13 | Fasciola_hepatica |
| Fhe.2 | Fasciola_hepatica_SL_2_Unique_SL-37 | Fasciola_hepatica |
| Fgi.3 | Fasciola_gigantica_SL_3_Unique_SL-35 | Fasciola_gigantica |
| Fgi.4 | Fasciola_gigantica_SL_4_Unique_SL-36 | Fasciola_gigantica |
| Fbu.1 | Fasciolopsis_buski_SL_1_Unique_SL-38 | Fasciolopsis_buski |
| Fbu.2 | Fasciolopsis_buski_SL_2_Unique_SL-39 | Fasciolopsis_buski |
| Sbo.1 | Schistosoma_bovis_SL_1_Unique_SL-18 | Schistosoma_bovis |
| Sha.2 | Schistosoma_haematobium_SL_2_Unique_SL-20 | Schistosoma_haematobium |
| Sma.4 | Schistosoma_mansoni_SL_4_Unique_SL-55 | Schistosoma_mansoni |
| Sma.3 | Schistosoma_mansoni_SL_3_Unique_SL-54 | Schistosoma_mansoni |
| Sma.7 | Schistosoma_mansoni_SL_7_Unique_SL-58 | Schistosoma_mansoni |
| Sbo.2 | Schistosoma_bovis_SL_2_Unique_SL-19 | Schistosoma_bovis |
| Sma.1 | Schistosoma_mansoni_SL_1_Unique_SL-19 | Schistosoma_mansoni |
| Sma.8 | Trematoda_Schistosoma_mansoni_rajko.90nt.slRNA-31.1.1_Reference_SL_Unique_SL-19 | Schistosoma_mansoni |
| Sma.11 | Trematoda_Schistosoma_mansoni_rajko.90nt.slRNA-46.1.1_Reference_SL_Unique_SL-19 | Schistosoma_mansoni |
| Sma.9 | Trematoda_Schistosoma_mansoni_rajko.90nt.slRNA-39.1.1_Reference_SL_Unique_SL-19 | Schistosoma_mansoni |
| Sma.12 | Trematoda_Schistosoma_mansoni_rajko.90nt.slRNA-5.1.1_Reference_SL_Unique_SL-19 | Schistosoma_mansoni |
| Sha.1 | Schistosoma_haematobium_SL_1_Unique_SL-19 | Schistosoma_haematobium |
| Sbo.3 | Schistosoma_bovis_SL_3_Unique_SL-48 | Schistosoma_bovis |
| Sbo.4 | Schistosoma_bovis_SL_4_Unique_SL-49 | Schistosoma_bovis |
| Sma.10 | Trematoda_Schistosoma_mansoni_rajko.90nt.slRNA-45.1.1_Reference_SL_Unique_SL-75 | Schistosoma_mansoni |
| Sha.3 | Schistosoma_haematobium_SL_3_Unique_SL-50 | Schistosoma_haematobium |
| Sja.2 | Schistosoma_japonicum_SL_2_Unique_SL-22 | Schistosoma_japonicum |
| Sja.1 | Schistosoma_japonicum_SL_1_Unique_SL-21 | Schistosoma_japonicum |
| Sma.6 | Schistosoma_mansoni_SL_6_Unique_SL-57 | Schistosoma_mansoni |
| Sma.13 | Trematoda_Schistosoma_mansoni_Smp_rajko_90nt_slRNA-1.1.1_Reference_SL_Unique_SL-57 | Schistosoma_mansoni |
| Sja.3 | Schistosoma_japonicum_SL_3_Unique_SL-23 | Schistosoma_japonicum |
| Sja.4 | Schistosoma_japonicum_SL_4_Unique_SL-24 | Schistosoma_japonicum |
| Sja.6 | Schistosoma_japonicum_SL_6_Unique_SL-52 | Schistosoma_japonicum |
| Sja.7 | Schistosoma_japonicum_SL_7_Unique_SL-53 | Schistosoma_japonicum |
| Sja.5 | Schistosoma_japonicum_SL_5_Unique_SL-51 | Schistosoma_japonicum |
| Sma.2 | Schistosoma_mansoni_SL_2_Unique_SL-25 | Schistosoma_mansoni |
| Sma.5 | Schistosoma_mansoni_SL_5_Unique_SL-56 | Schistosoma_mansoni |
| Tre.5 | Trichobilharzia_regenti_SL_5_Unique_SL-69 | Trichobilharzia_regenti |
| Tre.7 | Trichobilharzia_regenti_SL_7_Unique_SL-71 | Trichobilharzia_regenti |
| Tre.1 | Trichobilharzia_regenti_SL_1_Unique_SL-65 | Trichobilharzia_regenti |
| Tre.4 | Trichobilharzia_regenti_SL_4_Unique_SL-68 | Trichobilharzia_regenti |
| Tre.2 | Trichobilharzia_regenti_SL_2_Unique_SL-66 | Trichobilharzia_regenti |
| Tre.6 | Trichobilharzia_regenti_SL_6_Unique_SL-70 | Trichobilharzia_regenti |
| Tre.3 | Trichobilharzia_regenti_SL_3_Unique_SL-67 | Trichobilharzia_regenti |
| Tas.2 | Taenia_asiatica_SL_2_Unique_SL-60 | Taenia_asiatica |
| Tsa.2 | Taenia_saginata_SL_2_Unique_SL-60 | Taenia_saginata |
| Tmu.4 | Taenia_multiceps_SL_4_Unique_SL-61 | Taenia_multiceps |
| Tso.3 | Taenia_solium_SL_2_Unique_SL-64 | Taenia_solium |
| Egr.2 | Echinococcus_granulosus_SL_1_Unique_SL-33 | Echinococcus_granulosus |
| Emu.12 | Echinococcus_multilocularis_SL_4_Unique_SL-2 | Echinococcus_multilocularis |
| Emu.13 | Echinococcus_multilocularis_SL_5_Unique_SL-3 | Echinococcus_multilocularis |
| Emu.4 | Cestodes_Echinococcus_multilocularis_Emu.SL2b_Reference_SL_Unique_SL-3 | Echinococcus_multilocularis |
| Emu.15 | Echinococcus_multilocularis_SL_7_Unique_SL-5 | Echinococcus_multilocularis |
| Emu.17 | Echinococcus_multilocularis_SL_9_Unique_SL-7 | Echinococcus_multilocularis |
| Emu.3 | Cestodes_Echinococcus_multilocularis_Emu.SL2a_Reference_SL_Unique_SL-7 | Echinococcus_multilocularis |
| Emu.14 | Echinococcus_multilocularis_SL_6_Unique_SL-4 | Echinococcus_multilocularis |
| Emu.5 | Cestodes_Echinococcus_multilocularis_Emu.SL2c_Reference_SL_Unique_SL-4 | Echinococcus_multilocularis |
| Emu.16 | Echinococcus_multilocularis_SL_8_Unique_SL-6 | Echinococcus_multilocularis |
| Emu.6 | Cestodes_Echinococcus_multilocularis_Emu.SL2d_Reference_SL_Unique_SL-6 | Echinococcus_multilocularis |
| Hdi.1 | Hymenolepis_diminuta_SL_1_Unique_SL-15 | Hymenolepis_diminuta |
| Hmi.1 | Hymenolepis_microstoma_SL_1_Unique_SL-16 | Hymenolepis_microstoma |
| Hmi.3 | Hymenolepis_microstoma_SL_3_Unique_SL-43 | Hymenolepis_microstoma |
| Mco.1 | Mesocestoides_corti_SL_1_Unique_SL-45 | Mesocestoides_corti |
| Spr.2 | Sparganum_proliferum_SL_2_Unique_SL-27 | Sparganum_proliferum |
| Ser.2 | Spirometra_erinaceieuropaei_SL_2_Unique_SL-27 | Spirometra_erinaceieuropaei |
| Ser.3 | Spirometra_erinaceieuropaei_SL_3_Unique_SL-59 | Spirometra_erinaceieuropaei |
| Ser.1 | Spirometra_erinaceieuropaei_SL_1_Unique_SL-26 | Spirometra_erinaceieuropaei |
| Spr.1 | Sparganum_proliferum_SL_1_Unique_SL-26 | Sparganum_proliferum |
| Spr.3 | Sparganum_proliferum_SL_3_Unique_SL-26 | Sparganum_proliferum |
| Mco.2 | Mesocestoides_corti_SL_2_Unique_SL-46 | Mesocestoides_corti |
| Hdi.3 | Hymenolepis_diminuta_SL_3_Unique_SL-41 | Hymenolepis_diminuta |
| Hmi.2 | Hymenolepis_microstoma_SL_2_Unique_SL-42 | Hymenolepis_microstoma |
| Tsa.3 | Taenia_saginata_SL_3_Unique_SL-62 | Taenia_saginata |
| Tso.2 | Taenia_solium_SL_1_Unique_SL-63 | Taenia_solium |
| Hdi.2 | Hymenolepis_diminuta_SL_2_Unique_SL-40 | Hymenolepis_diminuta |
| Hmi.4 | Hymenolepis_microstoma_SL_4_Unique_SL-44 | Hymenolepis_microstoma |
| Egr.3 | Echinococcus_granulosus_SL_2_Unique_SL-34 | Echinococcus_granulosus |
| Tas.1 | Taenia_asiatica_SL_1_Unique_SL-28 | Taenia_asiatica |
| Tso.1 | Cestodes_Taenia_solium_AJ428456.1_Reference_SL_Unique_SL-28 | Taenia_solium |
| Tmu.2 | Taenia_multiceps_SL_2_Unique_SL-29 | Taenia_multiceps |

```
Tmu.1     Taenia_multiceps_SL_1_Unique_SL-28      Taenia_multiceps
Tmu.3     Taenia_multiceps_SL_3_Unique_SL-30      Taenia_multiceps
Tsa.1     Taenia_saginata_SL_1_Unique_SL-31       Taenia_saginata
Egr.1     Cestodes_Echinococcus_granulosus_AJ292377.1_Reference_SL_Unique_SL-72  Echinococcus_granulosus
Emu.10    Echinococcus_multilocularis_SL_2_Unique_SL-11     Echinococcus_multilocularis
Emu.11    Echinococcus_multilocularis_SL_3_Unique_SL-12     Echinococcus_multilocularis
Emu.8     Echinococcus_multilocularis_SL_10_Unique_SL-8     Echinococcus_multilocularis
Emu.1     Cestodes_Echinococcus_multilocularis_AJ292365.1_Reference_SL_Unique_SL-8        Echinococcus_multilocularis
Emu.7     Echinococcus_multilocularis_SL_1_Unique_SL-10     Echinococcus_multilocularis
Emu.2     Cestodes_Echinococcus_multilocularis_Emu.SL1_Reference_SL_Unique_SL-10 Echinococcus_multilocularis
Emu.9     Echinococcus_multilocularis_SL_11_Unique_SL-9     Echinococcus_multilocularis
```

_ _ _ _ _ _ _ _ _ _ _ _ _

Rooted tree:

_ _ _ _ _ _ _ _ _ _ _ _ _

```
                     /-Csi.1
                  /-|
                 |  |   /-Csi.2
               /-|   \-|
              |  |      \-Ofe.1
              |  |
            /-|   \-Ssp.1
           |  |
           |  |   /-Phe.1
           |   \-|
           |      \-Hsp.1
           |
           |               /-Fgi.1
         /-|            /-|
        |  |           |  |   /-Fgi.2
        |  |         /-|   \-|
        |  |        |  |      \-Fhe.1
        |  |      /-|  |
        |  |     |  |   \-Fhe.2
      /-|  |   /-|  |
     |  |  |  |  |   \-Fgi.3
     |  |   \-|  |
     |  |     |   \-Fgi.4
     |  |     |
     |  |      \-Fbu.1
     |  |
     |   \-Fbu.2
     |
     |                                 /-Sbo.1
     |                              /-|
     |                           /-|   \-Sha.2
     |                          |  |
     |                        /-|   \-Sma.4
     |                       |  |
     |                       |  |   /-Sma.3
     |                       |   \-|
     |                       |      \-Sma.7
     |                       |
     |                       |                        /-Sbo.2
     |                       |                     /-|
     |                       |                  /-|   \-Sma.1
     |                       |                 |  |
     |                       |               /-|   \-Sma.8
     |                       |              |  |
     |                       |            /-|   \-Sma.11
     |                     /-|           |  |
     |                    |  |         /-|   \-Sma.9
     |                    |  |        |  |
     |                    |  |      /-|   \-Sma.12
     |                    |  |     |  |
     |                    |  |     |   \-Sha.1
     |                    |  |   /-|
     |                    |  |  |  |      /-Sbo.3
     |                    |  |  |  |   /-|
   /-|                    |  |  |   \-|   \-Sbo.4
  |  |                    |  |  |     |
  |  |                    |  |  |      \-Sma.10
  |  |                    |  |  |
  |  |                    |  |  |         /-Sha.3
  |  |                    |   \-|      /-|
  |  |                    |     |     |   \-Sja.2
  |  |                  /-|     |   /-|
  |  |                 |  |     |  |  |   /-Sja.1
  |  |                 |  |     |  |   \-|
  |  |                 |  |     |  |     |   /-Sma.6
  |  |                 |  |     |  |      \-|
```

```
  |  |                 |  |     |  |         \-Sma.13
  |  |                 |  |      \-|
  |  |                 |  |        |         /-Sja.3
  |  |                 |  |        |      /-|
  |  |               /-|  |        |     |   \-Sja.4
  |  |              |  |  |        |   /-|
  |  |              |  |  |        |  |  |   /-Sja.6
  |  |              |  |  |         \-|   \-|
  |  |              |  |  |           |      \-Sja.7
  |  |              |  |  |           |
  |  |            /-|  |  |            \-Sja.5
  |  |           |  |  |  |
  |  |           |  |  |   \-Sma.2
  |  |           |  |  |
  |  |           |  |   \-Sma.5
  |  |         /-|  |
  |  |        |  |  |   /-Tre.5
  |  |        |  |   \-|
  |  |      /-|  |      \-Tre.7
  |  |     |  |  |
  |  |     |  |   \-Tre.1
--|  |   /-|  |
  |  |  |  |   \-Tre.4
  |  |  |  |
  |   \-|  |   /-Tre.2
  |     |   \-|
  |     |      \-Tre.6
  |     |
  |      \-Tre.3
  |
  |         /-Tas.2
  |      /-|
  |   /-|   \-Tsa.2
  |  |  |
  |  |   \-Tmu.4
  |  |
  |  |   /-Tso.3
  |  |  |
  |  |  |                              /-Egr.2
  |  |  |                             |
  |  |  |                             |         /-Emu.12
  |  |  |                             |        |
  |  |  |                             |        |         /-Emu.13
  |  |  |                             |      /-|      /-|
  |  |  |                             |     |  |   /-|   \-Emu.4
  |  |  |                             |     |  |  |  |
  |  |  |                             |     |   \-|   \-Emu.15
  |  |  |                             |     |     |
  |  |  |                             |     |     |   /-Emu.17
  |  |  |                             |   /-|      \-|
  |  |  |                             |  |  |         \-Emu.3
  |  |  |                             |  |  |
  |  |  |                           /-|  |  |      /-Emu.14
  |  |  |                          |  |  |  |   /-|
   \-|  |                          |  |  |  |  |   \-Emu.5
     |  |                          |  |  |   \-|
     |  |                          |  |  |     |   /-Emu.16
     |  |                          |  |  |      \-|
     |  |                          |  |  |         \-Emu.6
     |  |                          |  |  |
     |  |                          |  |  |            /-Hdi.1
     |  |                          |  |  |         /-|
     |  |                          |  |  |        |  |   /-Hmi.1
     |  |                          |  |  |        |   \-|
     |  |                          |  |  |        |      \-Hmi.3
     |  |                          |  |  |        |
     |  |                          |   \-|        |      /-Mco.1
     |  |                          |     |      /-|     |
     |  |                          |     |     |  |     |         /-Spr.2
     |  |                          |     |     |  |   /-|      /-|
     |  |                          |     |     |  |  |  |   /-|   \-Ser.2
     |  |                          |     |     |  |  |  |  |  |
     |  |                          |     |     |  |  |   \-|   \-Ser.3
     |  |                        /-|     |     |  |  |     |
     |  |                       |  |     |     |   \-|     |   /-Ser.1
     |  |                       |  |     |     |     |      \-|
     |  |                       |  |     |   /-|     |        |   /-Spr.1
      \-|                       |  |     |  |  |     |         \-|
        |                       |  |     |  |  |     |            \-Spr.3
        |                       |  |     |  |  |     |
        |                       |  |     |  |  |      \-Mco.2
        |                       |  |     |  |  |
        |                       |  |     |  |  |      /-Hdi.3
        |                       |  |      \-|  |   /-|
        |                       |  |        |  |  |   \-Hmi.2
```

```
|                       |  |        |   \-|
|                       |  |        |     |   /-Tsa.3
|                       |  |        |      \-|
|                     /-|  |        |         \-Tso.2
|                    |  |  |        |
|                    |  |  |        |   /-Hdi.2
|                    |  |  |         \-|
|                    |  |  |            \-Hmi.4
|                    |  |  |
|                    |  |   \-Egr.3
|                    |  |
|                    |  |               /-Tas.1
|                    |  |            /-|
|                  /-|  |         /-|   \-Tso.1
|                 |  |  |        |  |
|                 |  |  |      /-|   \-Tmu.2
|                 |  |  |     |  |
|                 |  |  |   /-|   \-Tmu.1
|                 |  |  |  |  |
|               /-|  |   \-|   \-Tmu.3
|              |  |  |     |
|              |  |  |      \-Tsa.1
|              |  |  |
|            /-|  |   \-Egr.1
|           |  |  |
|           |  |   \-Emu.10
|         /-|  |
|        |  |   \-Emu.11
|      /-|  |
|     |  |   \-Emu.8
|     |  |
|   /-|   \-Emu.1
|  |  |
|  |  |   /-Emu.7
 \-|   \-|
   |      \-Emu.2
   |
    \-Emu.9
```

— — — — — — — — — — — — —

Tree reconciliation (TR) algorithm:

— — — — — — — — — — — — —

(Intraspecific paralogs are indicated by underlined text, while interspecific paralogs within a genus are shown in bold)

ORTHOLOGY RELATIONSHIP: Csi.2 <====> Ofe.1

PARALOGY RELATIONSHIP: Csi.1 <====> Csi.2,Ofe.1

ORTHOLOGY RELATIONSHIP: Csi.1,Csi.2,Ofe.1 <====> Ssp.1

ORTHOLOGY RELATIONSHIP: Phe.1 <====> Hsp.1

PARALOGY RELATIONSHIP: Csi.1,Csi.2,Ofe.1,Ssp.1 <====> Phe.1,Hsp.1

ORTHOLOGY RELATIONSHIP: Fgi.2 <====> Fhe.1

**PARALOGY RELATIONSHIP: Fgi.1 <====> Fgi.2,Fhe.1**

**PARALOGY RELATIONSHIP: Fgi.1,Fgi.2,Fhe.1 <====> Fhe.2**

**PARALOGY RELATIONSHIP: Fgi.1,Fgi.2,Fhe.1,Fhe.2 <====> Fgi.3**

**PARALOGY RELATIONSHIP: Fgi.1,Fgi.2,Fhe.1,Fhe.2,Fgi.3 <====> Fgi.4**

ORTHOLOGY RELATIONSHIP: Fgi.1,Fgi.2,Fhe.1,Fhe.2,Fgi.3,Fgi.4 <====> Fbu.1

ORTHOLOGY RELATIONSHIP: Csi.1,Csi.2,Ofe.1,Ssp.1,Phe.1,Hsp.1 <====> Fgi.1,Fgi.2,Fhe.1,Fhe.2,Fgi.3,Fgi.4,Fbu.1

PARALOGY RELATIONSHIP: Csi.1,Csi.2,Ofe.1,Ssp.1,Phe.1,Hsp.1,Fgi.1,Fgi.2,Fhe.1,Fhe.2,Fgi.3,Fgi.4,Fbu.1 <====> Fbu.2

ORTHOLOGY RELATIONSHIP: Sbo.1 <====> Sha.2

ORTHOLOGY RELATIONSHIP: Sbo.1,Sha.2 <====> Sma.4

PARALOGY RELATIONSHIP: Sma.3 <====> Sma.7

**PARALOGY RELATIONSHIP: Sbo.1,Sha.2,Sma.4 <====> Sma.3,Sma.7**

ORTHOLOGY RELATIONSHIP: Sbo.2 <====> Sma.1

**PARALOGY RELATIONSHIP: Sbo.2,Sma.1 <====> Sma.8**

**PARALOGY RELATIONSHIP: Sbo.2,Sma.1,Sma.8 <====> Sma.11**

**PARALOGY RELATIONSHIP: Sbo.2,Sma.1,Sma.8,Sma.11 <====> Sma.9**

**PARALOGY RELATIONSHIP: Sbo.2,Sma.1,Sma.8,Sma.11,Sma.9 <====> Sma.12**

**PARALOGY RELATIONSHIP: Sbo.2,Sma.1,Sma.8,Sma.11,Sma.9,Sma.12 <====> Sha.1**

PARALOGY RELATIONSHIP: Sbo.3 <====> Sbo.4

ORTHOLOGY RELATIONSHIP: Sbo.3,Sbo.4 <====> Sma.10

**PARALOGY RELATIONSHIP: Sbo.2,Sma.1,Sma.8,Sma.11,Sma.9,Sma.12,Sha.1 <====> Sbo.3,Sbo.4,Sma.10**

ORTHOLOGY RELATIONSHIP: Sha.3 <====> Sja.2

PARALOGY RELATIONSHIP: Sma.6 <====> Sma.13

ORTHOLOGY RELATIONSHIP: Sja.1 <====> Sma.6,Sma.13

**PARALOGY RELATIONSHIP: Sha.3,Sja.2 <====> Sja.1,Sma.6,Sma.13**

PARALOGY RELATIONSHIP: Sja.3 <====> Sja.4

PARALOGY RELATIONSHIP: Sja.6 <====> Sja.7

PARALOGY RELATIONSHIP: Sja.3,Sja.4 <====> Sja.6,Sja.7

PARALOGY RELATIONSHIP: Sja.3,Sja.4,Sja.6,Sja.7 <====> Sja.5

**PARALOGY RELATIONSHIP: Sha.3,Sja.2,Sja.1,Sma.6,Sma.13 <====> Sja.3,Sja.4,Sja.6,Sja.7,Sja.5**

**PARALOGY RELATIONSHIP: Sbo.2,Sma.1,Sma.8,Sma.11,Sma.9,Sma.12,Sha.1,Sbo.3,Sbo.4,Sma.10 <====> Sha.3,Sja.2,Sja.1,Sma.6,Sma.13,Sja.3,Sja.4,Sja.6,Sja.7,Sja.5**

**PARALOGY RELATIONSHIP: Sbo.1,Sha.2,Sma.4,Sma.3,Sma.7 <====> Sbo.2,Sma.1,Sma.8,Sma.11,Sma.9,Sma.12,Sha.1,Sbo.3,Sbo.4,Sma.10,Sha.3,Sja.2,Sja.1,Sma.6,Sma.13,Sja.3,Sja.4,Sja.6,Sja.7,Sja.5**

**PARALOGY RELATIONSHIP: Sbo.1,Sha.2,Sma.4,Sma.3,Sma.7,Sbo.2,Sma.1,Sma.8,Sma.11,Sma.9,Sma.12,Sha.1,Sbo.3,Sbo.4,Sma.10,Sha.3,Sja.2,Sja.1,Sma.6,Sma.13,Sja.3,Sja.4,Sja.6,Sja.7,Sja.5 <====> Sma.2**

**PARALOGY RELATIONSHIP: Sbo.1,Sha.2,Sma.4,Sma.3,Sma.7,Sbo.2,Sma.1,Sma.8,Sma.11,Sma.9,Sma.12,Sha.1,Sbo.3,Sbo.4,Sma.10,Sha.3,Sja.2,Sja.1,Sma.6,Sma.13,Sja.3,Sja.4,Sja.6,Sja.7,Sja.5,Sma.2 <====> Sma.5**

PARALOGY RELATIONSHIP: Tre.5 <====> Tre.7

ORTHOLOGY RELATIONSHIP: Sbo.1,Sha.2,Sma.4,Sma.3,Sma.7,Sbo.2,Sma.1,Sma.8,Sma.11,Sma.9,Sma.12,Sha.1,Sbo.3,Sbo.4,Sma.10,Sha.3,Sja.2,Sja.1,Sma.6,Sma.13,Sja.3,Sja.4,Sja.6,Sja.7,Sja.5,Sma.2,Sma.5 <====> Tre.5,Tre.7

PARALOGY RELATIONSHIP: Sbo.1,Sha.2,Sma.4,Sma.3,Sma.7,Sbo.2,Sma.1,Sma.8,Sma.11,Sma.9,Sma.12,Sha.1,Sbo.3,Sbo.4,Sma.10,Sha.3,Sja.2,Sja.1,Sma.6,Sma.13,Sja.3,Sja.4,Sja.6,Sja.7,Sja.5,Sma.2,Sma.5,Tre.5,Tre.7 <====> Tre.1

PARALOGY RELATIONSHIP: Sbo.1,Sha.2,Sma.4,Sma.3,Sma.7,Sbo.2,Sma.1,Sma.8,Sma.11,Sma.9,Sma.12,Sha.1,Sbo.3,Sbo.4,Sma.10,Sha.3,Sja.2,Sja.1,Sma.6,Sma.13,Sja.3,Sja.4,Sja.6,Sja.7,Sja.5,Sma.2,Sma.5,Tre.5,Tre.7,Tre.1 <====> Tre.4

PARALOGY RELATIONSHIP: Tre.2 <====> Tre.6

PARALOGY RELATIONSHIP: Sbo.1,Sha.2,Sma.4,Sma.3,Sma.7,Sbo.2,Sma.1,Sma.8,Sma.11,Sma.9,Sma.12,Sha.1,Sbo.3,Sbo.4,Sma.10,Sha.3,Sja.2,Sja.1,Sma.6,Sma.13,Sja.3,Sja.4,Sja.6,Sja.7,Sja.5,Sma.2,Sma.5,Tre.5,Tre.7,Tre.1,Tre.4 <====> Tre.2,Tre.6

PARALOGY RELATIONSHIP: Sbo.1,Sha.2,Sma.4,Sma.3,Sma.7,Sbo.2,Sma.1,Sma.8,Sma.11,Sma.9,Sma.12,Sha.1,Sbo.3,Sbo.4,Sma.10,Sha.3,Sja.2,Sja.1,Sma.6,Sma.13,Sja.3,Sja.4,Sja.6,Sja.7,Sja.5,Sma.2,Sma.5,Tre.5,Tre.7,Tre.1,Tre.4,Tre.2,Tre.6 <====> Tre.3

ORTHOLOGY RELATIONSHIP: Csi.1,Csi.2,Ofe.1,Ssp.1,Phe.1,Hsp.1,Fgi.1,Fgi.2,Fhe.1,Fhe.2,Fgi.3,Fgi.4,Fbu.1,Fbu.2 <====> Sbo.1,Sha.2,Sma.4,Sma.3,Sma.7,Sbo.2,Sma.1,Sma.8,Sma.11,Sma.9,Sma.12,Sha.1,Sbo.3,Sbo.4,Sma.10,Sha.3,Sja.2,Sja.1,Sma.6,Sma.13,Sja.3,Sja.4,Sja.6,Sja.7,Sja.5,Sma.2,Sma.5,Tre.5,Tre.7,Tre.1,Tre.4,Tre.2,Tre.6,Tre.3

ORTHOLOGY RELATIONSHIP: Tas.2 <====> Tsa.2

ORTHOLOGY RELATIONSHIP: Tas.2,Tsa.2 <====> Tmu.4

PARALOGY RELATIONSHIP: Emu.13 <====> Emu.4

PARALOGY RELATIONSHIP: Emu.13,Emu.4 <====> Emu.15

PARALOGY RELATIONSHIP: Emu.17 <====> Emu.3

PARALOGY RELATIONSHIP: Emu.13,Emu.4,Emu.15 <====> Emu.17,Emu.3

PARALOGY RELATIONSHIP: Emu.12 <====> Emu.13,Emu.4,Emu.15,Emu.17,Emu.3

PARALOGY RELATIONSHIP: Emu.14 <====> Emu.5

PARALOGY RELATIONSHIP: Emu.16 <====> Emu.6

PARALOGY RELATIONSHIP: Emu.14,Emu.5 <====> Emu.16,Emu.6

PARALOGY RELATIONSHIP: Emu.12,Emu.13,Emu.4,Emu.15,Emu.17,Emu.3 <====> Emu.14,Emu.5,Emu.16,Emu.6

PARALOGY RELATIONSHIP: Hmi.1 <====> Hmi.3

ORTHOLOGY RELATIONSHIP: Hdi.1 <====> Hmi.1,Hmi.3

ORTHOLOGY RELATIONSHIP: Spr.2 <====> Ser.2

PARALOGY RELATIONSHIP: Spr.2,Ser.2 <====> Ser.3

PARALOGY RELATIONSHIP: Spr.1 <====> Spr.3

ORTHOLOGY RELATIONSHIP: Ser.1 <====> Spr.1,Spr.3

PARALOGY RELATIONSHIP: Spr.2,Ser.2,Ser.3 <====> Ser.1,Spr.1,Spr.3

ORTHOLOGY RELATIONSHIP: Mco.1 <====> Spr.2,Ser.2,Ser.3,Ser.1,Spr.1,Spr.3

PARALOGY RELATIONSHIP: Mco.1,Spr.2,Ser.2,Ser.3,Ser.1,Spr.1,Spr.3 <====> Mco.2

PARALOGY RELATIONSHIP: Hdi.1,Hmi.1,Hmi.3 <====> Mco.1,Spr.2,Ser.2,Ser.3,Ser.1,Spr.1,Spr.3,Mco.2

ORTHOLOGY RELATIONSHIP: Hdi.3 <====> Hmi.2

ORTHOLOGY RELATIONSHIP: Tsa.3 <====> Tso.2

ORTHOLOGY RELATIONSHIP: Hdi.3,Hmi.2 <====> Tsa.3,Tso.2

PARALOGY RELATIONSHIP: Hdi.1,Hmi.1,Hmi.3,Mco.1,Spr.2,Ser.2,Ser.3,Ser.1,Spr.1,Spr.3,Mco.2 <====> Hdi.3,Hmi.2,Tsa.3,Tso.2

ORTHOLOGY RELATIONSHIP: Hdi.2 <====> Hmi.4

PARALOGY RELATIONSHIP: Hdi.1,Hmi.1,Hmi.3,Mco.1,Spr.2,Ser.2,Ser.3,Ser.1,Spr.1,Spr.3,Mco.2,Hdi.3,Hmi.2,Tsa.3,Tso.2 <====> Hdi.2,Hmi.4

PARALOGY RELATIONSHIP: Emu.12,Emu.13,Emu.4,Emu.15,Emu.17,Emu.3,Emu.14,Emu.5,Emu.16,Emu.6 <====> Hdi.1,Hmi.1,Hmi.3,Mco.1,Spr.2,Ser.2,Ser.3,Ser.1,Spr.1,Spr.3,Mco.2,Hdi.3,Hmi.2,Tsa.3,Tso.2,Hdi.2,Hmi.4

PARALOGY RELATIONSHIP: Egr.2 <====> Emu.12,Emu.13,Emu.4,Emu.15,Emu.17,Emu.3,Emu.14,Emu.5,Emu.16,Emu.6,Hdi.1,Hmi.1,Hmi.3,Mco.1,Spr.2,Ser.2,Ser.3,Ser.1,Spr.1,Spr.3,Mco.2,Hdi.3,Hmi.2,Tsa.3,Tso.2,Hdi.2,Hmi.4

PARALOGY RELATIONSHIP: Egr.2,Emu.12,Emu.13,Emu.4,Emu.15,Emu.17,Emu.3,Emu.14,Emu.5,Emu.16,Emu.6,Hdi.1,Hmi.1,Hmi.3,Mco.1,Spr.2,Ser.2,Ser.3,Ser.1,Spr.1,Spr.3,Mco.2,Hdi.3,Hmi.2,Tsa.3,Tso.2,Hdi.2,Hmi.4 <====> Egr.3

ORTHOLOGY RELATIONSHIP: Tas.1 <====> Tso.1

**PARALOGY RELATIONSHIP: Tas.1,Tso.1 <====> Tmu.2**

**PARALOGY RELATIONSHIP: Tas.1,Tso.1,Tmu.2 <====> Tmu.1**

**PARALOGY RELATIONSHIP: Tas.1,Tso.1,Tmu.2,Tmu.1 <====> Tmu.3**

**PARALOGY RELATIONSHIP: Tas.1,Tso.1,Tmu.2,Tmu.1,Tmu.3 <====> Tsa.1**

PARALOGY RELATIONSHIP: Egr.2,Emu.12,Emu.13,Emu.4,Emu.15,Emu.17,Emu.3,Emu.14,Emu.5,Emu.16,Emu.6,Hdi.1,Hmi.1,Hmi.3,Mco.1,Spr.2,Ser.2,Ser.3,Ser.1,Spr.1,Spr.3,Mco.2,Hdi.3,Hmi.2,Tsa.3,Tso.2,Hdi.2,Hmi.4,Egr.3 <====> Tas.1,Tso.1,Tmu.2,Tmu.1,Tmu.3,Tsa.1

PARALOGY RELATIONSHIP: Egr.2,Emu.12,Emu.13,Emu.4,Emu.15,Emu.17,Emu.3,Emu.14,Emu.5,Emu.16,Emu.6,Hdi.1,Hmi.1,Hmi.3,Mco.1,Spr.2,Ser.2,Ser.3,Ser.1,Spr.1,Spr.3,Mco.2,Hdi.3,Hmi.2,Tsa.3,Tso.2,Hdi.2,Hmi.4,Egr.3,Tas.1,Tso.1,Tmu.2,Tmu.1,Tmu.3,Tsa.1 <====> Egr.1

PARALOGY RELATIONSHIP: Egr.2,Emu.12,Emu.13,Emu.4,Emu.15,Emu.17,Emu.3,Emu.14,Emu.5,Emu.16,Emu.6,Hdi.1,Hmi.1,Hmi.3,Mco.1,Spr.2,Ser.2,Ser.3,Ser.1,Spr.1,Spr.3,Mco.2,Hdi.3,Hmi.2,Tsa.3,Tso.2,Hdi.2,Hmi.4,Egr.3,Tas.1,Tso.1,Tmu.2,Tmu.1,Tmu.3,Tsa.1,Egr.1 <====> Emu.10

PARALOGY RELATIONSHIP: Egr.2,Emu.12,Emu.13,Emu.4,Emu.15,Emu.17,Emu.3,Emu.14,Emu.5,Emu.16,Emu.6,Hdi.1,Hmi.1,Hmi.3,Mco.1,Spr.2,Ser.2,Ser.3,Ser.1,Spr.1,Spr.3,Mco.2,Hdi.3,Hmi.2,Tsa.3,Tso.2,Hdi.2,Hmi.4,Egr.3,Tas.1,Tso.1,Tmu.2,Tmu.1,Tmu.3,Tsa.1,Egr.1,Emu.10 <====> Emu.11

PARALOGY RELATIONSHIP:
Egr.2,Emu.12,Emu.13,Emu.4,Emu.15,Emu.17,Emu.3,Emu.14,Emu.5,Emu.16,Emu.6,Hdi.1,Hmi.1,Hmi.3,Mco.1,Spr.2,Ser.2,Ser.3,Ser.1,Spr.1,Spr.
3,Mco.2,Hdi.3,Hmi.2,Tsa.3,Tso.2,Hdi.2,Hmi.4,Egr.3,Tas.1,Tso.1,Tmu.2,Tmu.1,Tmu.3,Tsa.1,Egr.1,Emu.10,Emu.11 <====> Emu.8

PARALOGY RELATIONSHIP:
Egr.2,Emu.12,Emu.13,Emu.4,Emu.15,Emu.17,Emu.3,Emu.14,Emu.5,Emu.16,Emu.6,Hdi.1,Hmi.1,Hmi.3,Mco.1,Spr.2,Ser.2,Ser.3,Ser.1,Spr.1,Spr.
3,Mco.2,Hdi.3,Hmi.2,Tsa.3,Tso.2,Hdi.2,Hmi.4,Egr.3,Tas.1,Tso.1,Tmu.2,Tmu.1,Tmu.3,Tsa.1,Egr.1,Emu.10,Emu.11,Emu.8 <====> Emu.1

PARALOGY RELATIONSHIP: Emu.7 <====> Emu.2

PARALOGY RELATIONSHIP:
Egr.2,Emu.12,Emu.13,Emu.4,Emu.15,Emu.17,Emu.3,Emu.14,Emu.5,Emu.16,Emu.6,Hdi.1,Hmi.1,Hmi.3,Mco.1,Spr.2,Ser.2,Ser.3,Ser.1,Spr.1,Spr.
3,Mco.2,Hdi.3,Hmi.2,Tsa.3,Tso.2,Hdi.2,Hmi.4,Egr.3,Tas.1,Tso.1,Tmu.2,Tmu.1,Tmu.3,Tsa.1,Egr.1,Emu.10,Emu.11,Emu.8,Emu.1 <====>
Emu.7,Emu.2

PARALOGY RELATIONSHIP:
Egr.2,Emu.12,Emu.13,Emu.4,Emu.15,Emu.17,Emu.3,Emu.14,Emu.5,Emu.16,Emu.6,Hdi.1,Hmi.1,Hmi.3,Mco.1,Spr.2,Ser.2,Ser.3,Ser.1,Spr.1,Spr.
3,Mco.2,Hdi.3,Hmi.2,Tsa.3,Tso.2,Hdi.2,Hmi.4,Egr.3,Tas.1,Tso.1,Tmu.2,Tmu.1,Tmu.3,Tsa.1,Egr.1,Emu.10,Emu.11,Emu.8,Emu.1,Emu.7,Emu.2
<====> Emu.9

PARALOGY RELATIONSHIP: Tso.3 <====>
Egr.2,Emu.12,Emu.13,Emu.4,Emu.15,Emu.17,Emu.3,Emu.14,Emu.5,Emu.16,Emu.6,Hdi.1,Hmi.1,Hmi.3,Mco.1,Spr.2,Ser.2,Ser.3,Ser.1,Spr.1,Spr.
3,Mco.2,Hdi.3,Hmi.2,Tsa.3,Tso.2,Hdi.2,Hmi.4,Egr.3,Tas.1,Tso.1,Tmu.2,Tmu.1,Tmu.3,Tsa.1,Egr.1,Emu.10,Emu.11,Emu.8,Emu.1,Emu.7,Emu.2,
Emu.9

PARALOGY RELATIONSHIP: Tas.2,Tsa.2,Tmu.4 <====>
Tso.3,Egr.2,Emu.12,Emu.13,Emu.4,Emu.15,Emu.17,Emu.3,Emu.14,Emu.5,Emu.16,Emu.6,Hdi.1,Hmi.1,Hmi.3,Mco.1,Spr.2,Ser.2,Ser.3,Ser.1,Spr.
1,Spr.3,Mco.2,Hdi.3,Hmi.2,Tsa.3,Tso.2,Hdi.2,Hmi.4,Egr.3,Tas.1,Tso.1,Tmu.2,Tmu.1,Tmu.3,Tsa.1,Egr.1,Emu.10,Emu.11,Emu.8,Emu.1,Emu.7,
Emu.2,Emu.9

ORTHOLOGY RELATIONSHIP:
Csi.1,Csi.2,Ofe.1,Ssp.1,Phe.1,Hsp.1,Fgi.1,Fgi.2,Fhe.1,Fhe.2,Fgi.3,Fgi.4,Fbu.1,Fbu.2,Sbo.1,Sha.2,Sma.4,Sma.3,Sma.7,Sbo.2,Sma.1,Sma.
8,Sma.11,Sma.9,Sma.12,Sha.1,Sbo.3,Sbo.4,Sma.10,Sha.3,Sja.2,Sja.1,Sma.6,Sma.13,Sja.3,Sja.4,Sja.6,Sja.7,Sja.5,Sma.2,Sma.5,Tre.5,Tre.
7,Tre.1,Tre.4,Tre.2,Tre.6,Tre.3 <====>
Tas.2,Tsa.2,Tmu.4,Tso.3,Egr.2,Emu.12,Emu.13,Emu.4,Emu.15,Emu.17,Emu.3,Emu.14,Emu.5,Emu.16,Emu.6,Hdi.1,Hmi.1,Hmi.3,Mco.1,Spr.2,Ser.
2,Ser.3,Ser.1,Spr.1,Spr.3,Mco.2,Hdi.3,Hmi.2,Tsa.3,Tso.2,Hdi.2,Hmi.4,Egr.3,Tas.1,Tso.1,Tmu.2,Tmu.1,Tmu.3,Tsa.1,Egr.1,Emu.10,Emu.11,
Emu.8,Emu.1,Emu.7,Emu.2,Emu.9

```
 _ _ _ _ _ _ _ _ _ _ _ _ _

 Species tree
 _ _ _ _ _ _ _ _ _ _ _ _ _

         /-Mco
        |
        |      /-Hmi
      /-|   /-|
     |  |  |   \-Hdi
     |  |  |
     |   \-|      /-Emu
     |     |   /-|
     |     |  |   \-Egr
     |      \-|
     |        |   /-Tso
   /-|         \-|
  |  |           |   /-Tmu
  |  |            \-|
  |  |              |   /-Tsa
  |  |               \-|
  |  |                  \-Tas
  |  |
  |  |   /-Ser
  |   \-|
--|      \-Spr
  |
  |      /-Tre
  |   /-|
  |  |  |   /-Sja
  |  |   \-|
  |  |     |   /-Sma
  |  |      \-|
  |  |        |   /-Sbo
  |  |         \-|
   \-|            \-Sha
     |
     |      /-Fbu
     |   /-|
     |  |  |   /-Fhe
     |  |   \-|
     |  |      \-Fgi
      \-|
        |      /-Ofe
        |   /-|
        |  |   \-Csi
         \-|
```

```
          |   /-Phe
           \-|
             |   /-Ssp
              \-|
                 \-Hsp
```

_ _ _ _ _ _ _ _ _ _ _ _ _

Reconciled tree

_ _ _ _ _ _ _ _ _ _ _ _ _

```
                    /-Phe
                 /-|
                |  |   /-Hsp
                |   \-|
                |      \-Ssp.1
              /-|
             |  |      /-Ofe
             |  |   /-|
             |  |  |   \-Csi.1
             |   \-|
             |     |   /-Csi.2
           /-|      \-|
          |  |         \-Ofe.1
          |  |
          |  |      /-Ofe
          |  |   /-|
          |  |  |   \-Csi
          |   \-|
          |     |      /-Ssp
          |     |   /-|
          |      \-|   \-Hsp.1
          |        |
          |         \-Phe.1
          |
          |                  /-Fhe
        /-|               /-|
       |  |              |   \-Fgi.1
       |  |            /-|
       |  |           |  |   /-Fgi.2
       |  |           |   \-|
       |  |         /-|      \-Fhe.1
       |  |        |  |
       |  |        |  |   /-Fgi
       |  |      /-|   \-|
       |  |     |  |      \-Fhe.2
       |  |     |  |
       |  |     |  |   /-Fhe
       |  |   /-|   \-|
     /-|  |  |  |      \-Fgi.3
    |  |  |  |  |
    |  |   \-|  |   /-Fhe
    |  |     |   \-|
    |  |     |      \-Fgi.4
    |  |     |
    |  |      \-Fbu.1
    |  |
    |  |         /-Fhe
    |  |      /-|
    |  |   /-|   \-Fgi
    |  |  |  |
    |  |  |   \-Fbu.2
    |   \-|
    |     |      /-Ofe
    |     |   /-|
    |     |  |   \-Csi
    |      \-|
    |        |   /-Phe
    |         \-|
    |           |   /-Ssp
    |            \-|
    |               \-Hsp
    |
    |                           /-Sja
    |                          |
    |                          |         /-Sbo.1
    |                        /-|      /-|
    |                       |  |   /-|   \-Sha.2
    |                       |  |  |  |
```

```
   |                       |  |  |   \-Sma.4
   |                       |   \-|
   |                       |     |      /-Sbo
   |                       |     |   /-|
   |                       |     |  |   \-Sha
   |                       |      \-|
   |                       |        |   /-Sma.3
   |                       |         \-|
   |                       |            \-Sma.7
   |                       |
   |                       |      /-Sja
   |                       |     |
   |                       |     |                        /-Sha
   |                       |     |                     /-|
   |                       |     |                  /-|   \-Sbo.2
   |                       |     |                 |  |
   |                       |     |                 |   \-Sma.1
   |                       |     |               /-|
   |                       |     |              |  |      /-Sbo
   |                       |     |              |  |   /-|
   |                       |     |              |   \-|   \-Sha
   |                       |     |            /-|     |
   |                       |     |           |  |      \-Sma.8
   |                     /-|     |           |  |
   |                    |  |     |           |  |      /-Sbo
   |                    |  |     |           |  |   /-|
   |                    |  |     |         /-|   \-|   \-Sha
   |                    |  |   /-|        |  |     |
   |                    |  |  |  |        |  |      \-Sma.11
   |                    |  |  |  |        |  |
   |                    |  |  |  |        |  |      /-Sbo
   |                    |  |  |  |        |  |   /-|
   |                    |  |  |  |      /-|   \-|   \-Sha
   |                    |  |  |  |     |  |     |
   |                    |  |  |  |     |  |      \-Sma.9
   |                    |  |  |  |     |  |
   |                    |  |  |  |     |  |      /-Sbo
   |                    |  |  |  |   /-|  |   /-|
   |                    |  |  |  |  |  |   \-|   \-Sha
   |                    |  |  |  |  |  |     |
   |                    |  |  |  |  |  |      \-Sma.12
   |                    |  |  |  |  |  |
   |                    |  |  |  |  |  |   /-Sma
   |                    |  |  |  |  |   \-|
   |                    |  |  |   \-|     |   /-Sbo
   |                    |  |  |     |      \-|
   |                    |  |  |     |         \-Sha.1
   |                    |  |  |     |
   |                    |  |  |     |      /-Sha
   |                    |   \-|     |   /-|
   |                    |     |     |  |  |   /-Sbo.3
   |                    |     |      \-|   \-|
 /-|                    |     |        |      \-Sbo.4
|  |                    |     |        |
|  |                    |     |         \-Sma.10
|  |                    |     |
|  |                    |     |            /-Sma
|  |                  /-|     |         /-|
|  |                 |  |     |        |  |   /-Sbo
|  |                 |  |     |      /-|   \-|
|  |                 |  |     |     |  |      \-Sha.3
|  |                 |  |     |     |  |
|  |                 |  |     |     |   \-Sja.2
|  |                 |  |     |     |
|  |                 |  |     |   /-|         /-Sbo
|  |                 |  |     |  |  |      /-|
|  |                 |  |     |  |  |     |   \-Sha
|  |                 |  |     |  |  |   /-|
|  |                 |  |     |  |  |  |  |   /-Sma.6
|  |                 |  |     |  |   \-|   \-|
|  |                 |  |     |  |     |      \-Sma.13
|  |                 |  |     |  |     |
|  |                 |  |      \-|      \-Sja.1
|  |                 |  |        |
|  |                 |  |        |      /-Sma
|  |                 |  |        |   /-|
|  |                 |  |        |  |  |   /-Sbo
|  |                 |  |        |  |   \-|
|  |               /-|  |        |  |      \-Sha
|  |              |  |  |        |  |
|  |              |  |  |         \-|         /-Sja.3
|  |              |  |  |           |      /-|
|  |              |  |  |           |     |   \-Sja.4
|  |              |  |  |           |   /-|
|  |              |  |  |           |  |  |   /-Sja.6
```

```
  |  |              |  |  |            \-|   \-|
  |  |              |  |  |              |      \-Sja.7
  |  |              |  |  |              |
  |  |              |  |  |               \-Sja.5
  |  |              |  |  |
  |  |              |  |  |   /-Sja
  |  |              |  |  |  |
  |  |            /-|  |   \-|      /-Sbo
  |  |           |  |  |     |   /-|
  |  |           |  |  |      \-|   \-Sha
  |  |           |  |  |        |
  |  |           |  |  |         \-Sma.2
  |  |           |  |  |
  |  |           |  |  |   /-Sja
  |  |           |  |  |  |
  |  |           |  |   \-|      /-Sbo
  |  |           |  |     |   /-|
  |  |           |  |      \-|   \-Sha
  |  |         /-|  |        |
  |  |        |  |  |         \-Sma.5
  |  |        |  |  |
  |  |        |  |  |   /-Tre.5
  |  |        |  |   \-|
  |  |        |  |      \-Tre.7
  |  |        |  |
  |  |        |  |      /-Sja
  |  |        |  |   /-|
  |  |        |  |  |  |   /-Sma
  |  |      /-|  |  |   \-|
  |  |     |  |   \-|     |   /-Sbo
  |  |     |  |     |      \-|
  |  |     |  |     |         \-Sha
  |  |     |  |     |
  |  |     |  |      \-Tre.1
  |  |     |  |
  |  |     |  |      /-Sja
  |  |     |  |   /-|
  |  |     |  |  |  |   /-Sma
  |  |     |  |  |   \-|
  |  |  /-|  \-|     |   /-Sbo
  |  |  |  |     |      \-|
  |  |  |  |     |         \-Sha
  |  |  |  |     |
  |  |  |  |      \-Tre.4
  |  |  |  |
  |  |  |  |      /-Sja
  |  |  |  |   /-|
  |  |  |  |  |  |   /-Sma
  |  |  |  |  |   \-|
  |  |  |  |  |     |   /-Sbo
  |   \-|   \-|      \-|
  |     |     |         \-Sha
  |     |     |
  |     |     |   /-Tre.2
  |     |      \-|
  |     |         \-Tre.6
  |     |
  |     |      /-Sja
  |     |   /-|
  |     |  |  |   /-Sma
  |     |  |   \-|
  |      \-|     |   /-Sbo
  |        |      \-|
  |        |         \-Sha
  |        |
  |         \-Tre.3
  |
  |         /-Mco
  |        |
  |        |      /-Hmi
  |      /-|   /-|
  |     |  |  |   \-Hdi
  |     |  |  |
  |     |   \-|      /-Emu
  |     |     |   /-|
  |     |     |  |   \-Egr
  |     |      \-|
--|     |        |   /-Tso
  |   /-|        |  |
  |  |  |         \-|      /-Tas.2
  |  |  |           |   /-|
  |  |  |            \-|   \-Tsa.2
  |  |  |              |
  |  |  |               \-Tmu.4
  |  |  |
```

```
|  |  |   /-Ser
|  |   \-|
|  |      \-Spr
|  |
|  |         /-Mco
|  |        |
|  |        |      /-Hmi
|  |      /-|   /-|
|  |     |  |  |   \-Hdi
|  |     |  |  |
|  |     |   \-|      /-Emu
|  |     |     |   /-|
|  |     |     |  |   \-Egr
|  |     |     |  |
|  |     |      \-|      /-Tmu
|  |   /-|        |   /-|
|  |  |  |        |  |  |   /-Tsa
|  |  |  |         \-|   \-|
|  |  |  |           |      \-Tas
|  |  |  |           |
|  |  |  |            \-Tso.3
|  |  |  |
|  |  |  |   /-Ser
|  |  |   \-|
|  |  |      \-Spr
|  |  |
|  |  |                                    /-Mco
|  |  |                                   |
|  |  |                                   |      /-Hmi
|  |  |                                 /-|   /-|
|  |  |                                |  |  |   \-Hdi
|  |  |                                |  |  |
|  |  |                                |   \-|      /-Emu
|  |  |                                |     |   /-|
|  |  |                                |     |  |   \-Egr.2
|  |  |                                |      \-|
|  |  |                                |        |   /-Tso
|  |  |                              /-|         \-|
|  |  |                             |  |           |   /-Tmu
|  |  |                             |  |            \-|
|  |  |                             |  |              |   /-Tsa
|  |  |                             |  |               \-|
|  |  |                             |  |                  \-Tas
|  |  |                             |  |
|  |  |                             |  |   /-Ser
|  |  |                             |   \-|
|  |  |                             |      \-Spr
|  |  |                             |
|  |  |                             |         /-Mco
|  |  |                             |        |
|  |  |                             |        |      /-Hmi
|  |  |                             |        |   /-|
|  |  |                             |        |  |   \-Hdi
|  |  |                             |      /-|  |
|  |  |                             |     |  |  |      /-Egr
|  |  |                             |     |  |  |     |
|  |  |                             |     |  |  |     |      /-Emu.12
|  |  |                             |     |  |  |     |     |
|  |  |                             |     |  |  |     |     |         /-Emu.13
|  |  |                             |     |   \-|   /-|   /-|      /-|
|  |  |                             |     |     |  |  |  |  |   /-|   \-Emu.4
|  |  |                             |     |     |  |  |  |  |  |  |
|  |  |                             |     |     |  |  |  |   \-|   \-Emu.15
|  |  |                             |     |     |  |  |  |     |
|  |  |                             |     |     |  |  |  |     |   /-Emu.17
|  |  |                             |     |     |  |   \-|      \-|
|  |  |                             |     |     |  |     |         \-Emu.3
|  |  |                             |     |     |  |     |
|  |  |                             |     |      \-|     |      /-Emu.14
|  |  |                             |   /-|        |     |   /-|
|  |  |                             |  |  |        |     |  |   \-Emu.5
|  |  |                             |  |  |        |      \-|
|  |  |                             |  |  |        |        |   /-Emu.16
|  |  |                             |  |  |        |         \-|
|  |  |                             |  |  |        |            \-Emu.6
|  |  |                             |  |  |        |
|  |  |                             |  |  |        |   /-Tso
|  |  |                             |  |  |         \-|
|  |  |                             |  |  |           |   /-Tmu
|  |  |                           /-|  |  |            \-|
|  |  |                          |  |  |  |              |   /-Tsa
|  |  |                          |  |  |  |               \-|
|  |  |                          |  |  |  |                  \-Tas
|  |  |                          |  |  |  |
|  |  |                          |  |  |  |   /-Ser
```

```
|  |  |                          |  |  |   \-|
|  |  |                          |  |  |      \-Spr
|  |  |                          |  |  |
|  |  |                          |  |  |               /-Mco
|  |  |                          |  |  |              |
|  |  |                          |  |  |              |         /-Emu
|  |  |                          |  |  |              |      /-|
|  |  |                          |  |  |              |     |   \-Egr
|  |  |                          |  |  |            /-|   /-|
|  |  |                          |  |  |           |  |  |  |   /-Tso
|  |  |                          |  |  |           |  |  |   \-|
|  |  |                          |  |  |           |  |  |     |   /-Tmu
|  |  |                          |  |  |           |  |  |      \-|
|  |  |                          |  |  |           |   \-|        |   /-Tsa
|  |  |                          |  |  |           |     |         \-|
|  |  |                          |  |  |           |     |            \-Tas
|  |  |                          |  |  |         /-|     |
 \-|  |                          |  |  |        |  |     |   /-Hdi.1
   |  |                          |  |  |        |  |      \-|
   |  |                          |  |  |        |  |        |   /-Hmi.1
   |  |                          |  |  |        |  |         \-|
   |  |                          |  |  |        |  |            \-Hmi.3
   |  |                          |  |  |        |  |
   |  |                          |  |  |        |  |   /-Ser
   |  |                          |  |  |        |   \-|
   |  |                          |  |  |        |      \-Spr
   |  |                          |  |  |        |
   |  |                          |  |  |        |               /-Hmi
   |  |                          |  |  |        |            /-|
   |  |                          |  |  |        |           |   \-Hdi
   |  |                          |  |  |        |           |
   |  |                          |  |  |        |         /-|      /-Emu
   |  |                          |  |  |        |        |  |   /-|
   |  |                          |  |  |        |        |  |  |   \-Egr
   |  |                          |  |  |        |        |   \-|
   |  |                          |  |  |        |        |     |   /-Tso
   |  |                          |  |  |        |        |      \-|
   |  |                          |   \-|      /-|      /-|        |   /-Tmu
   |  |                          |     |     |  |     |  |         \-|
   |  |                          |     |     |  |     |  |           |   /-Tsa
   |  |                          |     |     |  |     |  |            \-|
   |  |                          |     |     |  |     |  |               \-Tas
   |  |                          |     |     |  |     |  |
   |  |                          |     |     |  |     |   \-Mco.1
   |  |                          |     |     |  |   /-|
   |  |                          |     |     |  |  |  |         /-Spr.2
   |  |                          |     |     |  |  |  |      /-|
   |  |                          |     |     |  |  |  |     |   \-Ser.2
   |  |                          |     |     |  |  |  |   /-|
   |  |                          |     |     |  |  |  |  |  |   /-Spr
   |  |                          |     |     |  |  |  |  |   \-|
   |  |                          |     |     |  |  |   \-|      \-Ser.3
   |  |                          |     |     |  |  |     |
   |  |                          |     |     |  |  |     |   /-Ser.1
   |  |                          |     |     |  |  |      \-|
   |  |                          |     |     |  |  |        |   /-Spr.1
   |  |                          |     |     |  |  |         \-|
   |  |                          |     |     |  |  |            \-Spr.3
   |  |                          |     |     |   \-|
   |  |                          |     |     |     |            /-Hmi
   |  |                          |     |     |     |         /-|
   |  |                          |     |     |     |        |   \-Hdi
   |  |                          |     |     |     |        |
   |  |                          |     |     |     |      /-|      /-Emu
   |  |                          |     |     |     |     |  |   /-|
   |  |                        /-|     |   /-|     |     |  |  |   \-Egr
   |  |                       |  |     |  |  |     |     |   \-|
   |  |                       |  |     |  |  |     |     |     |   /-Tso
   |  |                       |  |     |  |  |     |     |      \-|
   |  |                       |  |     |  |  |     |   /-|        |   /-Tmu
   |  |                       |  |     |  |  |     |  |  |         \-|
   |  |                       |  |     |  |  |     |  |  |           |   /-Tsa
   |  |                       |  |     |  |  |     |  |  |            \-|
   |  |                       |  |     |  |  |      \-|  |               \-Tas
   |  |                       |  |     |  |  |        |  |
   |  |                       |  |     |  |  |        |   \-Mco.2
   |  |                       |  |     |  |  |        |
   |  |                       |  |     |  |  |        |   /-Ser
   |  |                       |  |     |  |  |         \-|
   |  |                       |  |     |  |  |            \-Spr
   |  |                       |  |     |  |  |
   |  |                       |  |     |  |  |      /-Mco
   |  |                       |  |     |  |  |     |
   |  |                       |  |     |  |  |     |         /-Emu
   |  |                       |  |     |  |  |     |      /-|
```

```
|  |                       |  |     |  |  |     |     |   \-Egr
|  |                       |  |     |  |  |   /-|     |
|  |                       |  |     |  |  |  |  |   /-|      /-Tmu
|  |                       |  |     |  |  |  |  |  |  |   /-|
|  |                       |  |     |  |  |  |  |  |  |  |  |   /-Tas
|  |                       |  |      \-|  |  |  |  |   \-|   \-|
|  |                       |  |        |  |  |   \-|     |      \-Tsa.3
|  |                       |  |        |  |  |     |     |
|  |                       |  |        |   \-|     |      \-Tso.2
|  |                       |  |        |     |     |
|  |                       |  |        |     |     |   /-Hdi.3
|  |                       |  |        |     |      \-|
|  |                       |  |        |     |         \-Hmi.2
|  |                       |  |        |     |
|  |                       |  |        |     |   /-Ser
|  |                       |  |        |      \-|
|  |                       |  |        |         \-Spr
|  |                       |  |        |
|  |                       |  |        |      /-Mco
|  |                       |  |        |     |
|  |                       |  |        |     |         /-Emu
|  |                       |  |        |     |      /-|
|  |                       |  |        |     |     |   \-Egr
|  |                       |  |        |   /-|   /-|
|  |                       |  |        |  |  |  |  |   /-Tso
|  |                       |  |        |  |  |  |   \-|
|  |                       |  |        |  |  |  |     |   /-Tmu
|  |                       |  |        |  |  |  |      \-|
|  |                       |  |        |  |   \-|        |   /-Tsa
|  |                       |  |        |  |     |         \-|
|  |                       |  |         \-|     |            \-Tas
|  |                       |  |           |     |
|  |                       |  |           |     |   /-Hdi.2
|  |                       |  |           |      \-|
|  |                       |  |           |         \-Hmi.4
|  |                     /-|  |           |
|  |                    |  |  |           |   /-Ser
|  |                    |  |  |            \-|
|  |                    |  |  |               \-Spr
|  |                    |  |  |
|  |                    |  |  |      /-Mco
 \-|                    |  |  |     |
   |                    |  |  |     |      /-Hmi
   |                    |  |  |   /-|   /-|
   |                    |  |  |  |  |  |   \-Hdi
   |                    |  |  |  |  |  |
   |                    |  |  |  |   \-|      /-Emu
   |                    |  |  |  |     |   /-|
   |                    |  |  |  |     |  |   \-Egr.3
   |                    |  |  |  |      \-|
   |                    |  |  |  |        |   /-Tso
   |                    |  |   \-|         \-|
   |                    |  |     |           |   /-Tmu
   |                    |  |     |            \-|
   |                    |  |     |              |   /-Tsa
   |                    |  |     |               \-|
   |                    |  |     |                  \-Tas
   |                    |  |     |
   |                    |  |     |   /-Ser
   |                    |  |      \-|
   |                    |  |         \-Spr
   |                    |  |
   |                    |  |      /-Mco
   |                    |  |     |
   |                    |  |     |      /-Hmi
   |                    |  |     |   /-|
   |                    |  |     |  |   \-Hdi
   |                    |  |     |  |
   |                    |  |   /-|  |      /-Emu
   |                    |  |  |  |  |   /-|
   |                    |  |  |  |  |  |   \-Egr
   |                    |  |  |  |  |  |
   |                    |  |  |  |  |  |                  /-Tmu
   |                    |  |  |  |  |  |               /-|
   |                    |  |  |   \-|  |              |  |   /-Tsa
   |                    |  |  |     |  |            /-|   \-|
   |                    |  |  |     |  |           |  |      \-Tas.1
   |                    |  |  |     |  |           |  |
   |                    |  |  |     |  |         /-|   \-Tso.1
   |                    |  |  |     |  |        |  |
   |                    |  |  |     |  |        |  |   /-Tso
   |                  /-|  |  |     |  |        |  |  |
   |                 |  |  |  |     |  |        |   \-|      /-Tsa
   |                 |  |  |  |      \-|        |     |   /-|
   |                 |  |  |  |        |      /-|      \-|   \-Tas
```

```
|                 |  |  |  |        |     |  |        |
|                 |  |  |  |        |     |  |         \-Tmu.2
|                 |  |  |  |        |     |  |
|                 |  |  |  |        |     |  |   /-Tso
|                 |  |  |  |        |     |  |  |
|                 |  |   \-|        |     |   \-|      /-Tsa
|                 |  |     |        |   /-|     |   /-|
|                 |  |     |        |  |  |      \-|   \-Tas
|                 |  |     |        |  |  |        |
|                 |  |     |        |  |  |         \-Tmu.1
|                 |  |     |        |  |  |
|                 |  |     |        |  |  |   /-Tso
|                 |  |     |        |  |  |  |
|                 |  |     |         \-|   \-|      /-Tsa
|                 |  |     |           |     |   /-|
|                 |  |     |           |      \-|   \-Tas
|                 |  |     |           |        |
|                 |  |     |           |         \-Tmu.3
|                 |  |     |           |
|                 |  |     |           |   /-Tso
|                 |  |     |            \-|
|                 |  |     |              |   /-Tmu
|                 |  |     |               \-|
|                 |  |     |                 |   /-Tas
|                 |  |     |                  \-|
|                 |  |     |                     \-Tsa.1
|                 |  |     |
|                 |  |     |   /-Ser
|                 |  |      \-|
|                 |  |         \-Spr
|               /-|  |
|              |  |  |      /-Mco
|              |  |  |     |
|              |  |  |     |      /-Hmi
|              |  |  |   /-|   /-|
|              |  |  |  |  |  |   \-Hdi
|              |  |  |  |  |  |
|              |  |  |  |   \-|      /-Emu
|              |  |  |  |     |   /-|
|              |  |  |  |     |  |   \-Egr.1
|              |  |  |  |      \-|
|              |  |  |  |        |   /-Tso
|              |  |   \-|         \-|
|              |  |     |           |   /-Tmu
|              |  |     |            \-|
|              |  |     |              |   /-Tsa
|              |  |     |               \-|
|              |  |     |                  \-Tas
|              |  |     |
|              |  |     |   /-Ser
|              |  |      \-|
|              |  |         \-Spr
|              |  |
|              |  |      /-Mco
|              |  |     |
|              |  |     |      /-Hmi
|              |  |   /-|   /-|
|              |  |  |  |  |   \-Hdi
|            /-|  |  |  |  |
|           |  |  |  |   \-|      /-Egr
|           |  |  |  |     |   /-|
|           |  |  |  |     |  |   \-Emu.10
|           |  |  |  |      \-|
|           |  |  |  |        |   /-Tso
|           |  |   \-|         \-|
|           |  |     |           |   /-Tmu
|           |  |     |            \-|
|           |  |     |              |   /-Tsa
|           |  |     |               \-|
|           |  |     |                  \-Tas
|           |  |     |
|           |  |     |   /-Ser
|           |  |      \-|
|           |  |         \-Spr
|           |  |
|           |  |      /-Mco
|           |  |     |
|           |  |     |      /-Hmi
|           |  |   /-|   /-|
|           |  |  |  |  |   \-Hdi
|           |  |  |  |  |
|           |  |  |   \-|      /-Egr
|           |  |  |     |   /-|
|         /-|  |  |     |  |   \-Emu.11
|        |  |  |  |      \-|
```

```
|        |  |  |  |        |   /-Tso
|        |  |   \-|         \-|
|        |  |     |           |   /-Tmu
|        |  |     |            \-|
|        |  |     |              |   /-Tsa
|        |  |     |               \-|
|        |  |     |                  \-Tas
|        |  |     |
|        |  |     |   /-Ser
|        |  |      \-|
|        |  |         \-Spr
|        |  |
|        |  |      /-Mco
|        |  |     |
|        |  |     |      /-Hmi
|        |  |   /-|   /-|
|        |  |  |  |  |   \-Hdi
|        |  |  |  |  |
|        |  |  |   \-|      /-Egr
|        |  |  |     |   /-|
|        |  |  |     |  |   \-Emu.8
|      /-|  |  |      \-|
|     |  |  |  |        |   /-Tso
|     |  |   \-|         \-|
|     |  |     |           |   /-Tmu
|     |  |     |            \-|
|     |  |     |              |   /-Tsa
|     |  |     |               \-|
|     |  |     |                  \-Tas
|     |  |     |
|     |  |     |   /-Ser
|     |  |      \-|
|     |  |         \-Spr
|     |  |
|     |  |      /-Mco
|     |  |     |
|     |  |     |      /-Hmi
|     |  |   /-|   /-|
|     |  |  |  |  |   \-Hdi
|     |  |  |  |  |
|     |  |  |   \-|      /-Egr
|     |  |  |     |   /-|
|     |  |  |     |  |   \-Emu.1
|     |  |  |      \-|
|   /-|  |  |        |   /-Tso
|  |  |   \-|         \-|
|  |  |     |           |   /-Tmu
|  |  |     |            \-|
|  |  |     |              |   /-Tsa
|  |  |     |               \-|
|  |  |     |                  \-Tas
|  |  |     |
|  |  |     |   /-Ser
|  |  |      \-|
|  |  |         \-Spr
|  |  |
|  |  |      /-Mco
|  |  |     |
|  |  |     |      /-Hmi
|  |  |   /-|   /-|
|  |  |  |  |  |   \-Hdi
|  |  |  |  |  |
|  |  |  |   \-|      /-Egr
|  |  |  |     |   /-|
|  |  |  |     |  |  |   /-Emu.7
|  |  |  |     |  |   \-|
|  |  |  |      \-|      \-Emu.2
 \-|  |  |        |
   |  \-|        |   /-Tso
   |     |         \-|
   |     |           |   /-Tmu
   |     |            \-|
   |     |              |   /-Tsa
   |     |               \-|
   |     |                  \-Tas
   |     |
   |     |   /-Ser
   |      \-|
   |         \-Spr
   |
   |      /-Mco
   |     |
   |     |      /-Hmi
   |   /-|   /-|
   |  |  |  |   \-Hdi
```

```
|  |  |  |
|  |   \-|      /-Egr
|  |     |   /-|
|  |     |  |   \-Emu.9
|  |      \-|
|  |        |   /-Tso
 \-|        \-|
   |          |   /-Tmu
   |          \-|
   |             |   /-Tsa
   |             \-|
   |                \-Tas
   |
   |   /-Ser
    \-|
       \-Spr
```

---

## **Genus Specific Paralogs (indicated in Figure 3):**

PARALOGY RELATIONSHIP: Fasciola_gigantica_SL_1_Unique_SL-13 <====> Fasciola_gigantica_SL_2_Unique_SL-14,Fasciola_hepatica_SL_1_Unique_SL-13

PARALOGY RELATIONSHIP: Fasciola_gigantica_SL_1_Unique_SL-13,Fasciola_gigantica_SL_2_Unique_SL-14,Fasciola_hepatica_SL_1_Unique_SL-13 <====> Fasciola_hepatica_SL_2_Unique_SL-37

PARALOGY RELATIONSHIP: Fasciola_gigantica_SL_1_Unique_SL-13,Fasciola_gigantica_SL_2_Unique_SL-14,Fasciola_hepatica_SL_1_Unique_SL-13,Fasciola_hepatica_SL_2_Unique_SL-37 <====> Fasciola_gigantica_SL_3_Unique_SL-35

PARALOGY RELATIONSHIP: Fasciola_gigantica_SL_1_Unique_SL-13,Fasciola_gigantica_SL_2_Unique_SL-14,Fasciola_hepatica_SL_1_Unique_SL-13,Fasciola_hepatica_SL_2_Unique_SL-37,Fasciola_gigantica_SL_3_Unique_SL-35 <====> Fasciola_gigantica_SL_4_Unique_SL-36

PARALOGY RELATIONSHIP: Schistosoma_bovis_SL_1_Unique_SL-18,Schistosoma_haematobium_SL_2_Unique_SL-20,Schistosoma_mansoni_SL_4_Unique_SL-55 <====> Schistosoma_mansoni_SL_3_Unique_SL-54,Schistosoma_mansoni_SL_7_Unique_SL-58

PARALOGY RELATIONSHIP: Schistosoma_bovis_SL_2_Unique_SL-19,Schistosoma_mansoni_SL_1_Unique_SL-19 <====> Trematoda_Schistosoma_mansoni_rajko.90nt.slRNA-31.1.1_Reference_SL_Unique_SL-19

PARALOGY RELATIONSHIP: Schistosoma_bovis_SL_2_Unique_SL-19,Schistosoma_mansoni_SL_1_Unique_SL-19,Trematoda_Schistosoma_mansoni_rajko.90nt.slRNA-31.1.1_Reference_SL_Unique_SL-19 <====> Trematoda_Schistosoma_mansoni_rajko.90nt.slRNA-46.1.1_Reference_SL_Unique_SL-19

PARALOGY RELATIONSHIP: Schistosoma_bovis_SL_2_Unique_SL-19,Schistosoma_mansoni_SL_1_Unique_SL-19,Trematoda_Schistosoma_mansoni_rajko.90nt.slRNA-31.1.1_Reference_SL_Unique_SL-19,Trematoda_Schistosoma_mansoni_rajko.90nt.slRNA-46.1.1_Reference_SL_Unique_SL-19 <====> Trematoda_Schistosoma_mansoni_rajko.90nt.slRNA-39.1.1_Reference_SL_Unique_SL-19

PARALOGY RELATIONSHIP: Schistosoma_bovis_SL_2_Unique_SL-19,Schistosoma_mansoni_SL_1_Unique_SL-19,Trematoda_Schistosoma_mansoni_rajko.90nt.slRNA-31.1.1_Reference_SL_Unique_SL-19,Trematoda_Schistosoma_mansoni_rajko.90nt.slRNA-46.1.1_Reference_SL_Unique_SL-19,Trematoda_Schistosoma_mansoni_rajko.90nt.slRNA-39.1.1_Reference_SL_Unique_SL-19 <====> Trematoda_Schistosoma_mansoni_rajko.90nt.slRNA-5.1.1_Reference_SL_Unique_SL-19

PARALOGY RELATIONSHIP: Schistosoma_bovis_SL_2_Unique_SL-19,Schistosoma_mansoni_SL_1_Unique_SL-19,Trematoda_Schistosoma_mansoni_rajko.90nt.slRNA-31.1.1_Reference_SL_Unique_SL-19,Trematoda_Schistosoma_mansoni_rajko.90nt.slRNA-46.1.1_Reference_SL_Unique_SL-19,Trematoda_Schistosoma_mansoni_rajko.90nt.slRNA-39.1.1_Reference_SL_Unique_SL-19,Trematoda_Schistosoma_mansoni_rajko.90nt.slRNA-5.1.1_Reference_SL_Unique_SL-19 <====> Schistosoma_haematobium_SL_1_Unique_SL-19

PARALOGY RELATIONSHIP: Schistosoma_bovis_SL_2_Unique_SL-19,Schistosoma_mansoni_SL_1_Unique_SL-19,Trematoda_Schistosoma_mansoni_rajko.90nt.slRNA-31.1.1_Reference_SL_Unique_SL-19,Trematoda_Schistosoma_mansoni_rajko.90nt.slRNA-46.1.1_Reference_SL_Unique_SL-19,Trematoda_Schistosoma_mansoni_rajko.90nt.slRNA-39.1.1_Reference_SL_Unique_SL-19,Trematoda_Schistosoma_mansoni_rajko.90nt.slRNA-5.1.1_Reference_SL_Unique_SL-19,Schistosoma_haematobium_SL_1_Unique_SL-19 <====> Schistosoma_bovis_SL_3_Unique_SL-48,Schistosoma_bovis_SL_4_Unique_SL-49,Trematoda_Schistosoma_mansoni_rajko.90nt.slRNA-45.1.1_Reference_SL_Unique_SL-75

PARALOGY RELATIONSHIP: Schistosoma_haematobium_SL_3_Unique_SL-50,Schistosoma_japonicum_SL_2_Unique_SL-22 <====> Schistosoma_japonicum_SL_1_Unique_SL-21,Schistosoma_mansoni_SL_6_Unique_SL-57,Trematoda_Schistosoma_mansoni_Smp_rajko_90nt_slRNA-1.1.1_Reference_SL_Unique_SL-57

PARALOGY RELATIONSHIP: Schistosoma_haematobium_SL_3_Unique_SL-50,Schistosoma_japonicum_SL_2_Unique_SL-22,Schistosoma_japonicum_SL_1_Unique_SL-21,Schistosoma_mansoni_SL_6_Unique_SL-57,Trematoda_Schistosoma_mansoni_Smp_rajko_90nt_slRNA-1.1.1_Reference_SL_Unique_SL-57 <====> Schistosoma_japonicum_SL_3_Unique_SL-23,Schistosoma_japonicum_SL_4_Unique_SL-24,Schistosoma_japonicum_SL_6_Unique_SL-52,Schistosoma_japonicum_SL_7_Unique_SL-53,Schistosoma_japonicum_SL_5_Unique_SL-51

PARALOGY RELATIONSHIP: Schistosoma_bovis_SL_2_Unique_SL-19,Schistosoma_mansoni_SL_1_Unique_SL-19,Trematoda_Schistosoma_mansoni_rajko.90nt.slRNA-31.1.1_Reference_SL_Unique_SL-19,Trematoda_Schistosoma_mansoni_rajko.90nt.slRNA-46.1.1_Reference_SL_Unique_SL-19,Trematoda_Schistosoma_mansoni_rajko.90nt.slRNA-39.1.1_Reference_SL_Unique_SL-19,Trematoda_Schistosoma_mansoni_rajko.90nt.slRNA-5.1.1_Reference_SL_Unique_SL-19,Schistosoma_haematobium_SL_1_Unique_SL-19,Schistosoma_bovis_SL_3_Unique_SL-48,Schistosoma_bovis_SL_4_Unique_SL-49,Trematoda_Schistosoma_mansoni_rajko.90nt.slRNA-45.1.1_Reference_SL_Unique_SL-75 <====> Schistosoma_haematobium_SL_3_Unique_SL-50,Schistosoma_japonicum_SL_2_Unique_SL-22,Schistosoma_japonicum_SL_1_Unique_SL-21,Schistosoma_mansoni_SL_6_Unique_SL-57,Trematoda_Schistosoma_mansoni_Smp_rajko_90nt_slRNA-1.1.1_Reference_SL_Unique_SL-57,Schistosoma_japonicum_SL_3_Unique_SL-

23,Schistosoma_japonicum_SL_4_Unique_SL-24,Schistosoma_japonicum_SL_6_Unique_SL-52,Schistosoma_japonicum_SL_7_Unique_SL-
53,Schistosoma_japonicum_SL_5_Unique_SL-51

PARALOGY RELATIONSHIP: Schistosoma_bovis_SL_1_Unique_SL-18,Schistosoma_haematobium_SL_2_Unique_SL-
20,Schistosoma_mansoni_SL_4_Unique_SL-55,Schistosoma_mansoni_SL_3_Unique_SL-54,Schistosoma_mansoni_SL_7_Unique_SL-58 <====>
Schistosoma_bovis_SL_2_Unique_SL-19,Schistosoma_mansoni_SL_1_Unique_SL-19,Trematoda_Schistosoma_mansoni_rajko.90nt.slRNA-
31.1.1_Reference_SL_Unique_SL-19,Trematoda_Schistosoma_mansoni_rajko.90nt.slRNA-46.1.1_Reference_SL_Unique_SL-
19,Trematoda_Schistosoma_mansoni_rajko.90nt.slRNA-39.1.1_Reference_SL_Unique_SL-19,Trematoda_Schistosoma_mansoni_rajko.90nt.slRNA-
5.1.1_Reference_SL_Unique_SL-19,Schistosoma_haematobium_SL_1_Unique_SL-19,Schistosoma_bovis_SL_3_Unique_SL-
48,Schistosoma_bovis_SL_4_Unique_SL-49,Trematoda_Schistosoma_mansoni_rajko.90nt.slRNA-45.1.1_Reference_SL_Unique_SL-
75,Schistosoma_haematobium_SL_3_Unique_SL-50,Schistosoma_japonicum_SL_2_Unique_SL-22,Schistosoma_japonicum_SL_1_Unique_SL-
21,Schistosoma_mansoni_SL_6_Unique_SL-57,Trematoda_Schistosoma_mansoni_Smp_rajko_90nt_slRNA-1.1.1_Reference_SL_Unique_SL-
57,Schistosoma_japonicum_SL_3_Unique_SL-23,Schistosoma_japonicum_SL_4_Unique_SL-24,Schistosoma_japonicum_SL_6_Unique_SL-
52,Schistosoma_japonicum_SL_7_Unique_SL-53,Schistosoma_japonicum_SL_5_Unique_SL-51

PARALOGY RELATIONSHIP: Schistosoma_bovis_SL_1_Unique_SL-18,Schistosoma_haematobium_SL_2_Unique_SL-
20,Schistosoma_mansoni_SL_4_Unique_SL-55,Schistosoma_mansoni_SL_3_Unique_SL-54,Schistosoma_mansoni_SL_7_Unique_SL-
58,Schistosoma_bovis_SL_2_Unique_SL-19,Schistosoma_mansoni_SL_1_Unique_SL-19,Trematoda_Schistosoma_mansoni_rajko.90nt.slRNA-
31.1.1_Reference_SL_Unique_SL-19,Trematoda_Schistosoma_mansoni_rajko.90nt.slRNA-46.1.1_Reference_SL_Unique_SL-
19,Trematoda_Schistosoma_mansoni_rajko.90nt.slRNA-39.1.1_Reference_SL_Unique_SL-19,Trematoda_Schistosoma_mansoni_rajko.90nt.slRNA-
5.1.1_Reference_SL_Unique_SL-19,Schistosoma_haematobium_SL_1_Unique_SL-19,Schistosoma_bovis_SL_3_Unique_SL-
48,Schistosoma_bovis_SL_4_Unique_SL-49,Trematoda_Schistosoma_mansoni_rajko.90nt.slRNA-45.1.1_Reference_SL_Unique_SL-
75,Schistosoma_haematobium_SL_3_Unique_SL-50,Schistosoma_japonicum_SL_2_Unique_SL-22,Schistosoma_japonicum_SL_1_Unique_SL-
21,Schistosoma_mansoni_SL_6_Unique_SL-57,Trematoda_Schistosoma_mansoni_Smp_rajko_90nt_slRNA-1.1.1_Reference_SL_Unique_SL-
57,Schistosoma_japonicum_SL_3_Unique_SL-23,Schistosoma_japonicum_SL_4_Unique_SL-24,Schistosoma_japonicum_SL_6_Unique_SL-
52,Schistosoma_japonicum_SL_7_Unique_SL-53,Schistosoma_japonicum_SL_5_Unique_SL-51 <====> Schistosoma_mansoni_SL_2_Unique_SL-25

PARALOGY RELATIONSHIP: Schistosoma_bovis_SL_1_Unique_SL-18,Schistosoma_haematobium_SL_2_Unique_SL-
20,Schistosoma_mansoni_SL_4_Unique_SL-55,Schistosoma_mansoni_SL_3_Unique_SL-54,Schistosoma_mansoni_SL_7_Unique_SL-
58,Schistosoma_bovis_SL_2_Unique_SL-19,Schistosoma_mansoni_SL_1_Unique_SL-19,Trematoda_Schistosoma_mansoni_rajko.90nt.slRNA-
31.1.1_Reference_SL_Unique_SL-19,Trematoda_Schistosoma_mansoni_rajko.90nt.slRNA-46.1.1_Reference_SL_Unique_SL-
19,Trematoda_Schistosoma_mansoni_rajko.90nt.slRNA-39.1.1_Reference_SL_Unique_SL-19,Trematoda_Schistosoma_mansoni_rajko.90nt.slRNA-
5.1.1_Reference_SL_Unique_SL-19,Schistosoma_haematobium_SL_1_Unique_SL-19,Schistosoma_bovis_SL_3_Unique_SL-
48,Schistosoma_bovis_SL_4_Unique_SL-49,Trematoda_Schistosoma_mansoni_rajko.90nt.slRNA-45.1.1_Reference_SL_Unique_SL-
75,Schistosoma_haematobium_SL_3_Unique_SL-50,Schistosoma_japonicum_SL_2_Unique_SL-22,Schistosoma_japonicum_SL_1_Unique_SL-
21,Schistosoma_mansoni_SL_6_Unique_SL-57,Trematoda_Schistosoma_mansoni_Smp_rajko_90nt_slRNA-1.1.1_Reference_SL_Unique_SL-
57,Schistosoma_japonicum_SL_3_Unique_SL-23,Schistosoma_japonicum_SL_4_Unique_SL-24,Schistosoma_japonicum_SL_6_Unique_SL-
52,Schistosoma_japonicum_SL_7_Unique_SL-53,Schistosoma_japonicum_SL_5_Unique_SL-51,Schistosoma_mansoni_SL_2_Unique_SL-25 <====>
Schistosoma_mansoni_SL_5_Unique_SL-56

PARALOGY RELATIONSHIP: Taenia_asiatica_SL_1_Unique_SL-28,Cestodes_Taenia_solium_AJ428456.1_Reference_SL_Unique_SL-28 <====>
Taenia_multiceps_SL_2_Unique_SL-29

PARALOGY RELATIONSHIP: Taenia_asiatica_SL_1_Unique_SL-28,Cestodes_Taenia_solium_AJ428456.1_Reference_SL_Unique_SL-
28,Taenia_multiceps_SL_2_Unique_SL-29 <====> Taenia_multiceps_SL_1_Unique_SL-28

PARALOGY RELATIONSHIP: Taenia_asiatica_SL_1_Unique_SL-28,Cestodes_Taenia_solium_AJ428456.1_Reference_SL_Unique_SL-
28,Taenia_multiceps_SL_2_Unique_SL-29,Taenia_multiceps_SL_1_Unique_SL-28 <====> Taenia_multiceps_SL_3_Unique_SL-30

PARALOGY RELATIONSHIP: Taenia_asiatica_SL_1_Unique_SL-28,Cestodes_Taenia_solium_AJ428456.1_Reference_SL_Unique_SL-
28,Taenia_multiceps_SL_2_Unique_SL-29,Taenia_multiceps_SL_1_Unique_SL-28,Taenia_multiceps_SL_3_Unique_SL-30 <====>
Taenia_saginata_SL_1_Unique_SL-31

## Species Specific Paralogs (indicated in Figure 3):

PARALOGY RELATIONSHIP: Schistosoma_mansoni_SL_3_Unique_SL-54 <====> Schistosoma_mansoni_SL_7_Unique_SL-58

PARALOGY RELATIONSHIP: Schistosoma_bovis_SL_3_Unique_SL-48 <====> Schistosoma_bovis_SL_4_Unique_SL-49

PARALOGY RELATIONSHIP: Schistosoma_mansoni_SL_6_Unique_SL-57 <====> Trematoda_Schistosoma_mansoni_Smp_rajko_90nt_slRNA-
1.1.1_Reference_SL_Unique_SL-57

PARALOGY RELATIONSHIP: Schistosoma_japonicum_SL_3_Unique_SL-23 <====> Schistosoma_japonicum_SL_4_Unique_SL-24

PARALOGY RELATIONSHIP: Schistosoma_japonicum_SL_6_Unique_SL-52 <====> Schistosoma_japonicum_SL_7_Unique_SL-53

PARALOGY RELATIONSHIP: Schistosoma_japonicum_SL_3_Unique_SL-23,Schistosoma_japonicum_SL_4_Unique_SL-24 <====>
Schistosoma_japonicum_SL_6_Unique_SL-52,Schistosoma_japonicum_SL_7_Unique_SL-53

PARALOGY RELATIONSHIP: Schistosoma_japonicum_SL_3_Unique_SL-23,Schistosoma_japonicum_SL_4_Unique_SL-
24,Schistosoma_japonicum_SL_6_Unique_SL-52,Schistosoma_japonicum_SL_7_Unique_SL-53 <====> Schistosoma_japonicum_SL_5_Unique_SL-51

PARALOGY RELATIONSHIP: Trichobilharzia_regenti_SL_5_Unique_SL-69 <====> Trichobilharzia_regenti_SL_7_Unique_SL-71

PARALOGY RELATIONSHIP: Trichobilharzia_regenti_SL_2_Unique_SL-66 <====> Trichobilharzia_regenti_SL_6_Unique_SL-70

PARALOGY RELATIONSHIP: Echinococcus_multilocularis_SL_5_Unique_SL-3 <====>
Cestodes_Echinococcus_multilocularis_Emu.SL2b_Reference_SL_Unique_SL-3

PARALOGY RELATIONSHIP: Echinococcus_multilocularis_SL_5_Unique_SL-
3,Cestodes_Echinococcus_multilocularis_Emu.SL2b_Reference_SL_Unique_SL-3 <====> Echinococcus_multilocularis_SL_7_Unique_SL-5

PARALOGY RELATIONSHIP: Echinococcus_multilocularis_SL_9_Unique_SL-7 <====>
Cestodes_Echinococcus_multilocularis_Emu.SL2a_Reference_SL_Unique_SL-7

PARALOGY RELATIONSHIP: Echinococcus_multilocularis_SL_5_Unique_SL-3,Cestodes_Echinococcus_multilocularis_Emu.SL2b_Reference_SL_Unique_SL-3,Echinococcus_multilocularis_SL_7_Unique_SL-5 <====>
Echinococcus_multilocularis_SL_9_Unique_SL-7,Cestodes_Echinococcus_multilocularis_Emu.SL2a_Reference_SL_Unique_SL-7

PARALOGY RELATIONSHIP: Echinococcus_multilocularis_SL_4_Unique_SL-2 <====> Echinococcus_multilocularis_SL_5_Unique_SL-3,Cestodes_Echinococcus_multilocularis_Emu.SL2b_Reference_SL_Unique_SL-3,Echinococcus_multilocularis_SL_7_Unique_SL-5,Echinococcus_multilocularis_SL_9_Unique_SL-7,Cestodes_Echinococcus_multilocularis_Emu.SL2a_Reference_SL_Unique_SL-7

PARALOGY RELATIONSHIP: Echinococcus_multilocularis_SL_6_Unique_SL-4 <====>
Cestodes_Echinococcus_multilocularis_Emu.SL2c_Reference_SL_Unique_SL-4

PARALOGY RELATIONSHIP: Echinococcus_multilocularis_SL_8_Unique_SL-6 <====>
Cestodes_Echinococcus_multilocularis_Emu.SL2d_Reference_SL_Unique_SL-6

PARALOGY RELATIONSHIP: Echinococcus_multilocularis_SL_6_Unique_SL-4,Cestodes_Echinococcus_multilocularis_Emu.SL2c_Reference_SL_Unique_SL-4 <====> Echinococcus_multilocularis_SL_8_Unique_SL-6,Cestodes_Echinococcus_multilocularis_Emu.SL2d_Reference_SL_Unique_SL-6

PARALOGY RELATIONSHIP: Echinococcus_multilocularis_SL_4_Unique_SL-2,Echinococcus_multilocularis_SL_5_Unique_SL-3,Cestodes_Echinococcus_multilocularis_Emu.SL2b_Reference_SL_Unique_SL-3,Echinococcus_multilocularis_SL_7_Unique_SL-5,Echinococcus_multilocularis_SL_9_Unique_SL-7,Cestodes_Echinococcus_multilocularis_Emu.SL2a_Reference_SL_Unique_SL-7 <====>
Echinococcus_multilocularis_SL_6_Unique_SL-4,Cestodes_Echinococcus_multilocularis_Emu.SL2c_Reference_SL_Unique_SL-4,Echinococcus_multilocularis_SL_8_Unique_SL-6,Cestodes_Echinococcus_multilocularis_Emu.SL2d_Reference_SL_Unique_SL-6

PARALOGY RELATIONSHIP: Hymenolepis_microstoma_SL_1_Unique_SL-16 <====> Hymenolepis_microstoma_SL_3_Unique_SL-43

PARALOGY RELATIONSHIP: Sparganum_proliferum_SL_1_Unique_SL-26 <====> Sparganum_proliferum_SL_3_Unique_SL-26

PARALOGY RELATIONSHIP: Echinococcus_multilocularis_SL_1_Unique_SL-10 <====>
Cestodes_Echinococcus_multilocularis_Emu.SL1_Reference_SL_Unique_SL-10

```
 _ _ _ _ _ _ _ _ _ _ _ _ _

 Species tree
 _ _ _ _ _ _ _ _ _ _ _ _ _

         /-Mco
        |
        |      /-Hmi
      /-|   /-|
     |  |  |   \-Hdi
     |  |  |
     |   \-|      /-Emu
     |     |   /-|
     |     |  |   \-Egr
     |      \-|
     |        |   /-Tso
   /-|         \-|
  |  |           |   /-Tmu
  |  |            \-|
  |  |              |   /-Tsa
  |  |               \-|
  |  |                  \-Tas
  |  |
  |  |   /-Ser
  |   \-|
--|      \-Spr
  |
  |      /-Tre
  |   /-|
  |  |  |   /-Sja
  |  |   \-|
  |  |     |   /-Sma
  |  |      \-|
  |  |        |   /-Sbo
  |  |         \-|
   \-|            \-Sha
     |
     |      /-Fbu
     |   /-|
     |  |  |   /-Fhe
     |  |   \-|
     |  |      \-Fgi
      \-|
        |      /-Ofe
        |   /-|
        |  |   \-Csi
         \-|
           |   /-Phe
            \-|
              |   /-Ssp
               \-|
```

```
                    \-Hsp



_ _ _ _ _ _ _ _ _ _ _ _ _ _

Reconciled tree
_ _ _ _ _ _ _ _ _ _ _ _ _ _



                    /-Phe
                 /-|
                |  |   /-Hsp
                |   \-|
                |      \-Trematoda_Stephanostomum_sp_U83576.1_Reference_SL_Unique_SL-74
              /-|
             |  |      /-Ofe
             |  |   /-|
             |  |  |   \-Clonorchis_sinensis_SL_1_Unique_SL-1
             |   \-|
             |     |   /-Clonorchis_sinensis_SL_2_Unique_SL-32
           /-|      \-|
          |  |         \-Opisthorchis_felineus_SL_1_Unique_SL-17
          |  |
          |  |      /-Ofe
          |  |   /-|
          |  |  |   \-Csi
          |   \-|
          |     |      /-Ssp
          |     |   /-|
          |      \-|   \-Trematoda_Haematolechus_sp_U83578.1_Reference_SL_Unique_SL-73
          |        |
          |         \-Paragonimus_heterotremus_SL_1_Unique_SL-47
          |
          |                  /-Fhe
        /-|               /-|
       |  |              |   \-Fasciola_gigantica_SL_1_Unique_SL-13
       |  |            /-|
       |  |           |  |   /-Fasciola_gigantica_SL_2_Unique_SL-14
       |  |           |   \-|
       |  |         /-|      \-Fasciola_hepatica_SL_1_Unique_SL-13
       |  |        |  |
       |  |        |  |   /-Fgi
       |  |      /-|   \-|
       |  |     |  |      \-Fasciola_hepatica_SL_2_Unique_SL-37
       |  |     |  |
       |  |     |  |   /-Fhe
       |  |   /-|   \-|
     /-|  |  |  |      \-Fasciola_gigantica_SL_3_Unique_SL-35
    |  |  |  |  |
    |  |   \-|  |   /-Fhe
    |  |     |   \-|
    |  |     |      \-Fasciola_gigantica_SL_4_Unique_SL-36
    |  |     |
    |  |      \-Fasciolopsis_buski_SL_1_Unique_SL-38
    |  |
    |  |         /-Fhe
    |  |      /-|
    |  |   /-|   \-Fgi
    |  |  |  |
    |  |  |   \-Fasciolopsis_buski_SL_2_Unique_SL-39
    |   \-|
    |     |      /-Ofe
    |     |   /-|
    |     |  |   \-Csi
    |      \-|
    |        |   /-Phe
    |         \-|
    |           |   /-Ssp
    |            \-|
    |               \-Hsp
    |
    |                           /-Sja
    |                          |
    |                          |         /-Schistosoma_bovis_SL_1_Unique_SL-18
    |                        /-|      /-|
    |                       |  |   /-|   \-Schistosoma_haematobium_SL_2_Unique_SL-20
    |                       |  |  |  |
    |                       |  |  |   \-Schistosoma_mansoni_SL_4_Unique_SL-55
    |                       |   \-|
    |                       |     |      /-Sbo
    |                       |     |   /-|
    |                       |     |  |   \-Sha
    |                       |      \-|
    |                       |        |   /-Schistosoma_mansoni_SL_3_Unique_SL-54
    |                       |         \-|
    |                       |            \-Schistosoma_mansoni_SL_7_Unique_SL-58
    |                       |
    |                       |      /-Sja
    |                       |     |
    |                       |     |                        /-Sha
    |                       |     |                     /-|
    |                       |     |                  /-|   \-Schistosoma_bovis_SL_2_Unique_SL-19
    |                       |     |                 |  |
    |                       |     |                 |   \-Schistosoma_mansoni_SL_1_Unique_SL-19
```

```
   |                       |     |               /-|
   |                       |     |              |  |      /-Sbo
   |                       |     |              |  |   /-|
   |                       |     |              |   \-|   \-Sha
   |                       |     |            /-|     |
   |                       |     |           |  |      \-Trematoda_Schistosoma_mansoni_rajko.90nt.slRNA-31.1.1_Reference_SL_Unique_SL-19
   |                     /-|     |           |  |
   |                    |  |     |           |  |      /-Sbo
   |                    |  |     |           |  |   /-|
   |                    |  |     |         /-|   \-|   \-Sha
   |                    |  |   /-|        |  |     |
   |                    |  |  |  |        |  |      \-Trematoda_Schistosoma_mansoni_rajko.90nt.slRNA-46.1.1_Reference_SL_Unique_SL-19
   |                    |  |  |  |        |  |
   |                    |  |  |  |        |  |      /-Sbo
   |                    |  |  |  |        |  |   /-|
   |                    |  |  |  |      /-|   \-|   \-Sha
   |                    |  |  |  |     |  |     |
   |                    |  |  |  |     |  |      \-Trematoda_Schistosoma_mansoni_rajko.90nt.slRNA-39.1.1_Reference_SL_Unique_SL-19
   |                    |  |  |  |     |  |
   |                    |  |  |  |     |  |      /-Sbo
   |                    |  |  |  |   /-|  |   /-|
   |                    |  |  |  |  |  |   \-|   \-Sha
   |                    |  |  |  |  |  |     |
   |                    |  |  |  |  |  |      \-Trematoda_Schistosoma_mansoni_rajko.90nt.slRNA-5.1.1_Reference_SL_Unique_SL-19
   |                    |  |  |  |  |  |
   |                    |  |  |  |  |  |   /-Sma
   |                    |  |  |  |  |   \-|
   |                    |  |  |   \-|     |   /-Sbo
   |                    |  |  |     |      \-|
   |                    |  |  |     |         \-Schistosoma_haematobium_SL_1_Unique_SL-19
   |                    |  |  |     |
   |                    |  |  |     |      /-Sha
   |                    |   \-|     |   /-|
   |                    |     |     |  |  |   /-Schistosoma_bovis_SL_3_Unique_SL-48
   |                    |     |      \-|   \-|
 /-|                    |     |        |      \-Schistosoma_bovis_SL_4_Unique_SL-49
|  |                    |     |        |
|  |                    |     |         \-Trematoda_Schistosoma_mansoni_rajko.90nt.slRNA-45.1.1_Reference_SL_Unique_SL-75
|  |                    |     |
|  |                    |     |            /-Sma
|  |                  /-|     |         /-|
|  |                 |  |     |        |  |   /-Sbo
|  |                 |  |     |      /-|   \-|
|  |                 |  |     |     |  |      \-Schistosoma_haematobium_SL_3_Unique_SL-50
|  |                 |  |     |     |  |
|  |                 |  |     |     |   \-Schistosoma_japonicum_SL_2_Unique_SL-22
|  |                 |  |     |     |
|  |                 |  |     |   /-|         /-Sbo
|  |                 |  |     |  |  |      /-|
|  |                 |  |     |  |  |     |   \-Sha
|  |                 |  |     |  |  |   /-|
|  |                 |  |     |  |  |  |  |   /-Schistosoma_mansoni_SL_6_Unique_SL-57
|  |                 |  |     |  |   \-|   \-|
|  |                 |  |     |  |     |      \-Trematoda_Schistosoma_mansoni_Smp_rajko_90nt_slRNA-1.1.1_Reference_SL_Unique_SL-57
|  |                 |  |     |  |     |
|  |                 |  |      \-|      \-Schistosoma_japonicum_SL_1_Unique_SL-21
|  |                 |  |        |
|  |                 |  |        |      /-Sma
|  |                 |  |        |   /-|
|  |                 |  |        |  |  |   /-Sbo
|  |                 |  |        |  |   \-|
|  |               /-|  |        |  |      \-Sha
|  |              |  |  |        |  |
|  |              |  |  |         \-|         /-Schistosoma_japonicum_SL_3_Unique_SL-23
|  |              |  |  |           |      /-|
|  |              |  |  |           |     |   \-Schistosoma_japonicum_SL_4_Unique_SL-24
|  |              |  |  |           |   /-|
|  |              |  |  |           |  |  |   /-Schistosoma_japonicum_SL_6_Unique_SL-52
|  |              |  |  |            \-|   \-|
|  |              |  |  |              |      \-Schistosoma_japonicum_SL_7_Unique_SL-53
|  |              |  |  |              |
|  |              |  |  |               \-Schistosoma_japonicum_SL_5_Unique_SL-51
|  |              |  |  |
|  |              |  |  |   /-Sja
|  |              |  |  |  |
|  |            /-|  |   \-|      /-Sbo
|  |           |  |  |     |   /-|
|  |           |  |  |      \-|   \-Sha
|  |           |  |  |        |
|  |           |  |  |         \-Schistosoma_mansoni_SL_2_Unique_SL-25
|  |           |  |  |
|  |           |  |  |   /-Sja
|  |           |  |  |  |
|  |           |  |   \-|      /-Sbo
|  |           |  |     |   /-|
|  |           |  |      \-|   \-Sha
|  |         /-|  |        |
|  |        |  |  |         \-Schistosoma_mansoni_SL_5_Unique_SL-56
|  |        |  |  |
|  |        |  |  |   /-Trichobilharzia_regenti_SL_5_Unique_SL-69
|  |        |  |   \-|
|  |        |  |      \-Trichobilharzia_regenti_SL_7_Unique_SL-71
|  |        |  |
|  |        |  |      /-Sja
|  |        |  |   /-|
|  |        |  |  |  |   /-Sma
|  |      /-|  |  |   \-|
|  |     |  |   \-|     |   /-Sbo
|  |     |  |     |      \-|
|  |     |  |     |         \-Sha
```

```
  |  |     |  |     |
  |  |     |  |      \-Trichobilharzia_regenti_SL_1_Unique_SL-65
  |  |     |  |
  |  |     |  |      /-Sja
  |  |     |  |   /-|
  |  |     |  |  |  |   /-Sma
  |  |     |  |  |   \-|
  |  |   /-|   \-|     |   /-Sbo
  |  |  |  |     |      \-|
  |  |  |  |     |         \-Sha
  |  |  |  |     |
  |  |  |  |      \-Trichobilharzia_regenti_SL_4_Unique_SL-68
  |  |  |  |
  |  |  |  |      /-Sja
  |  |  |  |   /-|
  |  |  |  |  |  |   /-Sma
  |  |  |  |  |   \-|
  |  |  |  |  |     |   /-Sbo
  |   \-|   \-|      \-|
  |     |     |         \-Sha
  |     |     |
  |     |     |   /-Trichobilharzia_regenti_SL_2_Unique_SL-66
  |     |      \-|
  |     |         \-Trichobilharzia_regenti_SL_6_Unique_SL-70
  |     |
  |     |      /-Sja
  |     |   /-|
  |     |  |  |   /-Sma
  |     |  |   \-|
  |      \-|     |   /-Sbo
  |        |      \-|
  |        |         \-Sha
  |        |
  |         \-Trichobilharzia_regenti_SL_3_Unique_SL-67
  |
  |         /-Mco
  |        |
  |        |      /-Hmi
  |      /-|   /-|
  |     |  |  |   \-Hdi
  |     |  |  |
  |     |   \-|      /-Emu
  |     |     |   /-|
  |     |     |  |   \-Egr
  |     |      \-|
--|     |        |   /-Tso
  |   /-|        |  |
  |  |  |         \-|      /-Taenia_asiatica_SL_2_Unique_SL-60
  |  |  |           |   /-|
  |  |  |            \-|   \-Taenia_saginata_SL_2_Unique_SL-60
  |  |  |              |
  |  |  |               \-Taenia_multiceps_SL_4_Unique_SL-61
  |  |  |
  |  |  |   /-Ser
  |  |   \-|
  |  |      \-Spr
  |  |
  |  |         /-Mco
  |  |        |
  |  |        |      /-Hmi
  |  |      /-|   /-|
  |  |     |  |  |   \-Hdi
  |  |     |  |  |
  |  |     |   \-|      /-Emu
  |  |     |     |   /-|
  |  |     |     |  |   \-Egr
  |  |     |     |  |
  |  |     |      \-|      /-Tmu
  |  |   /-|        |   /-|
  |  |  |  |        |  |  |   /-Tsa
  |  |  |  |         \-|   \-|
  |  |  |  |           |      \-Tas
  |  |  |  |           |
  |  |  |  |            \-Taenia_solium_SL_2_Unique_SL-64
  |  |  |  |
  |  |  |  |   /-Ser
  |  |  |   \-|
  |  |  |      \-Spr
  |  |  |
  |  |  |                                    /-Mco
  |  |  |                                   |
  |  |  |                                   |      /-Hmi
  |  |  |                                /-|   /-|
  |  |  |                               |  |  |   \-Hdi
  |  |  |                               |  |  |
  |  |  |                               |   \-|      /-Emu
  |  |  |                               |     |   /-|
  |  |  |                               |     |  |   \-Echinococcus_granulosus_SL_1_Unique_SL-33
  |  |  |                               |      \-|
  |  |  |                               |        |   /-Tso
  |  |  |                             /-|         \-|
  |  |  |                            |  |           |   /-Tmu
  |  |  |                            |  |            \-|
  |  |  |                            |  |              |   /-Tsa
  |  |  |                            |  |               \-|
  |  |  |                            |  |                  \-Tas
  |  |  |                            |  |
  |  |  |                            |  |   /-Ser
  |  |  |                            |   \-|
  |  |  |                            |      \-Spr
```

```
|  |  |                             |
|  |  |                             |         /-Mco
|  |  |                             |        |
|  |  |                             |        |      /-Hmi
|  |  |                             |        |   /-|
|  |  |                             |        |  |   \-Hdi
|  |  |                             |      /-|  |
|  |  |                             |     |  |  |      /-Egr
|  |  |                             |     |  |  |     |
|  |  |                             |     |  |  |     |      /-Echinococcus_multilocularis_SL_4_Unique_SL-2
|  |  |                             |     |  |  |     |     |
|  |  |                             |     |  |  |     |     |         /-Echinococcus_multilocularis_SL_5_Unique_SL-3
|  |  |                             |     |   \-|   /-|   /-|      /-|
|  |  |                             |     |     |  |  |  |  |   /-|   \-Cestodes_Echinococcus_multilocularis_Emu.SL2b_Reference_SL_Unique_SL-3
|  |  |                             |     |     |  |  |  |  |  |  |
|  |  |                             |     |     |  |  |  |   \-|   \-Echinococcus_multilocularis_SL_7_Unique_SL-5
|  |  |                             |     |     |  |  |  |     |
|  |  |                             |     |     |  |  |  |     |   /-Echinococcus_multilocularis_SL_9_Unique_SL-7
|  |  |                             |     |     |  |   \-|      \-|
|  |  |                             |     |     |  |     |         \-Cestodes_Echinococcus_multilocularis_Emu.SL2a_Reference_SL_Unique_SL-7
|  |  |                             |     |     |  |     |
|  |  |                             |     |      \-|     |      /-Echinococcus_multilocularis_SL_6_Unique_SL-4
|  |  |                             |   /-|        |     |   /-|
|  |  |                             |  |  |        |     |  |   \-Cestodes_Echinococcus_multilocularis_Emu.SL2c_Reference_SL_Unique_SL-4
|  |  |                             |  |  |        |      \-|
|  |  |                             |  |  |        |        |   /-Echinococcus_multilocularis_SL_8_Unique_SL-6
|  |  |                             |  |  |        |         \-|
|  |  |                             |  |  |        |            \-Cestodes_Echinococcus_multilocularis_Emu.SL2d_Reference_SL_Unique_SL-6
|  |  |                             |  |  |        |
|  |  |                             |  |  |        |   /-Tso
|  |  |                             |  |  |         \-|
|  |  |                             |  |  |           |   /-Tmu
|  |  |                           /-|  |  |            \-|
|  |  |                          |  |  |  |              |   /-Tsa
|  |  |                          |  |  |  |               \-|
|  |  |                          |  |  |  |                  \-Tas
|  |  |                          |  |  |  |
|  |  |                          |  |  |  |   /-Ser
|  |  |                          |  |  |   \-|
|  |  |                          |  |  |      \-Spr
|  |  |                          |  |  |
|  |  |                          |  |  |               /-Mco
|  |  |                          |  |  |              |
|  |  |                          |  |  |              |         /-Emu
|  |  |                          |  |  |              |      /-|
|  |  |                          |  |  |              |     |   \-Egr
|  |  |                          |  |  |            /-|   /-|
|  |  |                          |  |  |           |  |  |  |   /-Tso
|  |  |                          |  |  |           |  |  |   \-|
|  |  |                          |  |  |           |  |  |     |   /-Tmu
|  |  |                          |  |  |           |  |  |      \-|
|  |  |                          |  |  |           |   \-|        |   /-Tsa
|  |  |                          |  |  |           |     |         \-|
|  |  |                          |  |  |           |     |            \-Tas
|  |  |                          |  |  |         /-|     |
 \-|  |                          |  |  |        |  |     |   /-Hymenolepis_diminuta_SL_1_Unique_SL-15
   |  |                          |  |  |        |  |      \-|
   |  |                          |  |  |        |  |        |   /-Hymenolepis_microstoma_SL_1_Unique_SL-16
   |  |                          |  |  |        |  |         \-|
   |  |                          |  |  |        |  |            \-Hymenolepis_microstoma_SL_3_Unique_SL-43
   |  |                          |  |  |        |  |
   |  |                          |  |  |        |  |   /-Ser
   |  |                          |  |  |        |   \-|
   |  |                          |  |  |        |      \-Spr
   |  |                          |  |  |        |
   |  |                          |  |  |        |               /-Hmi
   |  |                          |  |  |        |            /-|
   |  |                          |  |  |        |           |   \-Hdi
   |  |                          |  |  |        |           |
   |  |                          |  |  |        |         /-|      /-Emu
   |  |                          |  |  |        |        |  |   /-|
   |  |                          |  |  |        |        |  |  |   \-Egr
   |  |                          |  |  |        |        |   \-|
   |  |                          |  |  |        |        |     |   /-Tso
   |  |                          |  |  |        |        |      \-|
   |  |                          |   \-|      /-|      /-|        |   /-Tmu
   |  |                          |     |     |  |     |  |         \-|
   |  |                          |     |     |  |     |  |           |   /-Tsa
   |  |                          |     |     |  |     |  |            \-|
   |  |                          |     |     |  |     |  |               \-Tas
   |  |                          |     |     |  |     |  |
   |  |                          |     |     |  |     |   \-Mesocestoides_corti_SL_1_Unique_SL-45
   |  |                          |     |     |  |   /-|
   |  |                          |     |     |  |  |  |         /-Sparganum_proliferum_SL_2_Unique_SL-27
   |  |                          |     |     |  |  |  |      /-|
   |  |                          |     |     |  |  |  |     |   \-Spirometra_erinaceieuropaei_SL_2_Unique_SL-27
   |  |                          |     |     |  |  |  |   /-|
   |  |                          |     |     |  |  |  |  |  |   /-Spr
   |  |                          |     |     |  |  |  |  |   \-|
   |  |                          |     |     |  |  |   \-|      \-Spirometra_erinaceieuropaei_SL_3_Unique_SL-59
   |  |                          |     |     |  |  |     |
   |  |                          |     |     |  |  |     |   /-Spirometra_erinaceieuropaei_SL_1_Unique_SL-26
   |  |                          |     |     |  |  |      \-|
   |  |                          |     |     |  |  |        |   /-Sparganum_proliferum_SL_1_Unique_SL-26
   |  |                          |     |     |  |  |         \-|
   |  |                          |     |     |  |  |            \-Sparganum_proliferum_SL_3_Unique_SL-26
   |  |                          |     |     |   \-|
   |  |                          |     |     |     |            /-Hmi
   |  |                          |     |     |     |         /-|
   |  |                          |     |     |     |        |   \-Hdi
```

```
|  |                          |     |     |     |        |
|  |                          |     |     |     |      /-|      /-Emu
|  |                          |     |     |     |     |  |   /-|
|  |                        /-|     |   /-|     |     |  |  |   \-Egr
|  |                       |  |     |  |  |     |     |   \-|
|  |                       |  |     |  |  |     |     |     |   /-Tso
|  |                       |  |     |  |  |     |     |      \-|
|  |                       |  |     |  |  |     |   /-|        |   /-Tmu
|  |                       |  |     |  |  |     |  |  |         \-|
|  |                       |  |     |  |  |     |  |  |           |   /-Tsa
|  |                       |  |     |  |  |     |  |  |            \-|
|  |                       |  |     |  |  |      \-|  |               \-Tas
|  |                       |  |     |  |  |        |  |
|  |                       |  |     |  |  |        |   \-Mesocestoides_corti_SL_2_Unique_SL-46
|  |                       |  |     |  |  |        |
|  |                       |  |     |  |  |        |   /-Ser
|  |                       |  |     |  |  |         \-|
|  |                       |  |     |  |  |            \-Spr
|  |                       |  |     |  |  |
|  |                       |  |     |  |  |      /-Mco
|  |                       |  |     |  |  |     |
|  |                       |  |     |  |  |     |         /-Emu
|  |                       |  |     |  |  |     |      /-|
|  |                       |  |     |  |  |     |     |   \-Egr
|  |                       |  |     |  |  |   /-|     |
|  |                       |  |     |  |  |  |  |   /-|      /-Tmu
|  |                       |  |     |  |  |  |  |  |  |   /-|
|  |                       |  |     |  |  |  |  |  |  |  |  |   /-Tas
|  |                       |  |      \-|  |  |  |  |   \-|   \-|
|  |                       |  |        |  |  |   \-|     |      \-Taenia_saginata_SL_3_Unique_SL-62
|  |                       |  |        |  |  |     |     |
|  |                       |  |        |   \-|     |      \-Taenia_solium_SL_1_Unique_SL-63
|  |                       |  |        |     |     |
|  |                       |  |        |     |     |   /-Hymenolepis_diminuta_SL_3_Unique_SL-41
|  |                       |  |        |     |      \-|
|  |                       |  |        |     |         \-Hymenolepis_microstoma_SL_2_Unique_SL-42
|  |                       |  |        |     |
|  |                       |  |        |     |   /-Ser
|  |                       |  |        |      \-|
|  |                       |  |        |         \-Spr
|  |                       |  |        |
|  |                       |  |        |      /-Mco
|  |                       |  |        |     |
|  |                       |  |        |     |         /-Emu
|  |                       |  |        |     |      /-|
|  |                       |  |        |     |     |   \-Egr
|  |                       |  |        |   /-|   /-|
|  |                       |  |        |  |  |  |  |   /-Tso
|  |                       |  |        |  |  |  |   \-|
|  |                       |  |        |  |  |  |     |   /-Tmu
|  |                       |  |        |  |  |  |      \-|
|  |                       |  |        |  |   \-|        |   /-Tsa
|  |                       |  |        |  |     |         \-|
|  |                       |  |         \-|     |            \-Tas
|  |                       |  |           |     |
|  |                       |  |           |     |   /-Hymenolepis_diminuta_SL_2_Unique_SL-40
|  |                       |  |           |      \-|
|  |                       |  |           |         \-Hymenolepis_microstoma_SL_4_Unique_SL-44
|  |                     /-|  |           |
|  |                    |  |  |           |   /-Ser
|  |                    |  |  |            \-|
|  |                    |  |  |               \-Spr
|  |                    |  |  |
|  |                    |  |  |      /-Mco
 \-|                    |  |  |     |
   |                    |  |  |     |      /-Hmi
   |                    |  |  |   /-|   /-|
   |                    |  |  |  |  |  |   \-Hdi
   |                    |  |  |  |  |  |
   |                    |  |  |  |   \-|      /-Emu
   |                    |  |  |  |     |   /-|
   |                    |  |  |  |     |  |   \-Echinococcus_granulosus_SL_2_Unique_SL-34
   |                    |  |  |  |      \-|
   |                    |  |  |  |        |   /-Tso
   |                    |  |   \-|         \-|
   |                    |  |     |           |   /-Tmu
   |                    |  |     |            \-|
   |                    |  |     |              |   /-Tsa
   |                    |  |     |               \-|
   |                    |  |     |                  \-Tas
   |                    |  |     |
   |                    |  |     |   /-Ser
   |                    |  |      \-|
   |                    |  |         \-Spr
   |                    |  |
   |                    |  |      /-Mco
   |                    |  |     |
   |                    |  |     |      /-Hmi
   |                    |  |     |   /-|
   |                    |  |     |  |   \-Hdi
   |                    |  |     |  |
   |                    |  |   /-|  |      /-Emu
   |                    |  |  |  |  |   /-|
   |                    |  |  |  |  |  |   \-Egr
   |                    |  |  |  |  |  |
   |                    |  |  |  |  |  |                  /-Tmu
   |                    |  |  |  |  |  |               /-|
   |                    |  |  |   \-|  |              |  |   /-Tsa
   |                    |  |  |     |  |            /-|   \-|
   |                    |  |  |     |  |           |  |      \-Taenia_asiatica_SL_1_Unique_SL-28
```

```
|                    |  |  |     |  |           |  |
|                    |  |  |     |  |         /-|   \-Cestodes_Taenia_solium_AJ428456.1_Reference_SL_Unique_SL-28
|                    |  |  |     |  |        |  |
|                    |  |  |     |  |        |  |   /-Tso
|                  /-|  |  |     |  |        |  |  |
|                 |  |  |  |     |  |        |   \-|      /-Tsa
|                 |  |  |  |      \-|        |     |   /-|
|                 |  |  |  |        |      /-|      \-|   \-Tas
|                 |  |  |  |        |     |  |        |
|                 |  |  |  |        |     |  |         \-Taenia_multiceps_SL_2_Unique_SL-29
|                 |  |  |  |        |     |  |
|                 |  |  |  |        |     |  |   /-Tso
|                 |  |  |  |        |     |  |  |
|                 |  |   \-|        |     |   \-|      /-Tsa
|                 |  |     |        |   /-|     |   /-|
|                 |  |     |        |  |  |      \-|   \-Tas
|                 |  |     |        |  |  |        |
|                 |  |     |        |  |  |         \-Taenia_multiceps_SL_1_Unique_SL-28
|                 |  |     |        |  |  |
|                 |  |     |        |  |  |   /-Tso
|                 |  |     |        |  |  |  |
|                 |  |     |         \-|   \-|      /-Tsa
|                 |  |     |           |     |   /-|
|                 |  |     |           |      \-|   \-Tas
|                 |  |     |           |        |
|                 |  |     |           |         \-Taenia_multiceps_SL_3_Unique_SL-30
|                 |  |     |           |
|                 |  |     |           |   /-Tso
|                 |  |     |            \-|
|                 |  |     |              |   /-Tmu
|                 |  |     |               \-|
|                 |  |     |                 |   /-Tas
|                 |  |     |                  \-|
|                 |  |     |                     \-Taenia_saginata_SL_1_Unique_SL-31
|                 |  |     |
|                 |  |     |   /-Ser
|                 |  |      \-|
|                 |  |         \-Spr
|               /-|  |
|              |  |  |      /-Mco
|              |  |  |     |
|              |  |  |     |      /-Hmi
|              |  |  |   /-|   /-|
|              |  |  |  |  |  |   \-Hdi
|              |  |  |  |  |  |
|              |  |  |  |   \-|      /-Emu
|              |  |  |  |     |   /-|
|              |  |  |  |     |  |   \-Cestodes_Echinococcus_granulosus_AJ292377.1_Reference_SL_Unique_SL-72
|              |  |  |  |      \-|
|              |  |  |  |        |   /-Tso
|              |  |   \-|         \-|
|              |  |     |           |   /-Tmu
|              |  |     |            \-|
|              |  |     |              |   /-Tsa
|              |  |     |               \-|
|              |  |     |                  \-Tas
|              |  |     |
|              |  |     |   /-Ser
|              |  |      \-|
|              |  |         \-Spr
|              |  |
|              |  |      /-Mco
|              |  |     |
|              |  |     |      /-Hmi
|              |  |   /-|   /-|
|              |  |  |  |  |   \-Hdi
|            /-|  |  |  |  |
|           |  |  |  |   \-|      /-Egr
|           |  |  |  |     |   /-|
|           |  |  |  |     |  |   \-Echinococcus_multilocularis_SL_2_Unique_SL-11
|           |  |  |  |      \-|
|           |  |  |  |        |   /-Tso
|           |  |   \-|         \-|
|           |  |     |           |   /-Tmu
|           |  |     |            \-|
|           |  |     |              |   /-Tsa
|           |  |     |               \-|
|           |  |     |                  \-Tas
|           |  |     |
|           |  |     |   /-Ser
|           |  |      \-|
|           |  |         \-Spr
|           |  |
|           |  |      /-Mco
|           |  |     |
|           |  |     |      /-Hmi
|           |  |   /-|   /-|
|           |  |  |  |  |   \-Hdi
|           |  |  |  |  |
|           |  |  |   \-|      /-Egr
|           |  |  |     |   /-|
|         /-|  |  |     |  |   \-Echinococcus_multilocularis_SL_3_Unique_SL-12
|        |  |  |  |      \-|
|        |  |  |  |        |   /-Tso
|        |  |   \-|         \-|
|        |  |     |           |   /-Tmu
|        |  |     |            \-|
|        |  |     |              |   /-Tsa
|        |  |     |               \-|
|        |  |     |                  \-Tas
```

```
|        |  |     |
|        |  |     |   /-Ser
|        |  |      \-|
|        |  |         \-Spr
|        |  |
|        |  |      /-Mco
|        |  |     |
|        |  |     |      /-Hmi
|        |  |   /-|   /-|
|        |  |  |  |  |   \-Hdi
|        |  |  |  |  |
|        |  |  |   \-|      /-Egr
|        |  |  |     |   /-|
|        |  |  |     |  |   \-Echinococcus_multilocularis_SL_10_Unique_SL-8
|      /-|  |  |      \-|
|     |  |  |  |        |   /-Tso
|     |  |   \-|         \-|
|     |  |     |           |   /-Tmu
|     |  |     |            \-|
|     |  |     |              |   /-Tsa
|     |  |     |               \-|
|     |  |     |                  \-Tas
|     |  |     |
|     |  |     |   /-Ser
|     |  |      \-|
|     |  |         \-Spr
|     |  |
|     |  |      /-Mco
|     |  |     |
|     |  |     |      /-Hmi
|     |  |   /-|   /-|
|     |  |  |  |  |   \-Hdi
|     |  |  |  |  |
|     |  |  |   \-|      /-Egr
|     |  |  |     |   /-|
|     |  |  |     |  |   \-Cestodes_Echinococcus_multilocularis_AJ292365.1_Reference_SL_Unique_SL-8
|     |  |  |      \-|
|   /-|  |  |        |   /-Tso
|  |  |   \-|         \-|
|  |  |     |           |   /-Tmu
|  |  |     |            \-|
|  |  |     |              |   /-Tsa
|  |  |     |               \-|
|  |  |     |                  \-Tas
|  |  |     |
|  |  |     |   /-Ser
|  |  |      \-|
|  |  |         \-Spr
|  |  |
|  |  |      /-Mco
|  |  |     |
|  |  |     |      /-Hmi
|  |  |   /-|   /-|
|  |  |  |  |  |   \-Hdi
|  |  |  |  |  |
|  |  |  |   \-|      /-Egr
|  |  |  |     |   /-|
|  |  |  |     |  |  |   /-Echinococcus_multilocularis_SL_1_Unique_SL-10
|  |  |  |     |  |   \-|
|  |  |  |      \-|      \-Cestodes_Echinococcus_multilocularis_Emu.SL1_Reference_SL_Unique_SL-10
 \-|  |  |        |
   |   \-|        |   /-Tso
   |     |         \-|
   |     |           |   /-Tmu
   |     |            \-|
   |     |              |   /-Tsa
   |     |               \-|
   |     |                  \-Tas
   |     |
   |     |   /-Ser
   |      \-|
   |         \-Spr
   |
   |      /-Mco
   |     |
   |     |      /-Hmi
   |   /-|   /-|
   |  |  |  |   \-Hdi
   |  |  |  |
   |  |   \-|      /-Egr
   |  |     |   /-|
   |  |     |  |   \-Echinococcus_multilocularis_SL_11_Unique_SL-9
   |  |      \-|
   |  |        |   /-Tso
    \-|         \-|
      |           |   /-Tmu
      |            \-|
      |              |   /-Tsa
      |               \-|
      |                  \-Tas
      |
      |   /-Ser
       \-|
          \-Spr
```
