# Supplementary material for: Evolution of SL-RNA Genes and Their Splicing Targets in Parasitic Flatworms
Source: Mol Biol Evol. 2025 Sep 23;42(11):msaf228. doi: 10.1093/molbev/msaf228 (PMC12582326; doi:10.1093/molbev/msaf228)
Supplement: msaf228_Supplementary_Data [file msaf228_supplementary_data.zip › Supplementary File 6 - 08082025.pdf]

Distribution of the number of genes with SL in each species from randomly sampled reads

A

Distribution of the number of genes with SL, generated from 5 x 10^5 randomly selected reads.

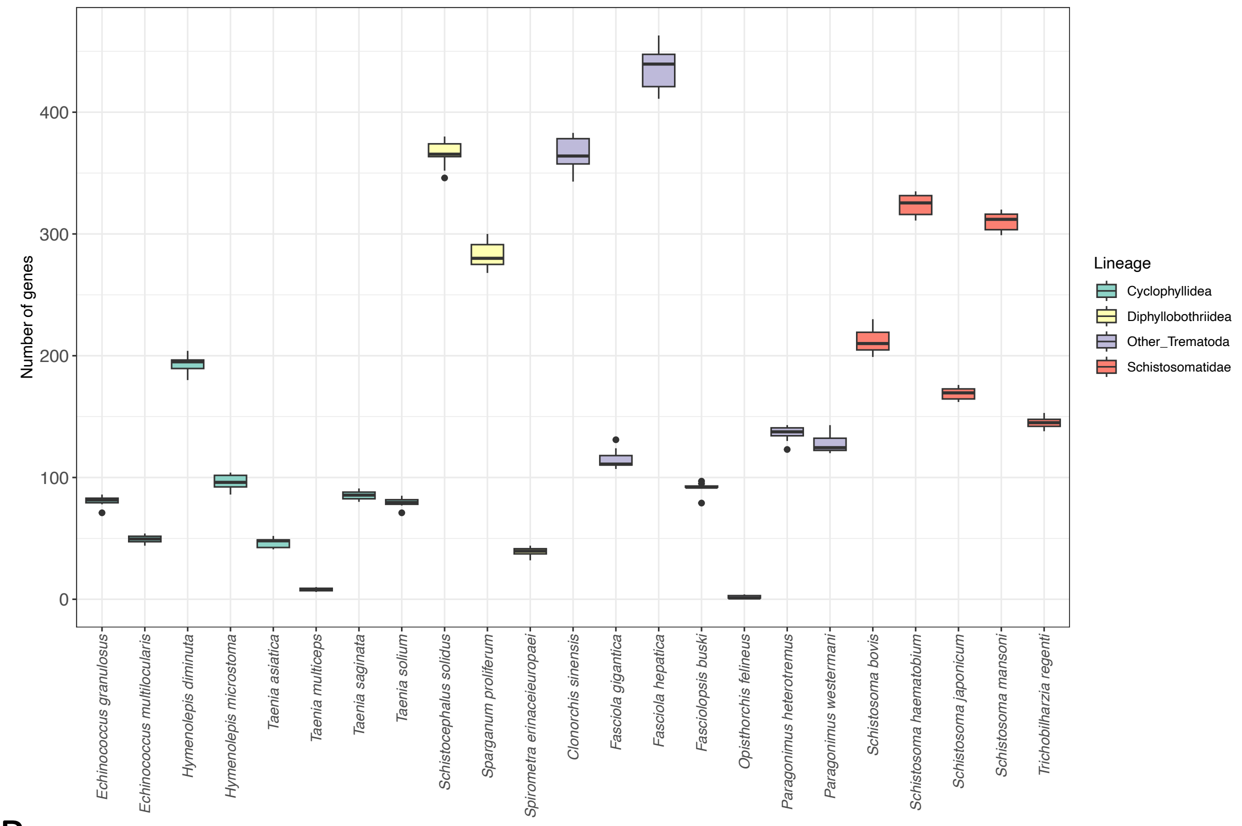

B

Distribution of the number of genes with SL, generated from 1500 randomly selected SL bearing reads.

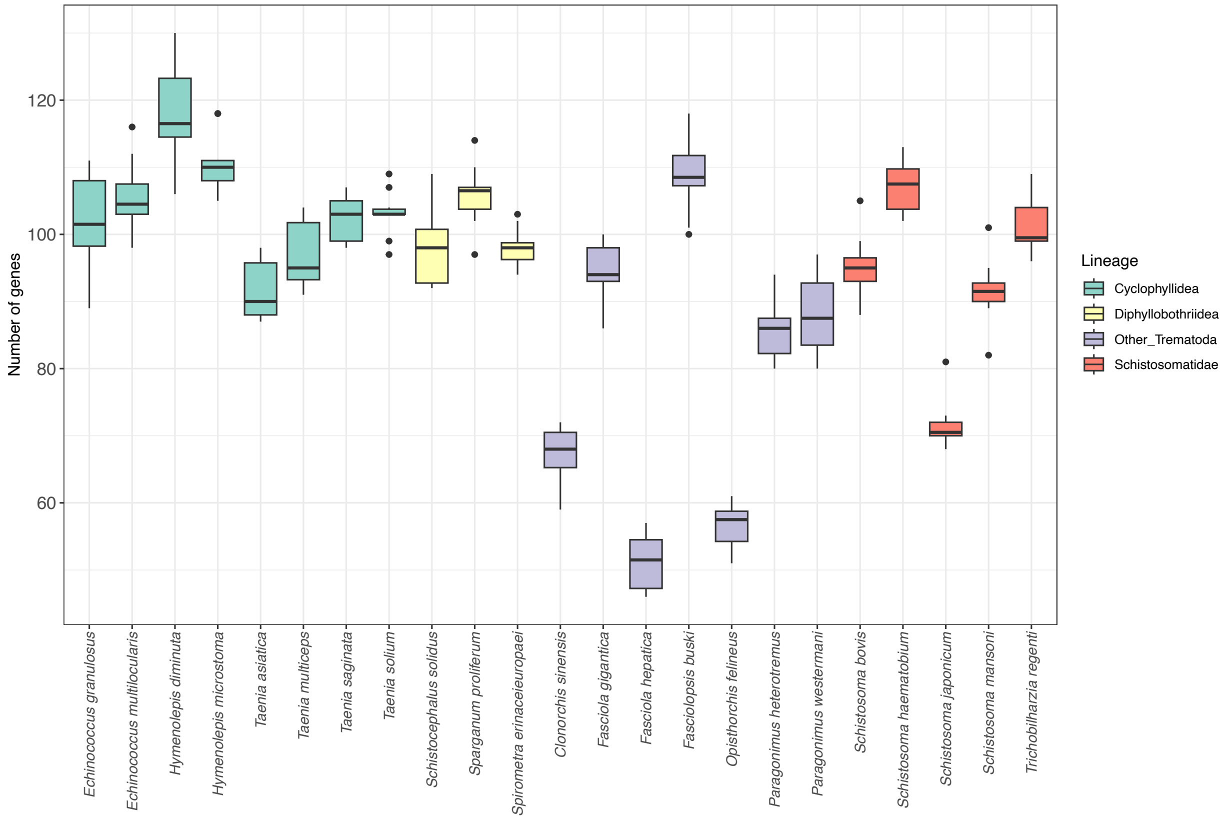

Supplementary File 6. Boxplots and rarefaction curves showing the estimated number of genes undergoing SL trans-splicing per species, based on random subsampling of reads (either total reads or SL-bearing reads, as indicated). For each species, 5 million reads were randomly subsampled, mapped to the genes, and the percentage of genes undergoing SLTS was estimated. Following the same approach, 1,500 SL-bearing reads were also randomly subsampled. Each subsampling was repeated 10 times per species to generate a distribution of the number of genes undergoing SLTS.

Rarefaction curves of SL-detected genes relative to the number of randomly selected reads

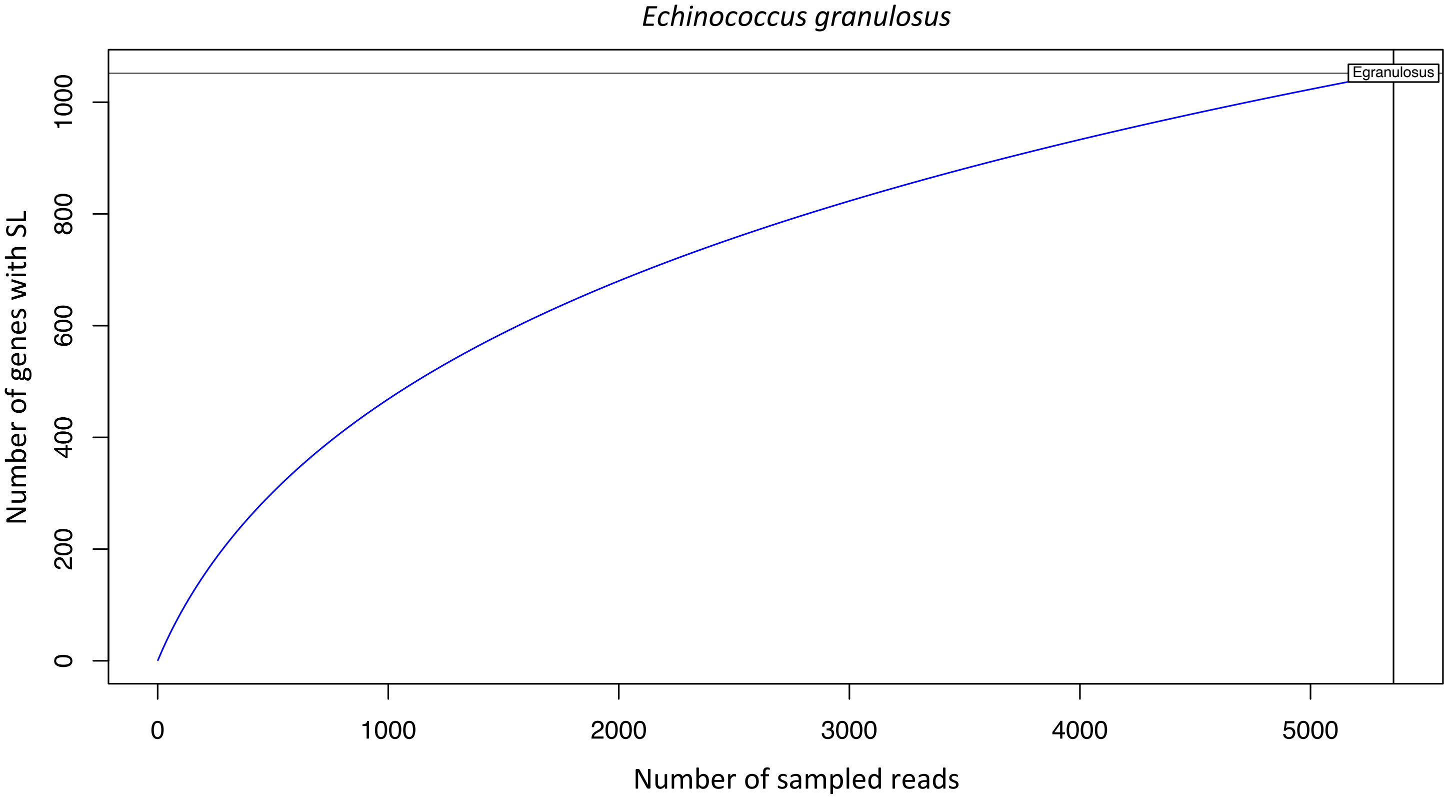

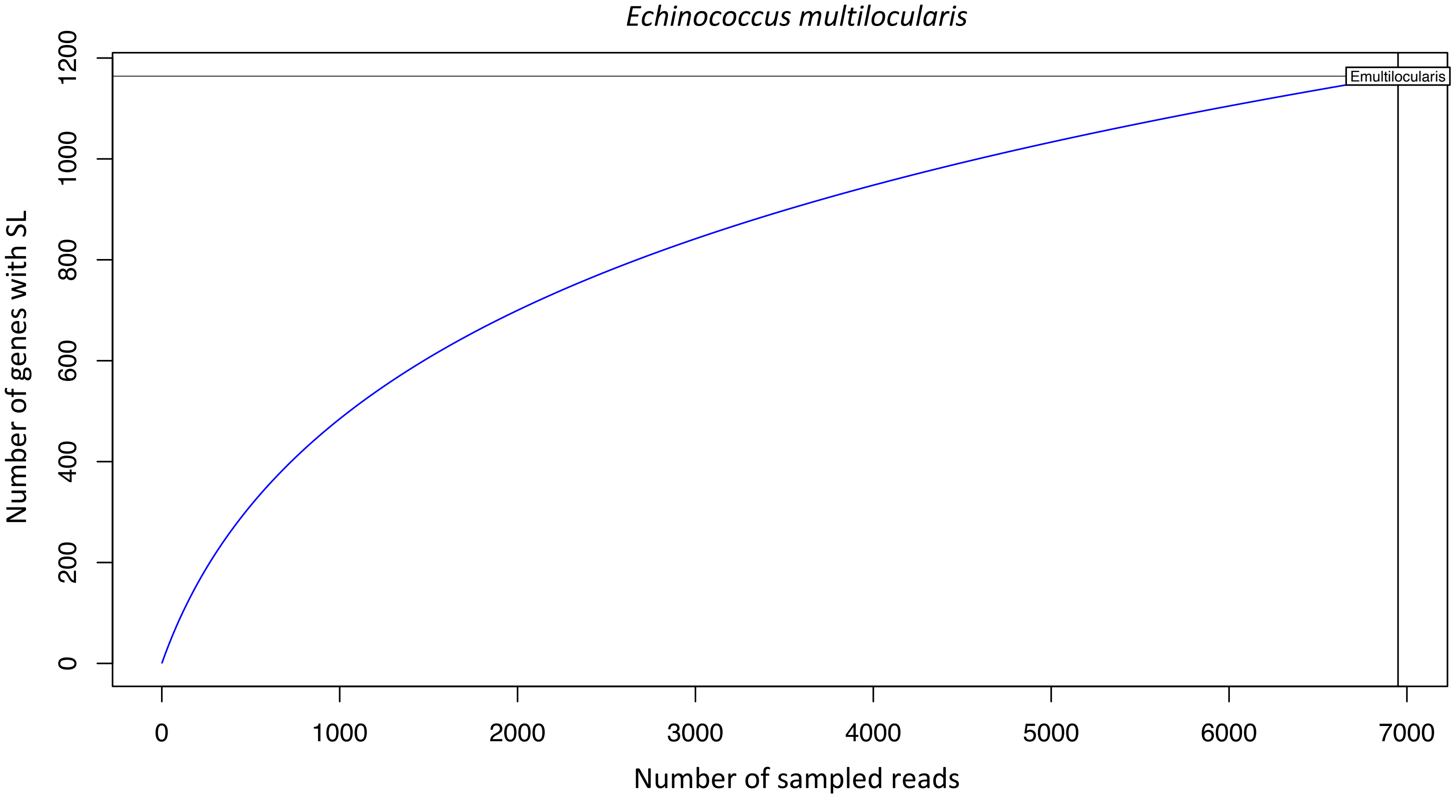

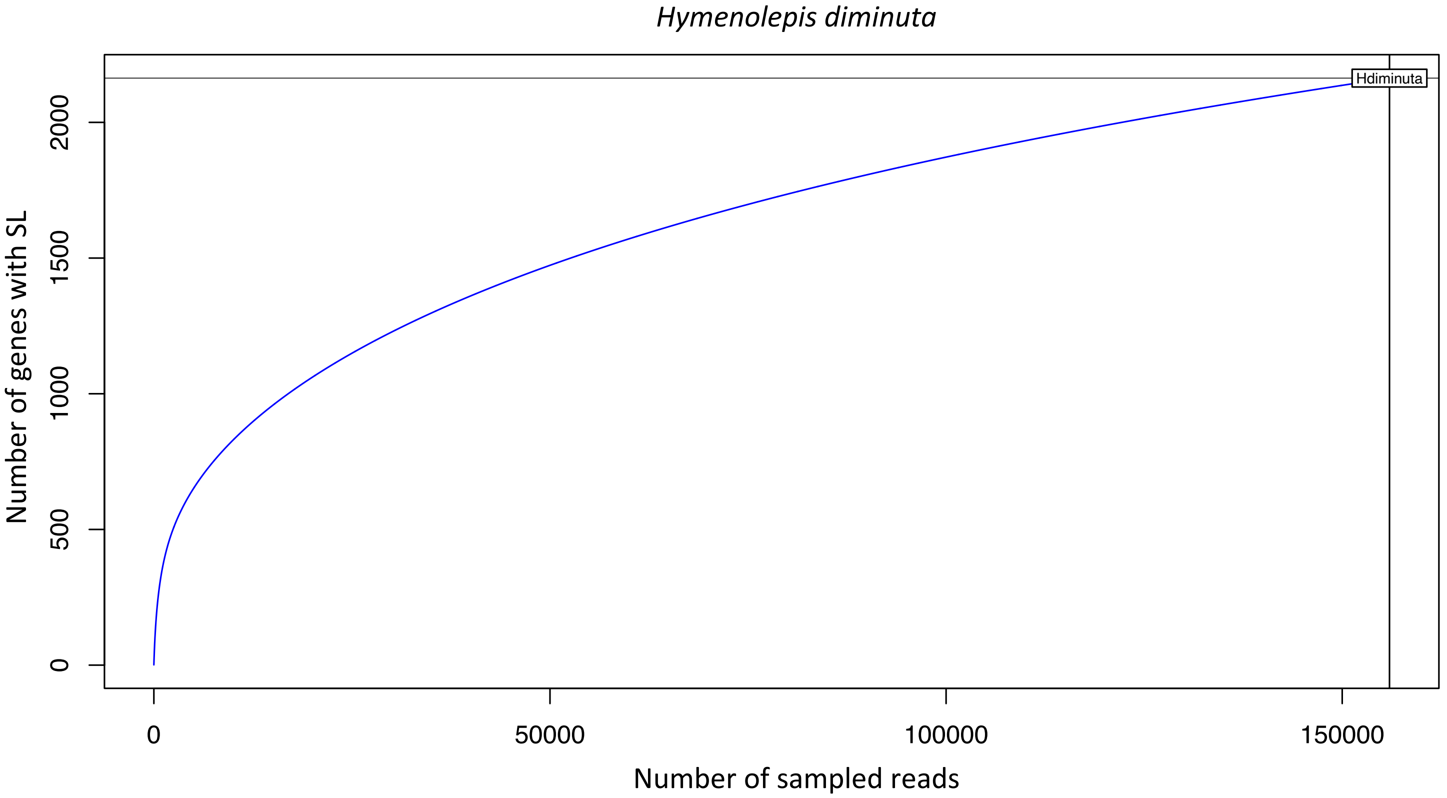
Hymenolepis diminuta
Hdiminuta
Number of genes with SL
0
500
1000
1500
2000
0
50000
100000
150000
Number of sampled reads

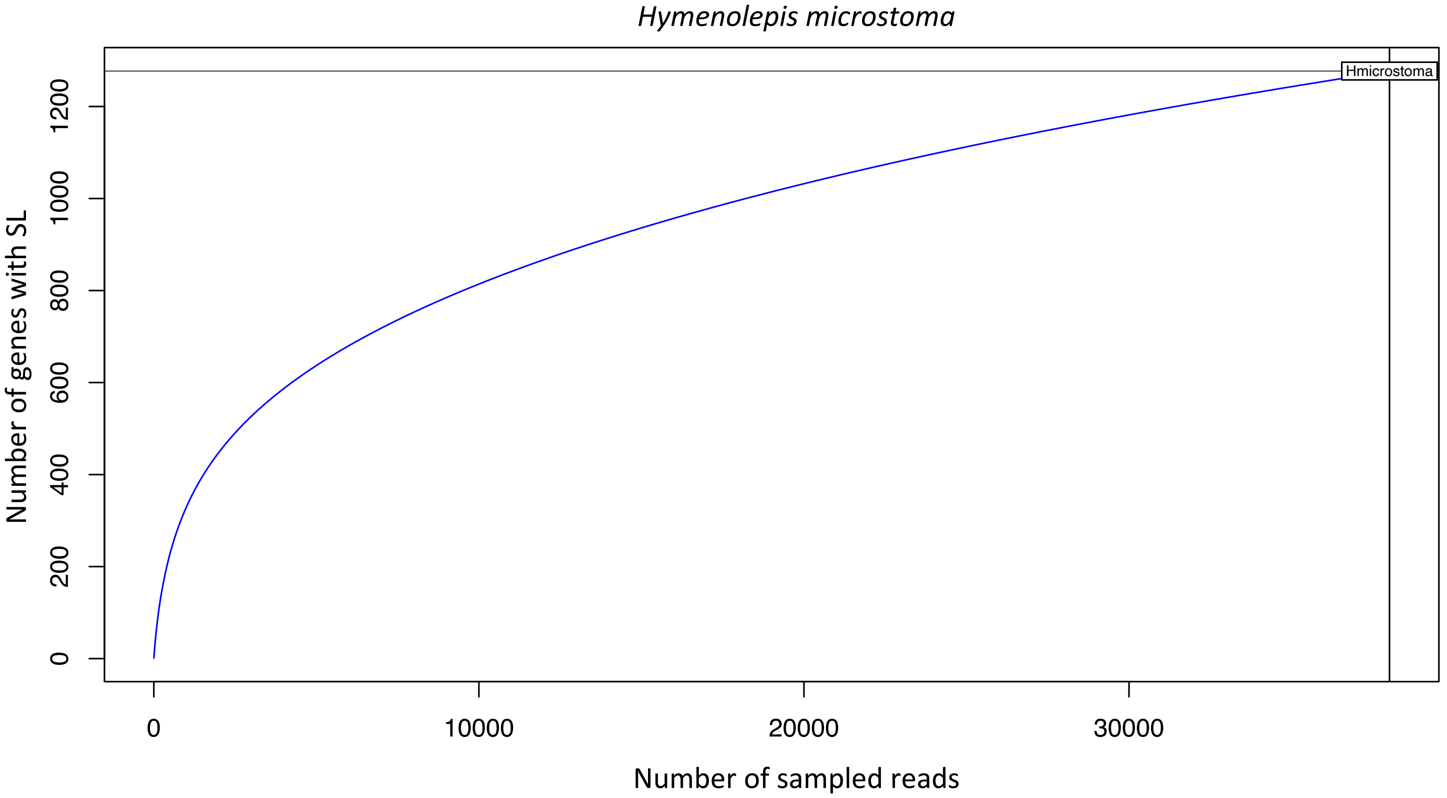
Hymenolepis microstoma
Hmicrostoma
Number of genes with SL
0
200
400
600
800
1000
1200
0
10000
20000
30000
Number of sampled reads

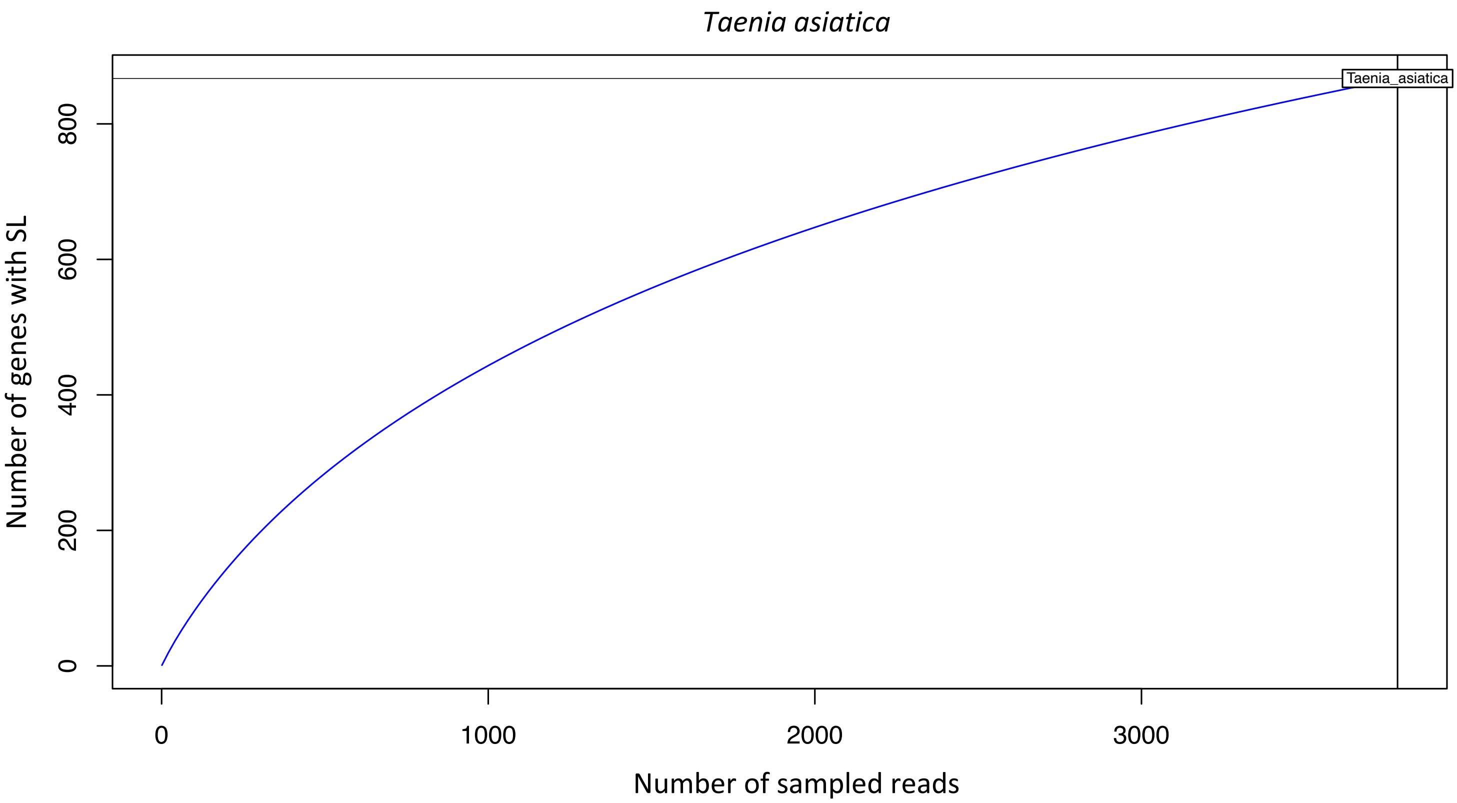
Taenia asiatica
Taenia_asiatica
Number of genes with SL
0
200
400
600
800
0
1000
2000
3000
Number of sampled reads

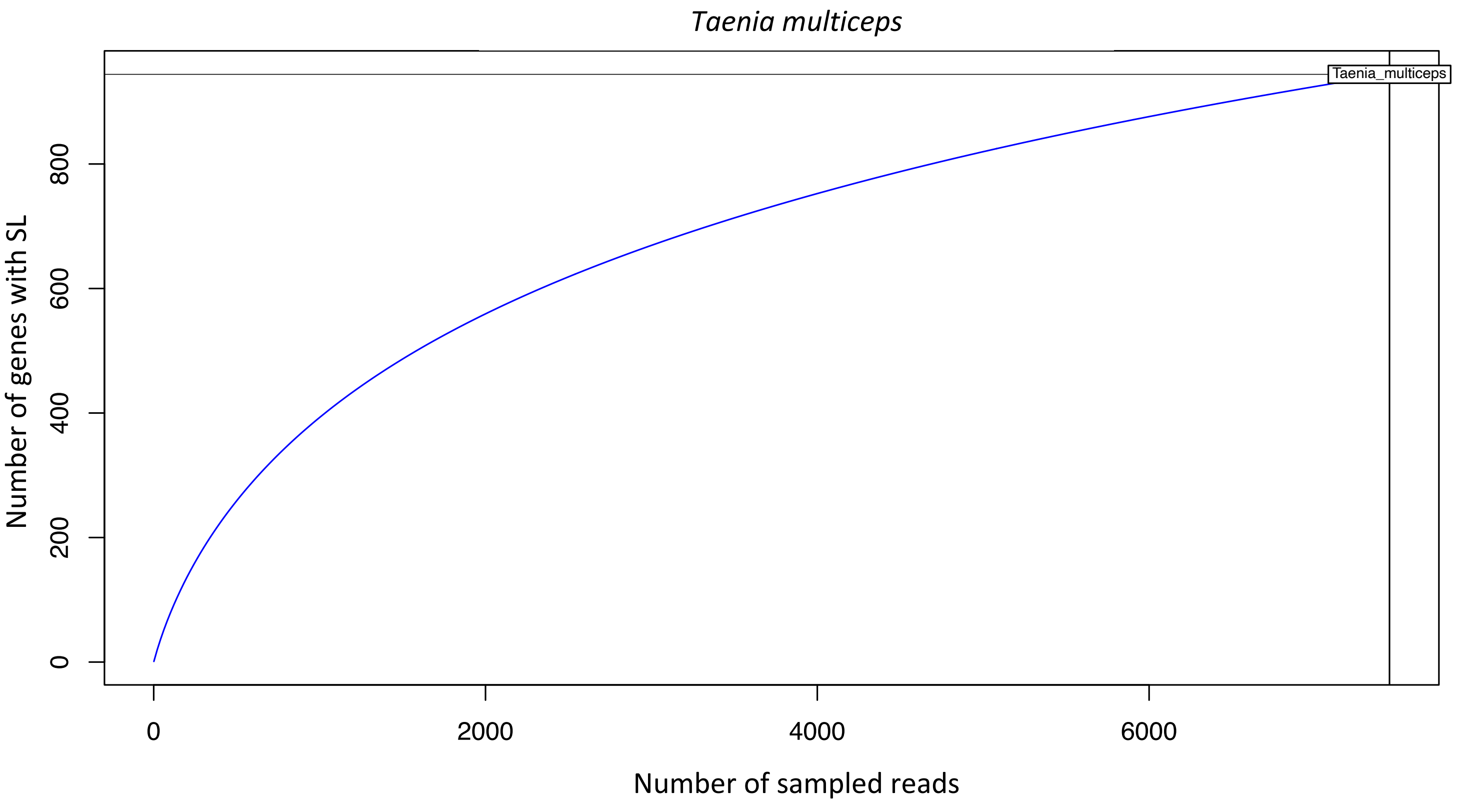
Taenia multiceps
Taenia_multiceps
Number of genes with SL
0
200
400
600
800
0
2000
4000
6000
Number of sampled reads

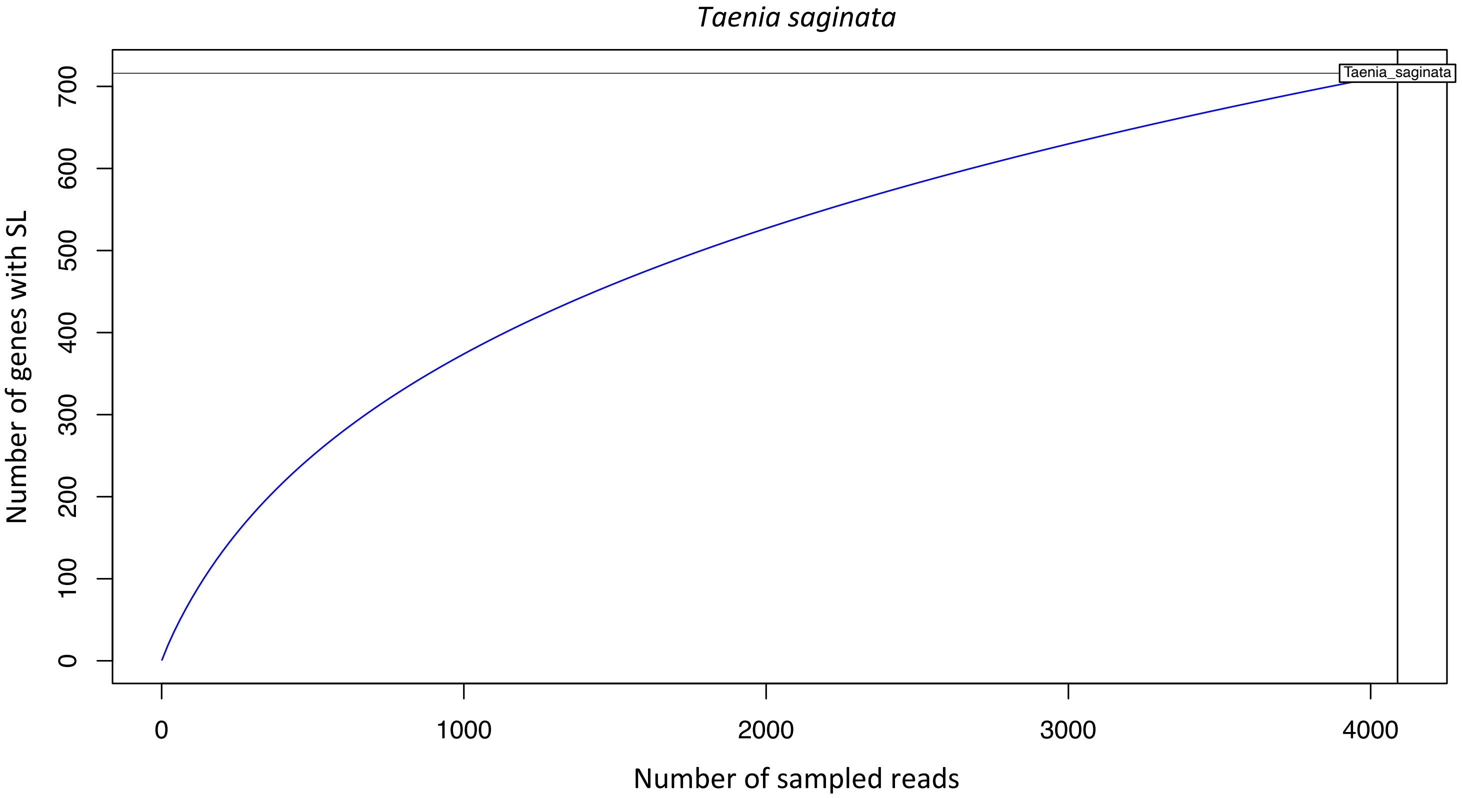
Taenia saginata
Taenia_saginata
Number of genes with SL
0
100
200
300
400
500
600
700
0
1000
2000
3000
4000
Number of sampled reads

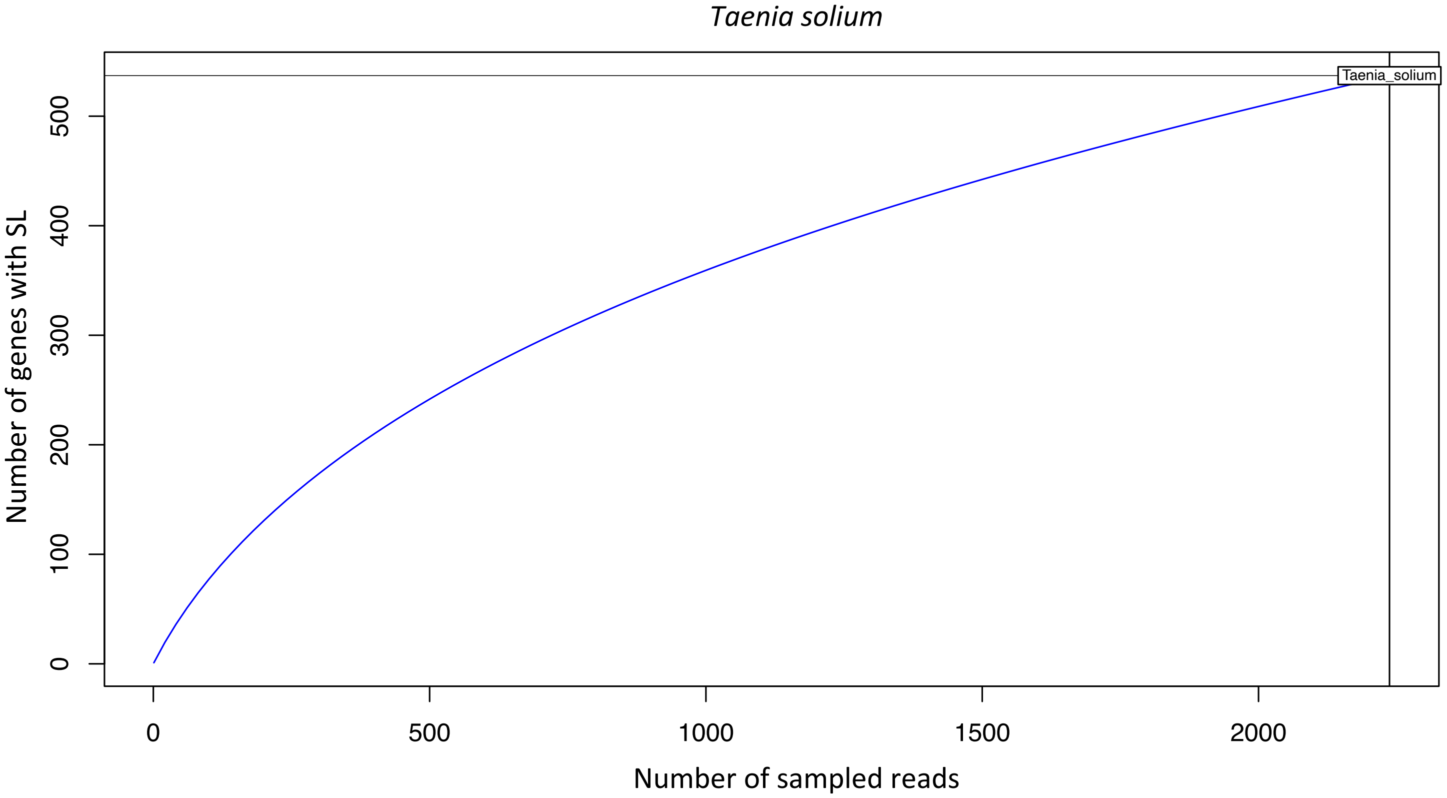
Taenia solium
Taenia_solium
Number of genes with SL
0
100
200
300
400
500
0
500
1000
1500
2000
Number of sampled reads

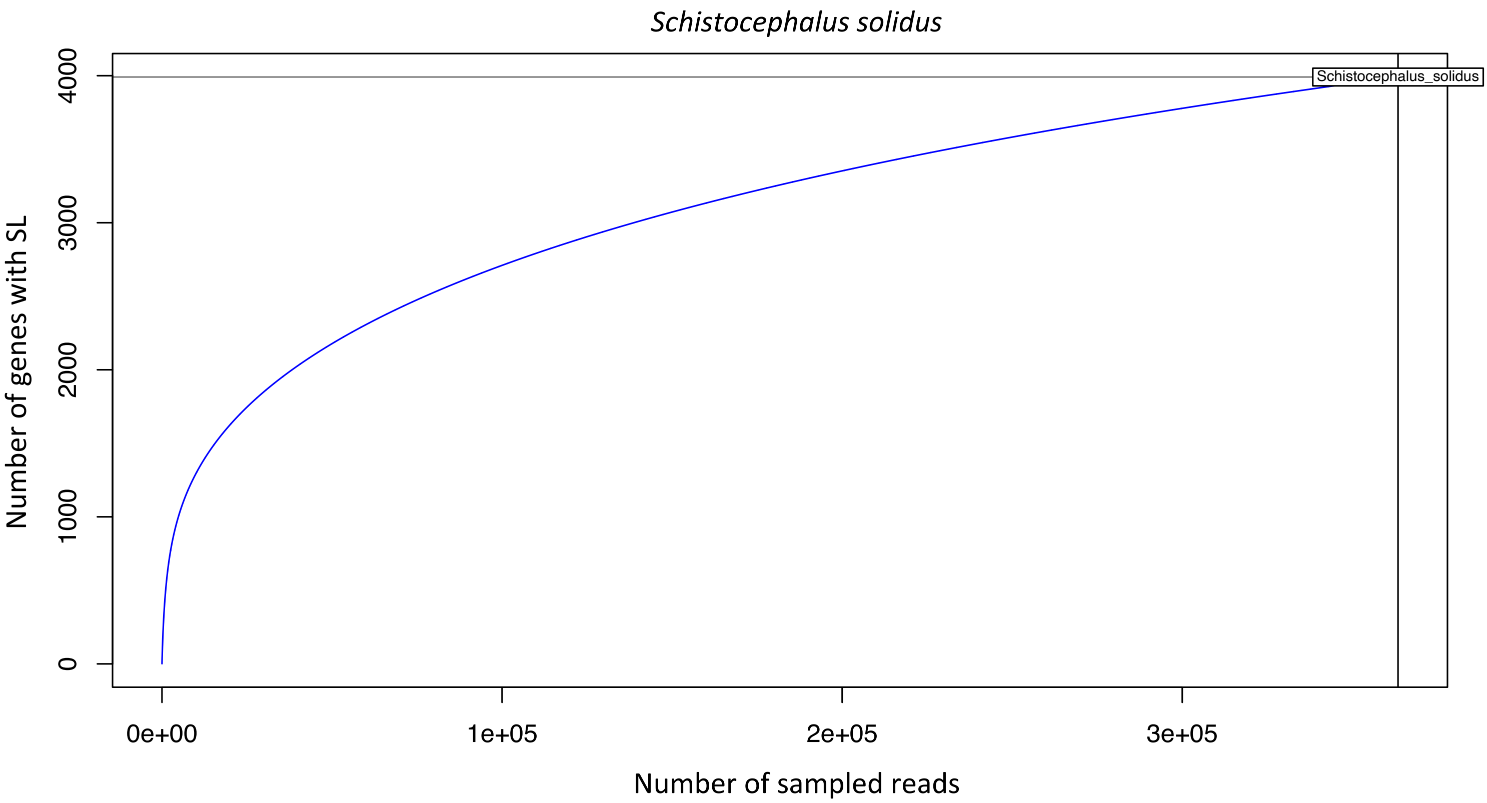
Schistocephalus solidus
Schistocephalus_solidus
Number of genes with SL
0
1000
2000
3000
4000
0e+00
1e+05
2e+05
3e+05
Number of sampled reads

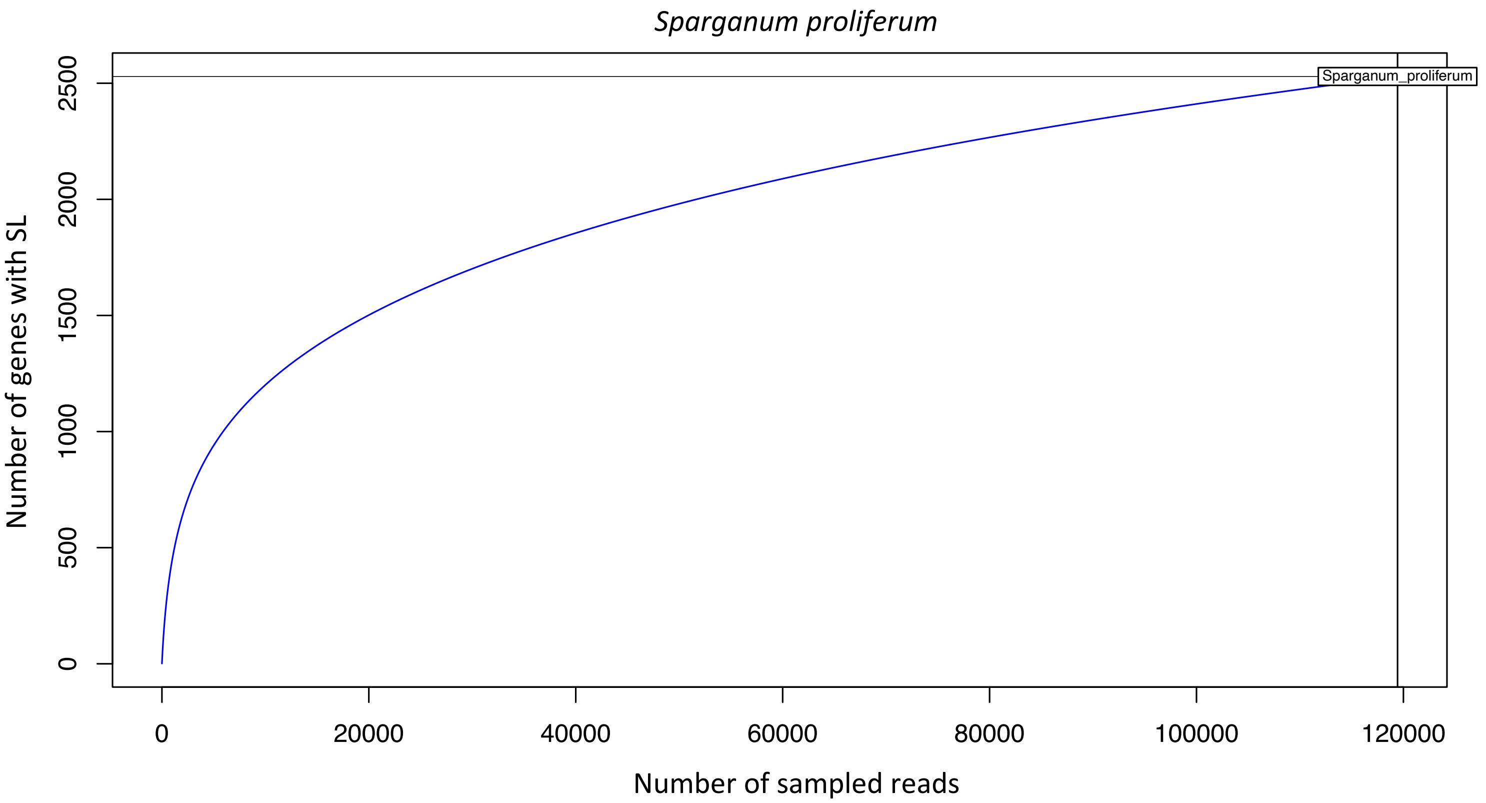
Sparganum proliferum
Sparganum_proliferum
Number of genes with SL
0
500
1000
1500
2000
2500
0
20000
40000
60000
80000
100000
120000
Number of sampled reads

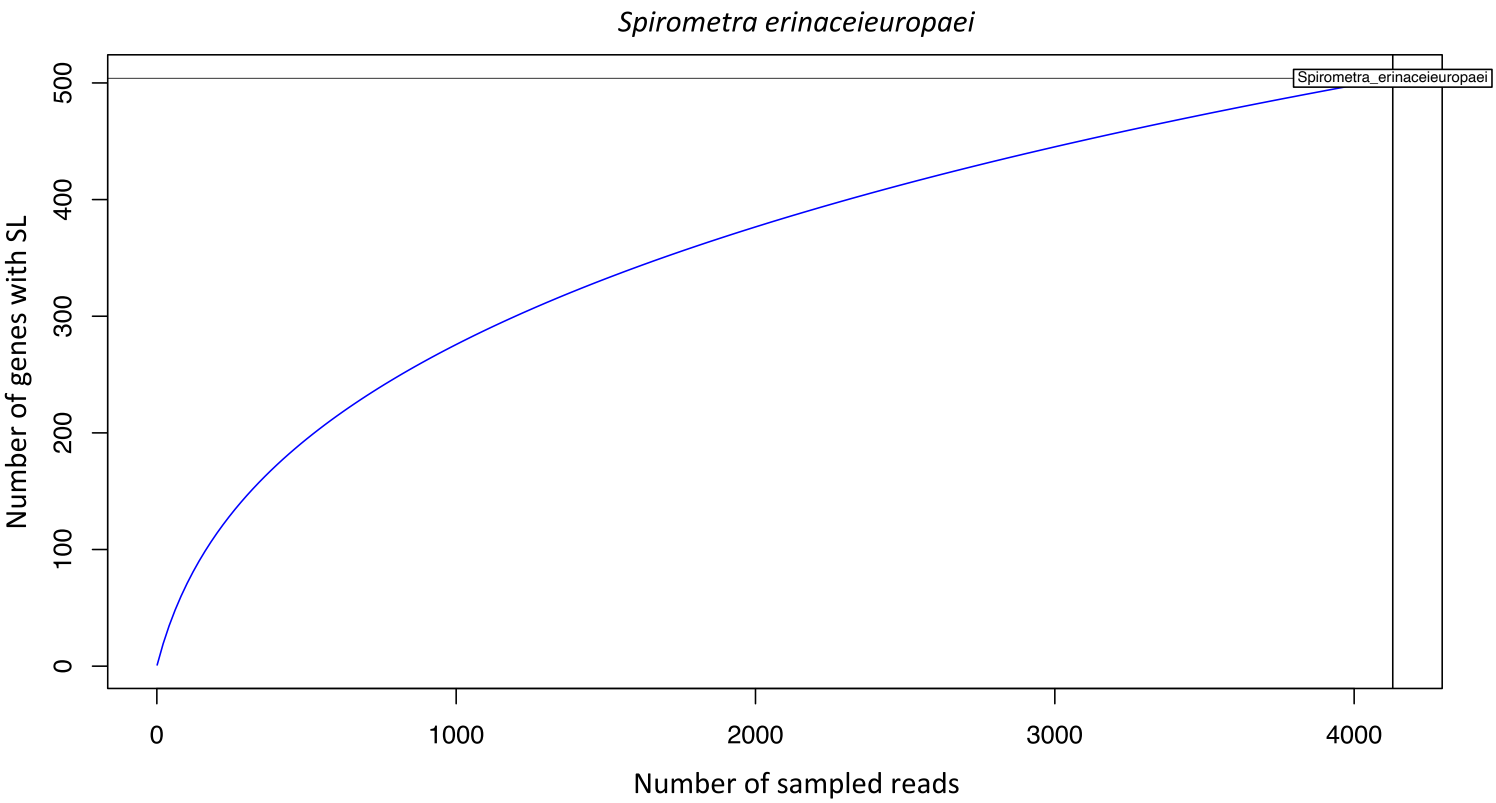
Spirometra erinaceieuropaei
Spirometra_erinaceieuropaei
Number of genes with SL
0
100
200
300
400
500
0
1000
2000
3000
4000
Number of sampled reads

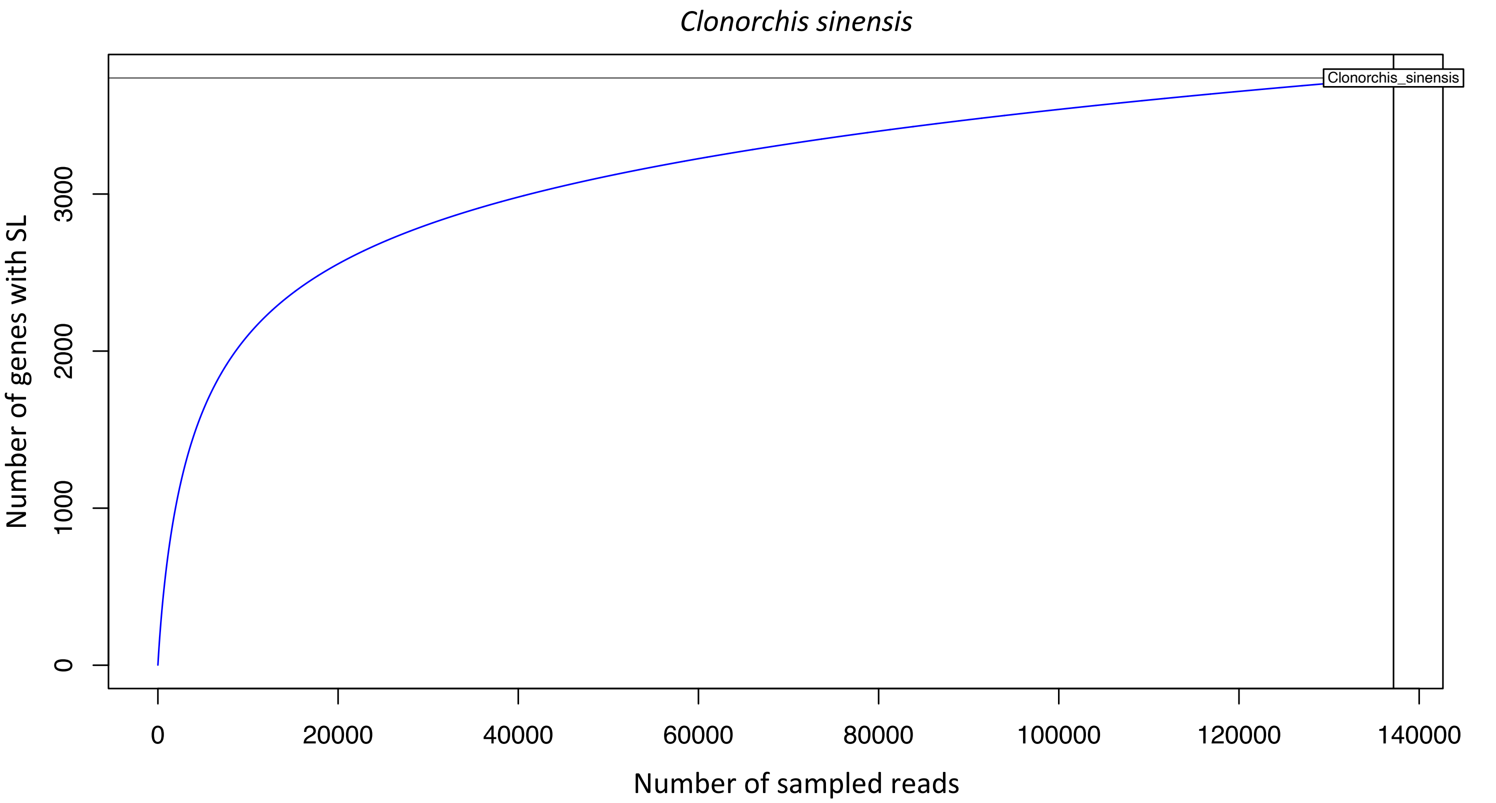
Clonorchis sinensis
Clonorchis_sinensis
Number of genes with SL
0
1000
2000
3000
0
20000
40000
60000
80000
100000
120000
140000
Number of sampled reads

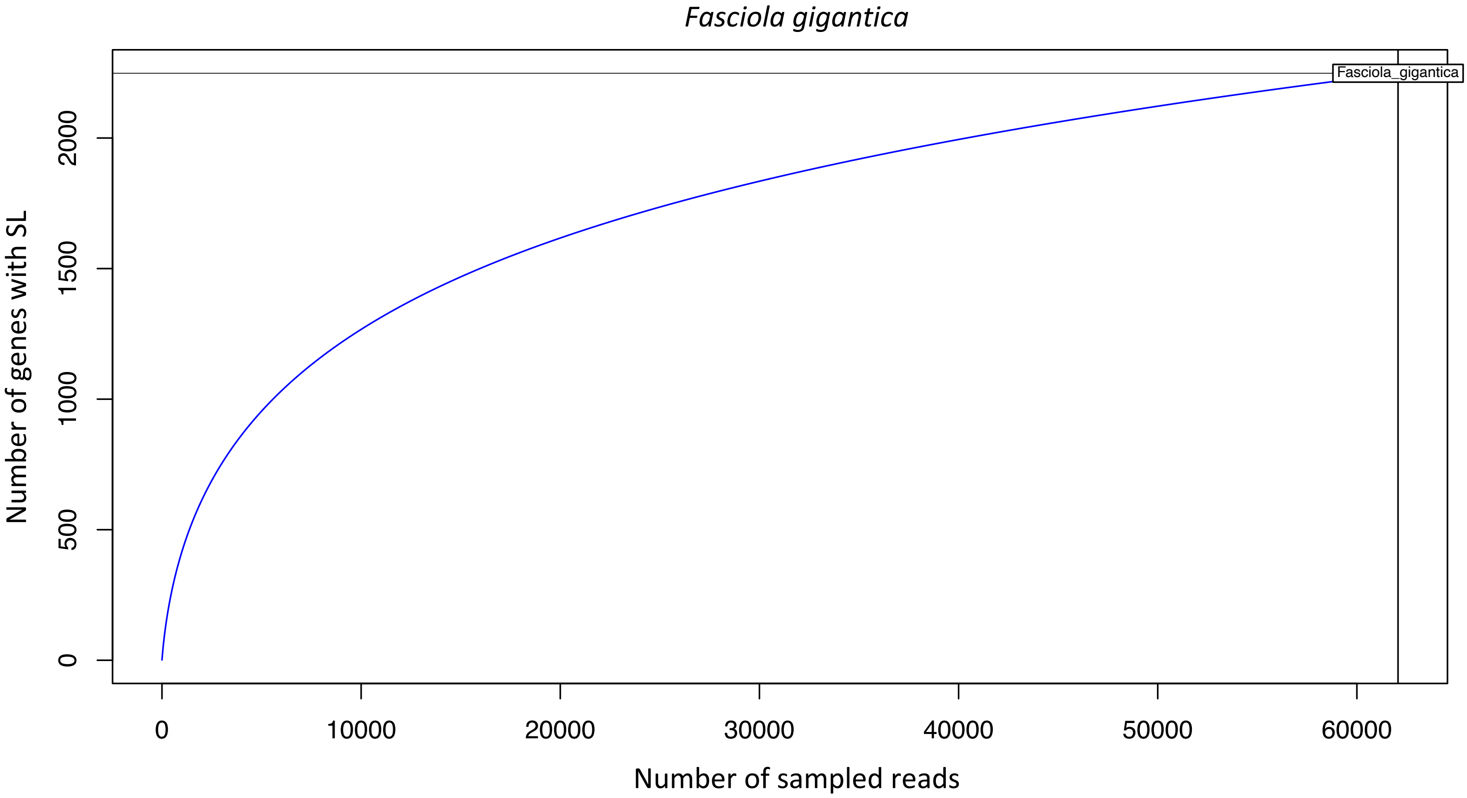
Fasciola gigantica
Fasciola_gigantica
Number of genes with SL
0
500
1000
1500
2000
0
10000
20000
30000
40000
50000
60000
Number of sampled reads

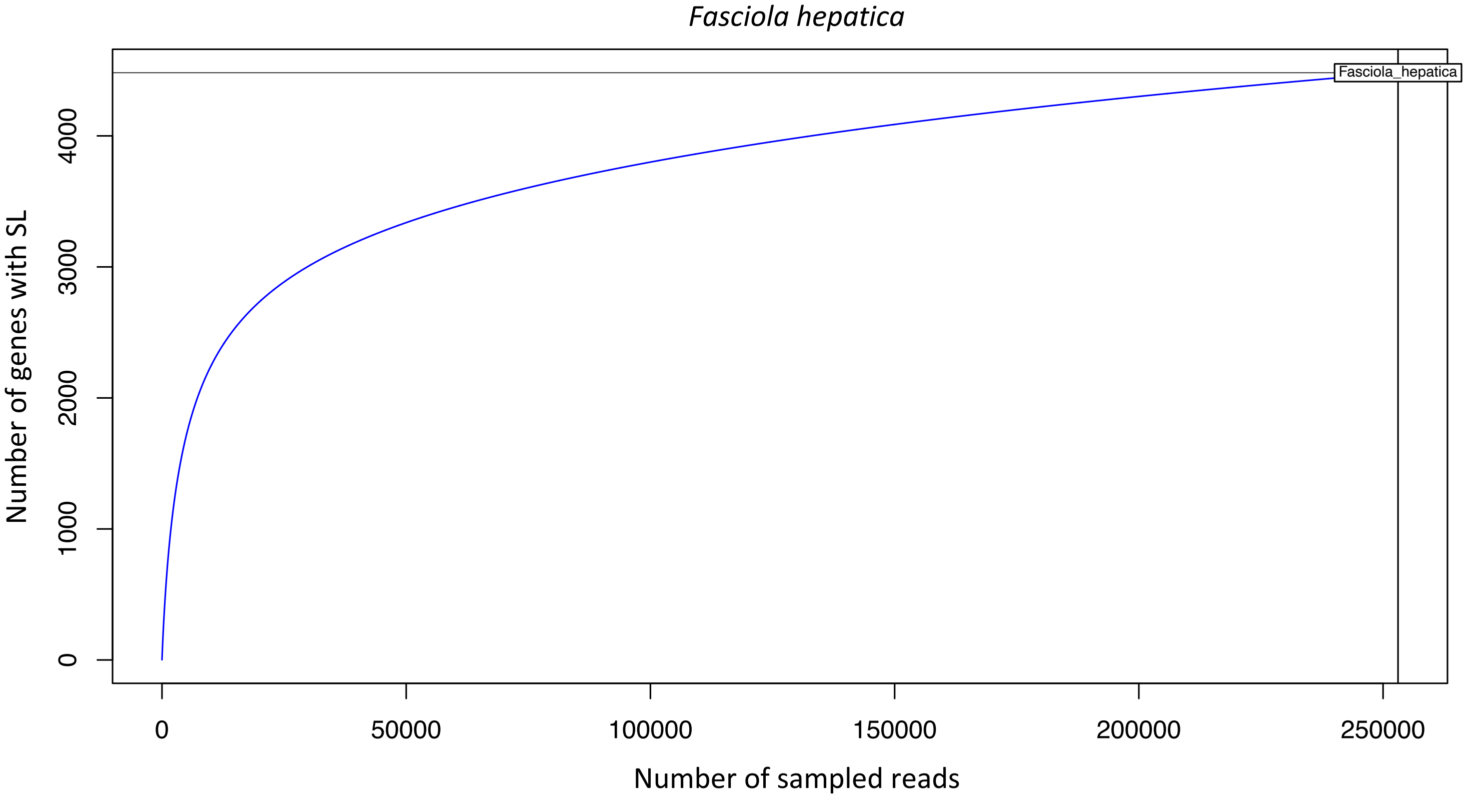
Fasciola hepatica
Fasciola_hepatica
Number of genes with SL
0
1000
2000
3000
4000
0
50000
100000
150000
200000
250000
Number of sampled reads

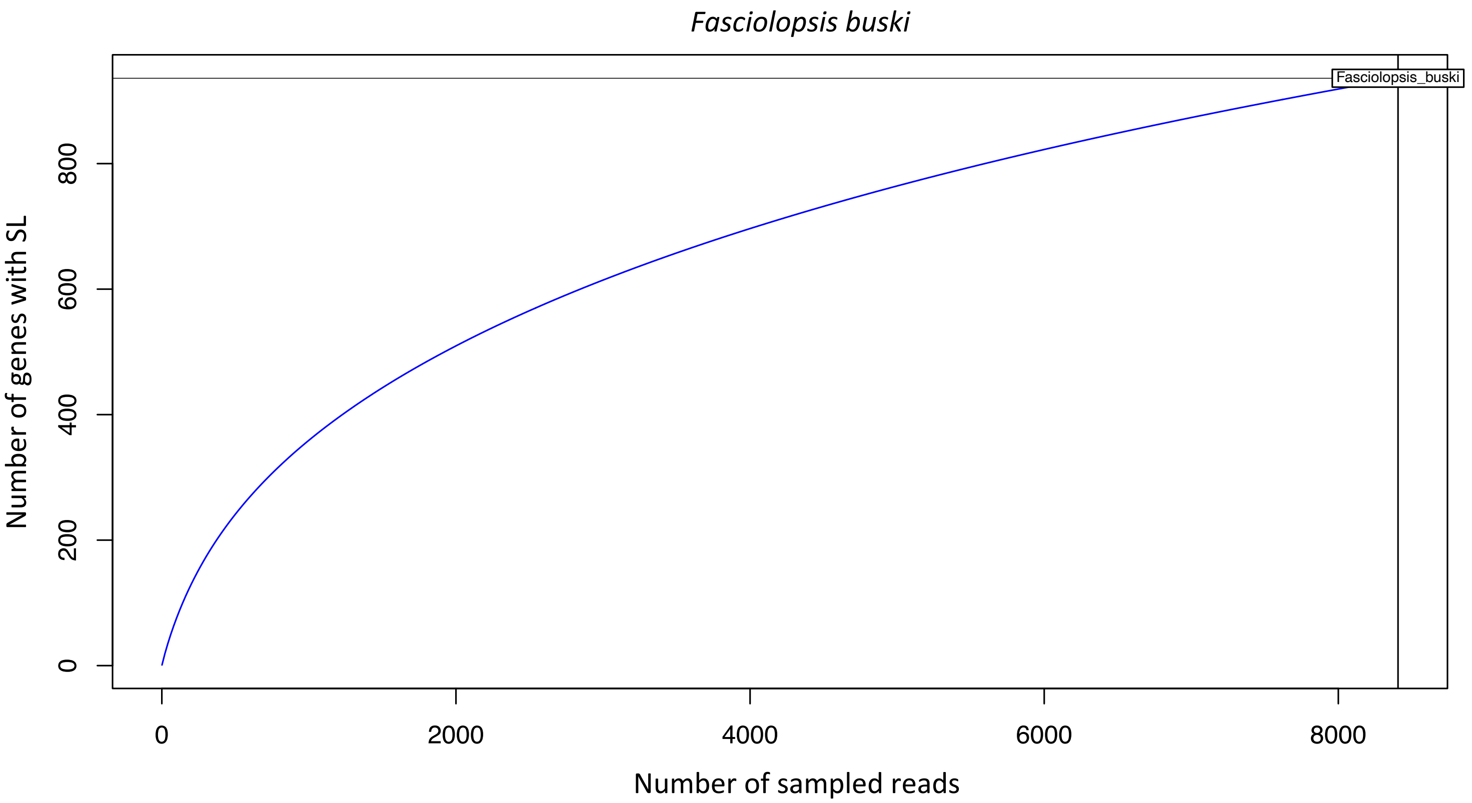
Fasciolopsis buski
Fasciolopsis_buski
Number of genes with SL
0
200
400
600
800
0
2000
4000
6000
8000
Number of sampled reads

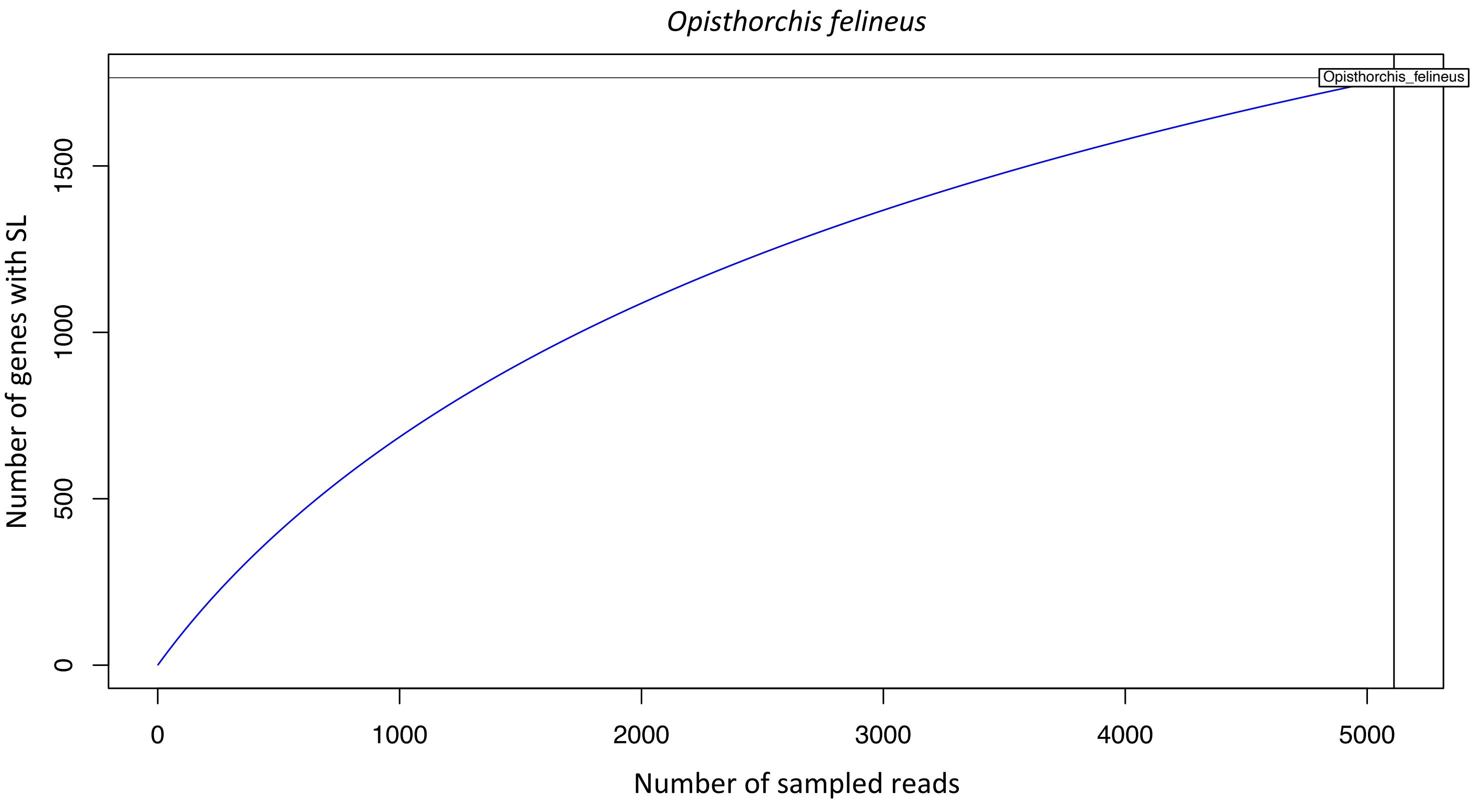
Opisthorchis felineus
Opisthorchis_felineus
Number of genes with SL
0
500
1000
1500
0
1000
2000
3000
4000
5000
Number of sampled reads

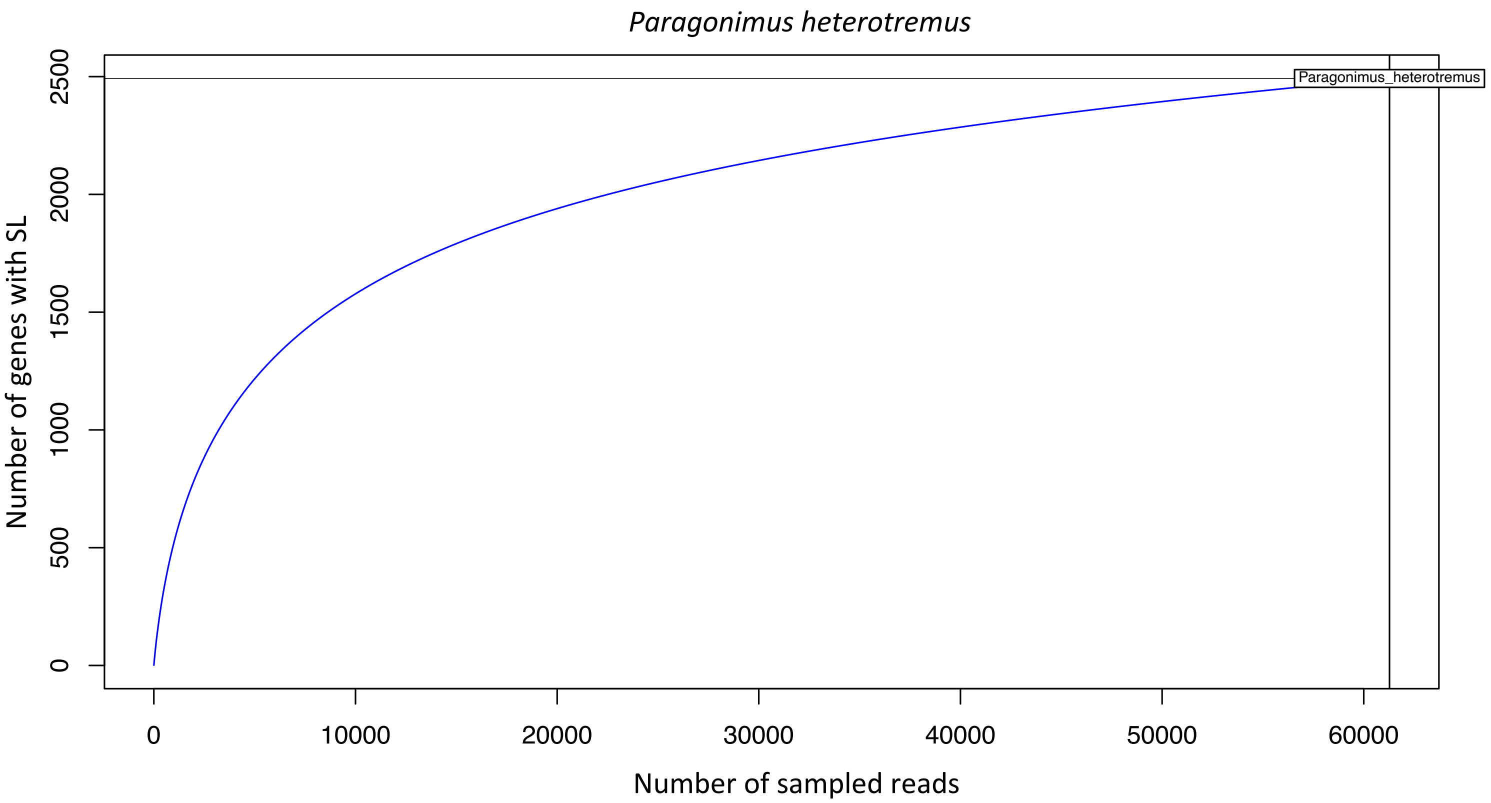
Paragonimus heterotremus
Paragonimus_heterotremus
Number of genes with SL
Number of sampled reads
0
500
1000
1500
2000
2500
0
10000
20000
30000
40000
50000
60000

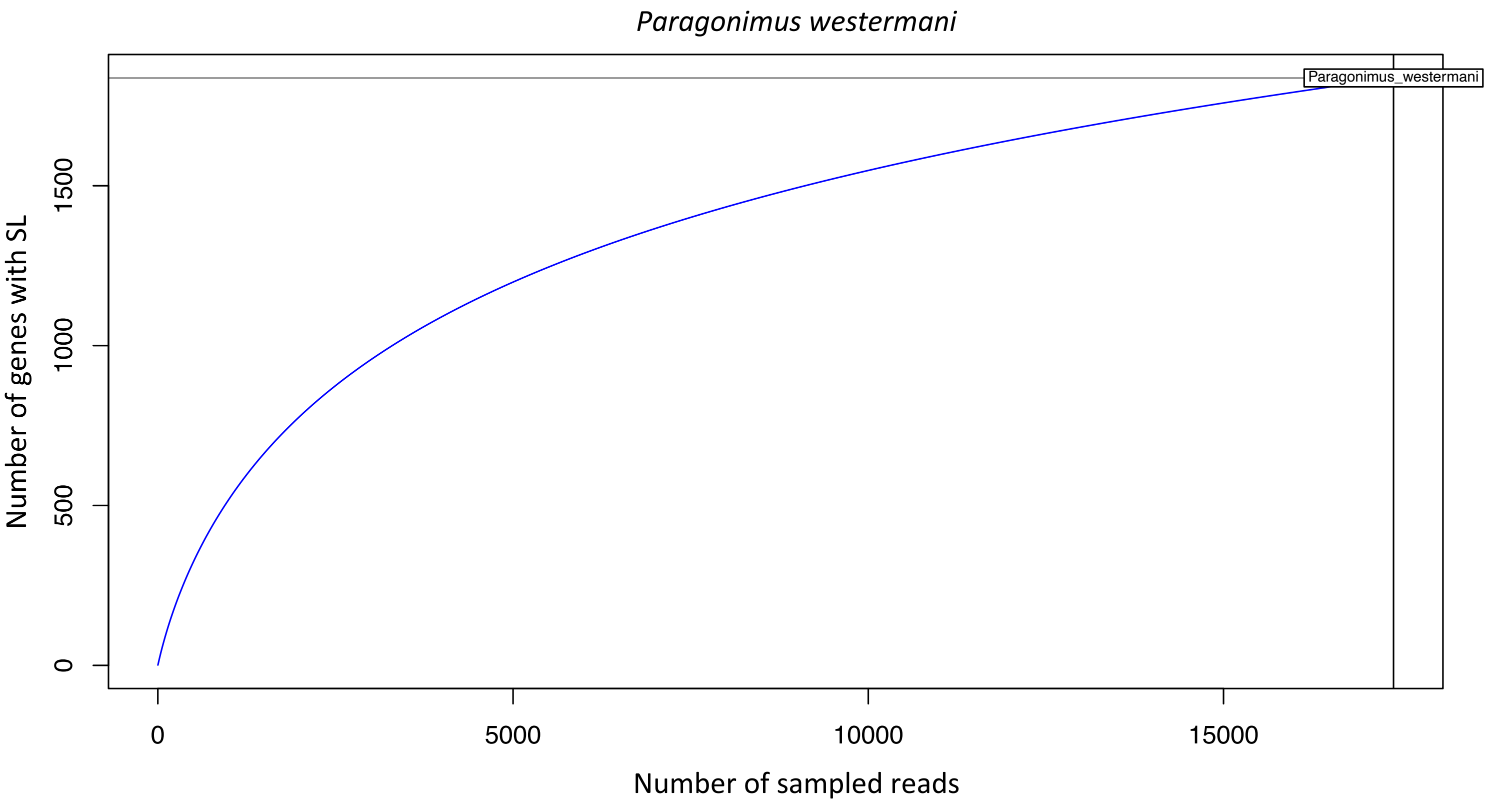
Paragonimus westermani
Paragonimus_westermani
Number of genes with SL
Number of sampled reads
0
500
1000
1500
0
5000
10000
15000

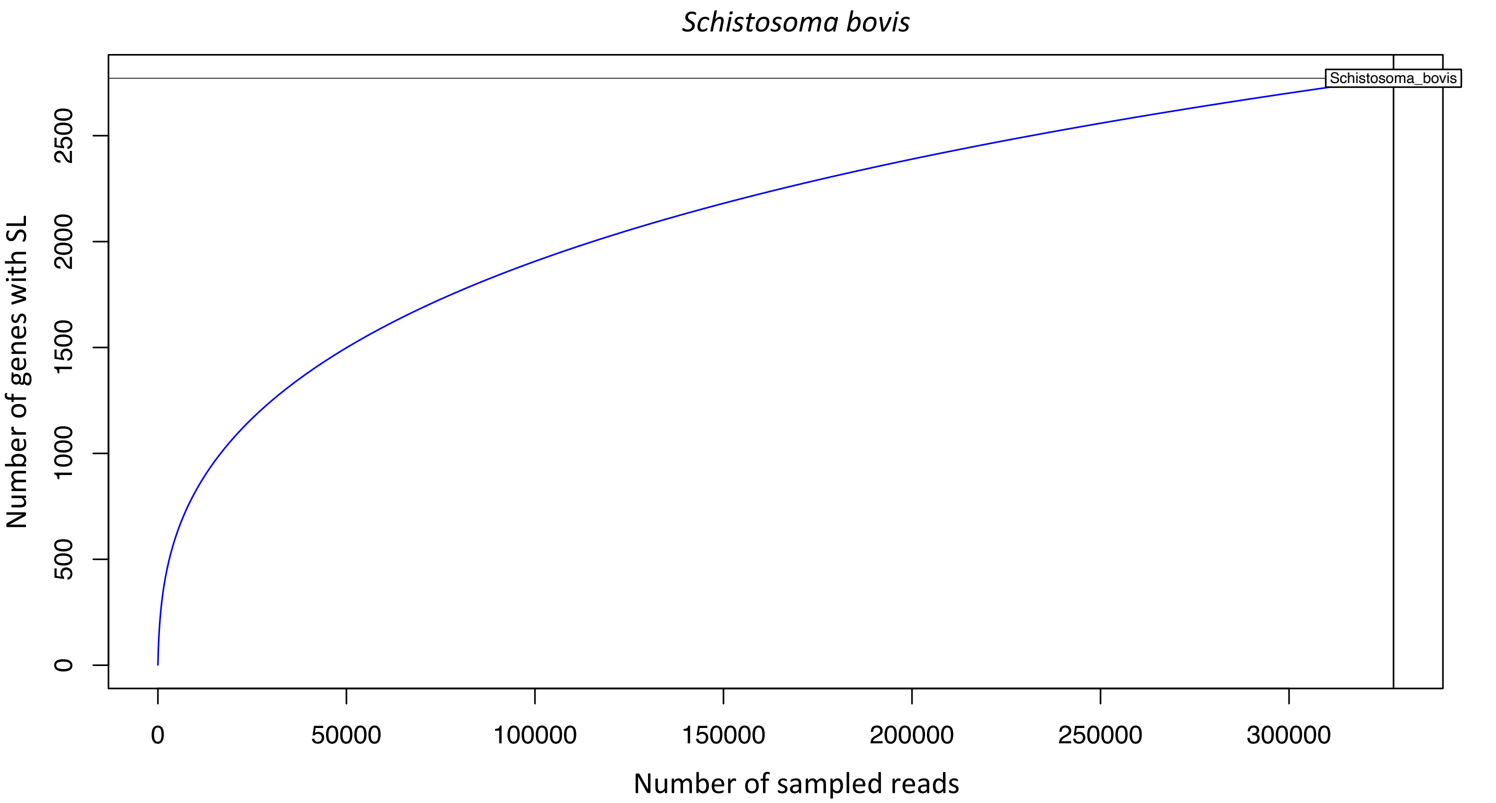
Schistosoma bovis
Number of genes with SL
0
500
1000
1500
2000
2500
0
50000
100000
150000
200000
250000
300000
Number of sampled reads
Schistosoma_bovis

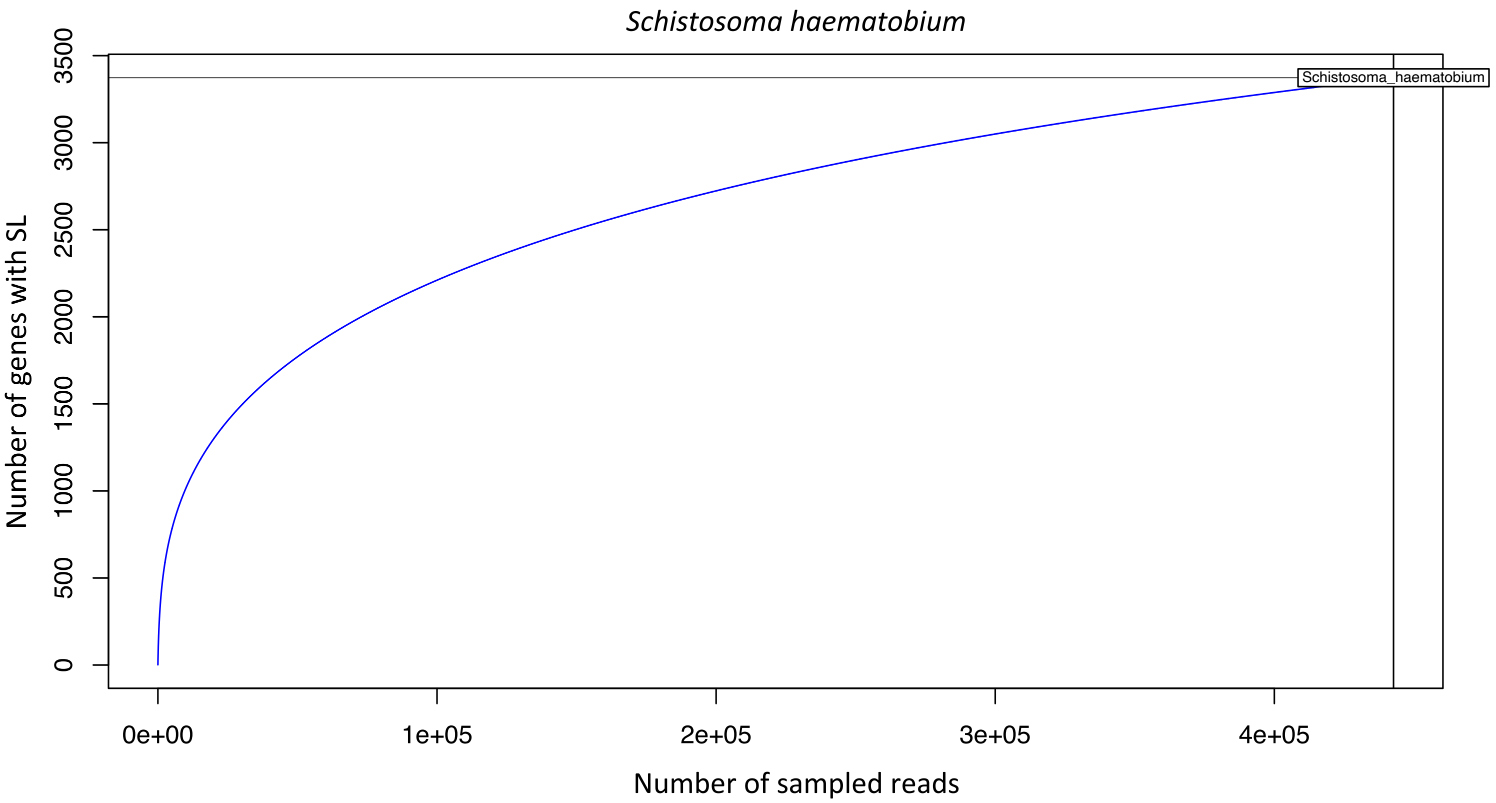
Schistosoma haematobium
Number of genes with SL
0
500
1000
1500
2000
2500
3000
3500
0e+00
1e+05
2e+05
3e+05
4e+05
Number of sampled reads
Schistosoma_haematobium

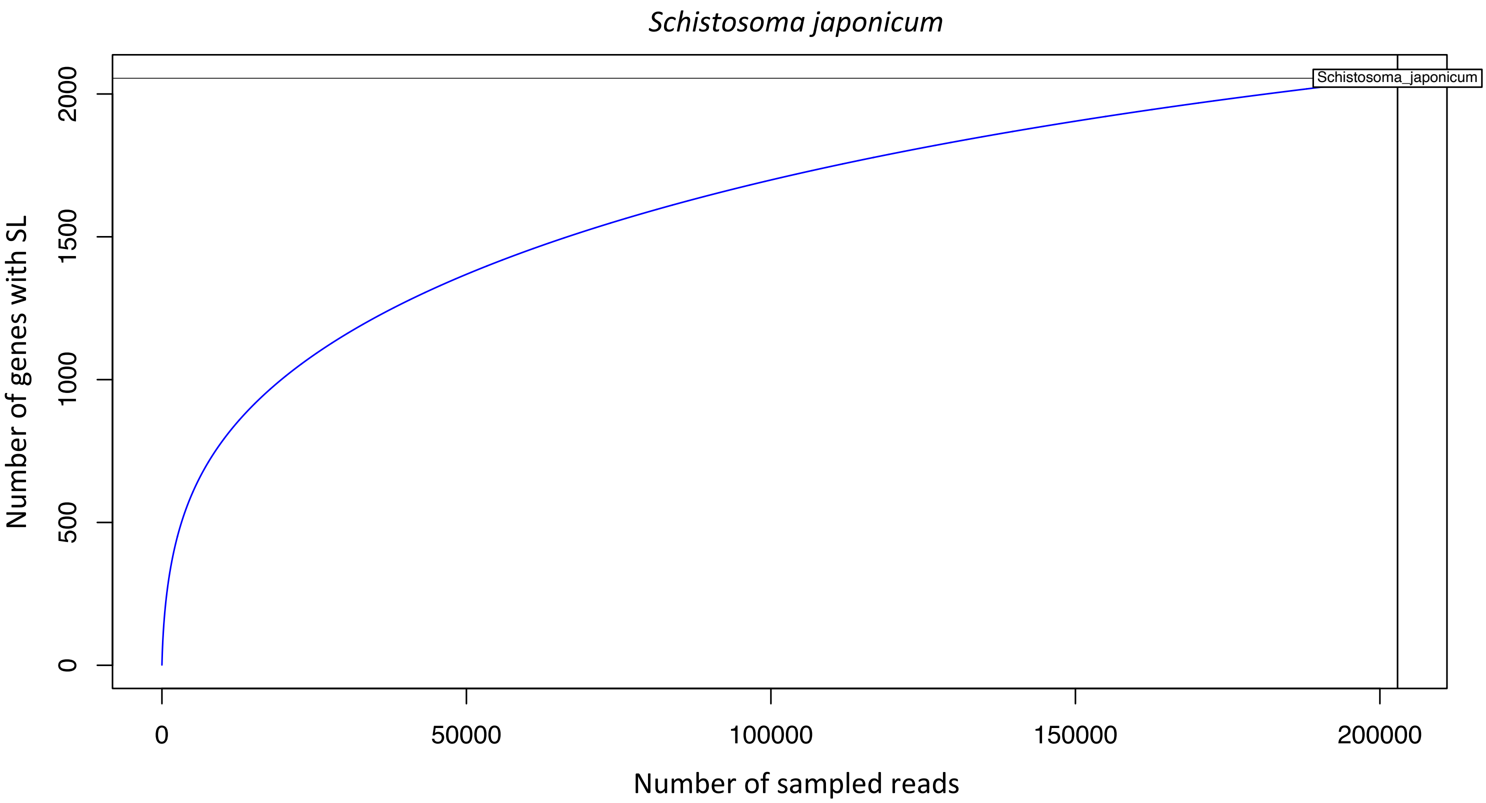
Schistosoma japonicum
Number of genes with SL
0
500
1000
1500
2000
Number of sampled reads
0
50000
100000
150000
200000
Schistosoma_japonicum

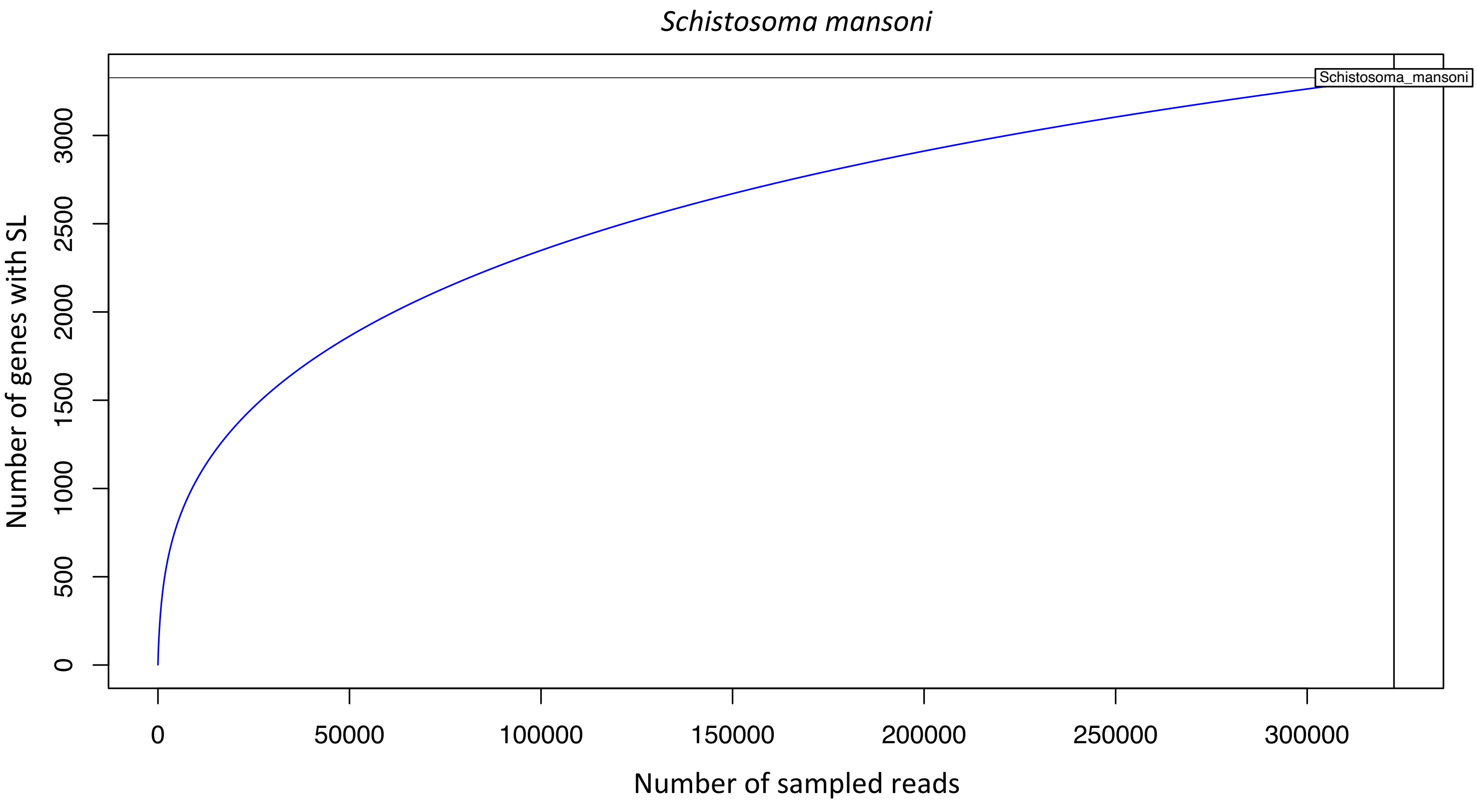
Schistosoma mansoni
Number of genes with SL
0
500
1000
1500
2000
2500
3000
Number of sampled reads
0
50000
100000
150000
200000
250000
300000
Schistosoma_mansoni

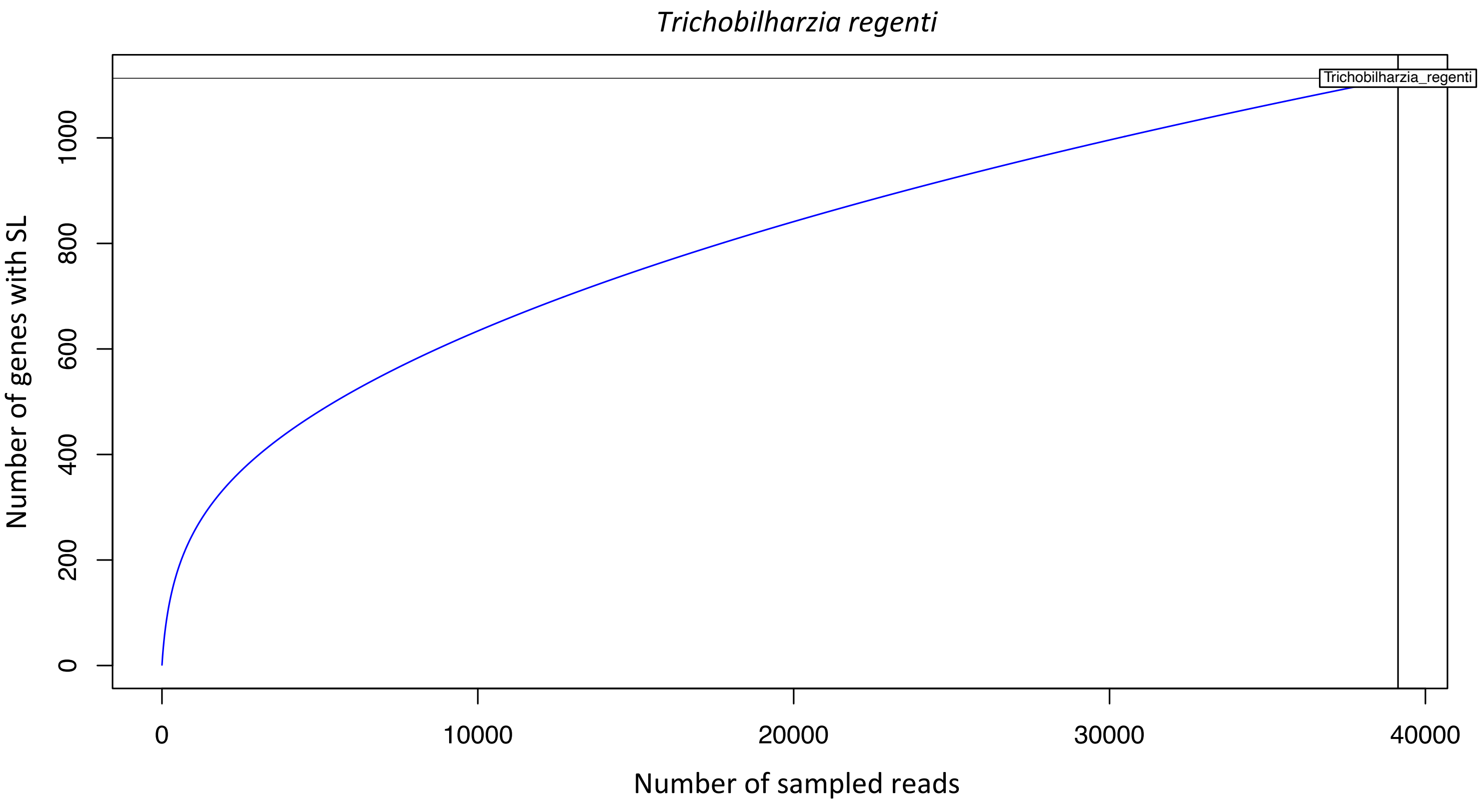

Trichobilharzia regenti
Trichobilharzia_regenti
Number of genes with SL
0
200
400
600
800
1000
0
10000
20000
30000
40000
Number of sampled reads
