# Supplementary material for: Evolution of SL-RNA Genes and Their Splicing Targets in Parasitic Flatworms
Source: Mol Biol Evol. 2025 Sep 23;42(11):msaf228. doi: 10.1093/molbev/msaf228 (PMC12582326; doi:10.1093/molbev/msaf228)
Supplement: msaf228_Supplementary_Data [file msaf228_supplementary_data.zip › Supplementary File 7 - 14082025.pdf]

Supplementary File 7. Histograms of the intergenic distances observed between gene models in the analyzed species. In all cases, each bin represents 50 bases, and the red line marks 300 bases, the distance used for operon candidate definitions. Only contiguous pairs of non-overlapping gene models encoded on the same strand and separated by less than 20 kb were included. Chimeric gene models were processed as reported in the original annotation.

Index:

Pag. 2: *Clonorchis sinensis*

Pag. 3: *Echinococcus granulosus*

Pag. 4: *Echinococcus multilocularis*

Pag. 5: *Fasciola gigantica*

Pag. 6: *Fasciola hepatica*

Pag. 7: *Fasciolopsis buski*

Pag. 8: *Hymenolepis diminuta*

Pag. 9: *Hymenolepis microstoma*

Pag. 10: *Mesocestoides corti*

Pag. 11: *Opisthorchis felineus*

Pag. 12: *Paragonimus heterotremus*

Pag. 13: *Paragonimus westermani*

Pag. 14: *Schistocephalus solidus*

Pag. 15: *Schistosoma bovis*

Pag. 16: *Schistosoma haematobium*

Pag. 17: *Schistosoma japonicum*

Pag. 18: *Schistosoma mansoni*

Pag. 19: *Sparganum proliferum*

Pag. 20: *Spirometra erinaceieuropaei*

Pag. 21: *Taenia asiatica*

Pag. 22: *Taenia multiceps*

Pag. 23: *Taenia saginata*

Pag. 24: Taenia solium

Pag. 25: Trichobilharzia regenti

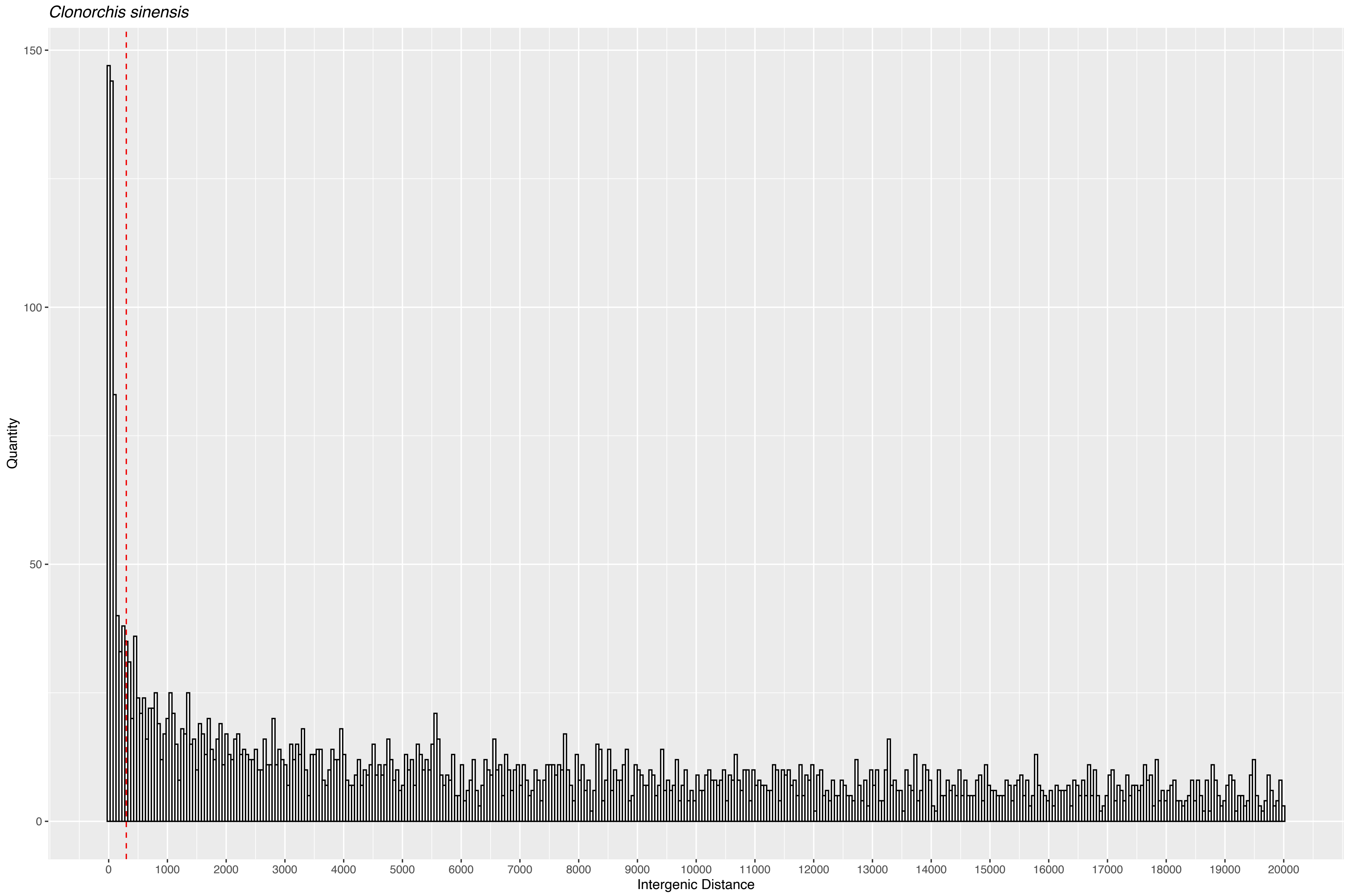

Clonorchis sinensis
Quantity
150
100
50
0
0
1000
2000
3000
4000
5000
6000
7000
8000
9000
10000
11000
12000
13000
14000
15000
16000
17000
18000
19000
20000
Intergenic Distance

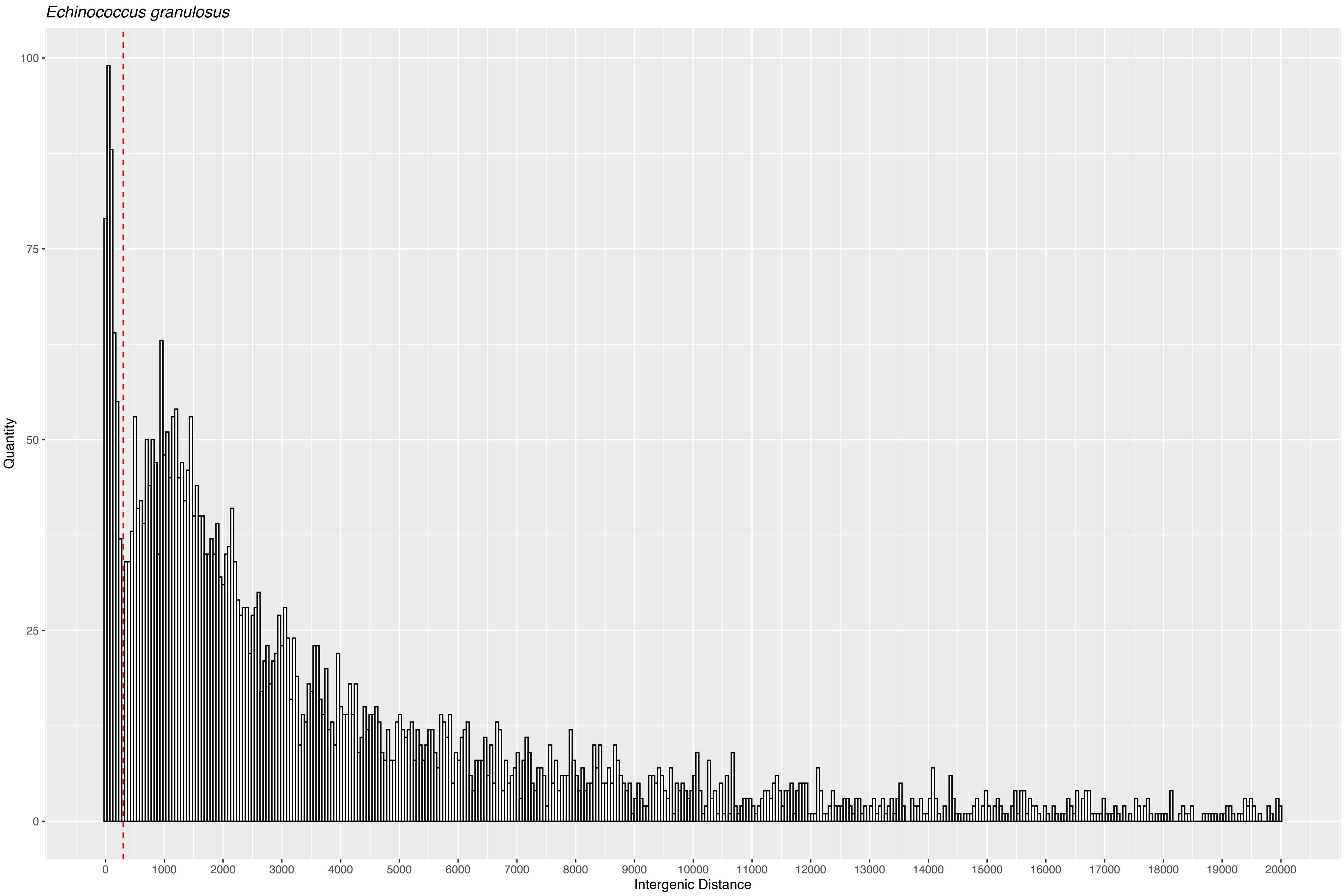

Echinococcus granulosus
Quantity
100
75
50
25
0
0
1000
2000
3000
4000
5000
6000
7000
8000
9000
10000
11000
12000
13000
14000
15000
16000
17000
18000
19000
20000
Intergenic Distance

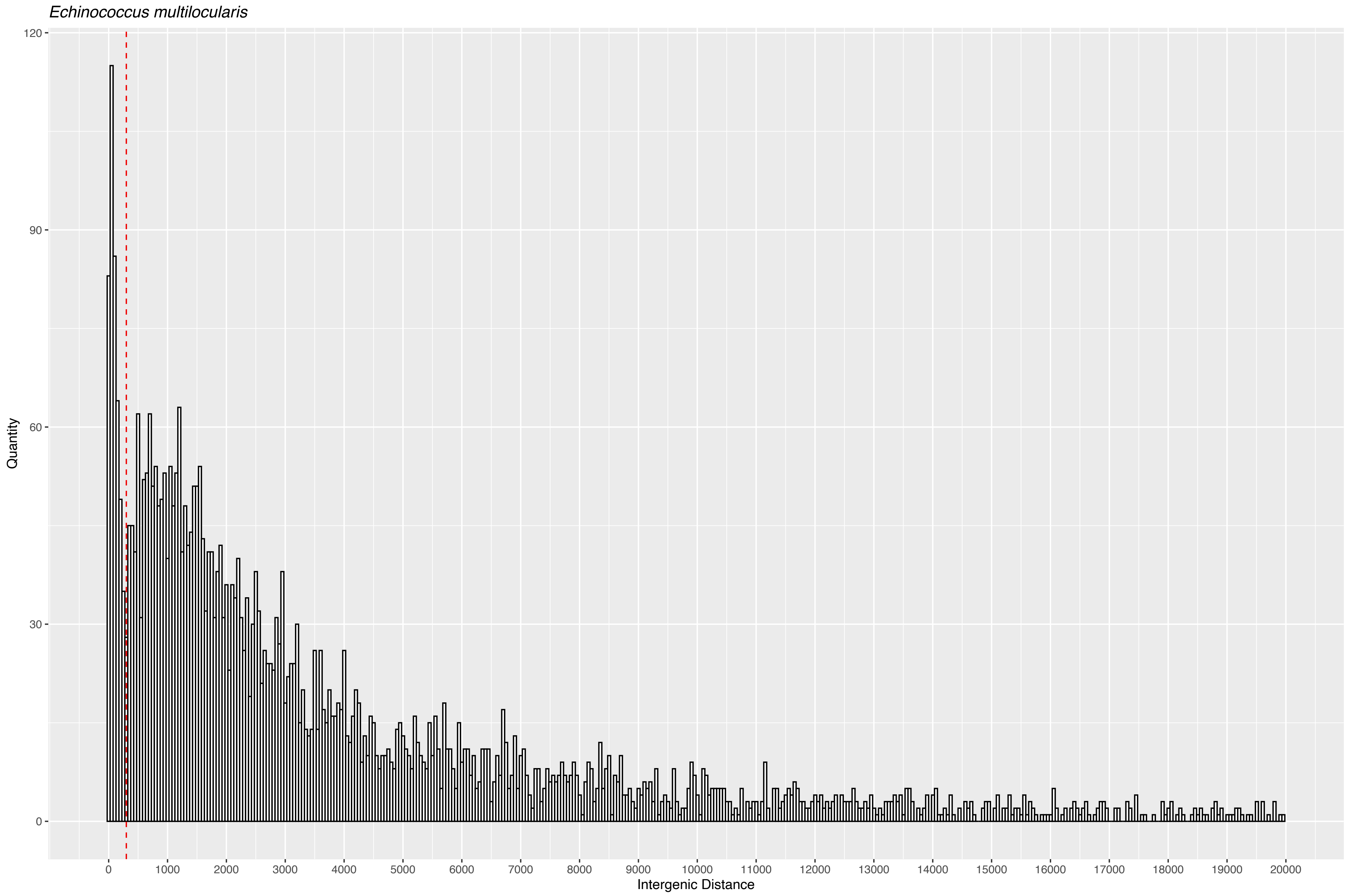

Echinococcus multilocularis
Quantity
0
30
60
90
120
0
1000
2000
3000
4000
5000
6000
7000
8000
9000
10000
11000
12000
13000
14000
15000
16000
17000
18000
19000
20000
Intergenic Distance

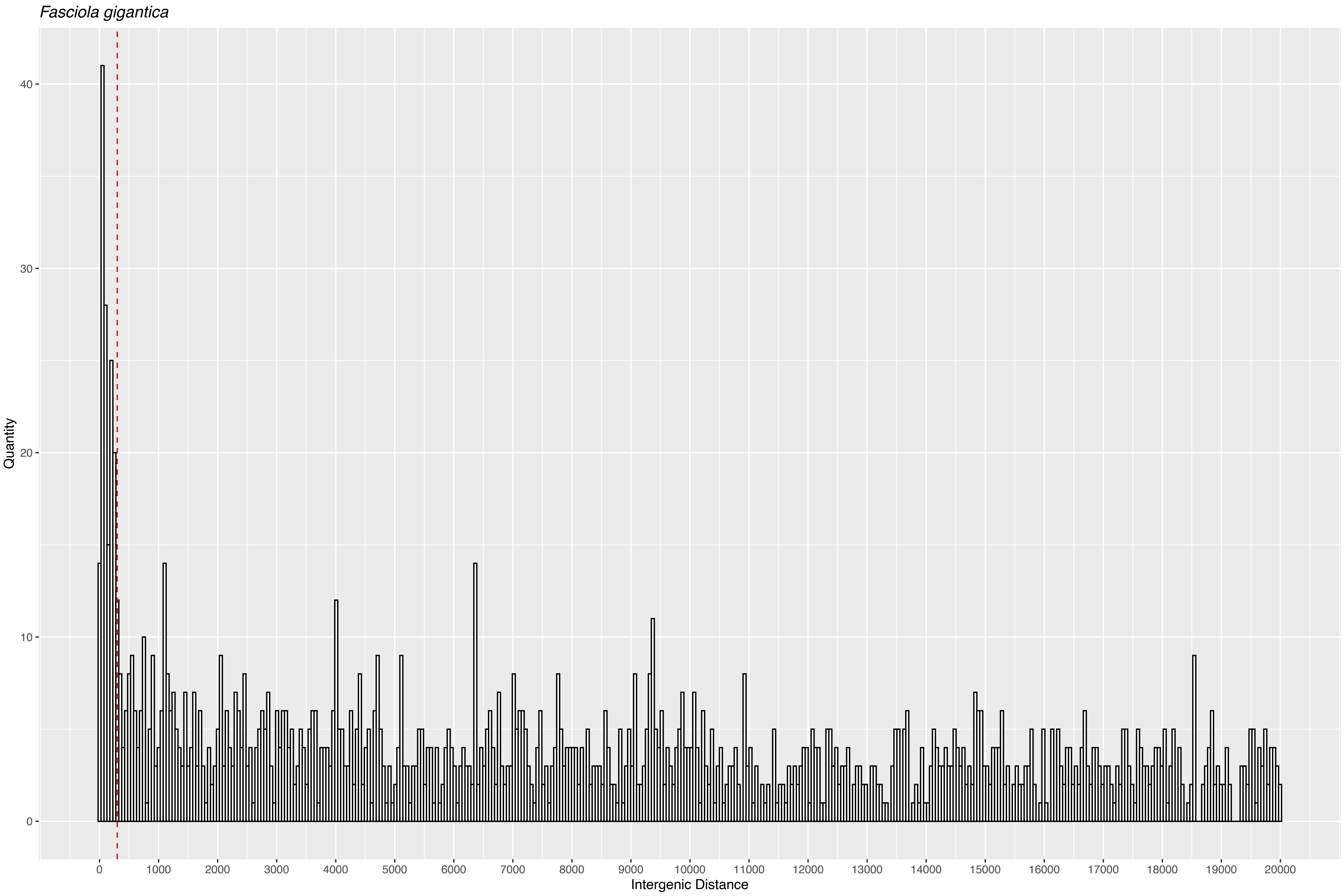

Fasciola gigantica
Quantity
Intergenic Distance
0
10
20
30
40
0
1000
2000
3000
4000
5000
6000
7000
8000
9000
10000
11000
12000
13000
14000
15000
16000
17000
18000
19000
20000

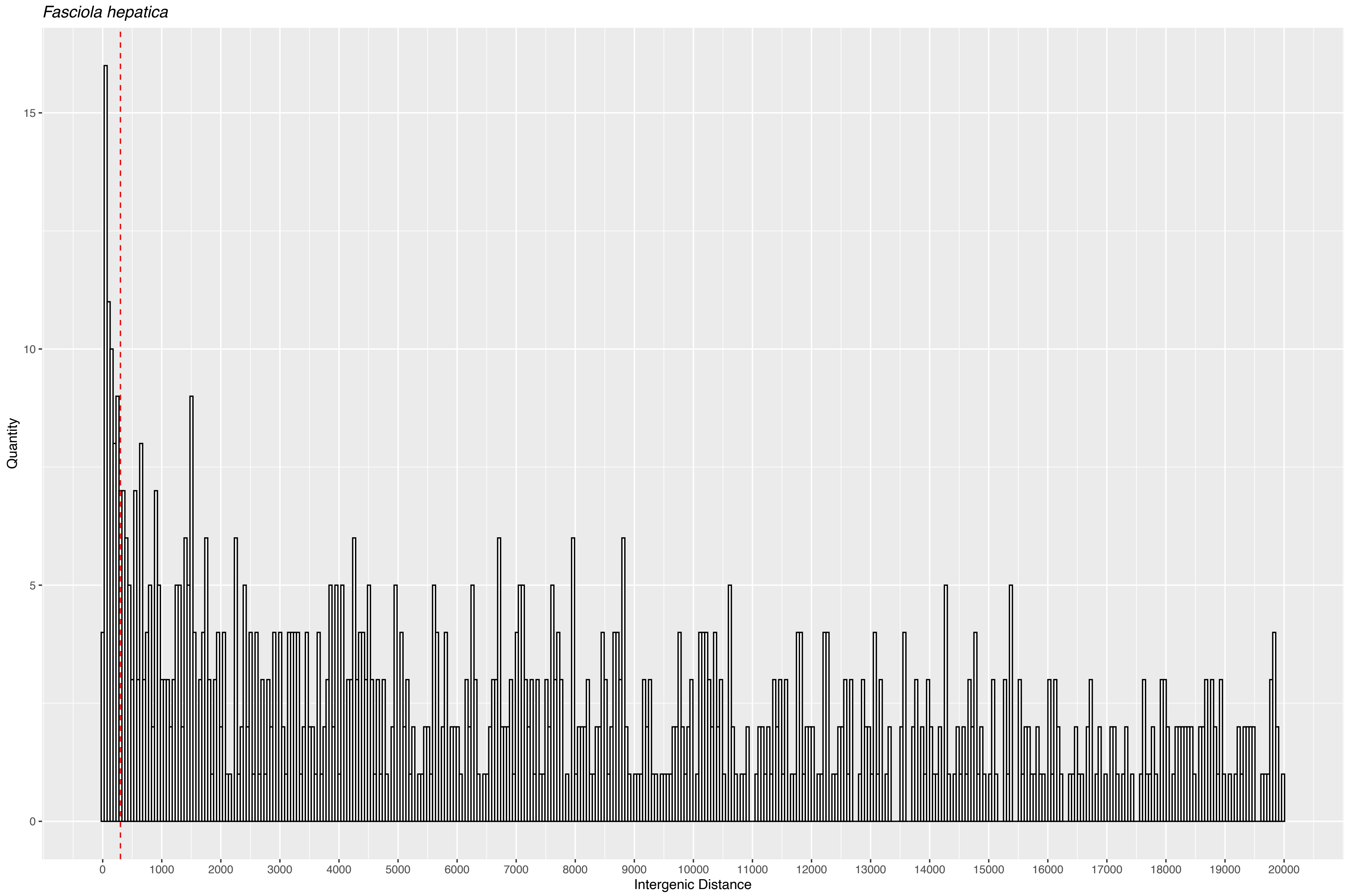

Fasciola hepatica
Quantity
0
5
10
15
0
1000
2000
3000
4000
5000
6000
7000
8000
9000
10000
11000
12000
13000
14000
15000
16000
17000
18000
19000
20000
Intergenic Distance

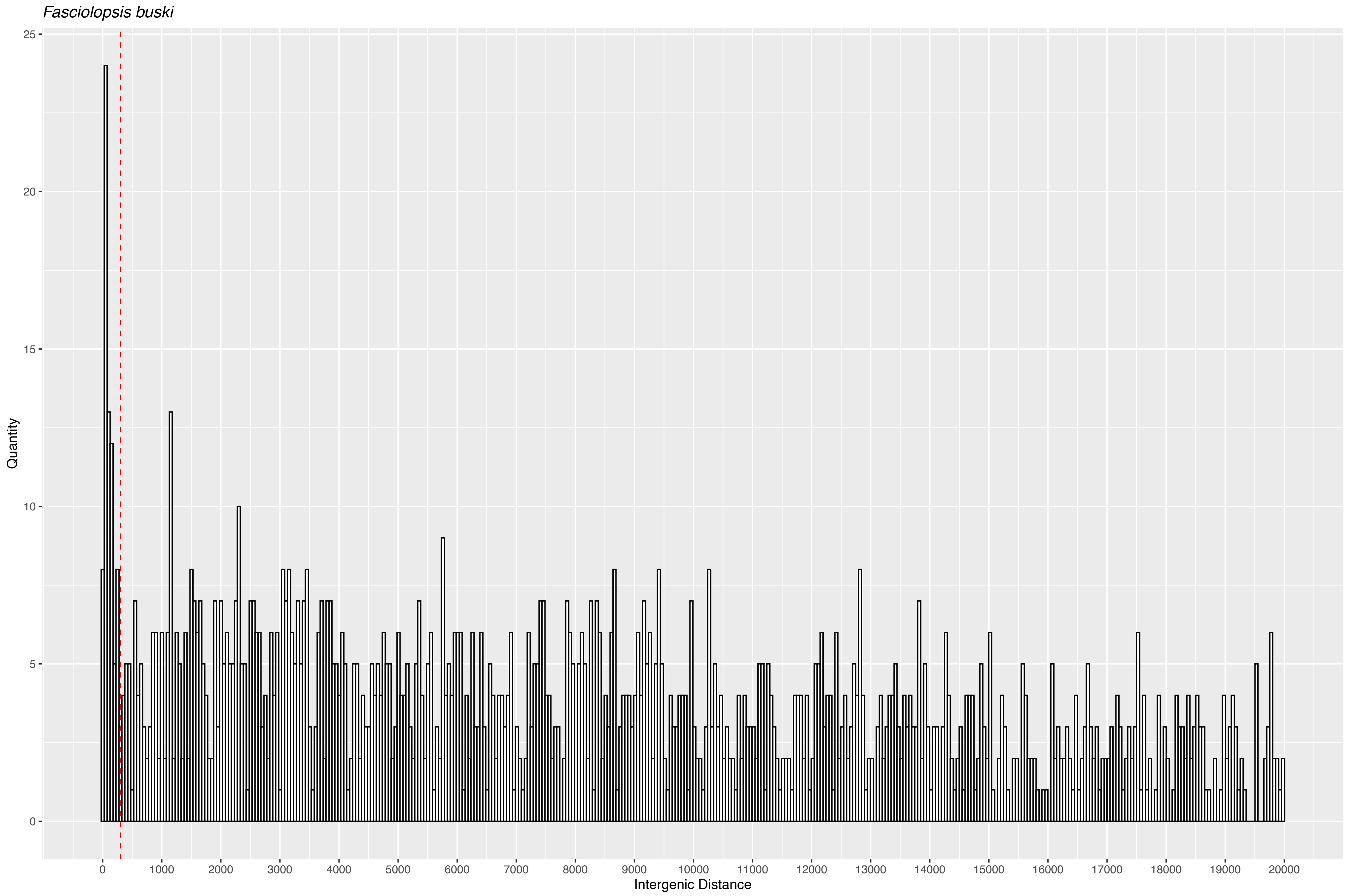

Fasciolopsis buski
Quantity
0
5
10
15
20
25
0
1000
2000
3000
4000
5000
6000
7000
8000
9000
10000
11000
12000
13000
14000
15000
16000
17000
18000
19000
20000
Intergenic Distance

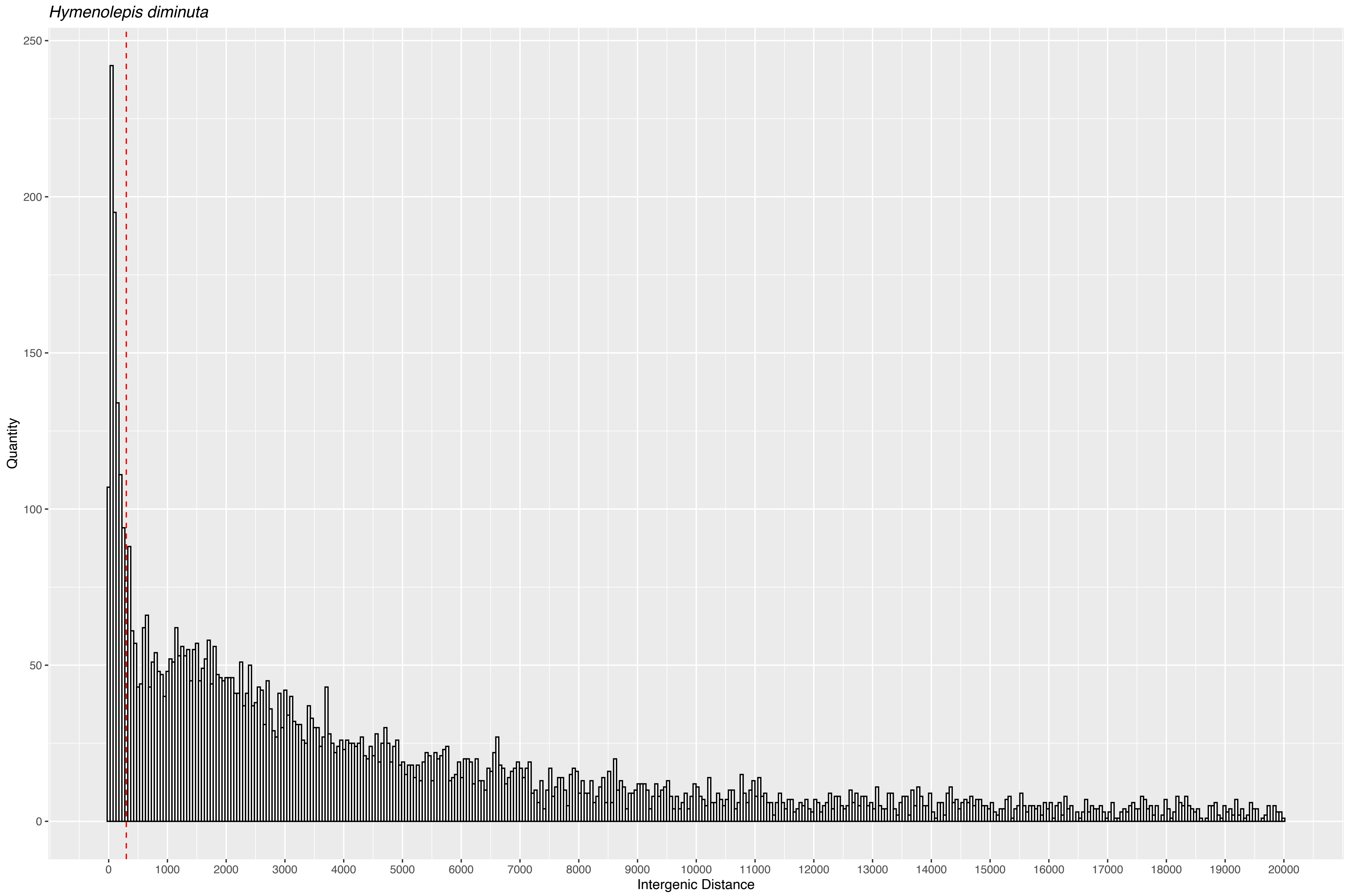
Hymenolepis diminuta
Quantity
0
50
100
150
200
250
0 1000 2000 3000 4000 5000 6000 7000 8000 9000 10000 11000 12000 13000 14000 15000 16000 17000 18000 19000 20000
Intergenic Distance

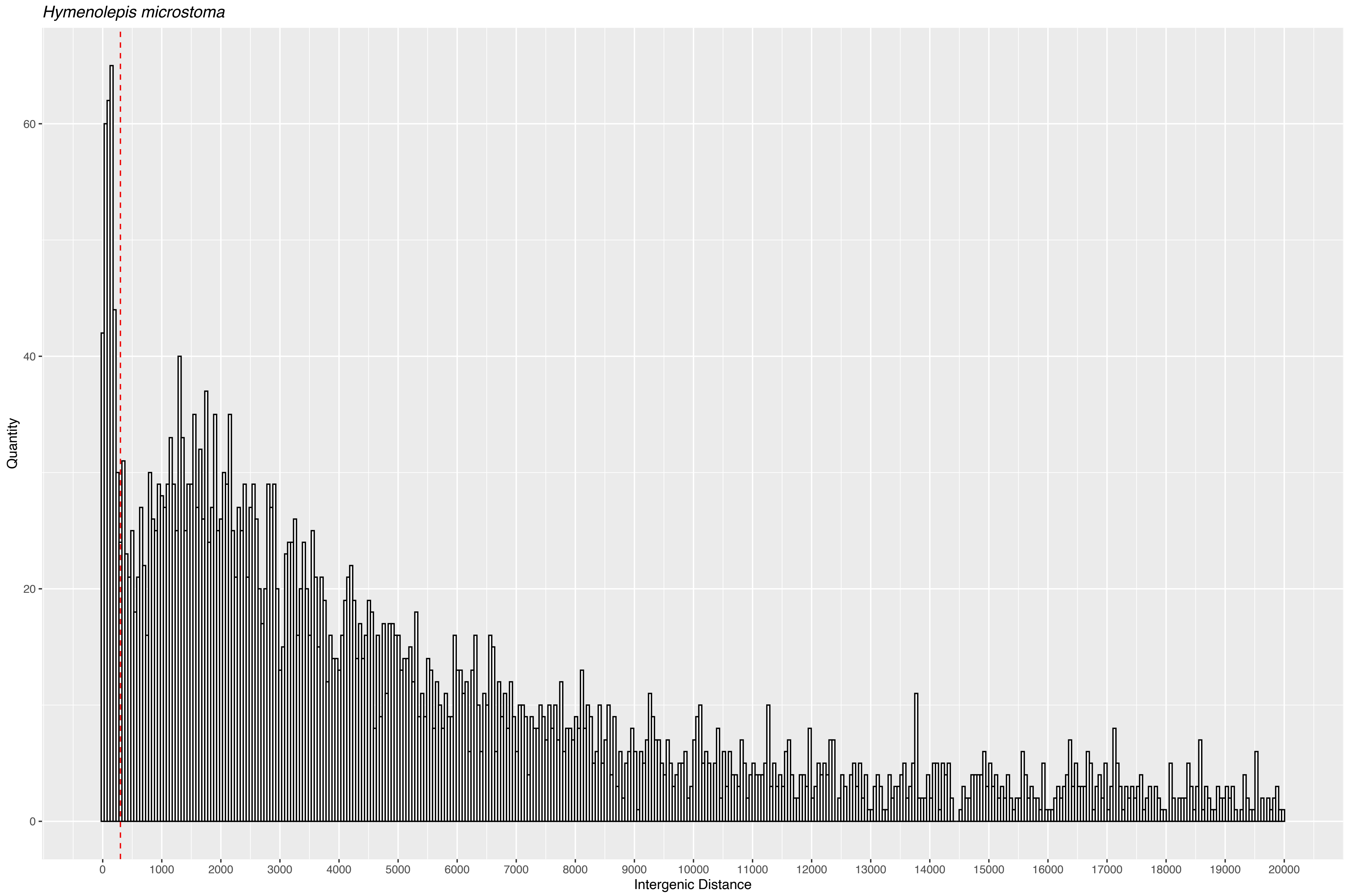
Hymenolepis microstoma
Quantity
0
20
40
60
Intergenic Distance
0
1000
2000
3000
4000
5000
6000
7000
8000
9000
10000
11000
12000
13000
14000
15000
16000
17000
18000
19000
20000

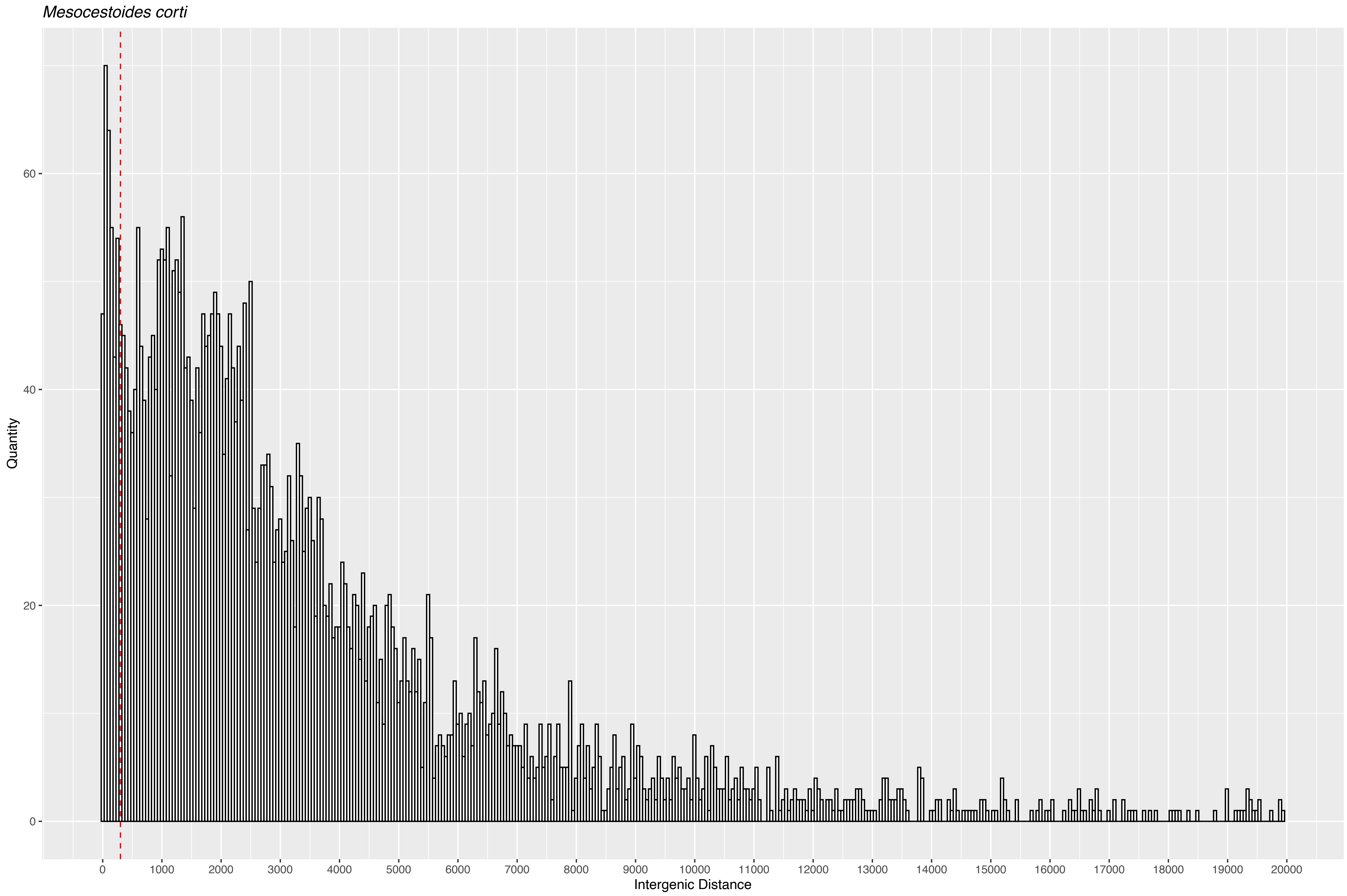

Mesocestoides corti
Quantity
Intergenic Distance
0
20
40
60
0
1000
2000
3000
4000
5000
6000
7000
8000
9000
10000
11000
12000
13000
14000
15000
16000
17000
18000
19000
20000

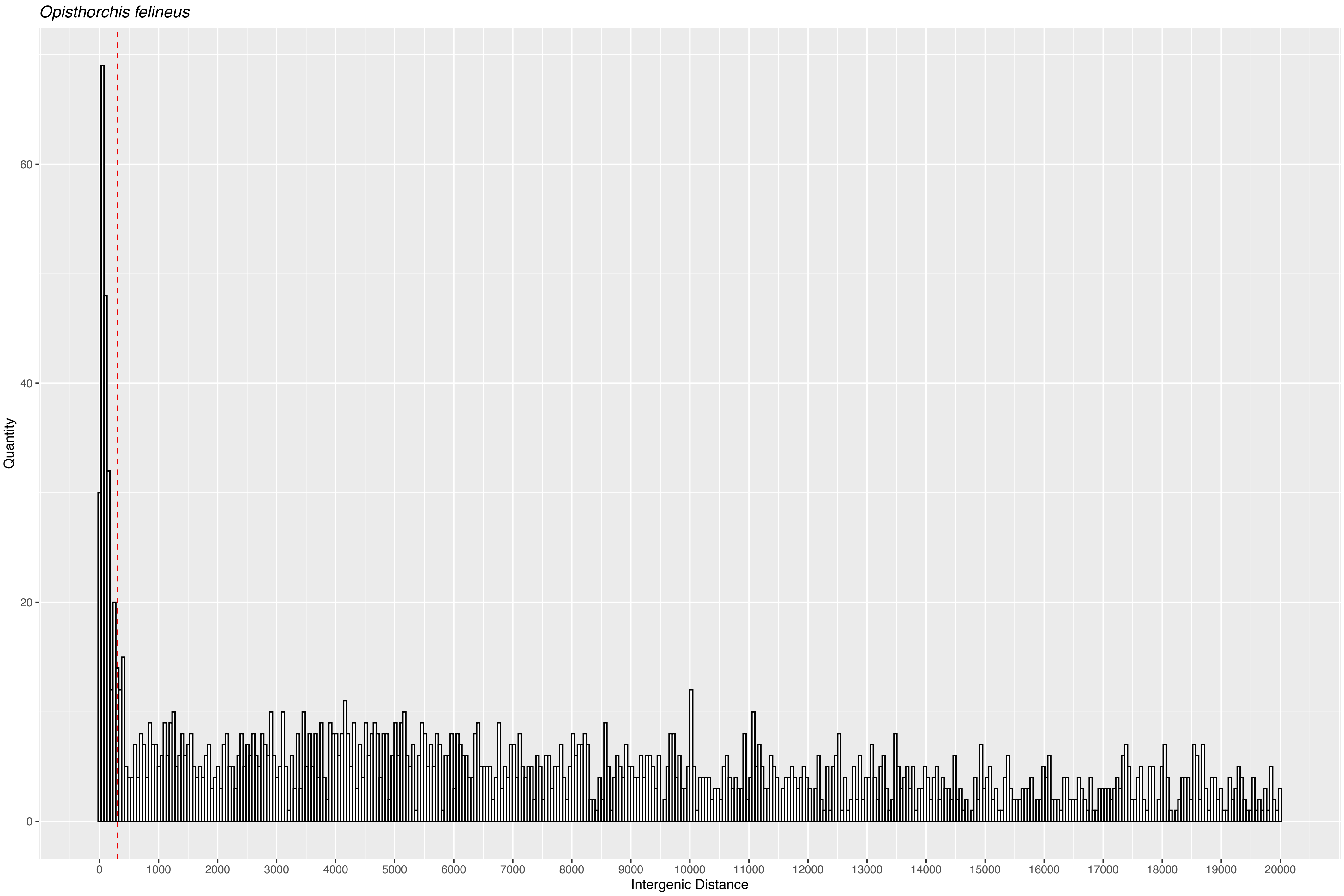

Opisthorchis felineus
Quantity
Intergenic Distance
0
20
40
60
0
1000
2000
3000
4000
5000
6000
7000
8000
9000
10000
11000
12000
13000
14000
15000
16000
17000
18000
19000
20000

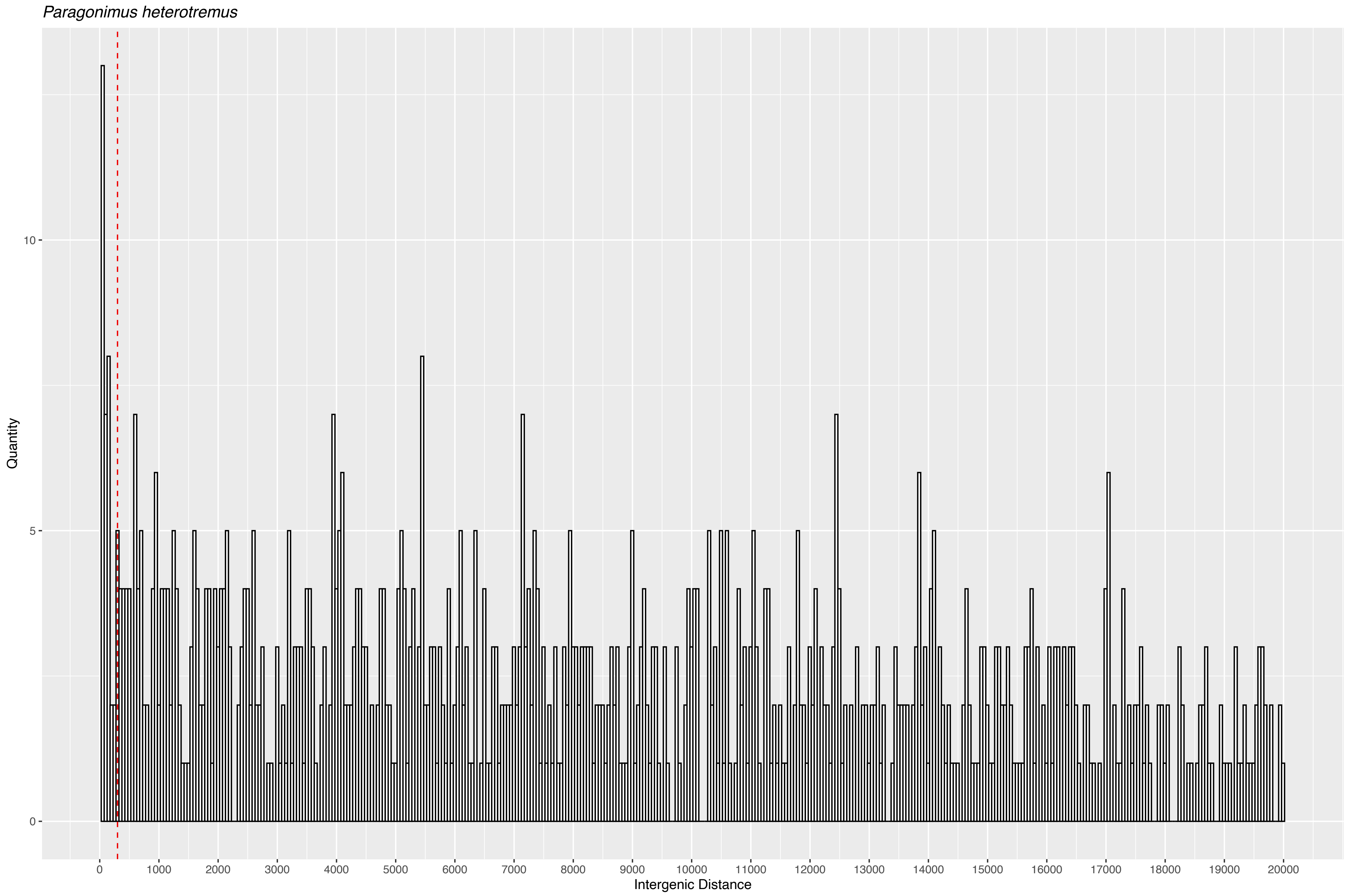

Paragonimus heterotremus
Quantity
0
5
10
Intergenic Distance
0
1000
2000
3000
4000
5000
6000
7000
8000
9000
10000
11000
12000
13000
14000
15000
16000
17000
18000
19000
20000

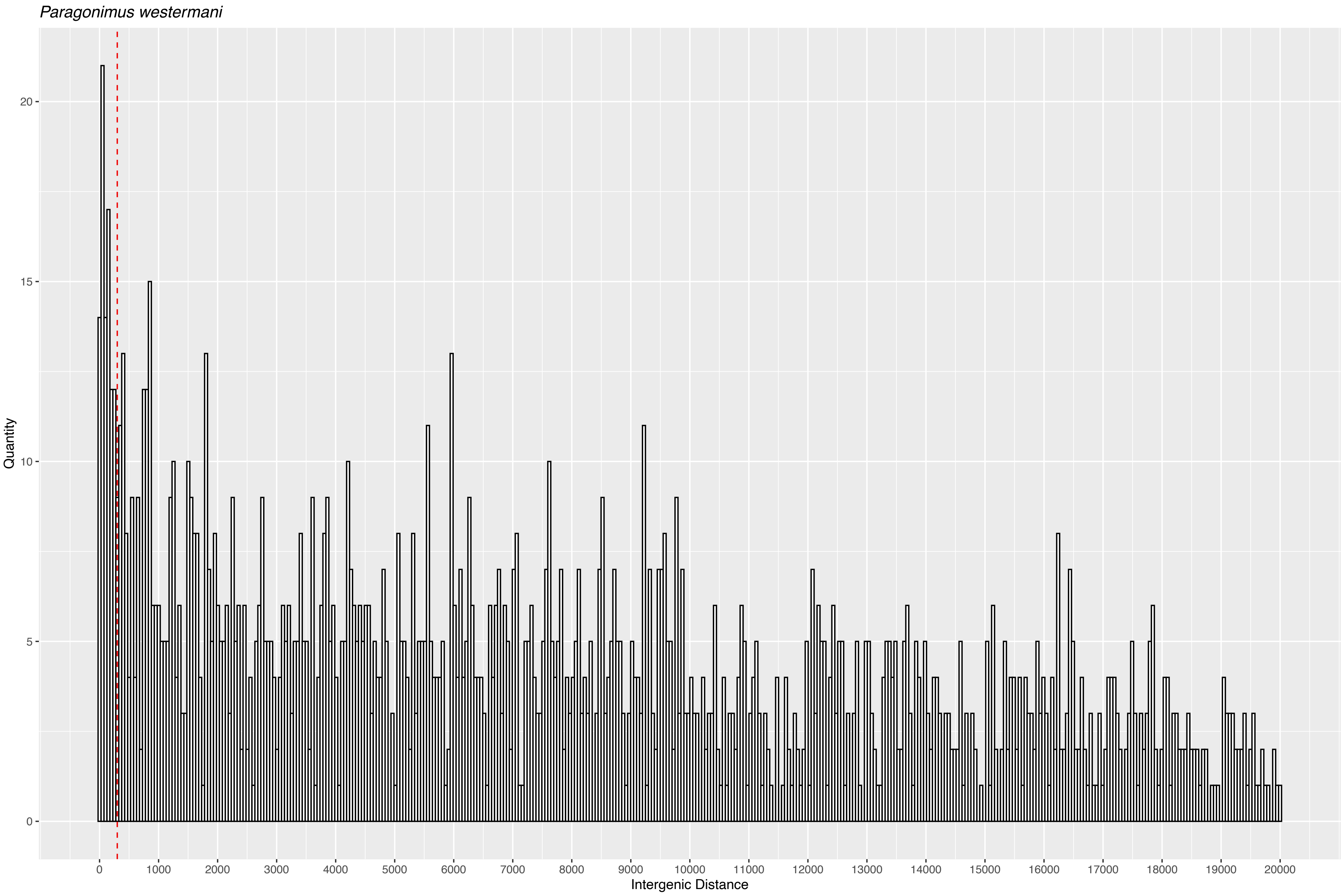

Paragonimus westermani
Quantity
Intergenic Distance
0
5
10
15
20
0
1000
2000
3000
4000
5000
6000
7000
8000
9000
10000
11000
12000
13000
14000
15000
16000
17000
18000
19000
20000

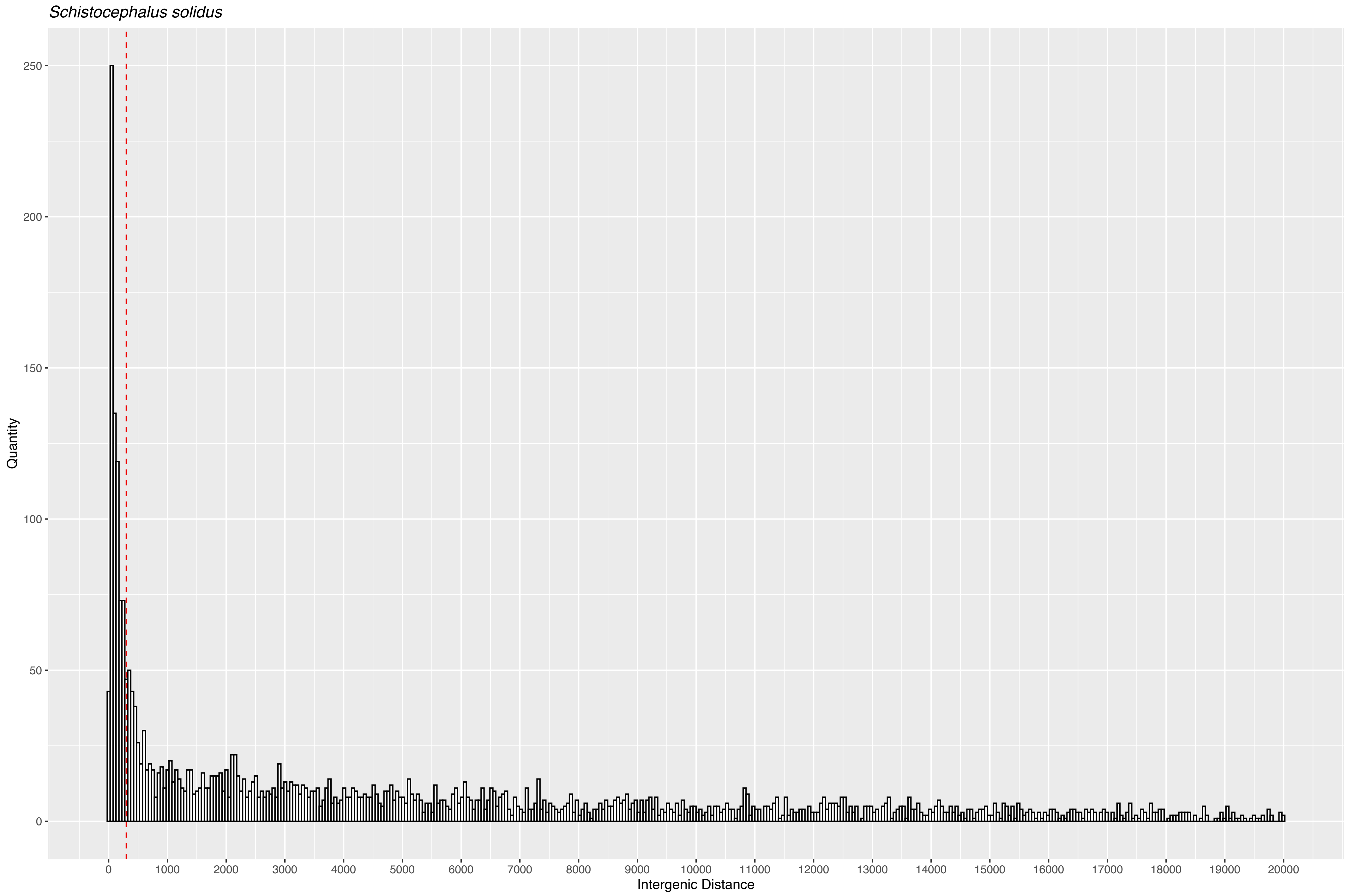

Schistocephalus solidus
Quantity
250
200
150
100
50
0
0
1000
2000
3000
4000
5000
6000
7000
8000
9000
10000
11000
12000
13000
14000
15000
16000
17000
18000
19000
20000
Intergenic Distance

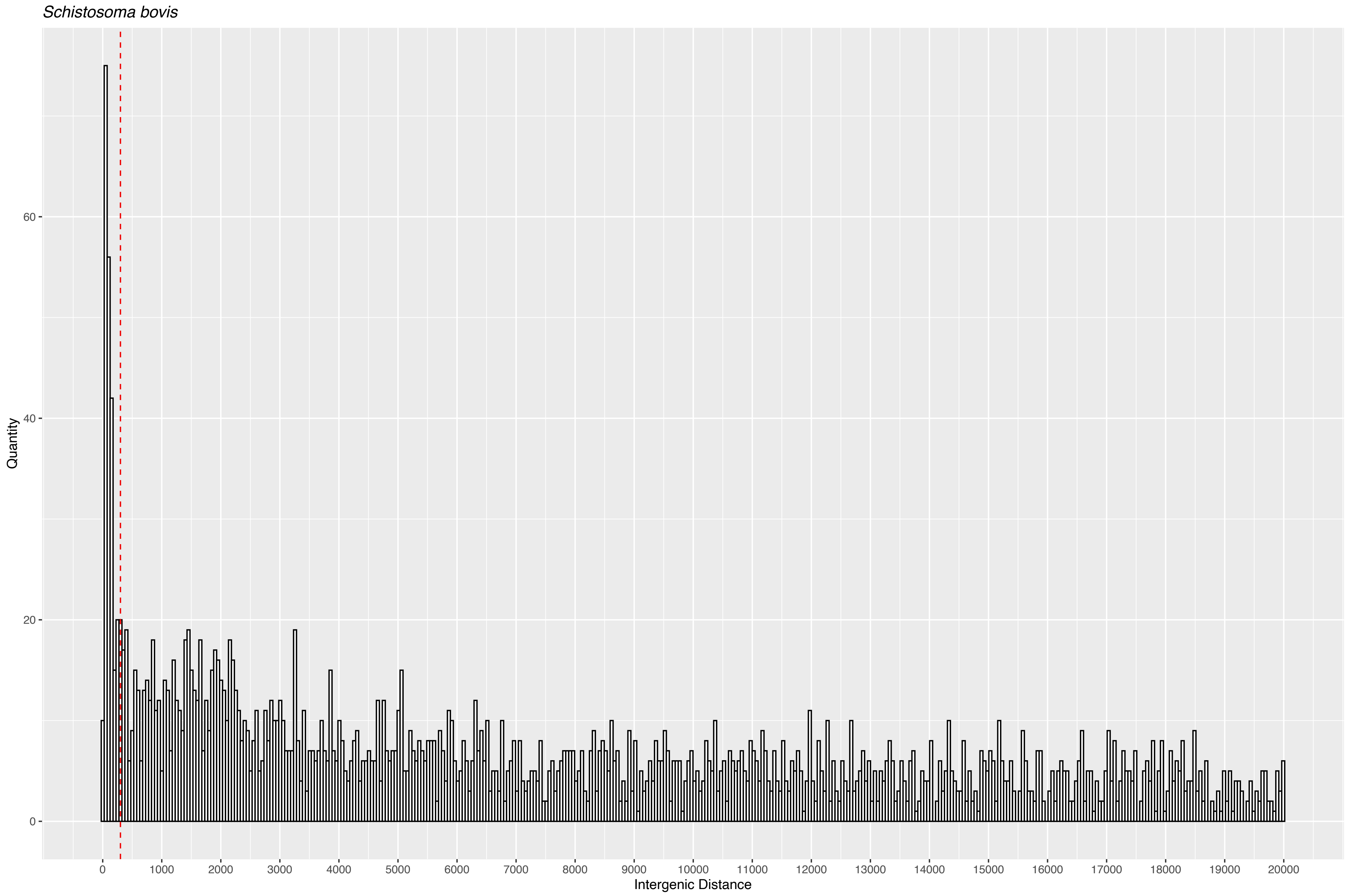
Schistosoma bovis
Quantity
0
20
40
60
0
1000
2000
3000
4000
5000
6000
7000
8000
9000
10000
11000
12000
13000
14000
15000
16000
17000
18000
19000
20000
Intergenic Distance

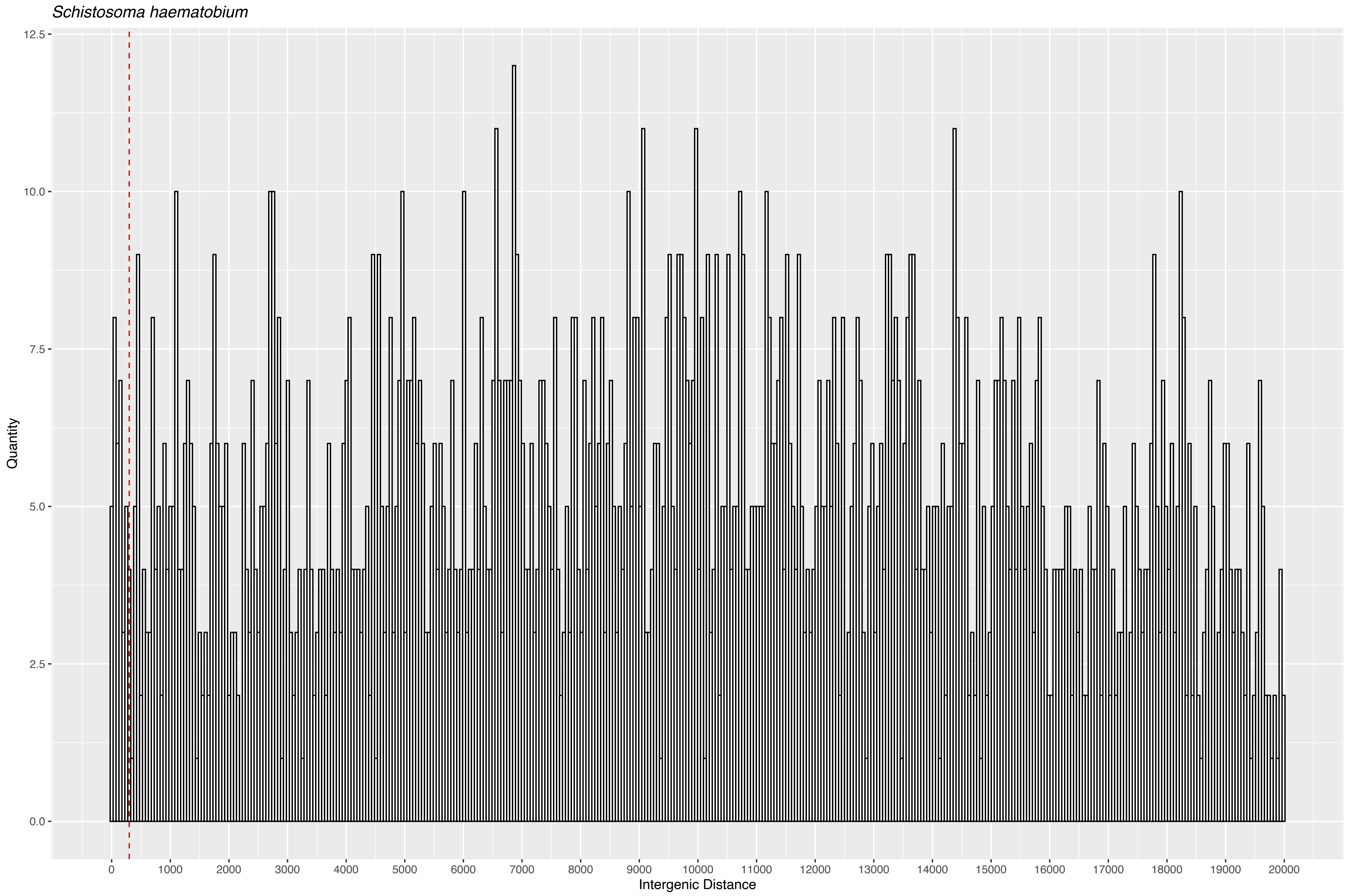

Schistosoma haematobium
Quantity
12.5
10.0
7.5
5.0
2.5
0.0
0
1000
2000
3000
4000
5000
6000
7000
8000
9000
10000
11000
12000
13000
14000
15000
16000
17000
18000
19000
20000
Intergenic Distance

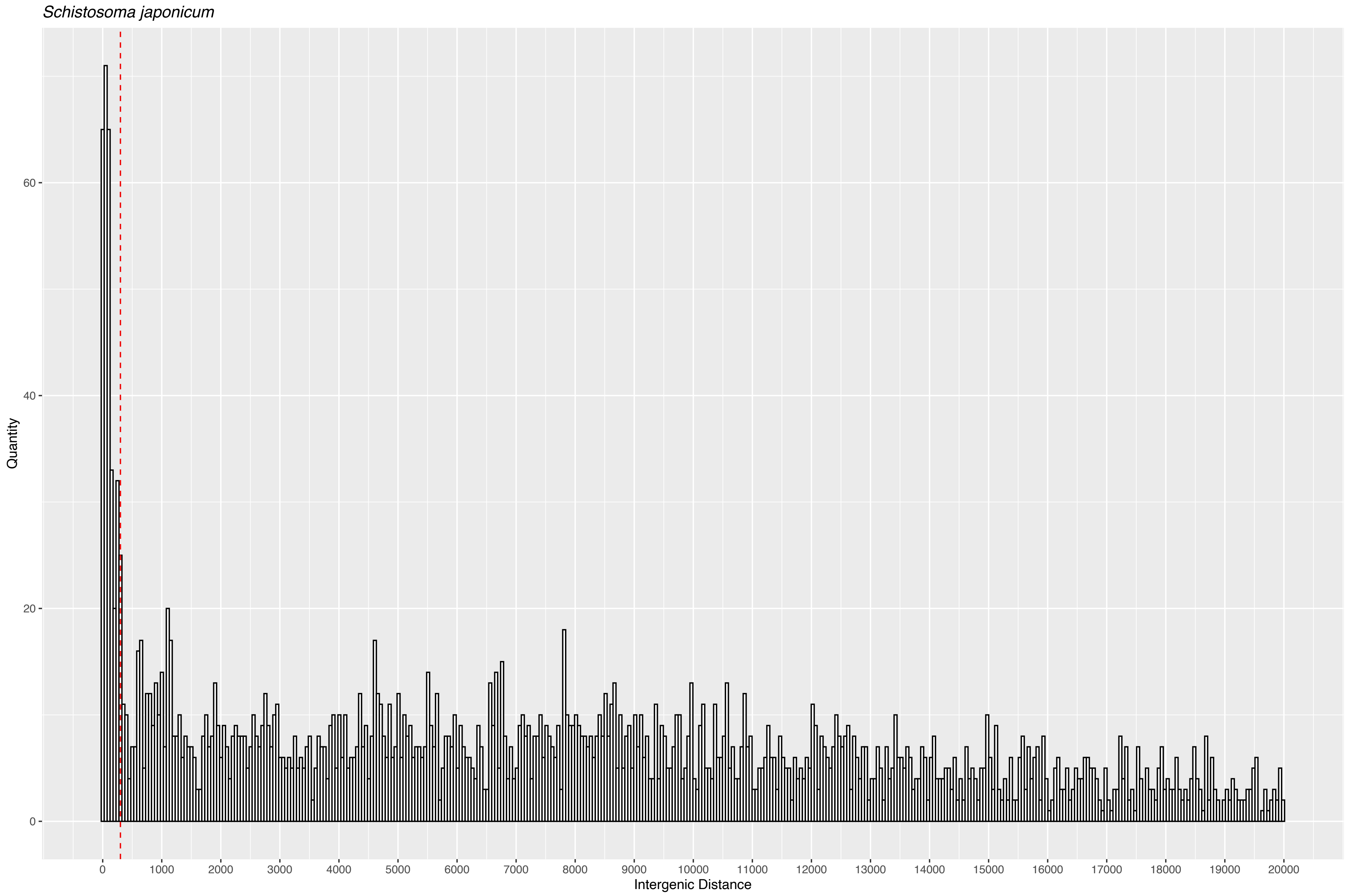

Schistosoma japonicum
Quantity
0
20
40
60
0
1000
2000
3000
4000
5000
6000
7000
8000
9000
10000
11000
12000
13000
14000
15000
16000
17000
18000
19000
20000
Intergenic Distance

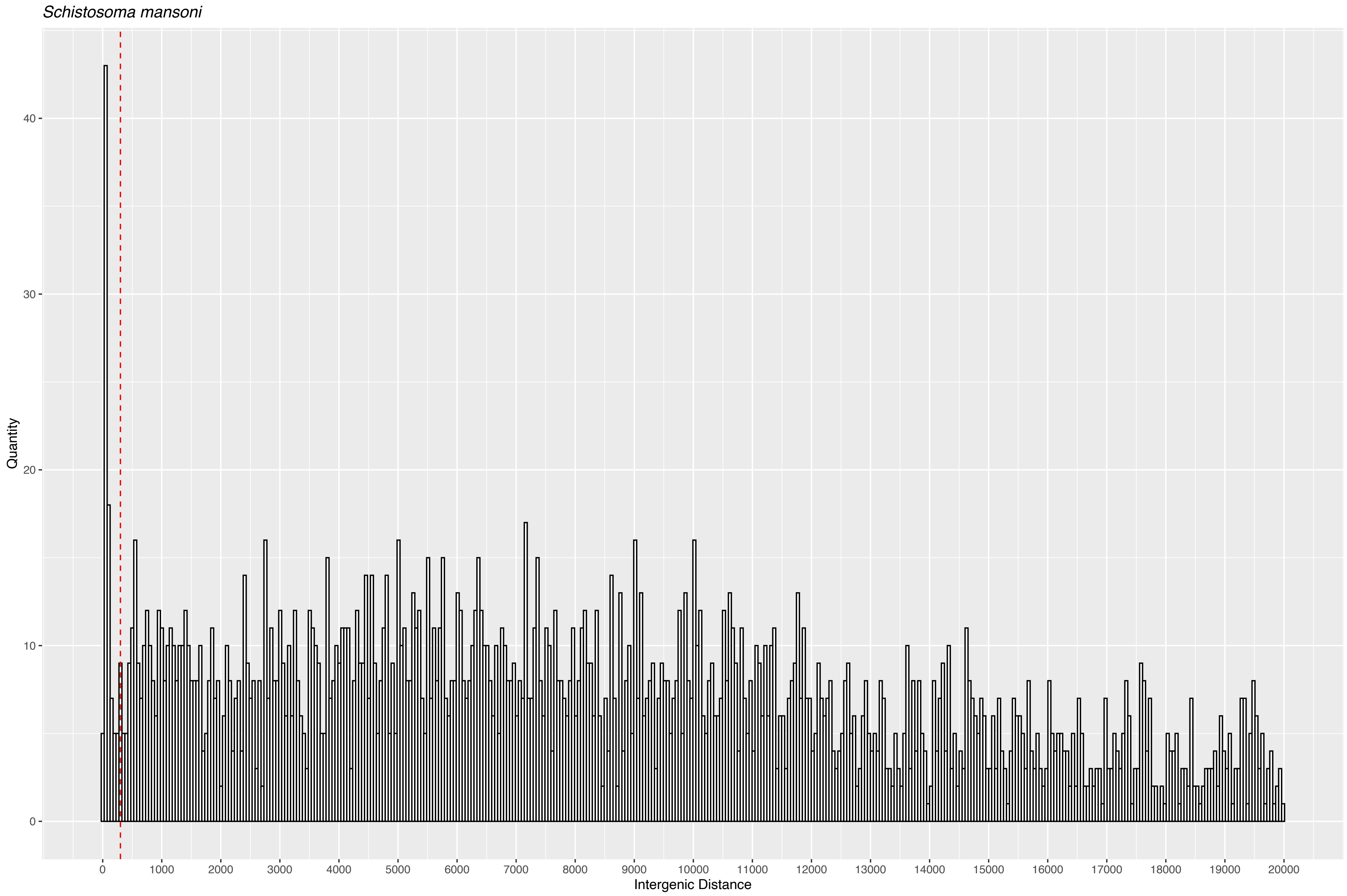

Schistosoma mansoni
Quantity
0
10
20
30
40
Intergenic Distance
0
1000
2000
3000
4000
5000
6000
7000
8000
9000
10000
11000
12000
13000
14000
15000
16000
17000
18000
19000
20000

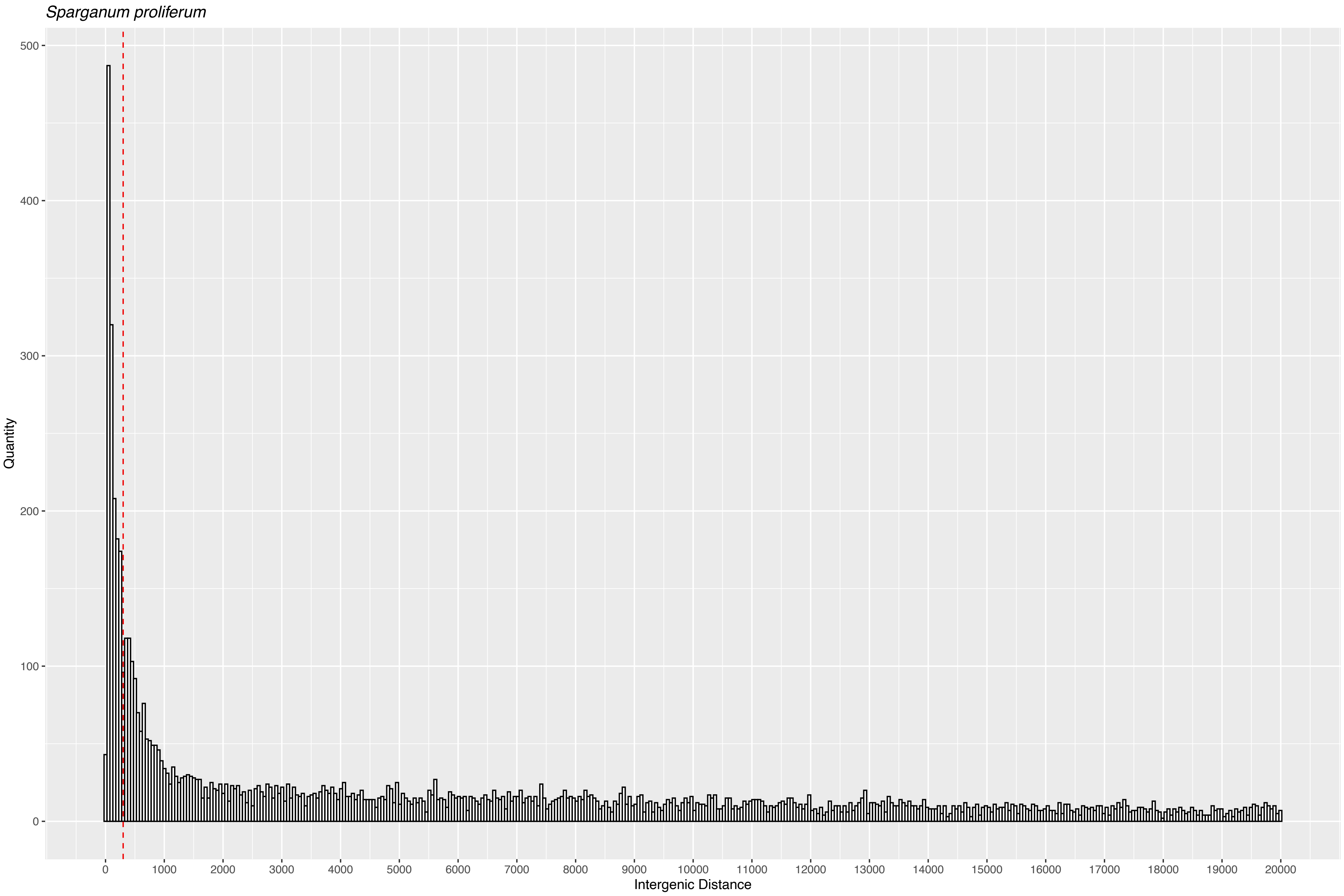

Sparganum proliferum
Quantity
0
100
200
300
400
500
0
1000
2000
3000
4000
5000
6000
7000
8000
9000
10000
11000
12000
13000
14000
15000
16000
17000
18000
19000
20000
Intergenic Distance

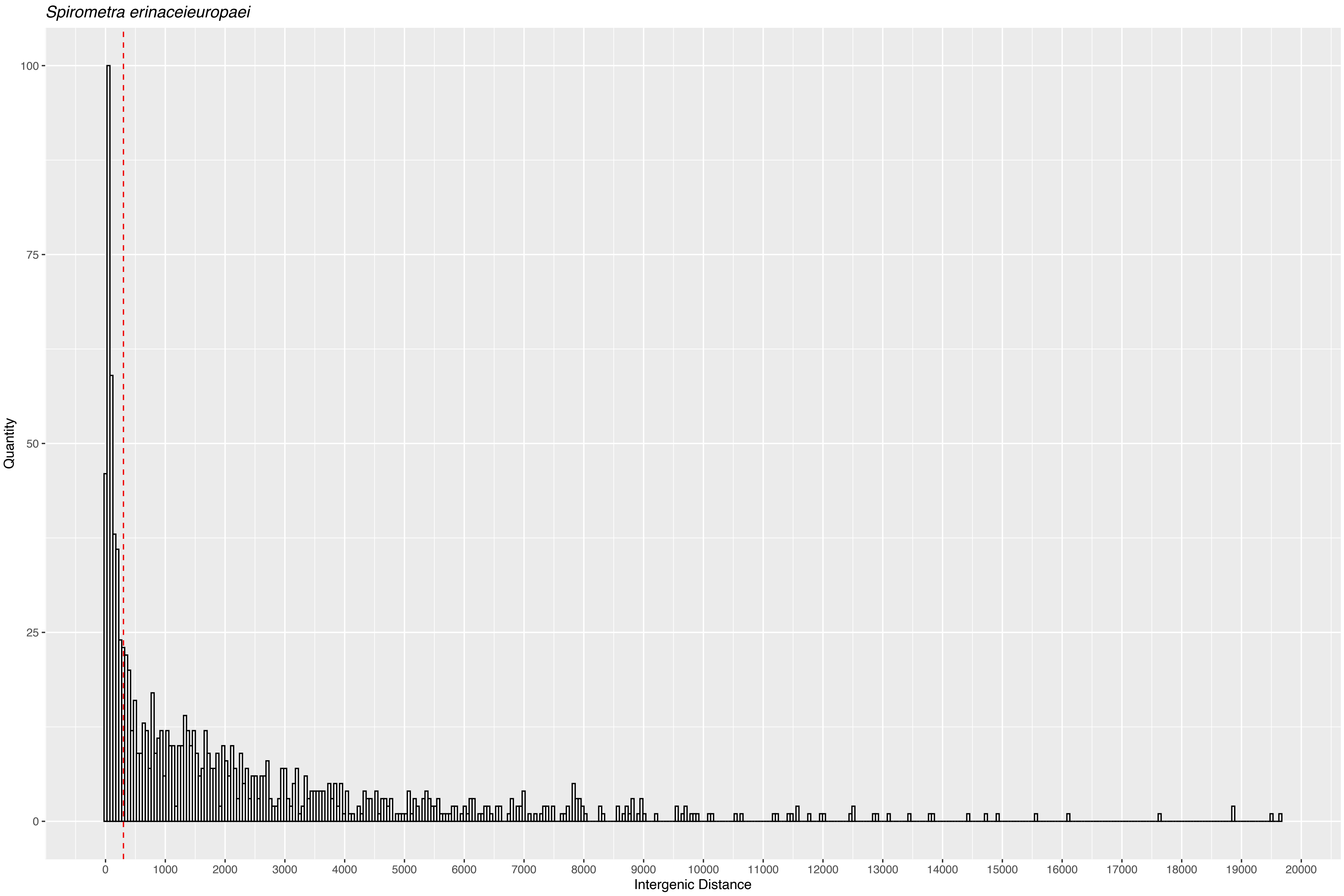

Spirometra erinaceieuropaei
Quantity
100
75
50
25
0
0
1000
2000
3000
4000
5000
6000
7000
8000
9000
10000
11000
12000
13000
14000
15000
16000
17000
18000
19000
20000
Intergenic Distance

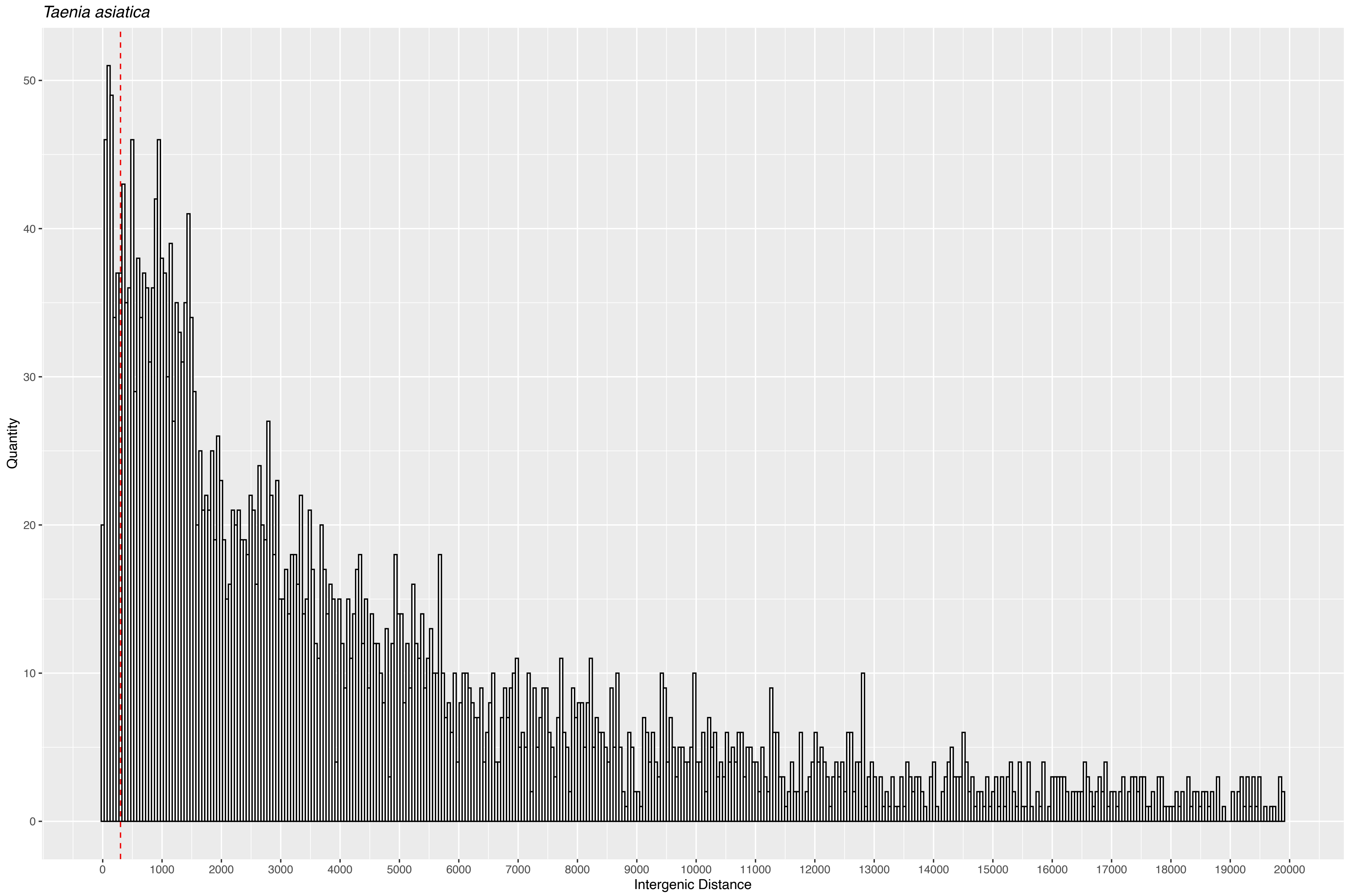

Taenia asiatica
Quantity
0
10
20
30
40
50
0
1000
2000
3000
4000
5000
6000
7000
8000
9000
10000
11000
12000
13000
14000
15000
16000
17000
18000
19000
20000
Intergenic Distance

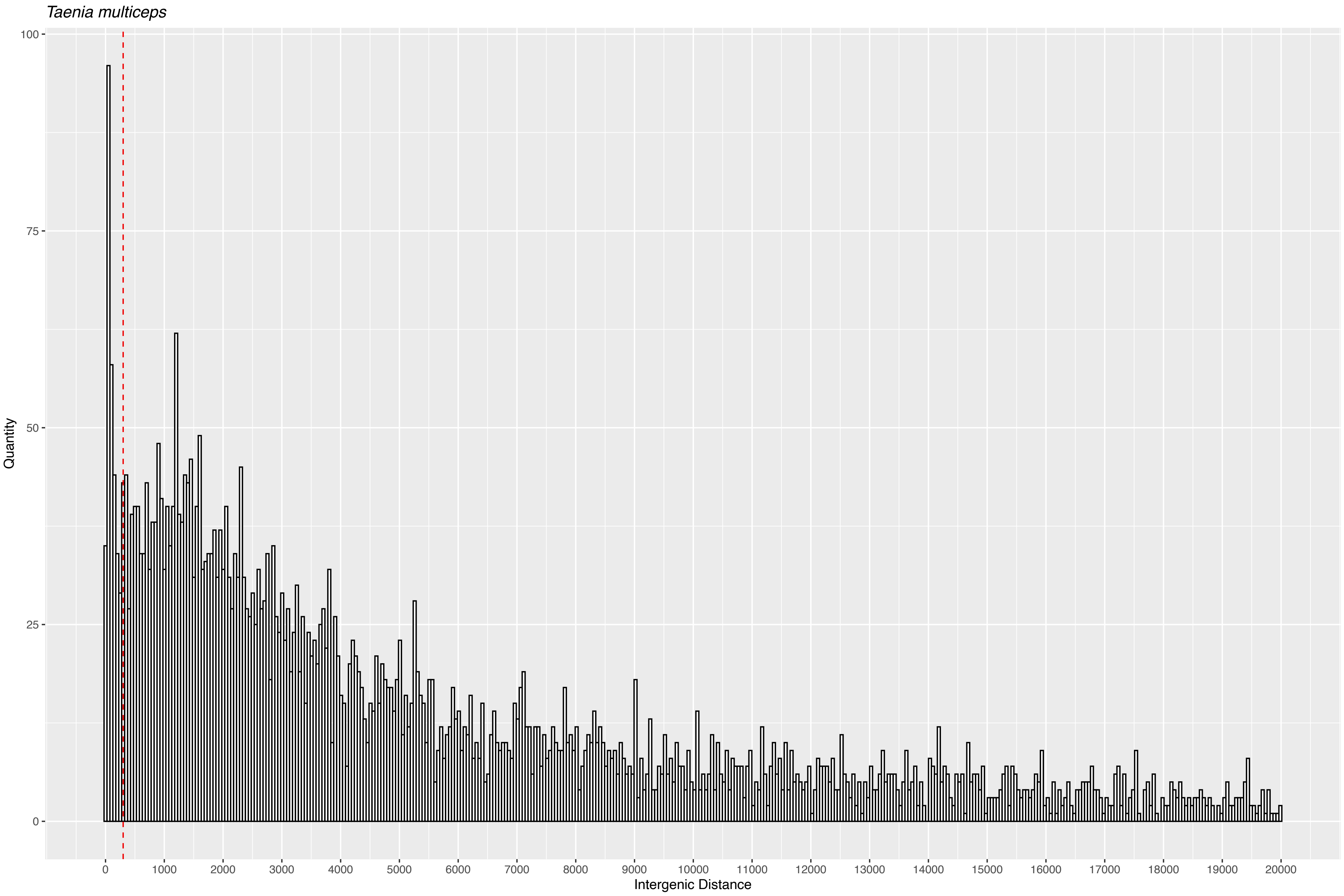

Taenia multiceps
Quantity
Intergenic Distance
100
75
50
25
0
0
1000
2000
3000
4000
5000
6000
7000
8000
9000
10000
11000
12000
13000
14000
15000
16000
17000
18000
19000
20000

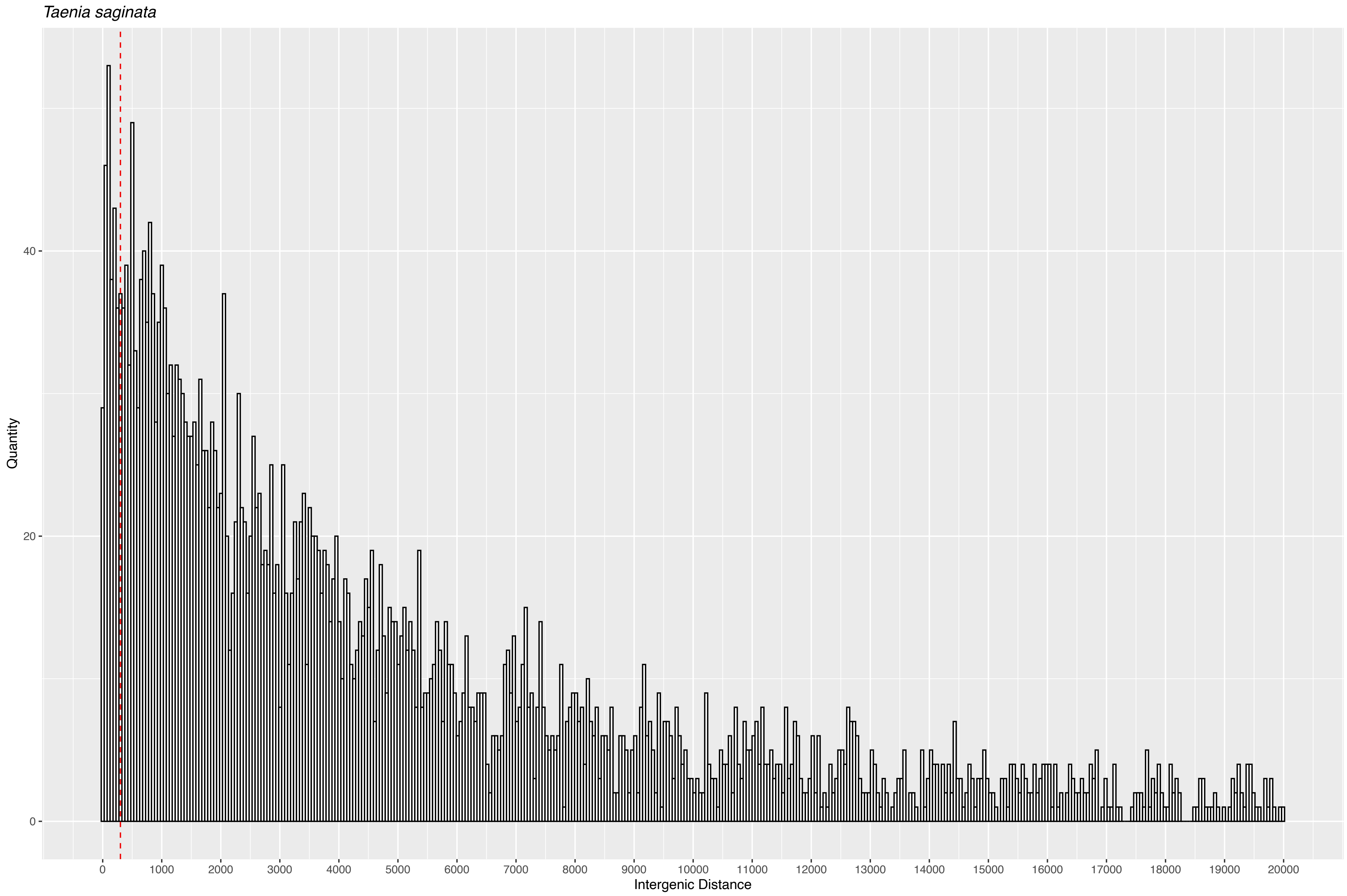

Taenia saginata
Quantity
0
20
40
Intergenic Distance
0
1000
2000
3000
4000
5000
6000
7000
8000
9000
10000
11000
12000
13000
14000
15000
16000
17000
18000
19000
20000

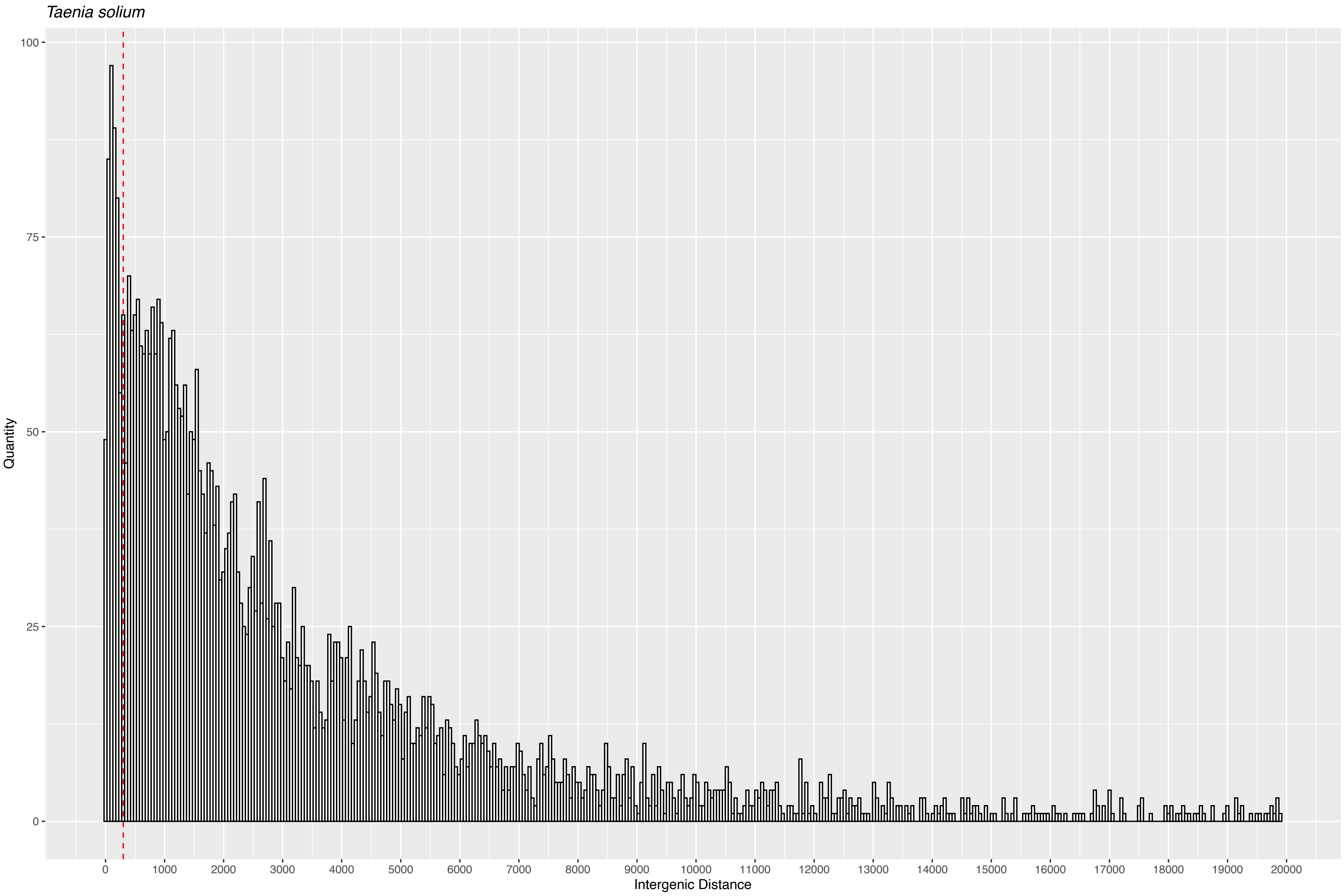

Taenia solium
Quantity
100
75
50
25
0
0
1000
2000
3000
4000
5000
6000
7000
8000
9000
10000
11000
12000
13000
14000
15000
16000
17000
18000
19000
20000
Intergenic Distance

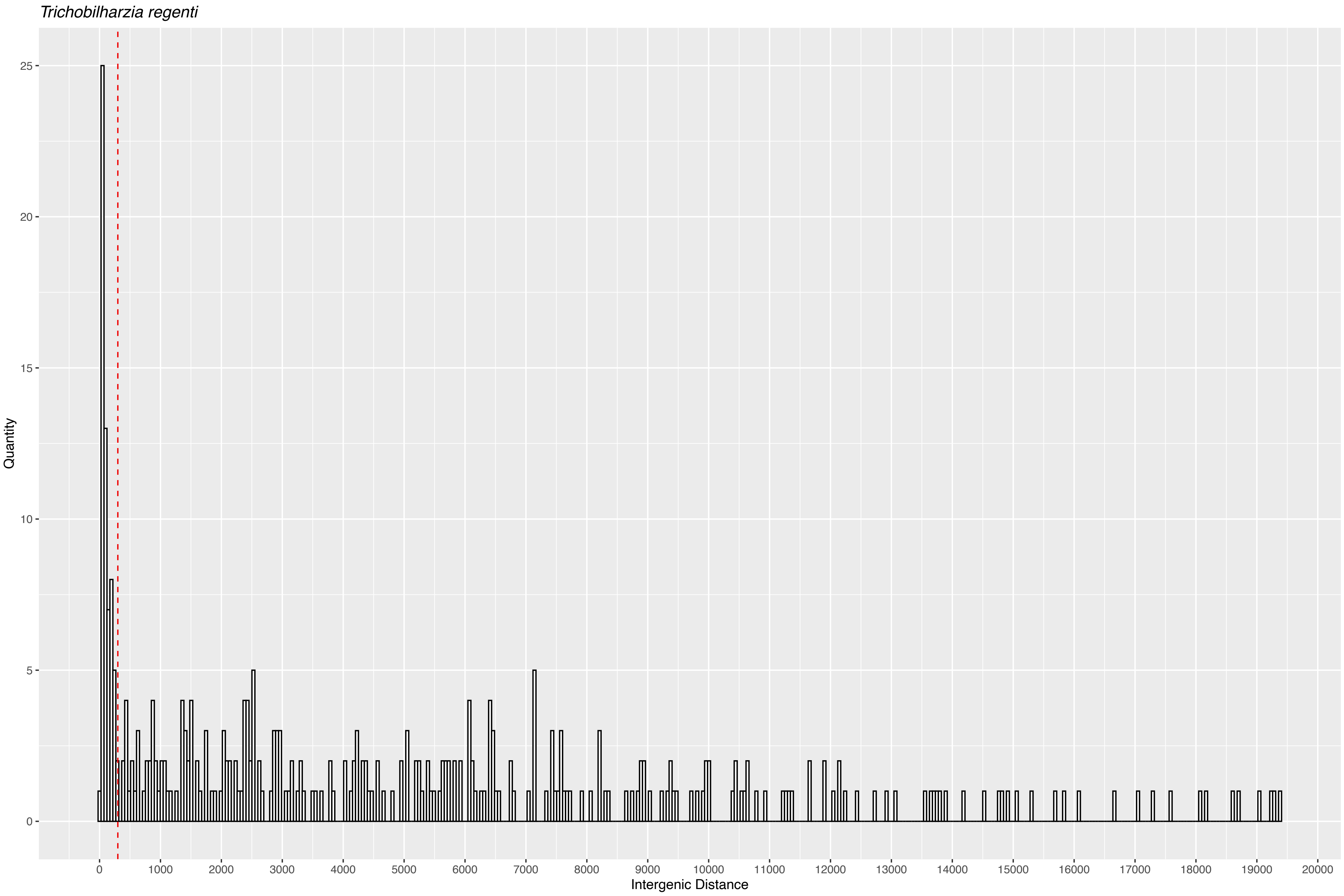

Trichobilharzia regenti
Quantity
0
5
10
15
20
25
0
1000
2000
3000
4000
5000
6000
7000
8000
9000
10000
11000
12000
13000
14000
15000
16000
17000
18000
19000
20000
Intergenic Distance
