# Supplementary material for: Evolution of SL-RNA Genes and Their Splicing Targets in Parasitic Flatworms
Source: Mol Biol Evol. 2025 Sep 23;42(11):msaf228. doi: 10.1093/molbev/msaf228 (PMC12582326; doi:10.1093/molbev/msaf228)
Supplement: msaf228_Supplementary_Data [file msaf228_supplementary_data.zip › Supplementary File 8 - 14082025.pdf]

Supplementary File 8: Maximum Likelihood phylogenetic trees of Phylogenetically Hierarchical Orthogroups (HOGs) selected based on their expression levels and evidence of SL trans-splicing. For each gene, evidence of SL trans-splicing is indicated by either a black star (above four reads on the same acceptor site) or a white star (below four reads). Additionally, a Normalized TPM relative to the median TPM of SL trans-spliced genes and the total number of SL-bearing reads in logarithmic scale are displayed with green and red bars, respectively. Both values were capped (at 10 for the normalized TPM and 100 for the SL-bearing reads), which are indicated by circles. Genes identified as operon candidates are marked with a blue check mark.

Index:

Pag. 2: N0.HOG0000771

Pag. 3: N0.HOG0000823

Pag. 4: N0.HOG0000874

Pag. 5: N0.HOG0000876

Pag. 6: N0.HOG0000970

Pag. 7: N0.HOG0001389

Pag. 8: N0.HOG0002036

Pag. 9: N0.HOG0002250

Pag. 10: N0.HOG0002707

Pag. 11: N0.HOG0003375

Pag. 12: N0.HOG0004575

Pag. 13: N0.HOG0005209

Pag. 14: N0.HOG0006807

Pag. 15: N0.HOG0007522

Pag. 16: N0.HOG0007613

Pag. 17: N0.HOG0007875

Pag. 18: N0.HOG0007943

Pag. 19: N0.HOG0008182

Pag. 20: N0.HOG0008418

Pag. 21: N0.HOG0008512

Pag. 22: N0.HOG0008654

Pag. 23: N0.HOG0009450

Pag. 24: N0.HOG0009887

Pag. 25: N0.HOG0009968

Pag. 26: N0.HOG0010306

Pag. 27: N0.HOG0010318

Pag. 28: N0.HOG0010377

Pag. 29: N0.HOG0010496

Pag. 30: N0.HOG0010643

Pag. 31: N0.HOG0010669

Pag. 32: N0.HOG0010755

Pag. 33: N0.HOG0010762

Pag. 34: N0.HOG0010786

Pag. 35: N0.HOG0010915

Pag. 36: N0.HOG0010953

Pag. 37: N0.HOG0011542

Pag. 38: N0.HOG0011745

Pag. 39: N0.HOG0011763

Pag. 40: N0.HOG0011930

Pag. 41: N0.HOG0012348

Pag. 42: N0.HOG0012854

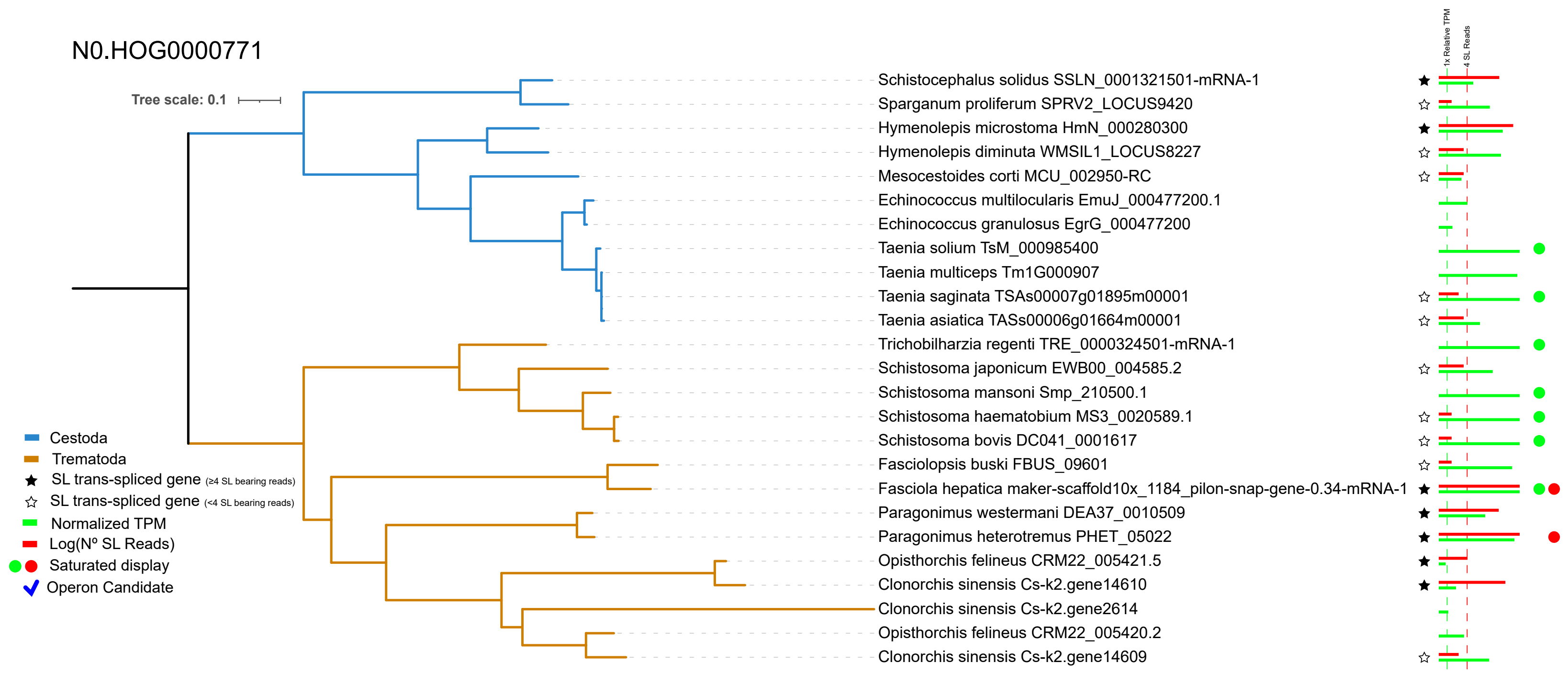

N0.HOG0000771
Tree scale: 0.1
Schistocephalus solidus SSLN_0001321501-mRNA-1
Sparganum proliferum SPRV2_LOCUS9420
Hymenolepis microstoma HmN_000280300
Hymenolepis diminuta WMSIL1_LOCUS8227
Mesocestoides corti MCU_002950-RC
Echinococcus multilocularis EmuJ_000477200.1
Echinococcus granulosus EgrG_000477200
Taenia solium TsM_000985400
Taenia multiceps Tm1G000907
Taenia saginata TSAs00007g01895m00001
Taenia asiatica TASs00006g01664m00001
Trichobilharzia regenti TRE_0000324501-mRNA-1
Schistosoma japonicum EWB00_004585.2
Schistosoma mansoni Smp_210500.1
Schistosoma haematobium MS3_0020589.1
Schistosoma bovis DC041_0001617
Fasciolopsis buski FBUS_09601
Fasciola hepatica maker-scaffold10x_1184_pilon-snap-gene-0.34-mRNA-1
Paragonimus westermani DEA37_0010509
Paragonimus heterotremus PHET_05022
Opisthorchis felineus CRM22_005421.5
Clonorchis sinensis Cs-k2.gene14610
Clonorchis sinensis Cs-k2.gene2614
Opisthorchis felineus CRM22_005420.2
Clonorchis sinensis Cs-k2.gene14609
1x Relative TPM
4 SL Reads
Cestoda
Trematoda
SL trans-spliced gene (≥4 SL bearing reads)
SL trans-spliced gene (<4 SL bearing reads)
Normalized TPM
Log(Nº SL Reads)
Saturated display
Operon Candidate

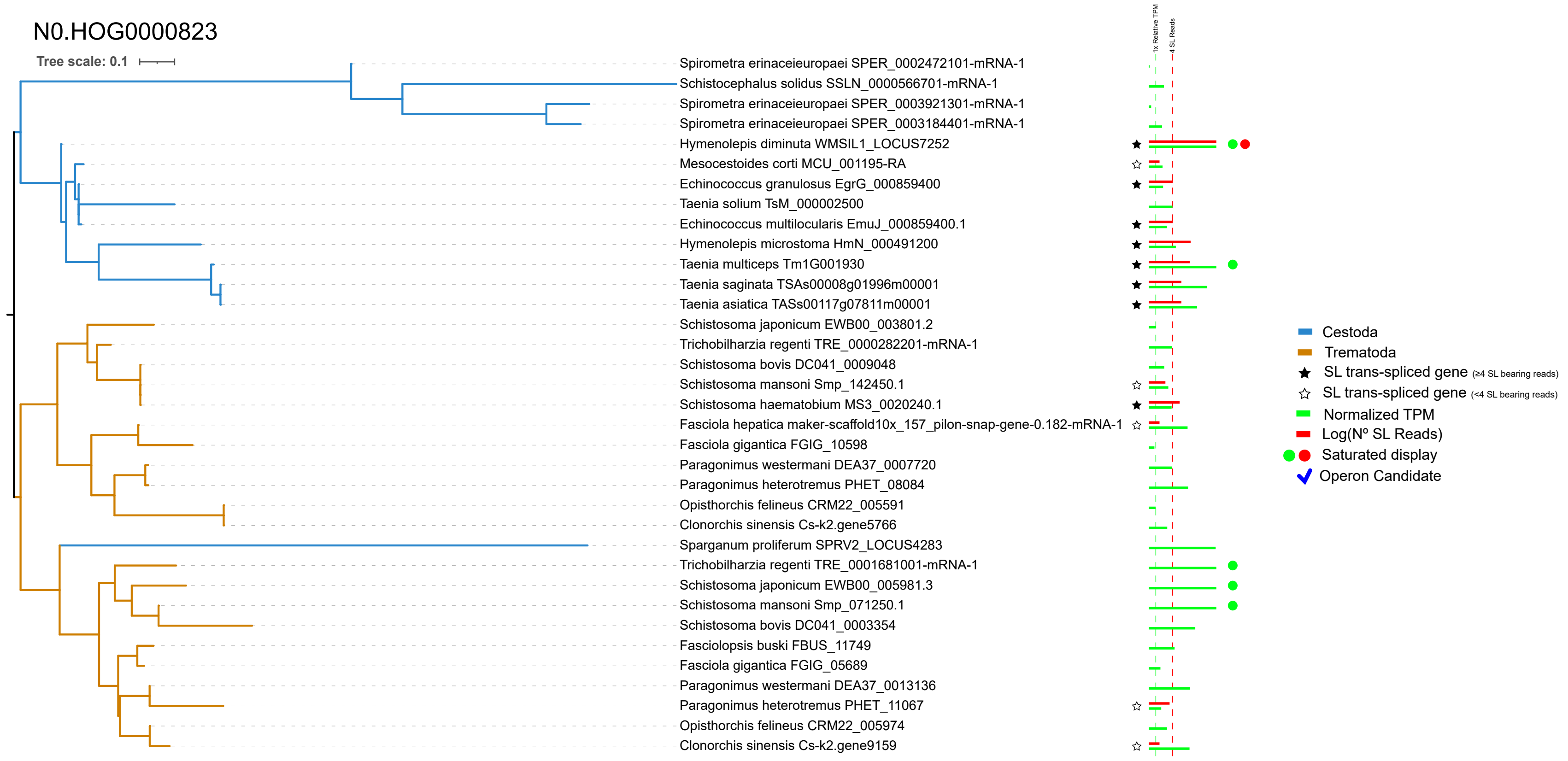
N0.HOG0000823
Tree scale: 0.1
1x Relative TPM
4 SL Reads
Spirometra erinaceieuropaei SPER_0002472101-mRNA-1
Schistocephalus solidus SSLN_0000566701-mRNA-1
Spirometra erinaceieuropaei SPER_0003921301-mRNA-1
Spirometra erinaceieuropaei SPER_0003184401-mRNA-1
Hymenolepis diminuta WMSIL1_LOCUS7252
Mesocestoides corti MCU_001195-RA
Echinococcus granulosus EgrG_000859400
Taenia solium TsM_000002500
Echinococcus multilocularis EmuJ_000859400.1
Hymenolepis microstoma HmN_000491200
Taenia multiceps Tm1G001930
Taenia saginata TSAs00008g01996m00001
Taenia asiatica TASs00117g07811m00001
Schistosoma japonicum EWB00_003801.2
Trichobilharzia regenti TRE_0000282201-mRNA-1
Schistosoma bovis DC041_0009048
Schistosoma mansoni Smp_142450.1
Schistosoma haematobium MS3_0020240.1
Fasciola hepatica maker-scaffold10x_157_pilon-snap-gene-0.182-mRNA-1
Fasciola gigantica FGIG_10598
Paragonimus westermani DEA37_0007720
Paragonimus heterotremus PHET_08084
Opisthorchis felineus CRM22_005591
Clonorchis sinensis Cs-k2.gene5766
Sparganum proliferum SPRV2_LOCUS4283
Trichobilharzia regenti TRE_0001681001-mRNA-1
Schistosoma japonicum EWB00_005981.3
Schistosoma mansoni Smp_071250.1
Schistosoma bovis DC041_0003354
Fasciolopsis buski FBUS_11749
Fasciola gigantica FGIG_05689
Paragonimus westermani DEA37_0013136
Paragonimus heterotremus PHET_11067
Opisthorchis felineus CRM22_005974
Clonorchis sinensis Cs-k2.gene9159
Cestoda
Trematoda
SL trans-spliced gene (≥4 SL bearing reads)
SL trans-spliced gene (<4 SL bearing reads)
Normalized TPM
Log(Nº SL Reads)
Saturated display
Operon Candidate

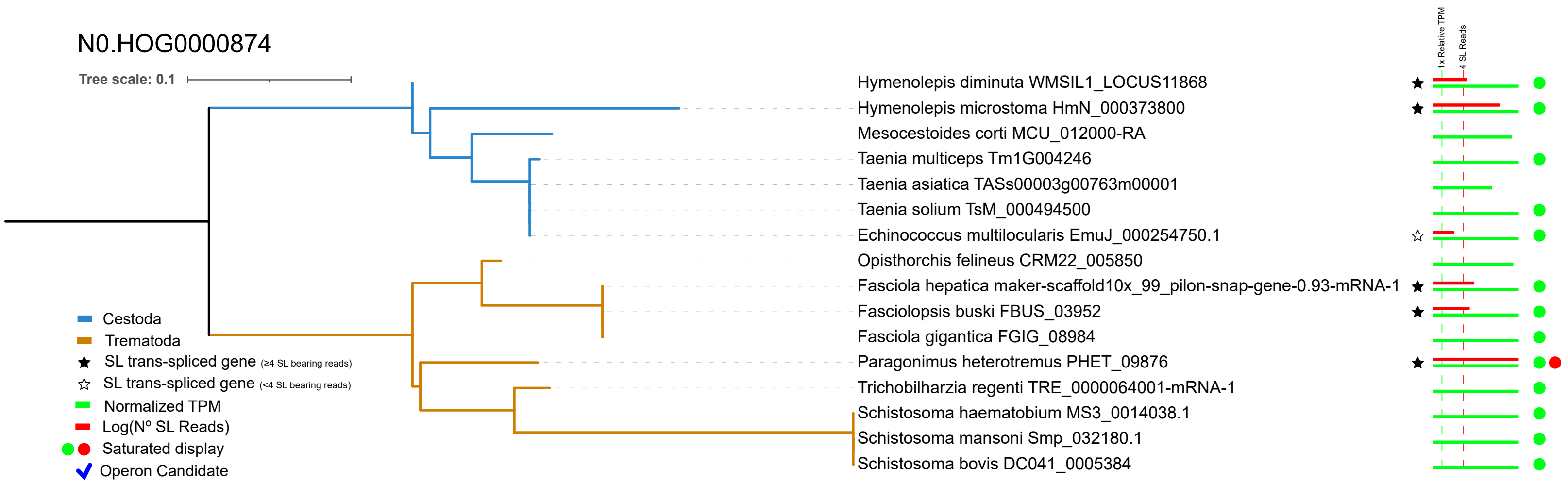
N0.HOG0000874
Tree scale: 0.1
1x Relative TPM
4 SL Reads
Hymenolepis diminuta WMSIL1_LOCUS11868
Hymenolepis microstoma HmN_000373800
Mesocestoides corti MCU_012000-RA
Taenia multiceps Tm1G004246
Taenia asiatica TASs00003g00763m00001
Taenia solium TsM_000494500
Echinococcus multilocularis EmuJ_000254750.1
Opisthorchis felineus CRM22_005850
Fasciola hepatica maker-scaffold10x_99_pilon-snap-gene-0.93-mRNA-1
Fasciolopsis buski FBUS_03952
Fasciola gigantica FGIG_08984
Paragonimus heterotremus PHET_09876
Trichobilharzia regenti TRE_0000064001-mRNA-1
Schistosoma haematobium MS3_0014038.1
Schistosoma mansoni Smp_032180.1
Schistosoma bovis DC041_0005384
Cestoda
Trematoda
SL trans-spliced gene (≥4 SL bearing reads)
SL trans-spliced gene (<4 SL bearing reads)
Normalized TPM
Log(Nº SL Reads)
Saturated display
Operon Candidate

N0.HOG0000876

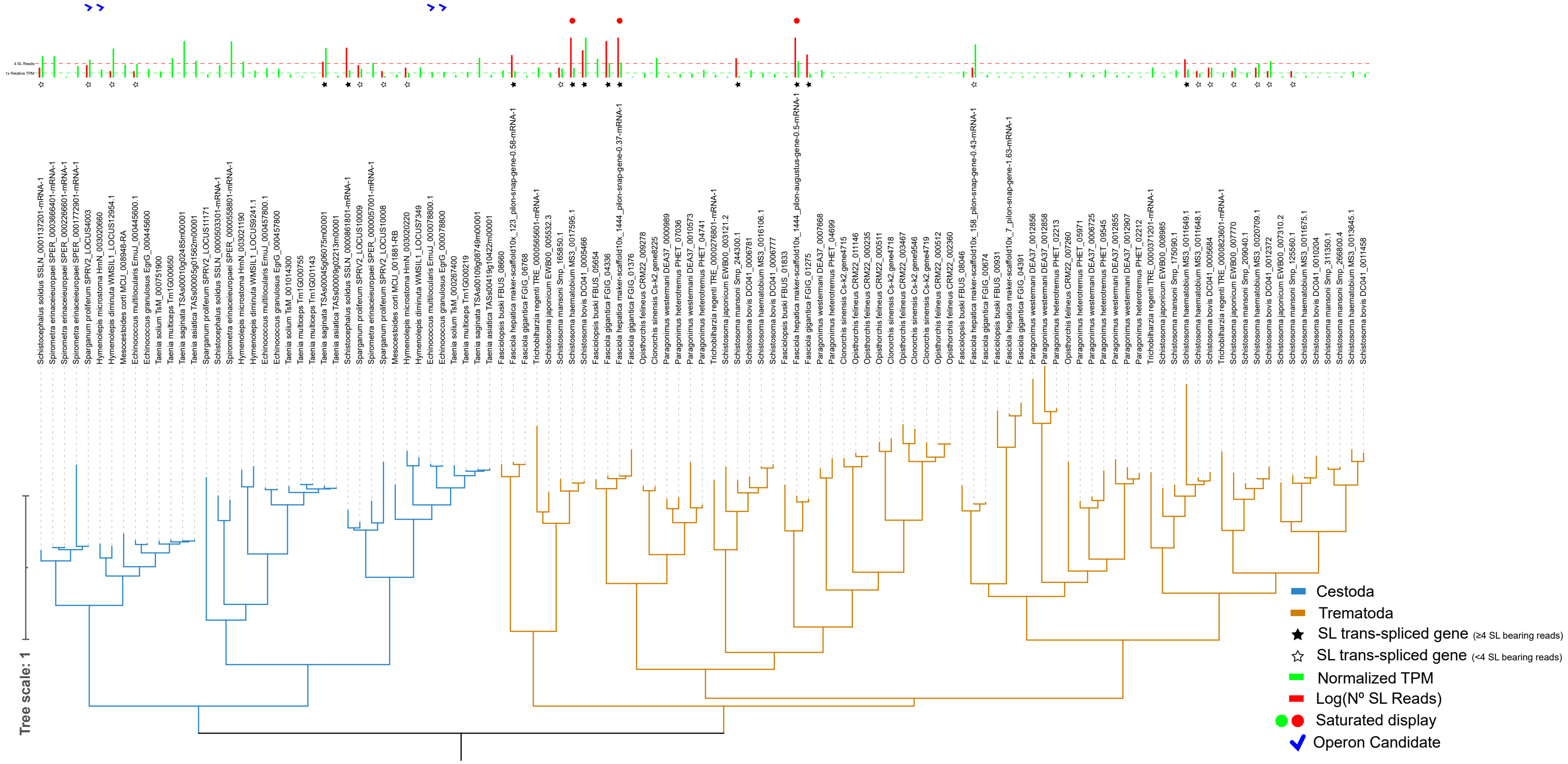

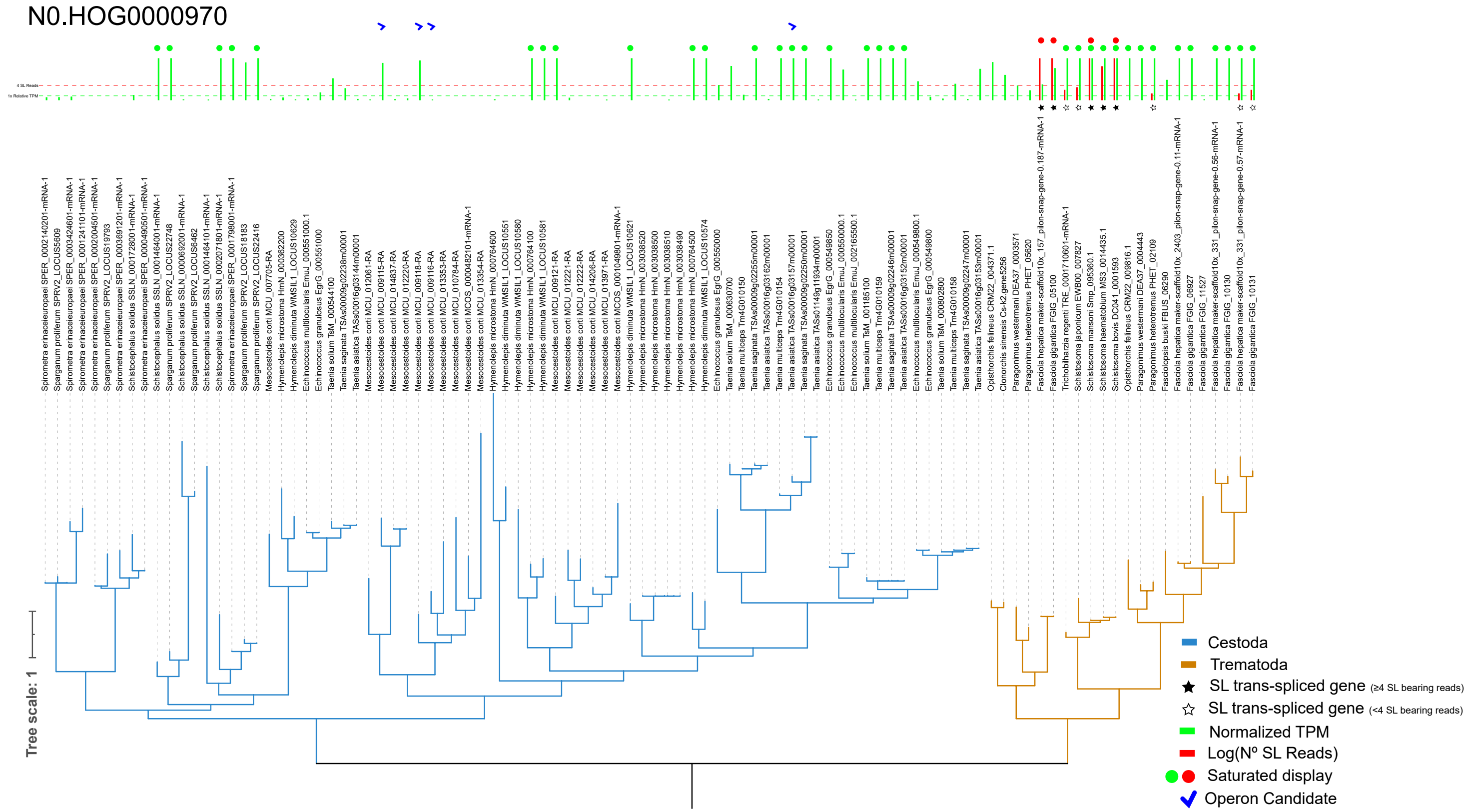
N0.HOG0000970
Tree scale: 1
Cestoda
Trematoda
SL trans-spliced gene (≥4 SL bearing reads)
SL trans-spliced gene (<4 SL bearing reads)
Normalized TPM
Log(Nº SL Reads)
Saturated display
Operon Candidate

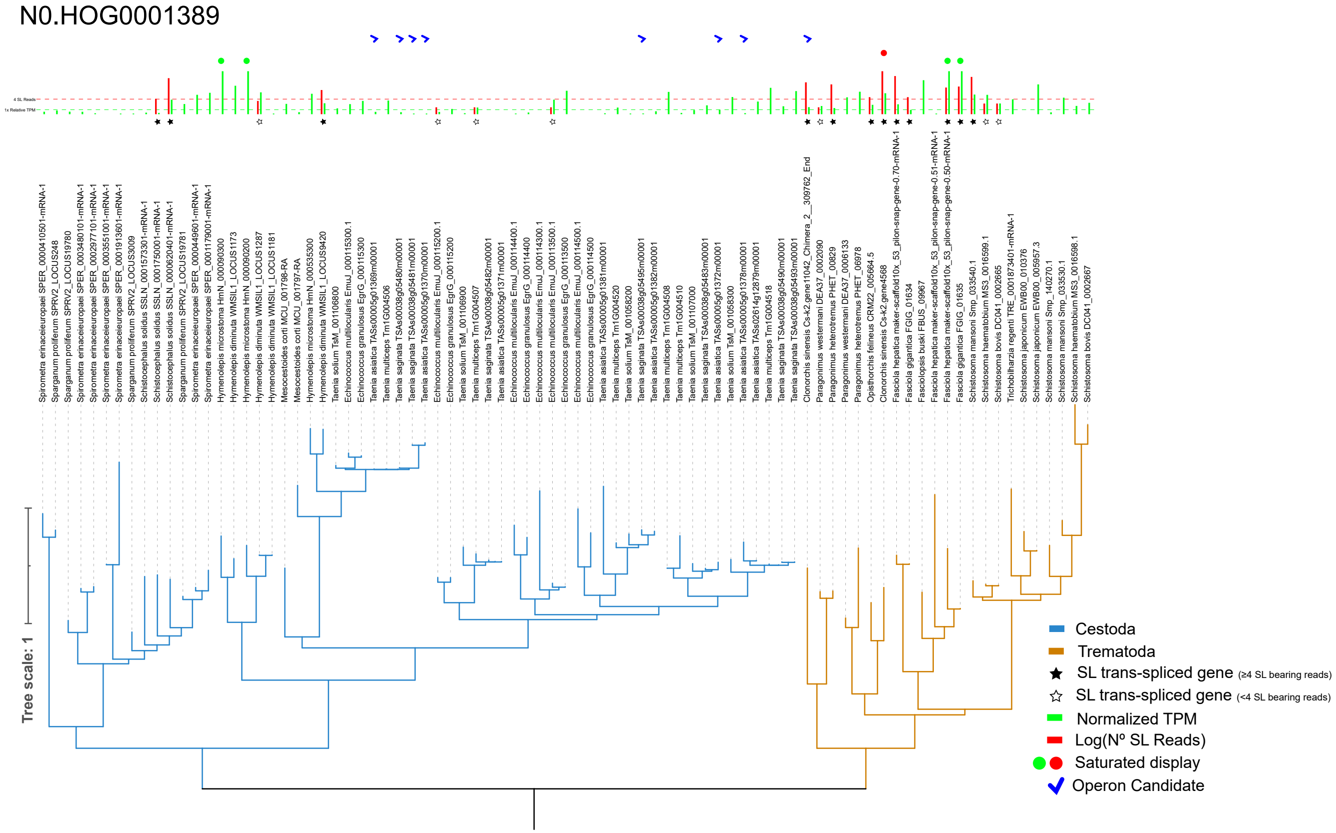
N0.HOG0001389
Tree scale: 1
Cestoda
Trematoda
SL trans-spliced gene (≥4 SL bearing reads)
SL trans-spliced gene (<4 SL bearing reads)
Normalized TPM
Log(Nº SL Reads)
Saturated display
Operon Candidate

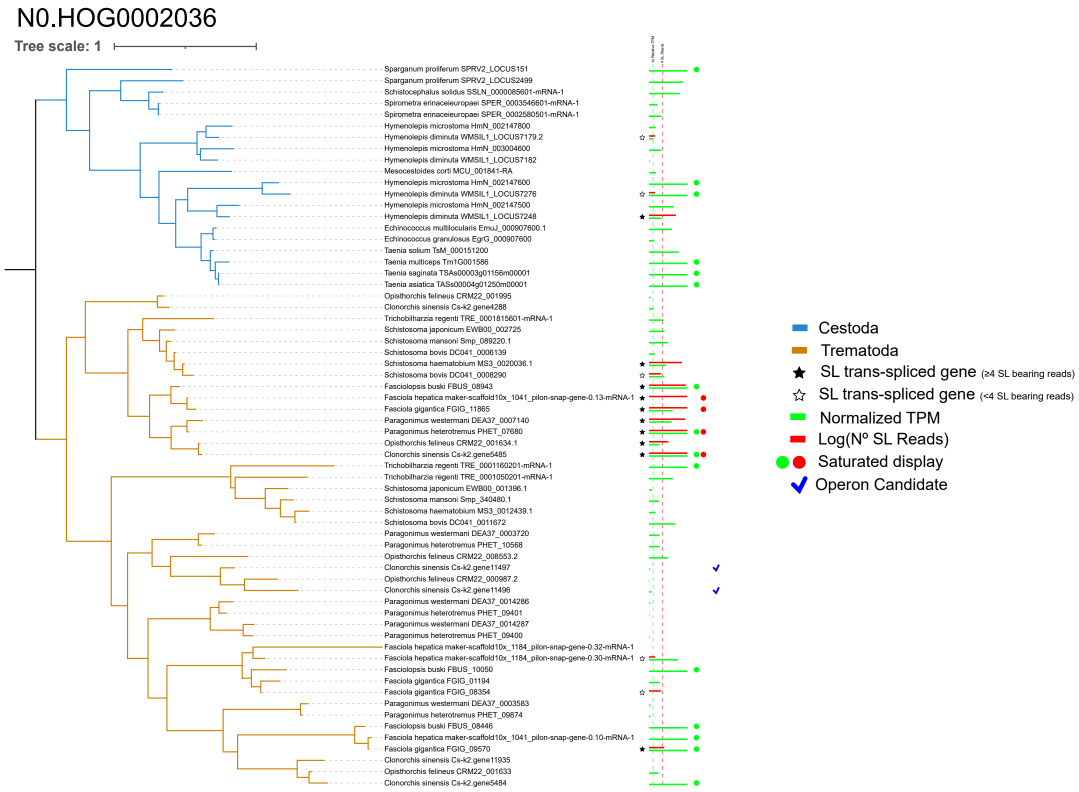
N0.HOG0002036
Tree scale: 1
1x Relative TPM
4 SL Reads
Sparganum proliferum SPRV2_LOCUS151
Sparganum proliferum SPRV2_LOCUS2499
Schistocephalus solidus SSLN_0000085601-mRNA-1
Spirometra erinaceieuropaei SPER_0003546601-mRNA-1
Spirometra erinaceieuropaei SPER_0002580501-mRNA-1
Hymenolepis microstoma HmN_002147800
Hymenolepis diminuta WMSIL1_LOCUS7179.2
Hymenolepis microstoma HmN_003004600
Hymenolepis diminuta WMSIL1_LOCUS7182
Mesocestoides corti MCU_001841-RA
Hymenolepis microstoma HmN_002147600
Hymenolepis diminuta WMSIL1_LOCUS7276
Hymenolepis microstoma HmN_002147500
Hymenolepis diminuta WMSIL1_LOCUS7248
Echinococcus multilocularis EmuJ_000907600.1
Echinococcus granulosus EgrG_000907600
Taenia solium TsM_000151200
Taenia multiceps Tm1G001586
Taenia saginata TSAs00003g01156m00001
Taenia asiatica TASs00004g01250m00001
Opisthorchis felineus CRM22_001995
Clonorchis sinensis Cs-k2.gene4288
Trichobilharzia regenti TRE_0001815601-mRNA-1
Schistosoma japonicum EWB00_002725
Schistosoma mansoni Smp_089220.1
Schistosoma bovis DC041_0006139
Schistosoma haematobium MS3_0020036.1
Schistosoma bovis DC041_0008290
Fasciolopsis buski FBUS_08943
Fasciola hepatica maker-scaffold10x_1041_pilon-snap-gene-0.13-mRNA-1
Fasciola gigantica FGIG_11865
Paragonimus westermani DEA37_0007140
Paragonimus heterotremus PHET_07680
Opisthorchis felineus CRM22_001634.1
Clonorchis sinensis Cs-k2.gene5485
Trichobilharzia regenti TRE_0001160201-mRNA-1
Trichobilharzia regenti TRE_0001050201-mRNA-1
Schistosoma japonicum EWB00_001396.1
Schistosoma mansoni Smp_340480.1
Schistosoma haematobium MS3_0012439.1
Schistosoma bovis DC041_0011672
Paragonimus westermani DEA37_0003720
Paragonimus heterotremus PHET_10568
Opisthorchis felineus CRM22_008553.2
Clonorchis sinensis Cs-k2.gene11497
Opisthorchis felineus CRM22_000987.2
Clonorchis sinensis Cs-k2.gene11496
Paragonimus westermani DEA37_0014286
Paragonimus heterotremus PHET_09401
Paragonimus westermani DEA37_0014287
Paragonimus heterotremus PHET_09400
Fasciola hepatica maker-scaffold10x_1184_pilon-snap-gene-0.32-mRNA-1
Fasciola hepatica maker-scaffold10x_1184_pilon-snap-gene-0.30-mRNA-1
Fasciolopsis buski FBUS_10050
Fasciola gigantica FGIG_01194
Fasciola gigantica FGIG_08354
Paragonimus westermani DEA37_0003583
Paragonimus heterotremus PHET_09874
Fasciolopsis buski FBUS_08446
Fasciola hepatica maker-scaffold10x_1041_pilon-snap-gene-0.10-mRNA-1
Fasciola gigantica FGIG_09570
Clonorchis sinensis Cs-k2.gene11935
Opisthorchis felineus CRM22_001633
Clonorchis sinensis Cs-k2.gene5484
Cestoda
Trematoda
SL trans-spliced gene (≥4 SL bearing reads)
SL trans-spliced gene (<4 SL bearing reads)
Normalized TPM
Log(Nº SL Reads)
Saturated display
Operon Candidate

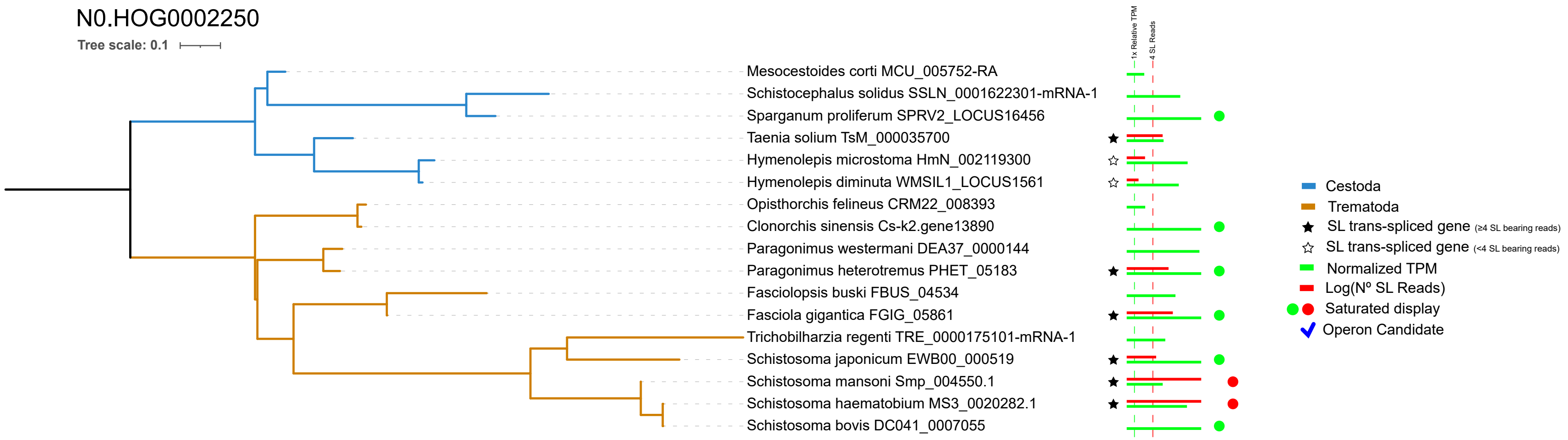

N0.HOG0002250
Tree scale: 0.1
1x Relative TPM
4 SL Reads
Mesocestoides corti MCU_005752-RA
Schistocephalus solidus SSLN_0001622301-mRNA-1
Sparganum proliferum SPRV2_LOCUS16456
Taenia solium TsM_000035700
Hymenolepis microstoma HmN_002119300
Hymenolepis diminuta WMSIL1_LOCUS1561
Opisthorchis felineus CRM22_008393
Clonorchis sinensis Cs-k2.gene13890
Paragonimus westermani DEA37_0000144
Paragonimus heterotremus PHET_05183
Fasciolopsis buski FBUS_04534
Fasciola gigantica FGIG_05861
Trichobilharzia regenti TRE_0000175101-mRNA-1
Schistosoma japonicum EWB00_000519
Schistosoma mansoni Smp_004550.1
Schistosoma haematobium MS3_0020282.1
Schistosoma bovis DC041_0007055
Cestoda
Trematoda
SL trans-spliced gene (≥4 SL bearing reads)
SL trans-spliced gene (<4 SL bearing reads)
Normalized TPM
Log(Nº SL Reads)
Saturated display
Operon Candidate

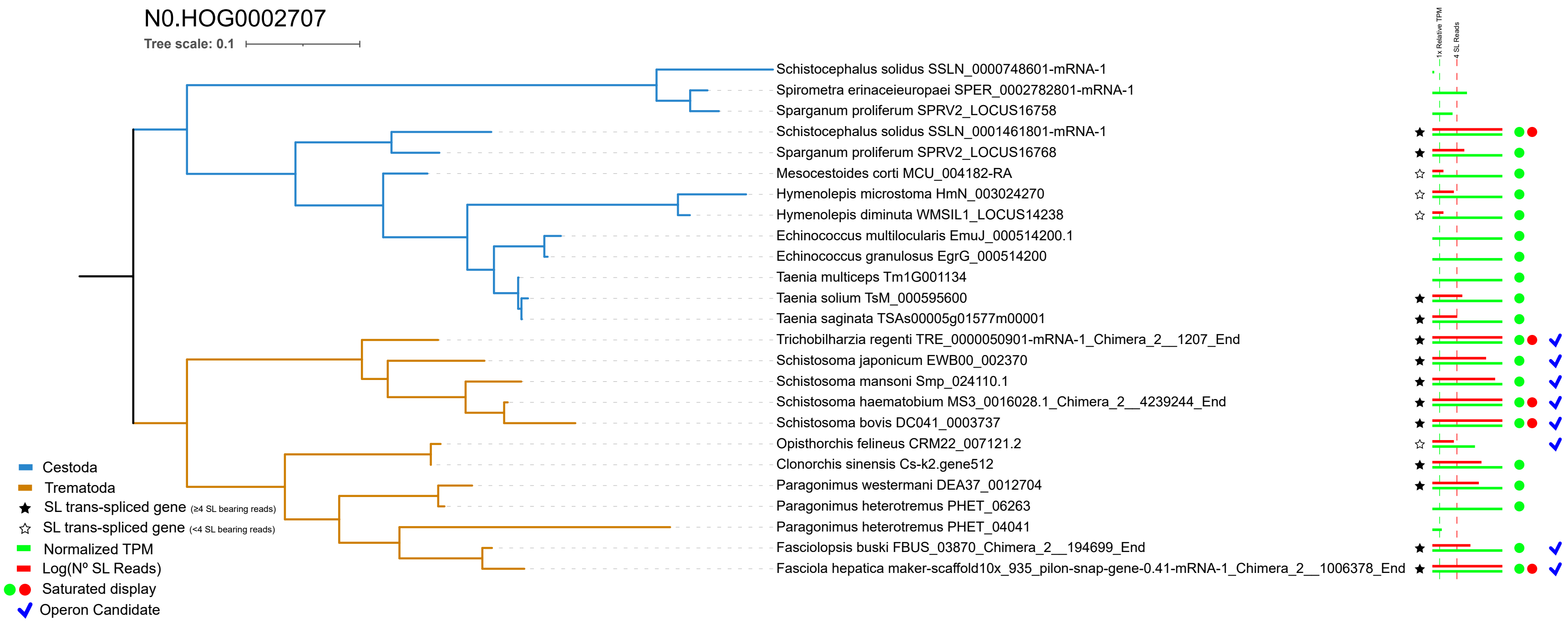
N0.HOG0002707
Tree scale: 0.1
Schistocephalus solidus SSLN_0000748601-mRNA-1
Spirometra erinaceieuropaei SPER_0002782801-mRNA-1
Sparganum proliferum SPRV2_LOCUS16758
Schistocephalus solidus SSLN_0001461801-mRNA-1
Sparganum proliferum SPRV2_LOCUS16768
Mesocestoides corti MCU_004182-RA
Hymenolepis microstoma HmN_003024270
Hymenolepis diminuta WMSIL1_LOCUS14238
Echinococcus multilocularis EmuJ_000514200.1
Echinococcus granulosus EgrG_000514200
Taenia multiceps Tm1G001134
Taenia solium TsM_000595600
Taenia saginata TSAs00005g01577m00001
Trichobilharzia regenti TRE_0000050901-mRNA-1_Chimera_2__1207_End
Schistosoma japonicum EWB00_002370
Schistosoma mansoni Smp_024110.1
Schistosoma haematobium MS3_0016028.1_Chimera_2__4239244_End
Schistosoma bovis DC041_0003737
Opisthorchis felineus CRM22_007121.2
Clonorchis sinensis Cs-k2.gene512
Paragonimus westermani DEA37_0012704
Paragonimus heterotremus PHET_06263
Paragonimus heterotremus PHET_04041
Fasciolopsis buski FBUS_03870_Chimera_2__194699_End
Fasciola hepatica maker-scaffold10x_935_pilon-snap-gene-0.41-mRNA-1_Chimera_2__1006378_End
1x Relative TPM
4 SL Reads
Cestoda
Trematoda
SL trans-spliced gene (≥4 SL bearing reads)
SL trans-spliced gene (<4 SL bearing reads)
Normalized TPM
Log(Nº SL Reads)
Saturated display
Operon Candidate

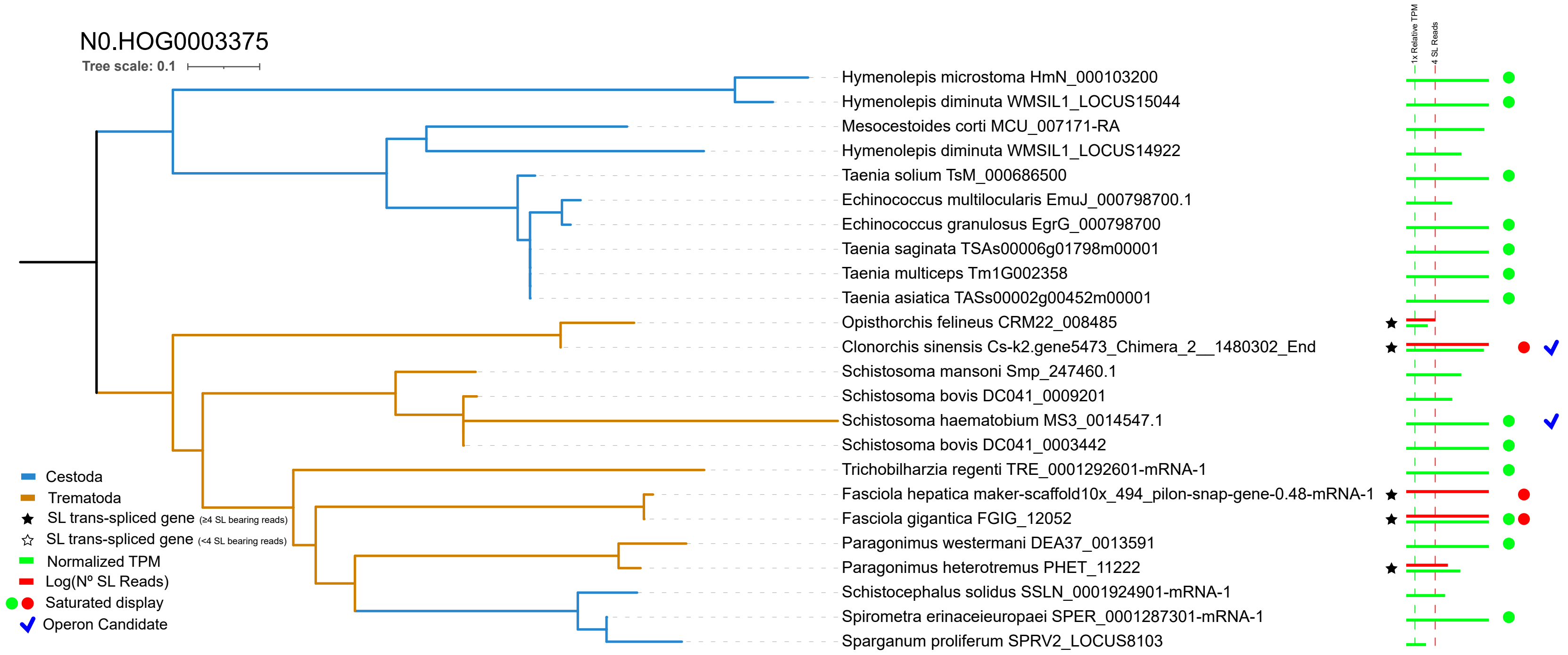

N0.HOG0003375
Tree scale: 0.1
1x Relative TPM
4 SL Reads
Hymenolepis microstoma HmN_000103200
Hymenolepis diminuta WMSIL1_LOCUS15044
Mesocestoides corti MCU_007171-RA
Hymenolepis diminuta WMSIL1_LOCUS14922
Taenia solium TsM_000686500
Echinococcus multilocularis EmuJ_000798700.1
Echinococcus granulosus EgrG_000798700
Taenia saginata TSAs00006g01798m00001
Taenia multiceps Tm1G002358
Taenia asiatica TASs00002g00452m00001
Opisthorchis felineus CRM22_008485
Clonorchis sinensis Cs-k2.gene5473_Chimera_2__1480302_End
Schistosoma mansoni Smp_247460.1
Schistosoma bovis DC041_0009201
Schistosoma haematobium MS3_0014547.1
Schistosoma bovis DC041_0003442
Trichobilharzia regenti TRE_0001292601-mRNA-1
Fasciola hepatica maker-scaffold10x_494_pilon-snap-gene-0.48-mRNA-1
Fasciola gigantica FGIG_12052
Paragonimus westermani DEA37_0013591
Paragonimus heterotremus PHET_11222
Schistocephalus solidus SSLN_0001924901-mRNA-1
Spirometra erinaceieuropaei SPER_0001287301-mRNA-1
Sparganum proliferum SPRV2_LOCUS8103
Cestoda
Trematoda
SL trans-spliced gene (≥4 SL bearing reads)
SL trans-spliced gene (<4 SL bearing reads)
Normalized TPM
Log(Nº SL Reads)
Saturated display
Operon Candidate

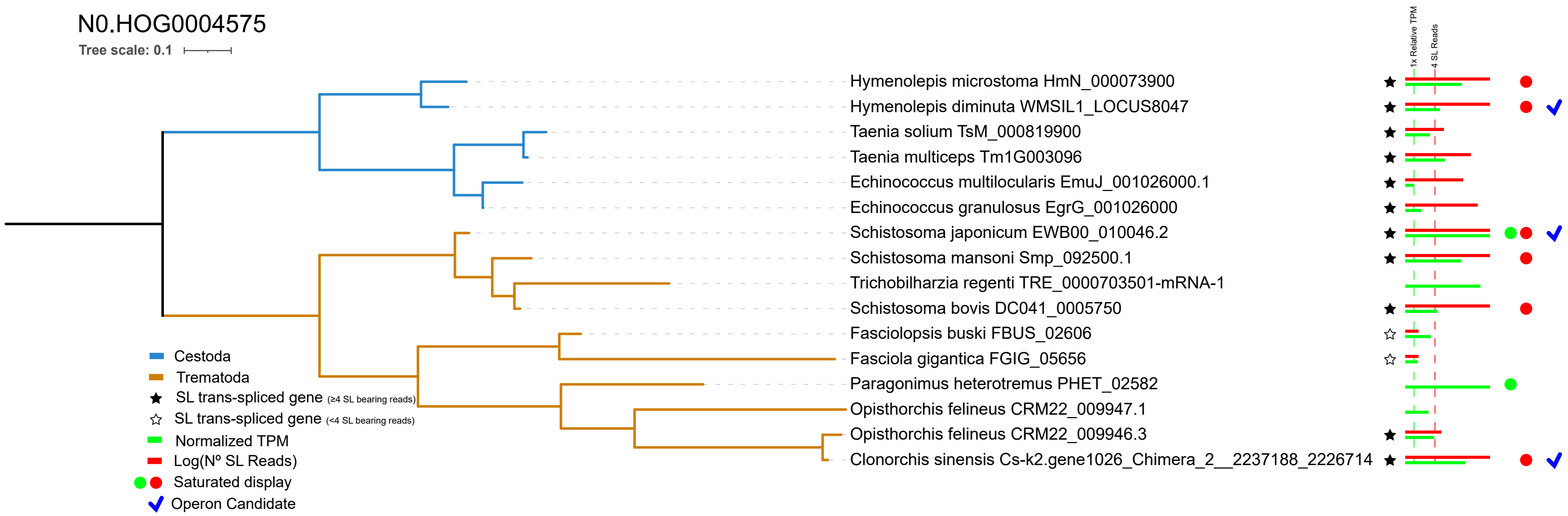
N0.HOG0004575
Tree scale: 0.1
1x Relative TPM
4 SL Reads
Hymenolepis microstoma HmN_000073900
Hymenolepis diminuta WMSIL1_LOCUS8047
Taenia solium TsM_000819900
Taenia multiceps Tm1G003096
Echinococcus multilocularis EmuJ_001026000.1
Echinococcus granulosus EgrG_001026000
Schistosoma japonicum EWB00_010046.2
Schistosoma mansoni Smp_092500.1
Trichobilharzia regenti TRE_0000703501-mRNA-1
Schistosoma bovis DC041_0005750
Fasciolopsis buski FBUS_02606
Fasciola gigantica FGIG_05656
Paragonimus heterotremus PHET_02582
Opisthorchis felineus CRM22_009947.1
Opisthorchis felineus CRM22_009946.3
Clonorchis sinensis Cs-k2.gene1026_Chimera_2__2237188_2226714
Cestoda
Trematoda
SL trans-spliced gene (≥4 SL bearing reads)
SL trans-spliced gene (<4 SL bearing reads)
Normalized TPM
Log(Nº SL Reads)
Saturated display
Operon Candidate

N0.HOG0005209

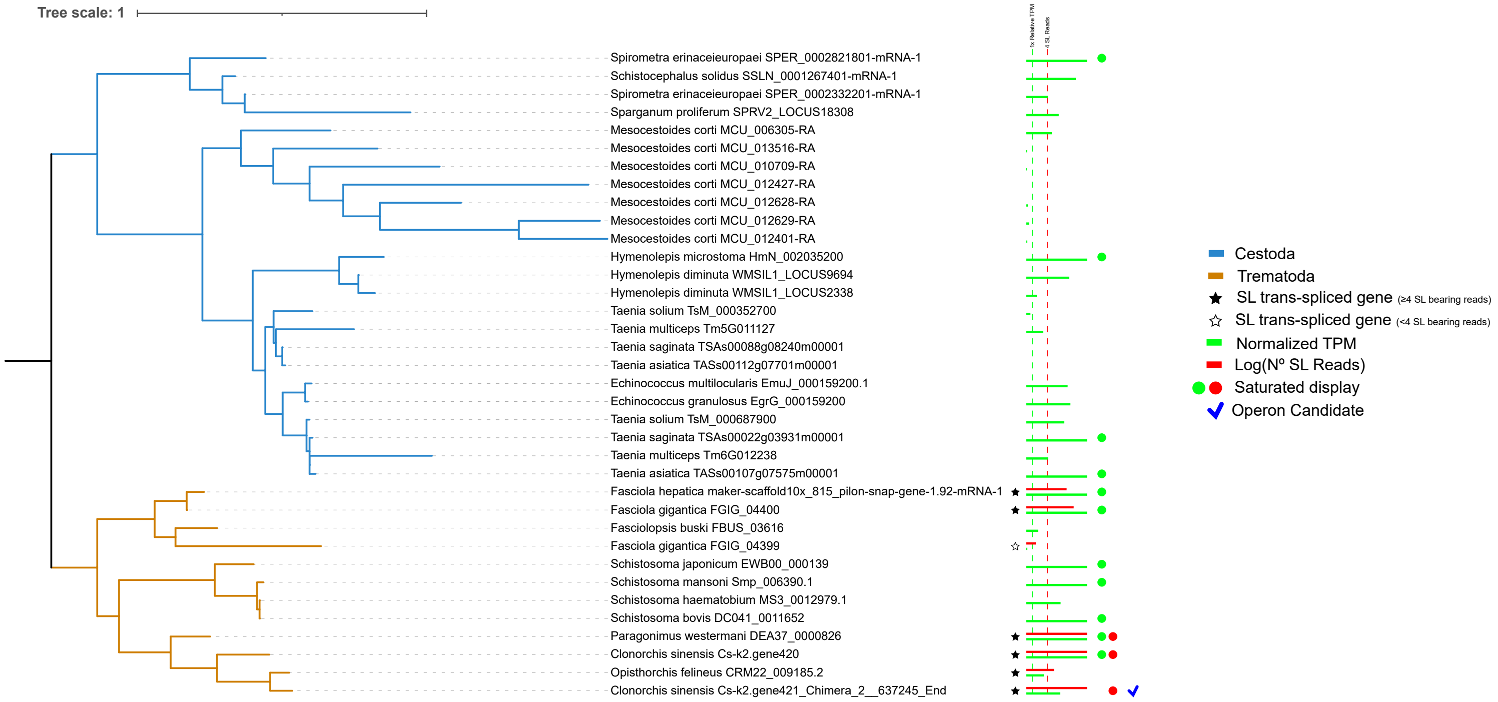

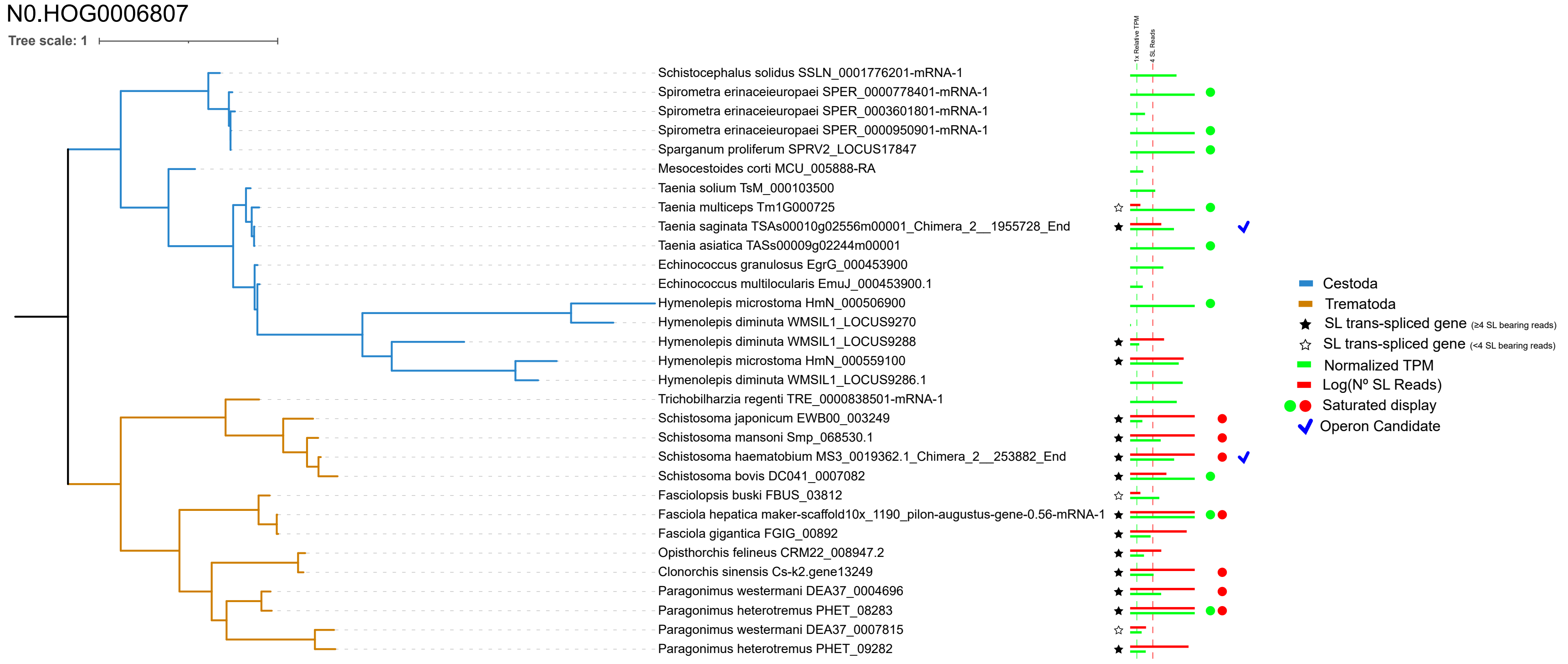
N0.HOG0006807
Tree scale: 1
1x Relative TPM
4 SL Reads
Schistocephalus solidus SSLN_0001776201-mRNA-1
Spirometra erinaceieuropaei SPER_0000778401-mRNA-1
Spirometra erinaceieuropaei SPER_0003601801-mRNA-1
Spirometra erinaceieuropaei SPER_0000950901-mRNA-1
Sparganum proliferum SPRV2_LOCUS17847
Mesocestoides corti MCU_005888-RA
Taenia solium TsM_000103500
Taenia multiceps Tm1G000725
Taenia saginata TSAs00010g02556m00001_Chimera_2__1955728_End
Taenia asiatica TASs00009g02244m00001
Echinococcus granulosus EgrG_000453900
Echinococcus multilocularis EmuJ_000453900.1
Hymenolepis microstoma HmN_000506900
Hymenolepis diminuta WMSIL1_LOCUS9270
Hymenolepis diminuta WMSIL1_LOCUS9288
Hymenolepis microstoma HmN_000559100
Hymenolepis diminuta WMSIL1_LOCUS9286.1
Trichobilharzia regenti TRE_0000838501-mRNA-1
Schistosoma japonicum EWB00_003249
Schistosoma mansoni Smp_068530.1
Schistosoma haematobium MS3_0019362.1_Chimera_2__253882_End
Schistosoma bovis DC041_0007082
Fasciolopsis buski FBUS_03812
Fasciola hepatica maker-scaffold10x_1190_pilon-augustus-gene-0.56-mRNA-1
Fasciola gigantica FGIG_00892
Opisthorchis felineus CRM22_008947.2
Clonorchis sinensis Cs-k2.gene13249
Paragonimus westermani DEA37_0004696
Paragonimus heterotremus PHET_08283
Paragonimus westermani DEA37_0007815
Paragonimus heterotremus PHET_09282
Cestoda
Trematoda
SL trans-spliced gene (≥4 SL bearing reads)
SL trans-spliced gene (<4 SL bearing reads)
Normalized TPM
Log(Nº SL Reads)
Saturated display
Operon Candidate

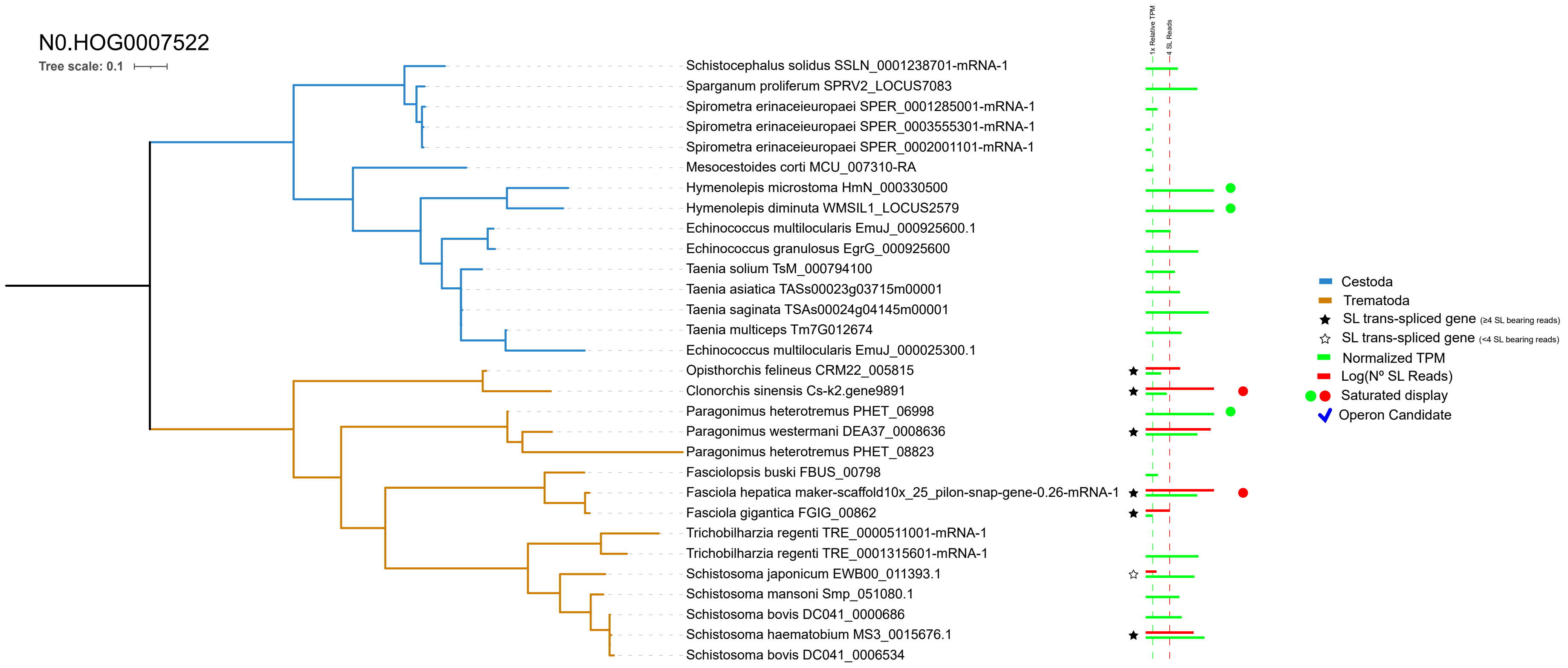

N0.HOG0007522
Tree scale: 0.1
1x Relative TPM
4 SL Reads
Schistocephalus solidus SSLN_0001238701-mRNA-1
Sparganum proliferum SPRV2_LOCUS7083
Spirometra erinaceieuropaei SPER_0001285001-mRNA-1
Spirometra erinaceieuropaei SPER_0003555301-mRNA-1
Spirometra erinaceieuropaei SPER_0002001101-mRNA-1
Mesocestoides corti MCU_007310-RA
Hymenolepis microstoma HmN_000330500
Hymenolepis diminuta WMSIL1_LOCUS2579
Echinococcus multilocularis EmuJ_000925600.1
Echinococcus granulosus EgrG_000925600
Taenia solium TsM_000794100
Taenia asiatica TASs00023g03715m00001
Taenia saginata TSAs00024g04145m00001
Taenia multiceps Tm7G012674
Echinococcus multilocularis EmuJ_000025300.1
Opisthorchis felineus CRM22_005815
Clonorchis sinensis Cs-k2.gene9891
Paragonimus heterotremus PHET_06998
Paragonimus westermani DEA37_0008636
Paragonimus heterotremus PHET_08823
Fasciolopsis buski FBUS_00798
Fasciola hepatica maker-scaffold10x_25_pilon-snap-gene-0.26-mRNA-1
Fasciola gigantica FGIG_00862
Trichobilharzia regenti TRE_0000511001-mRNA-1
Trichobilharzia regenti TRE_0001315601-mRNA-1
Schistosoma japonicum EWB00_011393.1
Schistosoma mansoni Smp_051080.1
Schistosoma bovis DC041_0000686
Schistosoma haematobium MS3_0015676.1
Schistosoma bovis DC041_0006534
Cestoda
Trematoda
SL trans-spliced gene (≥4 SL bearing reads)
SL trans-spliced gene (<4 SL bearing reads)
Normalized TPM
Log(Nº SL Reads)
Saturated display
Operon Candidate

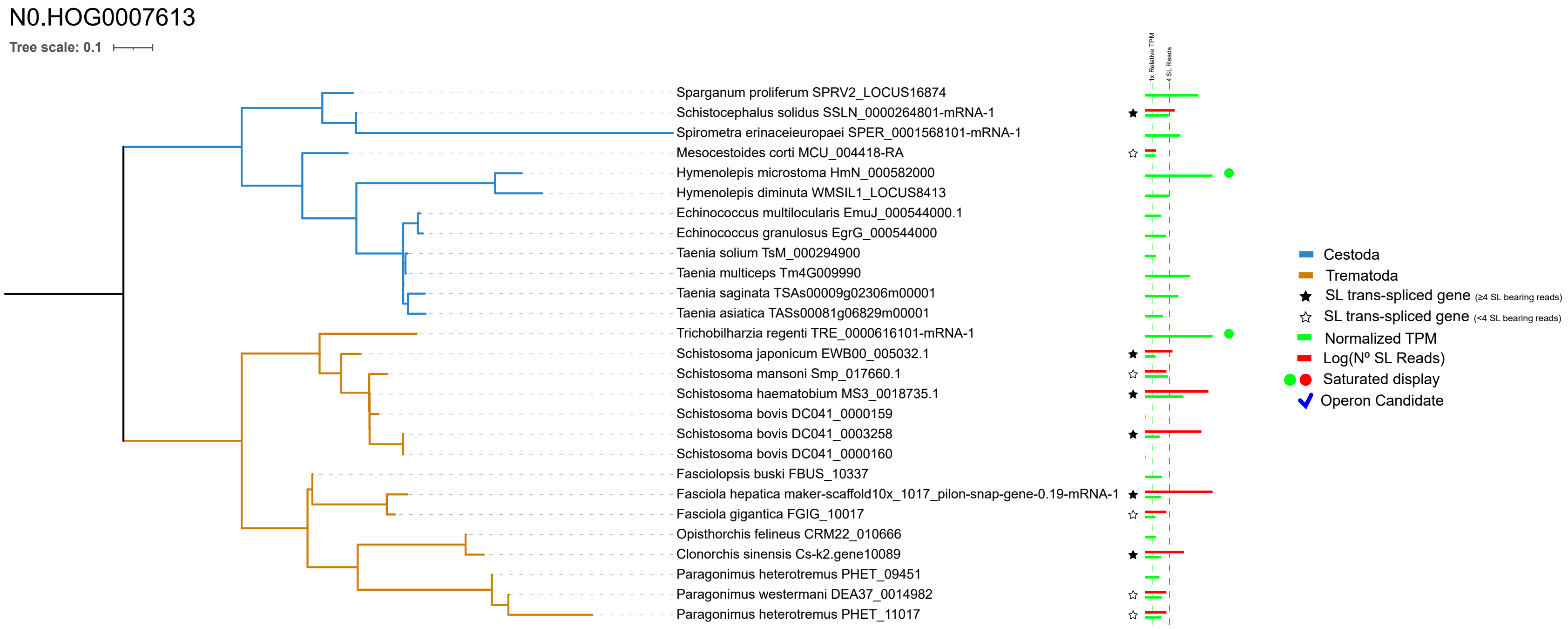

N0.HOG0007613
Tree scale: 0.1
1x Relative TPM
4 SL Reads
Sparganum proliferum SPRV2_LOCUS16874
Schistocephalus solidus SSLN_0000264801-mRNA-1
Spirometra erinaceieuropaei SPER_0001568101-mRNA-1
Mesocestoides corti MCU_004418-RA
Hymenolepis microstoma HmN_000582000
Hymenolepis diminuta WMSIL1_LOCUS8413
Echinococcus multilocularis EmuJ_000544000.1
Echinococcus granulosus EgrG_000544000
Taenia solium TsM_000294900
Taenia multiceps Tm4G009990
Taenia saginata TSAs00009g02306m00001
Taenia asiatica TASs00081g06829m00001
Trichobilharzia regenti TRE_0000616101-mRNA-1
Schistosoma japonicum EWB00_005032.1
Schistosoma mansoni Smp_017660.1
Schistosoma haematobium MS3_0018735.1
Schistosoma bovis DC041_0000159
Schistosoma bovis DC041_0003258
Schistosoma bovis DC041_0000160
Fasciolopsis buski FBUS_10337
Fasciola hepatica maker-scaffold10x_1017_pilon-snap-gene-0.19-mRNA-1
Fasciola gigantica FGIG_10017
Opisthorchis felineus CRM22_010666
Clonorchis sinensis Cs-k2.gene10089
Paragonimus heterotremus PHET_09451
Paragonimus westermani DEA37_0014982
Paragonimus heterotremus PHET_11017
Cestoda
Trematoda
SL trans-spliced gene (≥4 SL bearing reads)
SL trans-spliced gene (<4 SL bearing reads)
Normalized TPM
Log(Nº SL Reads)
Saturated display
Operon Candidate

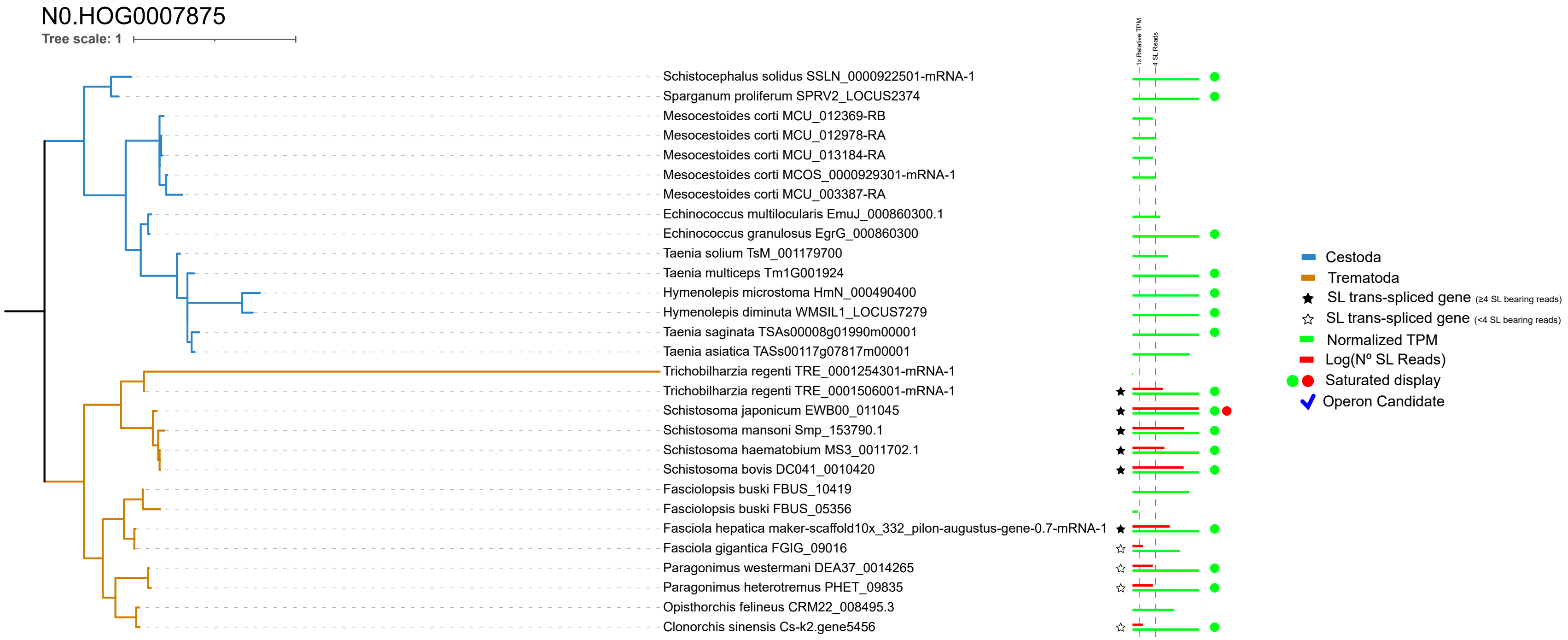
N0.HOG0007875
Tree scale: 1
1x Relative TPM
4 SL Reads
Schistocephalus solidus SSLN_0000922501-mRNA-1
Sparganum proliferum SPRV2_LOCUS2374
Mesocestoides corti MCU_012369-RB
Mesocestoides corti MCU_012978-RA
Mesocestoides corti MCU_013184-RA
Mesocestoides corti MCOS_0000929301-mRNA-1
Mesocestoides corti MCU_003387-RA
Echinococcus multilocularis EmuJ_000860300.1
Echinococcus granulosus EgrG_000860300
Taenia solium TsM_001179700
Taenia multiceps Tm1G001924
Hymenolepis microstoma HmN_000490400
Hymenolepis diminuta WMSIL1_LOCUS7279
Taenia saginata TSAs00008g01990m00001
Taenia asiatica TASs00117g07817m00001
Trichobilharzia regenti TRE_0001254301-mRNA-1
Trichobilharzia regenti TRE_0001506001-mRNA-1
Schistosoma japonicum EWB00_011045
Schistosoma mansoni Smp_153790.1
Schistosoma haematobium MS3_0011702.1
Schistosoma bovis DC041_0010420
Fasciolopsis buski FBUS_10419
Fasciolopsis buski FBUS_05356
Fasciola hepatica maker-scaffold10x_332_pilon-augustus-gene-0.7-mRNA-1
Fasciola gigantica FGIG_09016
Paragonimus westermani DEA37_0014265
Paragonimus heterotremus PHET_09835
Opisthorchis felineus CRM22_008495.3
Clonorchis sinensis Cs-k2.gene5456
Cestoda
Trematoda
SL trans-spliced gene (≥4 SL bearing reads)
SL trans-spliced gene (<4 SL bearing reads)
Normalized TPM
Log(Nº SL Reads)
Saturated display
Operon Candidate

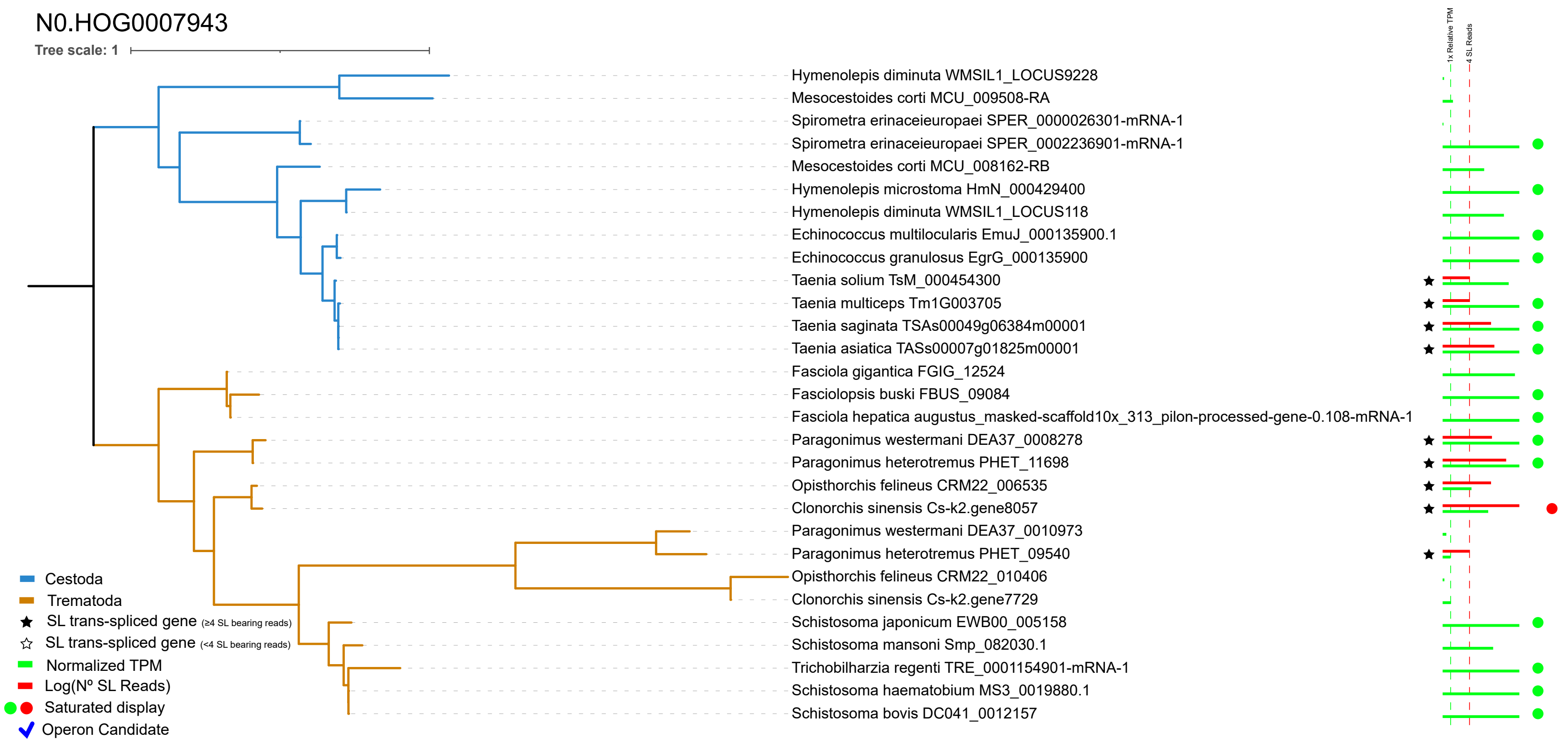

N0.HOG0007943
Tree scale: 1
1x Relative TPM
4 SL Reads
Hymenolepis diminuta WMSIL1_LOCUS9228
Mesocestoides corti MCU_009508-RA
Spirometra erinaceieuropaei SPER_0000026301-mRNA-1
Spirometra erinaceieuropaei SPER_0002236901-mRNA-1
Mesocestoides corti MCU_008162-RB
Hymenolepis microstoma HmN_000429400
Hymenolepis diminuta WMSIL1_LOCUS118
Echinococcus multilocularis EmuJ_000135900.1
Echinococcus granulosus EgrG_000135900
Taenia solium TsM_000454300
Taenia multiceps Tm1G003705
Taenia saginata TSAs00049g06384m00001
Taenia asiatica TASs00007g01825m00001
Fasciola gigantica FGIG_12524
Fasciolopsis buski FBUS_09084
Fasciola hepatica augustus_masked-scaffold10x_313_pilon-processed-gene-0.108-mRNA-1
Paragonimus westermani DEA37_0008278
Paragonimus heterotremus PHET_11698
Opisthorchis felineus CRM22_006535
Clonorchis sinensis Cs-k2.gene8057
Paragonimus westermani DEA37_0010973
Paragonimus heterotremus PHET_09540
Opisthorchis felineus CRM22_010406
Clonorchis sinensis Cs-k2.gene7729
Schistosoma japonicum EWB00_005158
Schistosoma mansoni Smp_082030.1
Trichobilharzia regenti TRE_0001154901-mRNA-1
Schistosoma haematobium MS3_0019880.1
Schistosoma bovis DC041_0012157
Cestoda
Trematoda
SL trans-spliced gene (≥4 SL bearing reads)
SL trans-spliced gene (<4 SL bearing reads)
Normalized TPM
Log(Nº SL Reads)
Saturated display
Operon Candidate

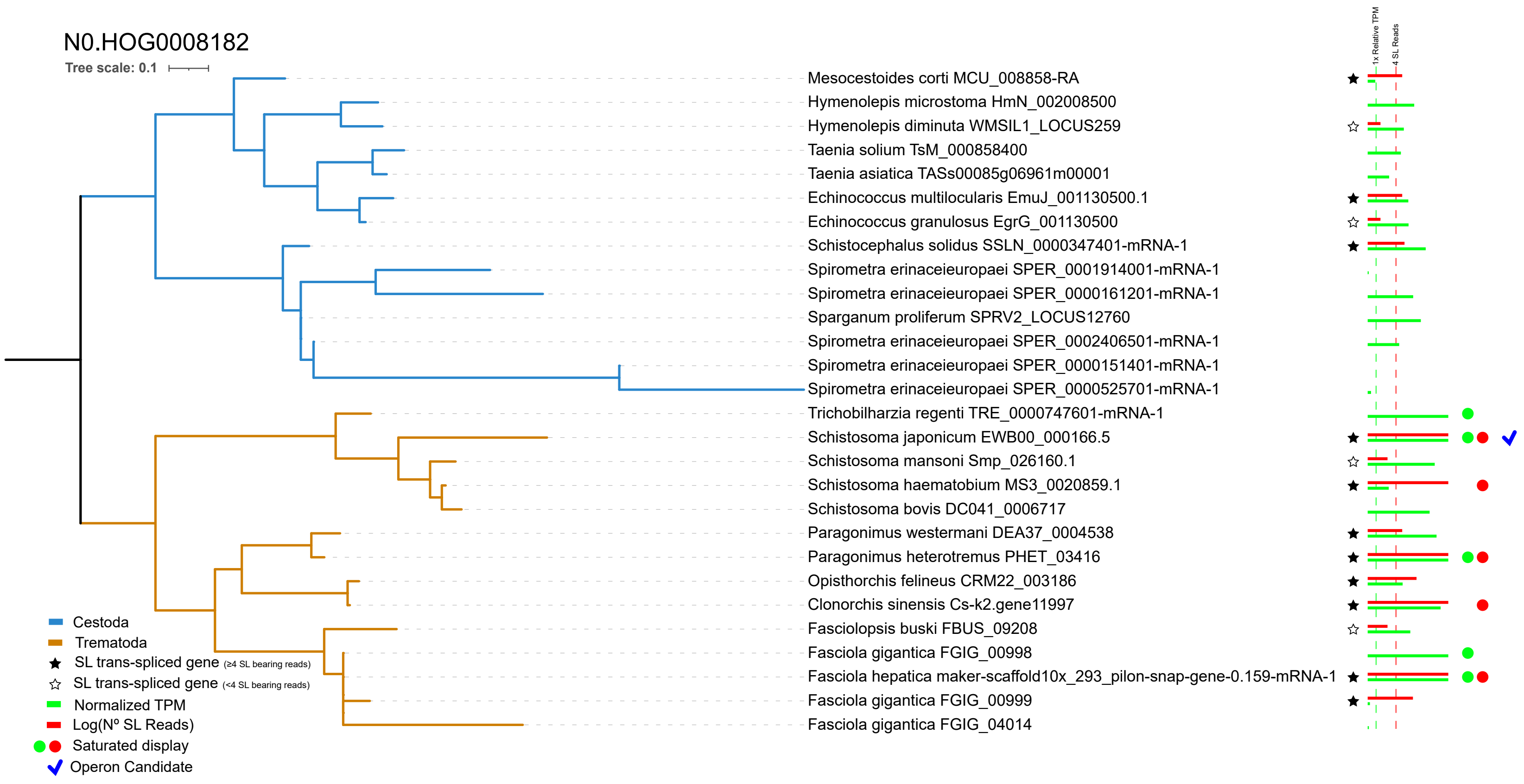

N0.HOG0008182
Tree scale: 0.1
1x Relative TPM
4 SL Reads
Mesocestoides corti MCU_008858-RA
Hymenolepis microstoma HmN_002008500
Hymenolepis diminuta WMSIL1_LOCUS259
Taenia solium TsM_000858400
Taenia asiatica TASs00085g06961m00001
Echinococcus multilocularis EmuJ_001130500.1
Echinococcus granulosus EgrG_001130500
Schistocephalus solidus SSLN_0000347401-mRNA-1
Spirometra erinaceieuropaei SPER_0001914001-mRNA-1
Spirometra erinaceieuropaei SPER_0000161201-mRNA-1
Sparganum proliferum SPRV2_LOCUS12760
Spirometra erinaceieuropaei SPER_0002406501-mRNA-1
Spirometra erinaceieuropaei SPER_0000151401-mRNA-1
Spirometra erinaceieuropaei SPER_0000525701-mRNA-1
Trichobilharzia regenti TRE_0000747601-mRNA-1
Schistosoma japonicum EWB00_000166.5
Schistosoma mansoni Smp_026160.1
Schistosoma haematobium MS3_0020859.1
Schistosoma bovis DC041_0006717
Paragonimus westermani DEA37_0004538
Paragonimus heterotremus PHET_03416
Opisthorchis felineus CRM22_003186
Clonorchis sinensis Cs-k2.gene11997
Fasciolopsis buski FBUS_09208
Fasciola gigantica FGIG_00998
Fasciola hepatica maker-scaffold10x_293_pilon-snap-gene-0.159-mRNA-1
Fasciola gigantica FGIG_00999
Fasciola gigantica FGIG_04014
Cestoda
Trematoda
SL trans-spliced gene (≥4 SL bearing reads)
SL trans-spliced gene (<4 SL bearing reads)
Normalized TPM
Log(Nº SL Reads)
Saturated display
Operon Candidate

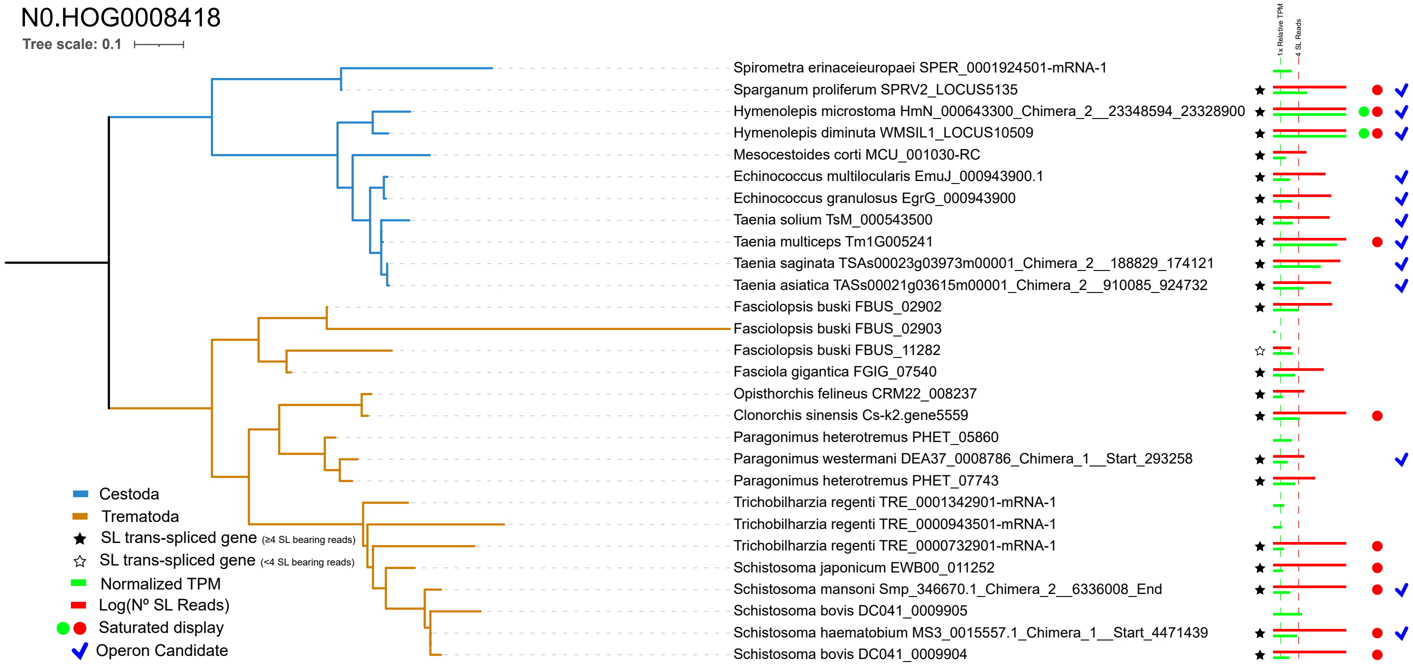
N0.HOG0008418
Tree scale: 0.1
1x Relative TPM
4 SL Reads
Spirometra erinaceieuropaei SPER_0001924501-mRNA-1
Sparganum proliferum SPRV2_LOCUS5135
Hymenolepis microstoma HmN_000643300_Chimera_2__23348594_23328900
Hymenolepis diminuta WMSIL1_LOCUS10509
Mesocestoides corti MCU_001030-RC
Echinococcus multilocularis EmuJ_000943900.1
Echinococcus granulosus EgrG_000943900
Taenia solium TsM_000543500
Taenia multiceps Tm1G005241
Taenia saginata TSAs00023g03973m00001_Chimera_2__188829_174121
Taenia asiatica TASs00021g03615m00001_Chimera_2__910085_924732
Fasciolopsis buski FBUS_02902
Fasciolopsis buski FBUS_02903
Fasciolopsis buski FBUS_11282
Fasciola gigantica FGIG_07540
Opisthorchis felineus CRM22_008237
Clonorchis sinensis Cs-k2.gene5559
Paragonimus heterotremus PHET_05860
Paragonimus westermani DEA37_0008786_Chimera_1__Start_293258
Paragonimus heterotremus PHET_07743
Trichobilharzia regenti TRE_0001342901-mRNA-1
Trichobilharzia regenti TRE_0000943501-mRNA-1
Trichobilharzia regenti TRE_0000732901-mRNA-1
Schistosoma japonicum EWB00_011252
Schistosoma mansoni Smp_346670.1_Chimera_2__6336008_End
Schistosoma bovis DC041_0009905
Schistosoma haematobium MS3_0015557.1_Chimera_1__Start_4471439
Schistosoma bovis DC041_0009904
Cestoda
Trematoda
SL trans-spliced gene (≥4 SL bearing reads)
SL trans-spliced gene (<4 SL bearing reads)
Normalized TPM
Log(Nº SL Reads)
Saturated display
Operon Candidate

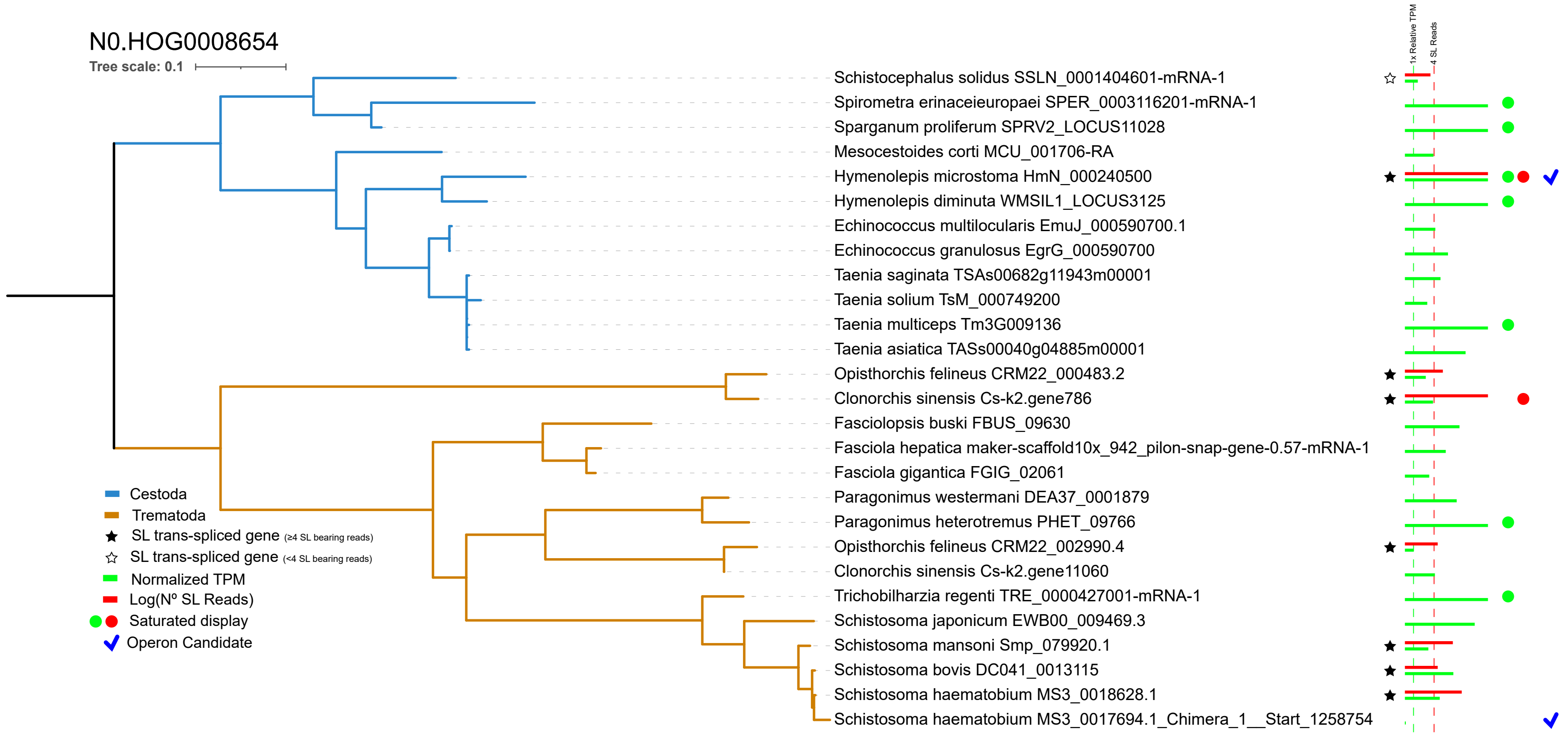

N0.HOG0008654
Tree scale: 0.1
1x Relative TPM
4 SL Reads
Schistocephalus solidus SSLN_0001404601-mRNA-1
Spirometra erinaceieuropaei SPER_0003116201-mRNA-1
Sparganum proliferum SPRV2_LOCUS11028
Mesocestoides corti MCU_001706-RA
Hymenolepis microstoma HmN_000240500
Hymenolepis diminuta WMSIL1_LOCUS3125
Echinococcus multilocularis EmuJ_000590700.1
Echinococcus granulosus EgrG_000590700
Taenia saginata TSAs00682g11943m00001
Taenia solium TsM_000749200
Taenia multiceps Tm3G009136
Taenia asiatica TASs00040g04885m00001
Opisthorchis felineus CRM22_000483.2
Clonorchis sinensis Cs-k2.gene786
Fasciolopsis buski FBUS_09630
Fasciola hepatica maker-scaffold10x_942_pilon-snap-gene-0.57-mRNA-1
Fasciola gigantica FGIG_02061
Paragonimus westermani DEA37_0001879
Paragonimus heterotremus PHET_09766
Opisthorchis felineus CRM22_002990.4
Clonorchis sinensis Cs-k2.gene11060
Trichobilharzia regenti TRE_0000427001-mRNA-1
Schistosoma japonicum EWB00_009469.3
Schistosoma mansoni Smp_079920.1
Schistosoma bovis DC041_0013115
Schistosoma haematobium MS3_0018628.1
Schistosoma haematobium MS3_0017694.1_Chimera_1__Start_1258754
Cestoda
Trematoda
SL trans-spliced gene (≥4 SL bearing reads)
SL trans-spliced gene (<4 SL bearing reads)
Normalized TPM
Log(Nº SL Reads)
Saturated display
Operon Candidate

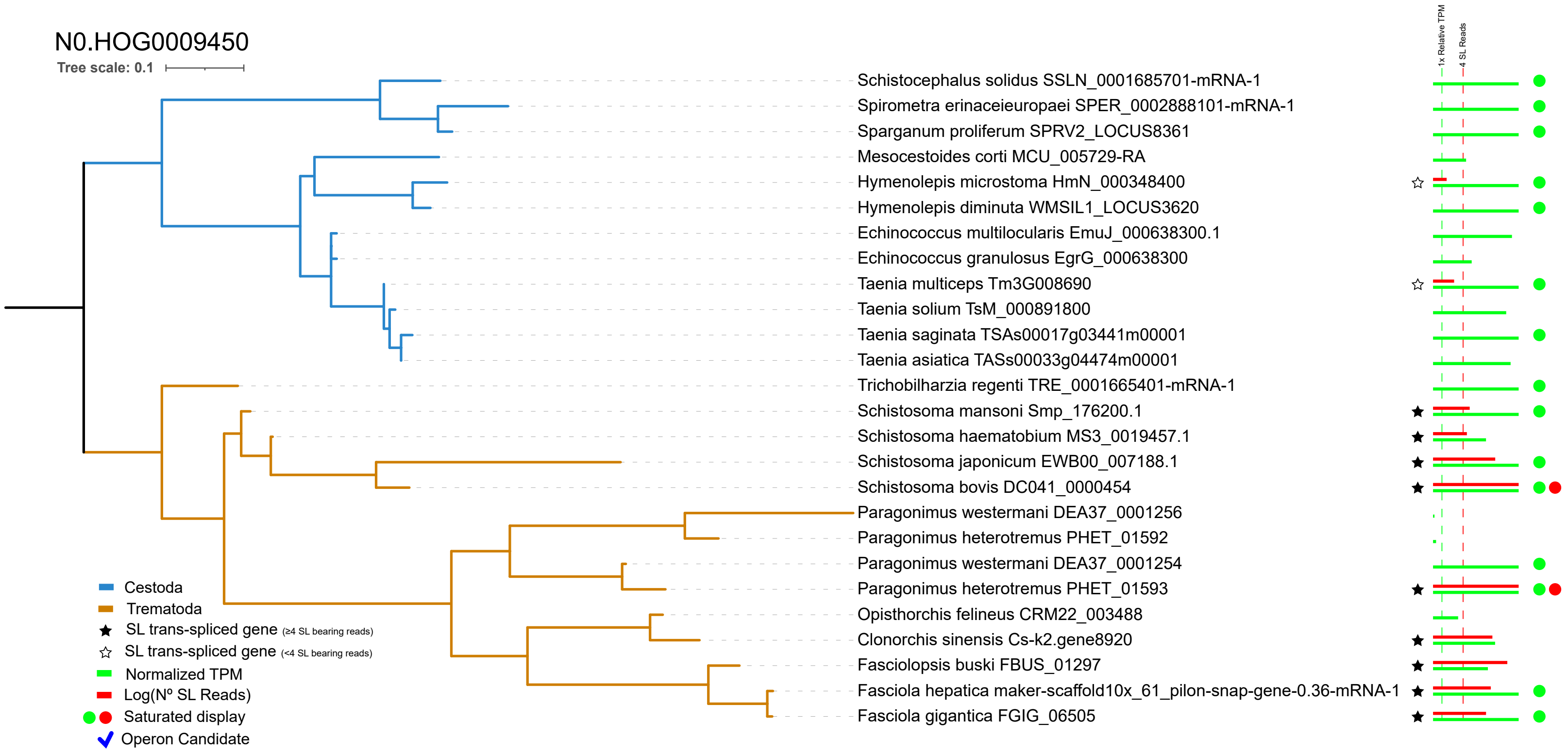
N0.HOG0009450
Tree scale: 0.1
1x Relative TPM
4 SL Reads
Schistocephalus solidus SSLN_0001685701-mRNA-1
Spirometra erinaceieuropaei SPER_0002888101-mRNA-1
Sparganum proliferum SPRV2_LOCUS8361
Mesocestoides corti MCU_005729-RA
Hymenolepis microstoma HmN_000348400
Hymenolepis diminuta WMSIL1_LOCUS3620
Echinococcus multilocularis EmuJ_000638300.1
Echinococcus granulosus EgrG_000638300
Taenia multiceps Tm3G008690
Taenia solium TsM_000891800
Taenia saginata TSAs00017g03441m00001
Taenia asiatica TASs00033g04474m00001
Trichobilharzia regenti TRE_0001665401-mRNA-1
Schistosoma mansoni Smp_176200.1
Schistosoma haematobium MS3_0019457.1
Schistosoma japonicum EWB00_007188.1
Schistosoma bovis DC041_0000454
Paragonimus westermani DEA37_0001256
Paragonimus heterotremus PHET_01592
Paragonimus westermani DEA37_0001254
Paragonimus heterotremus PHET_01593
Opisthorchis felineus CRM22_003488
Clonorchis sinensis Cs-k2.gene8920
Fasciolopsis buski FBUS_01297
Fasciola hepatica maker-scaffold10x_61_pilon-snap-gene-0.36-mRNA-1
Fasciola gigantica FGIG_06505
Cestoda
Trematoda
SL trans-spliced gene (≥4 SL bearing reads)
SL trans-spliced gene (<4 SL bearing reads)
Normalized TPM
Log(Nº SL Reads)
Saturated display
Operon Candidate

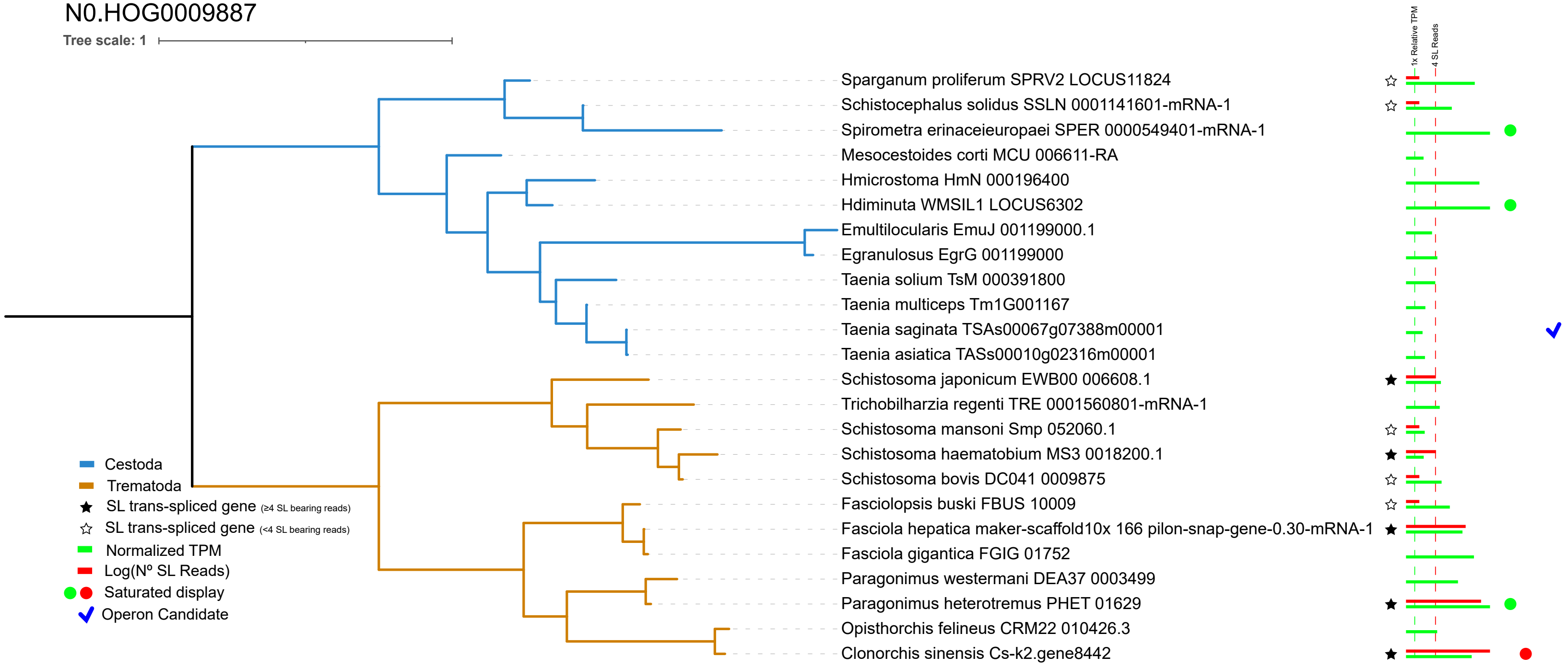

N0.HOG0009887
Tree scale: 1
1x Relative TPM
4 SL Reads
Sparganum proliferum SPRV2 LOCUS11824
Schistocephalus solidus SSLN 0001141601-mRNA-1
Spirometra erinaceieuropaei SPER 0000549401-mRNA-1
Mesocestoides corti MCU 006611-RA
Hmicrostoma HmN 000196400
Hdiminuta WMSIL1 LOCUS6302
Emultilocularis EmuJ 001199000.1
Egranulosus EgrG 001199000
Taenia solium TsM 000391800
Taenia multiceps Tm1G001167
Taenia saginata TSAs00067g07388m00001
Taenia asiatica TASs00010g02316m00001
Schistosoma japonicum EWB00 006608.1
Trichobilharzia regenti TRE 0001560801-mRNA-1
Schistosoma mansoni Smp 052060.1
Schistosoma haematobium MS3 0018200.1
Schistosoma bovis DC041 0009875
Fasciolopsis buski FBUS 10009
Fasciola hepatica maker-scaffold10x 166 pilon-snap-gene-0.30-mRNA-1
Fasciola gigantica FGIG 01752
Paragonimus westermani DEA37 0003499
Paragonimus heterotremus PHET 01629
Opisthorchis felineus CRM22 010426.3
Clonorchis sinensis Cs-k2.gene8442
Cestoda
Trematoda
SL trans-spliced gene (≥4 SL bearing reads)
SL trans-spliced gene (<4 SL bearing reads)
Normalized TPM
Log(Nº SL Reads)
Saturated display
Operon Candidate

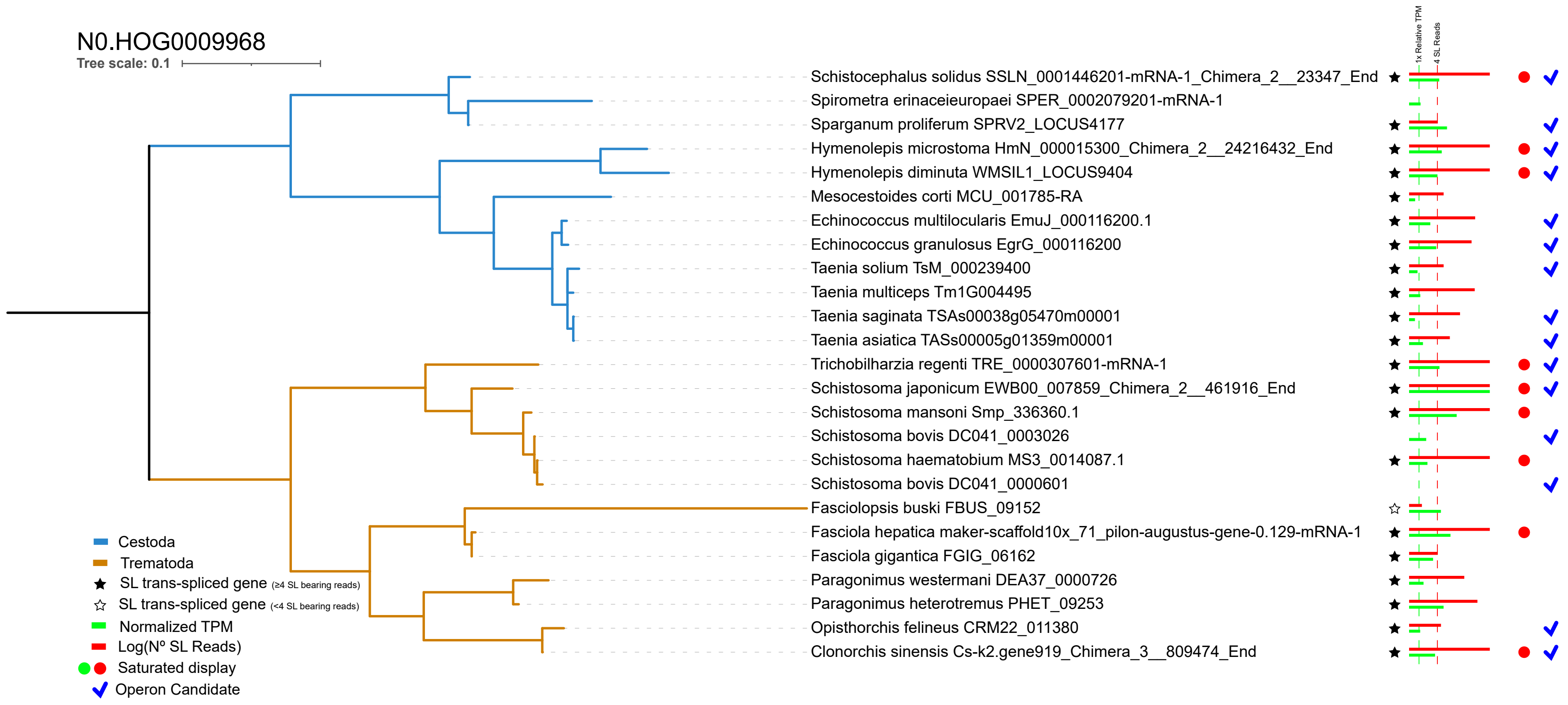

N0.HOG0009968
Tree scale: 0.1
1x Relative TPM
4 SL Reads
Schistocephalus solidus SSLN_0001446201-mRNA-1_Chimera_2__23347_End
Spirometra erinaceieuropaei SPER_0002079201-mRNA-1
Sparganum proliferum SPRV2_LOCUS4177
Hymenolepis microstoma HmN_000015300_Chimera_2__24216432_End
Hymenolepis diminuta WMSIL1_LOCUS9404
Mesocestoides corti MCU_001785-RA
Echinococcus multilocularis EmuJ_000116200.1
Echinococcus granulosus EgrG_000116200
Taenia solium TsM_000239400
Taenia multiceps Tm1G004495
Taenia saginata TSAs00038g05470m00001
Taenia asiatica TASs00005g01359m00001
Trichobilharzia regenti TRE_0000307601-mRNA-1
Schistosoma japonicum EWB00_007859_Chimera_2__461916_End
Schistosoma mansoni Smp_336360.1
Schistosoma bovis DC041_0003026
Schistosoma haematobium MS3_0014087.1
Schistosoma bovis DC041_0000601
Fasciolopsis buski FBUS_09152
Fasciola hepatica maker-scaffold10x_71_pilon-augustus-gene-0.129-mRNA-1
Fasciola gigantica FGIG_06162
Paragonimus westermani DEA37_0000726
Paragonimus heterotremus PHET_09253
Opisthorchis felineus CRM22_011380
Clonorchis sinensis Cs-k2.gene919_Chimera_3__809474_End
Cestoda
Trematoda
SL trans-spliced gene (≥4 SL bearing reads)
SL trans-spliced gene (<4 SL bearing reads)
Normalized TPM
Log(Nº SL Reads)
Saturated display
Operon Candidate

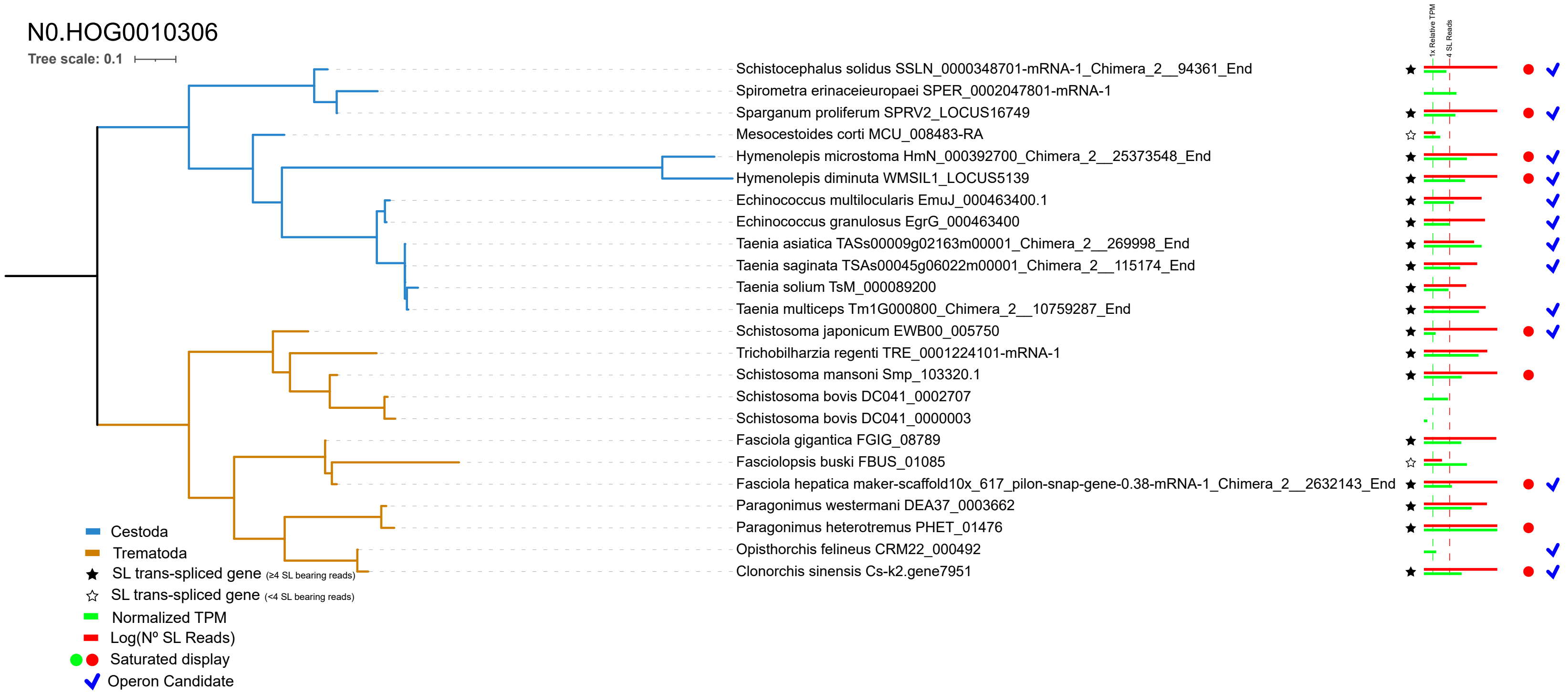

N0.HOG0010306
Tree scale: 0.1
1x Relative TPM
4 SL Reads
Schistocephalus solidus SSLN_0000348701-mRNA-1_Chimera_2__94361_End
Spirometra erinaceieuropaei SPER_0002047801-mRNA-1
Sparganum proliferum SPRV2_LOCUS16749
Mesocestoides corti MCU_008483-RA
Hymenolepis microstoma HmN_000392700_Chimera_2__25373548_End
Hymenolepis diminuta WMSIL1_LOCUS5139
Echinococcus multilocularis EmuJ_000463400.1
Echinococcus granulosus EgrG_000463400
Taenia asiatica TASs00009g02163m00001_Chimera_2__269998_End
Taenia saginata TSAs00045g06022m00001_Chimera_2__115174_End
Taenia solium TsM_000089200
Taenia multiceps Tm1G000800_Chimera_2__10759287_End
Schistosoma japonicum EWB00_005750
Trichobilharzia regenti TRE_0001224101-mRNA-1
Schistosoma mansoni Smp_103320.1
Schistosoma bovis DC041_0002707
Schistosoma bovis DC041_0000003
Fasciola gigantica FGIG_08789
Fasciolopsis buski FBUS_01085
Fasciola hepatica maker-scaffold10x_617_pilon-snap-gene-0.38-mRNA-1_Chimera_2__2632143_End
Paragonimus westermani DEA37_0003662
Paragonimus heterotremus PHET_01476
Opisthorchis felineus CRM22_000492
Clonorchis sinensis Cs-k2.gene7951
Cestoda
Trematoda
SL trans-spliced gene (≥4 SL bearing reads)
SL trans-spliced gene (<4 SL bearing reads)
Normalized TPM
Log(Nº SL Reads)
Saturated display
Operon Candidate

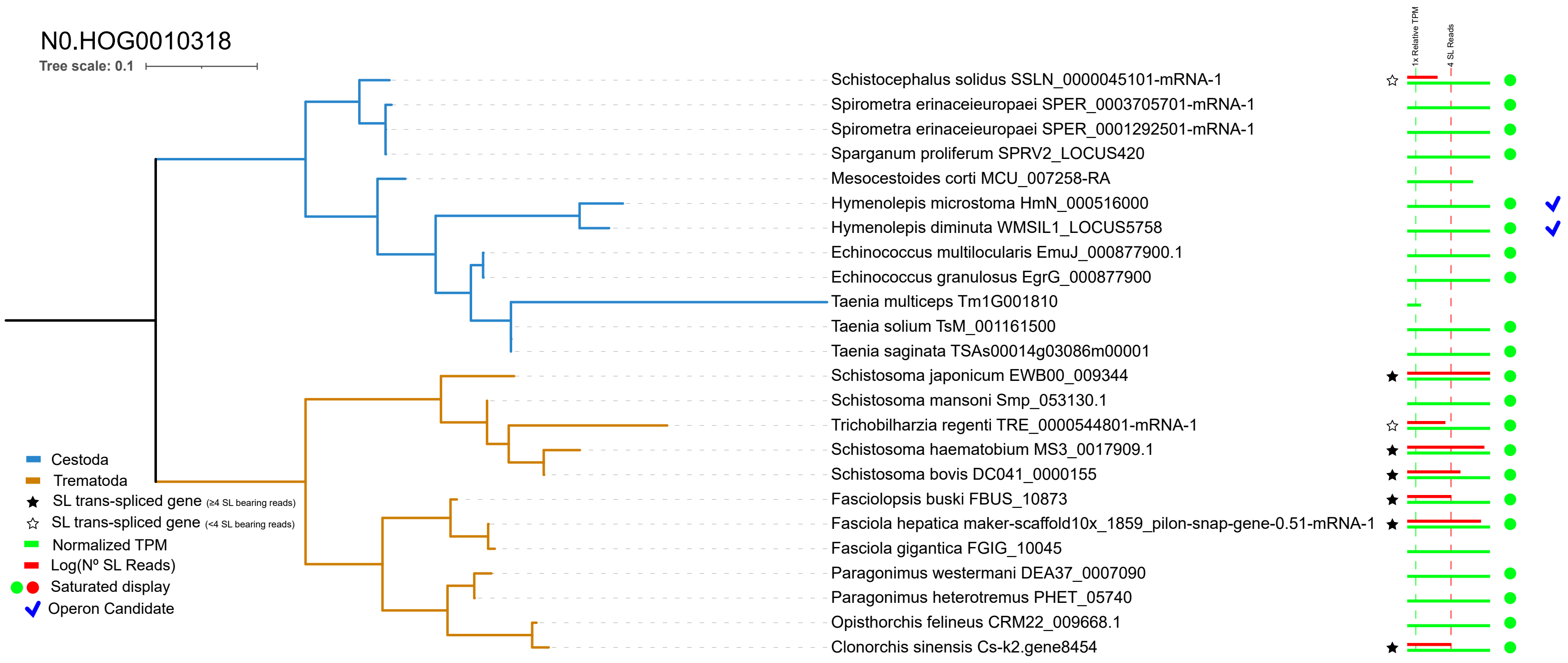

N0.HOG0010318
Tree scale: 0.1
1x Relative TPM
4 SL Reads
Schistocephalus solidus SSLN_0000045101-mRNA-1
Spirometra erinaceieuropaei SPER_0003705701-mRNA-1
Spirometra erinaceieuropaei SPER_0001292501-mRNA-1
Sparganum proliferum SPRV2_LOCUS420
Mesocestoides corti MCU_007258-RA
Hymenolepis microstoma HmN_000516000
Hymenolepis diminuta WMSIL1_LOCUS5758
Echinococcus multilocularis EmuJ_000877900.1
Echinococcus granulosus EgrG_000877900
Taenia multiceps Tm1G001810
Taenia solium TsM_001161500
Taenia saginata TSAs00014g03086m00001
Schistosoma japonicum EWB00_009344
Schistosoma mansoni Smp_053130.1
Trichobilharzia regenti TRE_0000544801-mRNA-1
Schistosoma haematobium MS3_0017909.1
Schistosoma bovis DC041_0000155
Fasciolopsis buski FBUS_10873
Fasciola hepatica maker-scaffold10x_1859_pilon-snap-gene-0.51-mRNA-1
Fasciola gigantica FGIG_10045
Paragonimus westermani DEA37_0007090
Paragonimus heterotremus PHET_05740
Opisthorchis felineus CRM22_009668.1
Clonorchis sinensis Cs-k2.gene8454
Cestoda
Trematoda
SL trans-spliced gene (≥4 SL bearing reads)
SL trans-spliced gene (<4 SL bearing reads)
Normalized TPM
Log(Nº SL Reads)
Saturated display
Operon Candidate

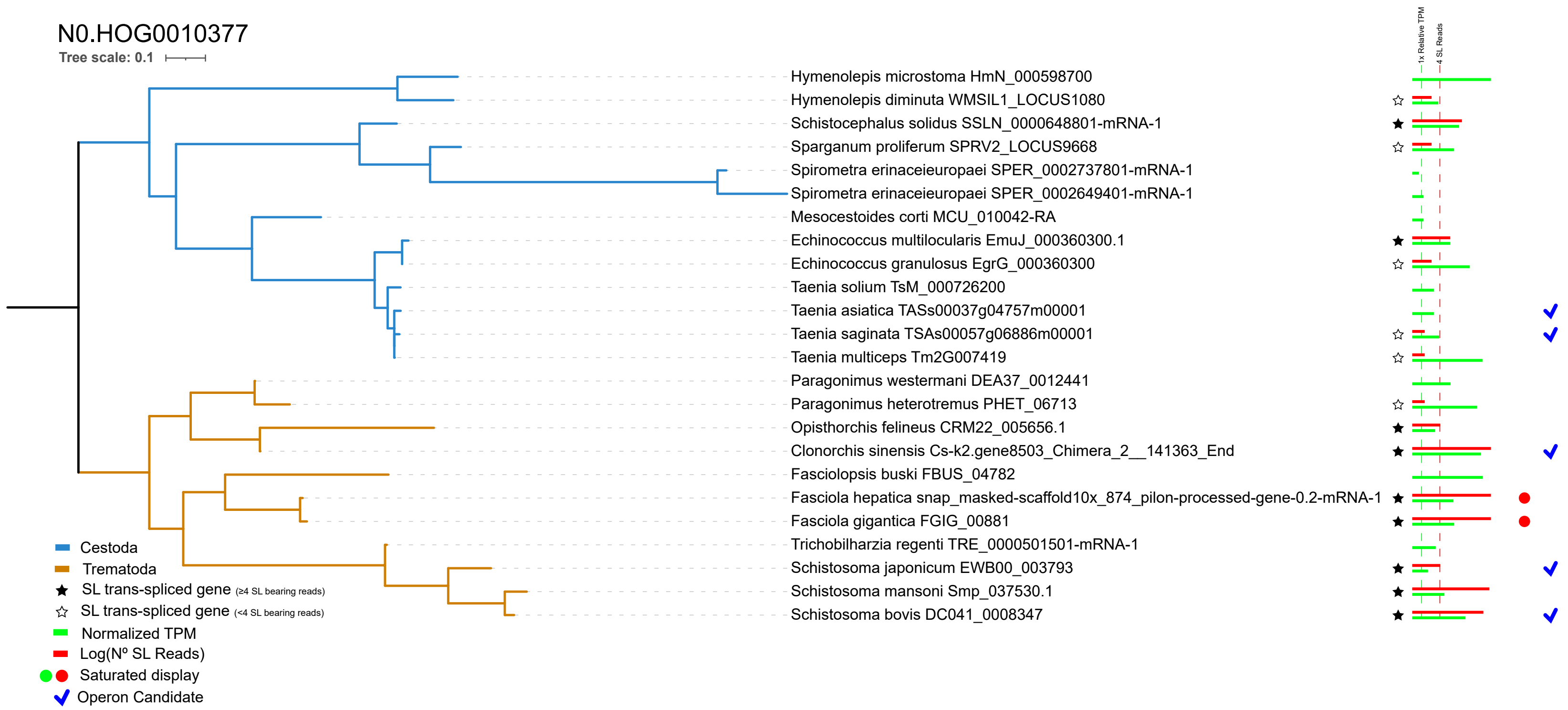

N0.HOG0010377
Tree scale: 0.1
1x Relative TPM
4 SL Reads
Hymenolepis microstoma HmN_000598700
Hymenolepis diminuta WMSIL1_LOCUS1080
Schistocephalus solidus SSLN_0000648801-mRNA-1
Sparganum proliferum SPRV2_LOCUS9668
Spirometra erinaceieuropaei SPER_0002737801-mRNA-1
Spirometra erinaceieuropaei SPER_0002649401-mRNA-1
Mesocestoides corti MCU_010042-RA
Echinococcus multilocularis EmuJ_000360300.1
Echinococcus granulosus EgrG_000360300
Taenia solium TsM_000726200
Taenia asiatica TASs00037g04757m00001
Taenia saginata TSAs00057g06886m00001
Taenia multiceps Tm2G007419
Paragonimus westermani DEA37_0012441
Paragonimus heterotremus PHET_06713
Opisthorchis felineus CRM22_005656.1
Clonorchis sinensis Cs-k2.gene8503_Chimera_2__141363_End
Fasciolopsis buski FBUS_04782
Fasciola hepatica snap_masked-scaffold10x_874_pilon-processed-gene-0.2-mRNA-1
Fasciola gigantica FGIG_00881
Trichobilharzia regenti TRE_0000501501-mRNA-1
Schistosoma japonicum EWB00_003793
Schistosoma mansoni Smp_037530.1
Schistosoma bovis DC041_0008347
Cestoda
Trematoda
SL trans-spliced gene (≥4 SL bearing reads)
SL trans-spliced gene (<4 SL bearing reads)
Normalized TPM
Log(Nº SL Reads)
Saturated display
Operon Candidate

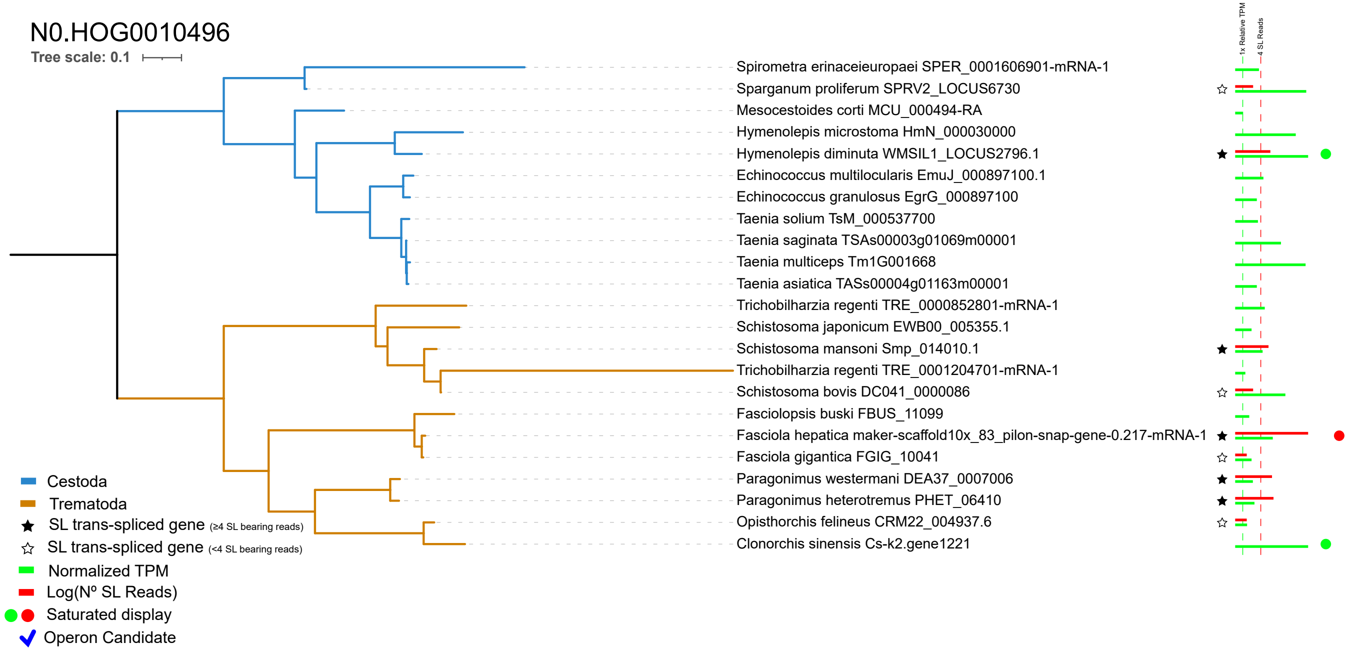
N0.HOG0010496
Tree scale: 0.1
1x Relative TPM
4 SL Reads
Spirometra erinaceieuropaei SPER_0001606901-mRNA-1
Sparganum proliferum SPRV2_LOCUS6730
Mesocestoides corti MCU_000494-RA
Hymenolepis microstoma HmN_000030000
Hymenolepis diminuta WMSIL1_LOCUS2796.1
Echinococcus multilocularis EmuJ_000897100.1
Echinococcus granulosus EgrG_000897100
Taenia solium TsM_000537700
Taenia saginata TSAs00003g01069m00001
Taenia multiceps Tm1G001668
Taenia asiatica TASs00004g01163m00001
Trichobilharzia regenti TRE_0000852801-mRNA-1
Schistosoma japonicum EWB00_005355.1
Schistosoma mansoni Smp_014010.1
Trichobilharzia regenti TRE_0001204701-mRNA-1
Schistosoma bovis DC041_0000086
Fasciolopsis buski FBUS_11099
Fasciola hepatica maker-scaffold10x_83_pilon-snap-gene-0.217-mRNA-1
Fasciola gigantica FGIG_10041
Paragonimus westermani DEA37_0007006
Paragonimus heterotremus PHET_06410
Opisthorchis felineus CRM22_004937.6
Clonorchis sinensis Cs-k2.gene1221
Cestoda
Trematoda
SL trans-spliced gene (≥4 SL bearing reads)
SL trans-spliced gene (<4 SL bearing reads)
Normalized TPM
Log(Nº SL Reads)
Saturated display
Operon Candidate

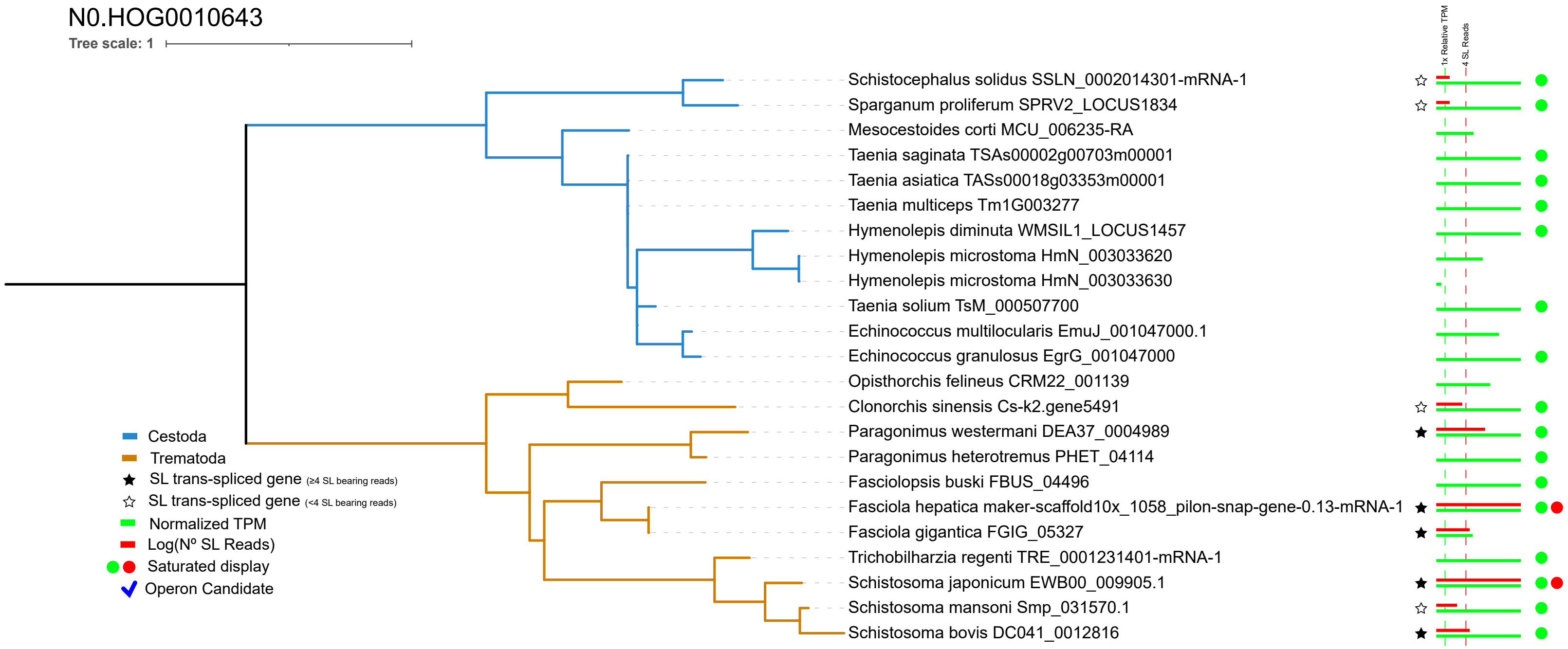

N0.HOG0010643
Tree scale: 1
1x Relative TPM
4 SL Reads
Schistocephalus solidus SSLN_0002014301-mRNA-1
Sparganum proliferum SPRV2_LOCUS1834
Mesocestoides corti MCU_006235-RA
Taenia saginata TSAs00002g00703m00001
Taenia asiatica TASs00018g03353m00001
Taenia multiceps Tm1G003277
Hymenolepis diminuta WMSIL1_LOCUS1457
Hymenolepis microstoma HmN_003033620
Hymenolepis microstoma HmN_003033630
Taenia solium TsM_000507700
Echinococcus multilocularis EmuJ_001047000.1
Echinococcus granulosus EgrG_001047000
Opisthorchis felineus CRM22_001139
Clonorchis sinensis Cs-k2.gene5491
Paragonimus westermani DEA37_0004989
Paragonimus heterotremus PHET_04114
Fasciolopsis buski FBUS_04496
Fasciola hepatica maker-scaffold10x_1058_pilon-snap-gene-0.13-mRNA-1
Fasciola gigantica FGIG_05327
Trichobilharzia regenti TRE_0001231401-mRNA-1
Schistosoma japonicum EWB00_009905.1
Schistosoma mansoni Smp_031570.1
Schistosoma bovis DC041_0012816
Cestoda
Trematoda
SL trans-spliced gene (≥4 SL bearing reads)
SL trans-spliced gene (<4 SL bearing reads)
Normalized TPM
Log(Nº SL Reads)
Saturated display
Operon Candidate

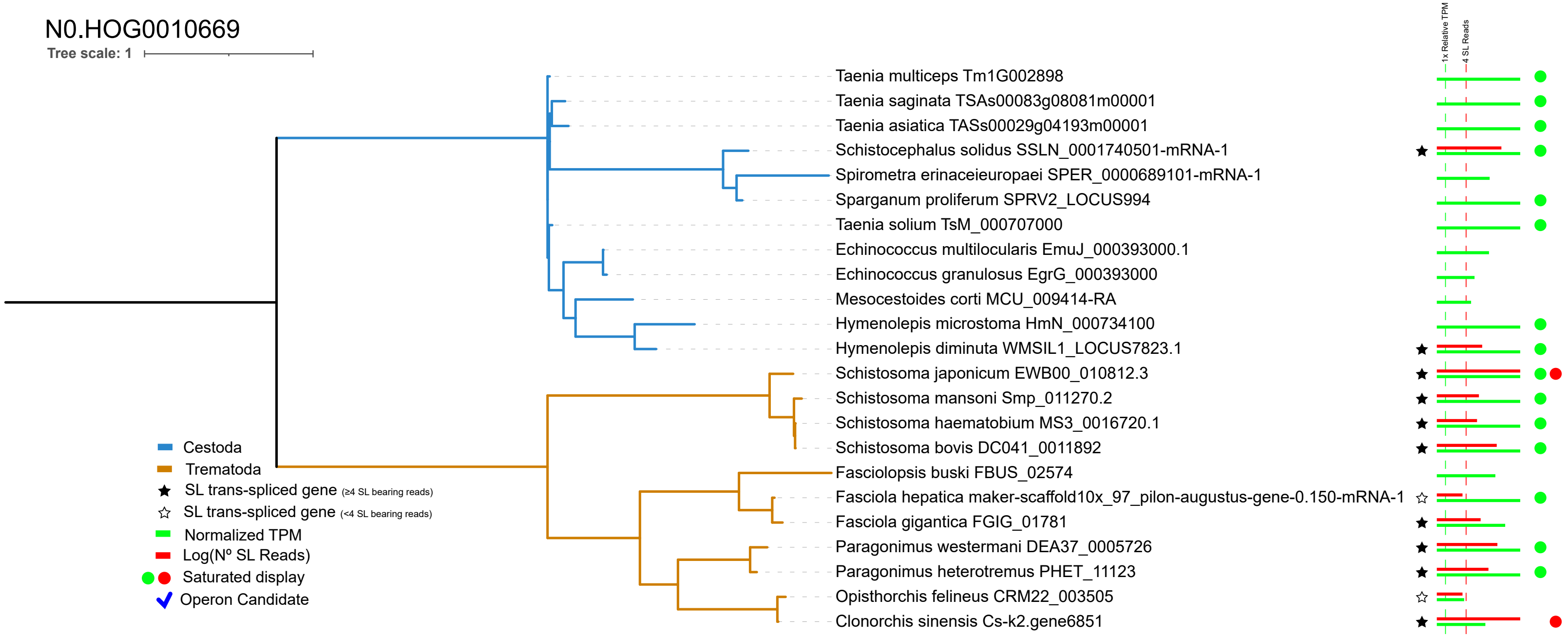
N0.HOG0010669
Tree scale: 1
1x Relative TPM
4 SL Reads
Taenia multiceps Tm1G002898
Taenia saginata TSAs00083g08081m00001
Taenia asiatica TASs00029g04193m00001
Schistocephalus solidus SSLN_0001740501-mRNA-1
Spirometra erinaceieuropaei SPER_0000689101-mRNA-1
Sparganum proliferum SPRV2_LOCUS994
Taenia solium TsM_000707000
Echinococcus multilocularis EmuJ_000393000.1
Echinococcus granulosus EgrG_000393000
Mesocestoides corti MCU_009414-RA
Hymenolepis microstoma HmN_000734100
Hymenolepis diminuta WMSIL1_LOCUS7823.1
Schistosoma japonicum EWB00_010812.3
Schistosoma mansoni Smp_011270.2
Schistosoma haematobium MS3_0016720.1
Schistosoma bovis DC041_0011892
Fasciolopsis buski FBUS_02574
Fasciola hepatica maker-scaffold10x_97_pilon-augustus-gene-0.150-mRNA-1
Fasciola gigantica FGIG_01781
Paragonimus westermani DEA37_0005726
Paragonimus heterotremus PHET_11123
Opisthorchis felineus CRM22_003505
Clonorchis sinensis Cs-k2.gene6851
Cestoda
Trematoda
SL trans-spliced gene (≥4 SL bearing reads)
SL trans-spliced gene (<4 SL bearing reads)
Normalized TPM
Log(Nº SL Reads)
Saturated display
Operon Candidate

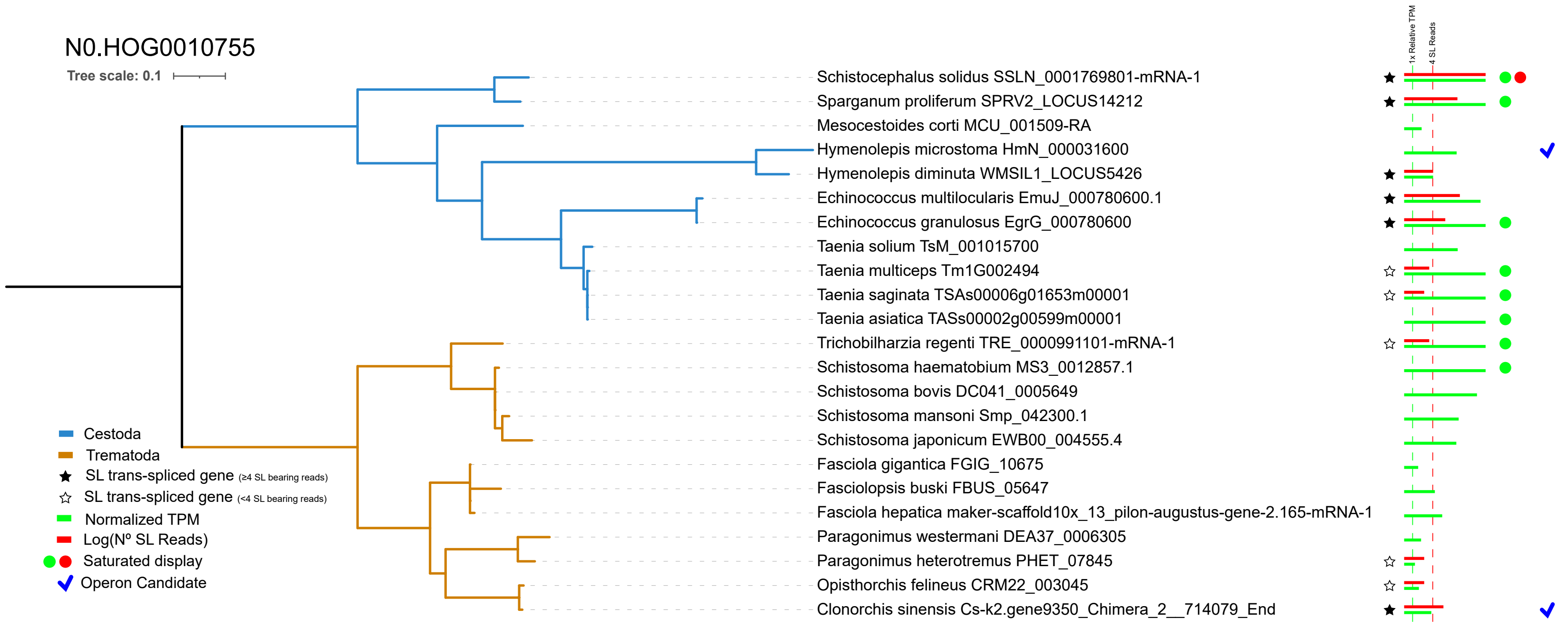
N0.HOG0010755
Tree scale: 0.1
1x Relative TPM
4 SL Reads
Schistocephalus solidus SSLN_0001769801-mRNA-1
Sparganum proliferum SPRV2_LOCUS14212
Mesocestoides corti MCU_001509-RA
Hymenolepis microstoma HmN_000031600
Hymenolepis diminuta WMSIL1_LOCUS5426
Echinococcus multilocularis EmuJ_000780600.1
Echinococcus granulosus EgrG_000780600
Taenia solium TsM_001015700
Taenia multiceps Tm1G002494
Taenia saginata TSAs00006g01653m00001
Taenia asiatica TASs00002g00599m00001
Trichobilharzia regenti TRE_0000991101-mRNA-1
Schistosoma haematobium MS3_0012857.1
Schistosoma bovis DC041_0005649
Schistosoma mansoni Smp_042300.1
Schistosoma japonicum EWB00_004555.4
Fasciola gigantica FGIG_10675
Fasciolopsis buski FBUS_05647
Fasciola hepatica maker-scaffold10x_13_pilon-augustus-gene-2.165-mRNA-1
Paragonimus westermani DEA37_0006305
Paragonimus heterotremus PHET_07845
Opisthorchis felineus CRM22_003045
Clonorchis sinensis Cs-k2.gene9350_Chimera_2__714079_End
Cestoda
Trematoda
SL trans-spliced gene (≥4 SL bearing reads)
SL trans-spliced gene (<4 SL bearing reads)
Normalized TPM
Log(Nº SL Reads)
Saturated display
Operon Candidate

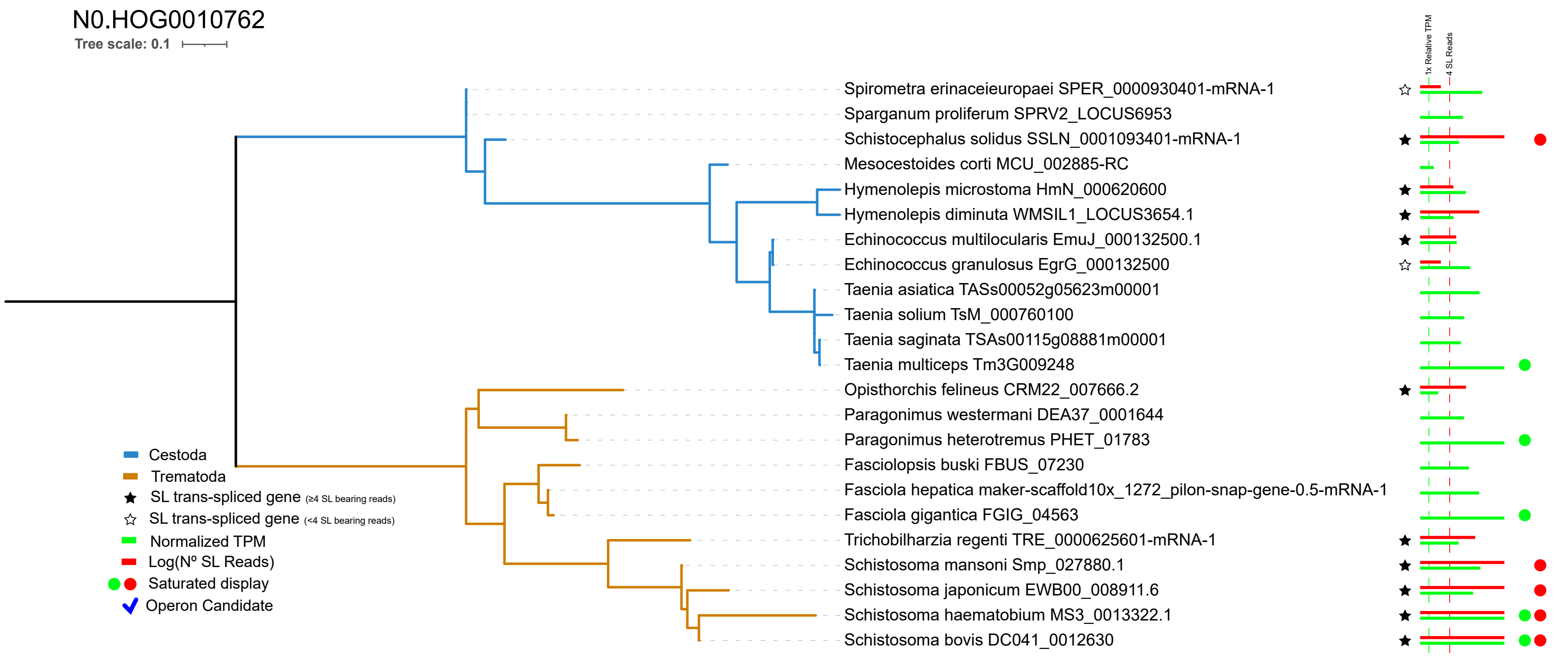

N0.HOG0010762
Tree scale: 0.1
1x Relative TPM
4 SL Reads
Spirometra erinaceieuropaei SPER_0000930401-mRNA-1
Sparganum proliferum SPRV2_LOCUS6953
Schistocephalus solidus SSLN_0001093401-mRNA-1
Mesocestoides corti MCU_002885-RC
Hymenolepis microstoma HmN_000620600
Hymenolepis diminuta WMSIL1_LOCUS3654.1
Echinococcus multilocularis EmuJ_000132500.1
Echinococcus granulosus EgrG_000132500
Taenia asiatica TASs00052g05623m00001
Taenia solium TsM_000760100
Taenia saginata TSAs00115g08881m00001
Taenia multiceps Tm3G009248
Opisthorchis felineus CRM22_007666.2
Paragonimus westermani DEA37_0001644
Paragonimus heterotremus PHET_01783
Fasciolopsis buski FBUS_07230
Fasciola hepatica maker-scaffold10x_1272_pilon-snap-gene-0.5-mRNA-1
Fasciola gigantica FGIG_04563
Trichobilharzia regenti TRE_0000625601-mRNA-1
Schistosoma mansoni Smp_027880.1
Schistosoma japonicum EWB00_008911.6
Schistosoma haematobium MS3_0013322.1
Schistosoma bovis DC041_0012630
Cestoda
Trematoda
SL trans-spliced gene (≥4 SL bearing reads)
SL trans-spliced gene (<4 SL bearing reads)
Normalized TPM
Log(Nº SL Reads)
Saturated display
Operon Candidate

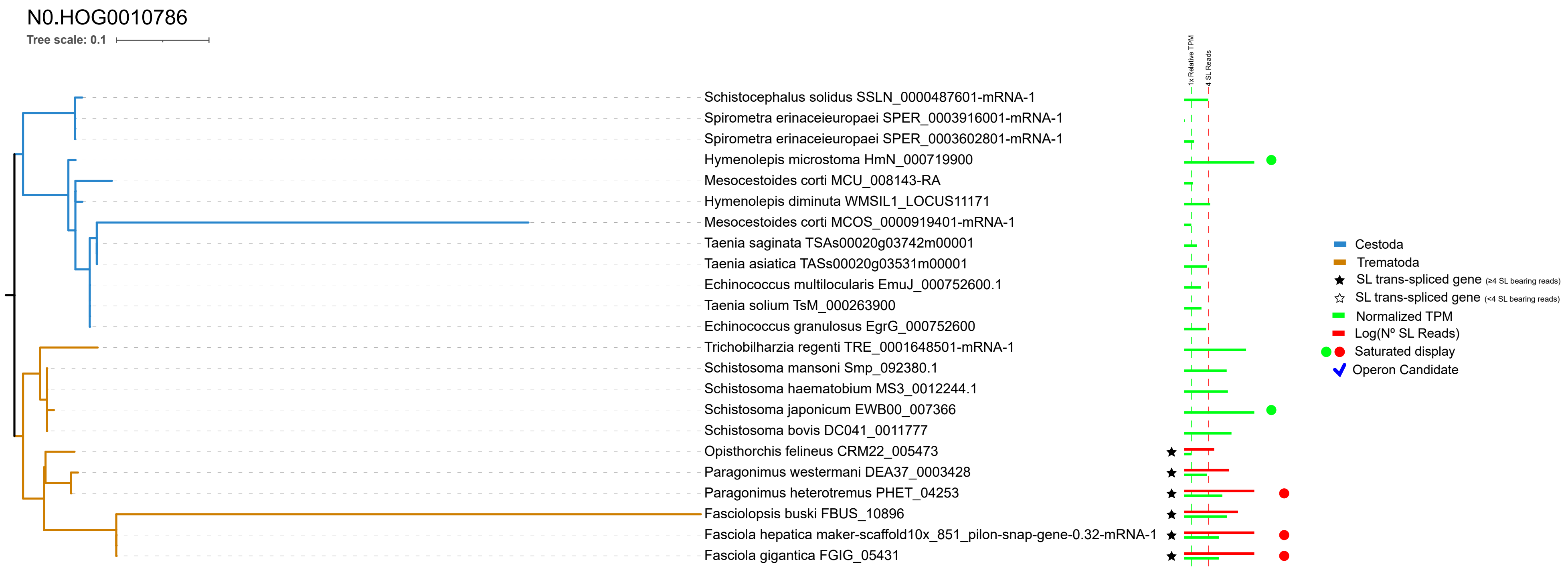

N0.HOG0010786
Tree scale: 0.1
1x Relative TPM
4 SL Reads
Schistocephalus solidus SSLN_0000487601-mRNA-1
Spirometra erinaceieuropaei SPER_0003916001-mRNA-1
Spirometra erinaceieuropaei SPER_0003602801-mRNA-1
Hymenolepis microstoma HmN_000719900
Mesocestoides corti MCU_008143-RA
Hymenolepis diminuta WMSIL1_LOCUS11171
Mesocestoides corti MCOS_0000919401-mRNA-1
Taenia saginata TSAs00020g03742m00001
Taenia asiatica TASs00020g03531m00001
Echinococcus multilocularis EmuJ_000752600.1
Taenia solium TsM_000263900
Echinococcus granulosus EgrG_000752600
Trichobilharzia regenti TRE_0001648501-mRNA-1
Schistosoma mansoni Smp_092380.1
Schistosoma haematobium MS3_0012244.1
Schistosoma japonicum EWB00_007366
Schistosoma bovis DC041_0011777
Opisthorchis felineus CRM22_005473
Paragonimus westermani DEA37_0003428
Paragonimus heterotremus PHET_04253
Fasciolopsis buski FBUS_10896
Fasciola hepatica maker-scaffold10x_851_pilon-snap-gene-0.32-mRNA-1
Fasciola gigantica FGIG_05431
Cestoda
Trematoda
SL trans-spliced gene (≥4 SL bearing reads)
SL trans-spliced gene (<4 SL bearing reads)
Normalized TPM
Log(Nº SL Reads)
Saturated display
Operon Candidate

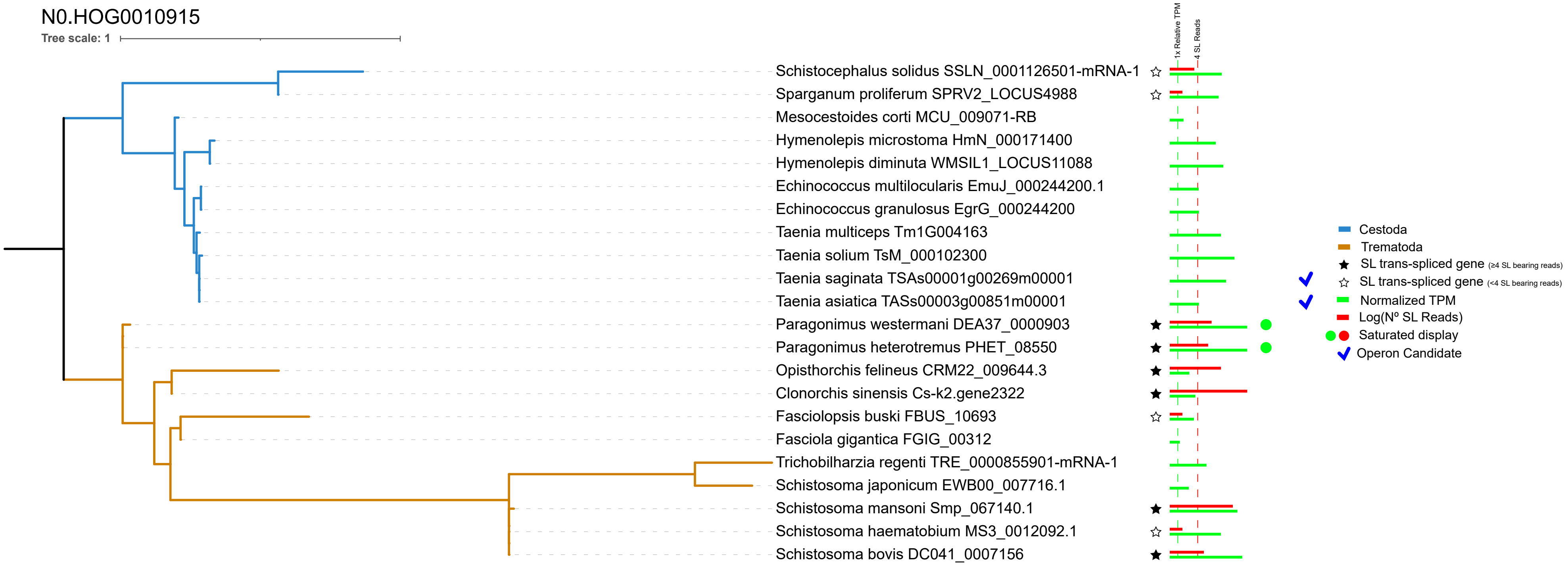
N0.HOG0010915
Tree scale: 1
1x Relative TPM
4 SL Reads
Schistocephalus solidus SSLN_0001126501-mRNA-1
Sparganum proliferum SPRV2_LOCUS4988
Mesocestoides corti MCU_009071-RB
Hymenolepis microstoma HmN_000171400
Hymenolepis diminuta WMSIL1_LOCUS11088
Echinococcus multilocularis EmuJ_000244200.1
Echinococcus granulosus EgrG_000244200
Taenia multiceps Tm1G004163
Taenia solium TsM_000102300
Taenia saginata TSAs00001g00269m00001
Taenia asiatica TASs00003g00851m00001
Paragonimus westermani DEA37_0000903
Paragonimus heterotremus PHET_08550
Opisthorchis felineus CRM22_009644.3
Clonorchis sinensis Cs-k2.gene2322
Fasciolopsis buski FBUS_10693
Fasciola gigantica FGIG_00312
Trichobilharzia regenti TRE_0000855901-mRNA-1
Schistosoma japonicum EWB00_007716.1
Schistosoma mansoni Smp_067140.1
Schistosoma haematobium MS3_0012092.1
Schistosoma bovis DC041_0007156
Cestoda
Trematoda
SL trans-spliced gene (≥4 SL bearing reads)
SL trans-spliced gene (<4 SL bearing reads)
Normalized TPM
Log(Nº SL Reads)
Saturated display
Operon Candidate

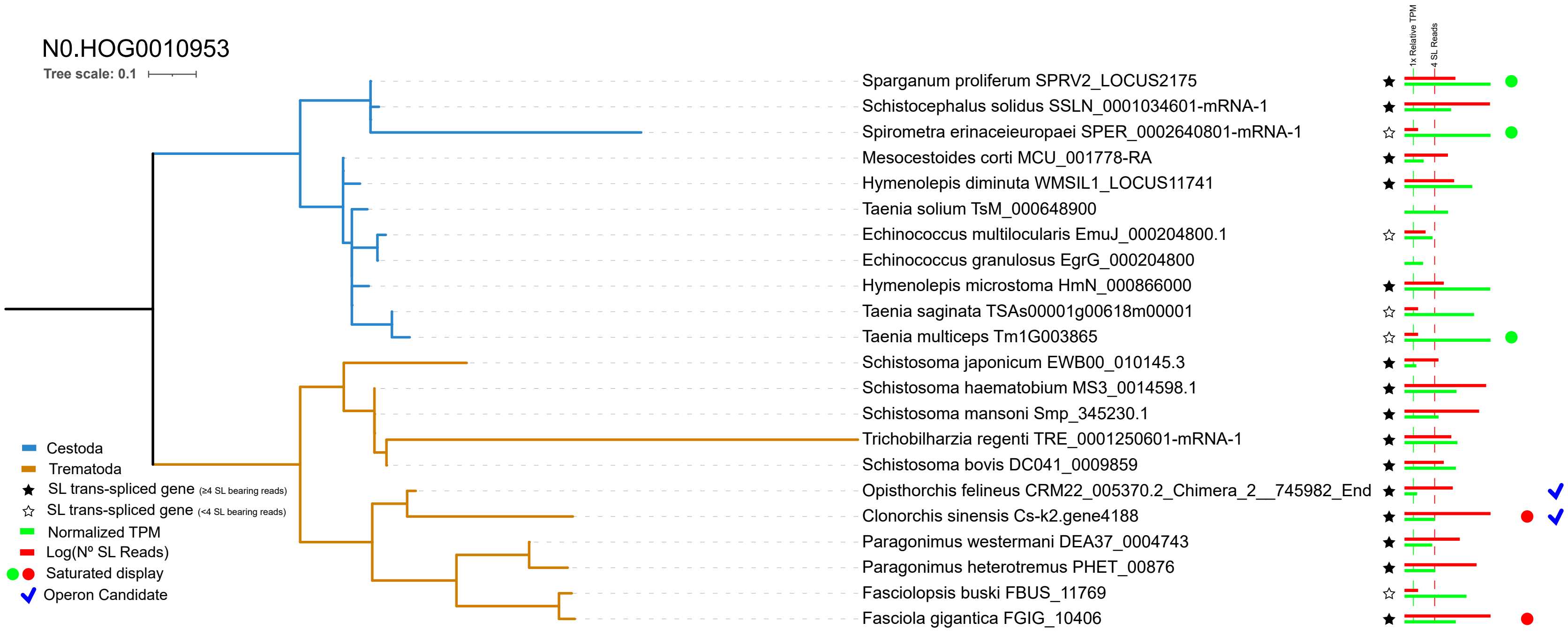

N0.HOG0010953
Tree scale: 0.1
1x Relative TPM
4 SL Reads
Sparganum proliferum SPRV2_LOCUS2175
Schistocephalus solidus SSLN_0001034601-mRNA-1
Spirometra erinaceieuropaei SPER_0002640801-mRNA-1
Mesocestoides corti MCU_001778-RA
Hymenolepis diminuta WMSIL1_LOCUS11741
Taenia solium TsM_000648900
Echinococcus multilocularis EmuJ_000204800.1
Echinococcus granulosus EgrG_000204800
Hymenolepis microstoma HmN_000866000
Taenia saginata TSAs00001g00618m00001
Taenia multiceps Tm1G003865
Schistosoma japonicum EWB00_010145.3
Schistosoma haematobium MS3_0014598.1
Schistosoma mansoni Smp_345230.1
Trichobilharzia regenti TRE_0001250601-mRNA-1
Schistosoma bovis DC041_0009859
Opisthorchis felineus CRM22_005370.2_Chimera_2__745982_End
Clonorchis sinensis Cs-k2.gene4188
Paragonimus westermani DEA37_0004743
Paragonimus heterotremus PHET_00876
Fasciolopsis buski FBUS_11769
Fasciola gigantica FGIG_10406
Cestoda
Trematoda
SL trans-spliced gene (≥4 SL bearing reads)
SL trans-spliced gene (<4 SL bearing reads)
Normalized TPM
Log(Nº SL Reads)
Saturated display
Operon Candidate

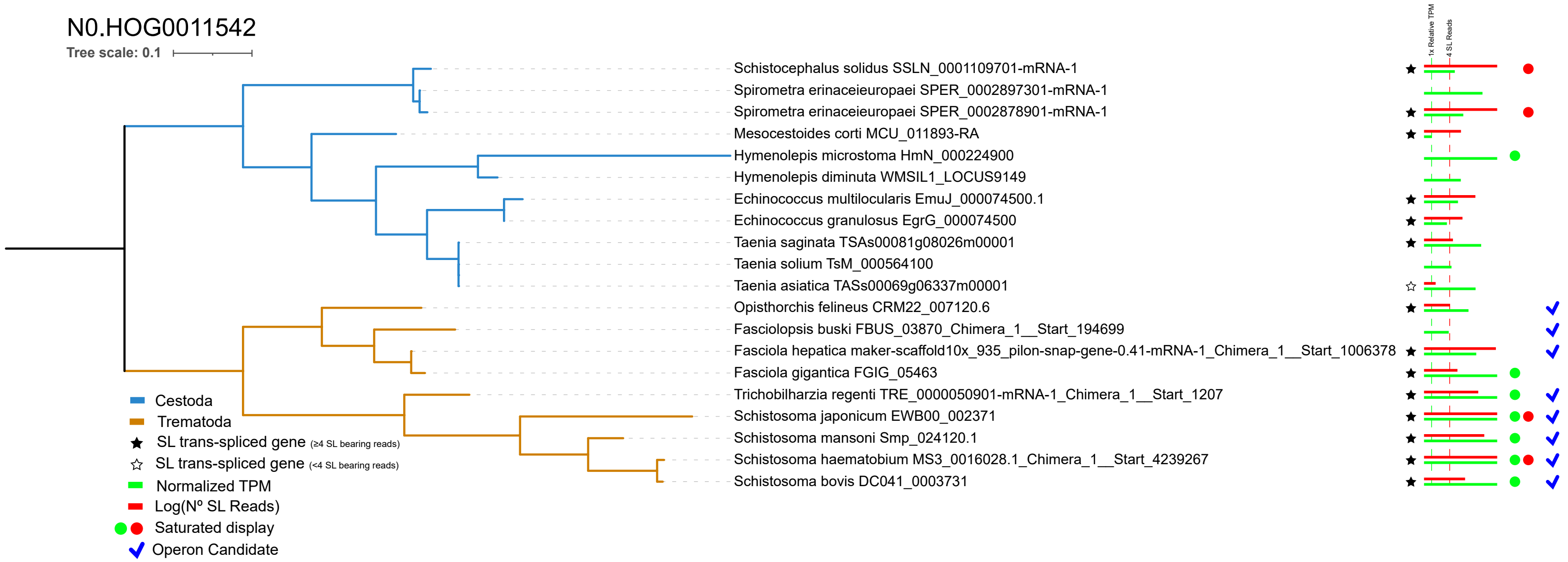

N0.HOG0011542
Tree scale: 0.1
1x Relative TPM
4 SL Reads
Schistocephalus solidus SSLN_0001109701-mRNA-1
Spirometra erinaceieuropaei SPER_0002897301-mRNA-1
Spirometra erinaceieuropaei SPER_0002878901-mRNA-1
Mesocestoides corti MCU_011893-RA
Hymenolepis microstoma HmN_000224900
Hymenolepis diminuta WMSIL1_LOCUS9149
Echinococcus multilocularis EmuJ_000074500.1
Echinococcus granulosus EgrG_000074500
Taenia saginata TSAs00081g08026m00001
Taenia solium TsM_000564100
Taenia asiatica TASs00069g06337m00001
Opisthorchis felineus CRM22_007120.6
Fasciolopsis buski FBUS_03870_Chimera_1__Start_194699
Fasciola hepatica maker-scaffold10x_935_pilon-snap-gene-0.41-mRNA-1_Chimera_1__Start_1006378
Fasciola gigantica FGIG_05463
Trichobilharzia regenti TRE_0000050901-mRNA-1_Chimera_1__Start_1207
Schistosoma japonicum EWB00_002371
Schistosoma mansoni Smp_024120.1
Schistosoma haematobium MS3_0016028.1_Chimera_1__Start_4239267
Schistosoma bovis DC041_0003731
Cestoda
Trematoda
SL trans-spliced gene (≥4 SL bearing reads)
SL trans-spliced gene (<4 SL bearing reads)
Normalized TPM
Log(Nº SL Reads)
Saturated display
Operon Candidate

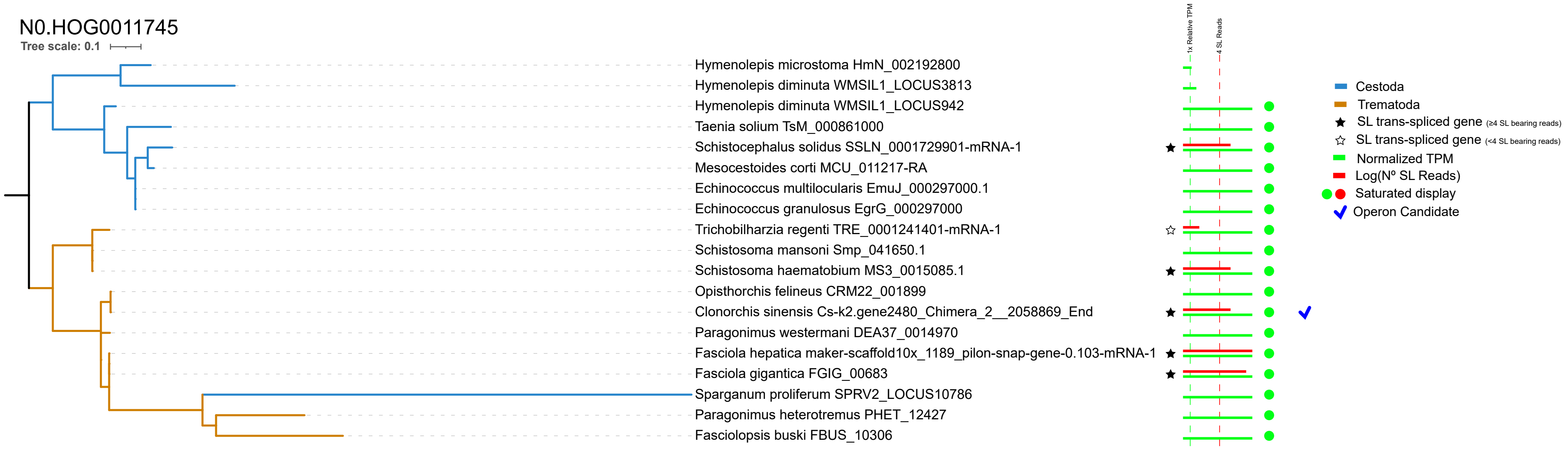
N0.HOG0011745
Tree scale: 0.1
1x Relative TPM
4 SL Reads
Hymenolepis microstoma HmN_002192800
Hymenolepis diminuta WMSIL1_LOCUS3813
Hymenolepis diminuta WMSIL1_LOCUS942
Taenia solium TsM_000861000
Schistocephalus solidus SSLN_0001729901-mRNA-1
Mesocestoides corti MCU_011217-RA
Echinococcus multilocularis EmuJ_000297000.1
Echinococcus granulosus EgrG_000297000
Trichobilharzia regenti TRE_0001241401-mRNA-1
Schistosoma mansoni Smp_041650.1
Schistosoma haematobium MS3_0015085.1
Opisthorchis felineus CRM22_001899
Clonorchis sinensis Cs-k2.gene2480_Chimera_2__2058869_End
Paragonimus westermani DEA37_0014970
Fasciola hepatica maker-scaffold10x_1189_pilon-snap-gene-0.103-mRNA-1
Fasciola gigantica FGIG_00683
Sparganum proliferum SPRV2_LOCUS10786
Paragonimus heterotremus PHET_12427
Fasciolopsis buski FBUS_10306
Cestoda
Trematoda
SL trans-spliced gene (≥4 SL bearing reads)
SL trans-spliced gene (<4 SL bearing reads)
Normalized TPM
Log(Nº SL Reads)
Saturated display
Operon Candidate

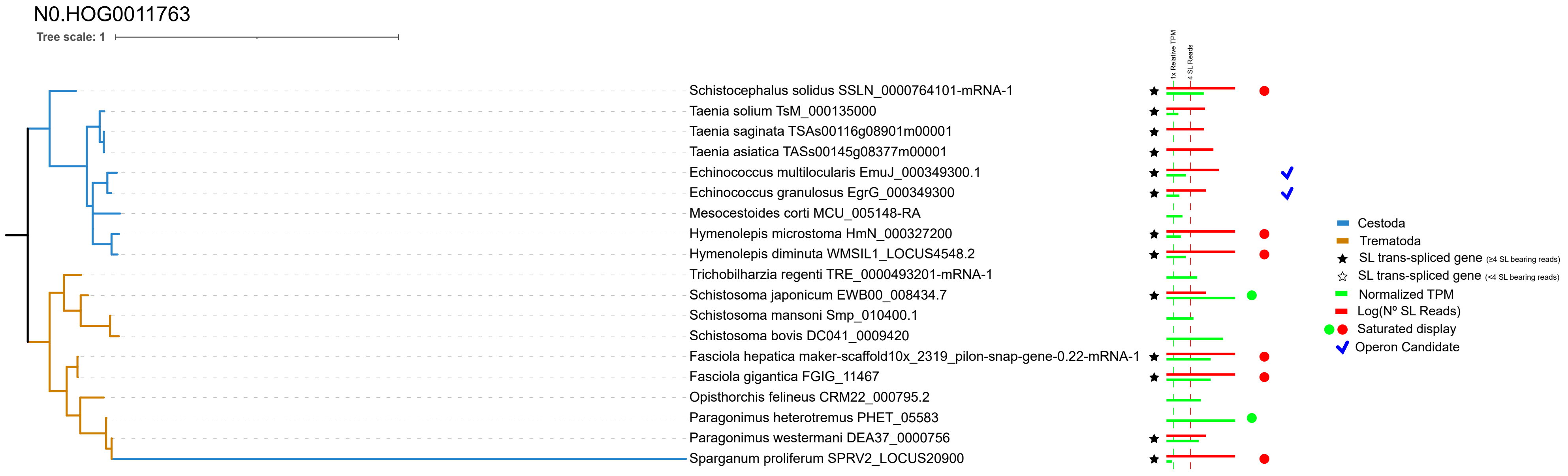

N0.HOG0011763
Tree scale: 1
1x Relative TPM
4 SL Reads
Schistocephalus solidus SSLN_0000764101-mRNA-1
Taenia solium TsM_000135000
Taenia saginata TSAs00116g08901m00001
Taenia asiatica TASs00145g08377m00001
Echinococcus multilocularis EmuJ_000349300.1
Echinococcus granulosus EgrG_000349300
Mesocestoides corti MCU_005148-RA
Hymenolepis microstoma HmN_000327200
Hymenolepis diminuta WMSIL1_LOCUS4548.2
Trichobilharzia regenti TRE_0000493201-mRNA-1
Schistosoma japonicum EWB00_008434.7
Schistosoma mansoni Smp_010400.1
Schistosoma bovis DC041_0009420
Fasciola hepatica maker-scaffold10x_2319_pilon-snap-gene-0.22-mRNA-1
Fasciola gigantica FGIG_11467
Opisthorchis felineus CRM22_000795.2
Paragonimus heterotremus PHET_05583
Paragonimus westermani DEA37_0000756
Sparganum proliferum SPRV2_LOCUS20900
Cestoda
Trematoda
SL trans-spliced gene (≥4 SL bearing reads)
SL trans-spliced gene (<4 SL bearing reads)
Normalized TPM
Log(Nº SL Reads)
Saturated display
Operon Candidate

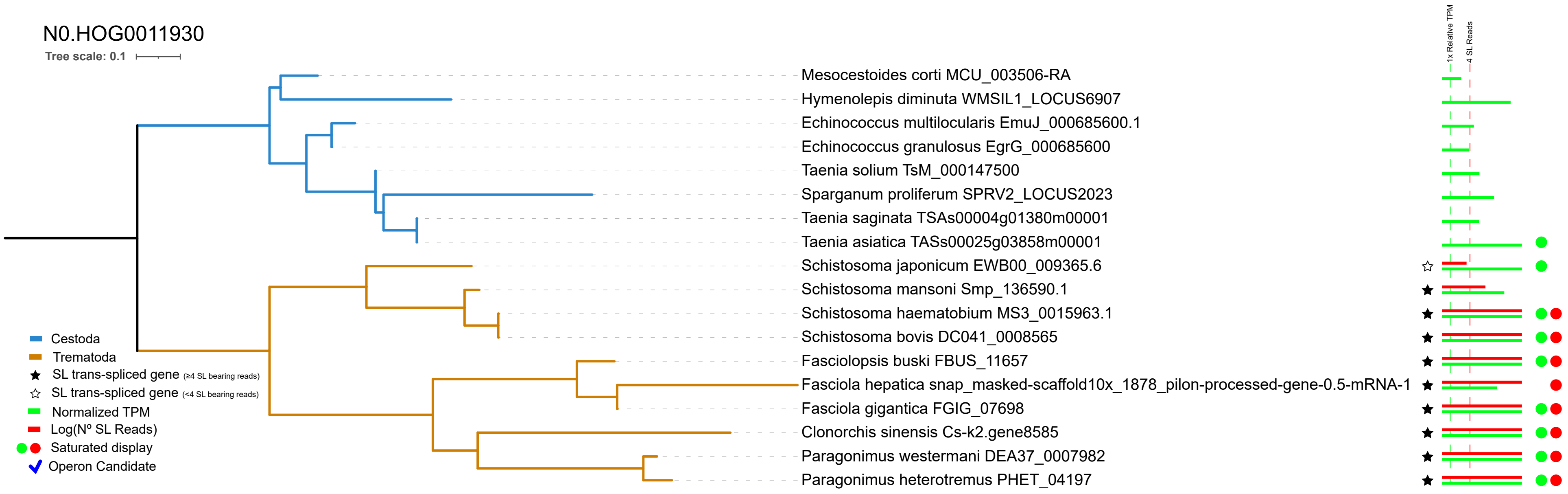
N0.HOG0011930
Tree scale: 0.1
1x Relative TPM
4 SL Reads
Mesocestoides corti MCU_003506-RA
Hymenolepis diminuta WMSIL1_LOCUS6907
Echinococcus multilocularis EmuJ_000685600.1
Echinococcus granulosus EgrG_000685600
Taenia solium TsM_000147500
Sparganum proliferum SPRV2_LOCUS2023
Taenia saginata TSAs00004g01380m00001
Taenia asiatica TASs00025g03858m00001
Schistosoma japonicum EWB00_009365.6
Schistosoma mansoni Smp_136590.1
Schistosoma haematobium MS3_0015963.1
Schistosoma bovis DC041_0008565
Fasciolopsis buski FBUS_11657
Fasciola hepatica snap_masked-scaffold10x_1878_pilon-processed-gene-0.5-mRNA-1
Fasciola gigantica FGIG_07698
Clonorchis sinensis Cs-k2.gene8585
Paragonimus westermani DEA37_0007982
Paragonimus heterotremus PHET_04197
Cestoda
Trematoda
SL trans-spliced gene (≥4 SL bearing reads)
SL trans-spliced gene (<4 SL bearing reads)
Normalized TPM
Log(Nº SL Reads)
Saturated display
Operon Candidate

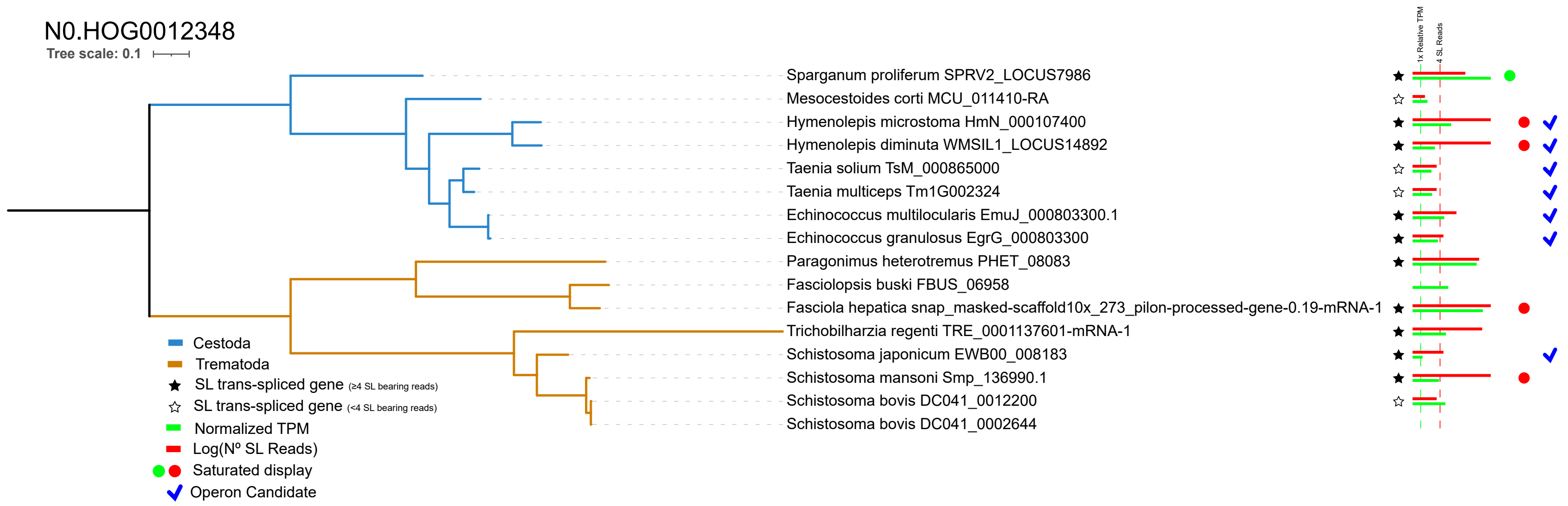

N0.HOG0012348
Tree scale: 0.1
1x Relative TPM
4 SL Reads
Sparganum proliferum SPRV2_LOCUS7986
Mesocestoides corti MCU_011410-RA
Hymenolepis microstoma HmN_000107400
Hymenolepis diminuta WMSIL1_LOCUS14892
Taenia solium TsM_000865000
Taenia multiceps Tm1G002324
Echinococcus multilocularis EmuJ_000803300.1
Echinococcus granulosus EgrG_000803300
Paragonimus heterotremus PHET_08083
Fasciolopsis buski FBUS_06958
Fasciola hepatica snap_masked-scaffold10x_273_pilon-processed-gene-0.19-mRNA-1
Trichobilharzia regenti TRE_0001137601-mRNA-1
Schistosoma japonicum EWB00_008183
Schistosoma mansoni Smp_136990.1
Schistosoma bovis DC041_0012200
Schistosoma bovis DC041_0002644
Cestoda
Trematoda
SL trans-spliced gene (≥4 SL bearing reads)
SL trans-spliced gene (<4 SL bearing reads)
Normalized TPM
Log(Nº SL Reads)
Saturated display
Operon Candidate

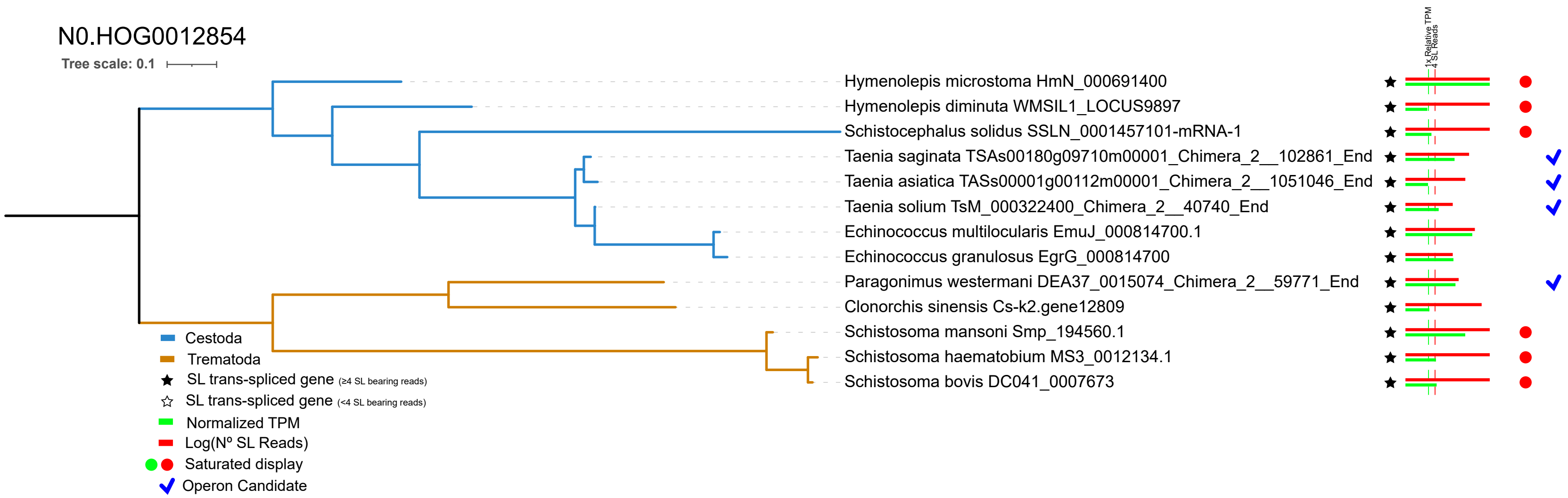

N0.HOG0012854
Tree scale: 0.1
1x Relative TPM
4 SL Reads
Hymenolepis microstoma HmN_000691400
Hymenolepis diminuta WMSIL1_LOCUS9897
Schistocephalus solidus SSLN_0001457101-mRNA-1
Taenia saginata TSAs00180g09710m00001_Chimera_2__102861_End
Taenia asiatica TASs00001g00112m00001_Chimera_2__1051046_End
Taenia solium TsM_000322400_Chimera_2__40740_End
Echinococcus multilocularis EmuJ_000814700.1
Echinococcus granulosus EgrG_000814700
Paragonimus westermani DEA37_0015074_Chimera_2__59771_End
Clonorchis sinensis Cs-k2.gene12809
Schistosoma mansoni Smp_194560.1
Schistosoma haematobium MS3_0012134.1
Schistosoma bovis DC041_0007673
Cestoda
Trematoda
SL trans-spliced gene (≥4 SL bearing reads)
SL trans-spliced gene (<4 SL bearing reads)
Normalized TPM
Log(Nº SL Reads)
Saturated display
Operon Candidate

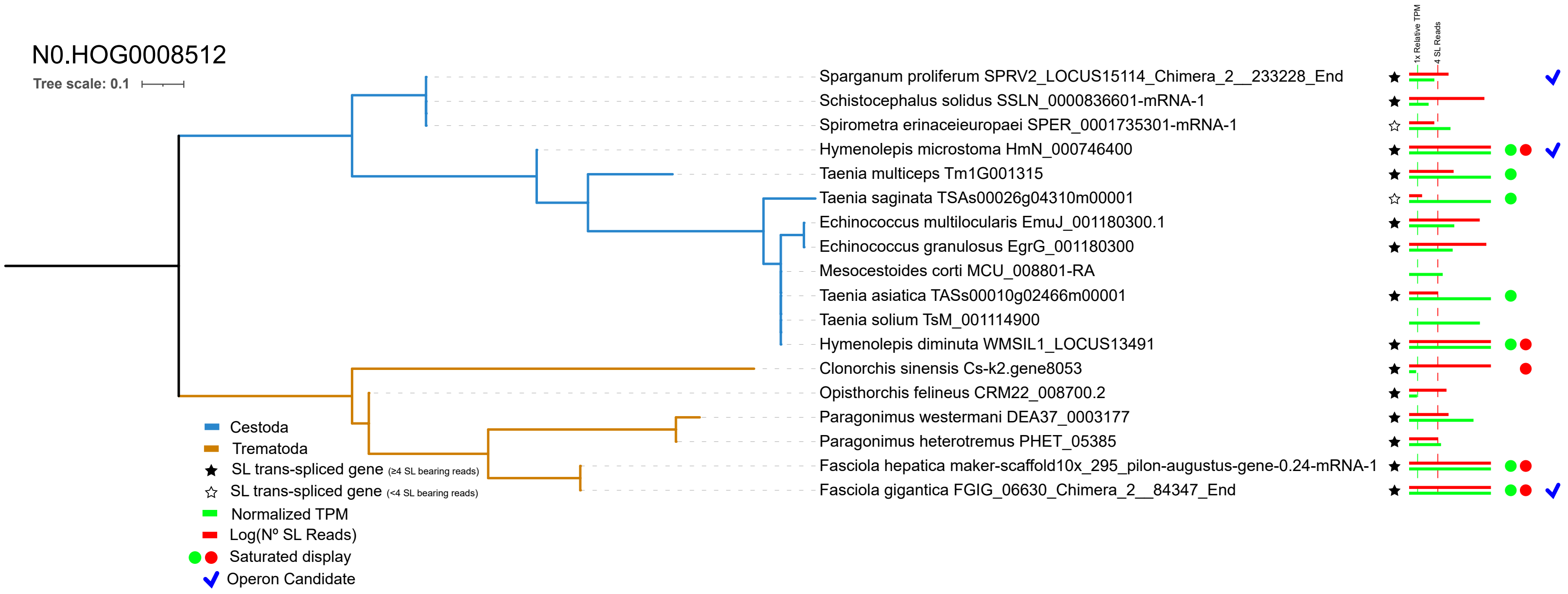

N0.HOG0008512
Tree scale: 0.1
1x Relative TPM
4 SL Reads
Sparganum proliferum SPRV2_LOCUS15114_Chimera_2__233228_End
Schistocephalus solidus SSLN_0000836601-mRNA-1
Spirometra erinaceieuropaei SPER_0001735301-mRNA-1
Hymenolepis microstoma HmN_000746400
Taenia multiceps Tm1G001315
Taenia saginata TSAs00026g04310m00001
Echinococcus multilocularis EmuJ_001180300.1
Echinococcus granulosus EgrG_001180300
Mesocestoides corti MCU_008801-RA
Taenia asiatica TASs00010g02466m00001
Taenia solium TsM_001114900
Hymenolepis diminuta WMSIL1_LOCUS13491
Clonorchis sinensis Cs-k2.gene8053
Opisthorchis felineus CRM22_008700.2
Paragonimus westermani DEA37_0003177
Paragonimus heterotremus PHET_05385
Fasciola hepatica maker-scaffold10x_295_pilon-augustus-gene-0.24-mRNA-1
Fasciola gigantica FGIG_06630_Chimera_2__84347_End
Cestoda
Trematoda
SL trans-spliced gene (≥4 SL bearing reads)
SL trans-spliced gene (<4 SL bearing reads)
Normalized TPM
Log(Nº SL Reads)
Saturated display
Operon Candidate
