# Supplementary material for: Evolution of SL-RNA Genes and Their Splicing Targets in Parasitic Flatworms
Source: Mol Biol Evol. 2025 Sep 23;42(11):msaf228. doi: 10.1093/molbev/msaf228 (PMC12582326; doi:10.1093/molbev/msaf228)
Supplement: msaf228_Supplementary_Data [file msaf228_supplementary_data.zip › Supplementary File 9 - 14082025.pdf]

Supplementary File 9: Sketches of manually analyzed operon candidates, selected based on the exclusiveness of their HOG pairs to either Cestodes or Trematodes, or their association with one of the HOGs selected by expression level.

Index:

Pag. 2: Op1
Pag. 3: Op2
Pag. 4: Op3
Pag. 5: Op4
Pag. 6: Op5
Pag. 7: Op6
Pag. 8: Op7
Pag. 9: Op8
Pag. 10: Op9
Pag. 11: Op10
Pag. 12: Op11
Pag. 13: Op12
Pag. 14: Op13
Pag. 15: Op14
Pag. 16: Op15
Pag. 17: Op16
Pag. 18: Op17
Pag. 19: Op18
Pag. 20: Op19
Pag. 21: Op20
Pag. 22: Op21
Pag. 23: Op22
Pag. 24: Op23
Pag. 25: Op24
Pag. 26: Op25
Pag. 27: Op26

Op1

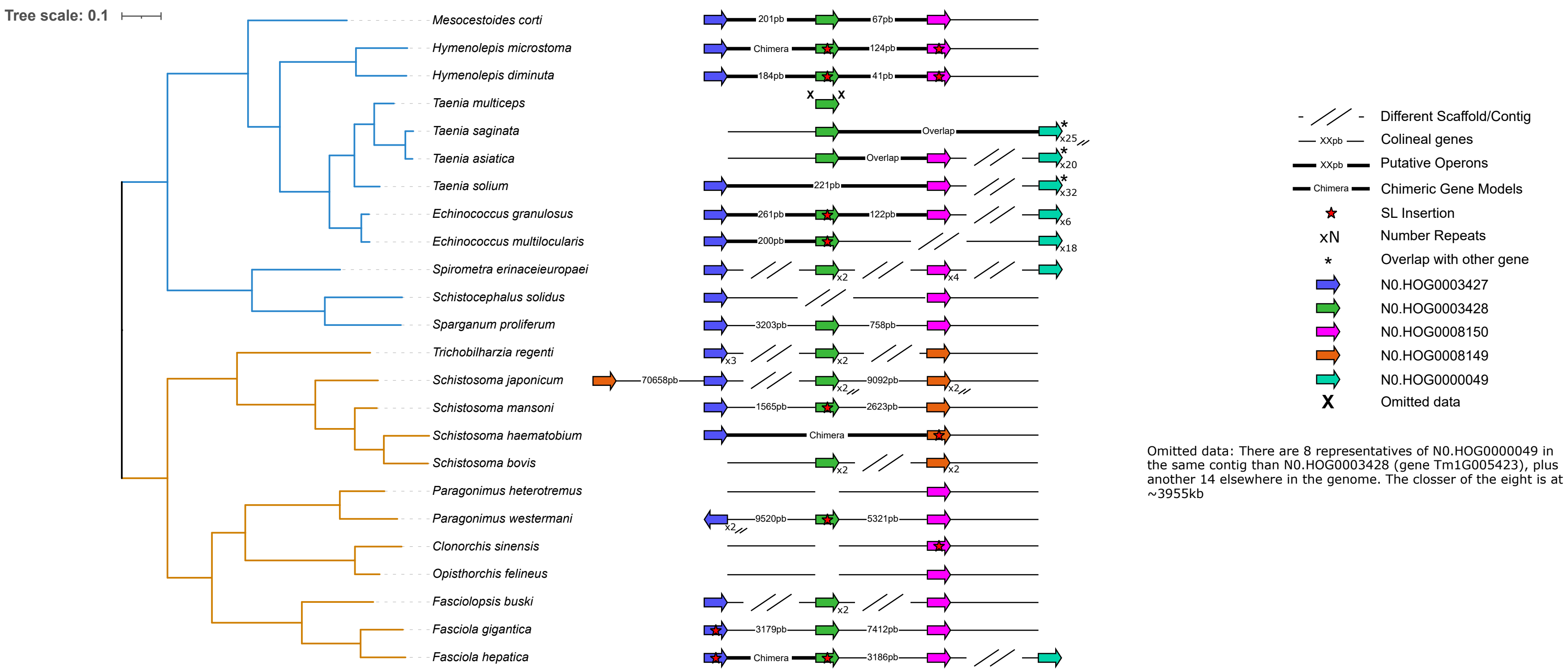
Tree scale: 0.1
Mesocestoides corti
Hymenolepis microstoma
Hymenolepis diminuta
Taenia multiceps
Taenia saginata
Taenia asiatica
Taenia solium
Echinococcus granulosus
Echinococcus multilocularis
Spirometra erinaceieuropaei
Schistocephalus solidus
Sparganum proliferum
Trichobilharzia regenti
Schistosoma japonicum
Schistosoma mansoni
Schistosoma haematobium
Schistosoma bovis
Paragonimus heterotremus
Paragonimus westermani
Clonorchis sinensis
Opisthorchis felineus
Fasciolopsis buski
Fasciola gigantica
Fasciola hepatica
201pb
67pb
Chimera
124pb
184pb
41pb
X
X
Overlap
x25
Overlap
x20
221pb
x32
261pb
122pb
x6
200pb
x18
x2
x4
3203pb
758pb
x3
x2
70658pb
x2
9092pb
x2
1565pb
2623pb
Chimera
x2
x2
9520pb
x2
5321pb
x2
3179pb
7412pb
Chimera
3186pb
Different Scaffold/Contig
XXpb
Colineal genes
XXpb
Putative Operons
Chimera
Chimeric Gene Models
SL Insertion
xN
Number Repeats
*
Overlap with other gene
N0.HOG0003427
N0.HOG0003428
N0.HOG0008150
N0.HOG0008149
N0.HOG0000049
X
Omitted data
Omitted data: There are 8 representatives of N0.HOG0000049 in the same contig than N0.HOG0003428 (gene Tm1G005423), plus another 14 elsewhere in the genome. The closser of the eight is at ~3955kb

Op2

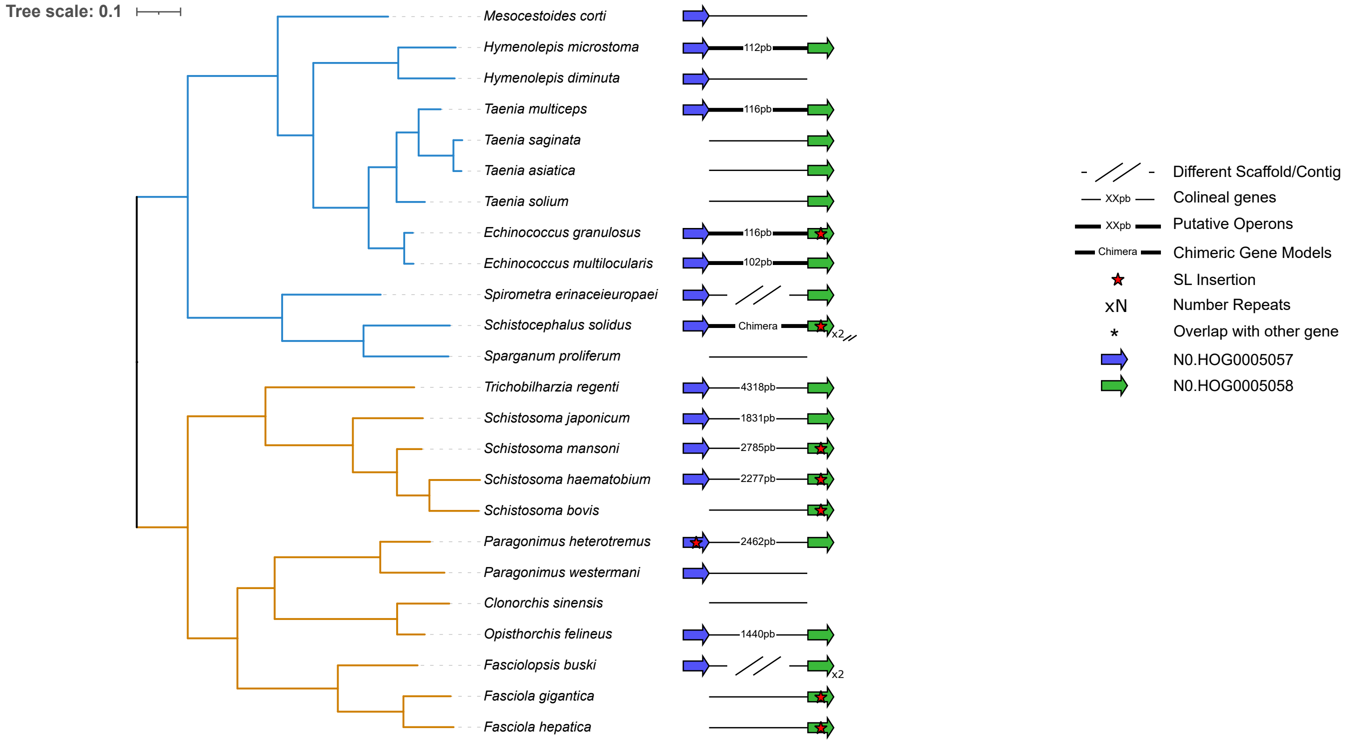

Tree scale: 0.1
Mesocestoides corti
Hymenolepis microstoma
112pb
Hymenolepis diminuta
Taenia multiceps
116pb
Taenia saginata
Taenia asiatica
Taenia solium
Echinococcus granulosus
116pb
Echinococcus multilocularis
102pb
Spirometra erinaceieuropaei
Schistocephalus solidus
Chimera
x2
Sparganum proliferum
Trichobilharzia regenti
4318pb
Schistosoma japonicum
1831pb
Schistosoma mansoni
2785pb
Schistosoma haematobium
2277pb
Schistosoma bovis
Paragonimus heterotremus
2462pb
Paragonimus westermani
Clonorchis sinensis
Opisthorchis felineus
1440pb
Fasciolopsis buski
x2
Fasciola gigantica
Fasciola hepatica
Different Scaffold/Contig
XXpb
Colineal genes
XXpb
Putative Operons
Chimera
Chimeric Gene Models
SL Insertion
xN
Number Repeats
*
Overlap with other gene
N0.HOG0005057
N0.HOG0005058

Op3

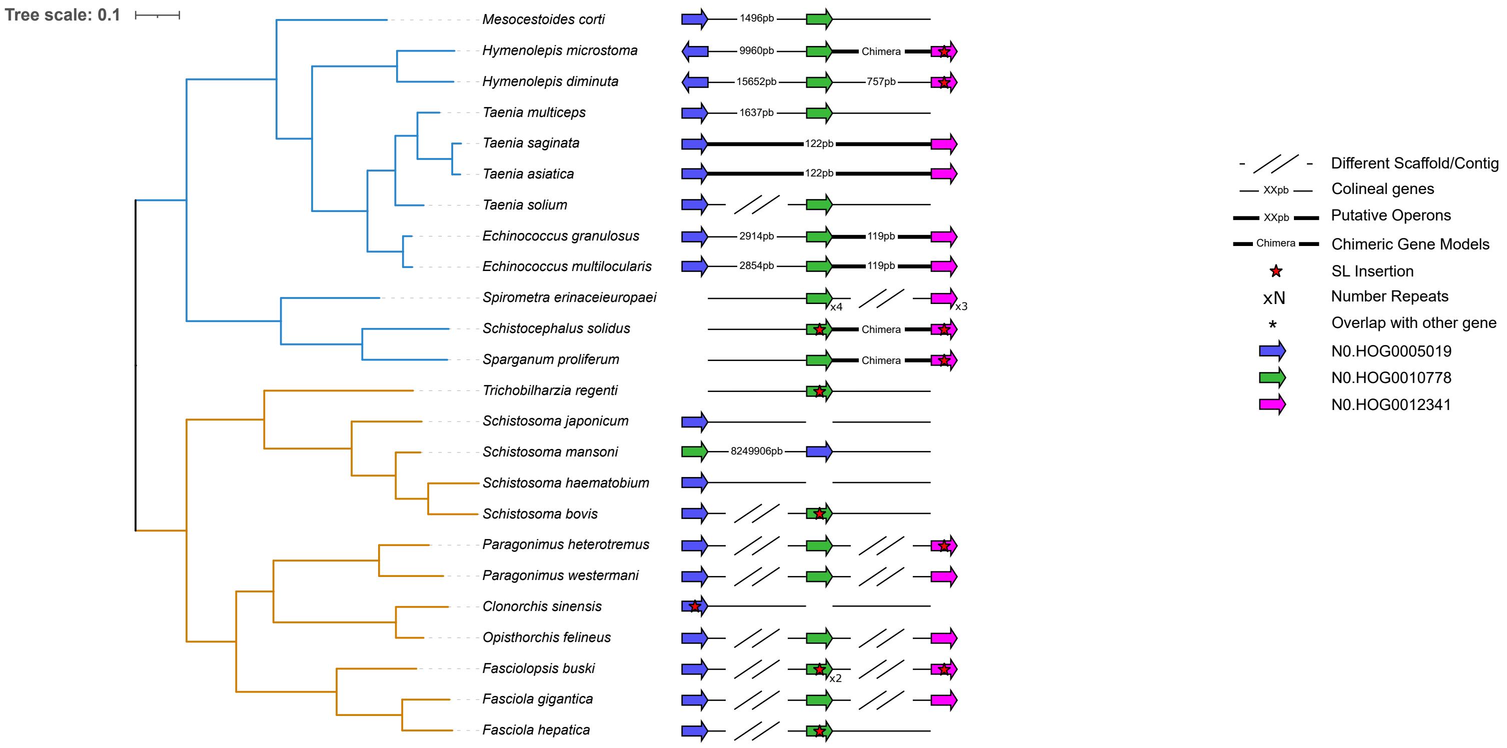
Tree scale: 0.1
Mesocestoides corti
Hymenolepis microstoma
Hymenolepis diminuta
Taenia multiceps
Taenia saginata
Taenia asiatica
Taenia solium
Echinococcus granulosus
Echinococcus multilocularis
Spirometra erinaceieuropaei
Schistocephalus solidus
Sparganum proliferum
Trichobilharzia regenti
Schistosoma japonicum
Schistosoma mansoni
Schistosoma haematobium
Schistosoma bovis
Paragonimus heterotremus
Paragonimus westermani
Clonorchis sinensis
Opisthorchis felineus
Fasciolopsis buski
Fasciola gigantica
Fasciola hepatica
1496pb
9960pb
Chimera
15652pb
757pb
1637pb
122pb
122pb
2914pb
119pb
2854pb
119pb
x4
x3
Chimera
Chimera
8249906pb
x2
Different Scaffold/Contig
XXpb
Colineal genes
XXpb
Putative Operons
Chimera
Chimeric Gene Models
SL Insertion
xN
Number Repeats
*
Overlap with other gene
N0.HOG0005019
N0.HOG0010778
N0.HOG0012341

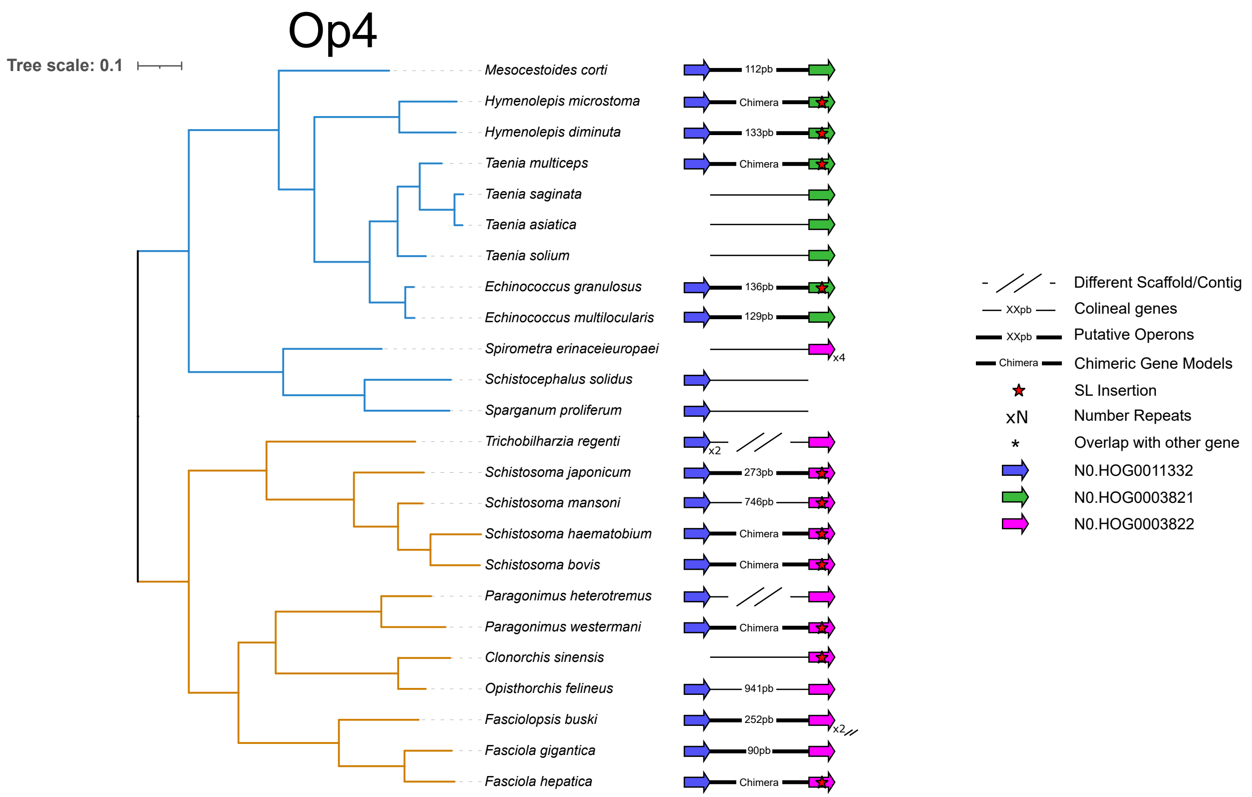
Op4
Tree scale: 0.1
Mesocestoides corti
Hymenolepis microstoma
Hymenolepis diminuta
Taenia multiceps
Taenia saginata
Taenia asiatica
Taenia solium
Echinococcus granulosus
Echinococcus multilocularis
Spirometra erinaceieuropaei
Schistocephalus solidus
Sparganum proliferum
Trichobilharzia regenti
Schistosoma japonicum
Schistosoma mansoni
Schistosoma haematobium
Schistosoma bovis
Paragonimus heterotremus
Paragonimus westermani
Clonorchis sinensis
Opisthorchis felineus
Fasciolopsis buski
Fasciola gigantica
Fasciola hepatica
112pb
Chimera
133pb
Chimera
136pb
129pb
x4
x2
273pb
746pb
Chimera
Chimera
Chimera
941pb
252pb
x2
90pb
Chimera
Different Scaffold/Contig
XXpb
Colineal genes
XXpb
Putative Operons
Chimera
Chimeric Gene Models
SL Insertion
xN
Number Repeats
*
Overlap with other gene
N0.HOG0011332
N0.HOG0003821
N0.HOG0003822

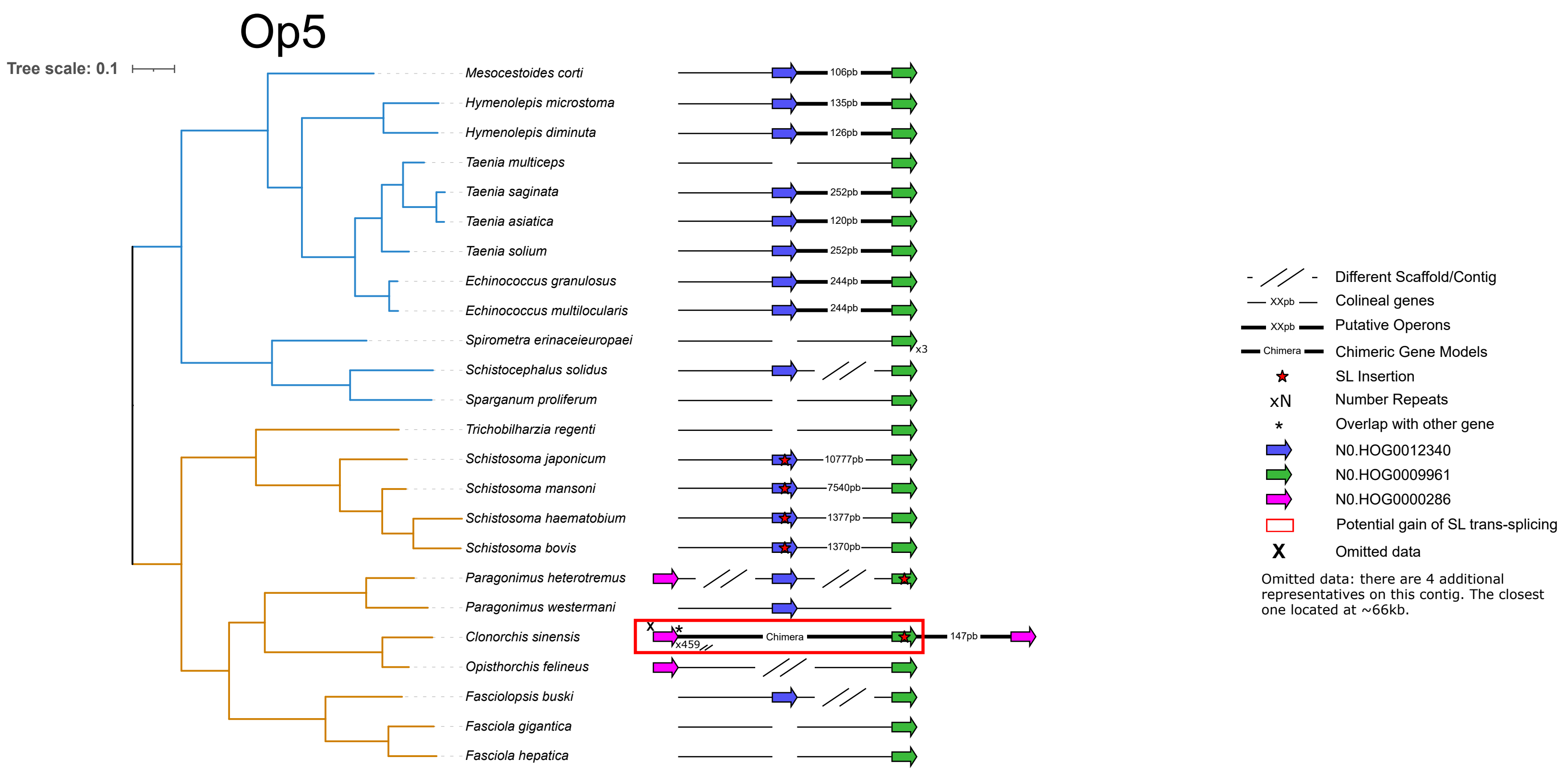

Op5
Tree scale: 0.1
Mesocestoides corti
Hymenolepis microstoma
Hymenolepis diminuta
Taenia multiceps
Taenia saginata
Taenia asiatica
Taenia solium
Echinococcus granulosus
Echinococcus multilocularis
Spirometra erinaceieuropaei
Schistocephalus solidus
Sparganum proliferum
Trichobilharzia regenti
Schistosoma japonicum
Schistosoma mansoni
Schistosoma haematobium
Schistosoma bovis
Paragonimus heterotremus
Paragonimus westermani
Clonorchis sinensis
Opisthorchis felineus
Fasciolopsis buski
Fasciola gigantica
Fasciola hepatica
106pb
135pb
126pb
252pb
120pb
252pb
244pb
244pb
x3
10777pb
7540pb
1377pb
1370pb
X
x459
Chimera
147pb
Different Scaffold/Contig
XXpb
Colineal genes
Putative Operons
Chimeric Gene Models
SL Insertion
xN
Number Repeats
Overlap with other gene
N0.HOG0012340
N0.HOG0009961
N0.HOG0000286
Potential gain of SL trans-splicing
Omitted data
Omitted data: there are 4 additional representatives on this contig. The closest one located at ~66kb.

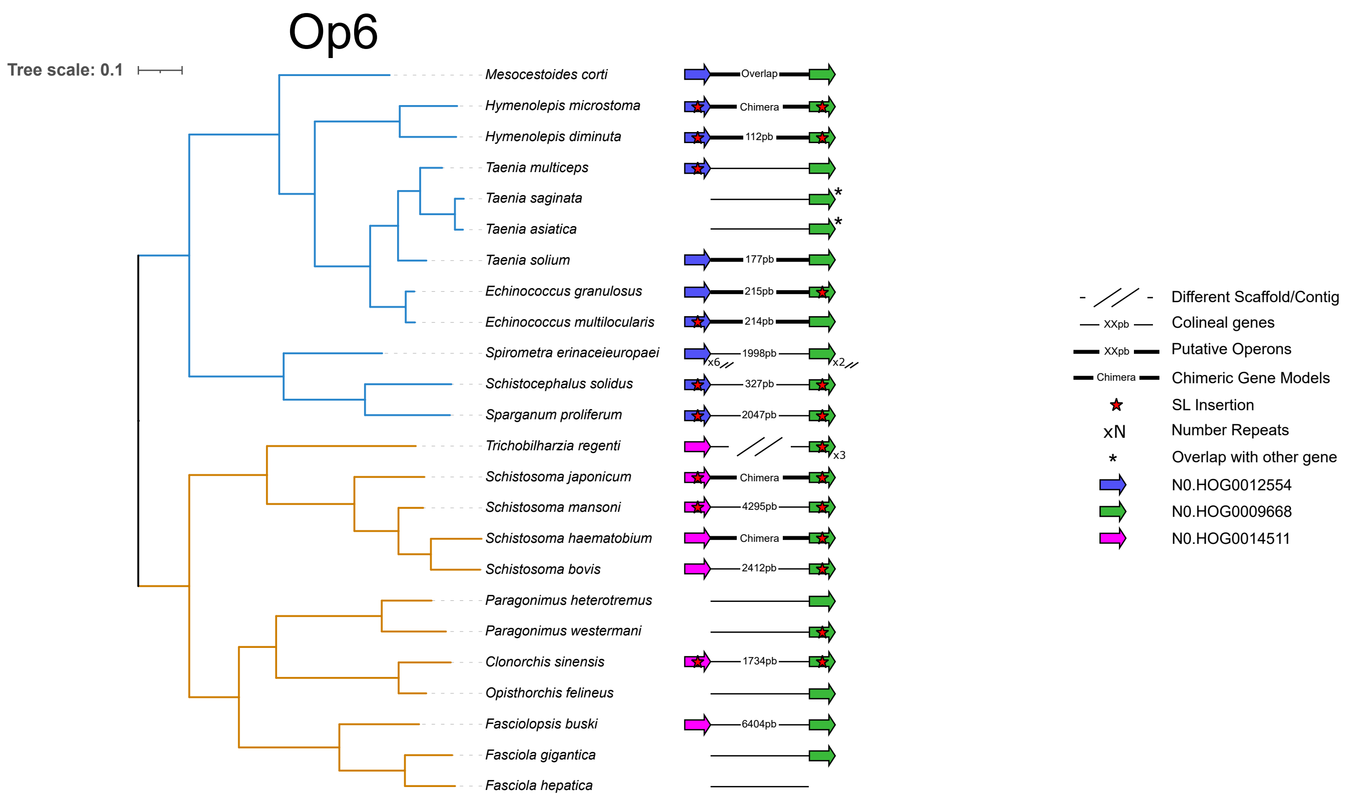
Op6
Tree scale: 0.1
Mesocestoides corti
Hymenolepis microstoma
Hymenolepis diminuta
Taenia multiceps
Taenia saginata
Taenia asiatica
Taenia solium
Echinococcus granulosus
Echinococcus multilocularis
Spirometra erinaceieuropaei
Schistocephalus solidus
Sparganum proliferum
Trichobilharzia regenti
Schistosoma japonicum
Schistosoma mansoni
Schistosoma haematobium
Schistosoma bovis
Paragonimus heterotremus
Paragonimus westermani
Clonorchis sinensis
Opisthorchis felineus
Fasciolopsis buski
Fasciola gigantica
Fasciola hepatica
Overlap
Chimera
112pb
177pb
215pb
214pb
1998pb
x6
x2
327pb
2047pb
x3
Chimera
4295pb
Chimera
2412pb
1734pb
6404pb
Different Scaffold/Contig
XXpb
Colineal genes
XXpb
Putative Operons
Chimera
Chimeric Gene Models
SL Insertion
xN
Number Repeats
*
Overlap with other gene
N0.HOG0012554
N0.HOG0009668
N0.HOG0014511

Op7

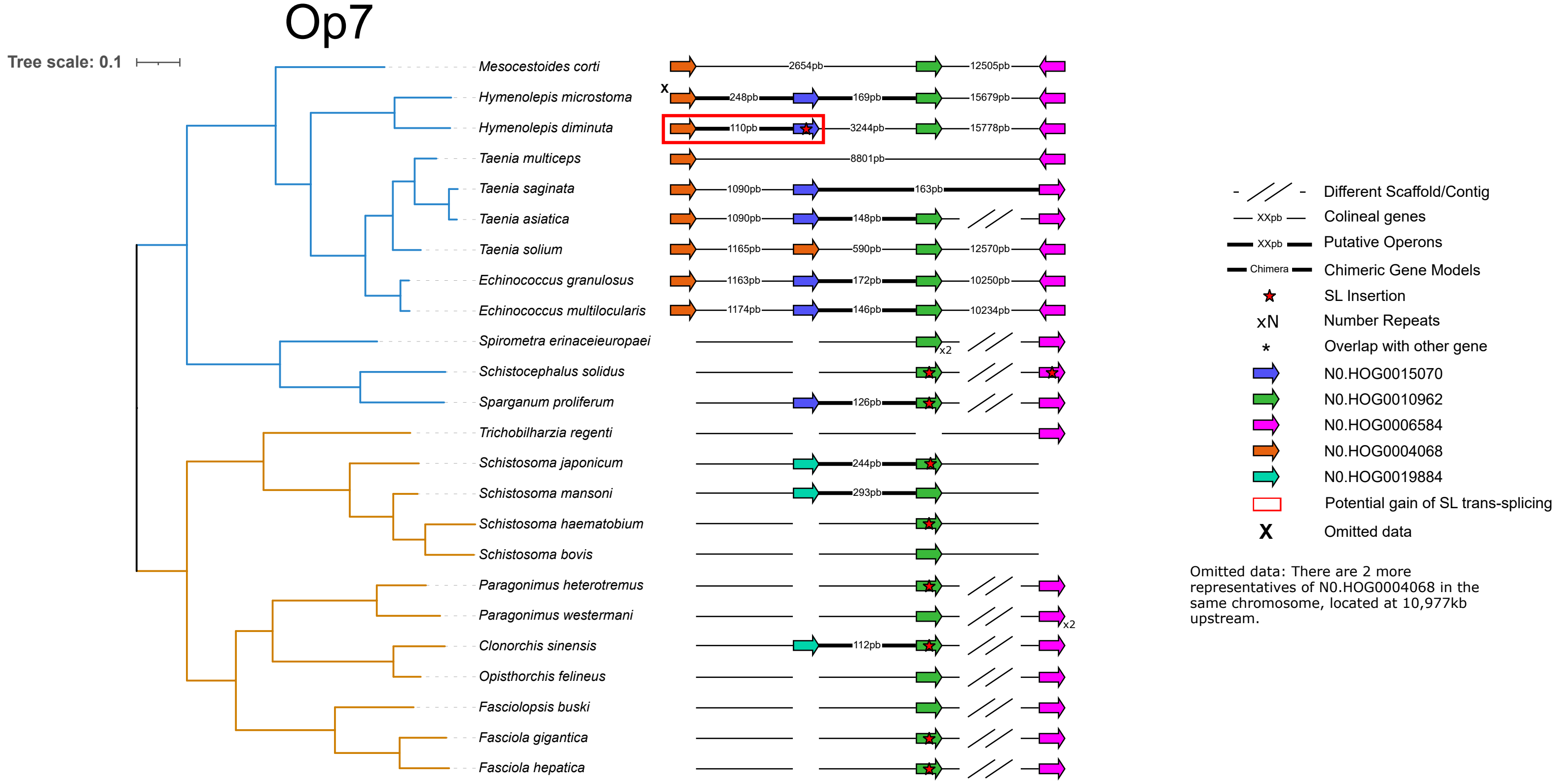

Omitted data: There are 2 more representatives of N0.HOG0004068 in the same chromosome, located at 10,977kb upstream.

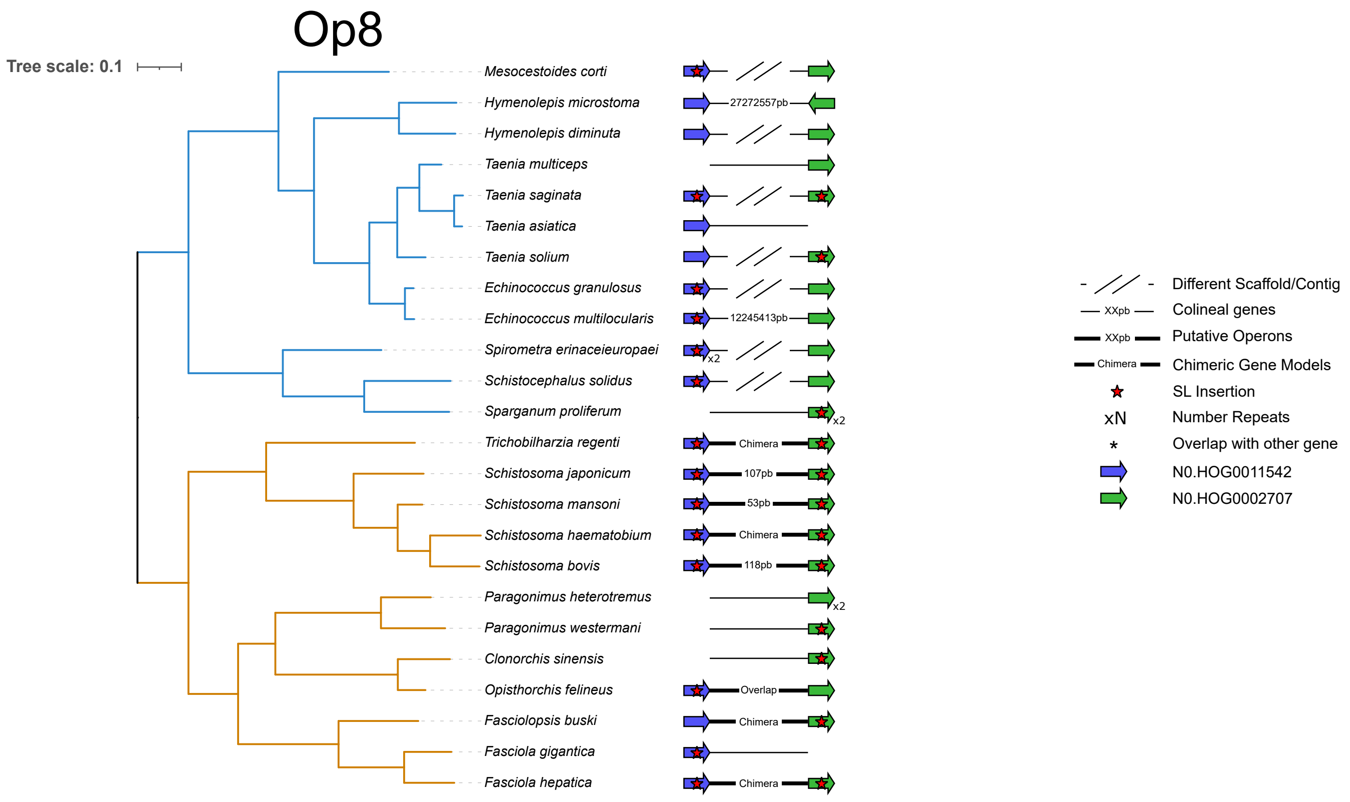
Op8
Tree scale: 0.1
Mesocestoides corti
Hymenolepis microstoma
27272557pb
Hymenolepis diminuta
Taenia multiceps
Taenia saginata
Taenia asiatica
Taenia solium
Echinococcus granulosus
Echinococcus multilocularis
12245413pb
Spirometra erinaceieuropaei
x2
Schistocephalus solidus
Sparganum proliferum
x2
Trichobilharzia regenti
Chimera
Schistosoma japonicum
107pb
Schistosoma mansoni
53pb
Schistosoma haematobium
Chimera
Schistosoma bovis
118pb
Paragonimus heterotremus
x2
Paragonimus westermani
Clonorchis sinensis
Opisthorchis felineus
Overlap
Fasciolopsis buski
Chimera
Fasciola gigantica
Fasciola hepatica
Chimera
Different Scaffold/Contig
XXpb
Colineal genes
XXpb
Putative Operons
Chimera
Chimeric Gene Models
SL Insertion
xN
Number Repeats
*
Overlap with other gene
N0.HOG0011542
N0.HOG0002707

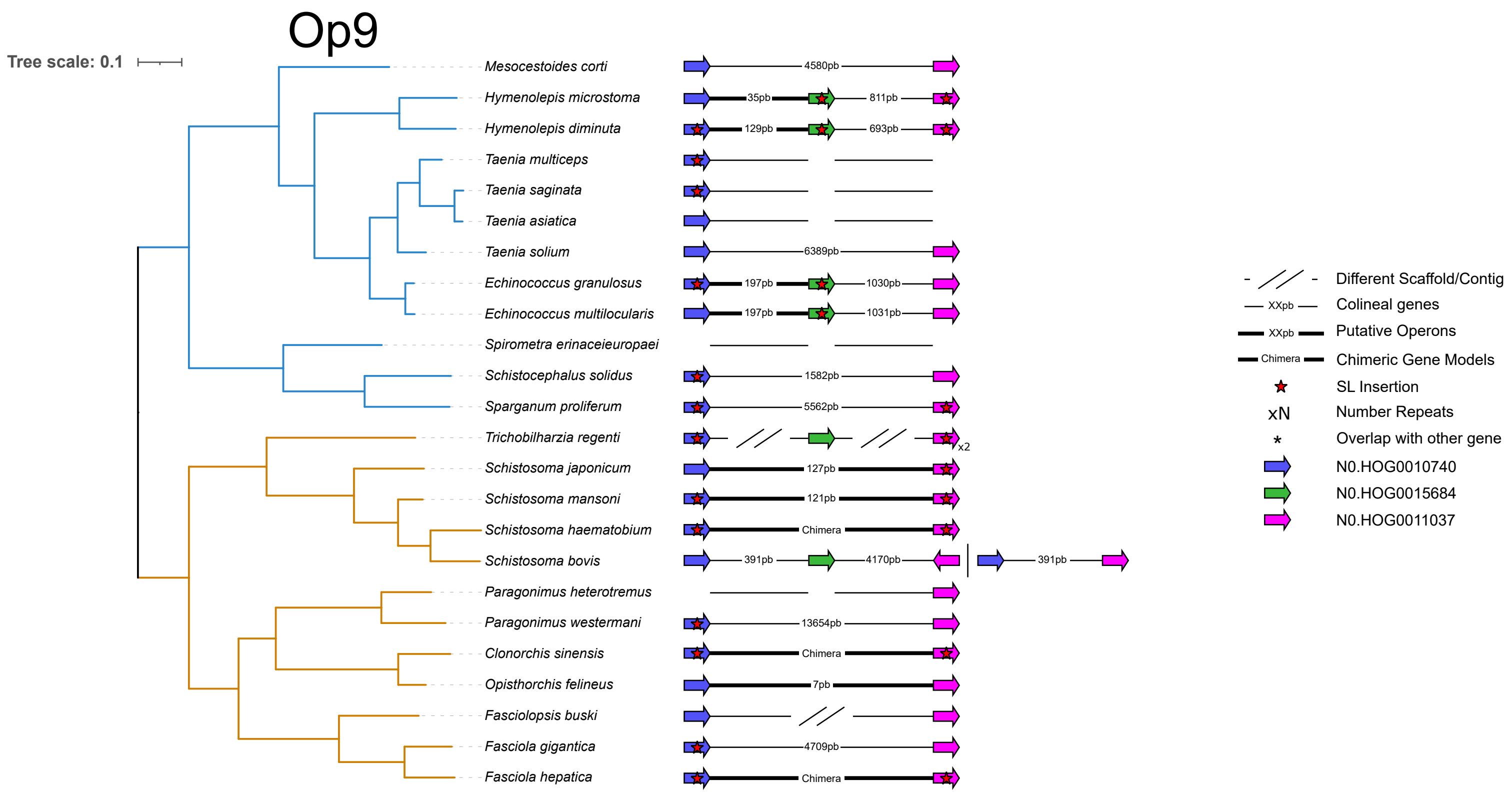

Op9
Tree scale: 0.1
Mesocestoides corti
Hymenolepis microstoma
Hymenolepis diminuta
Taenia multiceps
Taenia saginata
Taenia asiatica
Taenia solium
Echinococcus granulosus
Echinococcus multilocularis
Spirometra erinaceieuropaei
Schistocephalus solidus
Sparganum proliferum
Trichobilharzia regenti
Schistosoma japonicum
Schistosoma mansoni
Schistosoma haematobium
Schistosoma bovis
Paragonimus heterotremus
Paragonimus westermani
Clonorchis sinensis
Opisthorchis felineus
Fasciolopsis buski
Fasciola gigantica
Fasciola hepatica
4580pb
35pb
811pb
129pb
693pb
6389pb
197pb
1030pb
197pb
1031pb
1582pb
5562pb
x2
127pb
121pb
Chimera
391pb
4170pb
391pb
13654pb
Chimera
7pb
4709pb
Chimera
Different Scaffold/Contig
XXpb
Colineal genes
XXpb
Putative Operons
Chimera
Chimeric Gene Models
SL Insertion
xN
Number Repeats
Overlap with other gene
N0.HOG0010740
N0.HOG0015684
N0.HOG0011037

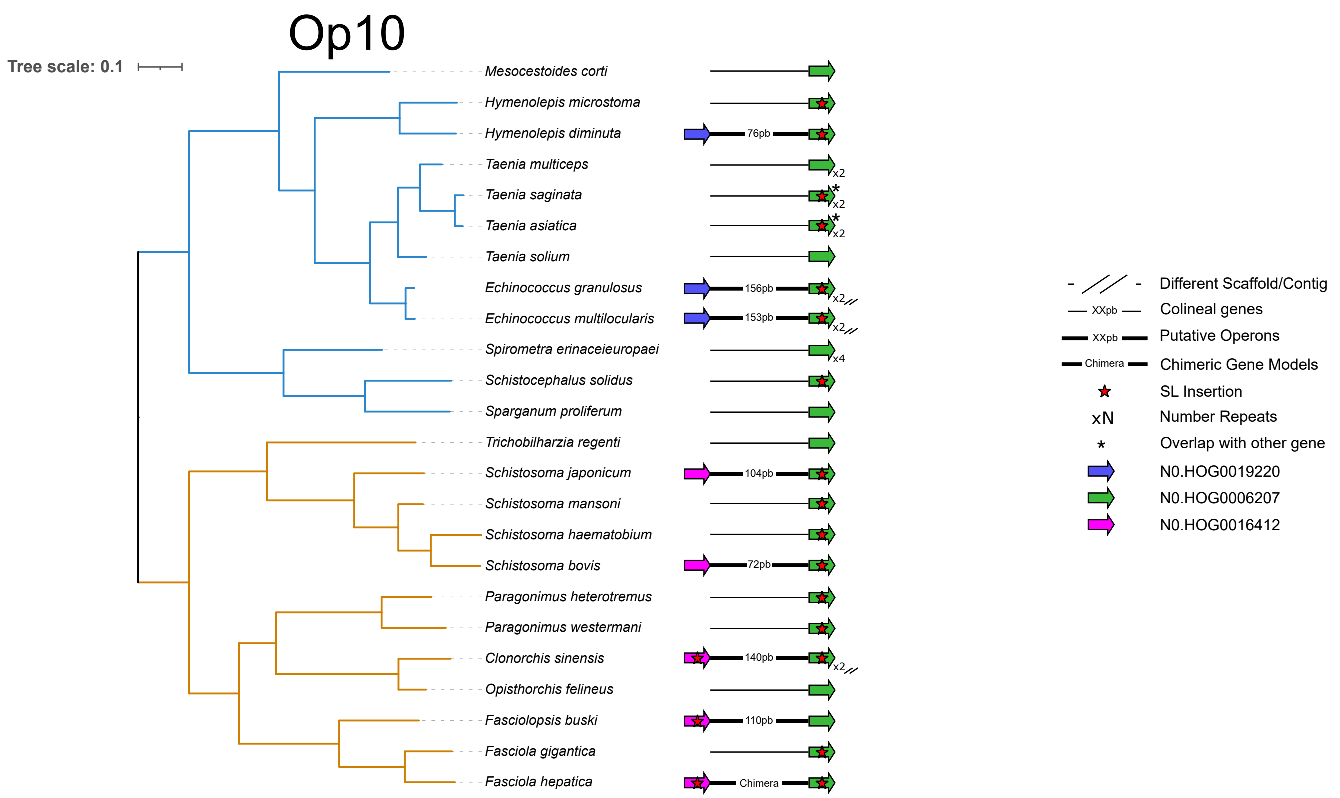
Op10
Tree scale: 0.1
Mesocestoides corti
Hymenolepis microstoma
Hymenolepis diminuta
76pb
Taenia multiceps
x2
Taenia saginata
*
x2
Taenia asiatica
*
x2
Taenia solium
Echinococcus granulosus
156pb
x2
Echinococcus multilocularis
153pb
x2
Spirometra erinaceieuropaei
x4
Schistocephalus solidus
Sparganum proliferum
Trichobilharzia regenti
Schistosoma japonicum
104pb
Schistosoma mansoni
Schistosoma haematobium
Schistosoma bovis
72pb
Paragonimus heterotremus
Paragonimus westermani
Clonorchis sinensis
140pb
x2
Opisthorchis felineus
Fasciolopsis buski
110pb
Fasciola gigantica
Fasciola hepatica
Chimera
Different Scaffold/Contig
XXpb
Colineal genes
XXpb
Putative Operons
Chimera
Chimeric Gene Models
SL Insertion
xN
Number Repeats
*
Overlap with other gene
N0.HOG0019220
N0.HOG0006207
N0.HOG0016412

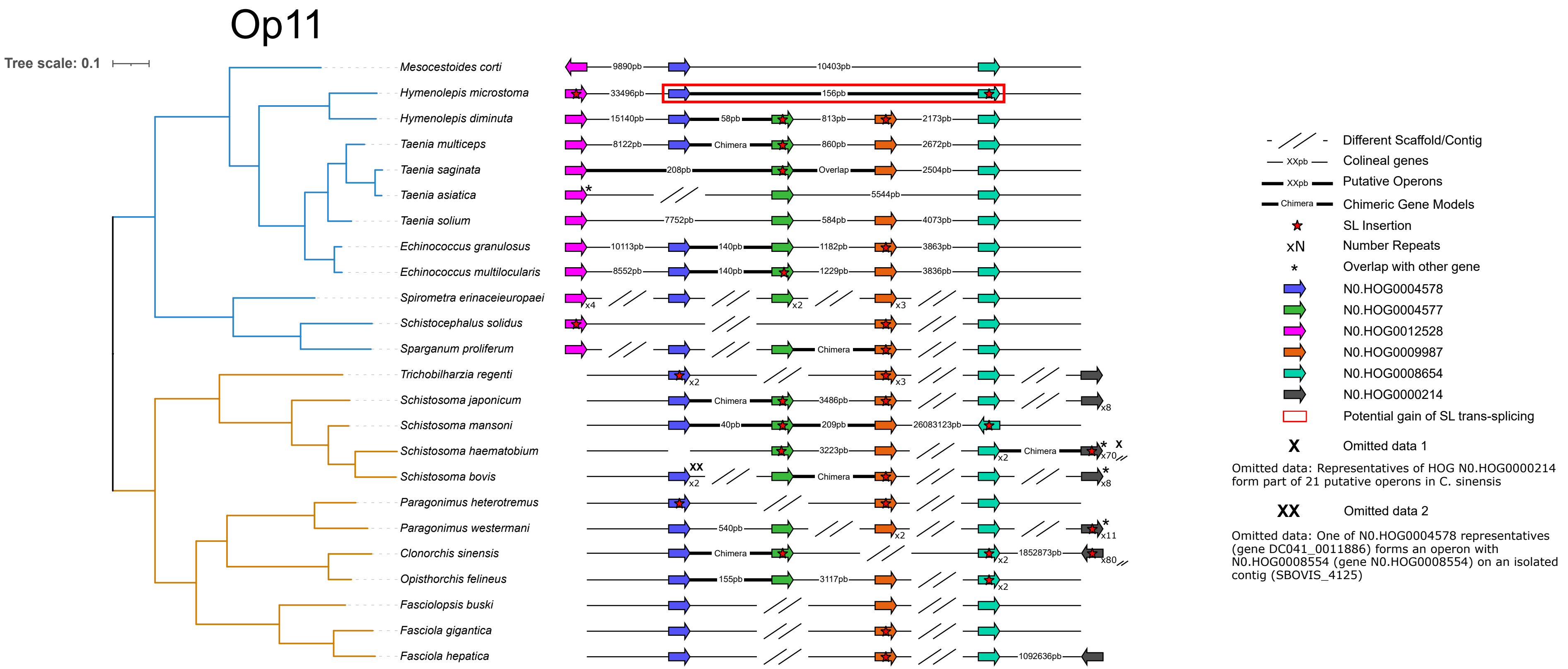

Op11
Tree scale: 0.1
Mesocestoides corti
Hymenolepis microstoma
Hymenolepis diminuta
Taenia multiceps
Taenia saginata
Taenia asiatica
Taenia solium
Echinococcus granulosus
Echinococcus multilocularis
Spirometra erinaceieuropaei
Schistocephalus solidus
Sparganum proliferum
Trichobilharzia regenti
Schistosoma japonicum
Schistosoma mansoni
Schistosoma haematobium
Schistosoma bovis
Paragonimus heterotremus
Paragonimus westermani
Clonorchis sinensis
Opisthorchis felineus
Fasciolopsis buski
Fasciola gigantica
Fasciola hepatica
Different Scaffold/Contig
Colineal genes
Putative Operons
Chimeric Gene Models
SL Insertion
xN
Number Repeats
Overlap with other gene
N0.HOG0004578
N0.HOG0004577
N0.HOG0012528
N0.HOG0009987
N0.HOG0008654
N0.HOG0000214
Potential gain of SL trans-splicing
X
Omitted data 1
Omitted data: Representatives of HOG N0.HOG0000214 form part of 21 putative operons in C. sinensis
XX
Omitted data 2
Omitted data: One of N0.HOG0004578 representatives (gene DC041_0011886) forms an operon with N0.HOG0008554 (gene N0.HOG0008554) on an isolated contig (SBOVIS_4125)

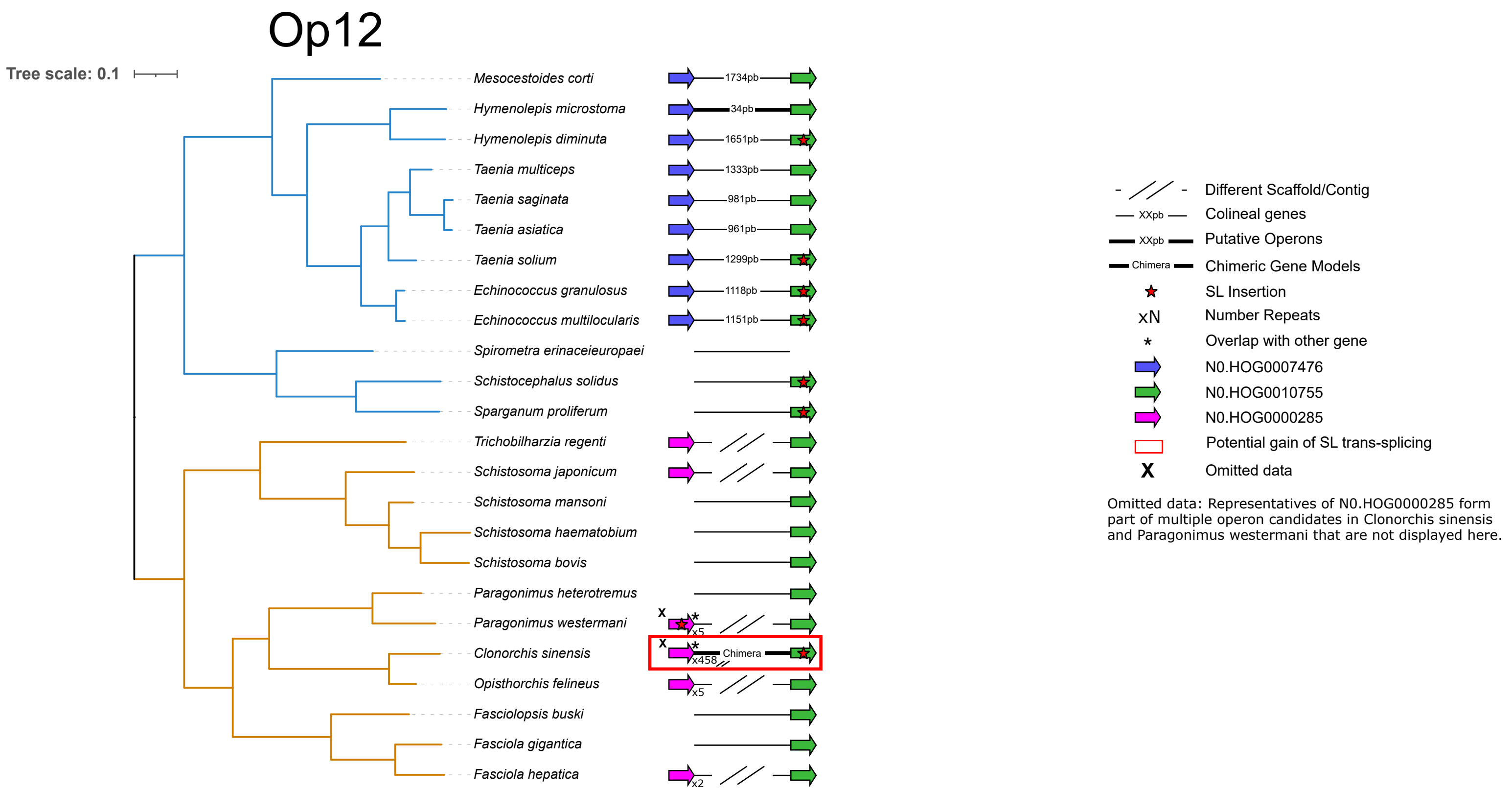

Omitted data: Representatives of N0.HOG0000285 form part of multiple operon candidates in Clonorchis sinensis and Paragonimus westermani that are not displayed here.

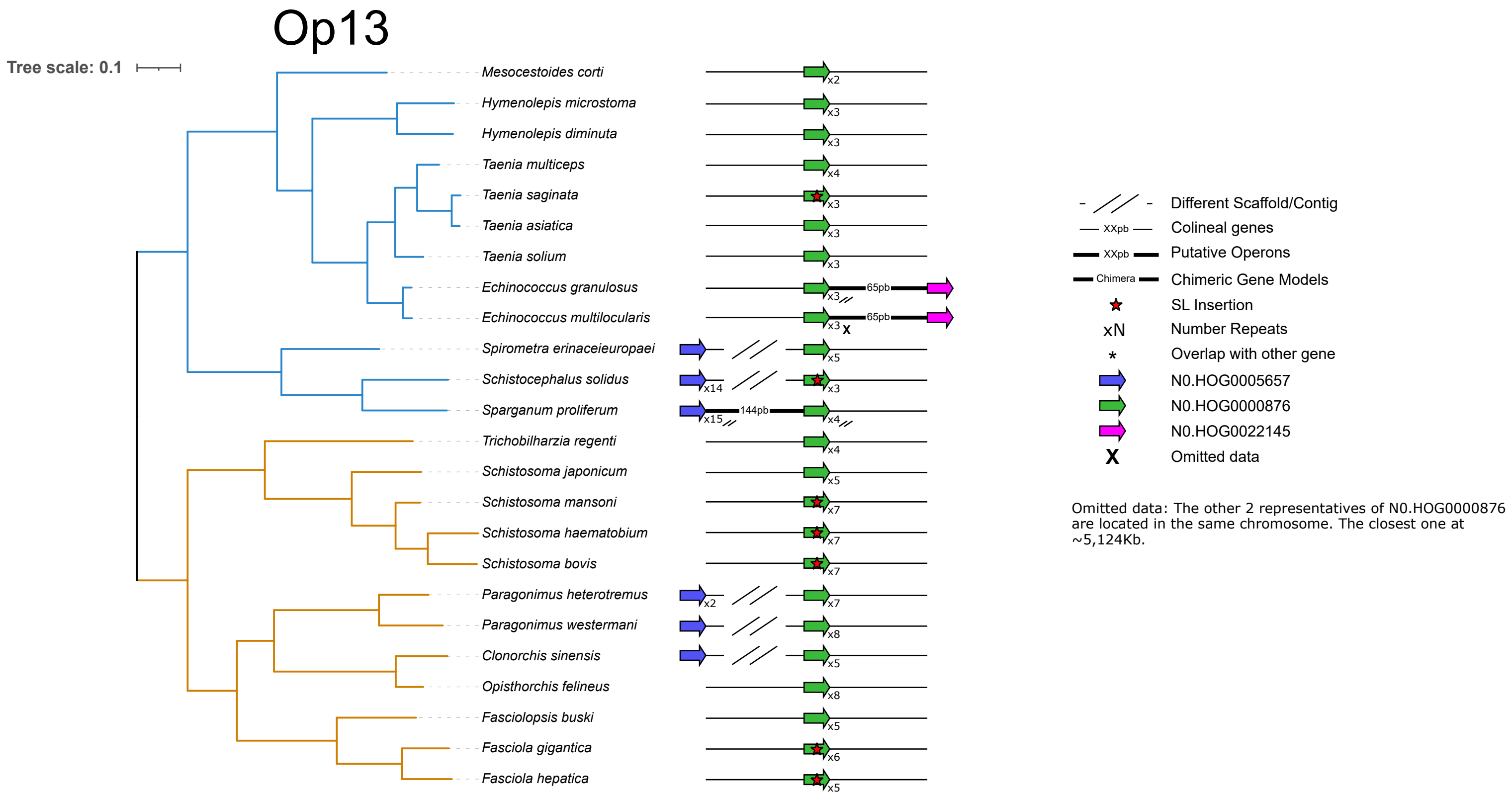

Op13
Tree scale: 0.1
Mesocestoides corti
Hymenolepis microstoma
Hymenolepis diminuta
Taenia multiceps
Taenia saginata
Taenia asiatica
Taenia solium
Echinococcus granulosus
Echinococcus multilocularis
Spirometra erinaceieuropaei
Schistocephalus solidus
Sparganum proliferum
Trichobilharzia regenti
Schistosoma japonicum
Schistosoma mansoni
Schistosoma haematobium
Schistosoma bovis
Paragonimus heterotremus
Paragonimus westermani
Clonorchis sinensis
Opisthorchis felineus
Fasciolopsis buski
Fasciola gigantica
Fasciola hepatica
65pb
144pb
Different Scaffold/Contig
Colineal genes
Putative Operons
Chimeric Gene Models
SL Insertion
xN
Number Repeats
Overlap with other gene
N0.HOG0005657
N0.HOG0000876
N0.HOG0022145
X
Omitted data
Omitted data: The other 2 representatives of N0.HOG0000876 are located in the same chromosome. The closest one at ~5,124Kb.

Op14

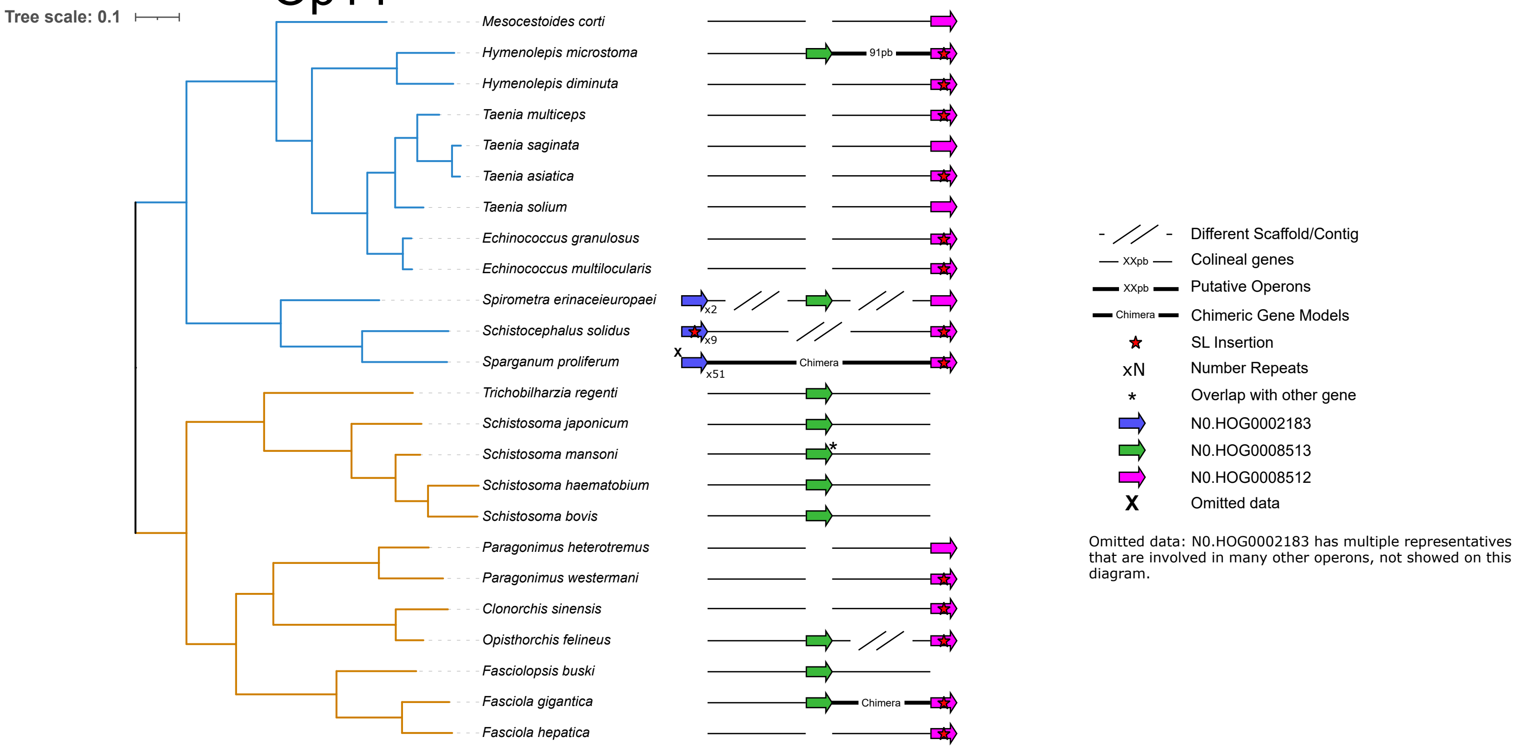

Tree scale: 0.1
Mesocestoides corti
Hymenolepis microstoma
91pb
Hymenolepis diminuta
Taenia multiceps
Taenia saginata
Taenia asiatica
Taenia solium
Echinococcus granulosus
Echinococcus multilocularis
Spirometra erinaceieuropaei
x2
Schistocephalus solidus
x9
Sparganum proliferum
X
x51
Chimera
Trichobilharzia regenti
Schistosoma japonicum
Schistosoma mansoni
*
Schistosoma haematobium
Schistosoma bovis
Paragonimus heterotremus
Paragonimus westermani
Clonorchis sinensis
Opisthorchis felineus
Fasciolopsis buski
Fasciola gigantica
Chimera
Fasciola hepatica
Different Scaffold/Contig
XXpb
Colineal genes
XXpb
Putative Operons
Chimera
Chimeric Gene Models
SL Insertion
xN
Number Repeats
*
Overlap with other gene
N0.HOG0002183
N0.HOG0008513
N0.HOG0008512
X
Omitted data
Omitted data: N0.HOG0002183 has multiple representatives that are involved in many other operons, not showed on this diagram.

Op15

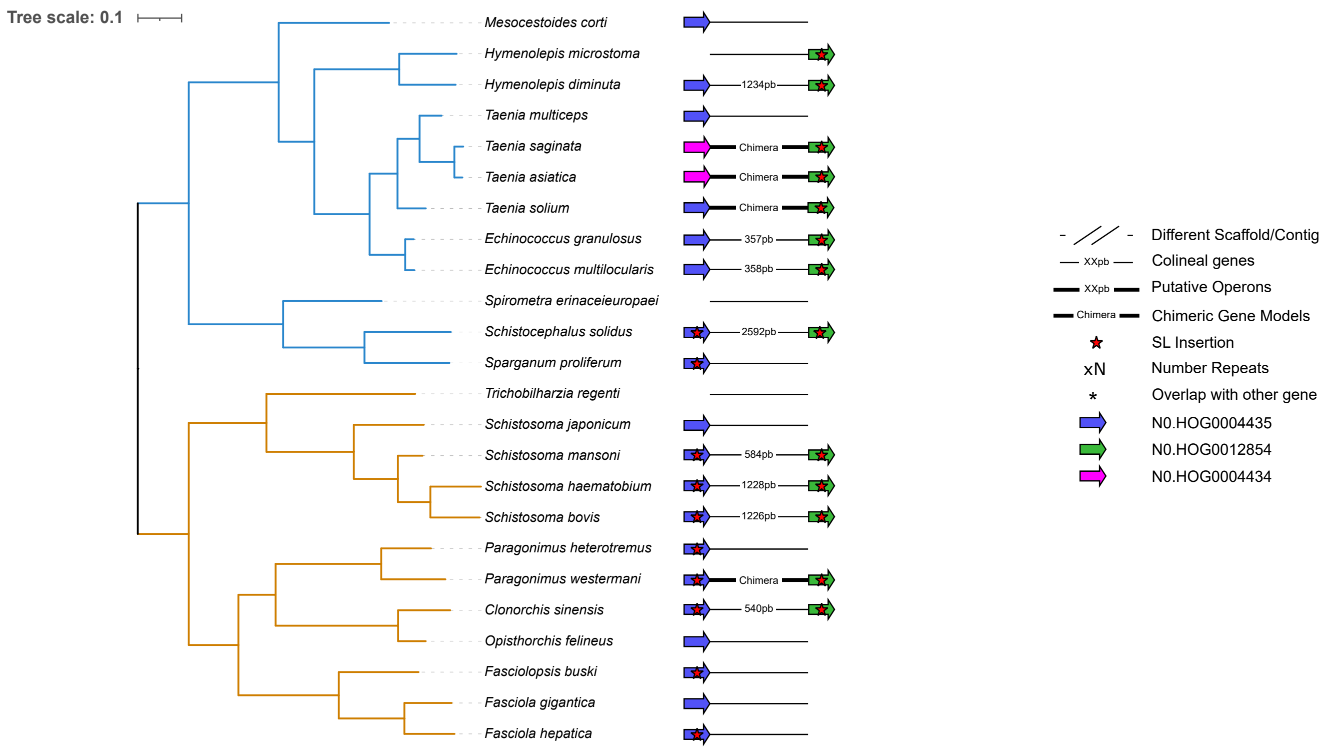

Tree scale: 0.1
Mesocestoides corti
Hymenolepis microstoma
Hymenolepis diminuta
1234pb
Taenia multiceps
Taenia saginata
Chimera
Taenia asiatica
Chimera
Taenia solium
Chimera
Echinococcus granulosus
357pb
Echinococcus multilocularis
358pb
Spirometra erinaceieuropaei
Schistocephalus solidus
2592pb
Sparganum proliferum
Trichobilharzia regenti
Schistosoma japonicum
Schistosoma mansoni
584pb
Schistosoma haematobium
1228pb
Schistosoma bovis
1226pb
Paragonimus heterotremus
Paragonimus westermani
Chimera
Clonorchis sinensis
540pb
Opisthorchis felineus
Fasciolopsis buski
Fasciola gigantica
Fasciola hepatica
Different Scaffold/Contig
XXpb
Colineal genes
XXpb
Putative Operons
Chimera
Chimeric Gene Models
SL Insertion
xN
Number Repeats
*
Overlap with other gene
N0.HOG0004435
N0.HOG0012854
N0.HOG0004434

Op16

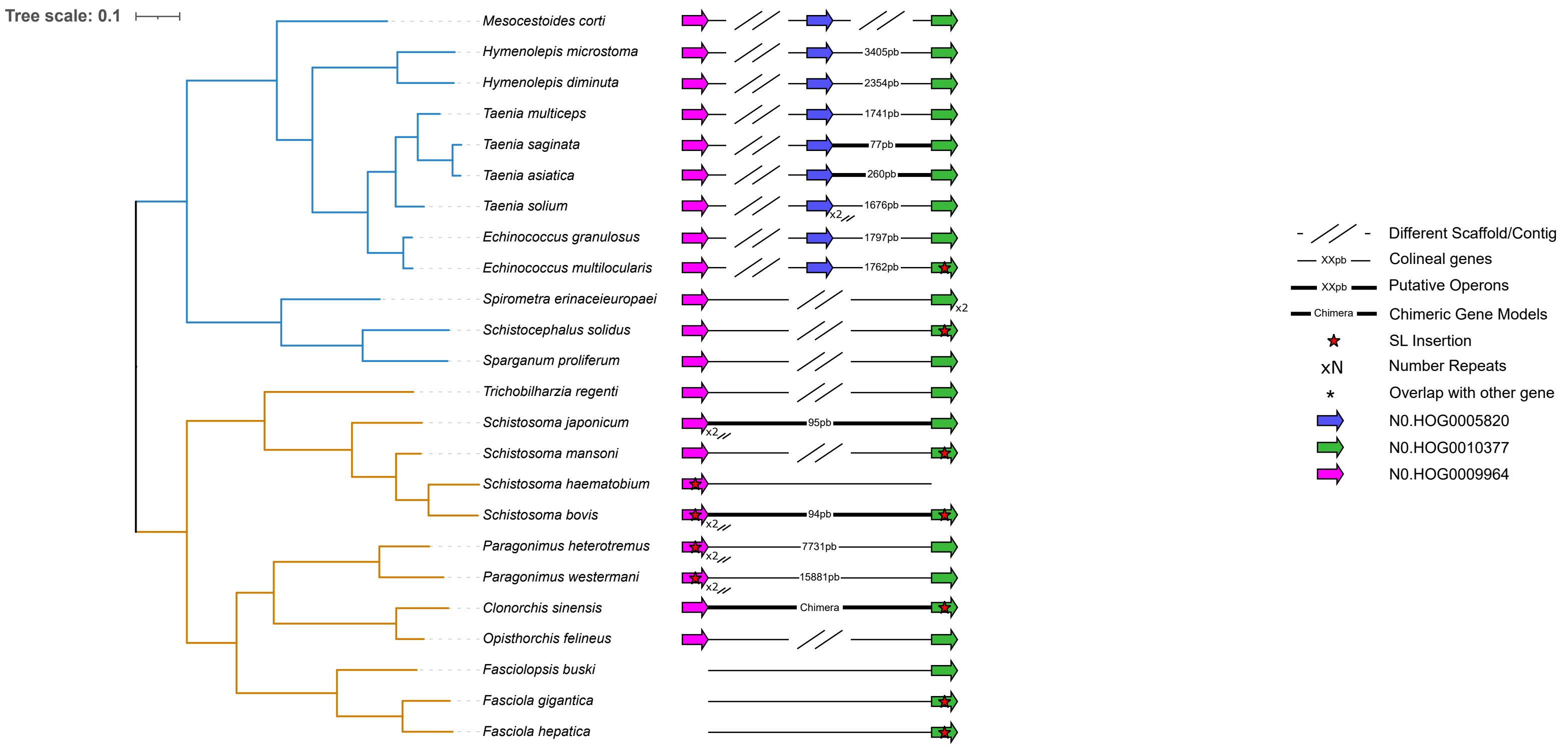

Tree scale: 0.1
Mesocestoides corti
Hymenolepis microstoma
Hymenolepis diminuta
Taenia multiceps
Taenia saginata
Taenia asiatica
Taenia solium
Echinococcus granulosus
Echinococcus multilocularis
Spirometra erinaceieuropaei
Schistocephalus solidus
Sparganum proliferum
Trichobilharzia regenti
Schistosoma japonicum
Schistosoma mansoni
Schistosoma haematobium
Schistosoma bovis
Paragonimus heterotremus
Paragonimus westermani
Clonorchis sinensis
Opisthorchis felineus
Fasciolopsis buski
Fasciola gigantica
Fasciola hepatica
3405pb
2354pb
1741pb
77pb
260pb
1676pb
1797pb
1762pb
95pb
94pb
7731pb
15881pb
Chimera
x2
Different Scaffold/Contig
XXpb
Colineal genes
XXpb
Putative Operons
Chimera
Chimeric Gene Models
SL Insertion
xN
Number Repeats
*
Overlap with other gene
N0.HOG0005820
N0.HOG0010377
N0.HOG0009964

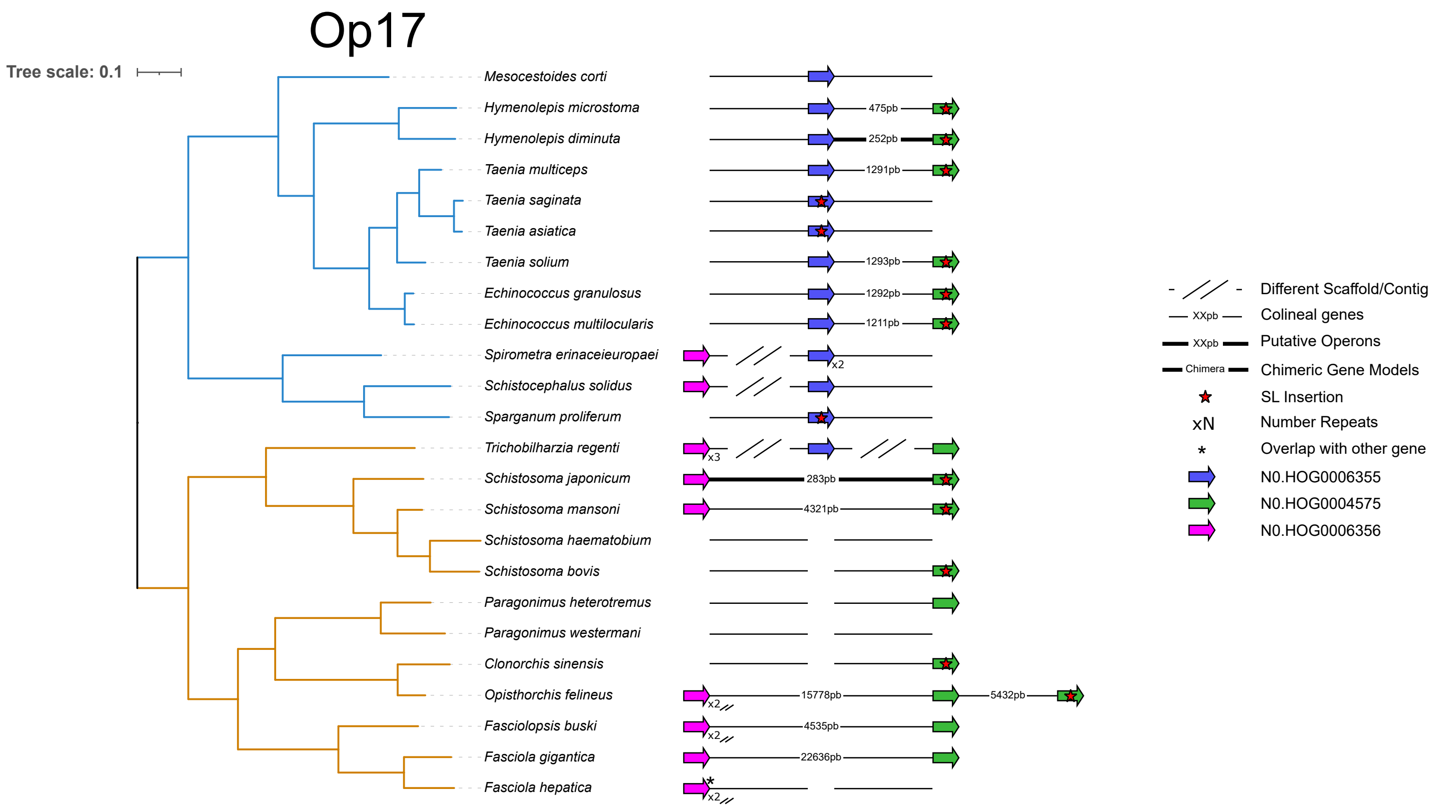
Op17
Tree scale: 0.1
Mesocestoides corti
Hymenolepis microstoma
Hymenolepis diminuta
Taenia multiceps
Taenia saginata
Taenia asiatica
Taenia solium
Echinococcus granulosus
Echinococcus multilocularis
Spirometra erinaceieuropaei
Schistocephalus solidus
Sparganum proliferum
Trichobilharzia regenti
Schistosoma japonicum
Schistosoma mansoni
Schistosoma haematobium
Schistosoma bovis
Paragonimus heterotremus
Paragonimus westermani
Clonorchis sinensis
Opisthorchis felineus
Fasciolopsis buski
Fasciola gigantica
Fasciola hepatica
475pb
252pb
1291pb
1293pb
1292pb
1211pb
x2
x3
283pb
4321pb
15778pb
5432pb
4535pb
22636pb
Different Scaffold/Contig
XXpb
Colineal genes
Putative Operons
Chimera
Chimeric Gene Models
SL Insertion
xN
Number Repeats
*
Overlap with other gene
N0.HOG0006355
N0.HOG0004575
N0.HOG0006356

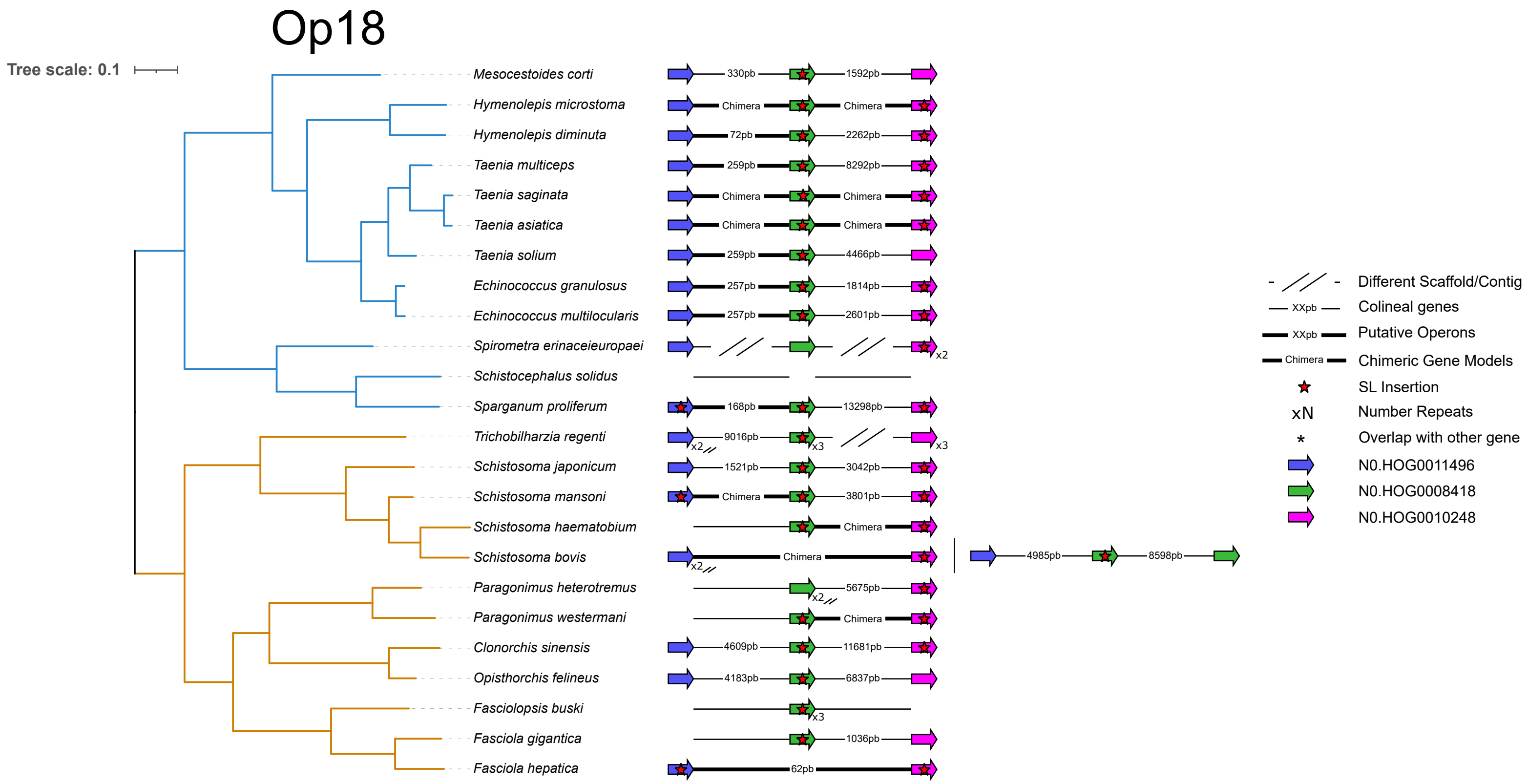
Op18
Tree scale: 0.1
Mesocestoides corti
Hymenolepis microstoma
Hymenolepis diminuta
Taenia multiceps
Taenia saginata
Taenia asiatica
Taenia solium
Echinococcus granulosus
Echinococcus multilocularis
Spirometra erinaceieuropaei
Schistocephalus solidus
Sparganum proliferum
Trichobilharzia regenti
Schistosoma japonicum
Schistosoma mansoni
Schistosoma haematobium
Schistosoma bovis
Paragonimus heterotremus
Paragonimus westermani
Clonorchis sinensis
Opisthorchis felineus
Fasciolopsis buski
Fasciola gigantica
Fasciola hepatica
330pb
1592pb
Chimera
72pb
2262pb
259pb
8292pb
4466pb
257pb
1814pb
2601pb
x2
168pb
13298pb
9016pb
x3
1521pb
3042pb
3801pb
4985pb
8598pb
5675pb
4609pb
11681pb
4183pb
6837pb
1036pb
62pb
Different Scaffold/Contig
XXpb
Colineal genes
Putative Operons
Chimeric Gene Models
SL Insertion
xN
Number Repeats
Overlap with other gene
N0.HOG0011496
N0.HOG0008418
N0.HOG0010248

Op19

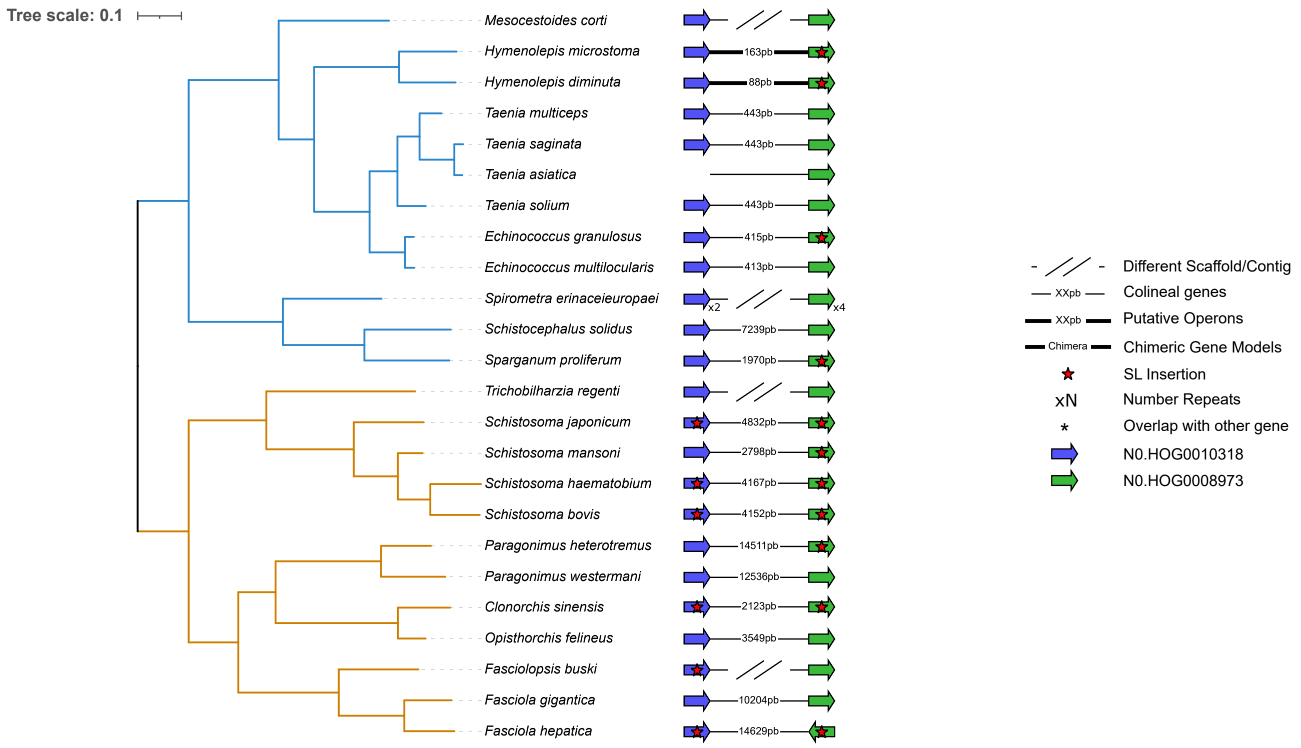

Tree scale: 0.1
Mesocestoides corti
Hymenolepis microstoma
163pb
Hymenolepis diminuta
88pb
Taenia multiceps
443pb
Taenia saginata
443pb
Taenia asiatica
Taenia solium
443pb
Echinococcus granulosus
415pb
Echinococcus multilocularis
413pb
Spirometra erinaceieuropaei
x2
x4
Schistocephalus solidus
7239pb
Sparganum proliferum
1970pb
Trichobilharzia regenti
Schistosoma japonicum
4832pb
Schistosoma mansoni
2798pb
Schistosoma haematobium
4167pb
Schistosoma bovis
4152pb
Paragonimus heterotremus
14511pb
Paragonimus westermani
12536pb
Clonorchis sinensis
2123pb
Opisthorchis felineus
3549pb
Fasciolopsis buski
Fasciola gigantica
10204pb
Fasciola hepatica
14629pb
Different Scaffold/Contig
XXpb
Colineal genes
XXpb
Putative Operons
Chimera
Chimeric Gene Models
SL Insertion
xN
Number Repeats
*
Overlap with other gene
N0.HOG0010318
N0.HOG0008973

Op20

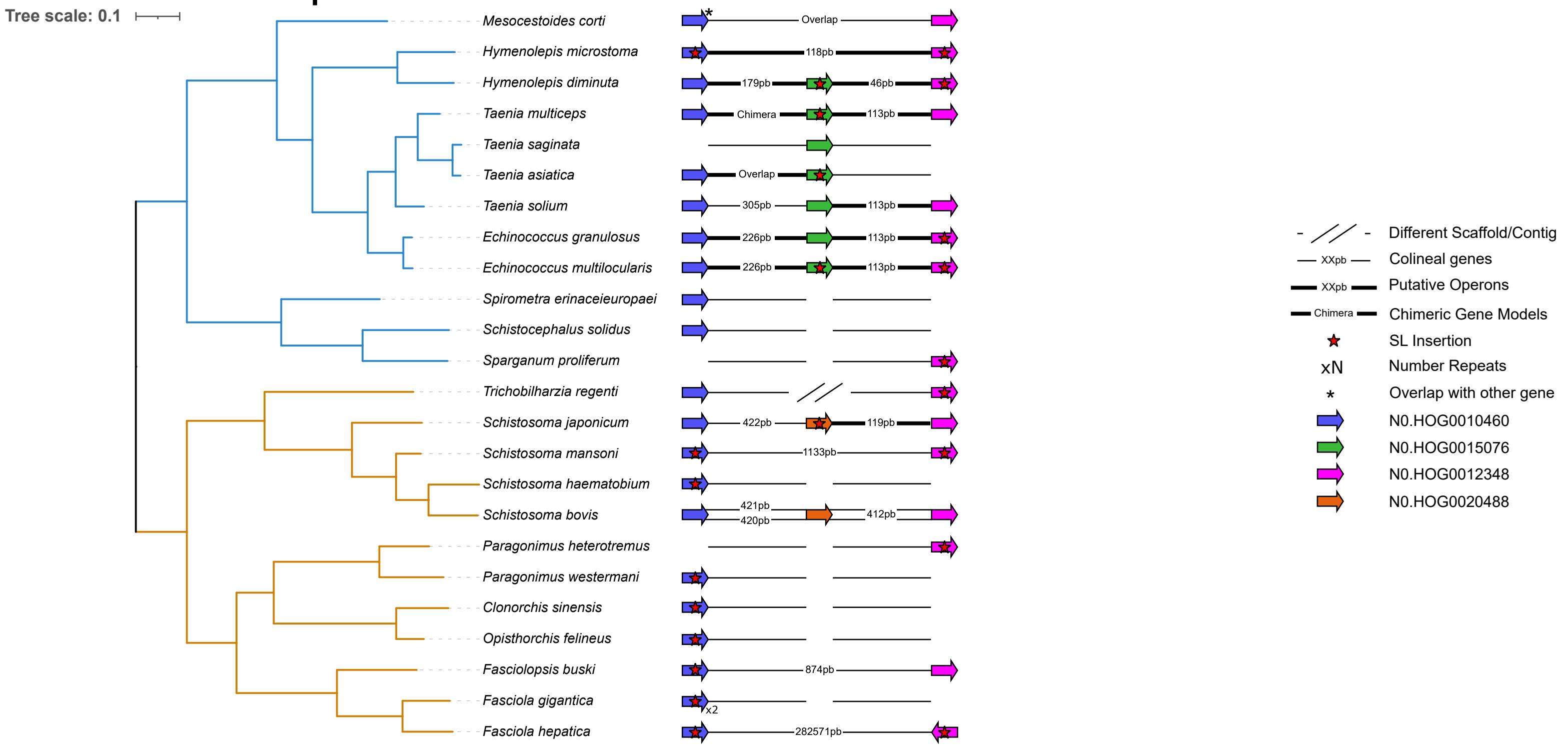

Tree scale: 0.1
Mesocestoides corti
Hymenolepis microstoma
Hymenolepis diminuta
Taenia multiceps
Taenia saginata
Taenia asiatica
Taenia solium
Echinococcus granulosus
Echinococcus multilocularis
Spirometra erinaceieuropaei
Schistocephalus solidus
Sparganum proliferum
Trichobilharzia regenti
Schistosoma japonicum
Schistosoma mansoni
Schistosoma haematobium
Schistosoma bovis
Paragonimus heterotremus
Paragonimus westermani
Clonorchis sinensis
Opisthorchis felineus
Fasciolopsis buski
Fasciola gigantica
Fasciola hepatica
Overlap
118pb
179pb
46pb
Chimera
113pb
Overlap
305pb
113pb
226pb
113pb
226pb
113pb
422pb
119pb
1133pb
421pb
420pb
412pb
874pb
x2
282571pb
Different Scaffold/Contig
XXpb
Colineal genes
XXpb
Putative Operons
Chimera
Chimeric Gene Models
SL Insertion
xN
Number Repeats
*
Overlap with other gene
N0.HOG0010460
N0.HOG0015076
N0.HOG0012348
N0.HOG0020488

Op21

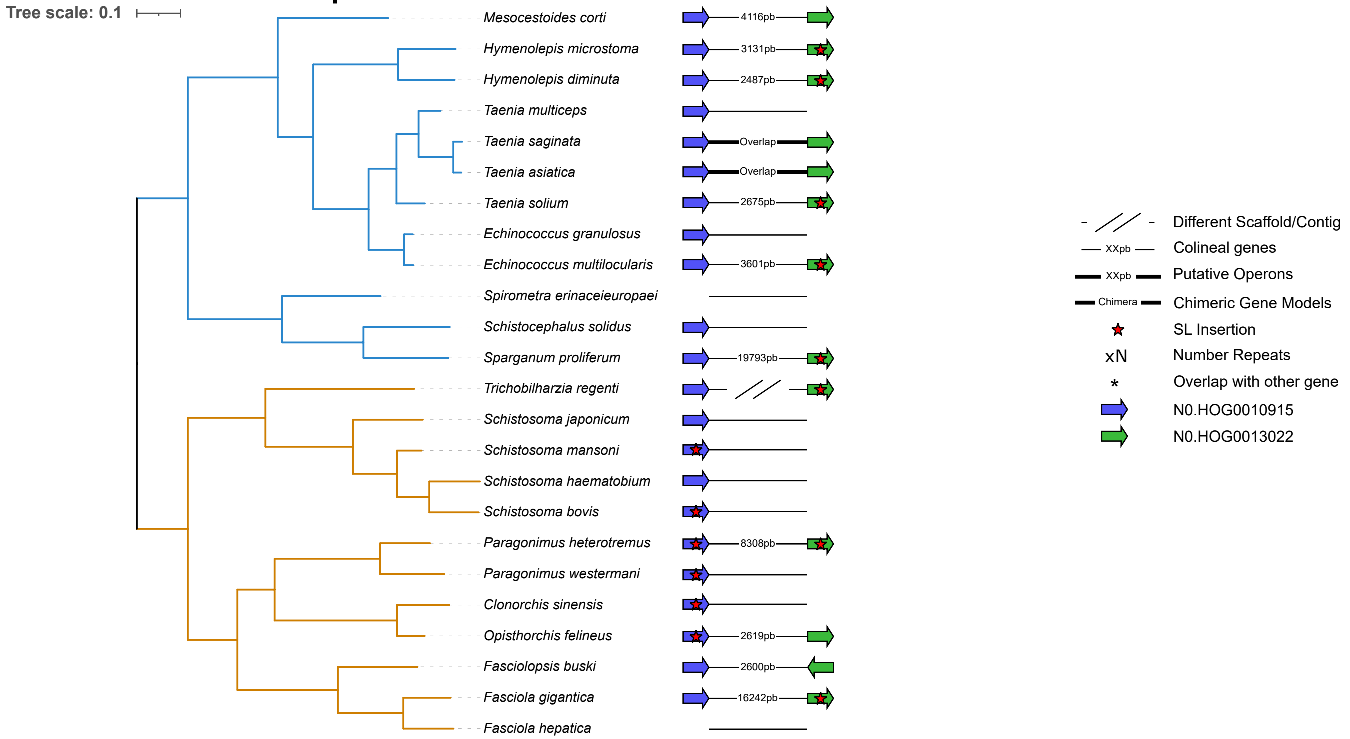
Tree scale: 0.1
Mesocestoides corti
4116pb
Hymenolepis microstoma
3131pb
Hymenolepis diminuta
2487pb
Taenia multiceps
Taenia saginata
Overlap
Taenia asiatica
Overlap
Taenia solium
2675pb
Echinococcus granulosus
Echinococcus multilocularis
3601pb
Spirometra erinaceieuropaei
Schistocephalus solidus
Sparganum proliferum
19793pb
Trichobilharzia regenti
Schistosoma japonicum
Schistosoma mansoni
Schistosoma haematobium
Schistosoma bovis
Paragonimus heterotremus
8308pb
Paragonimus westermani
Clonorchis sinensis
Opisthorchis felineus
2619pb
Fasciolopsis buski
2600pb
Fasciola gigantica
16242pb
Fasciola hepatica
Different Scaffold/Contig
XXpb
Colineal genes
XXpb
Putative Operons
Chimera
Chimeric Gene Models
SL Insertion
xN
Number Repeats
*
Overlap with other gene
N0.HOG0010915
N0.HOG0013022

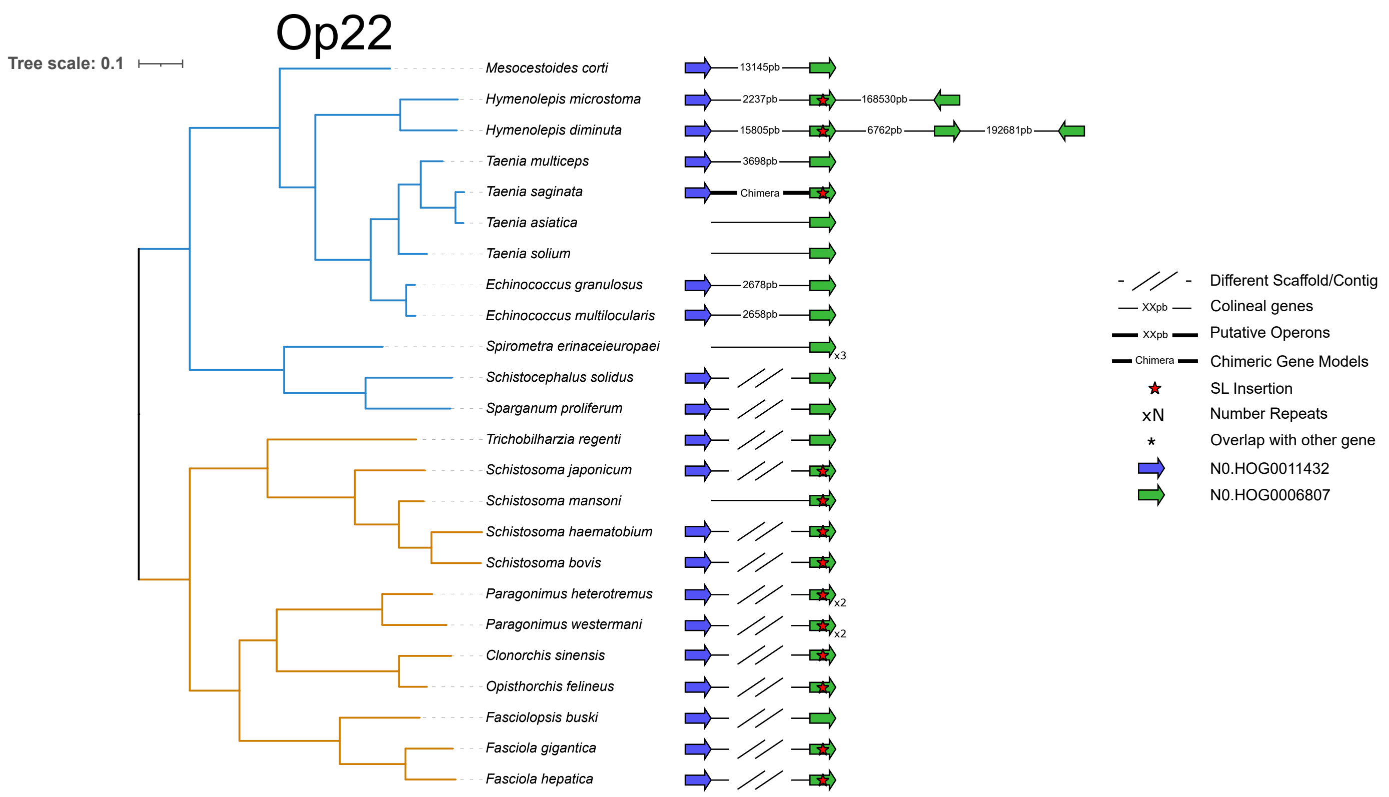

Op22
Tree scale: 0.1
Mesocestoides corti
Hymenolepis microstoma
Hymenolepis diminuta
Taenia multiceps
Taenia saginata
Taenia asiatica
Taenia solium
Echinococcus granulosus
Echinococcus multilocularis
Spirometra erinaceieuropaei
Schistocephalus solidus
Sparganum proliferum
Trichobilharzia regenti
Schistosoma japonicum
Schistosoma mansoni
Schistosoma haematobium
Schistosoma bovis
Paragonimus heterotremus
Paragonimus westermani
Clonorchis sinensis
Opisthorchis felineus
Fasciolopsis buski
Fasciola gigantica
Fasciola hepatica
13145pb
2237pb
168530pb
15805pb
6762pb
192681pb
3698pb
Chimera
2678pb
2658pb
x3
x2
x2
Different Scaffold/Contig
XXpb
Colineal genes
XXpb
Putative Operons
Chimera
Chimeric Gene Models
SL Insertion
xN
Number Repeats
Overlap with other gene
N0.HOG0011432
N0.HOG0006807

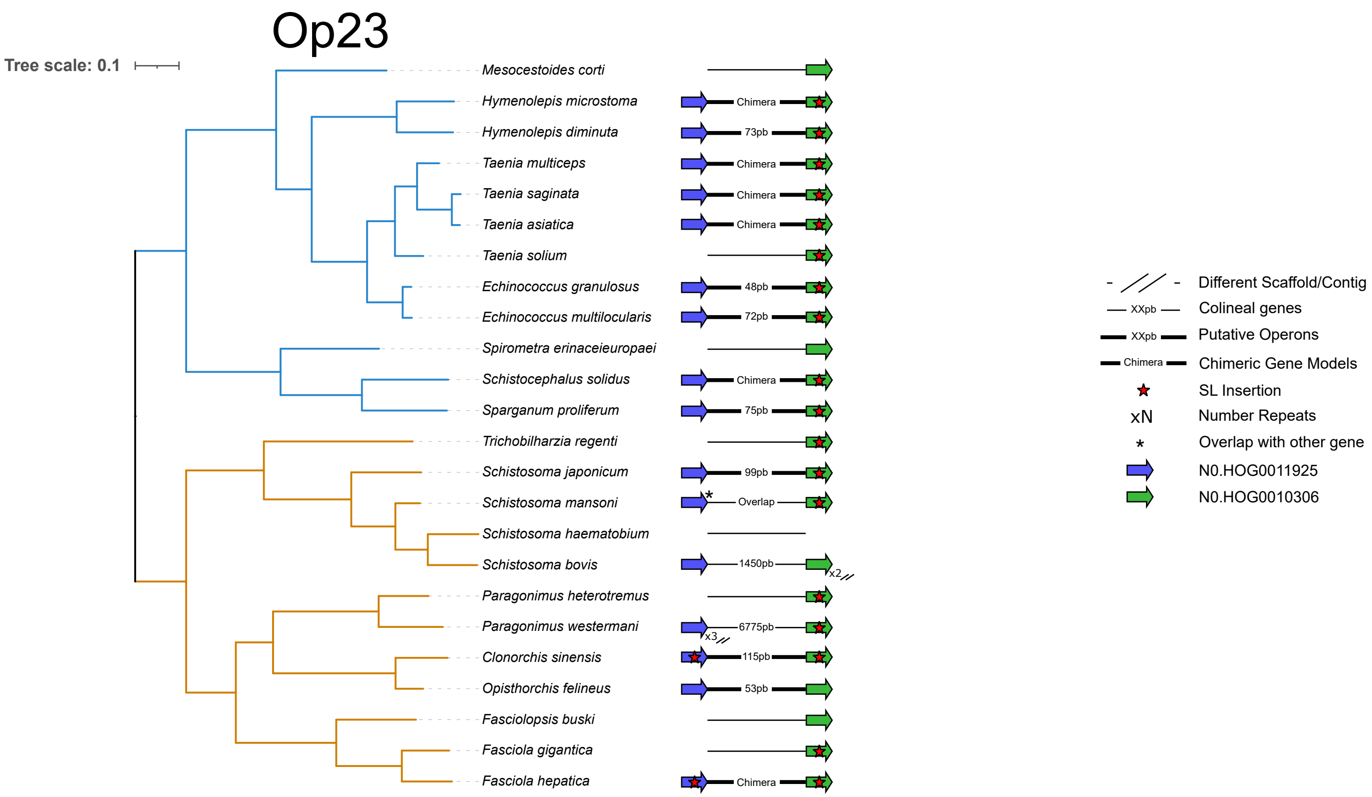

Op23
Tree scale: 0.1
Mesocestoides corti
Hymenolepis microstoma
Chimera
Hymenolepis diminuta
73pb
Taenia multiceps
Chimera
Taenia saginata
Chimera
Taenia asiatica
Chimera
Taenia solium
Echinococcus granulosus
48pb
Echinococcus multilocularis
72pb
Spirometra erinaceieuropaei
Schistocephalus solidus
Chimera
Sparganum proliferum
75pb
Trichobilharzia regenti
Schistosoma japonicum
99pb
Schistosoma mansoni
Overlap
Schistosoma haematobium
Schistosoma bovis
1450pb
x2
Paragonimus heterotremus
Paragonimus westermani
6775pb
x3
Clonorchis sinensis
115pb
Opisthorchis felineus
53pb
Fasciolopsis buski
Fasciola gigantica
Fasciola hepatica
Chimera
Different Scaffold/Contig
XXpb
Colineal genes
XXpb
Putative Operons
Chimera
Chimeric Gene Models
SL Insertion
xN
Number Repeats
*
Overlap with other gene
N0.HOG0011925
N0.HOG0010306

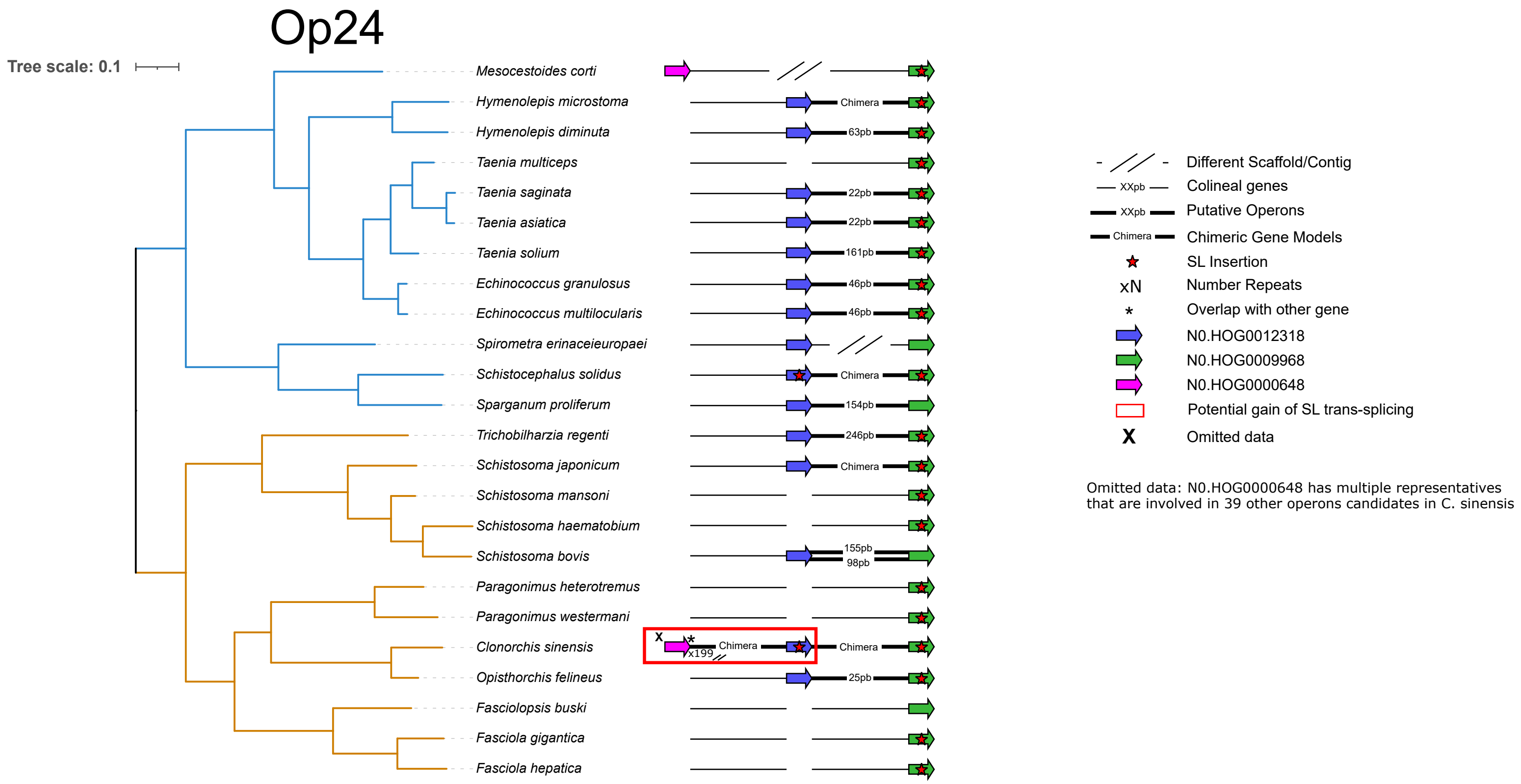

Op24
Tree scale: 0.1
Mesocestoides corti
Hymenolepis microstoma
Hymenolepis diminuta
Taenia multiceps
Taenia saginata
Taenia asiatica
Taenia solium
Echinococcus granulosus
Echinococcus multilocularis
Spirometra erinaceieuropaei
Schistocephalus solidus
Sparganum proliferum
Trichobilharzia regenti
Schistosoma japonicum
Schistosoma mansoni
Schistosoma haematobium
Schistosoma bovis
Paragonimus heterotremus
Paragonimus westermani
Clonorchis sinensis
Opisthorchis felineus
Fasciolopsis buski
Fasciola gigantica
Fasciola hepatica
Chimera
63pb
22pb
22pb
161pb
46pb
46pb
Chimera
154pb
246pb
Chimera
155pb
98pb
X
x199
Chimera
Chimera
25pb
Different Scaffold/Contig
XXpb
Colineal genes
XXpb
Putative Operons
Chimera
Chimeric Gene Models
SL Insertion
xN
Number Repeats
Overlap with other gene
N0.HOG0012318
N0.HOG0009968
N0.HOG0000648
Potential gain of SL trans-splicing
X
Omitted data
Omitted data: N0.HOG0000648 has multiple representatives that are involved in 39 other operons candidates in C. sinensis

Op25

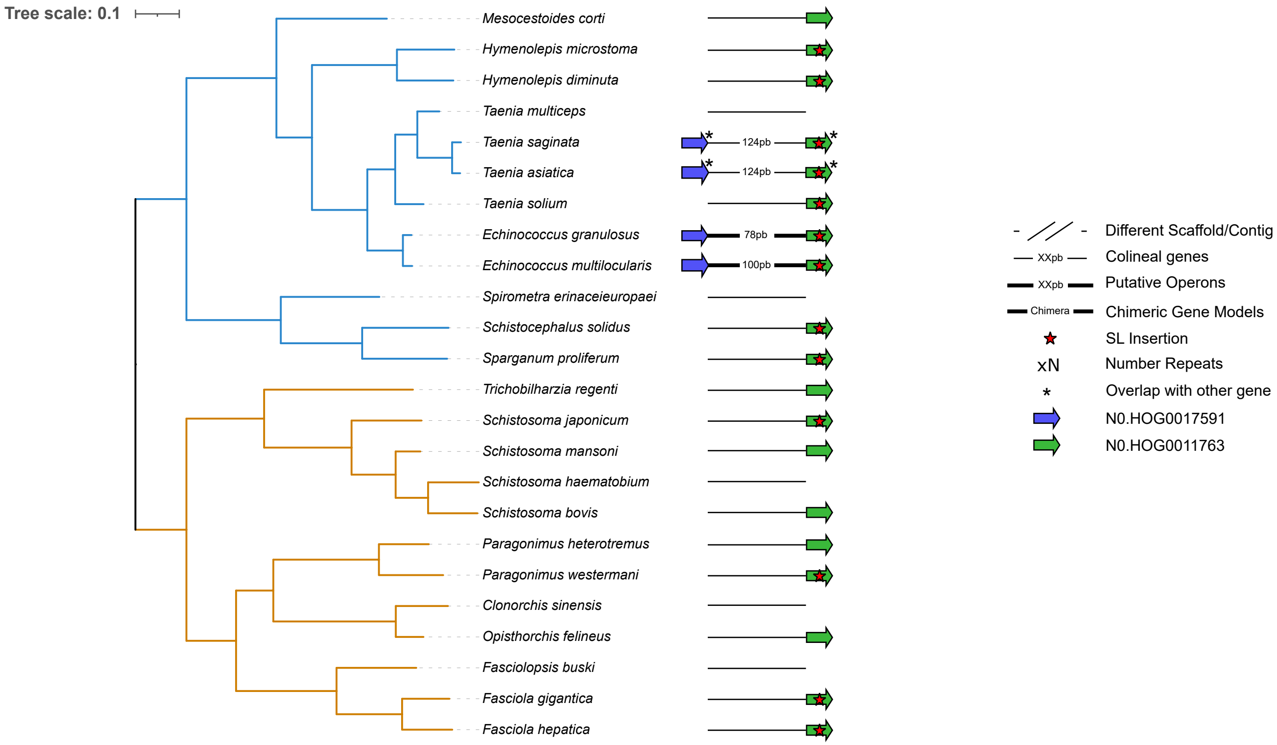
Tree scale: 0.1
Mesocestoides corti
Hymenolepis microstoma
Hymenolepis diminuta
Taenia multiceps
Taenia saginata
124pb
Taenia asiatica
124pb
Taenia solium
Echinococcus granulosus
78pb
Echinococcus multilocularis
100pb
Spirometra erinaceieuropaei
Schistocephalus solidus
Sparganum proliferum
Trichobilharzia regenti
Schistosoma japonicum
Schistosoma mansoni
Schistosoma haematobium
Schistosoma bovis
Paragonimus heterotremus
Paragonimus westermani
Clonorchis sinensis
Opisthorchis felineus
Fasciolopsis buski
Fasciola gigantica
Fasciola hepatica
Different Scaffold/Contig
XXpb
Colineal genes
XXpb
Putative Operons
Chimera
Chimeric Gene Models
SL Insertion
xN
Number Repeats
*
Overlap with other gene
N0.HOG0017591
N0.HOG0011763

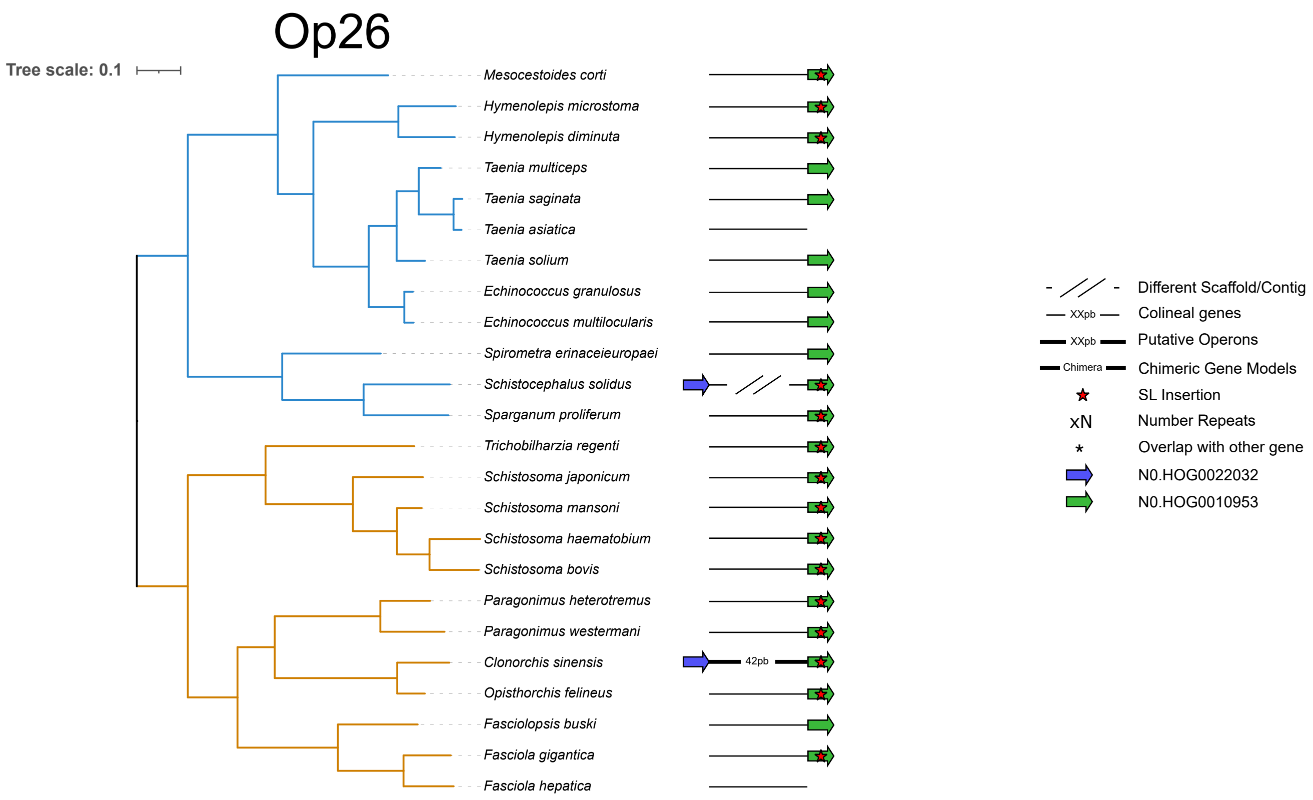

Op26
Tree scale: 0.1
Mesocestoides corti
Hymenolepis microstoma
Hymenolepis diminuta
Taenia multiceps
Taenia saginata
Taenia asiatica
Taenia solium
Echinococcus granulosus
Echinococcus multilocularis
Spirometra erinaceieuropaei
Schistocephalus solidus
Sparganum proliferum
Trichobilharzia regenti
Schistosoma japonicum
Schistosoma mansoni
Schistosoma haematobium
Schistosoma bovis
Paragonimus heterotremus
Paragonimus westermani
Clonorchis sinensis
Opisthorchis felineus
Fasciolopsis buski
Fasciola gigantica
Fasciola hepatica
42pb
Different Scaffold/Contig
XXpb
Colineal genes
XXpb
Putative Operons
Chimera
Chimeric Gene Models
SL Insertion
xN
Number Repeats
Overlap with other gene
N0.HOG0022032
N0.HOG0010953
